# Phosphoproteome of the cyanobacterium *Synechocystis* sp. PCC 6803 and its dynamics during nitrogen starvation

**DOI:** 10.3389/fmicb.2015.00248

**Published:** 2015-03-31

**Authors:** Philipp Spät, Boris Maček, Karl Forchhammer

**Affiliations:** ^1^Department of Organismic Interactions, Interfaculty Institute for Microbiology and Infection Medicine, Eberhard-Karls-University TübingenTübingen, Germany; ^2^Interfaculty Institute for Cell Biology, Proteome Center Tuebingen, Eberhard-Karls-University TübingenTübingen, Germany

**Keywords:** cyanobacteria, *Synechocystis* sp. PCC 6803, nitrogen starvation, phosphoproteome, peptide dimethylation labeling, LTQ-Orbitrap

## Abstract

Cyanobacteria have shaped the earth's biosphere as the first oxygenic photoautotrophs and still play an important role in many ecosystems. The ability to adapt to changing environmental conditions is an essential characteristic in order to ensure survival. To this end, numerous studies have shown that bacteria use protein post-translational modifications such as Ser/Thr/Tyr phosphorylation in cell signaling, adaptation, and regulation. Nevertheless, our knowledge of cyanobacterial phosphoproteomes and their dynamic response to environmental stimuli is relatively limited. In this study, we applied gel-free methods and high accuracy mass spectrometry toward the detection of Ser/Thr/Tyr phosphorylation events in the model cyanobacterium *Synechocystis* sp. PCC 6803. We could identify over 300 phosphorylation events in cultures grown on nitrate as exclusive nitrogen source. Chemical dimethylation labeling was applied to investigate proteome and phosphoproteome dynamics during nitrogen starvation. Our dataset describes the most comprehensive (phospho)proteome of *Synechocystis* to date, identifying 2382 proteins and 183 phosphorylation events and quantifying 2111 proteins and 148 phosphorylation events during nitrogen starvation. Global protein phosphorylation levels were increased in response to nitrogen depletion after 24 h. Among the proteins with increased phosphorylation, the P_II_ signaling protein showed the highest fold-change, serving as positive control. Other proteins with increased phosphorylation levels comprised functions in photosynthesis and in carbon and nitrogen metabolism. This study reveals dynamics of *Synechocystis* phosphoproteome in response to environmental stimuli and suggests an important role of protein Ser/Thr/Tyr phosphorylation in fundamental mechanisms of homeostatic control in cyanobacteria.

## Introduction

Cyanobacteria constitute one of the most widely distributed groups of prokaryotes in the biosphere, where they inhabit almost all illuminated environments. They are oxygenic photoautotrophs, employing photosynthetic machinery that resembles that of plants, due to the endosymbiotic origin of chloroplasts. The substrate diversity of cyanobacteria is limited since photoautotrophs only need H_2_O as an electron donor, CO_2_ as a carbon source, and inorganic salts such as nitrogen, phosphorous, sulfur, and iron to fulfill their anabolic needs. Among these, the highest demand is for nitrogen, whose cellular abundance amounts to a carbon to nitrogen ratio of 5:1.

**Figure 1:**
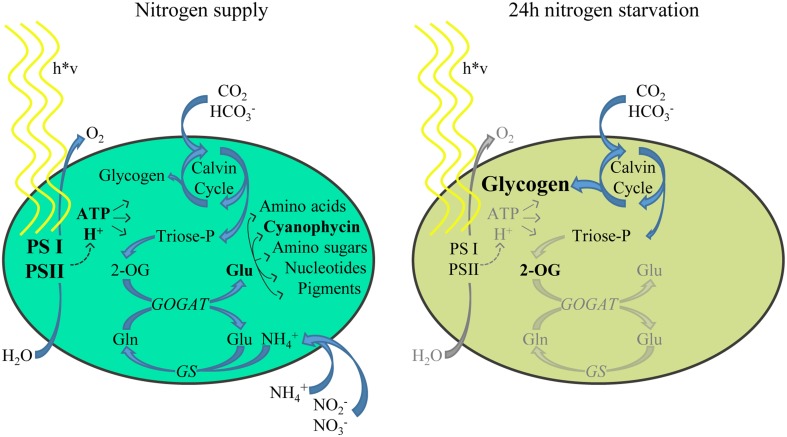
**Schematic representation of the major metabolic processes in nitrogen supplied cells (left) and nitrogen-starved cells (right)**. Due to the lack of combined nitrogen sources, glutamine synthetase (GS) cannot produce glutamine, and in consequence, glutamate synthase (GOGAT) reaction becomes substrate-limited, leading to increased 2-OG levels. Anabolic reactions toward nitrogen containing metabolites are tuned down and growth is arrested. The reduction equivalents generated by the light reactions of photosynthesis are now redirected toward glycogen synthesis, whose cellular abundance increases dramatically (Joseph et al., 2014).

Cyanobacteria encounter many environmental fluctuations, such as changing light conditions, nutrient availability and the ambient physico–chemical properties (osmolarity, temperature etc.). During evolution, these microbes have developed sophisticated regulator systems to adapt cellular processes and maintain metabolic homeostasis in response to these challenges. Nitrogen starvation induces in non-diazotrophic cyanobacteria a highly dynamic response, which can be classified in three phases. Their temporal occurrence depends on many environmental and intrinsic conditions (Schwarz and Forchhammer, 2005). In response to nitrogen depletion, an acclimation process is initiated that is known as chlorosis (Allen and Smith, 1969). Chlorosis is characterized by a rapid degradation of the phycobilisome antenna and a cell cycle arrest after completion of a final cell division (Allen and Smith, 1969; Collier and Grossman, 1992). This initial phase (phase I) is followed by a subsequent gradual and consecutive decrease in photosystem (PS) II and PSI activities (phase II), down-tuning of metabolic activities and proteolytic degradation of cellular proteins. Only after 10–15 days, cells have reached a final resting state, where they are completely bleached (phase III) and can survive for prolonged periods (Görl et al., 1998). Figure 1 indicates the metabolic changes in the cell in the phase II chlorosis stage, which starts after 24 h of nitrogen starvation.

Among the short-term acclimation processes, protein phosphorylation plays a prominent role (Biggins and Bruce, 1987; Stock et al., 1989; Hagemann et al., 1993). In bacteria, the two-component sensory transduction pathways have been historically known as the hallmark of bacterial signal transduction, involving protein histidine kinases and response regulators phosphorylated on aspartate residues. These are universally distributed and have been systematically investigated in several cyanobacterial strains (Ashby and Houmard, 2006). Among these, the 47 histidine kinases and 45 response regulators in the unicellular strain *Synechocystis* sp. PCC 6803 substrain Kazusa (hereafter designated as *Synechocystis*) has received the most thorough investigations (Murata and Suzuki, 2006).

Phosphorylation on serine, threonine and tyrosine residues (Ser/Thr/Tyr), referred to as S/T/Y phosphorylation or O-phosphorylation, was initially thought to occur exclusively in eukaryotic signal transduction. However, primarily due to advances in mass spectrometry proteomics technologies, it is now clear that this type of modification frequently occurs in bacteria serving an important role in prokaryotic signal transduction (Macek and Mijakovic, 2011). Most bacterial Ser/Thr kinases resemble those classified in eukaryotes and are termed as Hanks-like kinases, whereas, most bacterial tyrosine (BY)-kinases are unique to prokaryotes (Mijakovic and Macek, 2012; Soufi et al., 2012). Genome sequencing revealed that Hanks-like Ser/Thr kinases, BY-kinases, and protein phosphatases are globally distributed in the bacterial domain (Pereira et al., 2011). Bacterial S/T/Y phosphorylation is widely involved in signal transduction in response to environmental stimuli and regulates many physiological processes in a bacterial cell (Petranovic et al., 2007; Mijakovic and Macek, 2012).

A survey of the genome of *Synechocystis* revealed the presence of 11 Ser/Thr kinases, one Tyr kinase and seven protein phosphatases (Zhang et al., 2005). Pioneering work in the late 1980s and in the 1990s, employing *in vivo* and *in vitro* labeling experiments using [γ^32^P] phosphate shed the first light on the occurrence of protein phosphorylation events in cyanobacteria (reviewed by Mann, 1994). Protein phosphorylation was shown to be dynamic and to respond to a variety of environmental stimuli, such as changing illumination, nutrient supply, or osmolarity (Sanders et al., 1989; Hagemann et al., 1993).

One of the first discovered and most intensively studied phosphoproteins in cyanobacteria is the signal transduction protein P_II_ (Forchhammer and Tandeau De Marsac, 1994); reviewed in Forchhammer (2004, 2008). The P_II_ signaling protein, a homotrimer of 12.4 kDa, is phosphorylated at the tip of a large, surface exposed loop (termed T-loop) in response to the cellular nitrogen supply. The effector molecule 2-oxoglutarate (2-OG) whose abundance reflects the cellular state of nitrogen assimilation (Muro-Pastor et al., 2001), binds to P_II_ and elicits its phosphorylation on T-loop residue Ser49 (Forchhammer and Tandeau De Marsac, 1995). In its phosphorylated state, P_II_ is not able to bind to and activate the key enzyme of the ornithine/arginine biosynthesis pathway, N-acetyl-L-glutamate kinase (NAGK) (Heinrich et al., 2004). Shifting nitrogen-starved cells back to nitrogen-sufficient conditions, the cellular 2-OG level drops, leading to dephosphorylation of P_II_ (Irmler and Forchhammer, 2001) with a concomitant activation of NAGK (Maheswaran et al., 2006), thereby feeding nitrogen into the arginine pool.

In addition to P_II_, the functions of only a few other phosphoproteins have been identified in *Synechocystis*, such as the phosphorylation of phycobiliproteins and phycobilisome linker proteins (Harrison et al., 1991; Piven et al., 2005), or the oscillating phosphorylation of the circadian clock protein KaiC (Nishiwaki et al., 2004). Except these specific investigations focusing on particular phosphoproteins, Mikkat et al. recently published a snapshot of the phosphoproteome of *Synechocystis* based on 2D gel electrophoresis, where the authors identified about 30 phosphoproteins, of which the modified residue could be localized in eight cases. Among these, most previously identified phosphoproteins were included (Mikkat et al., 2014). However, the physiological function of most phosphorylation events remains still unclear.

In the recent years, the analysis of global phosphoproteomes has made dramatic progress due to the development of gel-free workflows and biochemical phosphopeptide enrichment strategies such as titanium dioxide (TiO_2_) chromatography coupled to high accuracy mass spectrometry. The development of chemical stable isotope labeling by dimethylation of tryptic peptides enables the application of a cost efficient and reliable quantification strategy also to autotrophic organisms, without the need of profound genetic manipulation of amino acid metabolic pathways (Boersema et al., 2009). Despite these advantages, the dimethylation approach was never applied in global phosphoproteomic studies in a prokaryotic organism.

The first global study of a cyanobacterial phosphoproteome using gel-free methods was performed in a qualitative manner in the marine unicellular cyanobacterium *Synechococcus* sp. PCC 7002, which resulted in the identification of 410 phosphorylation sites on 245 phosphoproteins (Yang et al., 2013). One general drawback in global studies in cyanobacterial organisms is the high percentage of integral membrane and membrane-associated proteins. Particularly, such membrane proteins are often involved in protein phosphorylation based signal transduction and represent a high potential for phosphorylation (Jers et al., 2008). Therefore, efficient extraction of cytosolic as well as of membrane proteins is essential in order to attain deep phosphoproteome coverage. So far, about 900 known or predicted membrane (associated) proteins have been identified in *Synechocystis* by previous large-scale studies (24.5% of 3672 protein coding genes list on Cyanobase). However, 52.7% of the total protein coding genes was thus far not identified on the proteome level, indicating a need for improvement in protein extraction methods, particularly with respect to hydrophobic (membrane)proteins (Wang et al., 2009).

In the present work, we applied state of the art mass spectrometric analysis to define the phosphoproteome of *Synechocystis*. To ensure an efficient extraction of cytosolic as well as of membrane (phospho)proteins, we compared three common, in gel-free approaches frequently used extraction methods in a qualitative experiment. Here, in total 301 phosphorylation events could be identified, of which 262 could be localized to a specific Ser, Thr, or Tyr residue. This dataset represents the so far most comprehensive qualitative phosphoproteome dataset of *Synechocystis*. It encouraged us to investigate phosphoproteome dynamics in response to nitrogen starvation in a quantitative manner employing for the first time in a bacterial system the method of chemical dimethylation labeling. Extracts from *Synechocystis* cultures grown in the presence of nitrate or ammonia and cultures subjected to nitrogen starvation were dimethyl labeled. Thereby, we could identify altogether 2382 proteins and 183 phosphorylation events. Of those, the dynamics of 2111 proteins and 148 phosphorylation events from two independent experiments could be quantified. The P_II_ signaling protein, which served as a positive control to validate the experiments, showed the highest increase in phosphorylation level upon nitrogen starvation. Other proteins with increased phosphorylation levels comprised functions in photosynthesis and in carbon and nitrogen metabolism, suggesting an important role of protein S/T/Y phosphorylation in such a fundamental acclimation response.

## Material and methods

### Cell culture and harvest

Wild-type *Synechocystis* sp. PCC 6803 substrain Kazusa was grown photoautotrophically at a constant photon flux density of 40 μmol photons m^−2^ s^−1^ in 5 mM NaHCO_3_ and 20 mM HEPES supplemented BG11 medium (Rippka, 1988), with either 17.6 mM sodium nitrate (NaNO_3_) or 5 mM ammonium chloride (NH_4_Cl) as exclusive nitrogen source, at 26°C. A stationary culture of *Synechocystis* in BG11 nitrate medium served as a stock for the final inoculation of experimental 500 mL batch cultures in the qualitative experiments. For the quantitative experiment 1, pre-cultures were grown from the stock in fresh nitrate or ammonia medium to optical density (OD)_750_ = 0.6–0.8 and were directly used as inoculum for the experimental 500 mL batch cultures. In experiment 2, the pre-cultures were repeatedly cultivated to OD_750_ = 0.6–0.8 and after five cycles used as inoculum for the experimental 500 mL batch cultures. All experimental cultures were inoculated to an initial OD_750_ = 0.2 and grown to OD_750_ = 0.6 with ambient air bubbling and magnetic stirring. Cultures were harvested by addition of 100 g ice for rapid cooling and centrifugation at 7477 × g for 10 min. The supernatant was removed and cell pellets were resuspended in 50 mL ice cold nitrogen-free BG11 medium and centrifuged again. Cell pellets were quick-frozen in liquid nitrogen. For nitrogen starvation conditions, nitrate grown cells (at OD_750_ = 0.6) were shifted to nitrogen-free BG11 medium by centrifugation with 3500 × g for 8 min at room temperature (RT). The supernatant was decanted, the cell pellet was gently overlaid with nitrogen-free BG11 medium, swiveled and decanted again and finally, the cell pellet was resuspended in the same volume as before centrifugation. Cells were kept for 24 h under previous incubation conditions and harvested as described.

### Protein extraction, in-solution digestion, and peptide dimethylation labeling

Protein extraction method A: Filter Aided Sample Preparation was performed as described previously (Wisniewski et al., 2009) with the following exceptions: In brief, a frozen cell pellet from a nitrate grown culture was resuspended in 2 mL lysis buffer, containing 4% (w/v) sodium dodecyl sulfate (SDS) and 100 mM dithiothreitol (DTT) in 100 mM Tris/HCl, pH 7.5, and each 5 mM of the following phosphatase inhibitors: glycerol-2-phosphate; sodium fluoride (both Sigma-Aldrich) and sodium orthovanadate (Alfa Aesar). The sample was incubated for 10 min at 95°C in a water bath and subsequently sonified on ice for 30 s with a Branson Sonifier 250/Microtip 5 at output control 4 and 40% duty cycle for DNA comminution. The lysate was centrifuged at 13,000 × g for 5 min and the supernatant was transferred onto an Amicon Ultra-15 3 kDa Centrifugal Filter Unit. The lysate was mixed with 5 mL urea buffer (6 M urea/2 M thiourea in 100 mM Tris/HCl; pH 7.5) and centrifuged with 3345 × g for 10 min at RT. This step was repeated once for washing. Alkylation of reduced cysteine disulfide bonds was performed by washing the filter unit with 5 mL urea buffer containing 50 mM iodoacetamide (IAA) and centrifugation for 15 min as before, followed by incubation with 2 mL IAA-urea buffer for 20 min in the dark. The filter was washed for three times with each 2 mL urea buffer and 50 mM NH_4_HCO_3_ buffer, subsequently. For protein digestion, 50 μg trypsin (MS grade; Thermo Scientific) in 500 μL NH_4_HCO_3_ buffer, pH 8.0, were added onto the filter and incubated overnight (o.n.) at 37°C. Peptides were collected in a fresh tube by centrifugation as before for 10 min and additional with 500 μL NH_4_HCO_3_ buffer.

Protein extraction method B: SDS buffer protein extraction and acetone/methanol precipitation: Proteins from frozen cell pellets were extracted with 2 mL lysis buffer (see method A, without DTT), supplemented with 10 mM ethylenediaminetetraacetic acid (EDTA). Cell lysates were sonified on ice as described in method A and subsequently reduced with a final concentration of 10 mM DTT for 1 h at RT under agitation at 650 rpm on a shaker. Alkylation was performed with a final concentration of 5.5 mM IAA for 1 h at RT under agitation in the dark, followed by centrifugation at 13,000 × g for 5 min. Proteins in the supernatant were precipitated by mixing with eight volumes ice cold acetone and one volume ice cold methanol, followed by incubation at −20°C o.n. The precipitate was washed five times with 5 mL ice cold 80% (v/v) acetone in water and centrifugation at 1000 × g. The protein pellets were air dried and dissolved in urea buffer.

Protein extraction method C: Protein extraction with Y-PER (Yeast Protein Extraction Reagent; Thermo Scientific) and acetone/methanol precipitation: A frozen cell pellet was resuspended in 2 mL Y-PER, supplemented with 50 μg/mL lysozyme (from chicken egg, Sigma-Aldrich), c0mplete protease inhibitors (Roche) and phosphatase inhibitors, as described in method A. The suspension was incubated at 37°C for 20 min under agitation at 650 rpm. Cell lysates were sonified on ice and centrifuged as described before. Proteins in the supernatant were acetone/methanol precipitated, washed and dissolved in urea buffer as described in method B. Reduction and alkylation of cysteines was subsequently performed as described in method B.

Protein concentration of samples derived from methods B and C were measured by Bradford assay (Bio-Rad) and subsequently pre-digested with endoproteinase Lys-C (Waco) for 3 h and digested further o.n. with trypsin (MS grade; Thermo Scientific) at RT. The protease/protein ratio was for both enzymes 1:100 (w/w). The resulting peptide mixtures from methods A, B, and C were acidified to pH 2.5 with trifluoroacetic acid (TFA) and desalted by solid phase extraction on SepPak C18 columns (Waters) according to the manufacturer's instructions.

For the quantitative experiments, samples were extracted with method B, in-solution digested and on-column (SepPak C18) dimethylation labeled as described previously (Boersema et al., 2009). In brief, 5 mL of the respective labeling solutions with CH_2_O (Sigma-Aldrich) and NaBH_3_CN (Fluka) for light-, CD_2_O (Sigma-Aldrich) and NaBH_3_CN for medium-heavy and ^13^CD_2_O (Sigma-Aldrich) and NaBD_3_CN (Sigma-Aldrich) for heavy labeling were flushed with 15 min contact time through the column. Labeled peptides were washed with 5 mL HPLC Solvent A (0.5% acetic acid) on the column and eluted with HPLC Solvent B (80% acetonitrile in 0.5% acetic acid). For validation of labeling efficiency and correct mixing of the labeled peptides, two times 5 μg of each labeled sample (based on Bradford measurements) were used for separate measurements or mixed 1:1:1 and subjected after purification by C18 stage tips (Ishihama et al., 2006) to pilot LC-MS/MS measurements. Based on the obtained label ratios, correction factors were applied for correct mixing of samples. The labeling efficiencies were in all cases ≥94%.

### Sample preparation

Sample fractionation for the qualitative proteome experiments was performed on the peptide level by strong anion exchange chromatography (SAX). For quantitative proteome analysis, isoelectric focusing (OffGel fractionation strategy) was performed: For SAX, 100 μg peptides were loaded onto in-house packed SAX column (Empore™) and subsequently eluted stepwise in five pH fractions with 20 mM of acetic-, phosphoric- and boric acid at pH 3, 4, 5, 6 and 8, (pH adjusted with sodium hydroxide), respectively. Each fraction was subsequently purified by C18 stage tips. For OffGel fractionation, 100 μg of the labeled peptide mixture was separated on a linear 13 cm pH 3–10 IEF strip (GE Healthcare) and focused on a OffGel Fractionator 3100 (Aglient), according to the manufacturer's protocol, with 20 kVh at maximum 50 μA. Peptides focused in 12 fractions were separately purified by C18 stage tips.

Phosphopeptide enrichment for qualitative analyses was performed from the digested samples of protein extraction methods A, B and C by TiO_2_ chromatography (Soares et al., 2013), with the following exceptions: Obtained peptide solutions from solid phase extraction (pH 2.5) were directly incubated with each 5 mg TiO_2_ spheres (10 μm; MZ Analysetechnik), pre-incubated with 2,5-dihydrobenzoic acid (final concentration 30 mg/mL) for five consecutive rounds of 30 min. TiO_2_ spheres were washed and eluted as described previously and purified by C18 stage tips. The sample volume was reduced by vacuum centrifugation at RT and subjected to nano-LC-MS/MS analysis.

For the quantitative study, labeled phosphopeptides were enriched by TiO_2_ chromatography from each 15 mg peptide mixture. For TiO_2_ chromatography, a modified protocol was applied: peptides were eluted from SepPak columns with 80% acetonitrile in 6% TFA and enriched for eight consecutive rounds with each 5 mg TiO_2_ spheres (5 μm Sachtopore NP) for 10 min. TiO_2_ spheres were washed twice with each 1 mL 80% acetonitrile in 6% TFA for 1 min at 1000 rpm and loaded onto C8 (Empore™) stage tips. The spheres were washed once with 200 μL HPLC Solvent B and phosphopeptides were eluted with 30 μL 5% ammonium hydroxide solution in 60% acetonitrile, pH 11, for 30 min at 4°C into 30 μL 20% TFA in a fresh tube. Eluates were purified by C18 stage tips. The sample volume was reduced by vacuum centrifugation at RT and subjected to nano-LC-MS/MS analysis.

### Mass spectrometry

For nanoLC-MS/MS analyses of proteome and phosphoproteome samples, peptides were loaded onto an in-house packed 15 cm reverse-phase C18 (3 μm; Dr. Maisch) nanoHPLC column on an EasyLC nano-HPLC (Proxeon Biosystems). Separation was performed by 90 min (for OffGel-separated samples) or 130 min (for TiO_2_-enriched samples) segmented linear gradients with 5–90% HPLC solvent B. Eluted peptides were directly ionized and measured on a LTQ Orbitrap XL (qualitative study), or LTQ Orbitrap Elite mass spectrometer (quantitative study). Mass spectrometers were operated in the positive ion mode.

The LTQ Orbitrap XL had the following acquisition cycle: one initial full (MS) scan in the Orbitrap mass analyzer was acquired at resolution 60,000 and scan range of m/z 300–2000, followed by collision induced dissociation (CID) of the 5 (phosphoenrichment) or 15 (proteome) most intense multiply charged ions in the linear ion trap mass analyzer (LTQ). For phosphoproteome analysis, multi stage activation (MSA) in all MS/MS events with neutral losses of phosphoric acid on singly (−97.97 Th), doubly (−48.99 Th) or triply (−33.66 Th) charged precursor ions, was activated.

The LTQ Orbitrap Elite was conducting higher energy collision dissociation (HCD) of the 20 most intense multiply charged ions at the same scan range at resolution 120,000.

Dynamic exclusion of sequenced precursor ions for 90 s and the lock mass option (Olsen et al., 2005) for real time recalibration were enabled on both instruments.

The mass spectrometry proteomics data have been deposited to the ProteomeXchange Consortium (http://proteomecentral.proteomexchange.org) via the PRIDE partner repository (Vizcaíno et al., 2013) with the dataset identifier PXD001831.

### Data processing and validation

All raw MS spectra were processed with MaxQuant software suite (version 1.5.1.0) (Cox et al., 2009) and default settings. Identified peaks were searched against the target-decoy databases of *Synechocystis* sp. PCC 6803 from Cyanobase (http://genome.microbedb.jp/cyanobase) and Uniprot (http://www.uniprot.org), containing 3672 and 3507 protein sequences and 245 common contaminants, with the following database search criteria: Trypsin was defined as cleaving enzyme and up to two missed cleavages were allowed. Carbamido-methylation of cysteines was set as a fixed and methionine oxidation, protein N-termini acetylation and S/T/Y phosphorylation were set as variable modifications. Light-, medium-heavy- and heavy- dimethylation labeling on peptide N-termini and lysine residues was defined for samples from the quantitative experiments. The initial mass tolerance of precursor ions was limited to 6 ppm and 0.5 ppm for fragment ions. False discovery rates (FDRs) of peptides and proteins were set to 1%, respectively. Quantification of dimethylation labeled peptides required at least two ratio counts. Peptides were only allowed with a posterior error probability (PEP) <1% at the peptide- and <5% at the phosphopeptide level. Phosphopeptide MS/MS spectra were further manually filtered and validated with stringent acceptance criteria: Phosphorylation site localization with ≥ 75% localization probability was manually validated as well as comprehensive coverage of b- and y-ion series for CID and HCD spectra as well as a low noise to signal ratio (Supplementary MS Spectra). To exclude quantitation bias of phosphopeptide ratios due to fluctuations of corresponding proteins, phosphopeptide ratios were normalized by division with corresponding protein ratios. Significance analysis of regulated phosphorylation events was performed with Perseus software (version 1.5.0.31), normalized phosphopeptide ratios were log2 transformed and plotted against the respective log10 transformed phosphopeptide intensities. Significantly regulated phosphorylation events were identified by significance A analysis with a *p*-value of 0.05.

### Immunoblot analysis

Proteins from 2 mL cell culture aliquots were extracted in 50 mM Tris/HCl, 5 mM EDTA buffer, pH 7.4, with a RiboLyser, conducting five cycles with a speed of 6.5 m s^−1^ for 15 s at 4°C. Protein concentration was measured by Bradford assay and 50 μg or 15 μg total protein content was separated either by SDS- or by non-denaturing clear native polyacrylamide gel electrophoresis (PAGE), respectively. Pre-cast Tris-glycine 4–20% gradient SDS polyacrylamide gels (NuSep) or 4–16% gradient native polyacrylamide gels (Serva) were used according to the manufacturer's protocols. Protein transfer was performed on a semi-dry blotting system (peqlab) onto polyvinylidene fluoride (BioTrace™ PVDF, Pall Corporation) membranes. Membranes were blocked o.n. in TBS and 1% Tween20 at 4°C. Polyclonal primary antibodies against the P_II_ protein (serum, produced in rabbit) or the AtpB protein (Abcam 65378, produced in rabbit) were incubated for 2 h at RT in a 1:5000 dilution in TBS-T after washing of the membrane with TBS-T buffer. Membranes were washed again and peroxidase coupled secondary antibody (Sigma A6154) was added in a 1:2000 dilution for 1 h at RT. Proteins were visualized by Lumi-Light Plus detection reagent (Roche).

## Results

As an initial step to systematically approach the global phosphoproteome of *Synechocystis* PCC 6803, we first performed a conventional qualitative gel-free phosphoproteome analysis, based on TiO_2_ enrichment of phosphopeptides from a tryptic digest of the crude protein extract, followed by mass spectrometry (MS) analysis. In order to achieve the most comprehensive coverage of the phosphoproteome, we compared three common sample preparation workflows for MS identification of phosphorylation events as well as for total proteome analysis (see below). Based on the obtained results, we applied the most successful protein extraction strategy for a quantitative study to analyze phosphoproteome dynamics in response to nitrogen starvation.

### The qualitative (phospho)proteome of *synechocystis* under growth on nitrate

Cells were harvested from a nitrate-grown *Synechocystis* culture at mid exponential growth phase (OD_750_ = 0.6) and split into three equal parts that were subjected to the following workflows: (A) Filter Aided Sample Preparation (FASP), (B) protein extraction as in the FASP protocol in combination with acetone/methanol protein precipitation, and (C) extraction with Y-PER in combination with acetone/methanol protein precipitation (hereafter designated as methods A, B and C, respectively; for details see Materials and Methods). For total proteome analyses, one portion of the sample was fractionated into five fractions by strong anion exchange chromatography. The remaining sample was used for five consecutive rounds of phosphopeptide enrichment by TiO_2_ chromatography. All samples were analyzed by nanoLC-MS/MS on an LTQ Orbitrap XL mass spectrometer.

On the proteome level, 2055 proteins were identified in the combined analysis of all three tested sample preparation workflows, including proteins detected in the proteome measurements and (unmodified) proteins detected from the TiO_2_ chromatography. Method A recovered 1835 proteins, method B 2014 proteins and method C 1983 proteins in total (Supplementary Table 1). At the phosphoproteome level, 301 phosphorylation events were identified with high confidence after manual validation of phosphopeptide MS/MS spectra, of which 262 non-redundant phosphorylation events, corresponding to 242 non-redundant phosphopeptides and 188 phosphoproteins, could be located on a specific S/T/Y residue. Additionally, 39 non-redundant phosphorylation events could be located on specific peptides and are referred to as unlocalized. Method A, B or C recovered, respectively, 21, 235, or 67 non-redundant phosphorylation events in total (Supplementary Table 2; Supplementary Information 1).

We concluded that all three methods had a similar performance on the protein level; however, striking differences in the performance were observed on the phosphoproteome level, with method B obviously being the method of choice. These results clearly demonstrate the importance of a potent and suited protein extraction protocol for the analysis of the phosphoproteome in organisms with a high content of membranous compartments such as cyanobacteria. As evidenced here, the overall number of identified proteins did not automatically lead to high numbers of identified phosphorylation events. Since phosphorylation events have in many cases a low occupancy in prokaryotic organisms (Soares et al., 2013), the quantity of specific extracted (phospho)proteins may be crucial, however this issue only can be analyzed in a quantitative experiment.

### Quantification of the *synechocystis* (phospho)proteome

The qualitative phosphoproteome analysis of the nitrate-grown *Synechocystis* sp. PCC 6803 cells showed a high abundance of protein S/T/Y phosphorylation; therefore, we were interested in the dynamic response of the (phospho)proteome in response to nitrogen starvation. Since autotrophically growing cyanobacteria are not suitable for metabolic stable isotope labeling, we used a protocol in which samples are labeled after protein digestion, using stable isotope dimethylation of peptides on N-termini and lysine side chains (Boersema et al., 2009). The experimental setup allowed us to analyze and compare three different metabolic conditions: (1) cells exponentially growing on nitrate; (2) cells exponentially growing on ammonia; (3) cells from nitrogen-free medium (nitrogen starvation for 24 h after growth on nitrate). We utilized this approach to perform a global quantitative phosphoproteome study including two experiments with slightly different cultivation settings. In the first experiment, cells from a stationary culture were diluted into fresh medium, grown to an optical density of 0.6 and then used as an inoculum for the final experimental culture. In the second experiment, the cells were pre-adapted to their respective nitrogen source by five cultivation cycles, from where the final experimental culture was started. The exact cultivation conditions for both experiments are described in the Materials and Methods section. The comparison between nitrate and ammonium-grown cells was not further addressed in this study.

Proteins were extracted from cell pellets by “method B” as described in Materials and Methods, precipitated by acetone-methanol mixture and subjected to in-solution digestion by trypsin. Obtained peptide solutions were loaded onto C18 reverse phase columns and subsequently chemically labeled by dimethylation. Different isotopoloques of formaldehyde and cyanoborohydride were used: Samples from nitrate grown cultures were light labeled with CH_2_O and NaBH_3_CN, samples from ammonia grown cultures were medium-heavy labeled with CD_2_O and NaBH_3_CN and samples from nitrogen starved cells were heavy labeled with ^13^CD_2_O and NaBD_3_CN. Labeling efficiency was in all cases 94% or greater (Supplementary Information 2). Labeled samples from nitrate (NO^−^_3_; light), ammonium (NH^+^_4_; medium-heavy), and nitrogen starved (-N; heavy) cells were then mixed in a ratio of 1:1:1 based on the underlying protein amounts. Correct mixing of labeled peptides was validated by a dedicated MS measurement. The mixed sample was used for proteome and phosphoproteome analyses. For proteome analysis, isoelectric focusing (IEF) of the peptide mixtures was performed (OffGel strategy) and phosphopeptide enrichment was conducted by TiO_2_ chromatography in two technical replicates. All samples were analyzed by nanoLC-MS/MS on an LTQ Orbitrap Elite mass spectrometer. The experimental workflow is shown in Supplementary Information 3. To exclude quantification bias of phosphorylation events induced by possible alterations in the abundance of the corresponding proteins, measured phosphopeptide ratios were normalized by the respective unmodified protein ratios from the proteome measurements.

### Technical reproducibility of the experiments

To estimate the technical reproducibility of the phosphoproteome, we analyzed the correlation of phosphorylation event ratios, determined by phosphopeptide enrichments, between the two technical replicates of each experiment. To this end, we calculated Pearson correlation coefficients of 65 and 67 shared quantified phosphorylation events between technical replicates for experiments 1 and 2, respectively. The calculated Pearson correlation coefficients of unnormalized phosphorylation events were 0.905 for –N/NO^−^_3_ ratios and 0.937 for –N/NH^+^_4_ ratios for the first experiment and 0.948 for –N/NO^−^_3_ ratios and 0.913 for –N/NH^+^_4_ ratios for the second experiment, revealing a good technical reproducibility and high overlap of phosphorylation events. Correlation plots from technical replicates of phosphopeptide ratios are shown in the supplement (Supplementary Information 4). When comparing the correlation between experiments 1 and 2 in the final dataset, differences on the proteome level as well as on the phosphoproteome level could be observed, which might be accounted to the different pre-culture treatments. On the proteome level, the calculated Pearson correlation coefficients were 0.758 for –N/NO^−^_3_ protein ratios and 0.867 for –N/NH^+^_4_ protein ratios between experiments 1 and 2. On the phosphoproteome level, the Pearson correlation coefficient were 0.687 for 87 shared –N/NO^−^_3_ phosphorylation event ratios and 0.638 for 89 shared –N/NH^+^_4_ phosphorylation event ratios between experiments 1 and 2 (Supplementary Information 5).

### Protein phosphorylation dynamics in response to nitrogen starvation

The combined dataset including proteome and phosphoproteome measurements from all experiments led to the overall identification of 232,924 MS/MS spectra, covering 16,774 non-redundant peptides from 2382 proteins. The estimated false discovery rate (FDR) was 0.18% at the peptide level and 1.04% at the protein level. Of the identified 2382 proteins, 2111 proteins fulfilled the requirement of having at least two quantification events (ratio counts) in at least one of both experiments and were considered as quantified.

Wang and coworkers cataloged the so far identified *Synechocystis* sp. PCC 6803 proteome by combining previously published proteome studies. Thereby, they reported a total of 1738 experimentally identified proteins, which amounts to 47.3% of the possible 3672 protein coding genes listed on Cyanobase (Wang et al., 2009). By comparison, the present quantitative gel-free proteome analysis identified 64.9% of the theoretical *Synechocystis* proteome (2382 of 3672 proteins), with a quantification rate of 57.5% of the total proteome (2111 of 3672). These numbers are presumably close to the completeness of the expressed proteome under the actual experimental conditions, since of the previously identified 1738 proteins, 90.0% (1565) were also identified in the present study. Of the 1935 previously unidentified proteins, 44.3% (857) were identified and 32.0% (620) were even quantified in our combined dataset. In a global transcriptome study, analyzing ten different growth and stress conditions, Kopf and coworkers could recently identify a total of 3101 expressed genes in *Synechocystis*. Under nitrogen starvation and nitrate growth conditions, approximately 75% of these expressed genes were present (approx. 2300) (Kopf et al., 2014). These results match very closely to the number of identified proteins in our MS based analysis (2382).

On the phosphoproteome level, manual inspection of phosphopeptide MS/MS spectra identified in both experiments 183 S/T/Y phosphorylation events with high confidence. Of these, 148 phosphorylation events, corresponding to 86 phosphoproteins, fulfilled the following two criteria: quantification of the phosphorylation event with at least two ratio counts in at least one experiment and normalization by the corresponding protein ratio of the respective experiment. Of these 148 phosphorylation events, 105 were quantified in experiment 1 and 130 in experiment 2, with an overlap of 87 phosphorylation events between both experiments. All detected proteins and phosphopeptides (including unlocalized and localized phosphorylation events) from the quantitative study are shown in the supplement (Supplementary Tables 3, 4).

**Figure 2:**
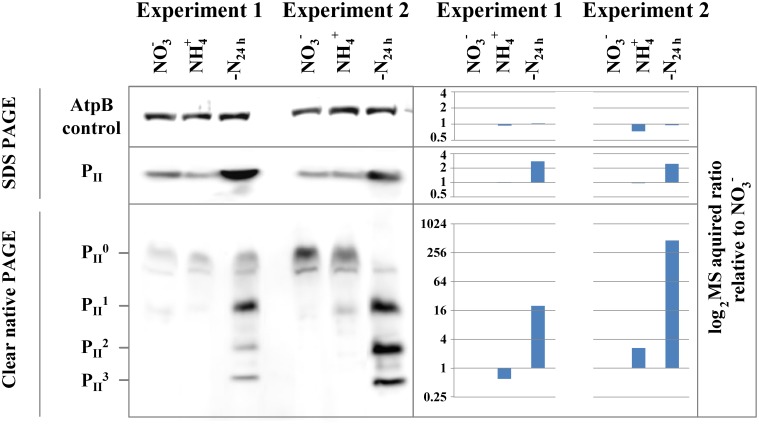
**Immunoblot validation of P_II_ protein and phosphorylation dynamics**. The change in protein abundance (SDS PAGE) and phosphorylation (Clear native PAGE) of the N-regulatory P_II_ protein between nitrate- (NO^−^_3_) or ammonia-grown (NH^+^_4_) and 24h h nitrogen starved cells (-N_24h_) from both experiments is shown on the left panel. P^0^_II_ represents the unphosphorylated trimeric complex and P^1–3^_II_ represent P _II_ trimers with one, two and three phosphorylated subunits. The AtpB protein serves as loading control for SDS and Clear native PAGE. Corresponding MS acquired protein and phosphorylation event ratios in log_2_ scale, relative to the NO^−^_3_ are shown on the right panel.

As a test case for our quantitative mass spectrometry acquired (phospho)proteome data, we analyzed the expression profile of the nitrogen regulatory signal protein P_II_ by immunoblot analysis on the protein level by SDS-polyacrylamide gel electrophoresis (PAGE) and on the phosphorylation level by non-denaturing PAGE (Forchhammer and Tandeau De Marsac, 1994). Under nitrogen-limiting conditions, the P_II_ signal protein accumulates, associated with enhanced phosphorylation of residue Ser49 at the apex of the surface exposed T-loop (Forchhammer, 2004). Under nitrogen-depleted conditions, all three subunits of the homotrimeric P_II_ protein are phosphorylated (Forchhammer and Tandeau De Marsac, 1994). In the present experiments, P_II_ protein levels were similar between nitrate or ammonium grown cells but increased under nitrogen starvation (Figure 2). This is in accordance with transcription analysis of the *glnB* gene (encoding the P_II_ protein), showing up-regulation under nitrogen starvation (Fadi Aldehni et al., 2003; Aguirre Von Wobeser et al., 2011). The result of the analysis of the P_II_ phosphorylation state by non-denaturing PAGE from both experiments is shown below in the figure. As expected, the Ser49 phosphorylation strongly increased under nitrogen starvation, with the phosphorylated P_II_ trimer isoforms becoming the most prominent bands. The unphosphorylated P_II_ protein is in contrast the most intense band in nitrate and ammonia grown conditions. Overall, MS based quantification of the P_II_ signaling protein with respect to protein abundance as well as on the phosphorylation level, as indicated in the figure, is in good agreement with the immunoblot results, implying that the quantitative MS analysis is valid.

**Figure 3:**
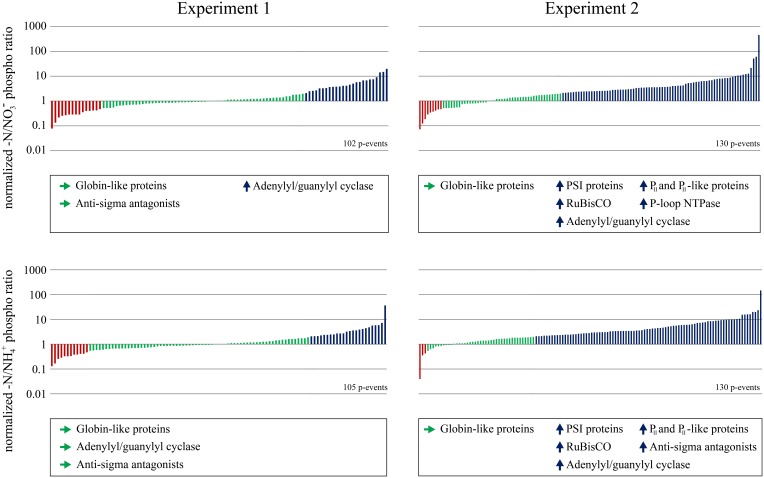
**Distribution of quantified phosphorylation events**. Ratio distributions of quantified phosphorylation events from nitrogen starved cells to nitrate grown cells (-N/NO^−^_3_) and nitrogen starved cells to ammonia grown cells (-N/NH^+^_4_) from both experiments are shown. Down-regulated phosphorylation events under nitrogen starvation (threshold 0.5) are colored red, static phosphorylation events are colored green and up-regulated phosphorylation events (threshold 2.0) are colored blue. Classified InterPro terms from phosphoproteins, identified at least twice for each experiment and ratio are listed below the corresponding experimental conditions -N/NO^−^_3_ or -N/NH^+^_4_. Classified proteins of each indicated InterPro term are listed in Supplementary information 6.

To analyze the response of the *Synechocystis* phosphoproteome to nitrogen starvation, we classified the quantified –N/NO^−^_3_ and –N/NH^+^_4_ phosphorylation event ratios into up- or down-regulated and static patterns, separately for both experiments (Figure 3). Functional assignment of phosphoproteins, comprising all proteins with one or more quantified phosphorylation events in our dataset (proteins with multiple phosphorylation events were only listed once), could be retrieved from InterPro database. InterPro protein terms were obtained for 81 of the 86 phosphoproteins. For classification of phosphorylation event ratios, we defined an arbitrary threshold of a twofold change; phosphorylation events with a ratio below 0.5 were classified as down-regulated; phosphorylation events with ratios above 2.0 were classified as up-regulated; phosphorylation events with a ratio between 0.5 and 2.0 were classified as static. Interestingly, a difference in the percentage of regulated or static phosphorylation events was observed between both experiments in response to nitrogen starvation (for –N/NO^−^_3_ and –N/NH^+^_4_ ratios). In experiment 1, characterized by a short pre-adaptation to the respective nitrogen sources, the majority of phosphorylation events revealed a static pattern, whereas, in experiment 2, characterized by a long pre-adaptation, the majority revealed up-regulation. In both experiments, down-regulated phosphorylation events revealed the lowest occurrence. The occurrence and frequency of InterPro terms was analyzed in the three classes (down-regulated, static or up-regulated) between –N/NO^−^_3_ and –N/NH^+^_4_ ratios for each experiment (see Figure 3), to reveal whether the phosphorylation pattern correlates with potential protein functions. Whereas, for down-regulated phosphoproteins this analysis did not reveal any results, globin-like proteins (e.g., phycobilisome) revealed in all cases static phosphorylation patterns. In addition, phosphorylation on anti-sigma antagonist proteins was in principal static in the first, but up-regulated in the second experiment. With the exception of the –N/NH^+^_4_ ratios in experiment 1, phosphorylation on adenylyl/guanylyl cyclase proteins was in principal up-regulated. In experiment 2, following proteins showed additionally up-regulated phosphorylation dynamics: proteins of photosystem I (PSI) and RuBisCO, as well as P-loop NTPase and N-regulatory P_II_ and P_II_-like proteins. However, in experiment 1, these phosphoprotein classes did not fulfill the requirement of having at least two up-regulated evidences. The respective classified proteins are indicated in Supplementary Information 6.

**Figure 4:**
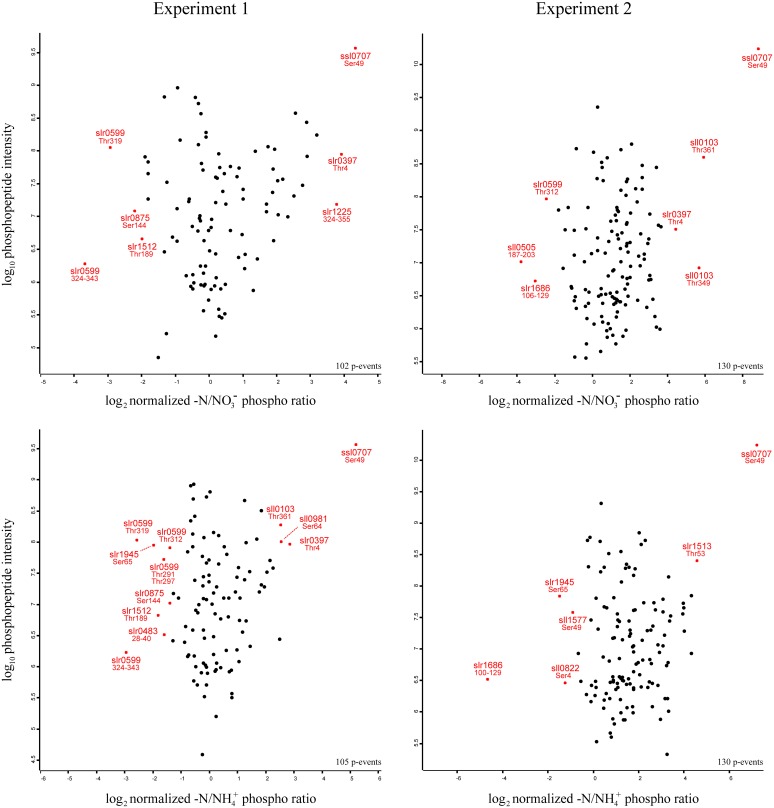
**Site specific distribution of significantly regulated phosphorylation events**. Log_2_ transformed ratio distributions of quantified phosphorylation events from nitrogen starved cells in comparison to nitrate grown cells (-N/NO^−^_3_) and nitrogen starved cells to ammonia grown cells (-N/NH^+^_4_), relative to the Log_10_ transformed phosphopeptide intensities are shown. Significantly regulated phosphorylation events from the respective experiments are displayed as stars (in red) and the protein ID and the localized phosphorylation site or phosphopeptide span is indicated.

This classification provides a comprehensive overview about general protein functions, which are frequently regulated under nitrogen starvation by S/T/Y phosphorylation in *Synechocystis*. In order to obtain a more detailed insight about specific phosphorylation events with strong dynamics, and their abundance under the studied conditions, we plotted the quantified phosphorylation event –N/NO^−^_3_ and –N/NH^+^_4_ ratios against the respective phosphopeptide intensities, separately for each experiment (Figure 4). Based on the overall distribution of the specific phosphorylation events, significantly regulated events (both, localized and unlocalized on the detected peptide) were identified by significance analysis, (*p*-value = 0.05; for details see Materials and Methods). Phosphorylation events revealing significant up- or down-regulation are depicted in the scatter plots as stars (in red), and the respective phosphoprotein and modification site (or phosphopeptide span in case of unlocalized phosphorylation events) is indicated. All significant phosphorylation events (between N/NO^−^_3_ and –N/NH^+^_4_ ratios) from both experiments are listed in Table 1.

**Table 1:** **List of significantly regulated phosphorylation events**.

**Phospho protein**	**Phosphopeptide sequence**	**Significantly regulated phosphorylation events**			
		**Experiment 1**		**Experiment 2**	
		**Down-regulated**	**Up-regulated**	**Down-regulated**	**Up-regulated**
Ssl0707	YRG**pS^49^**EYTVEFLQK	**-**	**–N/NO^−^_3_ and –N/NH^+^_4_**	**-**	**–N/NO^−^_3_ and –N/NH^+^_4_**
Slr1945	GQMGN**pS^65^**EVGHLNLGAGR	**–N/NH^+^_4_**	**-**	**–N/NH^+^_4_**	**-**
Slr1686	**^100^**LQSELSTLEQELAQQDSSPTGPGEEQKSSR**^129^**	**-**	**-**	**–N/NO^−^_3_ and –N/NH^+^_4_**	**-**
Slr1513	SSGQPN**pT^53^**SDIEANIK	**-**	**-**	**-**	**–N/NH^+^_4_**
Slr1512	QPVAAGDYGDQ**pT^189^**DYPR	**–N/NO^−^_3_ and –N/NH^+^_4_**	**-**	**-**	**-**
Slr1225	**^324^**APPGATVSTPQGTNTQIQPTPASSASPLTAPK**^355^**	**-**	**–N/NO^−^_3_**	**-**	**-**
Slr0875	LITTLENQQSS**pS^144^**Q	**–N/NO^−^_3_ and –N/NH^+^_4_**	**-**	**-**	**-**
Slr0599	GDRPGN**pT^291^**VANGK**pT^297^**K	**–N/NH^+^_4_**	**-**	**-**	**-**
Slr0599	SNHQPTAPTLVVG**pT^312^**PYNANDTQATK	**–N/NH^+^_4_**	**-**	**–N/NO^−^_3_**	**-**
Slr0599	SNHQPTAPTLVVGTPYNAND**pT^319^**QATK	**–N/NO^−^_3_ and –N/NH^+^_4_**	**-**	**-**	**-**
Slr0599	**^324^**VYTQEFTGYTETQEGSPLMK**^343^**	**–N/NO^−^_3_ and –N/NH^+^_4_**	**-**	**-**	**-**
Slr0483	**^28^**TDVGPITTPNPQK**^40^**	**–N/NH^+^_4_**	**-**	**-**	**-**
Slr0397	VL**pT^4^**QFASSPAPPQSFAVAPNR	**-**	**–N/NO^−^_3_ and –N/NH^+^_4_**	**-**	**–N/NO^−^_3_**
Sll1577	ITGNA**pS^49^**AIVSNAAR	**-**	**-**	**–N/NH^+^_4_**	**-**
Sll0981	LTL**pS^64^**AQGGNK	**-**	**–N/NH^+^_4_**	**-**	**-**
Sll0822	**pS^4^**NATKPLTGEALLEK	**-**	**-**	**–N/NH^+^_4_**	**-**
Sll0505	**^187^**LVAAGVILPLSERSASR**^203^**	**–N/NO^−^_3_**	**-**	**-**	**-**
Sll0103	YRQTQIAE**pT^349^**K	**-**	**-**	**-**	**–N/NO^−^_3_**
Sll0103	QGAA**pT^361^**MLQTAAK	**-**	**–N/NH^+^_4_**	**-**	**–N/NO^−^_3_**

As expected from previous studies, P_II_ (Ssl0707) Ser49 phosphorylation exhibited the highest level of increase in phosphorylation and intensity throughout all experiments in response to nitrogen starvation. Interestingly, two Ser/Thr protein kinases with contrary phosphorylation dynamics were identified: The Ser/Thr protein kinase C (Slr0599) revealed multiple significantly down-regulated phosphorylation events in experiment 1 and for one of them in experiment 2. Four out of five events could be localized on a specific Thr residues (on positions 291, 297, 312, and 319), and one event could be quantified on the phosphopeptide ^324^VYTQEFTGYTETQEGSPLMK^343^. In contrast, the putative Ser/Thr protein kinase F (Slr1225) revealed one significantly up-regulated phosphorylation event, which was quantified on the phosphopeptide ^324^APPGATVSTPQGTNTQIQPTPASSASPLTAPK^355^ for –N/NO^−^_3_ ratios in experiment 1.

Other significantly down-regulated phosphorylation events were detected in experiment 1 on Ser144 of the large-conductance mechanosensitive channel (Slr0875) and on the phosphopeptides ^28^TDVGPITTPNPQK^40^ and ^187^LVAAGVILPLSERSASR^203^ of the hypothetical proteins Slr0483 and Sll0505, respectively. The phosphopeptide ^100^LQSELSTLEQELAQQDSSPTGPGEEQKSSR^129^, Ser4 and Ser49 of the hypothetical proteins Slr1686 and Sll0822 and the phycocyanin beta subunit (Sll1577), respectively, revealed down-regulated phosphorylation events in experiment 2. Interestingly, down-regulated phosphorylation on Ser65 of the 2,3-bisphosphoglycerate-independent phosphoglycerate mutase (Slr1945) was detected in both experiments for only –N/NH^+^_4_ ratios. Among other proteins with significantly up-regulated phosphorylation events in our study, Ser64 phosphorylation of the unknown protein Sll0981 was detected in experiment 1, whereas, phosphorylations on Thr4 of the hypothetical protein Slr0397 and Thr349 and Thr361 of the hypothetical protein Sll0103 were detected in both experiments. Remarkably, the sodium-dependent bicarbonate transporter SbtA (Slr1512) revealed in our study down-regulation of Thr189 phosphorylation in experiment 1, whereas, the neighboring gene product, the P_II_-like membrane-associated protein SbtB (Slr1513), revealed significant up-regulation on Thr53 in experiment 2 in response to nitrogen starvation. For a complete overview of the detected phosphorylation events revealing significant regulation in response to nitrogen starvation, see Table 1.

## Discussion

### Sample preparation and dimethylation labeling for phosphoproteome quantification

Numerous previous studies revealed insights with regards to the *Synechocystis* proteome and shed first light into the phosphoproteome. However, most of these studies were exclusively focused on specific cellular compartments such as different membrane fractions or the cytoplasm or on specific proteins. Therefore, most previous studies mapped only “snapshots” of either delimited cellular compartments or only one particular metabolic condition of the proteome or the phosphoproteome, mainly performed in a qualitative manner, based on 2D gel strategies. The present study aimed to establish a method to analyze the global dynamic changes of the *Synechocystis* phosphoproteome in a site specific and quantitative manner between distinct metabolic conditions. In the present study, this was exemplified using nitrate and ammonia grown cells in comparison to 24 h nitrogen starved cells.

This study led to a comprehensive detection of the entire expressed proteome, including the identification of 857 and the quantification of 620 previously not identified proteins, and thus far is the largest reported dataset of phosphorylation events in *Synechocystis*. The efficient extraction of phosphoproteins contributed in combination with a gel-free MS strategy to a high percentage of detections in the qualitative phosphoproteome dataset. Although similar identification numbers were detected on the proteome level, when comparing method B with the detergent buffer based method C (Y-PER), significantly more localized phosphorylation events (235 vs. 67) could be obtained exclusively with method B. This may be due to the fact that the overall extraction efficiency of hydrophobic proteins was higher with method B compared the other methods. In bacteria, phosphorylation events are predominantly present in low occupancy compared to eukaryotes. Therefore, an overall small percentage of phosphoproteins are actually phosphorylated compared to the respective unmodified protein (Soares et al., 2013). When more copies of a certain phosphoprotein with low phosphorylation occupancy are present in the sample, the chances to detect the phosphorylation event or even to localize the modified S/T/Y site are much higher. This becomes especially relevant for quantitative studies, where the corresponding phosphorylation events must be detected in all samples and stages, even if their abundances are strongly fluctuating as in the case of regulated phosphorylation events.

Subsequent to an efficient protein extraction method from the cyanobacterial samples, the quantification technique is crucial. Although many different techniques for quantitative phosphoproteome studies have been reported, SILAC has been established as a precise and robust method firstly in eukaryotic and later in prokaryotic organisms (Ong et al., 2002; Soufi et al., 2010). Previously, we could detect the phosphoproteome dynamics of the model bacterium *E. coli* K-12 throughout the growth in minimal medium (Soares et al., 2013). However, in autotrophic cyanobacteria species, metabolic SILAC labeling would require severe disruptions in the nitrogen metabolic pathway by knocking out key enzymes of the lysine and/or arginine amino acid synthesis pathways. We decided to bypass this drawback by application of chemical stable isotope labeling based on dimethylation of peptides on a C18 column as described earlier. As mentioned, the methodology was applied before successfully in quantitative phosphoproteome studies, but limited to the eukaryotic domain as reviewed previously (Kovanich et al., 2012).

Here, we expanded this method to the quantitative phosphoproteome analysis in prokaryotic organisms. All validation steps of the labeling methodology confirmed a high quality of the experiments. The labeling efficiency was high (≥94%). The correlation between technical replicates of the phosphoproteome revealed high reproducibility and the overlap of quantified phosphorylation events between technical replicates (see Supplementary Information 4) and experiments (see Supplementary Information 5) was high. We conclude that the dimethylation labeling strategy combined with an efficient protein extraction method is highly suitable for systematically detecting phosphoproteome dynamics in (cyano)bacteria.

### Dynamics of protein S/T/Y phosphorylations involved in nitrogen and carbon metabolism and photosynthesis under nitrogen starvation

In bacteria, research on protein phosphorylation on Ser/Thr and Tyr residues in the past years led to accumulating evidences revealing this modification as one of the most prominent mechanisms in signal transduction for fast cellular responses to environmental changes (Macek and Mijakovic, 2011; Soufi et al., 2012). Moreover, previous phosphoproteomic studies have revealed that a large portion of these phosphorylation events are associated with key metabolic processes in a cell, however, detailed information of the phosphorylation functions are rare. For cyanobacteria, having an autotrophic lifestyle, fast response to changing environmental conditions that affect the energy-, nitrogen-, and carbon metabolism appears to be essential. This statement agrees with the high numbers of identified and quantified phosphorylation events in our dataset that can be assigned to photosynthesis, carbon and nitrogen uptake and subsequent metabolic pathways.

Of the significantly regulated phosphoproteins, the sensory nitrogen signaling P_II_ protein showed the strongest dynamics in response to nitrogen starvation with Ser49 phosphorylation being strongly up-regulated. This is in agreement with previous studies and emphasizes its role as a marker for nitrogen-limited conditions (Forchhammer and Tandeau De Marsac, 1994, 1995; Mikkat et al., 2014). In *E. coli*, it was reported, that the P_II_ homolog protein GlnK regulates the influx of ammonia from the surrounding medium through the homotrimeric ammonium/methylammonium permease AmtB, and GlnK-AmtB interaction blocks the uptake (Van Heeswijk et al., 1996). A similar mechanism, although not yet demonstrated, could be assumed in *Synechocystis* between P_II_ and the AmtB homologue Amt1 (Sll0108), with the P_II_ Ser49 phosphorylation preventing this interaction to ensure efficient ammonia uptake under N-limited conditions. Interestingly, we identified Amt1 as a phosphoprotein, which is increasingly phosphorylated under N-limitation. This mechanism could reveal an additional and more sensitive regulative control mechanism to ensure maximum ammonia uptake. Remarkably, the P_II_-like membrane-associated protein SbtB (Slr1513) revealed significant increasing Thr53 phosphorylation under N-limitation, like the GlnB P_II_ protein. Interestingly, the *sbtB* gene is neighboring the sodium-dependent bicarbonate transporter Slr1512 (*sbtA*) gene on the chromosome, which revealed in our analysis significant decreasing Thr189 phosphorylation under the same conditions. It is thus tempting to speculate that SbtB may be involved in regulation of SbtA activity, in agreement with a recent analysis of the sbtAB operon (Du et al., 2014).

Phosphorylation of proteins involved in the inorganic carbon uptake mechanism and the Calvin cycle was also observed: On the carbon dioxide concentrating mechanism (Ccm) proteins M and N (Sll1031 and Sll1032), multiple phosphorylation events were detected, of which three could be quantified and localized on Sll1031 (pSer352, pThr358, and pThr609) and one on Sll1032 (pThr121). These phosphorylation events generally showed either static or slightly increasing phosphorylation under N-depletion, with the most prominent regulation on pThr609, showing up to threefold increase for –N/NH^+^_4_ ratios. More pronounced, phosphorylation of the phosphoribulokinase (Sll1525) on Ser17 increased strongly upon N-starvation. In addition, two phosphorylation events were quantified on the small and large subunits of the ribulose bisphosphate carboxylase/oxygenase RuBisCO (Slr0012 and Slr0009), both showing a three- to fourfold increase in response to nitrogen starvation. Both phosphorylation events are localized on seryl-residues, Ser78 on the small and supposedly Ser323 on the large subunit. Interestingly, similar phosphorylation sites were identified in a phosphoproteome study of *Arabidopsis thaliana* on the respective subunits. Here, Ser71 and Ser321 were phosphorylated on the small and large subunits, respectively, besides several other phosphorylation events (Aryal et al., 2011).

Several enzymes involved in the pentose phosphate pathway and in glycolysis were detected as phosphoproteins, generally with static or increasing phosphorylation dynamics toward N-starvation, such as the fructose-bisphosphate aldolase (Sll0018) and the 6-phosphogluconate dehydrogenase (Sll0329). Interestingly, the 2,3-bisphosphoglycerate-independent phosphoglycerate mutase (Slr1945) elicited an opposite response, with the level of Ser65 phosphorylation significantly decreasing under N-depletion.

Cyanobacteria are considered as progenitors of chloroplasts, which explains why the photosynthetic apparatus is highly similar to that of green plants. However, some features are unique to cyanobacteria, such as the phycobilisome light harvesting antenna, transferring light energy to the PSII. Previously, linker proteins from the phycobilisome antenna and ferredoxin-NADPH reductase were identified as phosphoproteins in *Synechocystis*, and dephosphorylation was driven upon long-term exposure to high light intensities and under nitrogen limitation, initiating proteolytic cleavage and degradation (Piven et al., 2005). In our study, we detected a high abundance of phosphorylation on proteins from the photosynthesis apparatus; we detected in the qualitative dataset 60 phosphorylation events on specific S/T/Y residues from 24 proteins associated with photosynthesis (see Supplementary Table 2). During quantitative analysis, only nine phosphoproteins associated with photosynthesis could be detected. This is in agreement with the general tendency of less identified phosphorylation events in the quantitative dataset.

Among the detected proteins, the most prominent dynamics were observed for PSI, phycocyanin and allophycocyanin proteins. Surprisingly, phosphorylation events on these proteins revealed increasing levels under nitrogen starvation, in contrast to the phycobilisome antenna linker proteins, which were described to undergo dephosphorylation under nitrogen starvation. We were not able to quantify the phosphorylation events on linker proteins. On the proteome level, we detected strong decreasing levels of linker proteins Sll1579 and Sll1471, whereas, all other linker proteins (Slr1459, Slr0335, Sll1580, Slr2051, Ssr3383, and Ssl3093) revealed only slight decreasing or static levels under nitrogen starvation. This result could indicate that either the process of chlorosis has not occurred to a level that is required in order to significantly reduce the level of phycobiliproteins, or phycobiliproteins were only partially degraded and peptide fragments were still present in the cells, which were then detected by MS analysis. To solve this issue, further experiments containing earlier and later time points are required.

### Dynamics of serine/threonine kinases

In our quantitative phosphoproteome dataset, we observed an overall trend of increased phosphorylation levels during nitrogen limitation. These dynamics are visualized in Figures 3, 4. Overall, there was a higher incidence of increasing than of decreasing phosphorylation in response to nitrogen limitation. This observation is more pronounced in experiment 2, characterized by longer pre-adaption in the respective nitrogen source. Interestingly, two Ser/Thr protein kinases were found among the phosphoproteins with significantly fluctuating phosphorylation dynamics. Ser/Thr protein kinase F (Slr1225) displayed in response to nitrogen starvation increasing phosphorylation of two phosphothreonines on positions 24 and 27 (2–3 fold increased), and on an unlocalized phosphorylation event on the phosphopeptide ^324^APPGATVSTPQGTNTQIQPTPASSASPLTAPK^355^. Contrary, we identified four phosphothreonines to be significantly decreasing under the same conditions on the Ser/Thr protein kinase C (Slr0599) and one unlocalized phosphorylation event (see Table 1). These two Ser/Thr kinases were shown previously *in vitro* by radiolabeling experiments to autophosphorylate (Kamei et al., 2002). Furthermore, both kinases were suggested in combination with Ser/Thr protein kinase K to be required for a phosphorylation cascade resulting in GroES phosphorylation (Zorina et al., 2011). It is conceivable that the phosphorylation dynamics of both kinases are interconnected and could involve a protein phosphatase.

In conclusion, this study reports the first global phosphoproteome analysis of *Synechocystis*, with the highest discovery rate of proteins (2382) and phosphorylation events (301 under nitrogen growth conditions) described thus far. We showed quantitative dynamics of the total proteome in response to nitrogen starvation. This is not only of broad relevance for *Synechocystis* molecular biology, but sets the ground for a comprehensive analysis of the phosphoproteome, with quantified dynamics of 148 phosphorylation events. Using nitrogen starvation acclimation as a test case, this study reveals that protein S/T/Y phosphorylation may play an outstanding regulatory role for metabolic adaptation in the model cyanobacterium *Synechocystis*. This analysis paves the way for an in depth analysis of the role of protein phosphorylation in cyanobacteria, to unravel its contribution to homeostatic control.

### Conflict of interest statement

The Guest Associate Editor Ivan Mijakovic declares that, despite having coauthored an article with the author Boris Macek in 2014, the review process was handled objectively. The authors declare that the research was conducted in the absence of any commercial or financial relationships that could be construed as a potential conflict of interest.

## References

[B1] Aguirre Von Wobeser E. Ibelings B. W. Bok J. Krasikov V. Huisman J. Matthijs H. C. P. (2011). Concerted changes in gene expression and cell physiology of the cyanobacterium Synechocystis sp. Strain PCC 6803 during transitions between nitrogen and light-limited growth. Plant Physiol. 155, 1445–1457. 10.1104/pp.110.165837 21205618 PMC3046598

[B2] Allen M. Smith A. (1969). Nitrogen chlorosis in blue-green algae. Arch. Mikrobiol. 69, 114–120. 10.1007/BF00409755 4986616

[B3] Aryal U. K. Krochko J. E. Ross A. R. S. (2011). Identification of phosphoproteins in *Arabidopsis thaliana* leaves using polyethylene glycol fractionation, immobilized metal-ion affinity chromatography, two-dimensional gel electrophoresis and mass spectrometry. J. Proteome Res. 11, 425–437. 10.1021/pr200917t 22092075

[B4] Ashby M. K. Houmard J. (2006). Cyanobacterial two-component proteins: structure, diversity, distribution, and evolution. Microbiol. Mol. Biol. Rev. 70, 472–509. 10.1128/MMBR.00046-05 16760311 PMC1489541

[B5] Biggins J. Bruce D. (1987). The relationships between protein kinase activity and chlorophyll a fluorescence changes in thylakoids from the cyanobacterium Synechococcus 6301, in Progress in Photosynthesis Research, ed Biggins J. (Dordrecht: Springer), 773–776.

[B6] Boersema P. J. Raijmakers R. Lemeer S. Mohammed S. Heck A. J. R. (2009). Multiplex peptide stable isotope dimethyl labeling for quantitative proteomics. Nat. Protoc. 4, 484–494. 10.1038/nprot.2009.21 19300442

[B7] Collier J. L. Grossman A. R. (1992). Chlorosis induced by nutrient deprivation in Synechococcus sp. strain PCC 7942: not all bleaching is the same. J. Bacteriol. 174, 4718–4726. 1624459 10.1128/jb.174.14.4718-4726.1992 PMC206268

[B8] Cox J. Matic I. Hilger M. Nagaraj N. Selbach M. Olsen J. V.. (2009). A practical guide to the MaxQuant computational platform for SILAC-based quantitative proteomics. Nat. Protoc. 4, 698–705. 10.1038/nprot.2009.36 19373234

[B9] Du J. Förster B. Rourke L. Howitt S. M. Price G. D. (2014). Characterisation of cyanobacterial bicarbonate transporters in *E*. coli shows that SbtA homologs are functional in this heterologous expression system. PLoS ONE 9:e115905. 10.1371/journal.pone.0115905 25536191 PMC4275256

[B10] Fa di Aldehni M. Sauer J. Spielhaupter C. Schmid R. Forchhammer K. (2003). Signal transduction protein P(II) Is required for NtcA-regulated gene expression during nitrogen deprivation in the cyanobacterium Synechococcus elongatus strain PCC 7942. J. Bacteriol. 185, 2582–2591. 10.1128/JB.185.8.2582-2591.2003 12670983 PMC152603

[B11] Forchhammer K. (2004). Global carbon/nitrogen control by PII signal transduction in cyanobacteria: from signals to targets. FEMS Microbiol. Rev. 28, 319–333. 10.1016/j.femsre.2003.11.001 15449606

[B12] Forchhammer K. (2008). PII signal transducers: novel functional and structural insights. Trends Microbiol. 16, 65–72. 10.1016/j.tim.2007.11.004 18182294

[B13] Forchhammer K. Tandeau De Marsac N. (1994). The PII protein in the cyanobacterium Synechococcus sp. strain PCC 7942 is modified by serine phosphorylation and signals the cellular N-status. J. Bacteriol. 176, 84–91. 8282715 10.1128/jb.176.1.84-91.1994 PMC205017

[B14] Forchhammer K. Tandeau De Marsac N. (1995). Phosphorylation of the PII protein (glnB gene product) in the cyanobacterium Synechococcus sp. strain PCC 7942: analysis of *in vitro* kinase activity. J. Bacteriol. 177, 5812–5817. 7592328 10.1128/jb.177.20.5812-5817.1995 PMC177403

[B15] Görl M. Sauer J. Baier T. Forchhammer K. (1998). Nitrogen-starvation-induced chlorosis in Synechococcus PCC 7942: adaptation to long-term survival. Microbiology 144, 2449–2458. 10.1099/00221287-144-9-2449 9782492

[B16] Hagemann M. Golldack D. Biggins J. Erdmann N. (1993). Salt-dependent protein phosphorylation in the cyanobacterium Synechocystis PCC 6803. FEMS Microbiol. Lett. 113, 205–209 10.1111/j.1574-6968.1993.tb06515.x

[B17] Harrison M. A. Tsinoremas N. F. Allen J. F. (1991). Cyanobacterial thylakoid membrane proteins are reversibly phosphorylated under plastoquinone-reducing conditions *in vitro*. FEBS Lett. 282, 295–299. 10.1016/0014-5793(91)80499-S 1903715

[B18] Heinrich A. Maheswaran M. Ruppert U. Forchhammer K. (2004). The *Synechococcus elongatus* PII signal transduction protein controls arginine synthesis by complex formation with N-acetyl-l-glutamate kinase. Mol. Microbiol. 52, 1303–1314. 10.1111/j.1365-2958.2004.04058.x 15165234

[B19] Irmler A. Forchhammer K. (2001). A PP2C-type phosphatase dephosphorylates the PII signaling protein in the cyanobacterium Synechocystis PCC 6803. Proc. Natl. Acad. Sci. U.S.A. 98, 12978–12983. 10.1073/pnas.231254998 11687619 PMC60810

[B20] Ishihama Y. Rappsilber J. Mann M. (2006). Modular stop and go extraction tips with stacked disks for parallel and multidimensional peptide fractionation in proteomics. J. Proteome Res. 5, 988–994. 10.1021/pr050385q 16602707

[B21] Jers C. Soufi B. Grangeasse C. Deutscher J. Mijakovic I. (2008). Phosphoproteomics in bacteria: towards a systemic understanding of bacterial phosphorylation networks. Expert Rev. Proteomics 5, 619–627. 10.1586/14789450.5.4.619 18761471

[B22] Joseph A. Aikawa S. Sasaki K. Teramura H. Hasunuma T. Matsuda F.. (2014). Rre37 stimulates accumulation of 2-oxoglutarate and glycogen under nitrogen starvation in Synechocystis sp. PCC 6803. FEBS Lett. 588, 466–471. 10.1016/j.febslet.2013.12.008 24374346

[B23] Kamei A. Yuasa T. Geng X. Ikeuchi M. (2002). Biochemical examination of the potential eukaryotic-type protein kinase genes in the complete genome of the unicellular cyanobacterium Synechocystis sp. PCC 6803. DNA Res. 9, 71–78. 10.1093/dnares/9.3.71 12168951

[B24] Kopf M. Klähn S. Scholz I. Matthiessen J. K. F. Hess W. R. Voß B. (2014). Comparative analysis of the primary transcriptome of *Synechocystis* sp. PCC 6803. DNA Res. 21, 527–539. 10.1093/dnares/dsu018 24935866 PMC4195498

[B25] Kovanich D. Cappadona S. Raijmakers R. Mohammed S. Scholten A. Heck A. R. (2012). Applications of stable isotope dimethyl labeling in quantitative proteomics. Anal. Bioanal. Chem. 404, 991–1009. 10.1007/s00216-012-6070-z 22644145

[B26] Macek B. Mijakovic I. (2011). Site-specific analysis of bacterial phosphoproteomes. Proteomics 11, 3002–3011. 10.1002/pmic.201100012 21726046

[B27] Maheswaran M. Ziegler K. Lockau W. Hagemann M. Forchhammer K. (2006). P(II)-Regulated arginine synthesis controls accumulation of cyanophycin in Synechocystis sp. Strain PCC 6803. J. Bacteriol. 188, 2730–2734. 10.1128/JB.188.7.2730-2734.2006 16547064 PMC1428389

[B28] Mann N. H. (1994). Protein phosphorylation in cyanobacteria. Microbiology 140, 3207–3215. 10.1099/13500872-140-12-3207 7881542

[B29] Mijakovic I. Macek B. (2012). Impact of phosphoproteomics on studies of bacterial physiology. FEMS Microbiol. Rev. 36, 877–892. 10.1111/j.1574-6976.2011.00314.x 22091997

[B30] Mikkat S. Fulda S. Hagemann M. (2014). A 2D gel electrophoresis-based snapshot of the phosphoproteome in the cyanobacterium Synechocystis sp. strain PCC 6803. Microbiology 160, 296–306. 10.1099/mic.0.074443-0 24275102

[B31] Murata N. Suzuki I. (2006). Exploitation of genomic sequences in a systematic analysis to access how cyanobacteria sense environmental stress. J. Exp. Bot. 57, 235–247. 10.1093/jxb/erj005 16317040

[B32] Muro-Pastor M. I. Reyes J. C. Florencio F. J. (2001). Cyanobacteria perceive nitrogen status by sensing intracellular 2-oxoglutarate levels. J. Biol. Chem. 276, 38320–38328. 10.1074/jbc.M105297200 11479309

[B33] Nishiwaki T. Satomi Y. Nakajima M. Lee C. Kiyohara R. Kageyama H.. (2004). Role of KaiC phosphorylation in the circadian clock system of Synechococcus elongatus PCC 7942. Proc. Natl. Acad. Sci. U.S.A. 101, 13927–13932. 10.1073/pnas.0403906101 15347812 PMC518855

[B34] Olsen J. V. De Godoy L. M. F. Li G. Macek B. Mortensen P. Pesch R.. (2005). Parts per Million Mass Accuracy on an Orbitrap Mass Spectrometer via Lock Mass Injection into a C-trap. Mol. Cell. Proteomics 4, 2010–2021. 10.1074/mcp.T500030-MCP200 16249172

[B35] Ong S.-E. Blagoev B. Kratchmarova I. Kristensen D. B. Steen H. Pandey A.. (2002). Stable isotope labeling by amino acids in cell culture, SILAC, as a simple and accurate approach to expression proteomics. Mol. Cell. Proteomics 1, 376–386. 10.1074/mcp.M200025-MCP200 12118079

[B36] Pereira S. F. F. Goss L. Dworkin J. (2011). Eukaryote-like serine/threonine kinases and phosphatases in bacteria. Microbiol. Mol. Biol. Rev. 75, 192–212. 10.1128/MMBR.00042-10 21372323 PMC3063355

[B37] Petranovic D. Michelsen O. Zahradka K. Silva C. Petranovic M. Jensen P. R.. (2007). *Bacillus subtilis* strain deficient for the protein-tyrosine kinase PtkA exhibits impaired DNA replication. Mol. Microbiol. 63, 1797–1805. 10.1111/j.1365-2958.2007.05625.x 17367396

[B38] Piven I. Ajlani G. Sokolenko A. (2005). Phycobilisome linker proteins are phosphorylated in Synechocystis sp. PCC 6803. J. Biol. Chem. 280, 21667–21672. 10.1074/jbc.M412967200 15805115

[B39] Rippka R. (1988). [1] Isolation and purification of cyanobacteria, in Methods in Enzymology, ed Lester Packer A. N. G. (Academic Press), 3–27. 10.1016/0076-6879(88)67004-2 3148836

[B40] Sanders C. E. Melis A. Allen J. F. (1989). *In vivo* phosphorylation of proteins in the cyanobacterium Synechococcus 6301 after chromatic acclimation to Photosystem I or Photosystem II light. Biochim. Biophys. Acta Bioenerg. 976, 168–172 10.1016/S0005-2728(89)80226-9

[B41] Schwarz R. Forchhammer K. (2005). Acclimation of unicellular cyanobacteria to macronutrient deficiency: emergence of a complex network of cellular responses. Microbiology 151, 2503–2514. 10.1099/mic.0.27883-0 16079330

[B42] Soares N. C. Spät P. Krug K. Macek B. (2013). Global dynamics of the *Escherichia coli* proteome and phosphoproteome during growth in minimal medium. J. Proteome Res. 12, 2611–2621. 10.1021/pr3011843 23590516

[B43] Soufi B. Kumar C. Gnad F. Mann M. Mijakovic I. Macek B. (2010). Stable isotope labeling by amino acids in cell culture (SILAC) applied to quantitative proteomics of *Bacillus subtilis*. J. Proteome Res. 9, 3638–3646. 10.1021/pr100150w 20509597

[B44] Soufi B. Soares N. C. Ravikumar V. Macek B. (2012). Proteomics reveals evidence of cross-talk between protein modifications in bacteria: focus on acetylation and phosphorylation. Curr. Opin. Microbiol. 15, 357–363. 10.1016/j.mib.2012.05.003 22633124

[B45] Stock J. B. Ninfa A. J. Stock A. M. (1989). Protein phosphorylation and regulation of adaptive responses in bacteria. Microbiol. Rev. 53, 450–490. 2556636 10.1128/mr.53.4.450-490.1989 PMC372749

[B46] Van Heeswijk W. C. Hoving S. Molenaar D. Stegeman B. Kahn D. Westerhoff H. V. (1996). An alternative PII protein in the regulation of glutamine synthetase in *Escherichia coli*. Mol. Microbiol. 21, 133–146. 10.1046/j.1365-2958.1996.6281349.x 8843440

[B47] Vizcaíno J. A. Côté R. G. Csordas A. Dianes J. A. Fabregat A. Foster J. M.. (2013). The proteomics identifications (PRIDE) database and associated tools: status in 2013. Nucleic Acids Res. 41, D1063–D1069. 10.1093/nar/gks1262 23203882 PMC3531176

[B48] Wang Y. Xu W. Chitnis P. (2009). Identification and bioinformatic analysis of the membrane proteins of synechocystis sp. PCC 6803. Proteome Sci. 7, 11. 10.1186/1477-5956-7-11 19320970 PMC2666656

[B49] Wisniewski J. R. Zougman A. Nagaraj N. Mann M. (2009). Universal sample preparation method for proteome analysis. Nat. Methods 6, 359–362. 10.1038/nmeth.1322 19377485

[B50] Yang M.-K. Qiao Z.-X. Zhang W.-Y. Xiong Q. Zhang J. Li T.. (2013). Global phosphoproteomic analysis reveals diverse functions of serine/threonine/tyrosine phosphorylation in the model cyanobacterium Synechococcus sp. Strain PCC 7002. J. Proteome Res. 12, 1909–1923. 10.1021/pr4000043 23461524

[B51] Zhang C. C. Jang J. Sakr S. Wang L. (2005). Protein phosphorylation on ser, thr and tyr residues in cyanobacteria. J. Mol. Microbiol. Biotechnol. 9, 154–166 10.1159/000089644 16415589

[B52] Zorina A. Stepanchenko N. Novikova G. V. Sinetova M. Panichkin V. B. Moshkov I. E.. (2011). Eukaryotic-like Ser/Thr protein kinases SpkC/F/K are involved in phosphorylation of GroES in the cyanobacterium Synechocystis. DNA Res. 18, 137–151. 10.1093/dnares/dsr006 21551175 PMC3111230

