# Supplementary material for: Phosphoproteome of the cyanobacterium Synechocystis sp. PCC 6803 and its dynamics during nitrogen starvation
Source: Front Microbiol. 2015 Mar 31;6:248. doi: 10.3389/fmicb.2015.00248 (PMC4379935; doi:10.3389/fmicb.2015.00248)
Supplement: Supplementary file 1 [file DataSheet1.ZIP › data sheet 1 (1)/Supplementary Information 1.PDF]

Supplementary information related to the identified phosphorylation events from the qualitative dataset

Of the 21, 235 or 67 non-redundant phosphorylation events, recovered with method A, B or C, respectively, three out of 21 phosphorylation events were exclusively identified in method A, 191 out of 235 exclusively in method B and 24 out of 67 exclusively in method C (**Supplementary table 2**). To determine the reliability of the uniquely identified phosphorylation events per method, median posterior error probability (PEP) of the modified peptides were calculated and showed low error probability for all exclusively identified phosphopeptides with localized modified residues from the three methods A, B and C of 2.34E-3, 9.20E-4 and 2.96E-3, compared to the total average PEP of all 262 localized phosphopeptides of 4.14E-4.

Supplementary information 2

Labeling efficiency of samples from the quantitative dataset

Experiment 1 | Experiment 2

Light dimethylation labeling

Labeling efficiency 94.2%

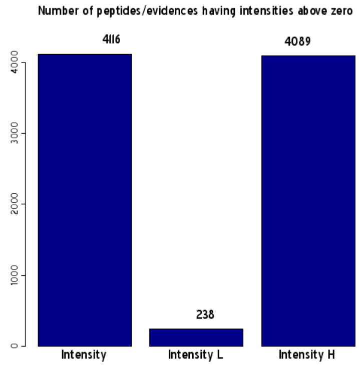

Labeling efficiency 95.5%

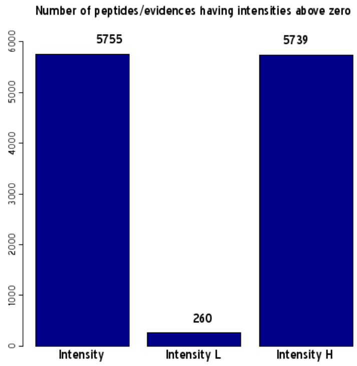

Medium-heavy dimethylation labeling

Labeling efficiency 96.2%

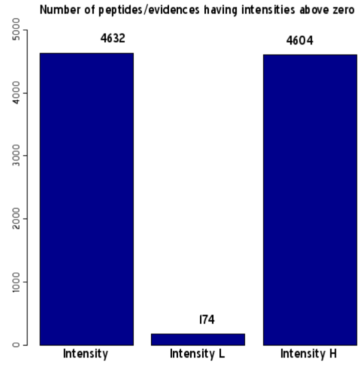

Labeling efficiency 97.5%

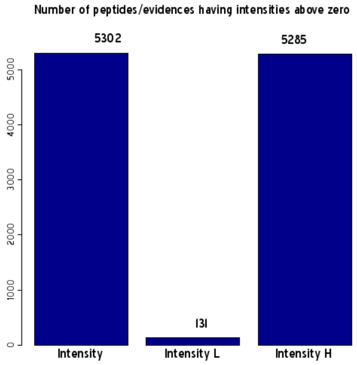

Heavy dimethylation labeling

Labeling efficiency 97.3%

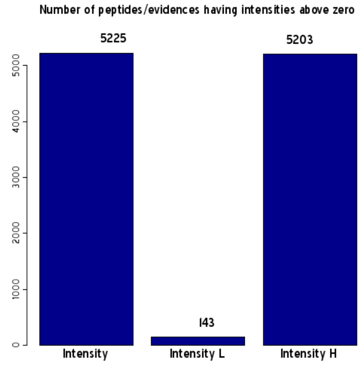

Labeling efficiency 98.5%

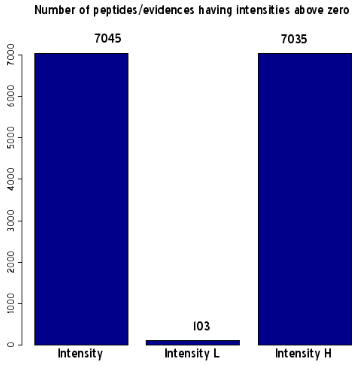

Supplementary information 3: Experimental workflow for quantitative experiments.

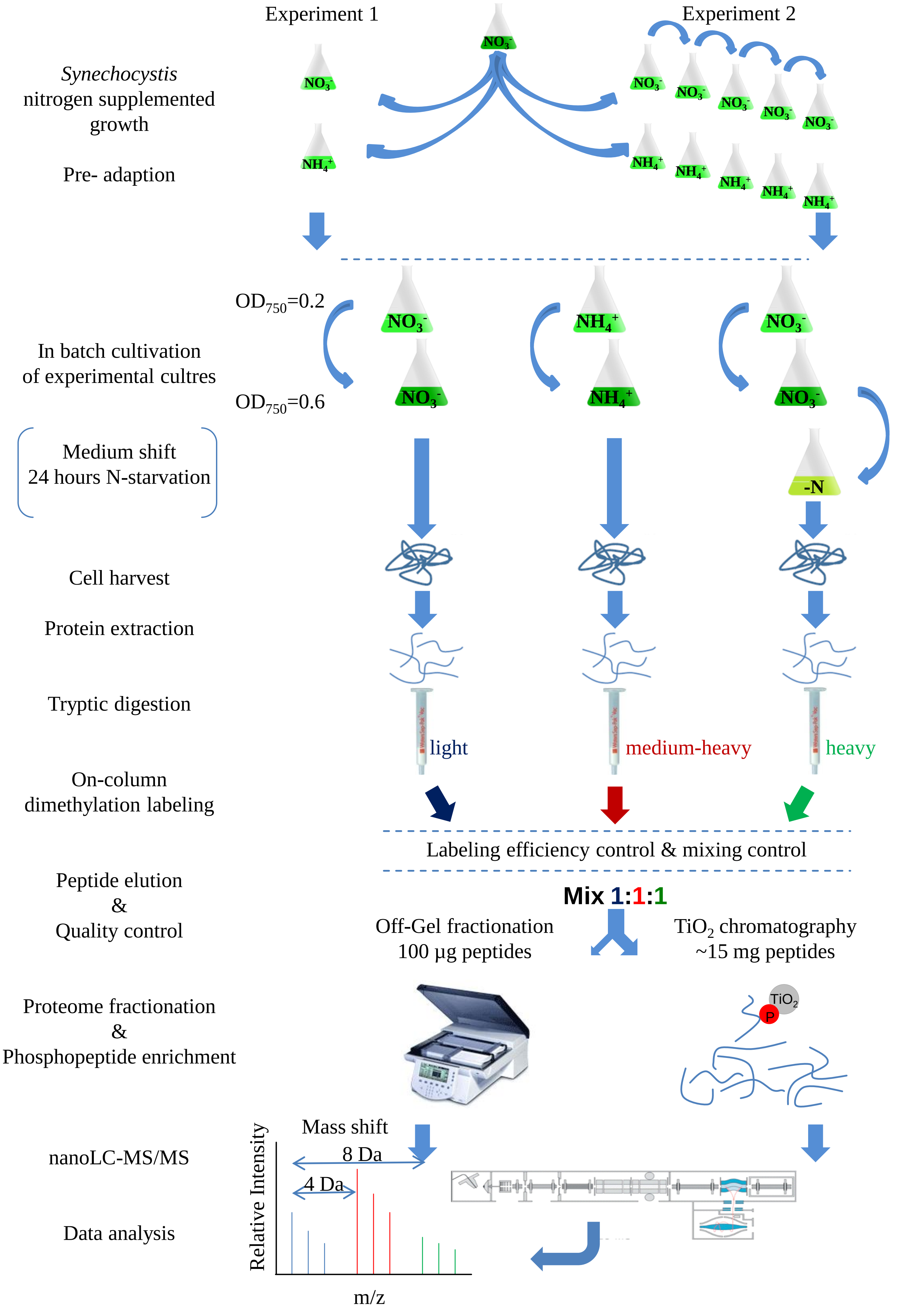

Supplementary information 4

Scatter plot phosphoproteome experiment 1 technical replicate A vs. B N-starvation/NO_3^- (H/L)

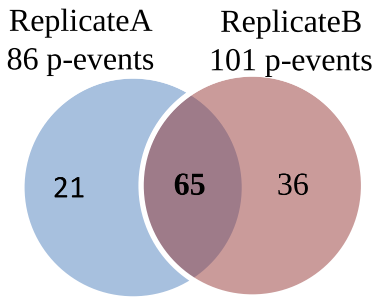

65	Number of valid pairs
25.59	Valid pairs percentage
0.905	Pearson correlation

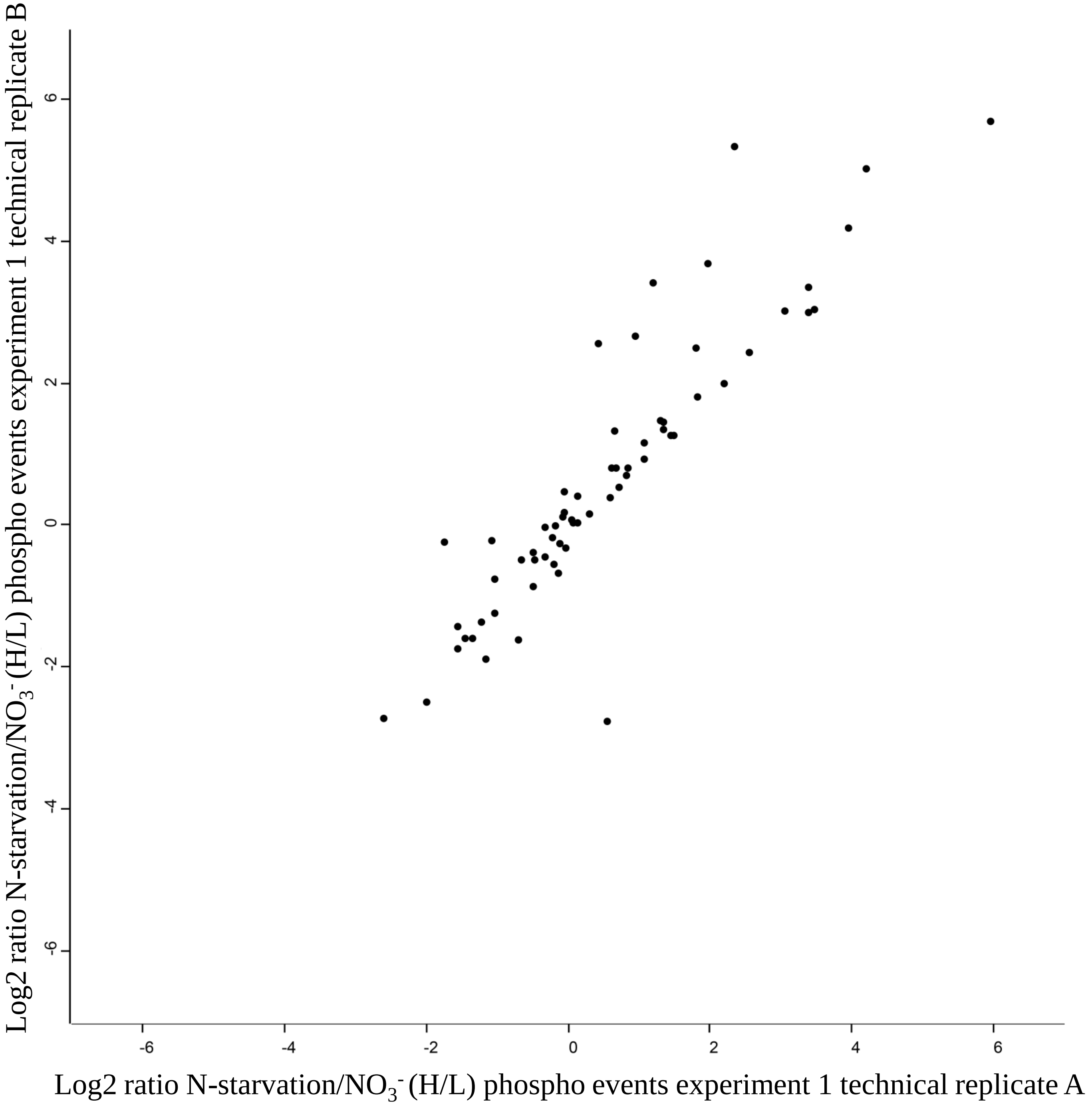

Supplementary information 4

Scatter plot phosphoproteome experiment 1 technical replicate A vs. B N-starvation/NH_4^+ (H/M)

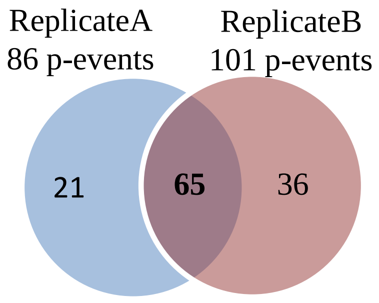

65	Number of valid pairs
25.59	Valid pairs percentage
0.937	Pearson correlation

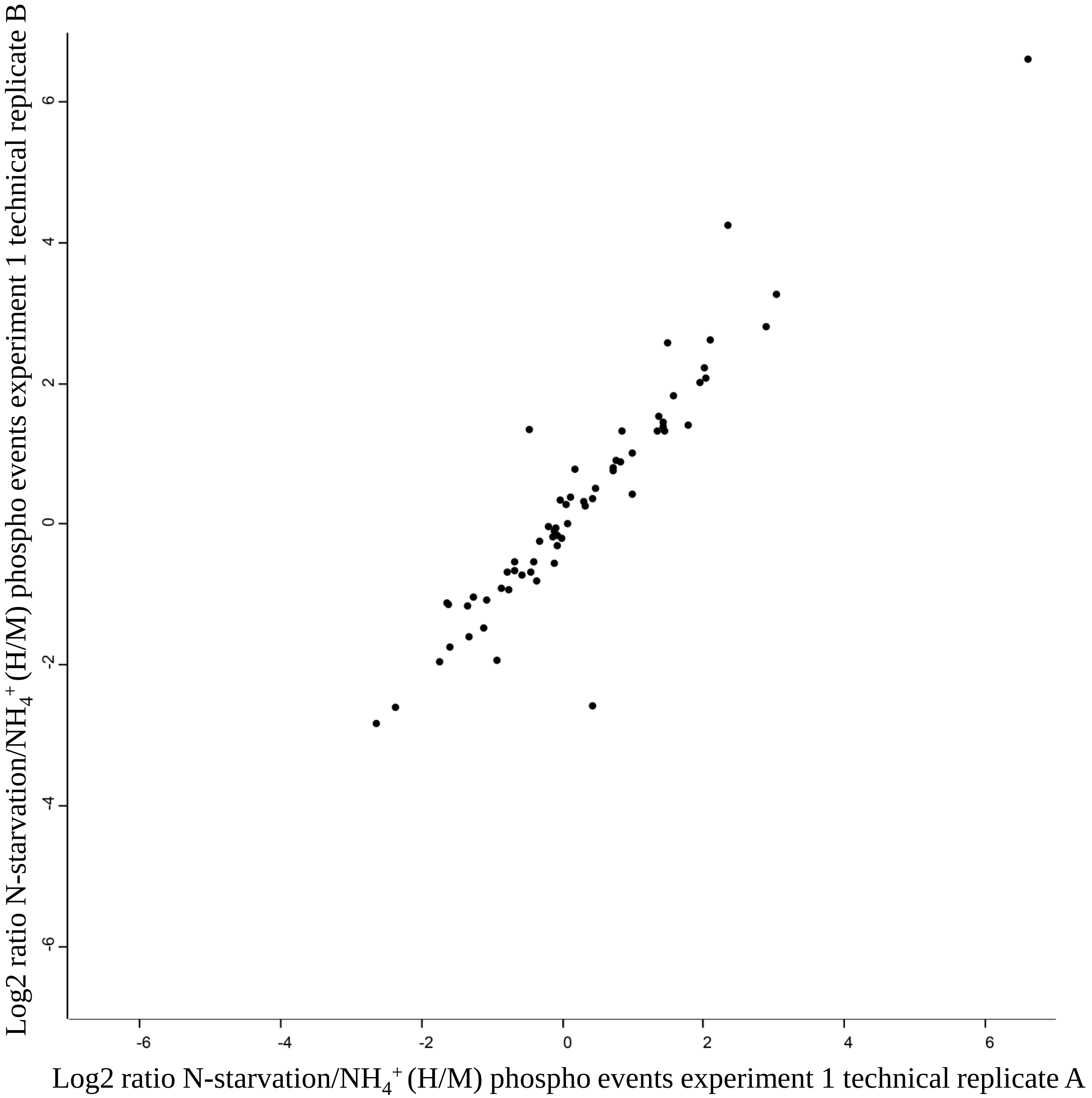

Supplementary information 4

Scatter plot phosphoproteome experiment 2 technical replicate A vs. B N-starvation/NO_3^- (H/L)

67	Number of valid pairs
26.38	Valid pairs percentage
0.948	Pearson correlation

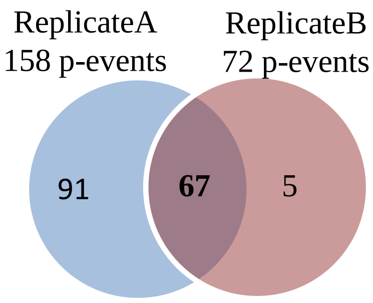

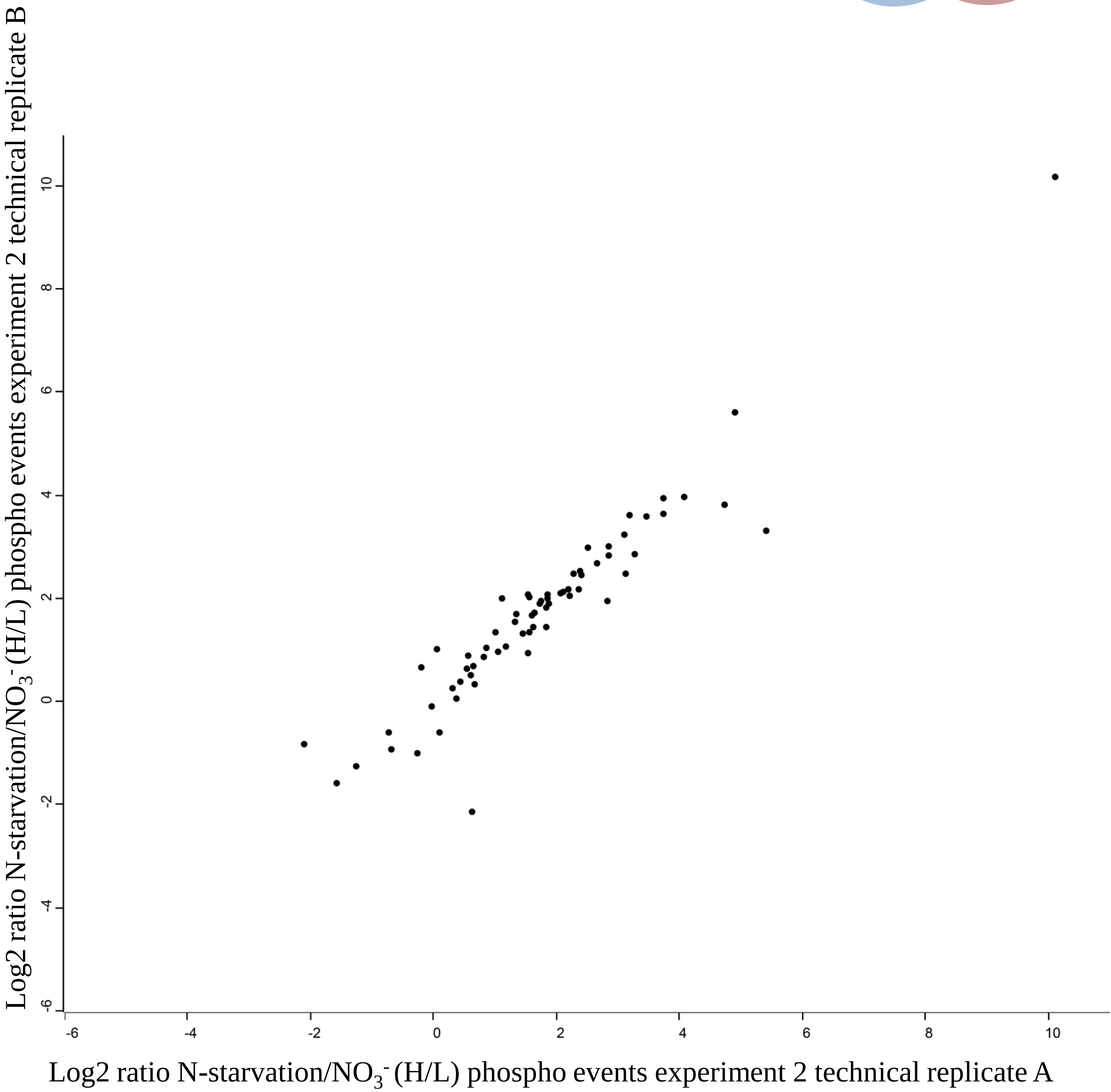

Supplementary information 4

Scatter plot phosphoproteome experiment 2 technical replicate A vs. B N-starvation/NH_4^+ (H/M)

67	Number of valid pairs
26.38	Valid pairs percentage
0.913	Pearson correlation

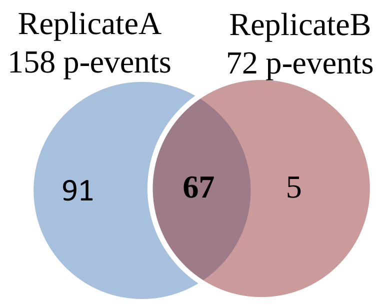

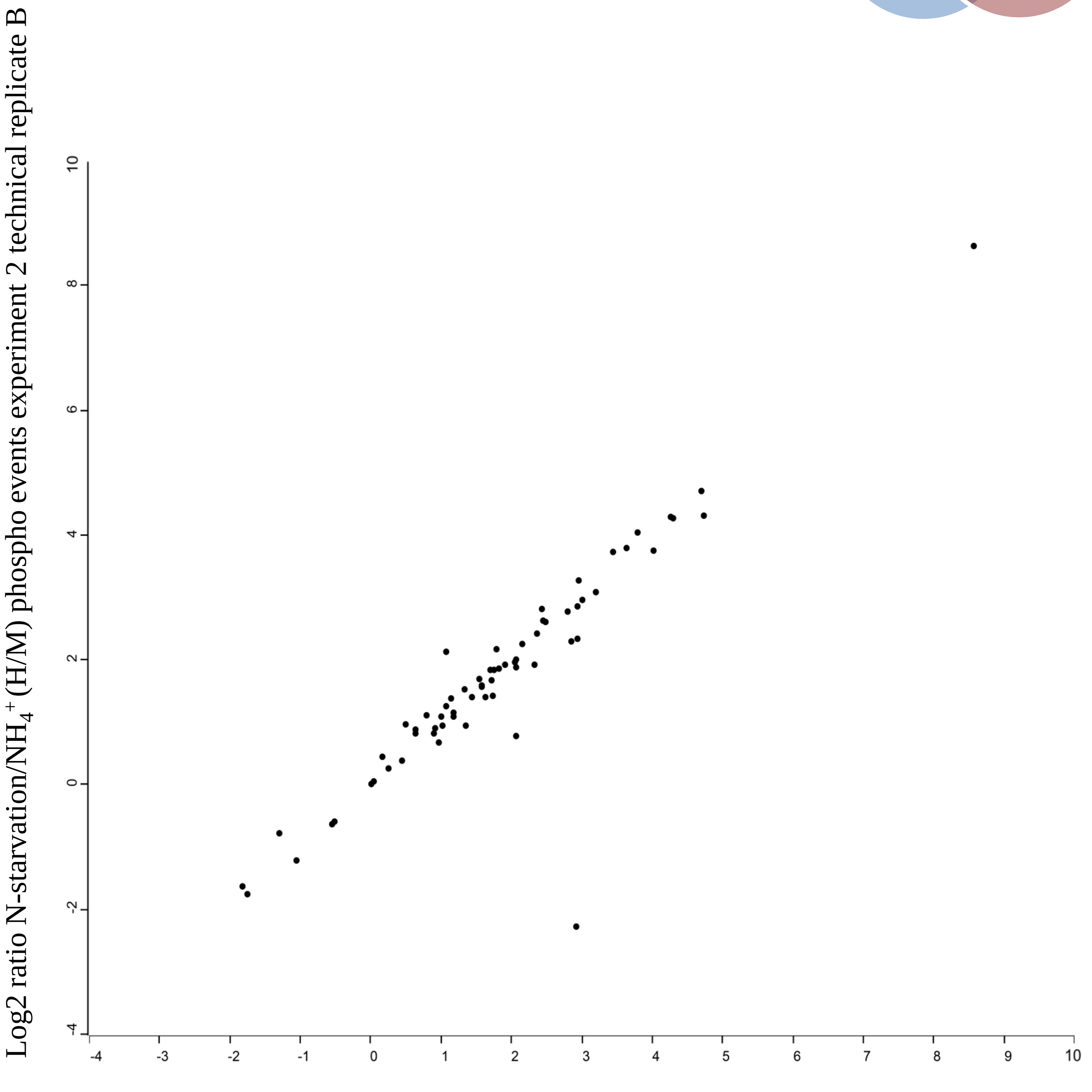

Scatter plot proteome experiment 1 vs. 2
N-starvation/NO_3^- (H/L)

1874	Number of valid pairs
78.67	Valid pairs percentage
0.758	Pearson correlation

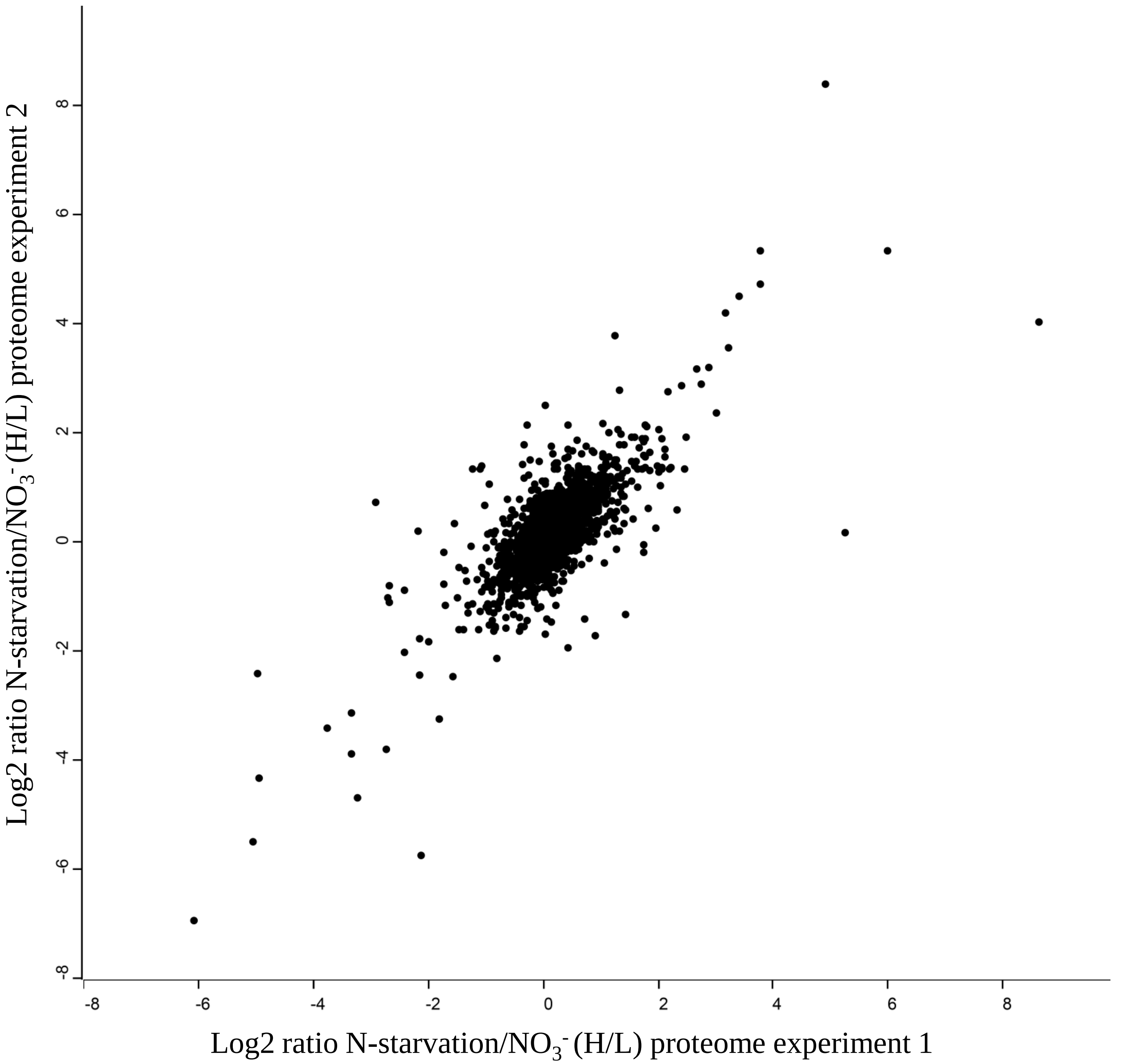

Supplementary information 5

Scatter plot proteome experiment 1 vs. 2
N-starvation/NH_4^+ (H/M)

1878	Number of valid pairs
78.84	Valid pairs percentage
0.867	Pearson correlation

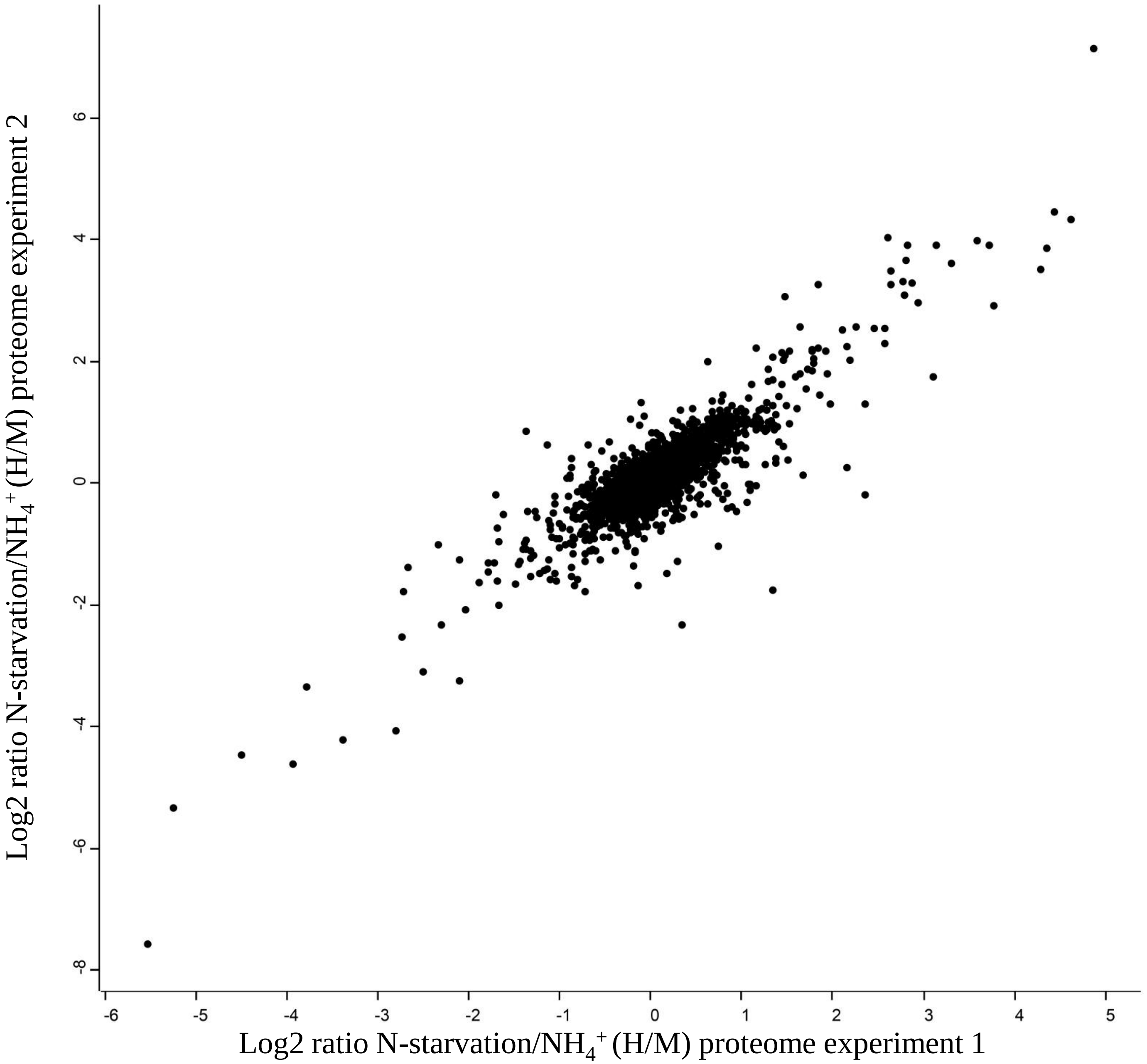

Supplementary information 5

Scatter plot phosphoproteome experiment 1 vs. 2
N-starvation/NO_3^- (H/L)

87	Number of valid pairs
46.03	Valid pairs percentage
0.687	Pearson correlation

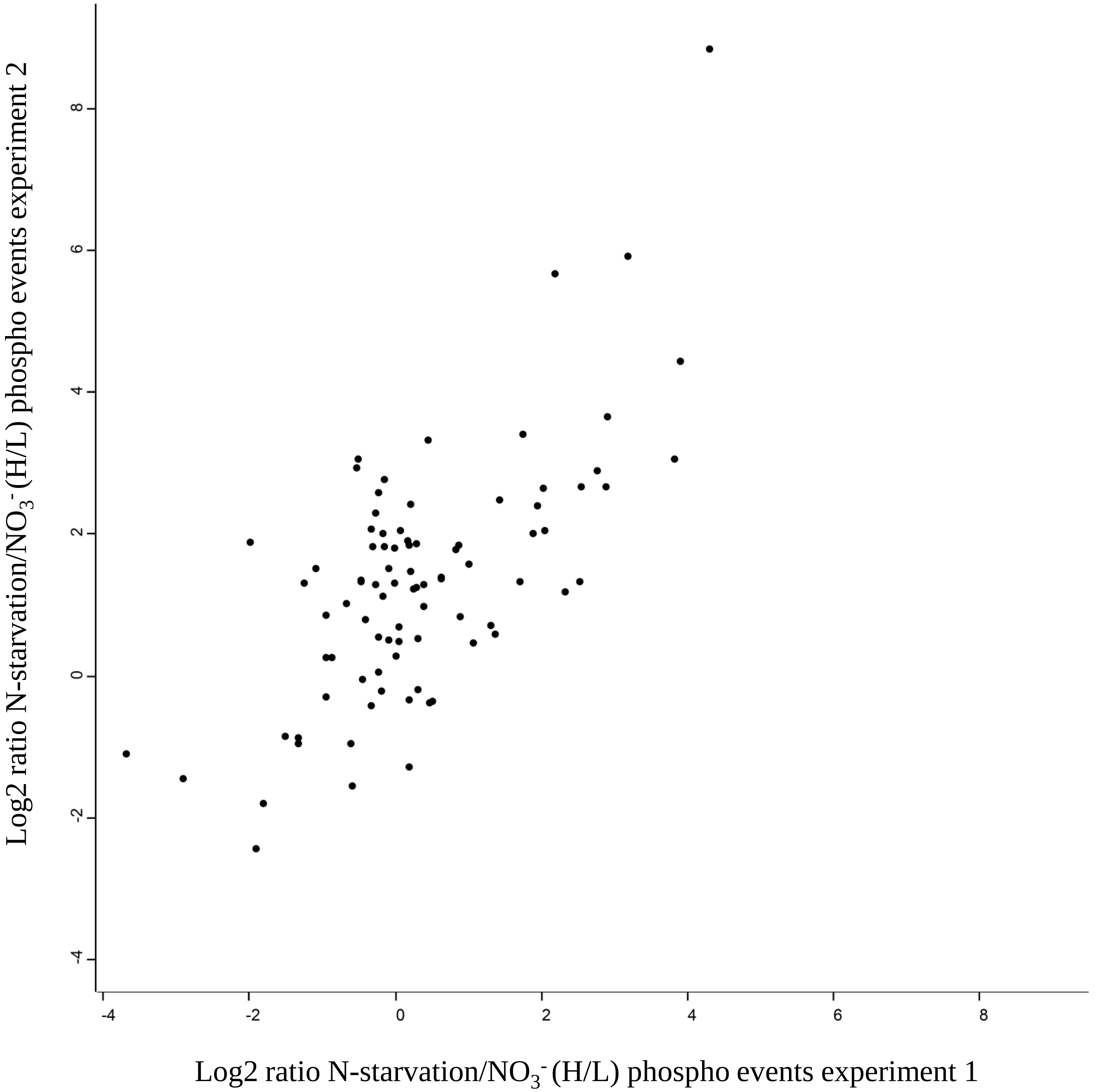

Scatter plot phosphoproteome experiment 1 vs. 2 N-starvation/NH_4^+ (H/M)

89	Number of valid pairs
47.09	Valid pairs percentage
0.638	Pearson correlation

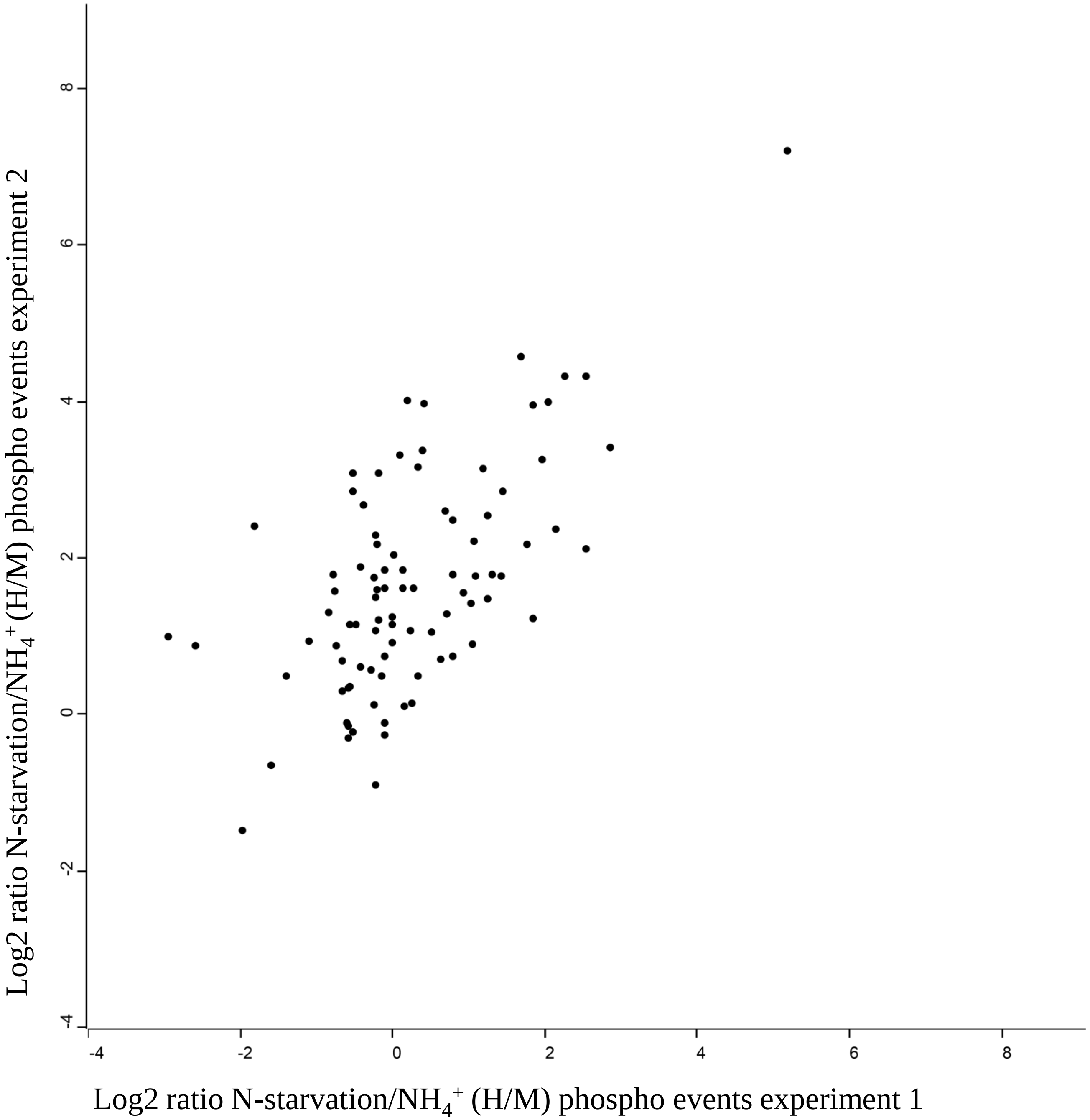

Supplementary information 6

Supplementary table to Figure 3, indicating the protein IDs of classified proteins.

	Experiment 1		Experiment 2	
	**InterPro term**	**Cyanobase protein ID**	**InterPro term**	**Cyanobase protein ID**
N-starvation /nitrate	**static:**		**static:**	
	Globin-like proteins:	sll0928 sll1577 slr2067	Globin-like proteins:	sll1577 sll1578 slr2067
	Anti-sigma antagonists:	slr1856 slr1859		
			**up-regulated:**	
	**up-regulated:**		PSI proteins:	sll0226 slr0737
	adenylyl/guanylyl cyclase:	slr1102 slr1103	adenylyl/guanylyl cyclase:	slr1102 slr1103
			P-loop NTPase:	sll0877 sll1525
			RuBisCo:	slr0009 slr0012
			P_{II} and P_{II}-like:	slr1513 ssl0707
N-starvation /ammonia	**static:**		**static:**	
	Globin-like proteins:	sll0928 sll1577 sll1578 slr2067	Globin-like proteins:	sll1577 sll1578 slr2067
	adenylyl/guanylyl cyclase:	slr1102 slr1103		
	Anti-sigma antagonists:	slr1856 slr1859	**up-regulated:**	
			PSI proteins:	slr0737 slr1655
			RuBisCo:	slr0009 slr0012
			P_{II} and P_{II}-like:	slr1513 ssl0707
			Anti-sigma antagonists:	slr1856 slr1859
			adenylyl/guanylyl cyclase:	slr1102 slr1103
