# Supplementary material for: Phosphoproteome of the cyanobacterium Synechocystis sp. PCC 6803 and its dynamics during nitrogen starvation
Source: Front Microbiol. 2015 Mar 31;6:248. doi: 10.3389/fmicb.2015.00248 (PMC4379935; doi:10.3389/fmicb.2015.00248)

Qualitative Dataset

Phosphopeptide MS/MS Spectra (CID)

Localized phosphorylation events

Raw file	Scan	Method	Score	m/z
20130708_PS_Phosphotest_6803wt_SDS04	10023	ITMS; CID	106.89	975.46

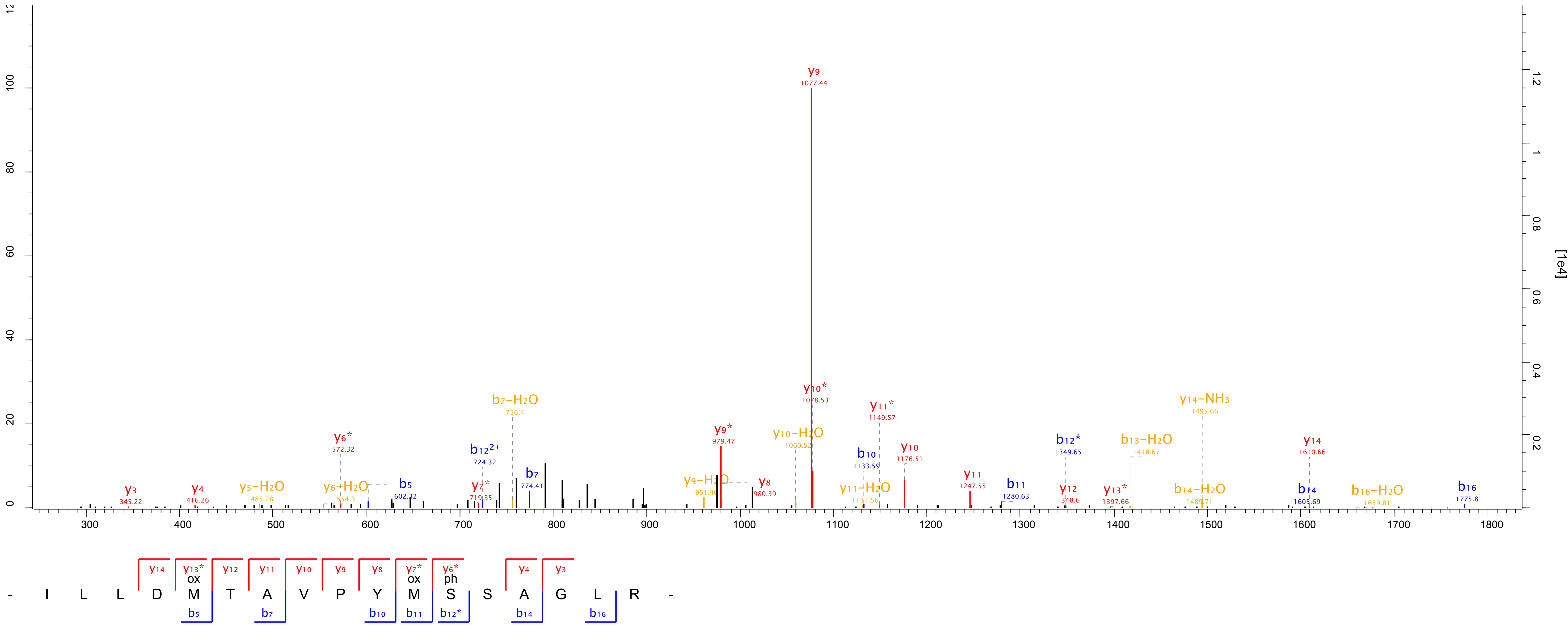

Raw file	Scan	Method	Score	m/z
20130708_PS_Phosphotest_6803wt_SDS01	10119	ITMS; CID	135.91	639.3

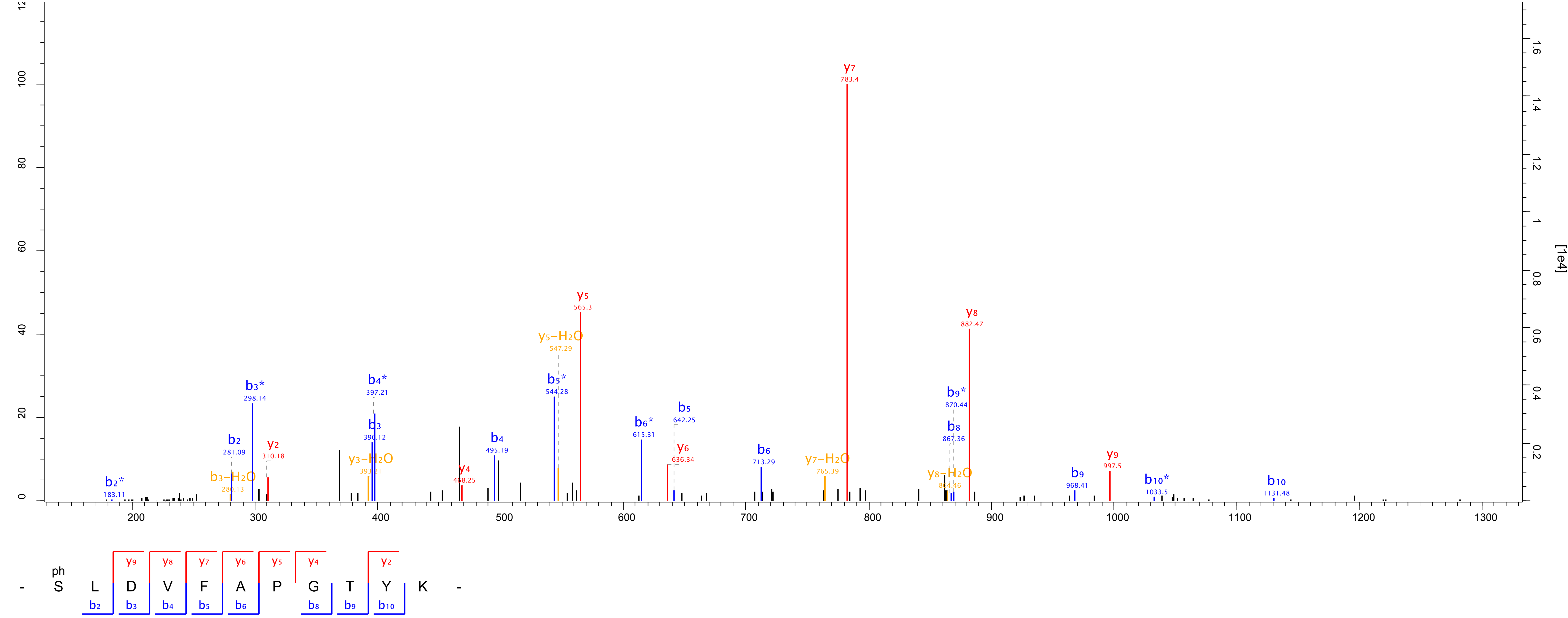

Raw file	Scan	Method	Score	m/z
20130708_PS_Phosphotest_6803wt_SDS01	10121	ITMS; CID	76.01	703.82

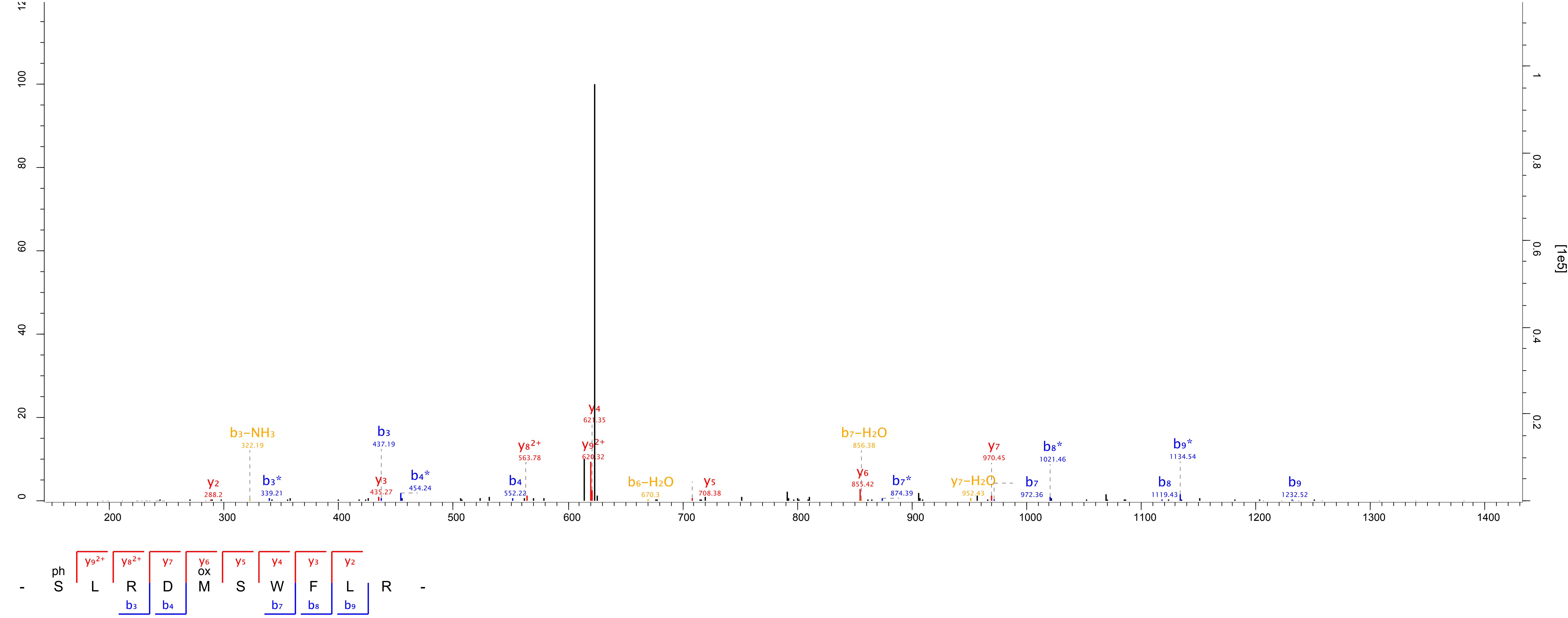

Raw file	Scan	Method	Score	m/z
20130708_PS_Phosphotest_6803wt_SDS01	10134	ITMS; CID	138.2	596.8

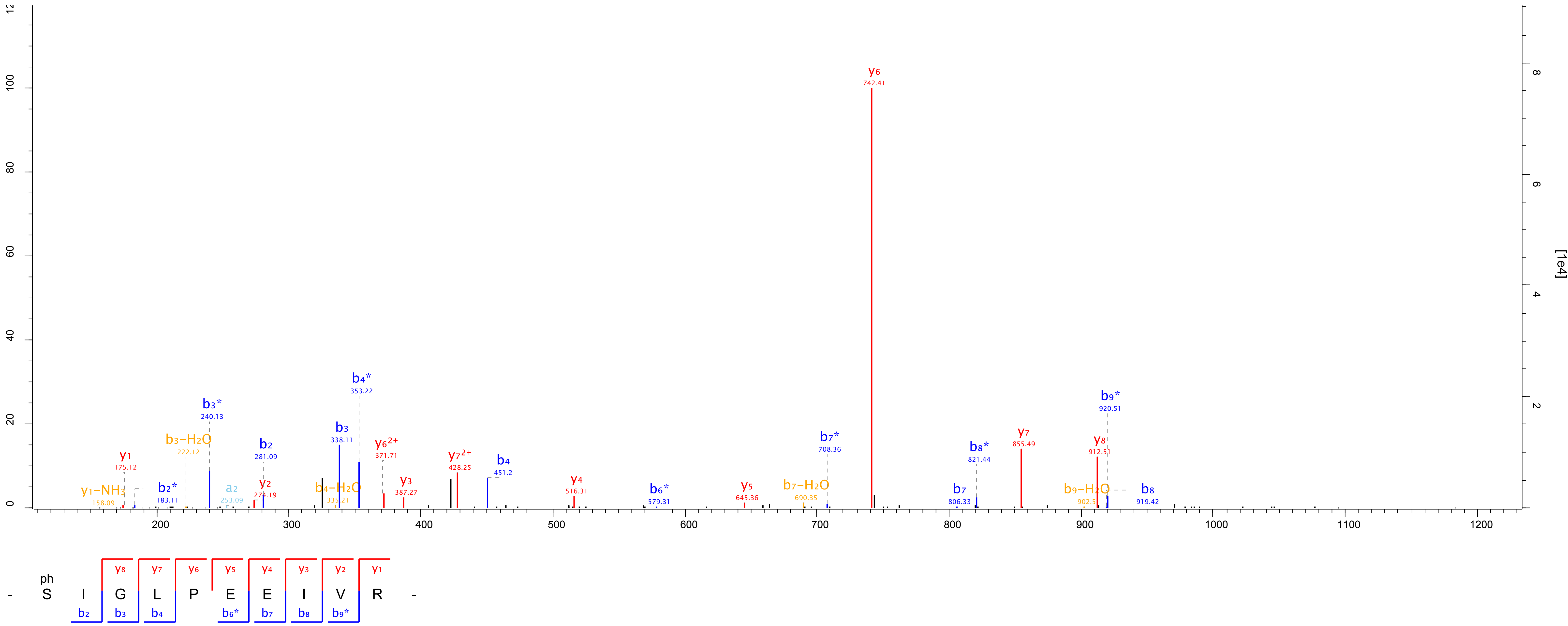

Raw file	Scan	Method	Score	m/z
20130708_PS_Phosphotest_6803wt_SDS01	10137	ITMS; CID	94.56	952.45

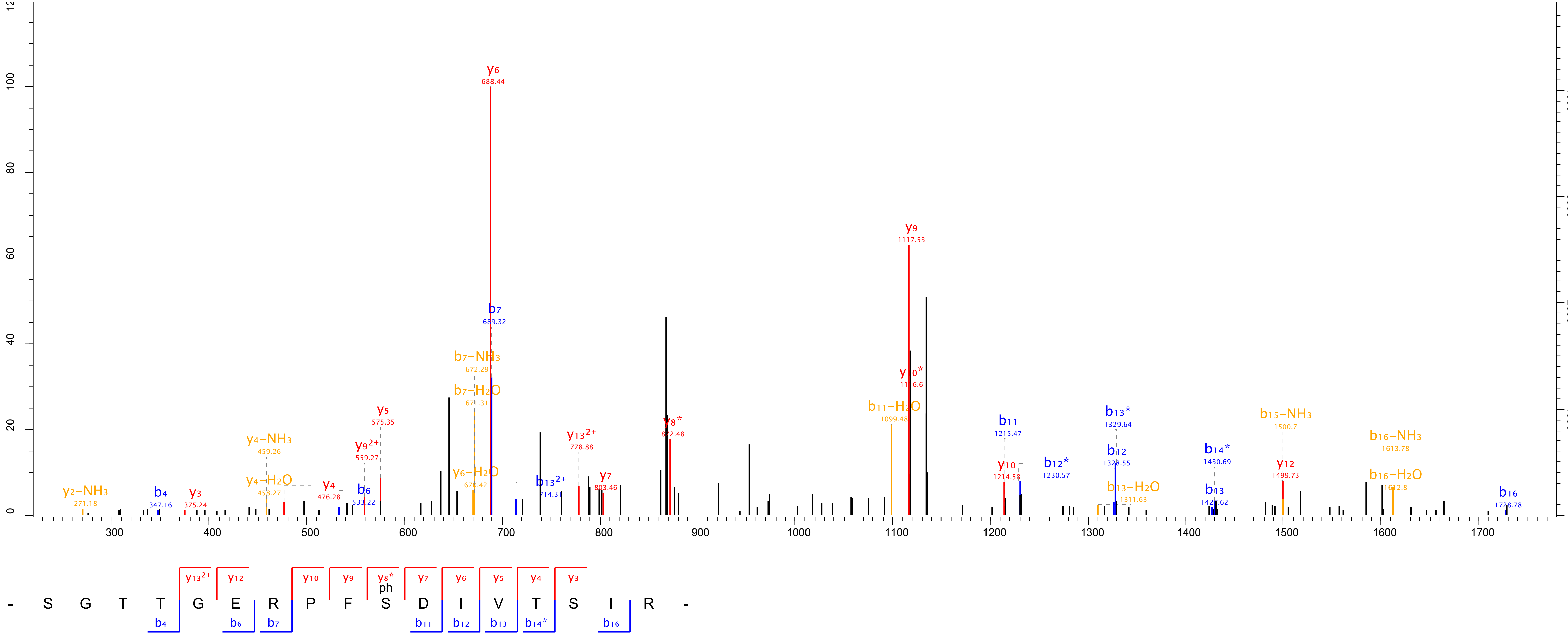

Raw file	Scan	Method	Score	m/z
20130708_PS_Phosphotest_6803wt_SDS01	10154	ITMS; CID	121.95	813.36

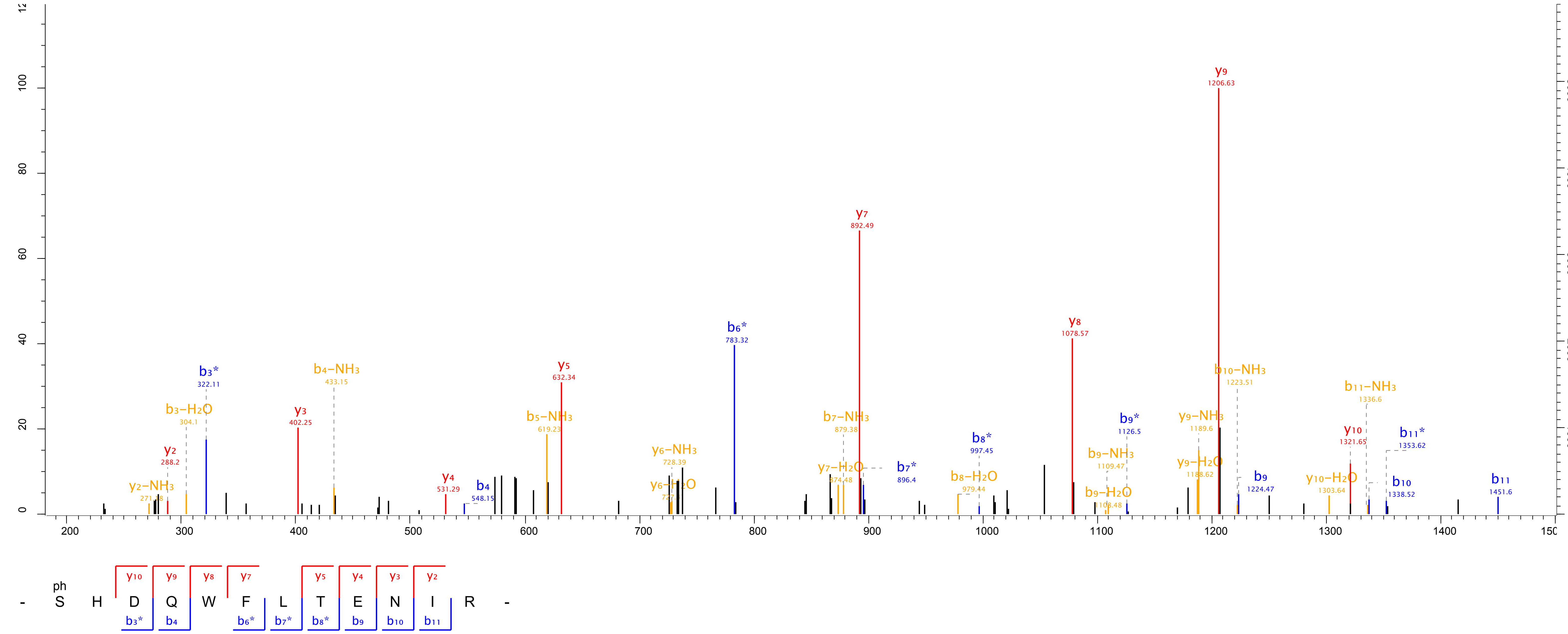

Raw file	Scan	Method	Score	m/z
20130708_PS_Phosphotest_6803wt_SDS01	10221	ITMS; CID	121.75	1126.56

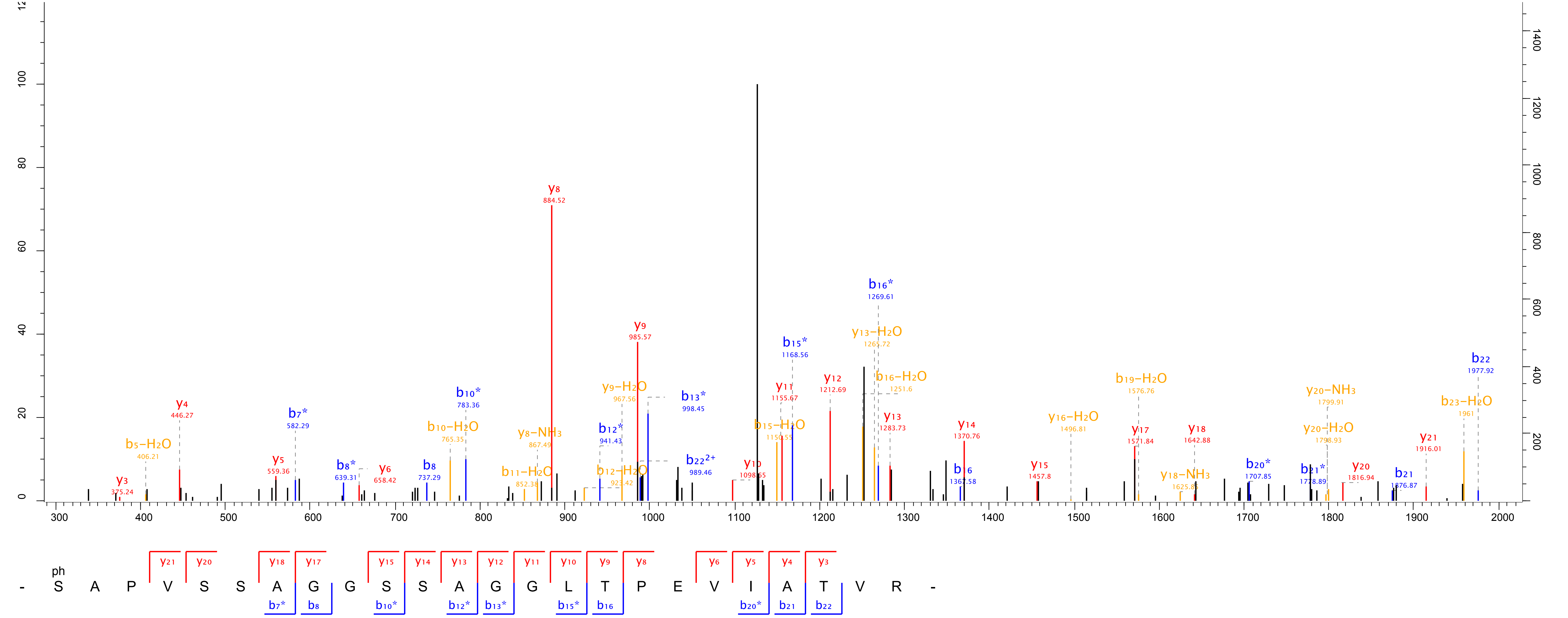

Raw file	Scan	Method	Score	m/z
20130708_PS_Phosphotest_6803wt_SDS01	10224	ITMS; CID	145.83	615.31

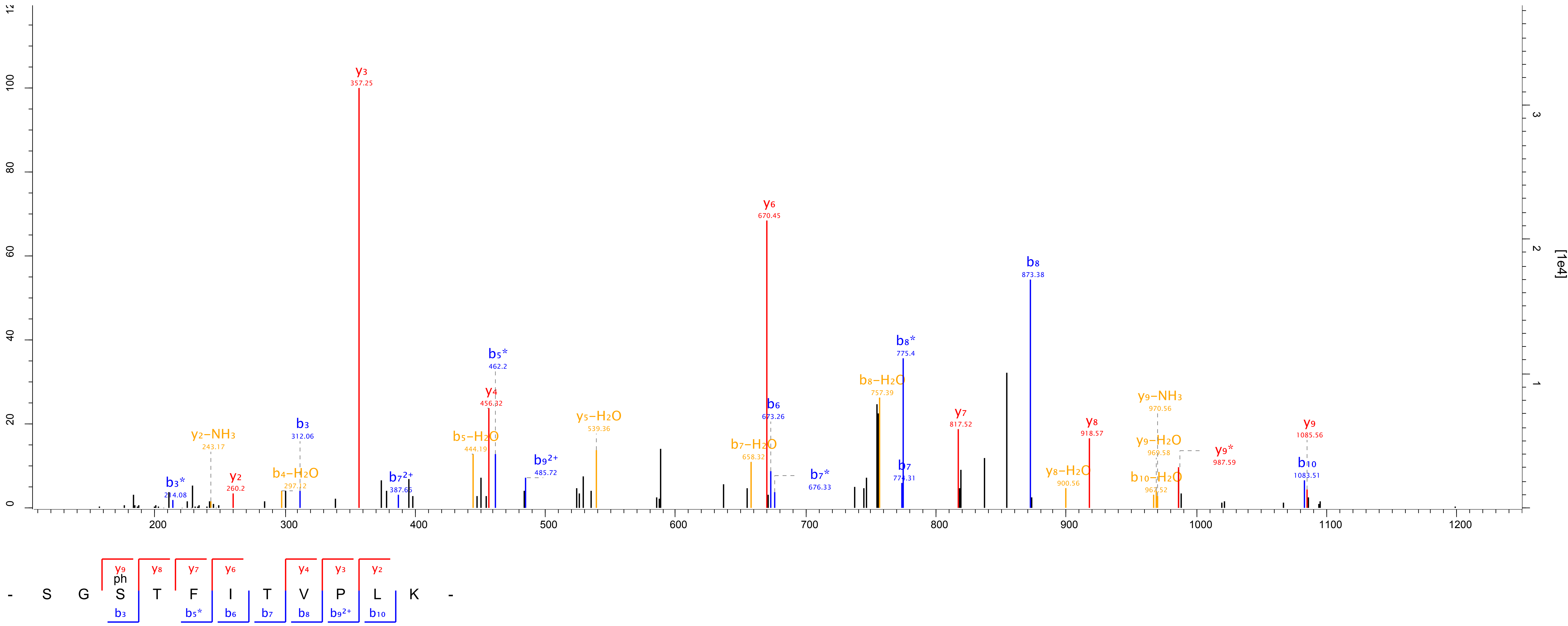

Raw file	Scan	Method	Score	m/z
20130708_PS_Phosphotest_6803wt_SDS01	10230	ITMS; CID	87.22	547.29

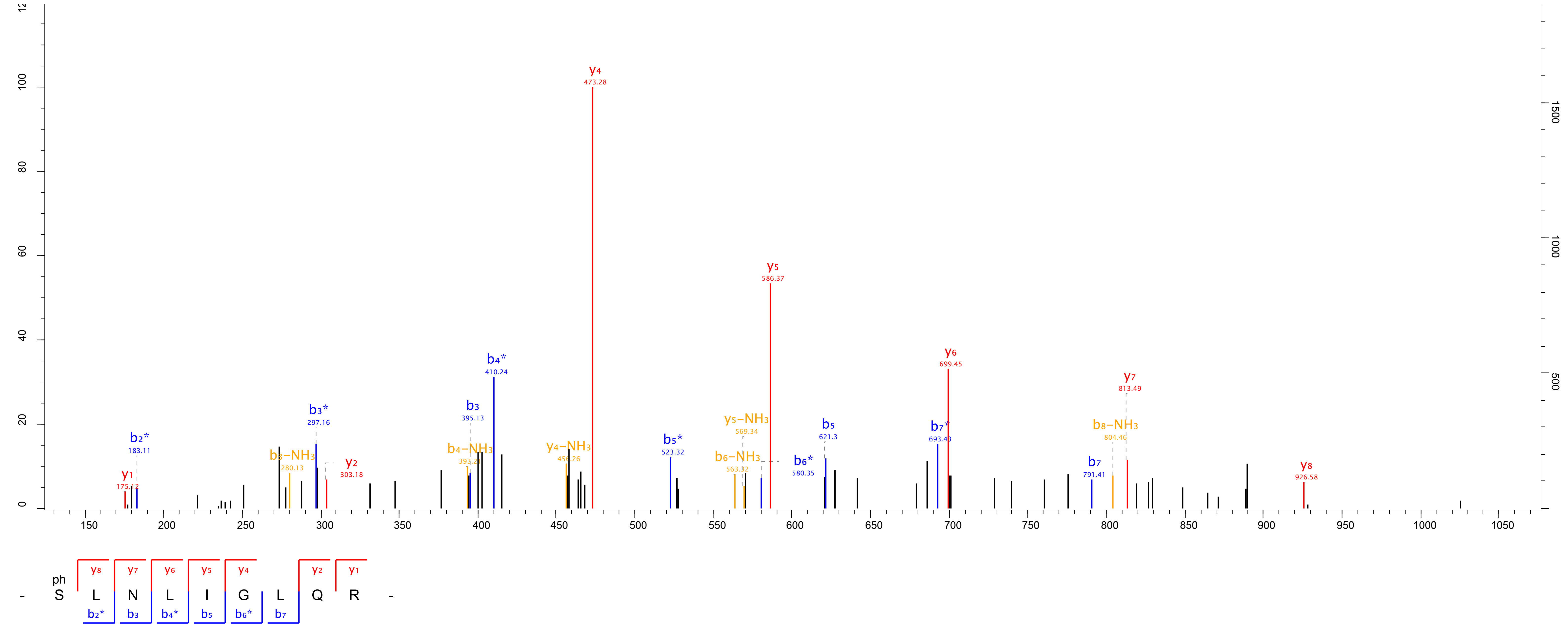

Raw file	Scan	Method	Score	m/z
20130708_PS_Phosphotest_6803wt_YPER01	10258	ITMS; CID	85.85	1158.55

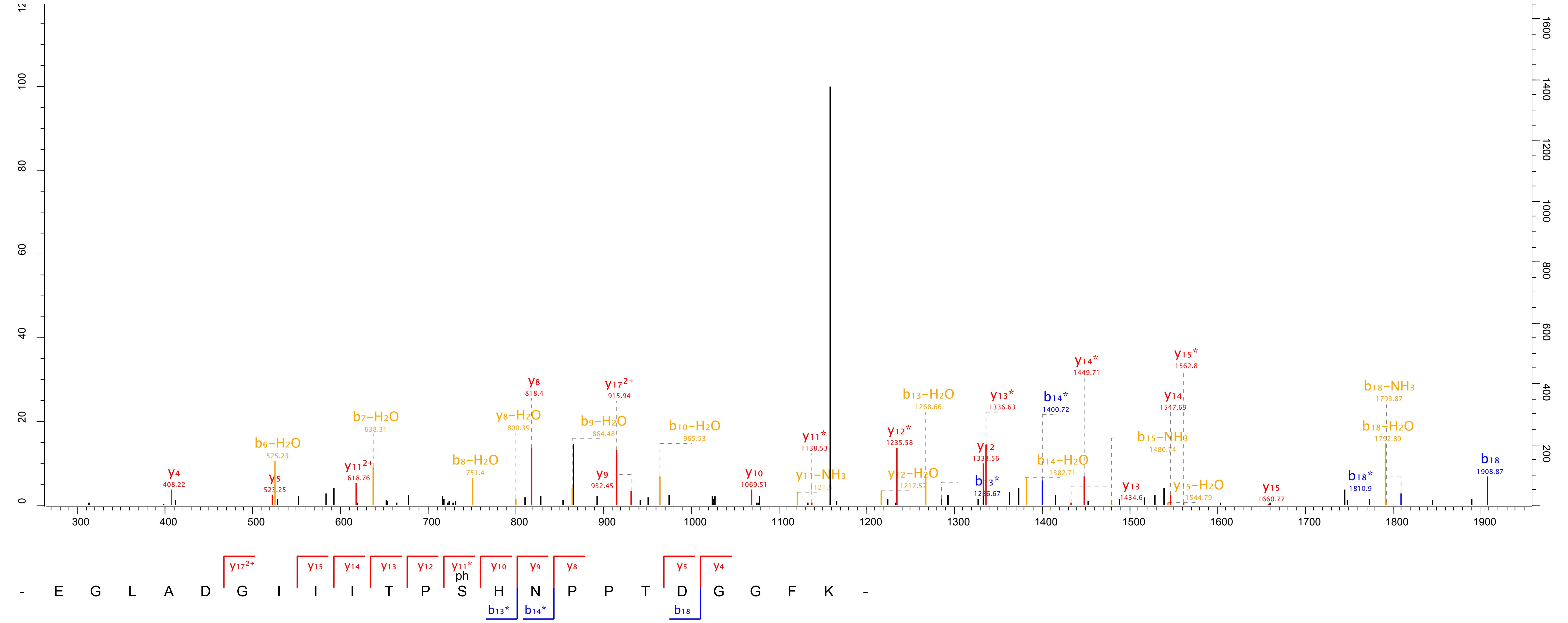

Raw file	Scan	Method	Score	m/z
20130708_PS_Phosphotest_6803wt_SDS02	10309	ITMS; CID	132.97	593.3

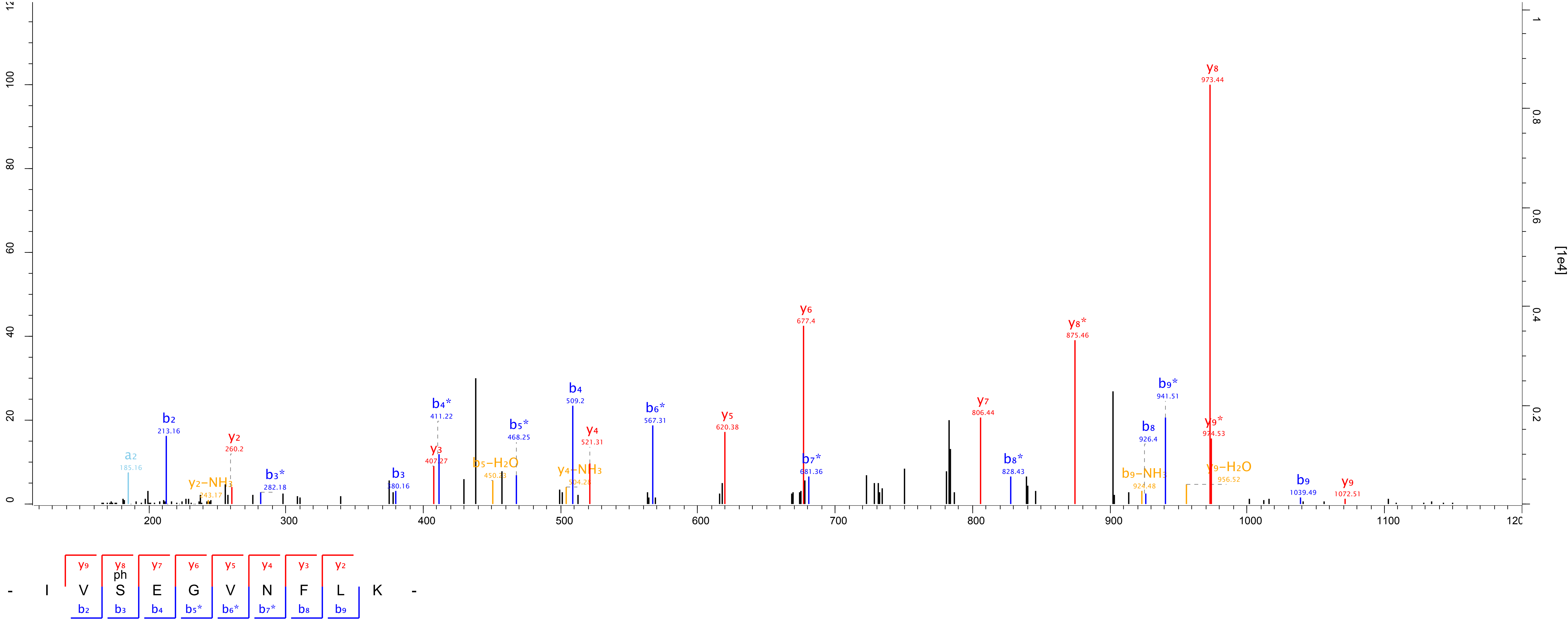

Raw file	Scan	Method	Score	m/z
20130708_PS_Phosphotest_6803wt_SDS01	10319	ITMS; CID	127.17	636.82

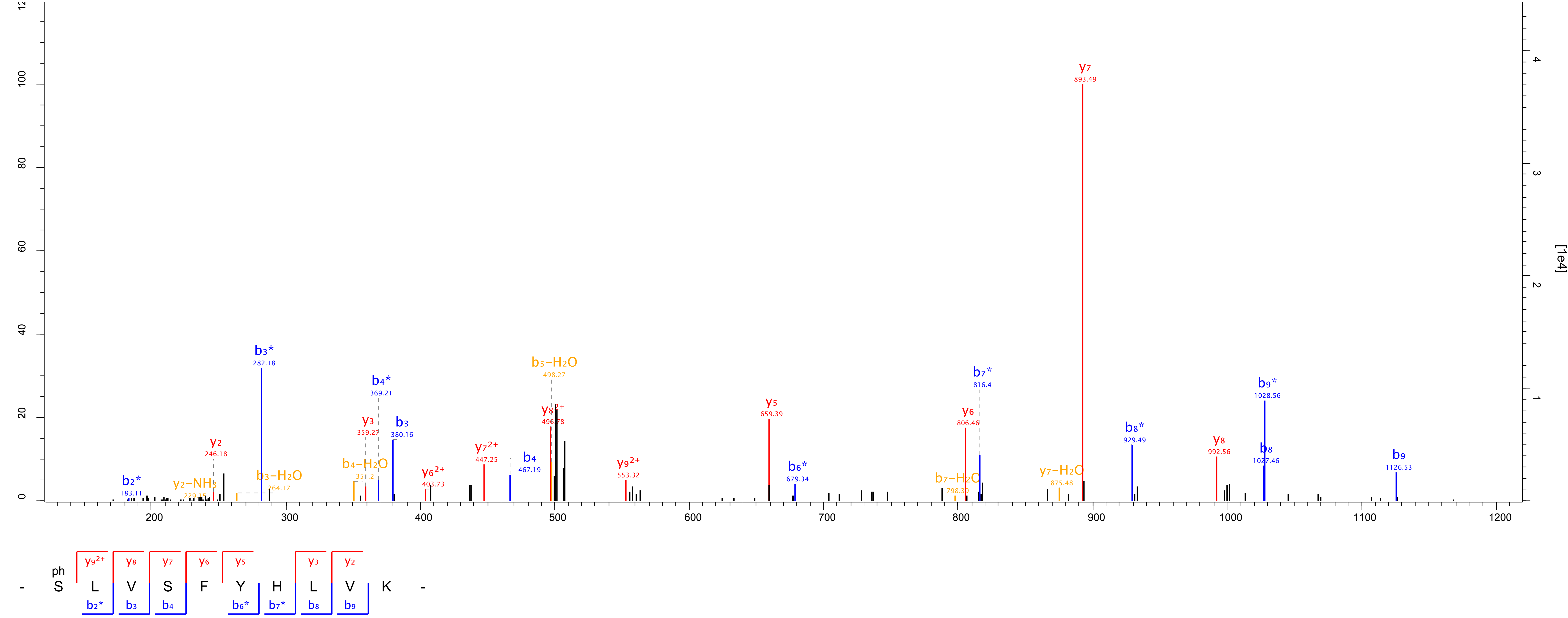

Raw file	Scan	Method	Score	m/z
20130708_PS_Phosphotest_6803wt_SDS01	10337	ITMS; CID	178.14	698.84

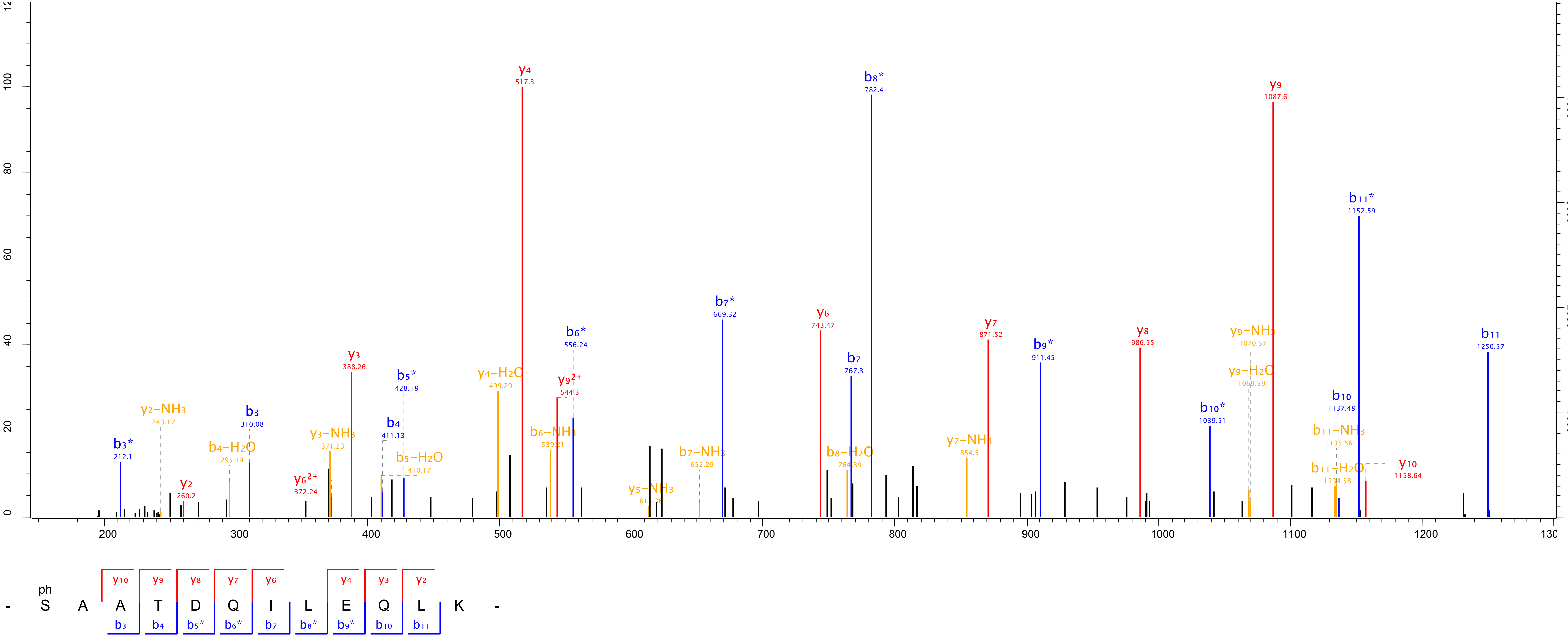

Raw file	Scan	Method	Score	m/z
20130708_PS_Phosphotest_6803wt_SDS01	10399	ITMS; CID	141.36	959.48

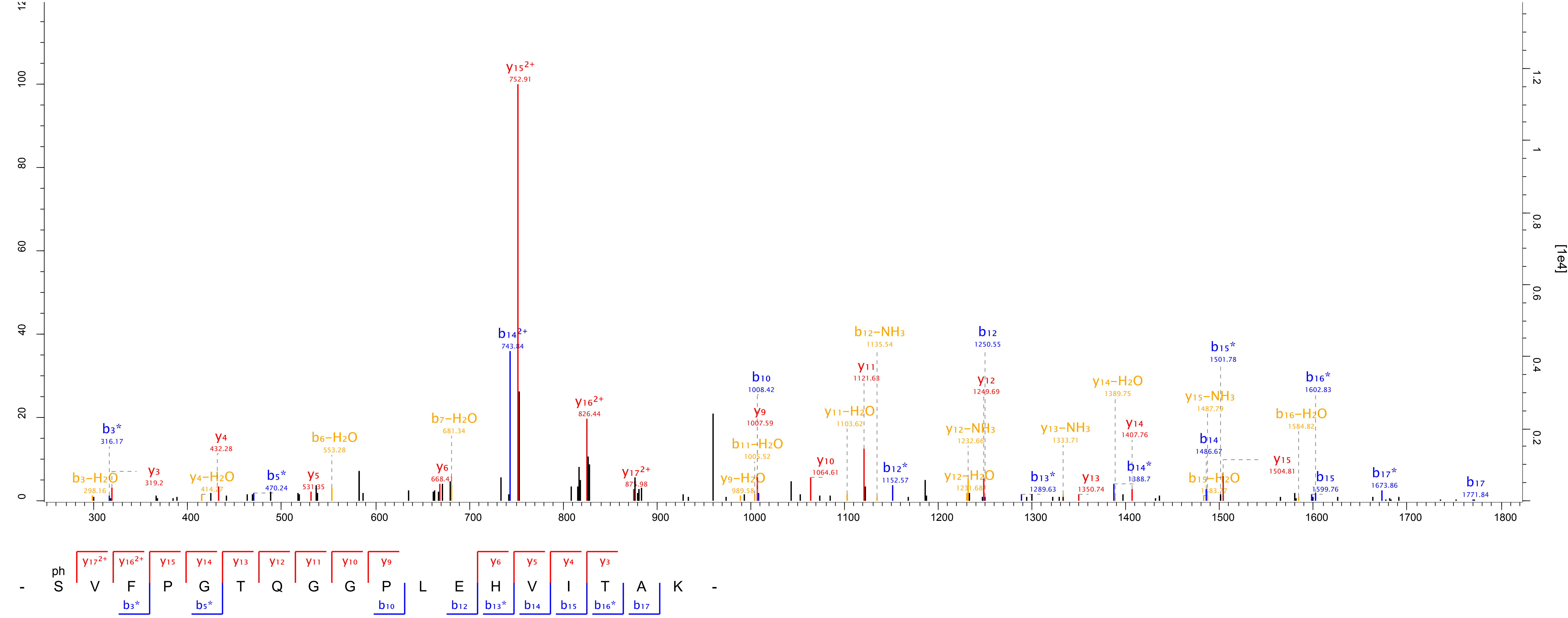

Raw file	Scan	Method	Score	m/z
20130708_PS_Phosphotest_6803wt_SDS02	10567	ITMS; CID	78.51	455.75

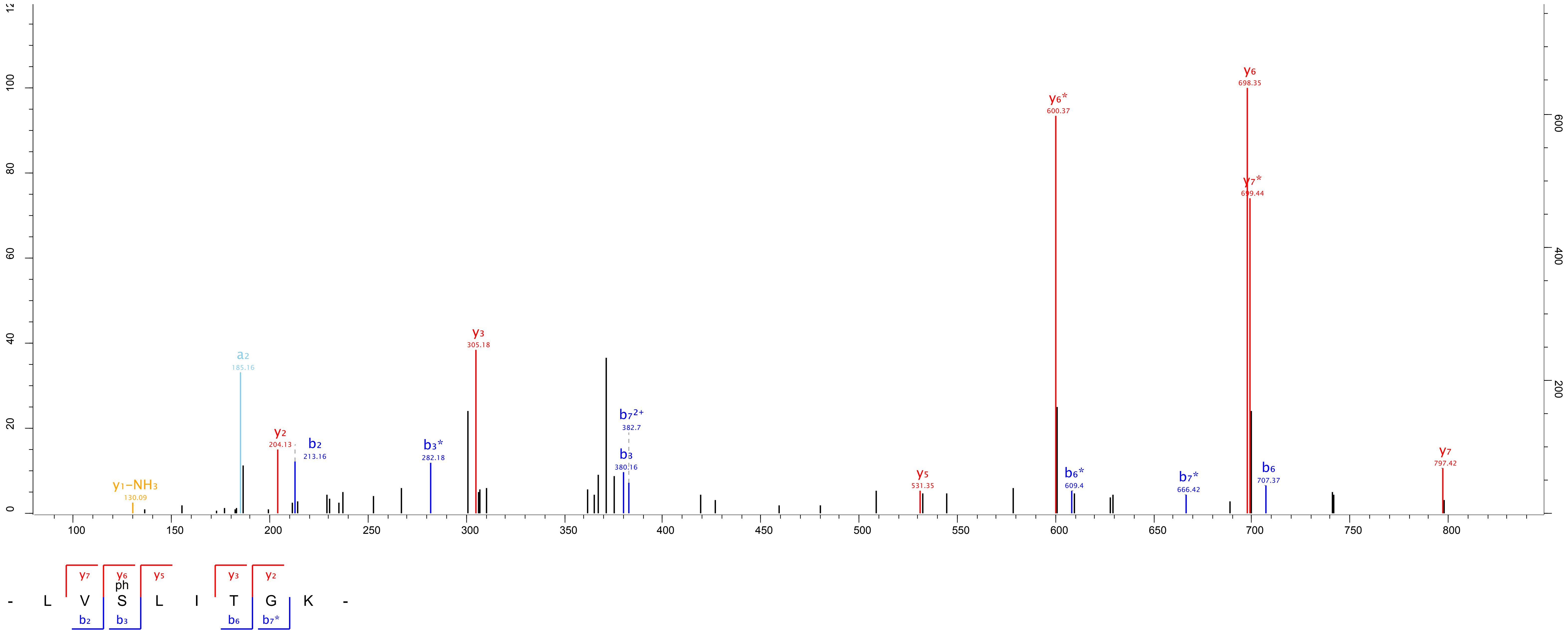

Raw file	Scan	Method	Score	m/z
20130708_PS_Phosphotest_6803wt_SDS01	10572	ITMS; CID	128.95	623.98

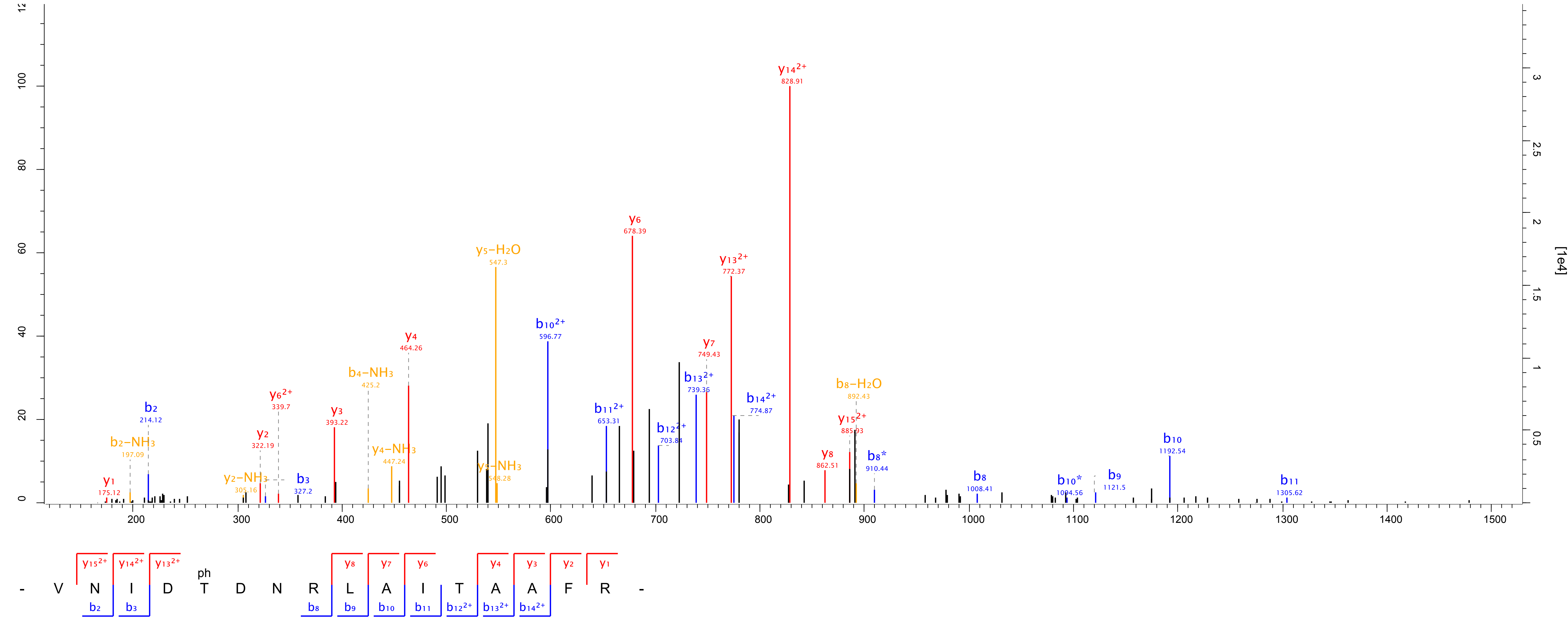

Raw file	Scan	Method	Score	m/z
20130708_PS_Phosphotest_6803wt_FASP01	10581	ITMS; CID	161.21	656.34

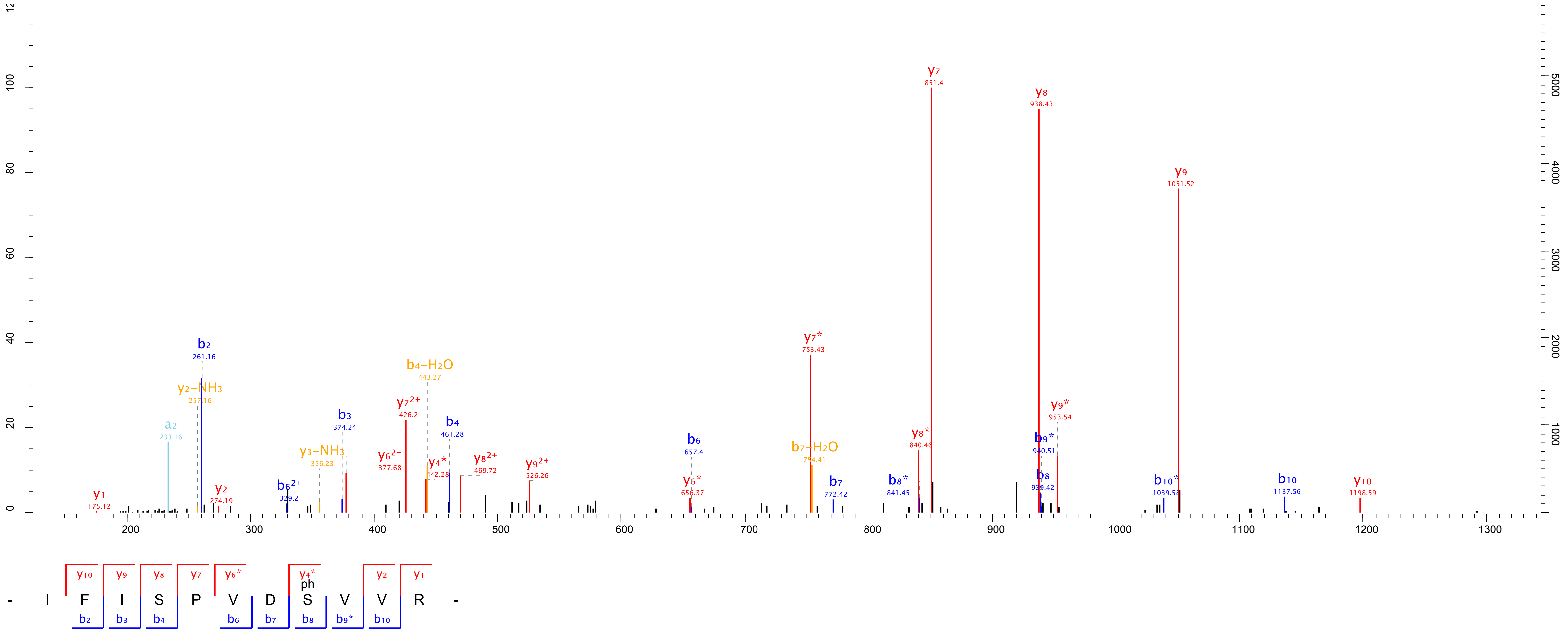

Raw file	Scan	Method	Score	m/z
20130708_PS_Phosphotest_6803wt_SDS03	10583	ITMS; CID	136.7	598.83

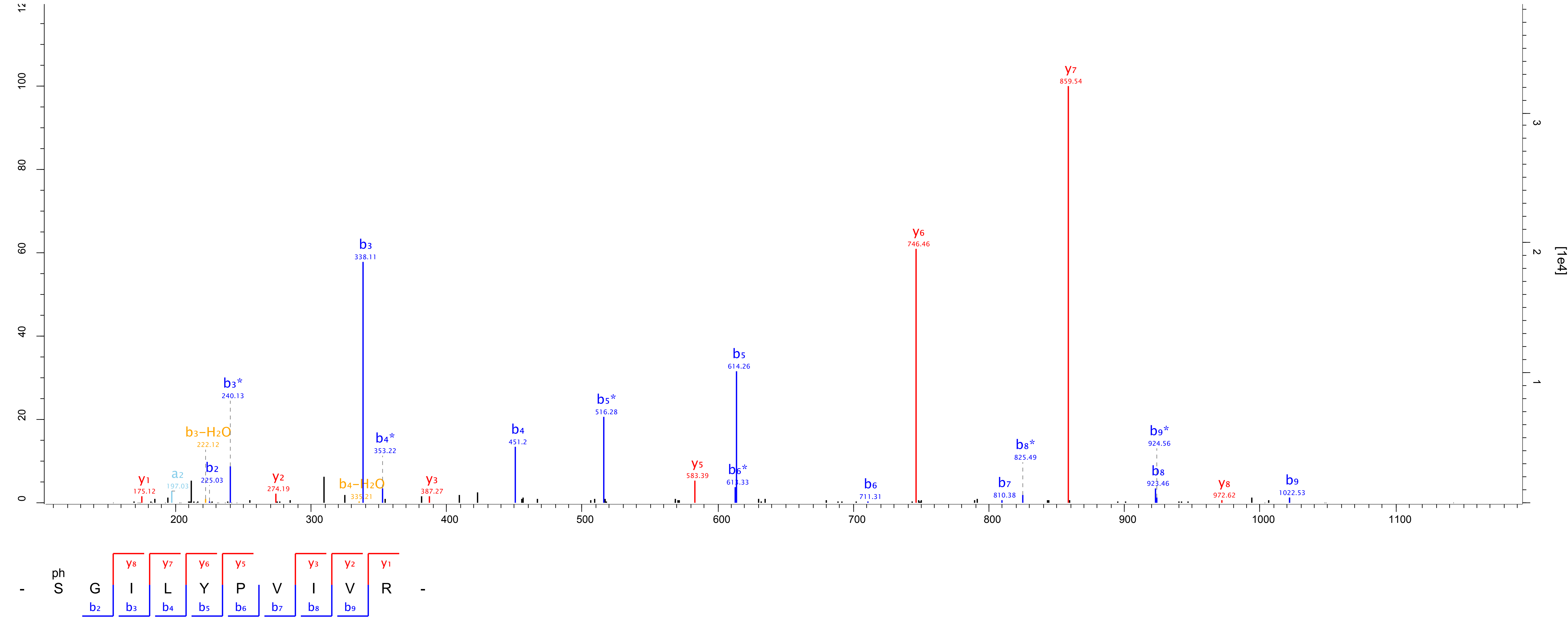

Raw file	Scan	Method	Score	m/z
20130708_PS_Phosphotest_6803wt_SDS01	10656	ITMS; CID	93.39	1097.99

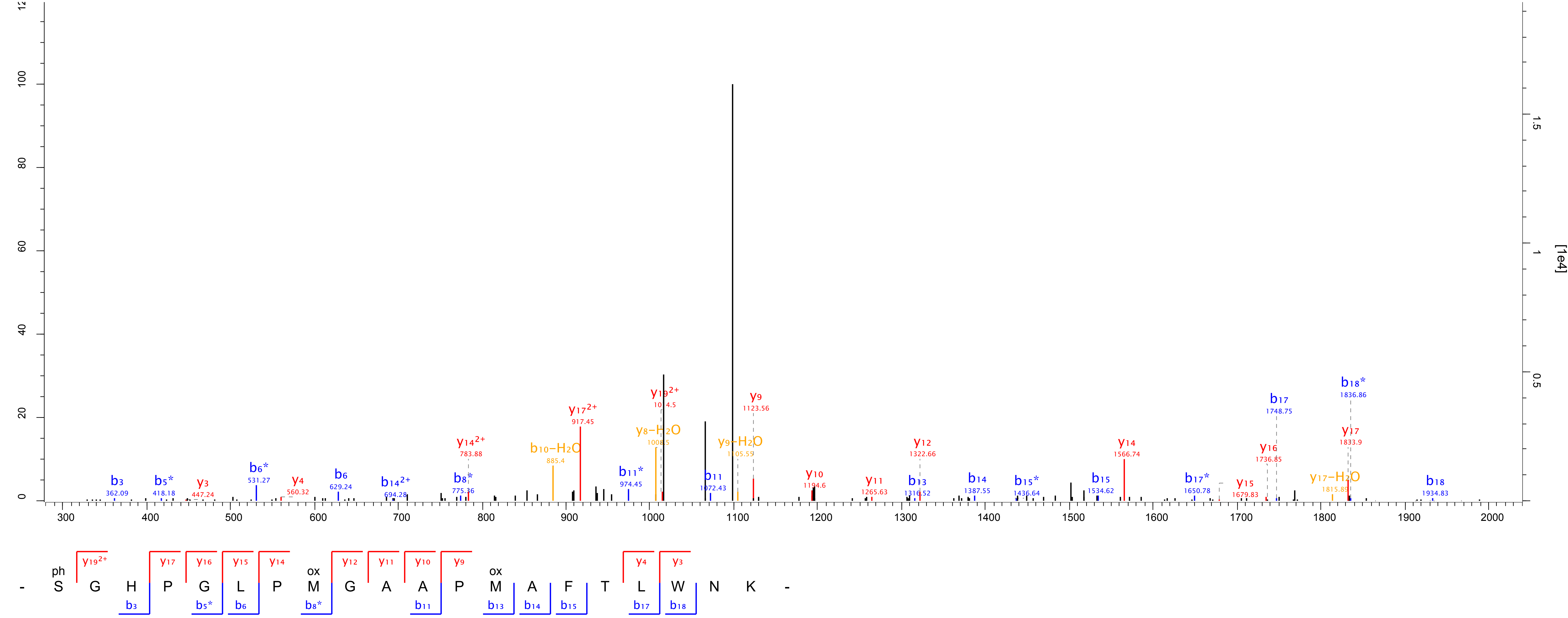

Raw file	Scan	Method	Score	m/z
20130708_PS_Phosphotest_6803wt_SDS01	10673	ITMS; CID	139.77	656.34

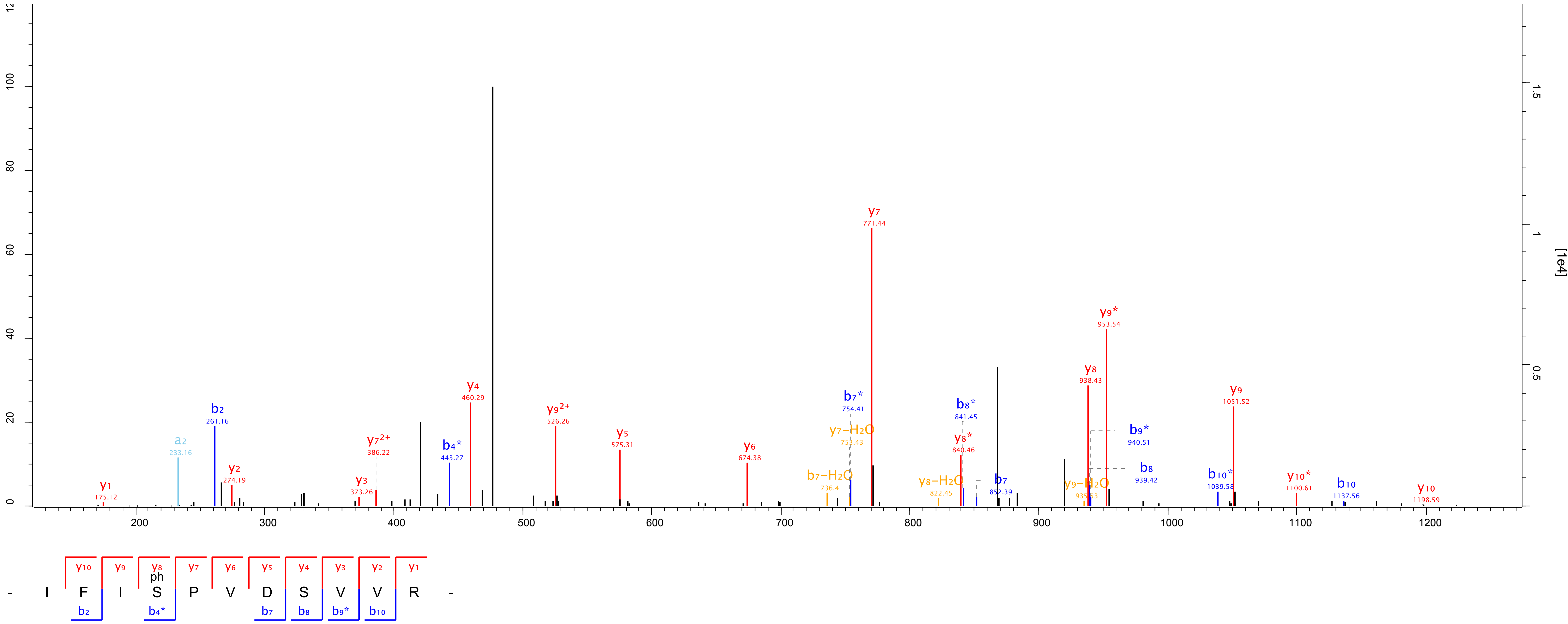

Raw file	Scan	Method	Score	m/z
20130708_PS_Phosphotest_6803wt_SDS01	10721	ITMS; CID	161.93	577.79

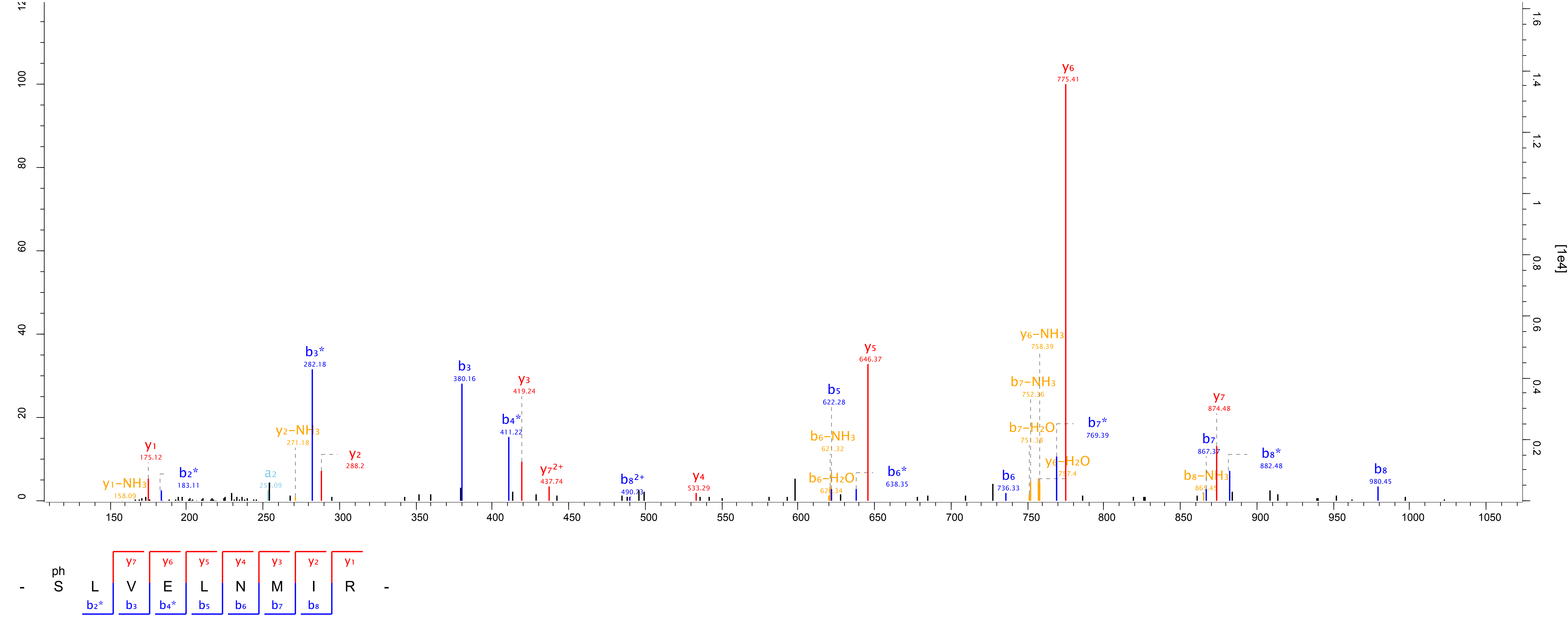

Raw file	Scan	Method	Score	m/z
20130708_PS_Phosphotest_6803wt_SDS01	10745	ITMS; CID	73.88	630.79

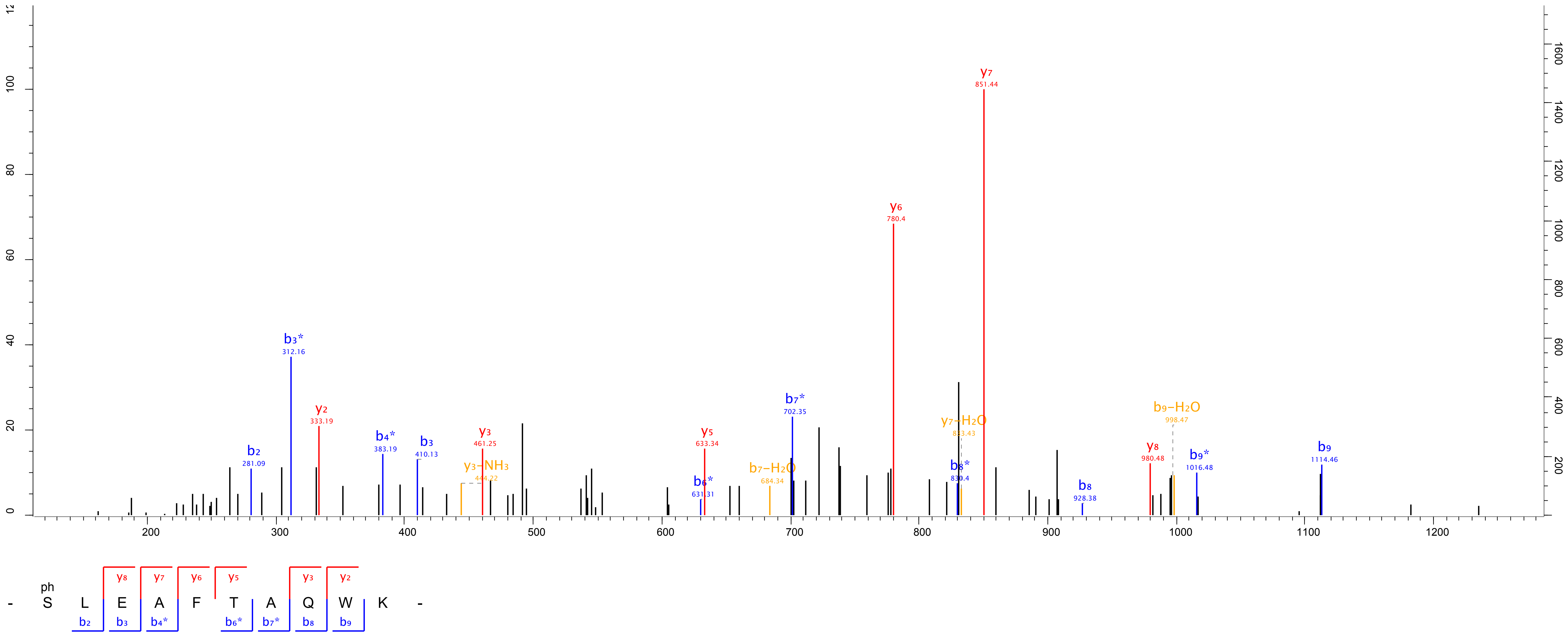

Raw file	Scan	Method	Score	m/z
20130708_PS_Phosphotest_6803wt_SDS01	10905	ITMS; CID	174.7	892.42

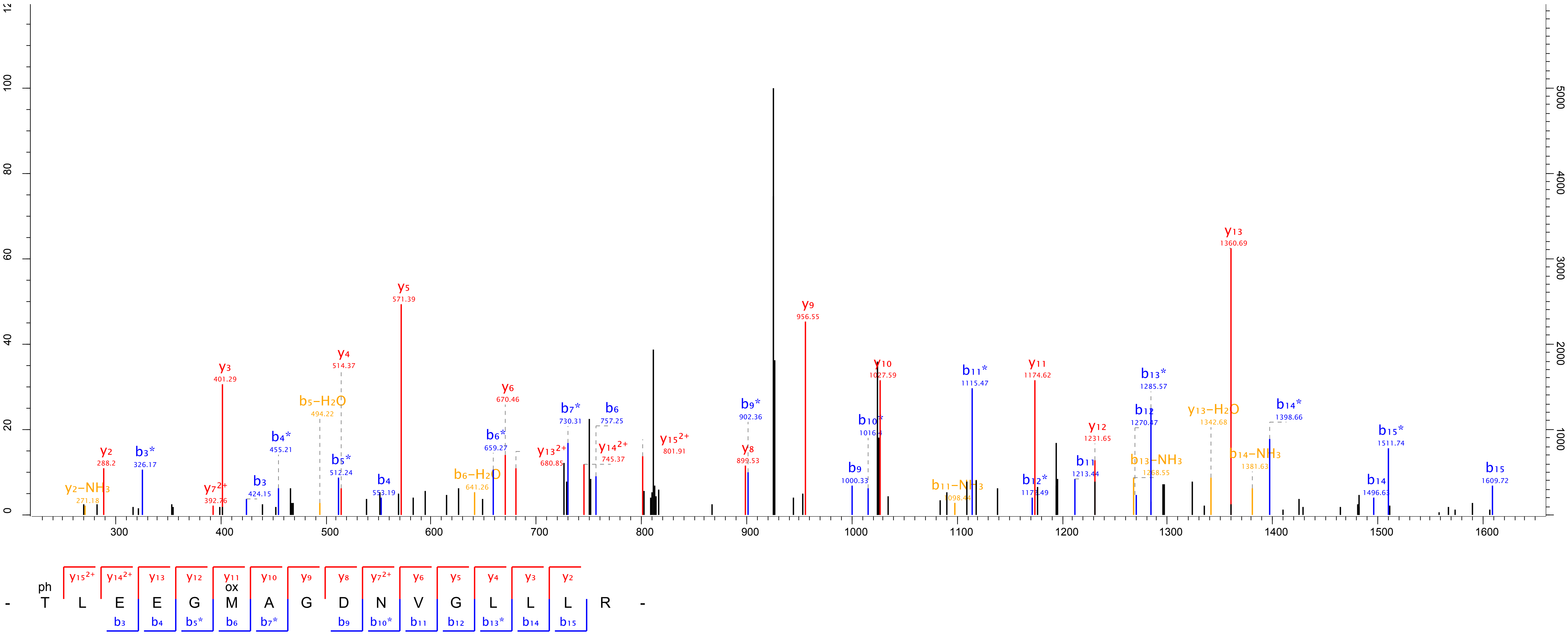

Raw file	Scan	Method	Score	m/z
20130708_PS_Phosphotest_6803wt_SDS01	10952	ITMS; CID	96.06	611.82

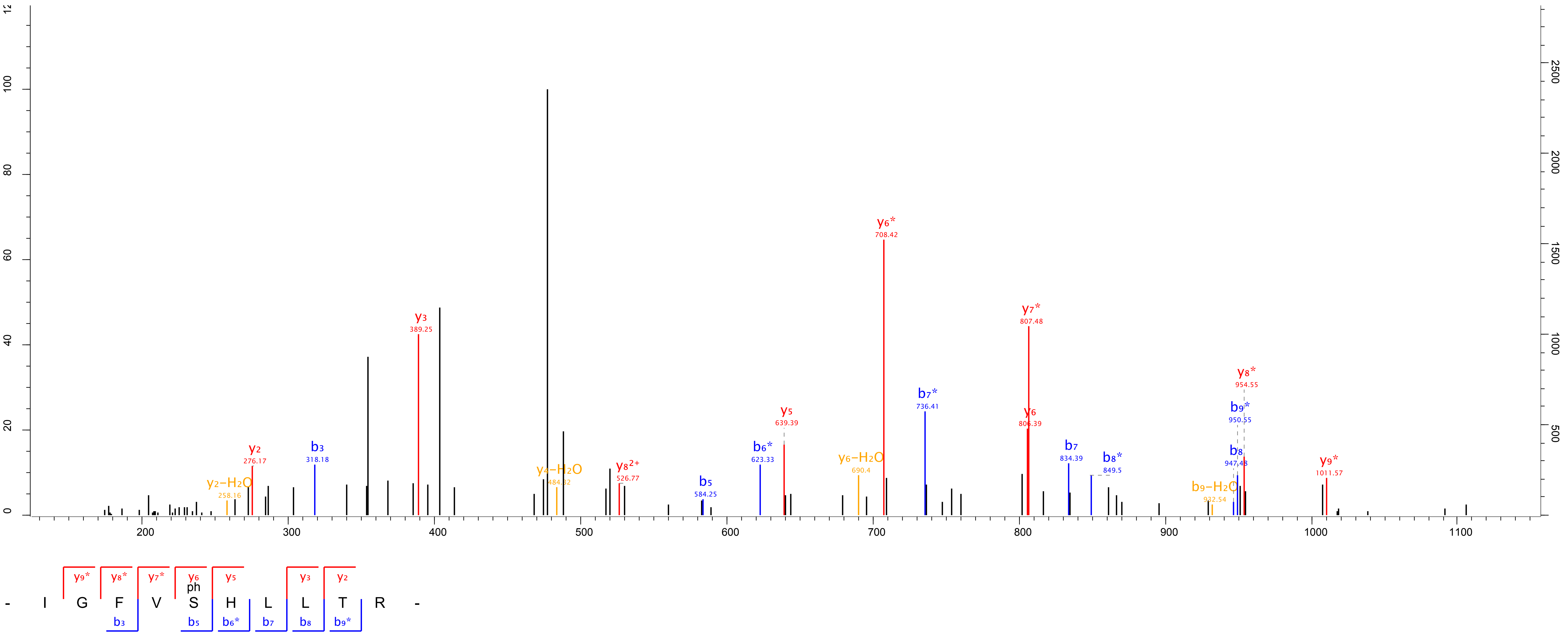

Raw file	Scan	Method	Score	m/z
20130708_PS_Phosphotest_6803wt_SDS01	10961	ITMS; CID	142.23	814.89

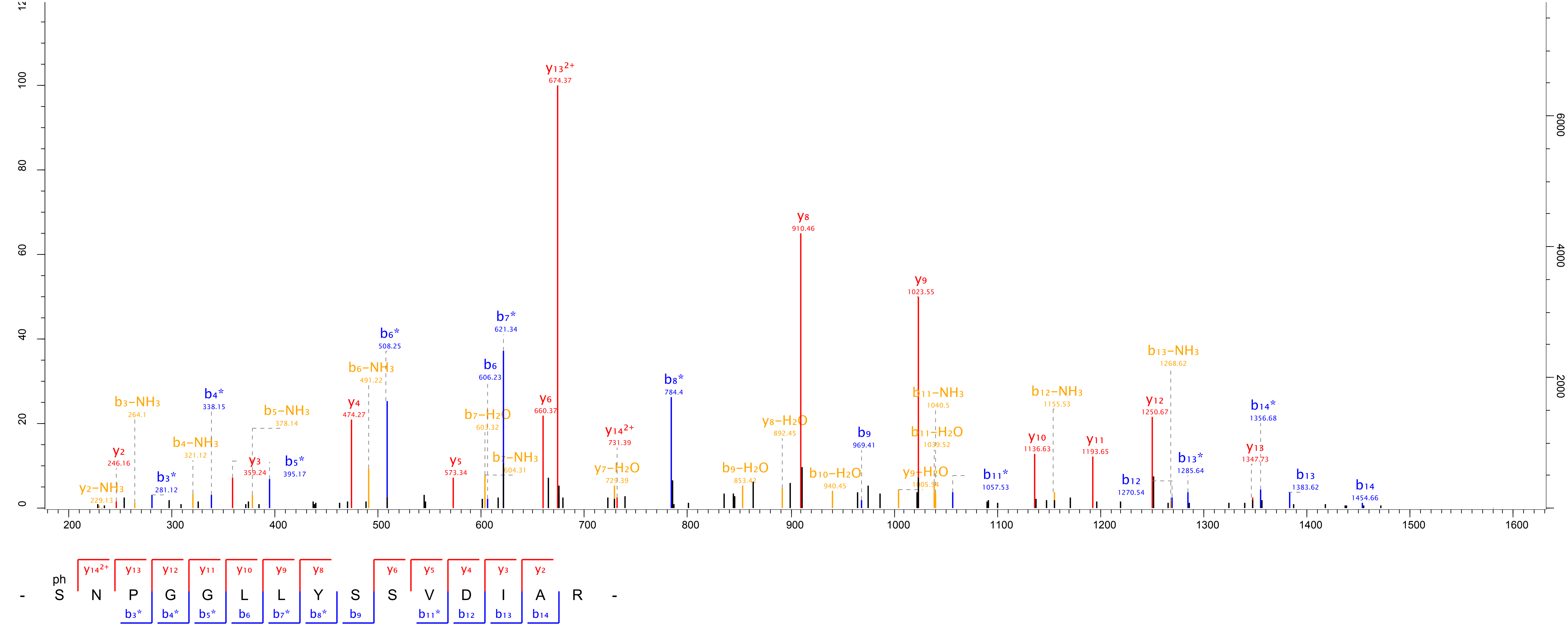

Raw file	Scan	Method	Score	m/z
20130708_PS_Phosphotest_6803wt_SDS01	11156	ITMS; CID	132.51	710.85

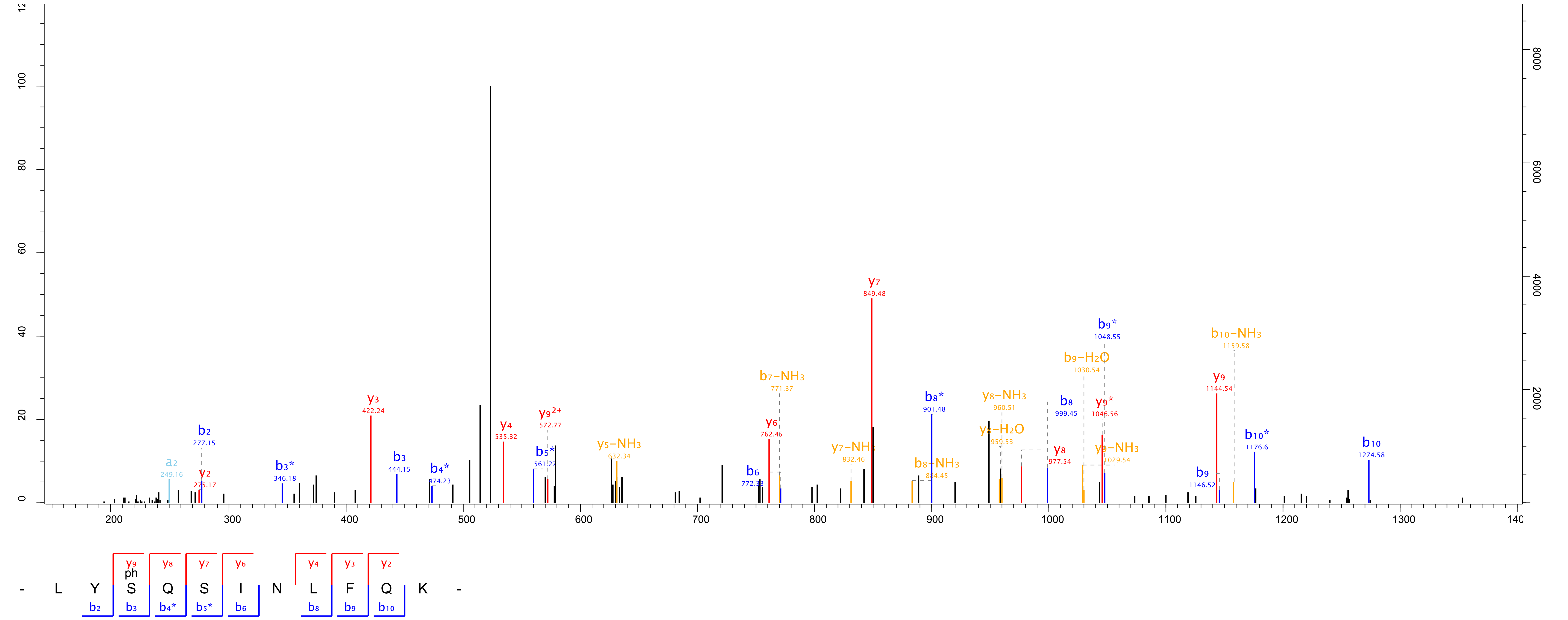

Raw file	Scan	Method	Score	m/z
20130708_PS_Phosphotest_6803wt_SDS01	11385	ITMS; CID	163.41	914.89

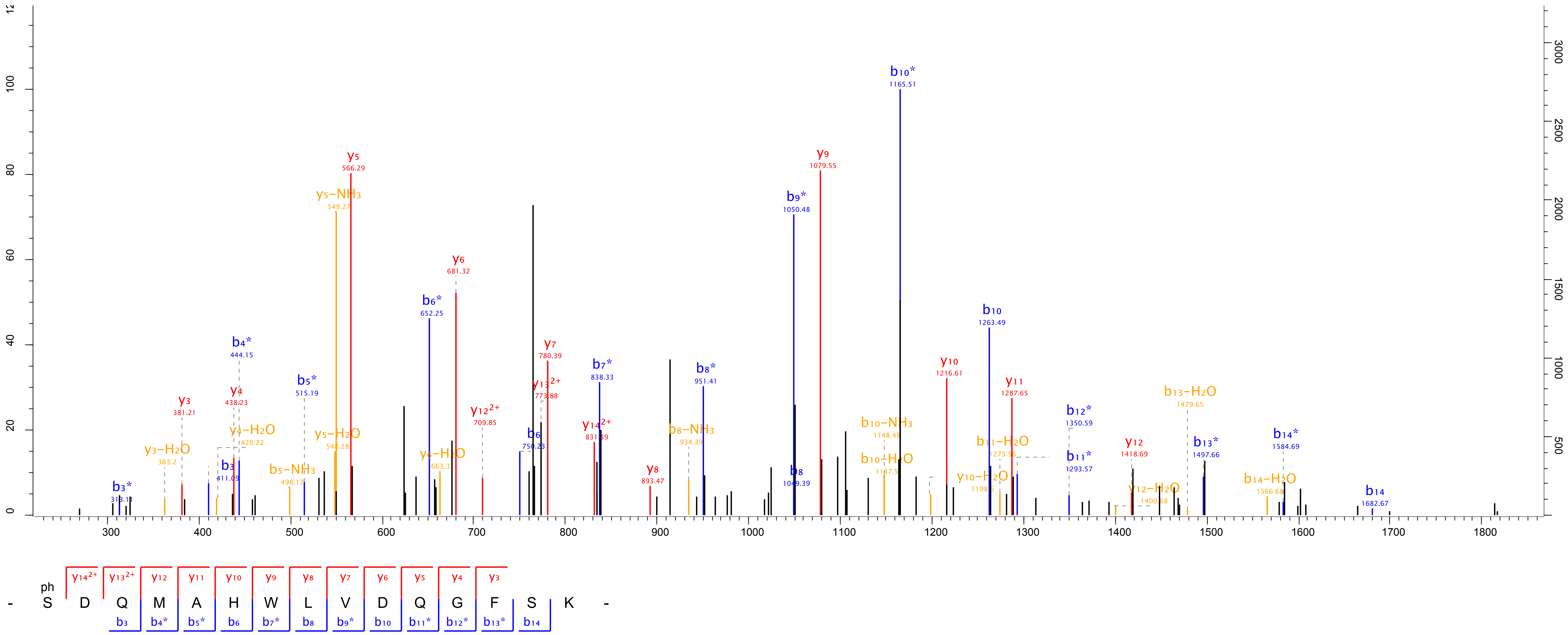

Raw file	Scan	Method	Score	m/z
20130708_PS_Phosphotest_6803wt_SDS01	11435	ITMS; CID	87.72	946.45

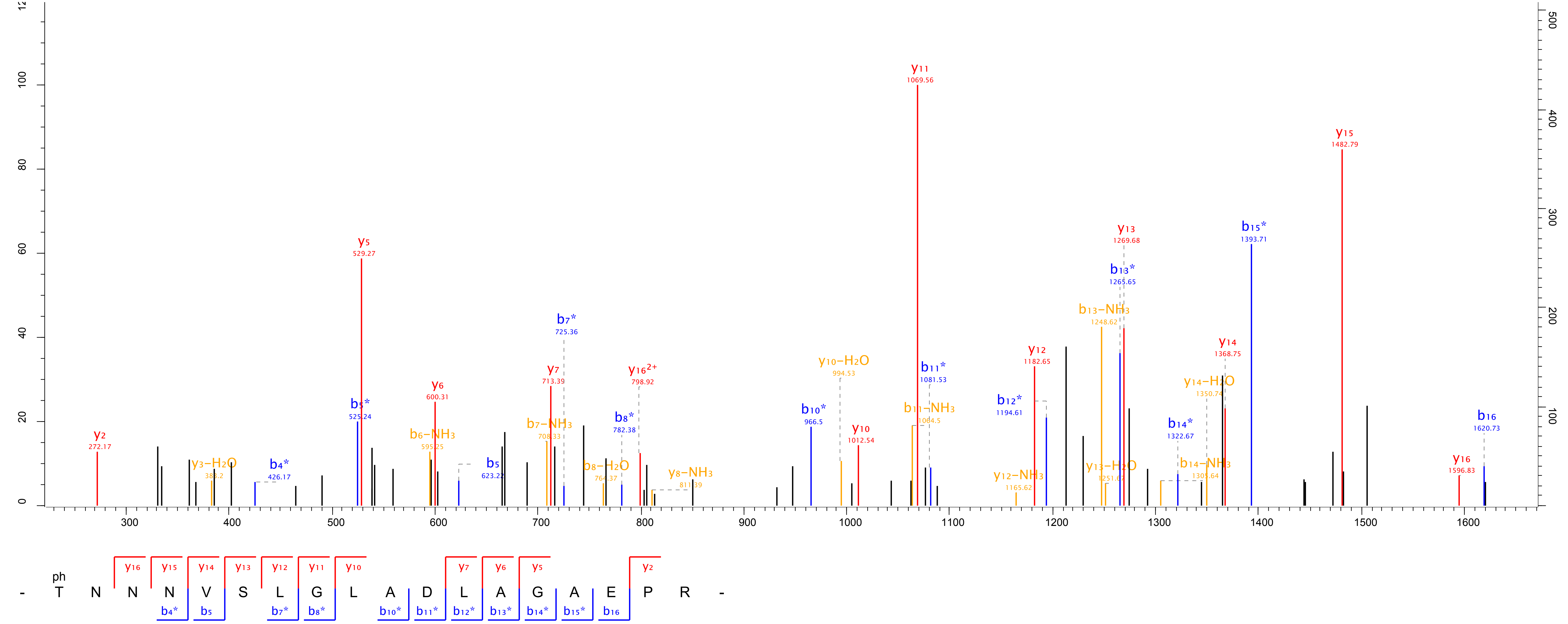

Raw file	Scan	Method	Score	m/z
20130708_PS_Phosphotest_6803wt_SDS01	11443	ITMS; CID	84.17	609.31

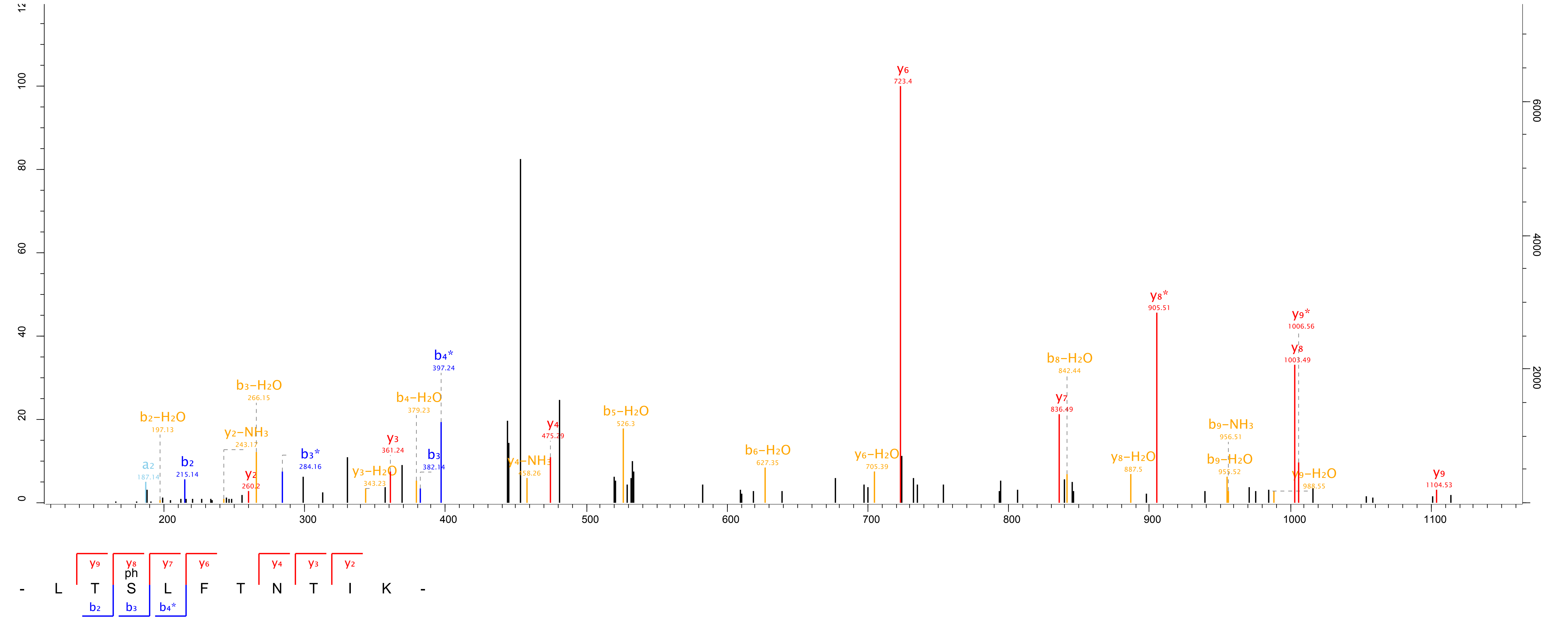

Raw file	Scan	Method	Score	m/z
20130708_PS_Phosphotest_6803wt_SDS01	11462	ITMS; CID	114.76	606.29

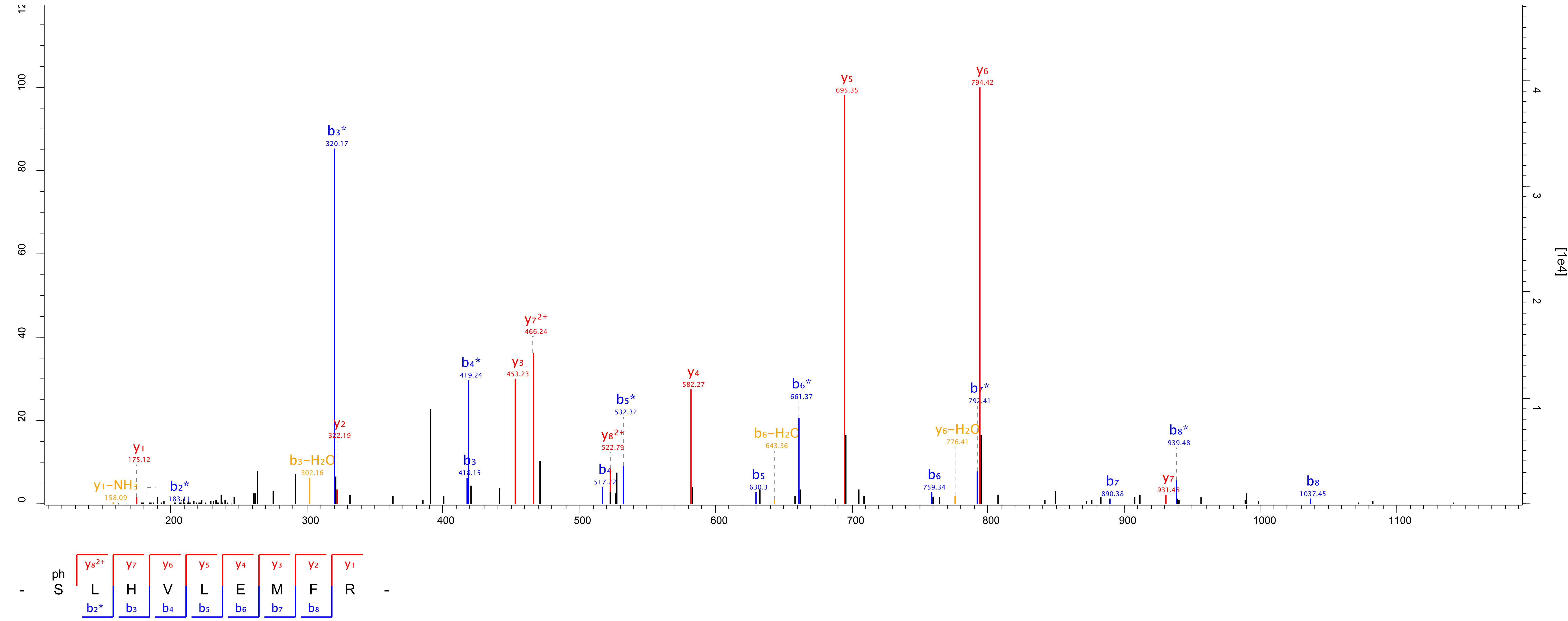

Raw file	Scan	Method	Score	m/z
20130708_PS_Phosphotest_6803wt_SDS01	11487	ITMS; CID	179.59	583.81

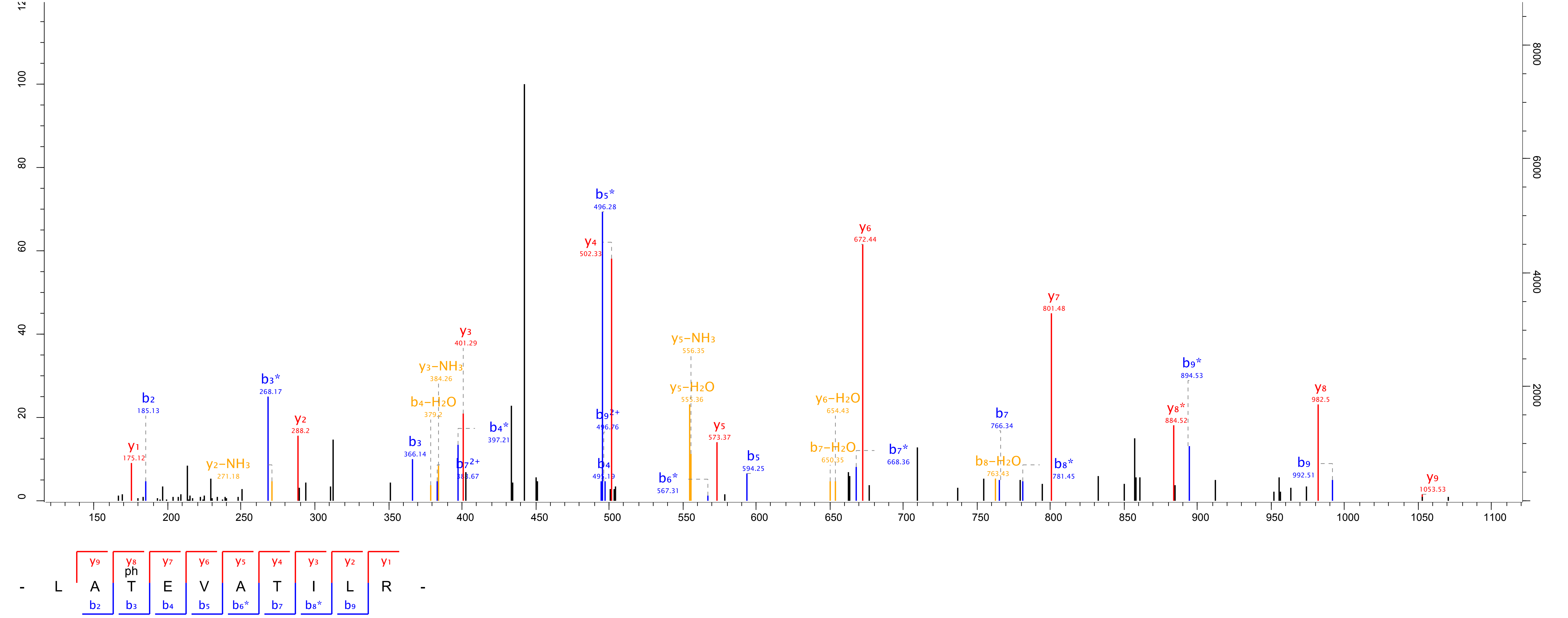

Raw file	Scan	Method	Score	m/z
20130708_PS_Phosphotest_6803wt_SDS01	11801	ITMS; CID	237.06	754.38

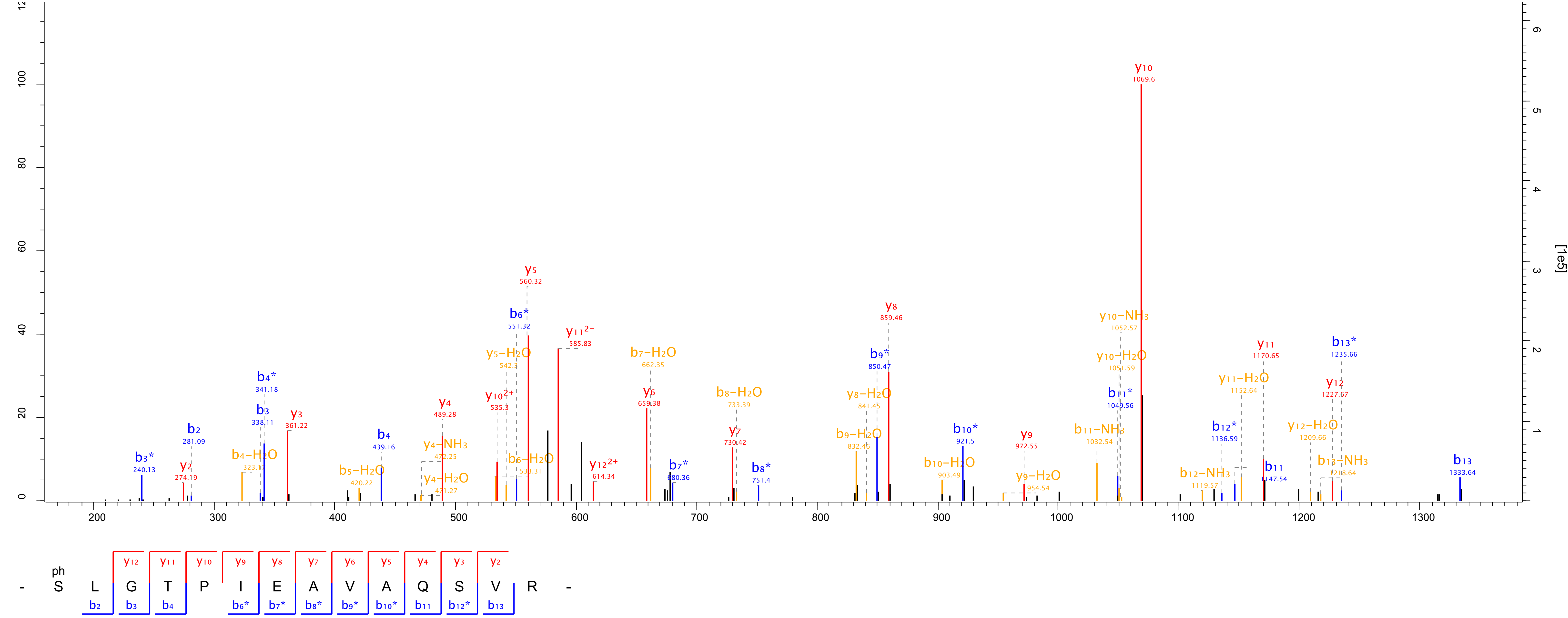

Raw file	Scan	Method	Score	m/z
20130708_PS_Phosphotest_6803wt_SDS01	11816	ITMS; CID	99.16	688.27

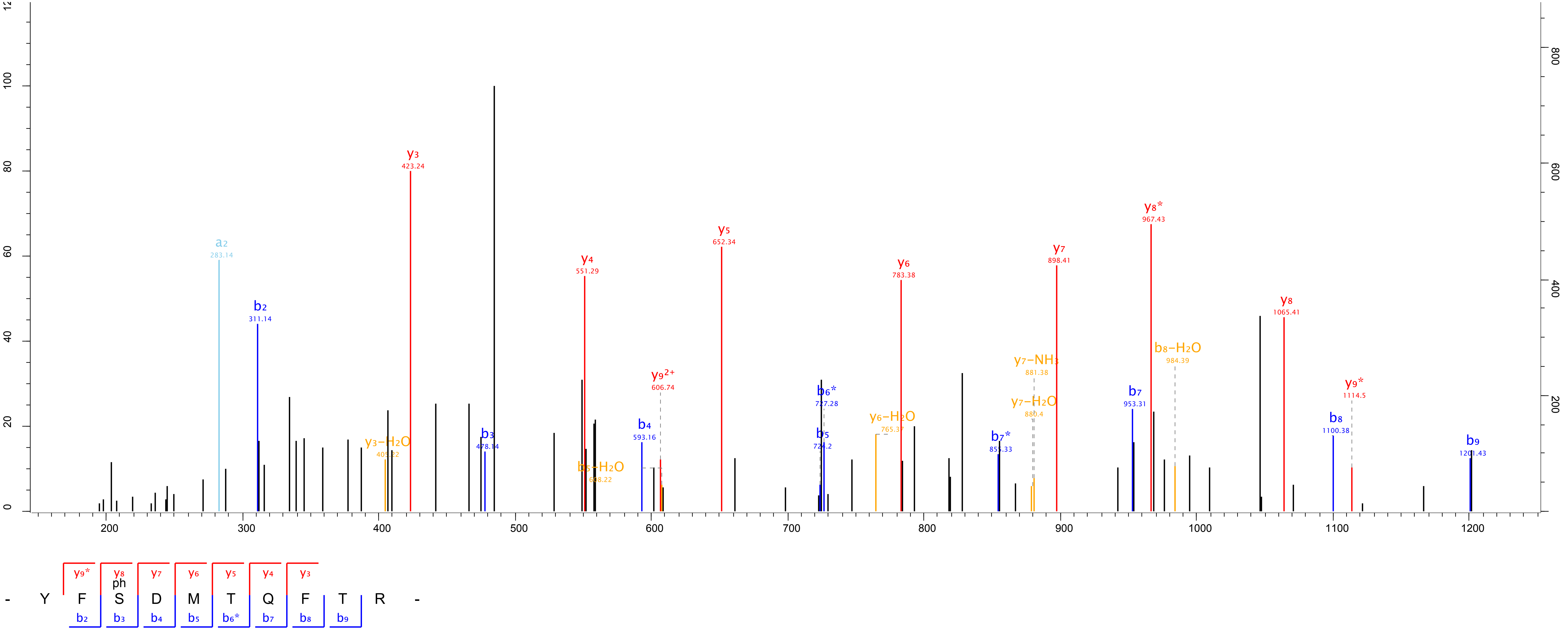

Raw file	Scan	Method	Score	m/z
20130708_PS_Phosphotest_6803wt_SDS01	11899	ITMS; CID	222.3	895.89

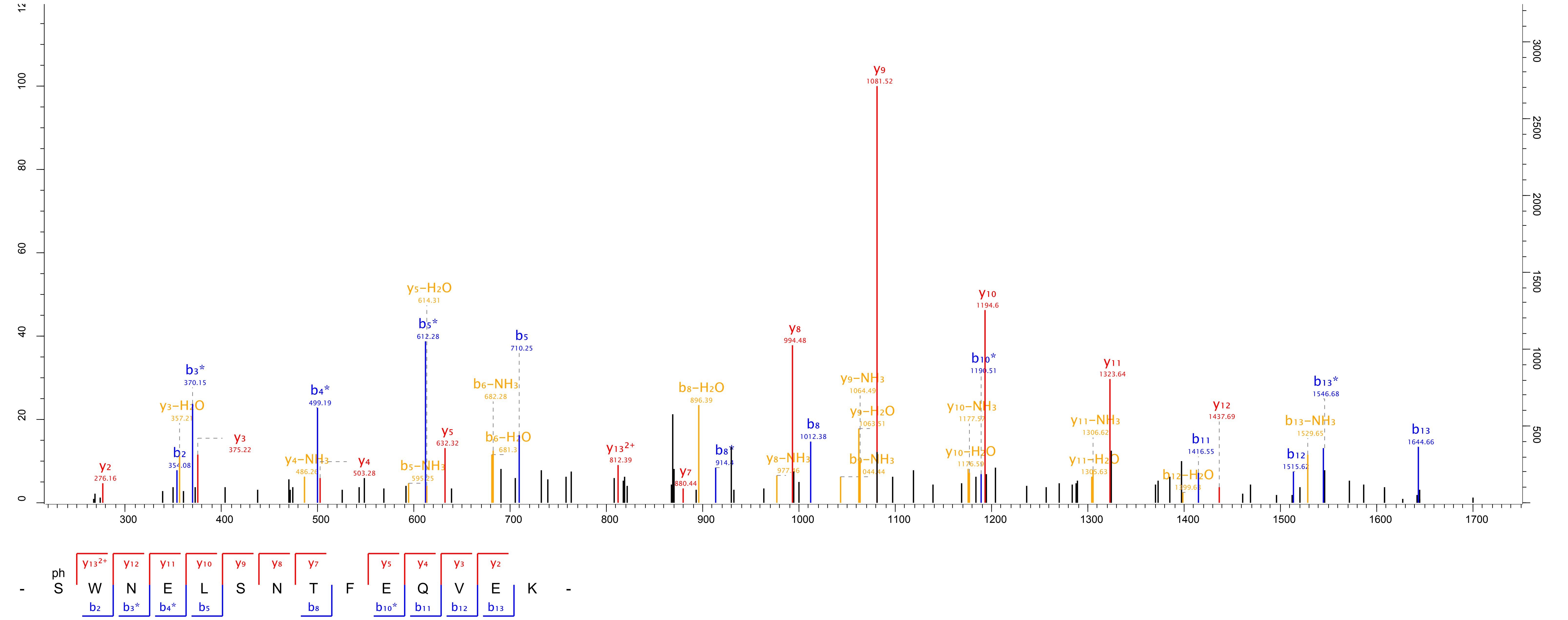

Raw file	Scan	Method	Score	m/z
20130708_PS_Phosphotest_6803wt_SDS01	12029	ITMS; CID	130.09	870.91

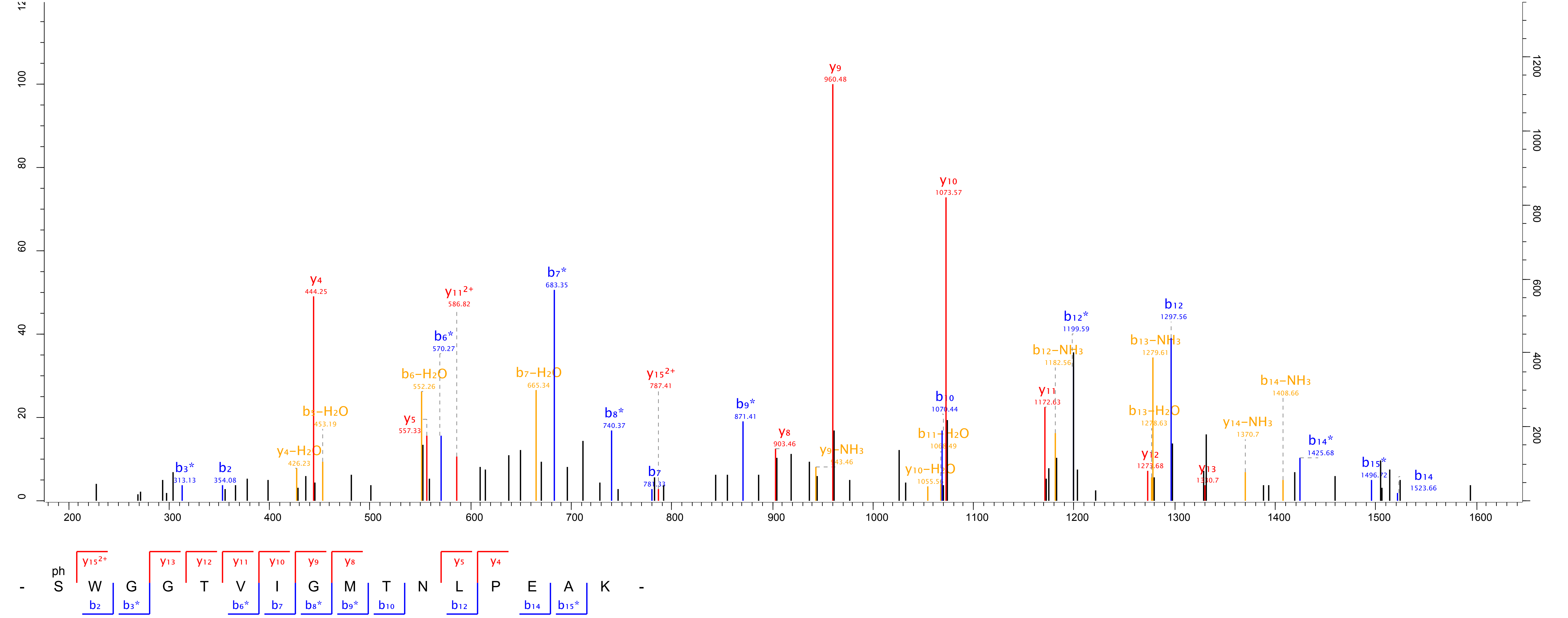

Raw file	Scan	Method	Score	m/z
20130708_PS_Phosphotest_6803wt_SDS01	12032	ITMS; CID	152.07	820.39

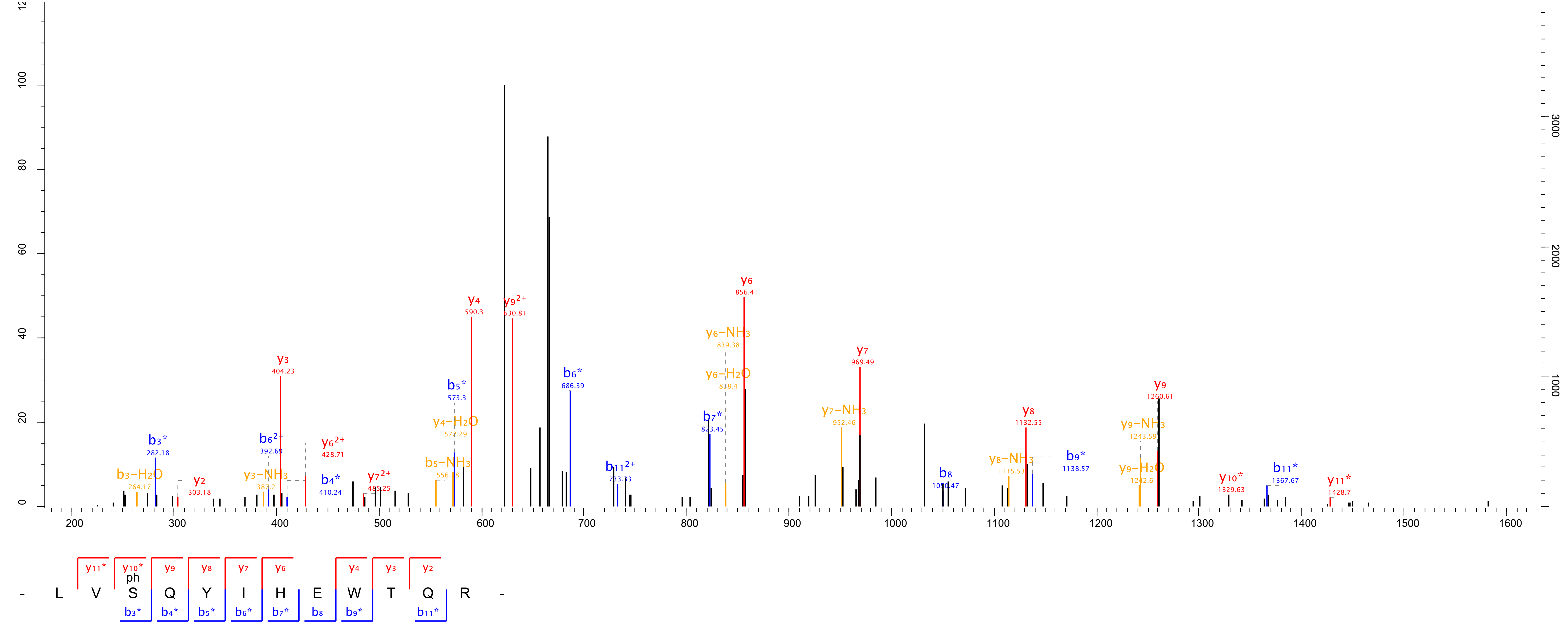

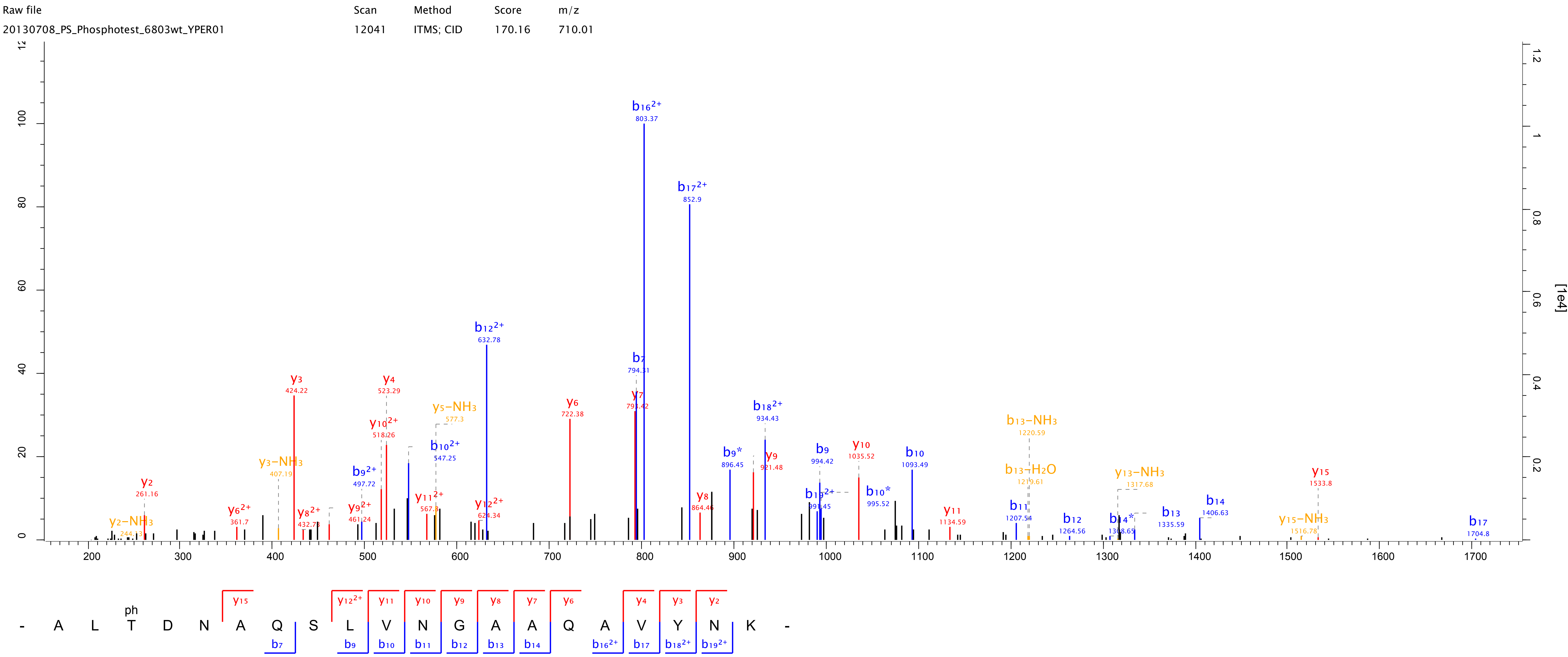

Raw file
20130708_PS_Phosphotest_6803wt_YPER01
Scan
12041
Method
ITMS; CID
Score
170.16
m/z
710.01
[1e4]
b16 2+ 803.37
b17 2+ 852.9
b12 2+ 632.78
b7 794.31
y7 793.42
y3 424.22
y4 523.29
y6 722.38
y5−NH3 577.3
y10 2+ 518.26
b10 2+ 547.25
b18 2+ 934.43
b13−NH3 1220.59
b13−H2O 1219.61
y10 1035.52
b10 1093.49
b9 994.42
b9* 896.45
y9 921.48
b9 2+ 497.72
y3−NH3 407.19
y2 261.16
y6 2+ 361.7
y8 2+ 432.78
y9 2+ 461.24
y11 2+ 567.3
y12 2+ 624.34
y8 864.46
b19 2+ 991.45
b10* 995.52
y11 1134.59
b11 1207.54
b12 1264.56
b14* 1308.65
b13 1335.59
b14 1406.63
y13−NH3 1317.68
y15 1533.8
y15−NH3 1516.78
b17 1704.8
y2−NH3
- A L T D N A Q S L V N G A A Q A V Y N K -
ph

Raw file	Scan	Method	Score	m/z
20130708_PS_Phosphotest_6803wt_YPER01	12044	ITMS; CID	140.2	1065.01

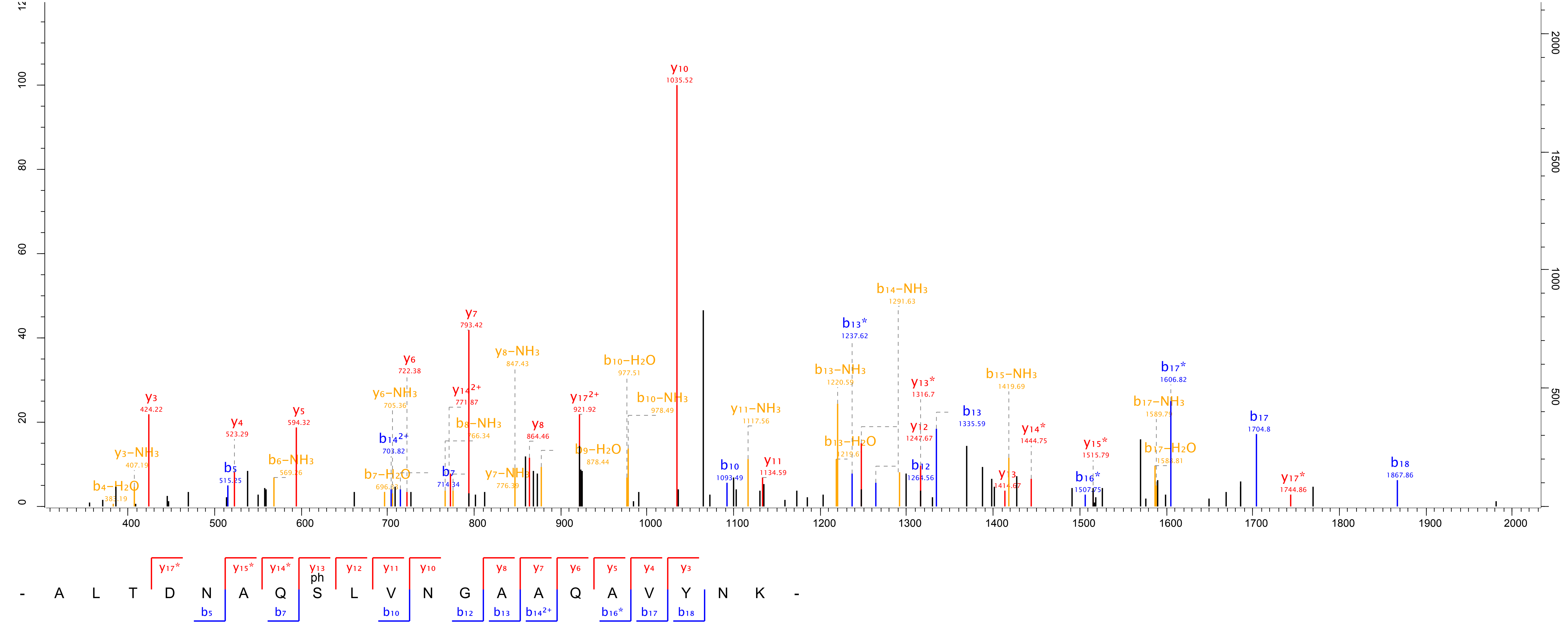

Raw file	Scan	Method	Score	m/z
20130708_PS_Phosphotest_6803wt_YPER01	12047	ITMS; CID	138.95	1096.01

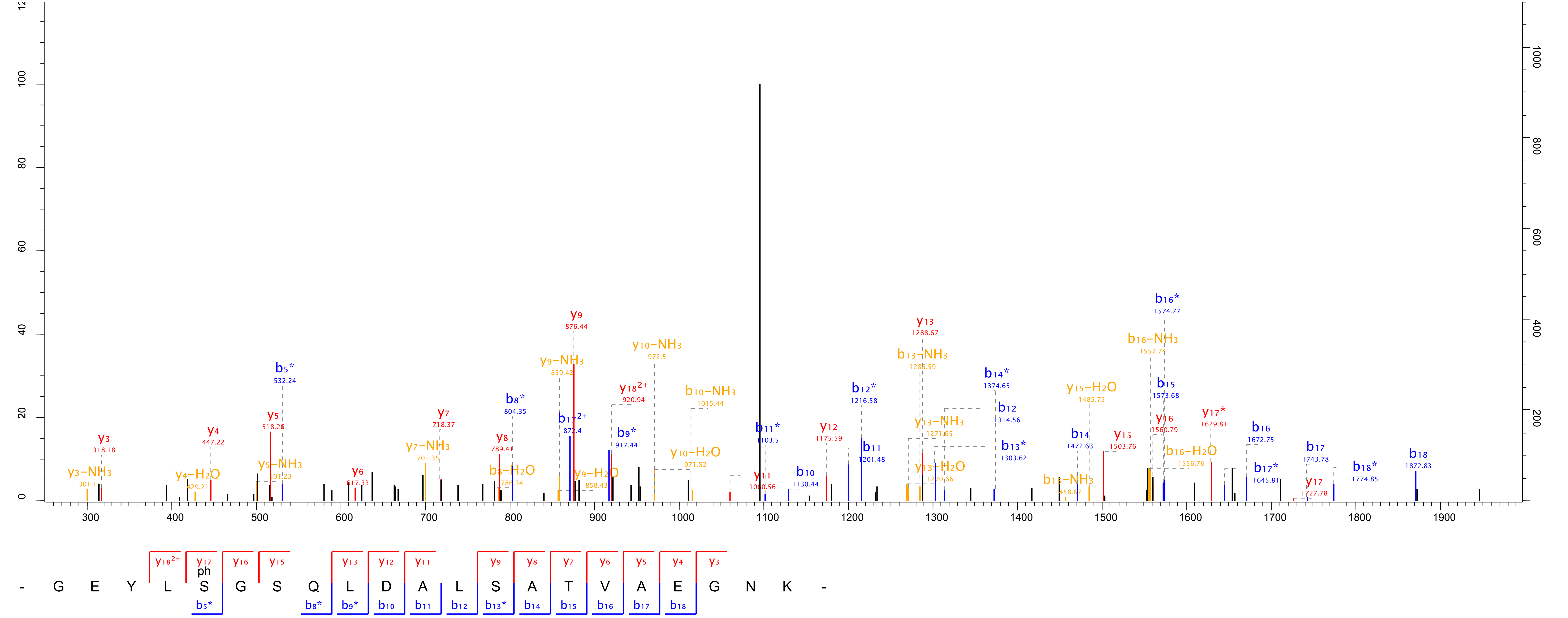

Raw file	Scan	Method	Score	m/z
20130708_PS_Phosphotest_6803wt_YPER03	12317	ITMS; CID	163.75	1095.5

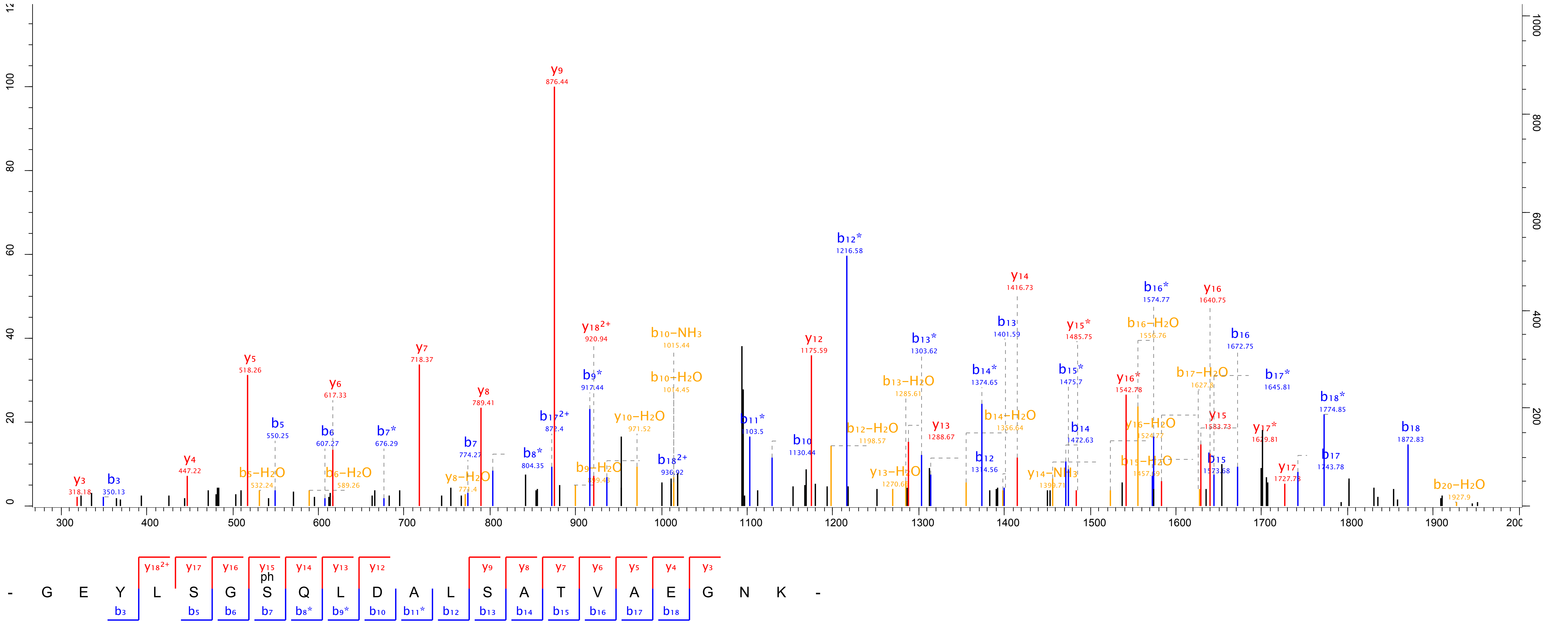

Raw file	Scan	Method	Score	m/z
20130708_PS_Phosphotest_6803wt_SDS03	12404	ITMS; CID	90.48	605.63

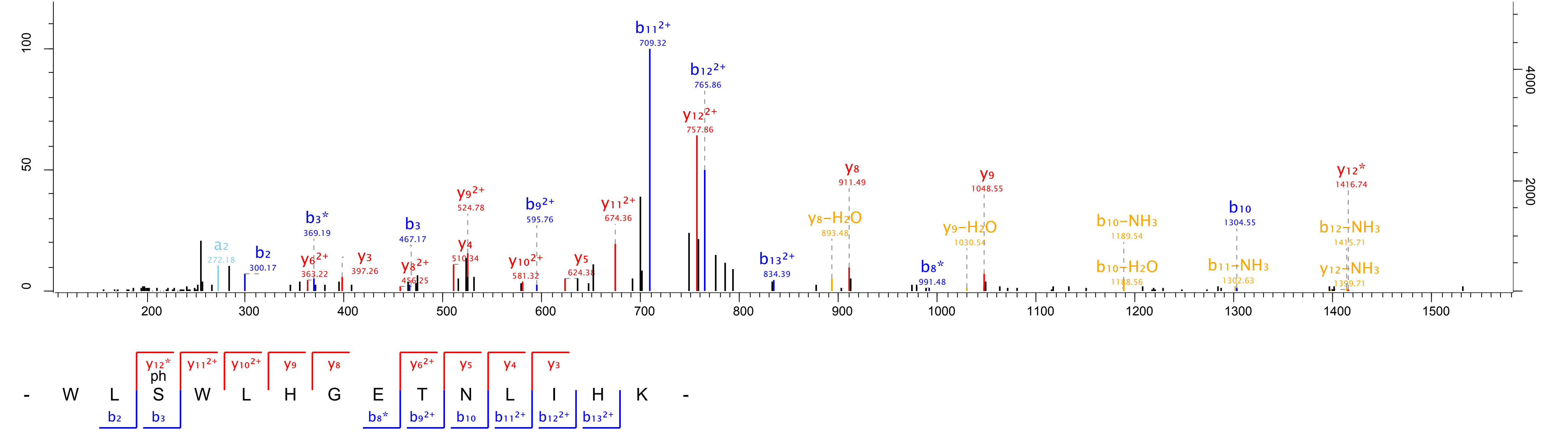

Raw file	Scan	Method	Score	m/z
20130708_PS_Phosphotest_6803wt_SDS01	12413	ITMS; CID	205.66	750.35

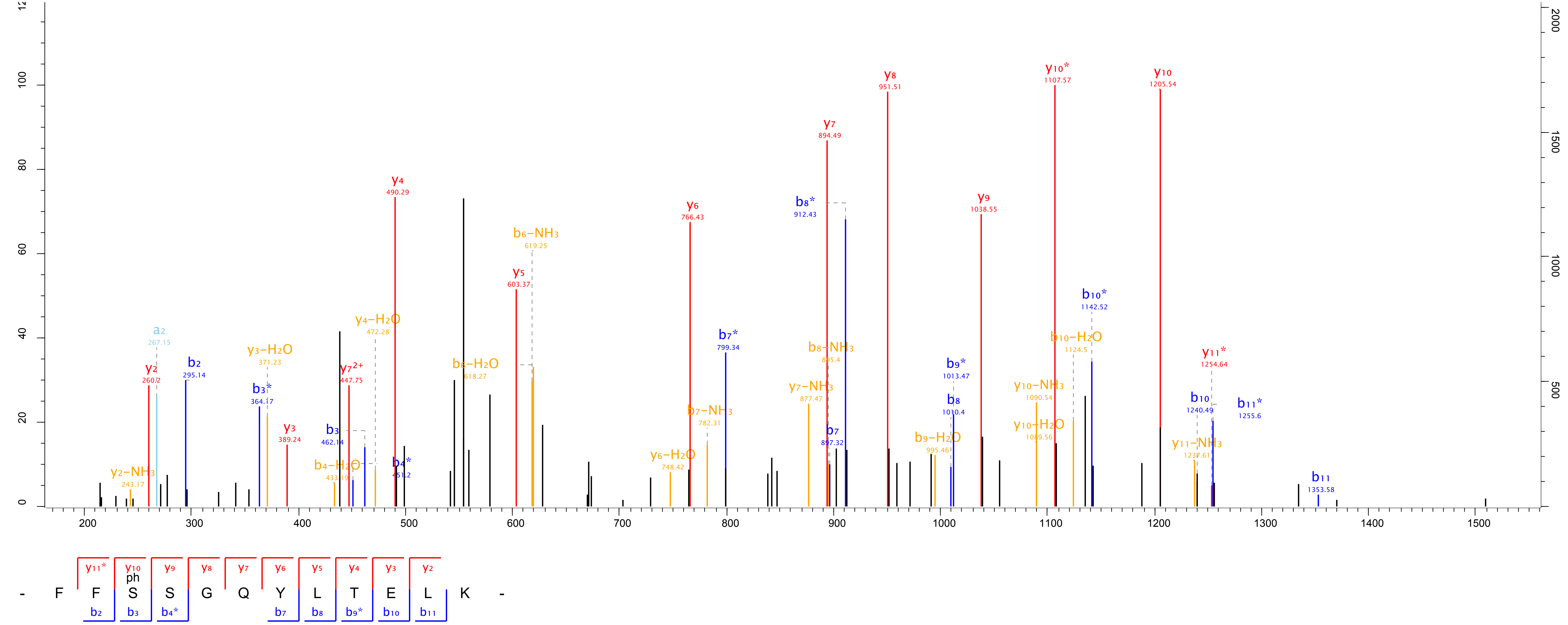

Raw file	Scan	Method	Score	m/z
20130708_PS_Phosphotest_6803wt_SDS01	12573	ITMS; CID	107.53	652.32

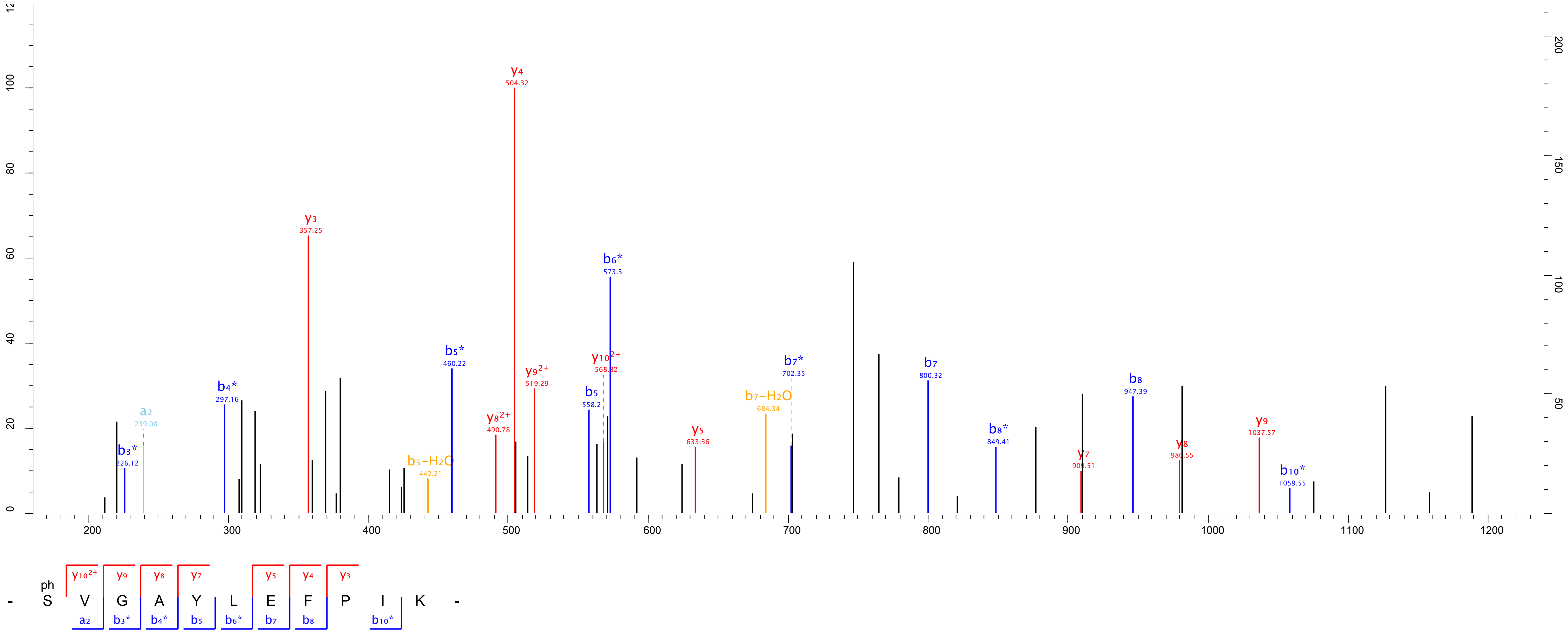

Raw file	Scan	Method	Score	m/z
20130708_PS_Phosphotest_6803wt_SDS01	12741	ITMS; CID	169.8	1132.53

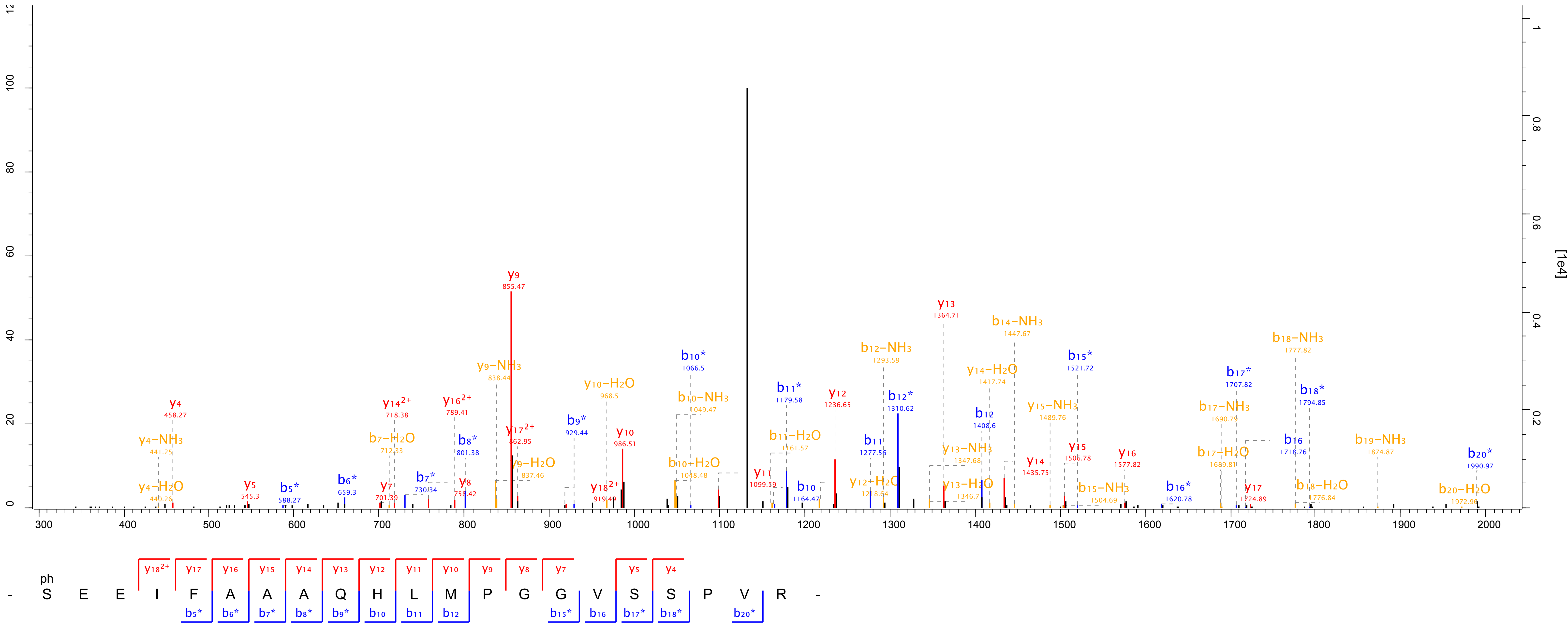

Raw file	Scan	Method	Score	m/z
20130708_PS_Phosphotest_6803wt_SDS01	12753	ITMS; CID	124.73	882.95

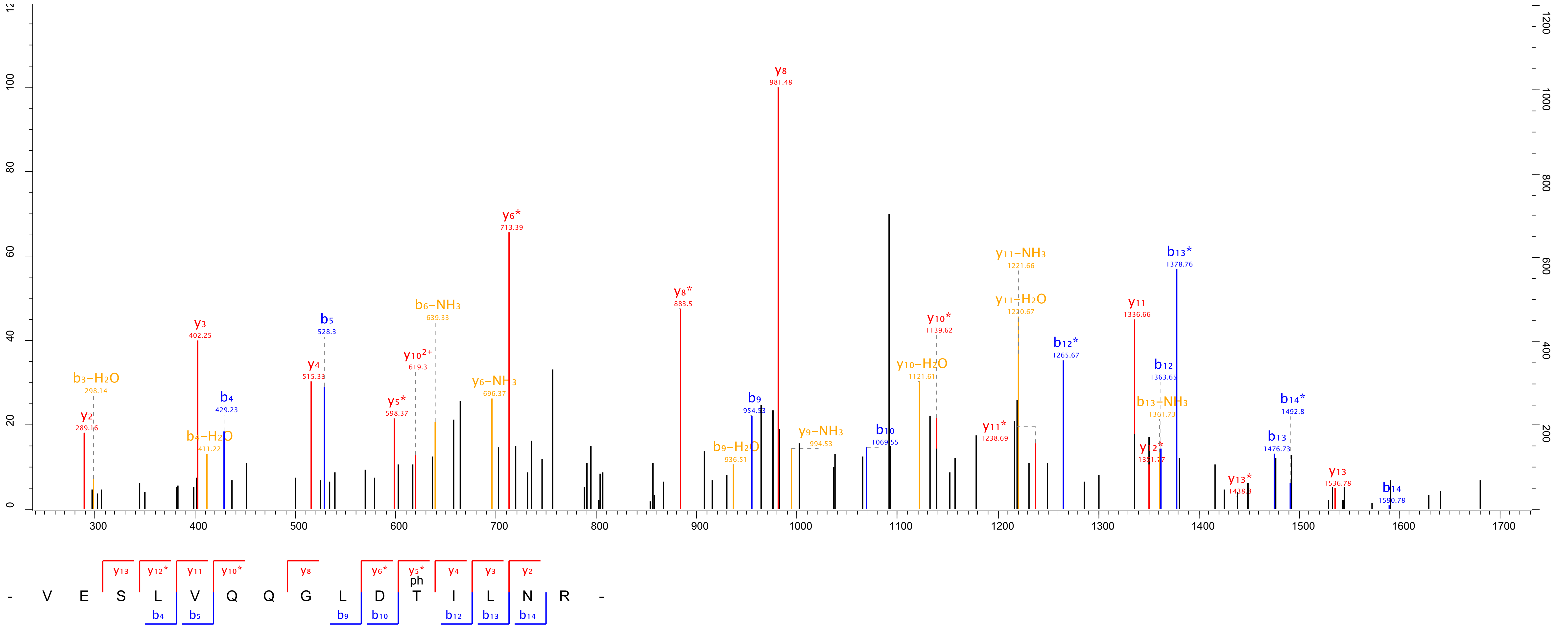

Raw file	Scan	Method	Score	m/z
20130708_PS_Phosphotest_6803wt_SDS01	12809	ITMS; CID	127.32	684.86

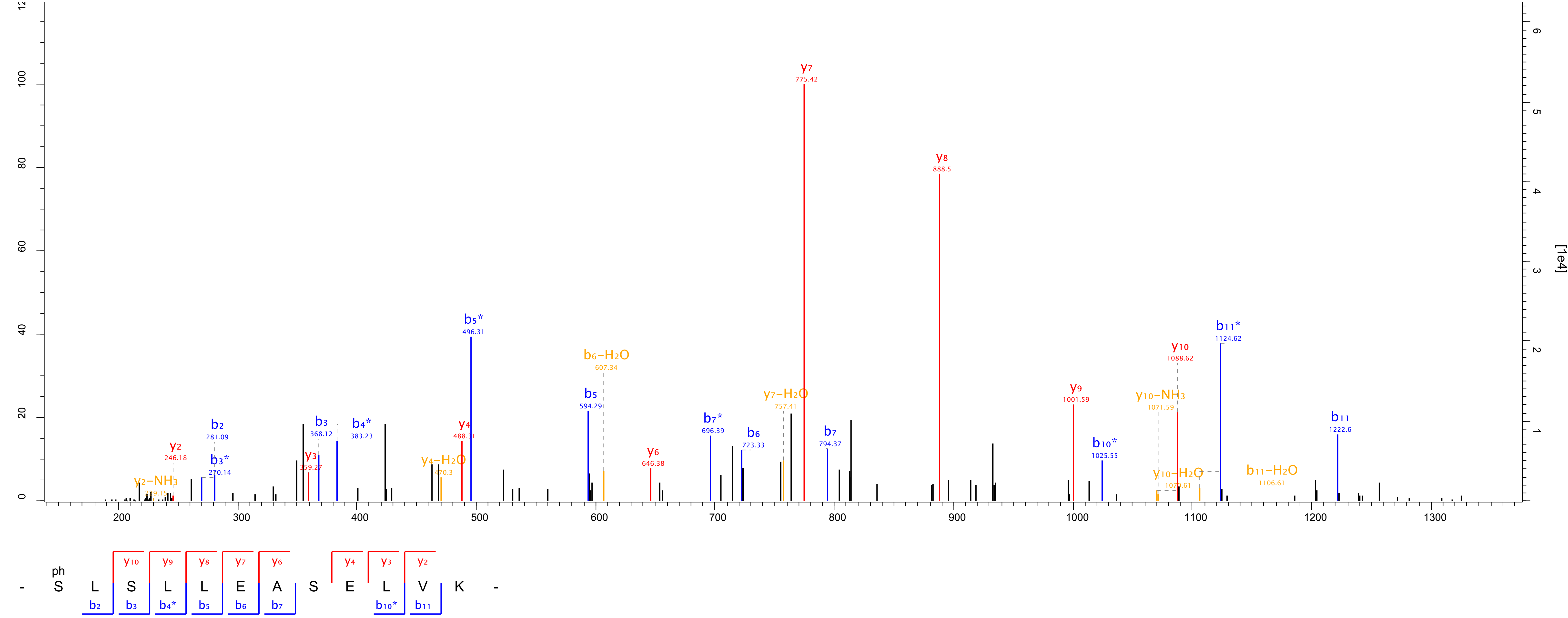

Raw file	Scan	Method	Score	m/z
20130708_PS_Phosphotest_6803wt_SDS01	12815	ITMS; CID	117.84	781.4

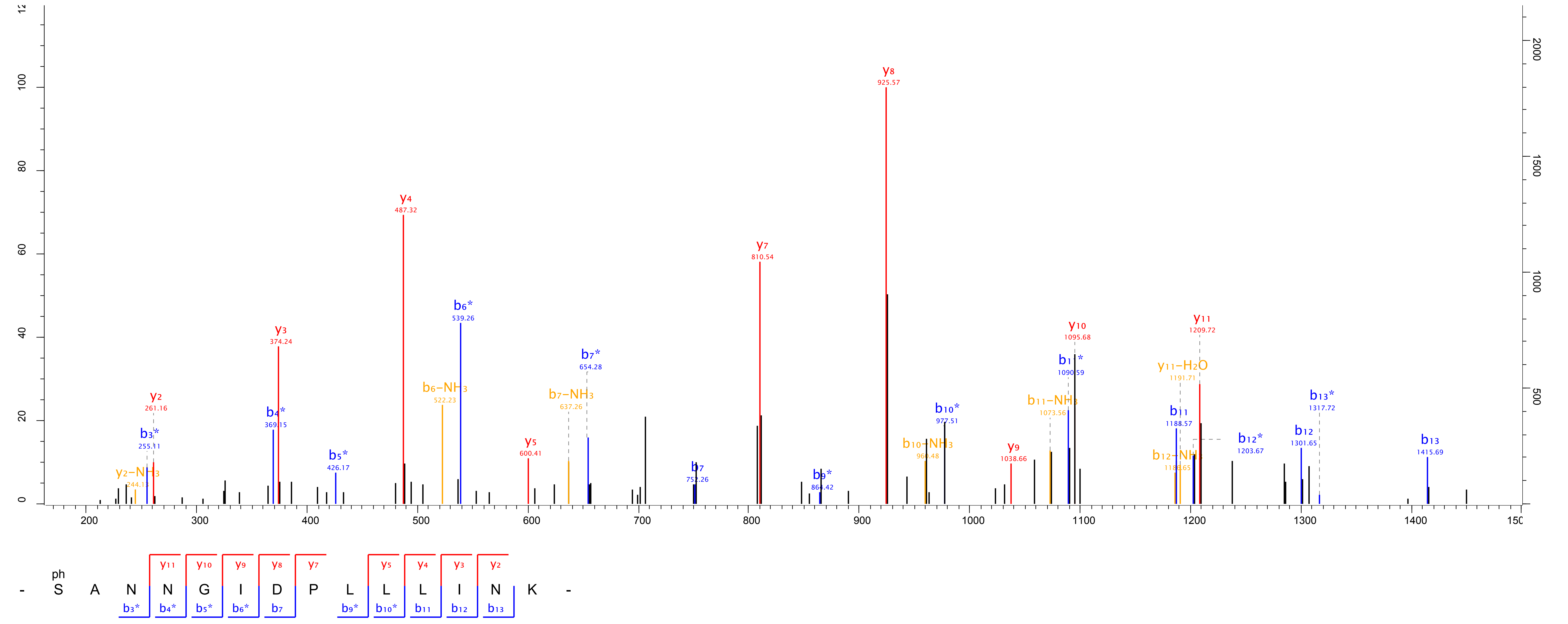

Raw file	Scan	Method	Score	m/z
20130708_PS_Phosphotest_6803wt_SDS01	12830	ITMS; CID	76.76	906.11

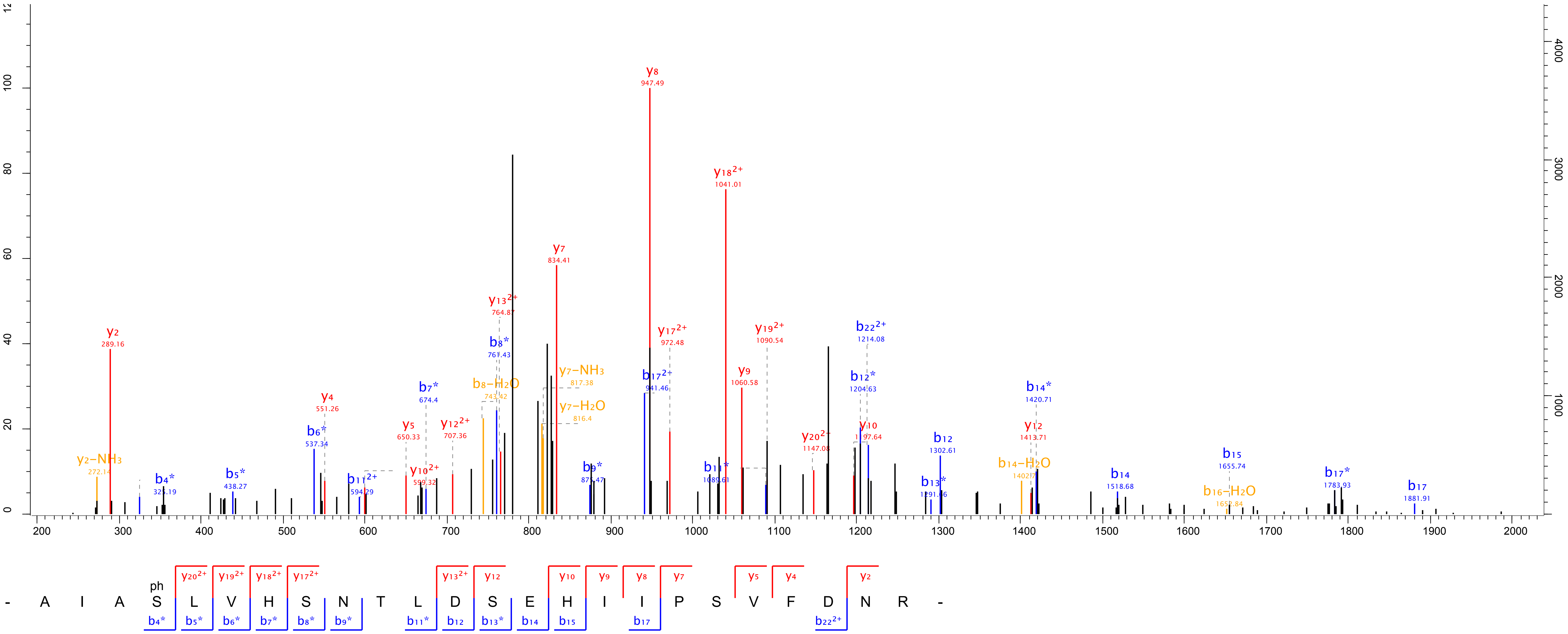

Raw file	Scan	Method	Score	m/z
20130708_PS_Phosphotest_6803wt_SDS01	12864	ITMS; CID	172.42	734.36

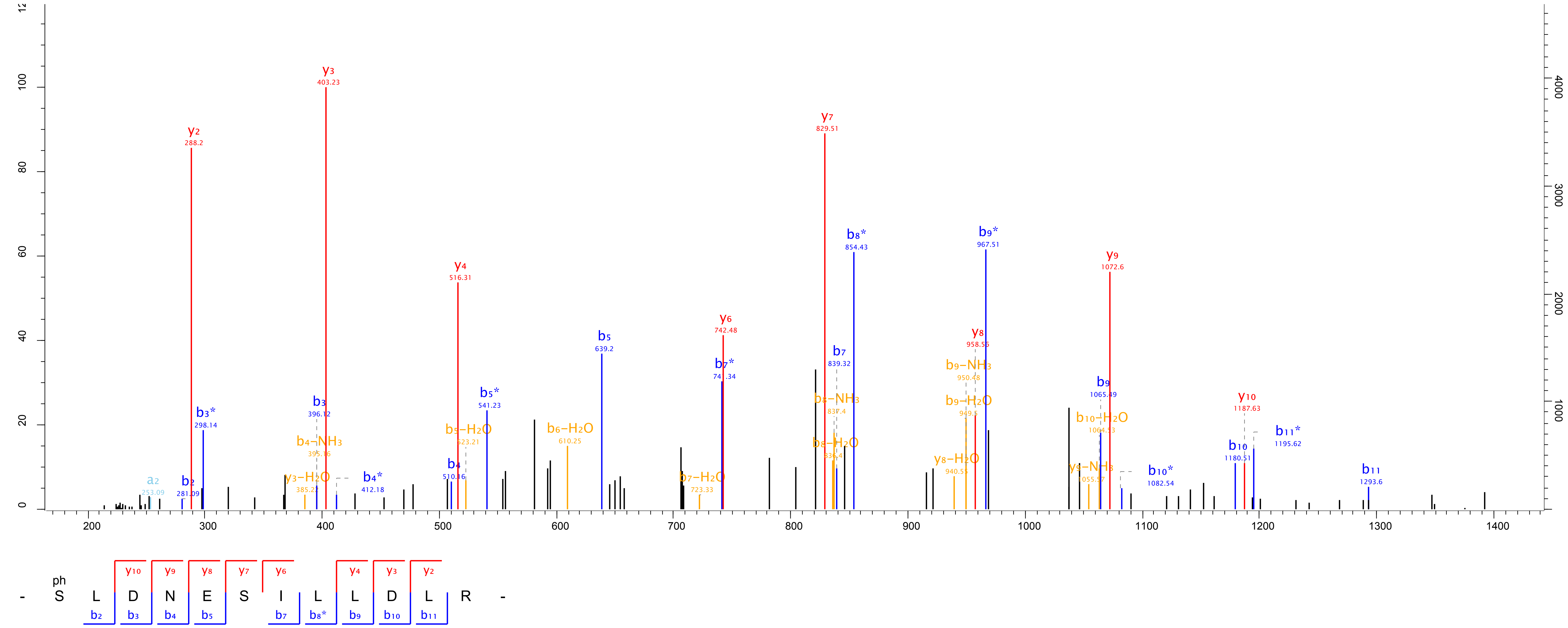

Raw file	Scan	Method	Score	m/z
20130708_PS_Phosphotest_6803wt_SDS01	12920	ITMS; CID	69.99	740.36

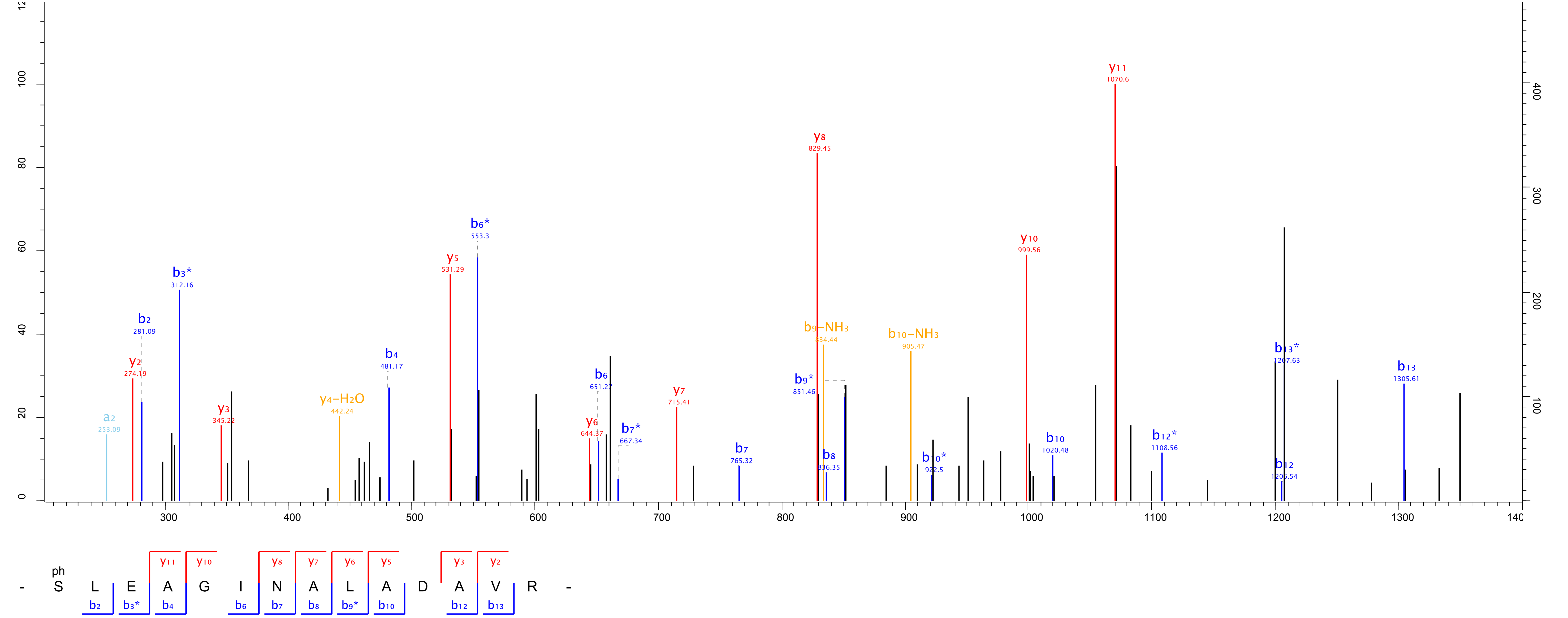

Raw file	Scan	Method	Score	m/z
20130708_PS_Phosphotest_6803wt_SDS01	12929	ITMS; CID	71.93	676.77

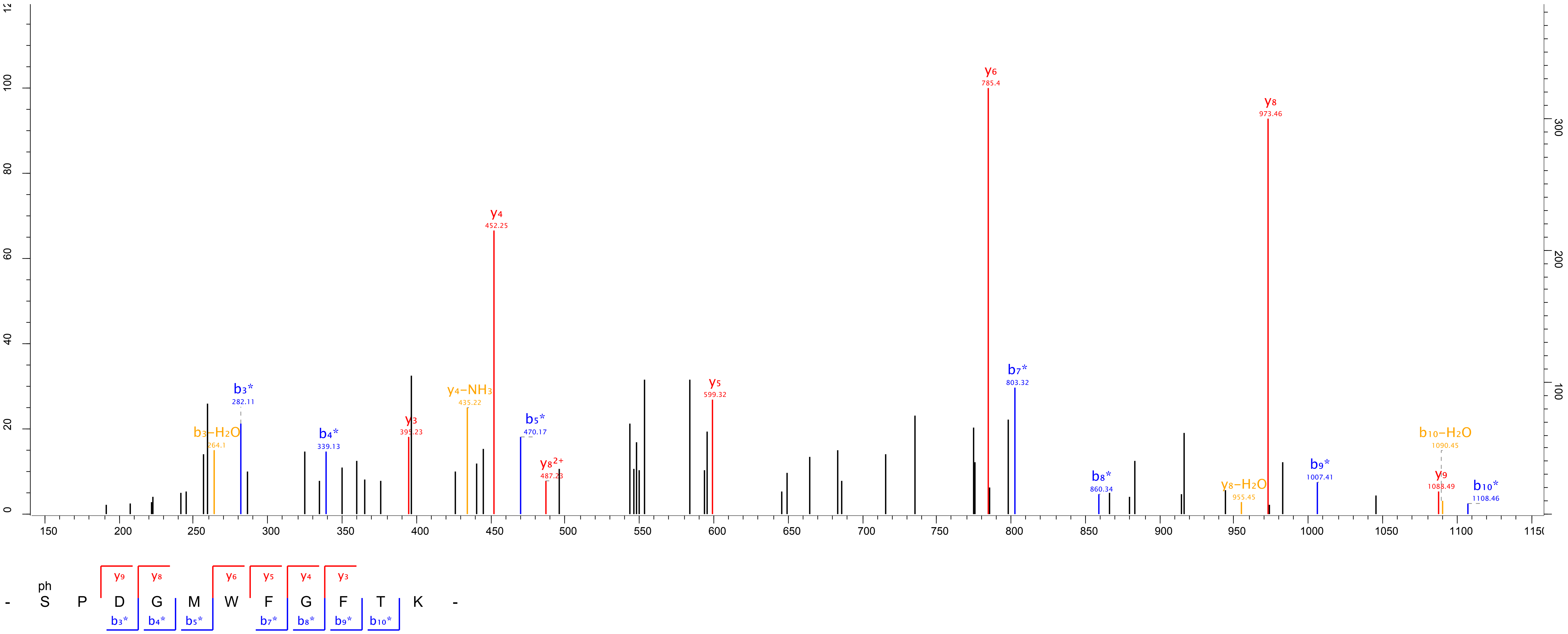

Raw file	Scan	Method	Score	m/z
20130708_PS_Phosphotest_6803wt_SDS01	12981	ITMS; CID	100.04	901.96

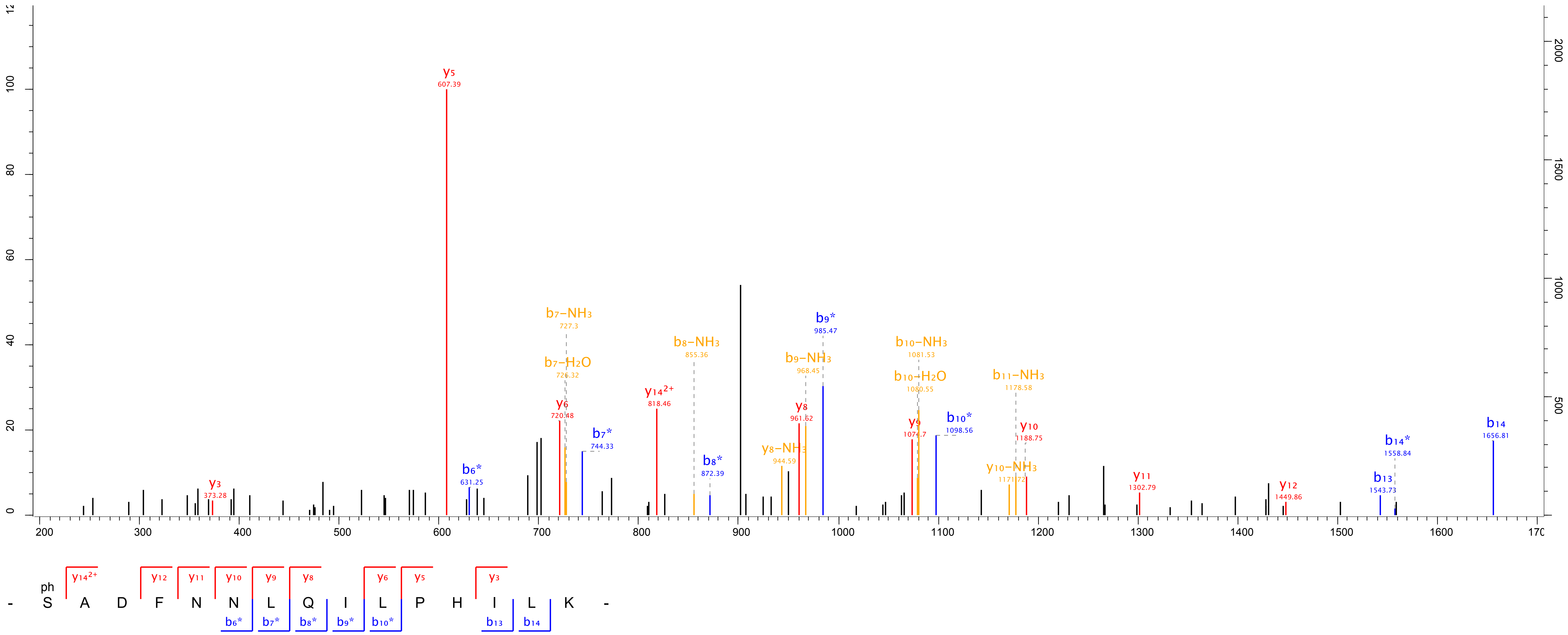

Raw file	Scan	Method	Score	m/z
20130708_PS_Phosphotest_6803wt_YPER01	13025	ITMS; CID	70.28	1111.18

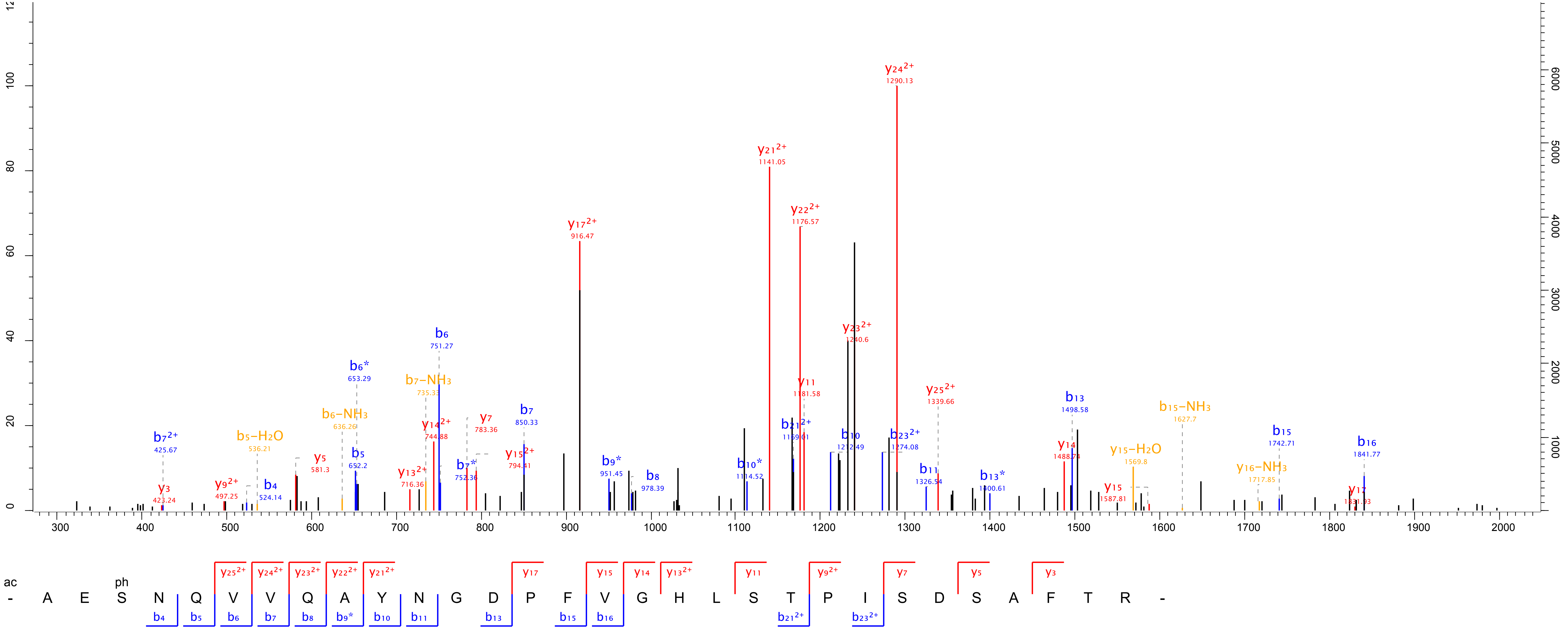

Raw file	Scan	Method	Score	m/z
20130708_PS_Phosphotest_6803wt_YPER03	13142	ITMS; CID	56.34	1252.1

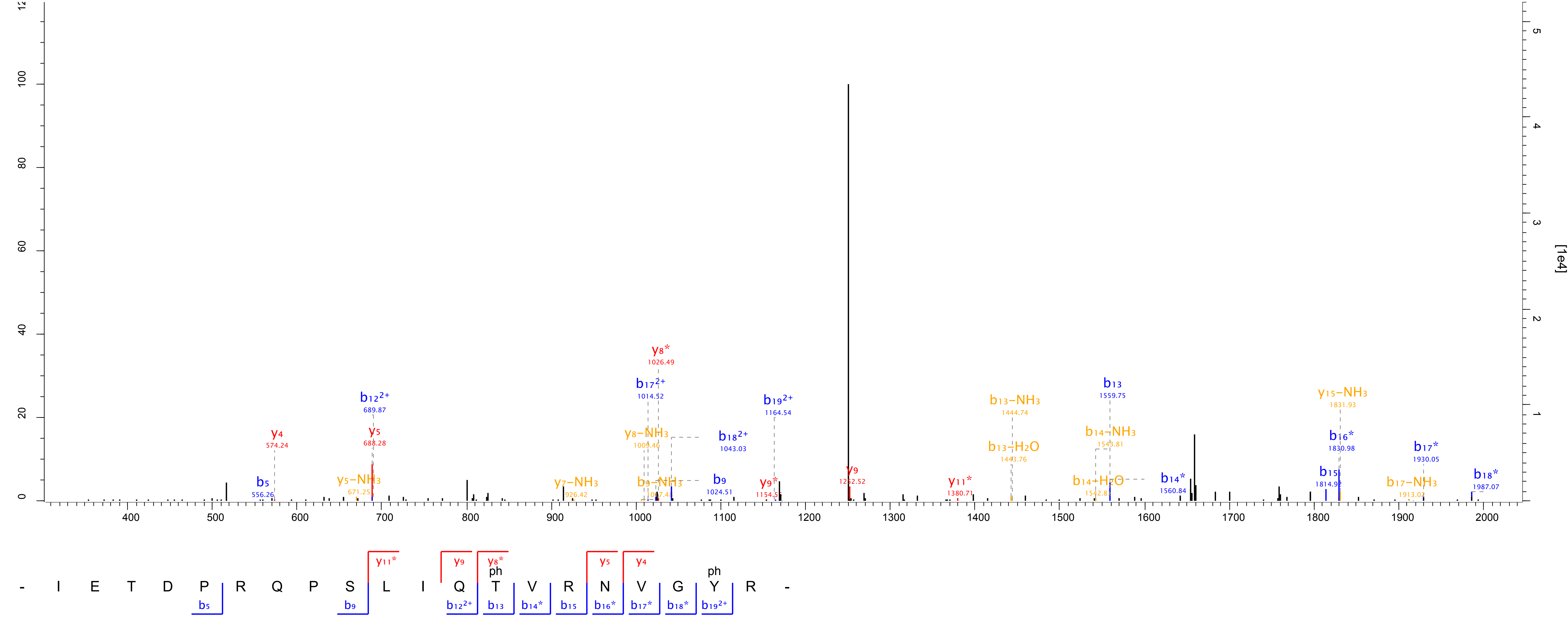

Raw file	Scan	Method	Score	m/z
20130708_PS_Phosphotest_6803wt_SDS01	13188	ITMS; CID	73.9	555.3

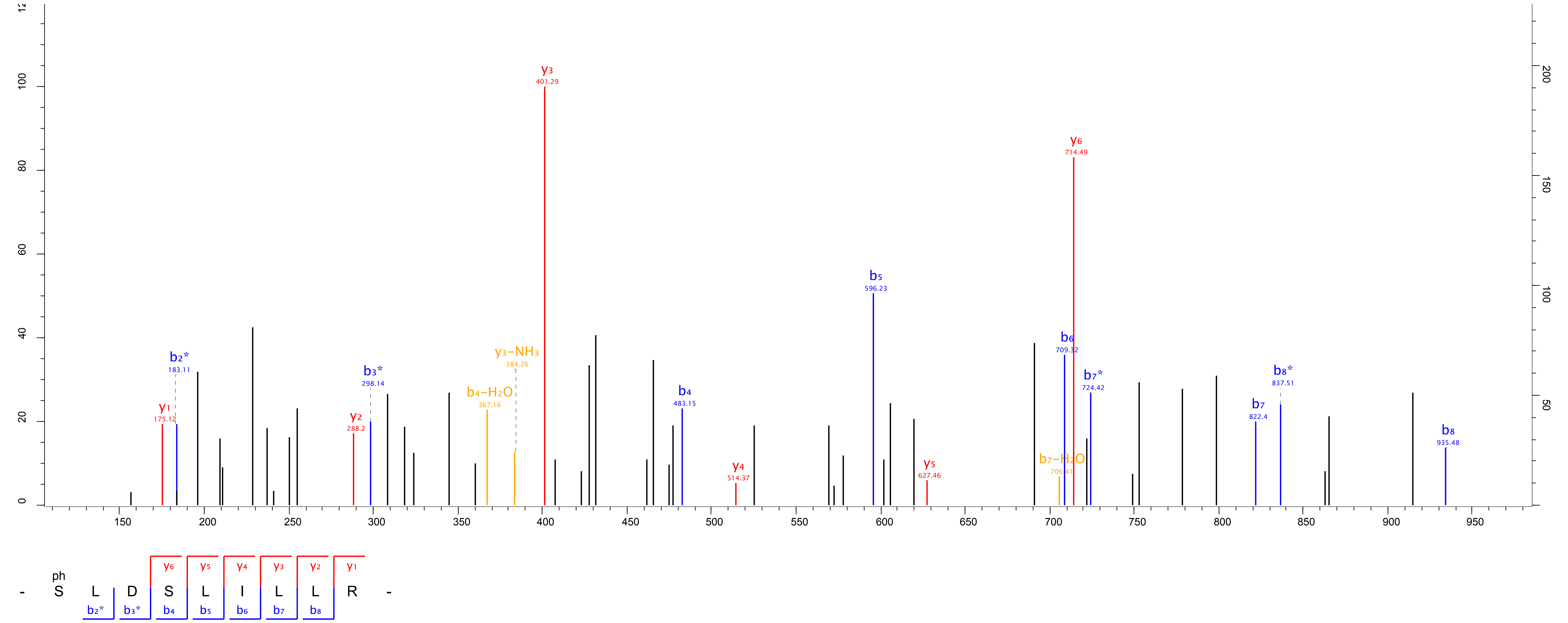

Raw file	Scan	Method	Score	m/z
20130708_PS_Phosphotest_6803wt_SDS01	13200	ITMS; CID	82.83	515.25

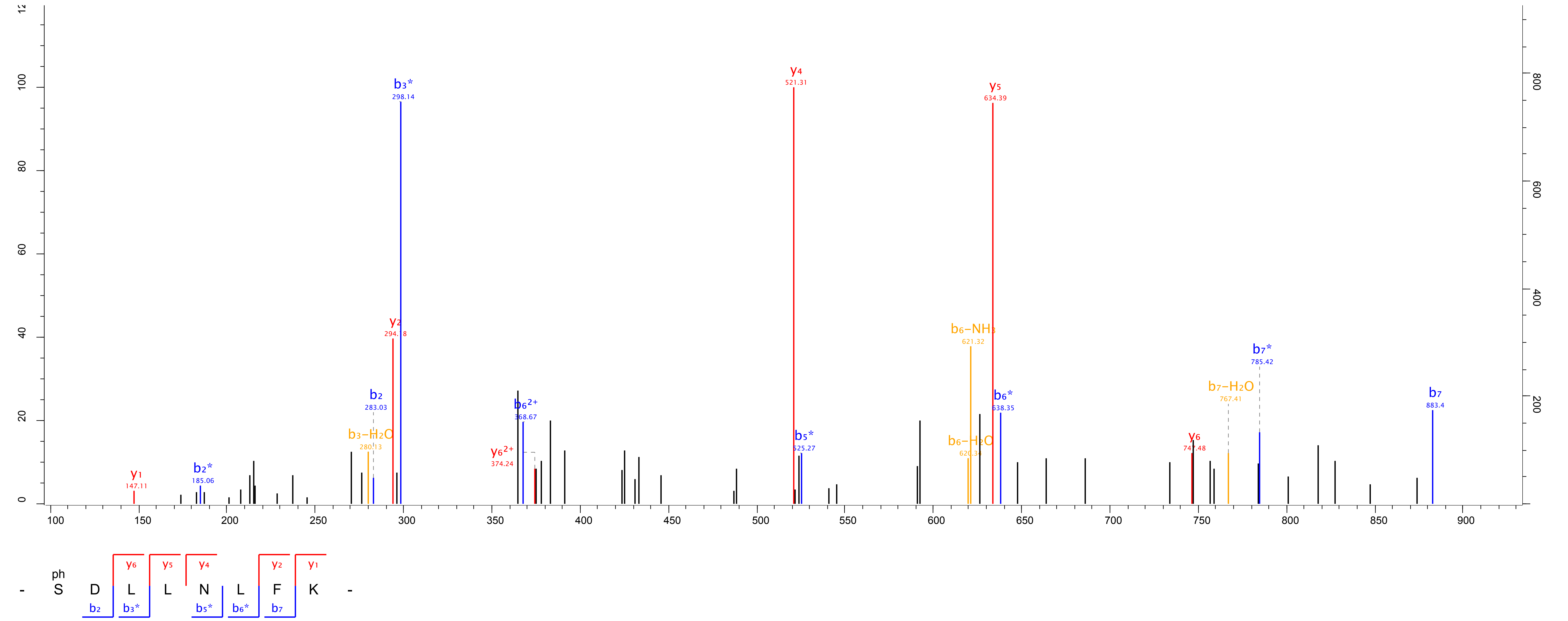

Raw file	Scan	Method	Score	m/z
20130708_PS_Phosphotest_6803wt_SDS01	13237	ITMS; CID	79.3	1068.17

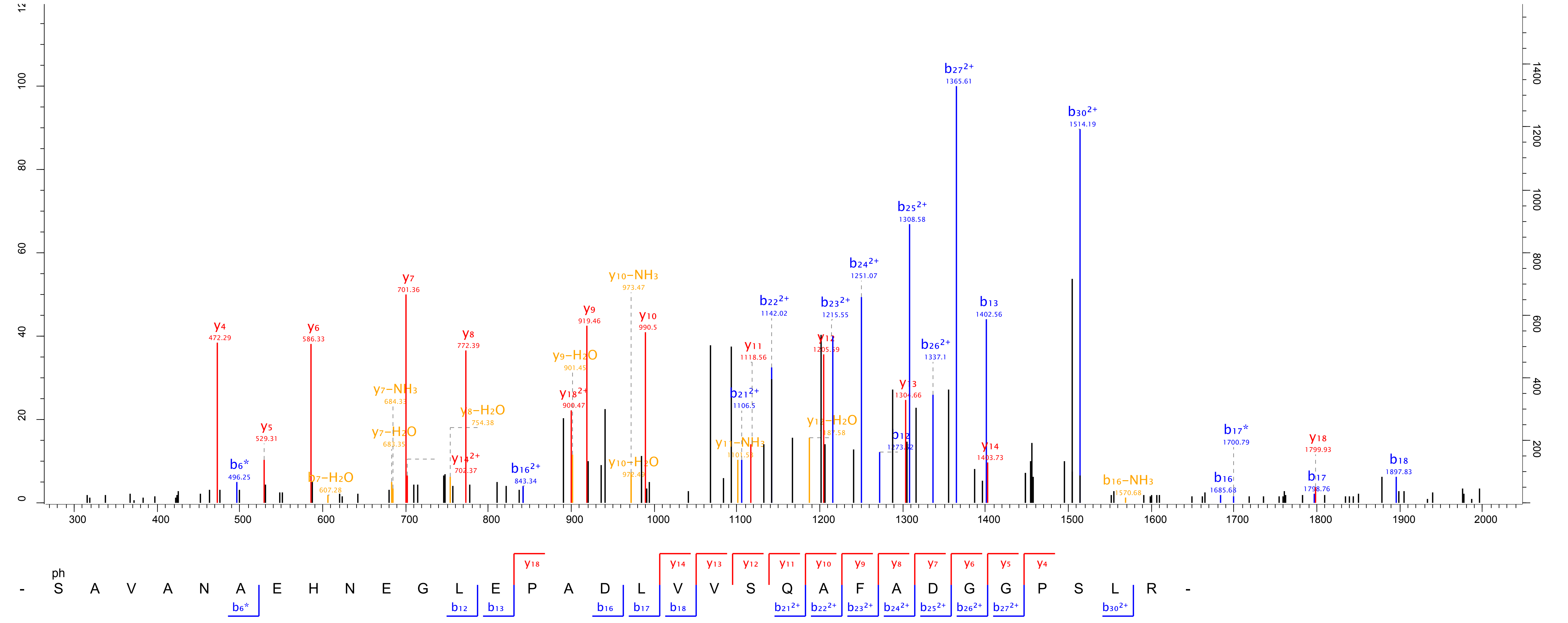

Raw file	Scan	Method	Score	m/z
20130708_PS_Phosphotest_6803wt_YPER01	13276	ITMS; CID	111.45	967.96

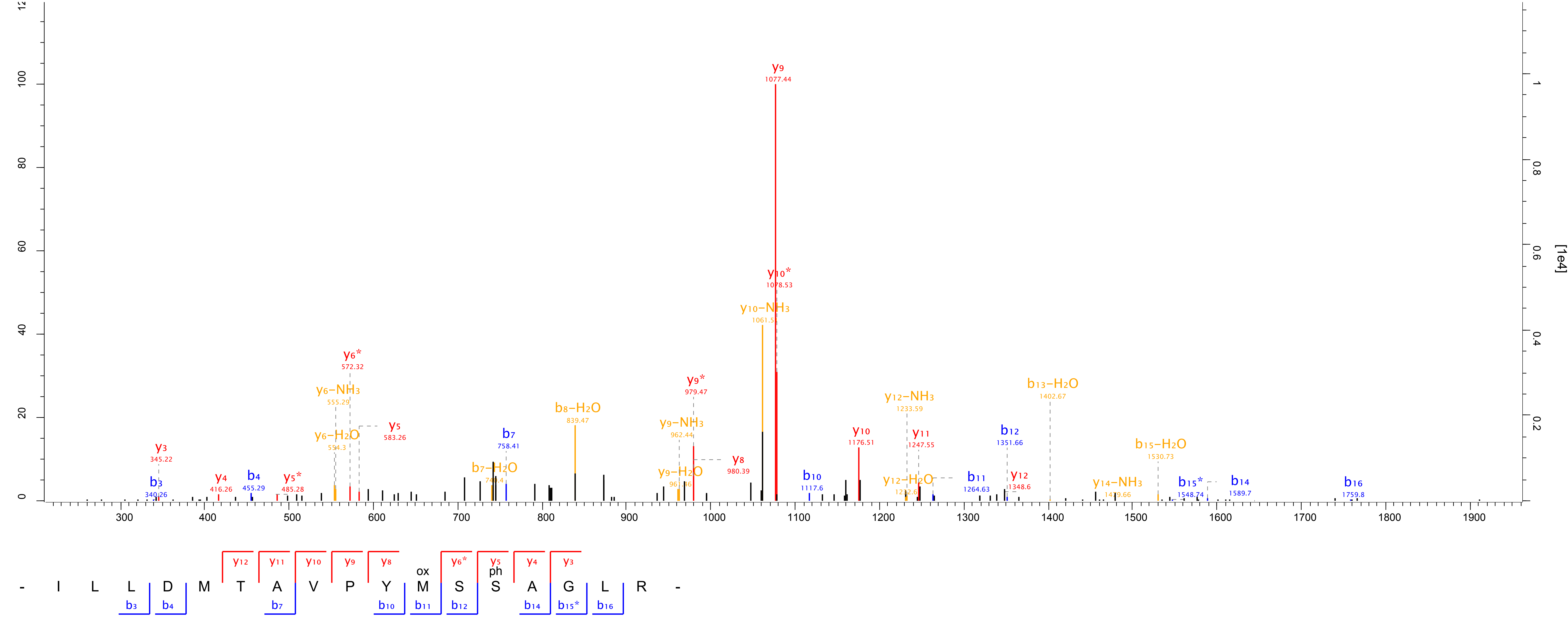

Raw file	Scan	Method	Score	m/z
20130708_PS_Phosphotest_6803wt_YPER03	13343	ITMS; CID	105.17	774.88

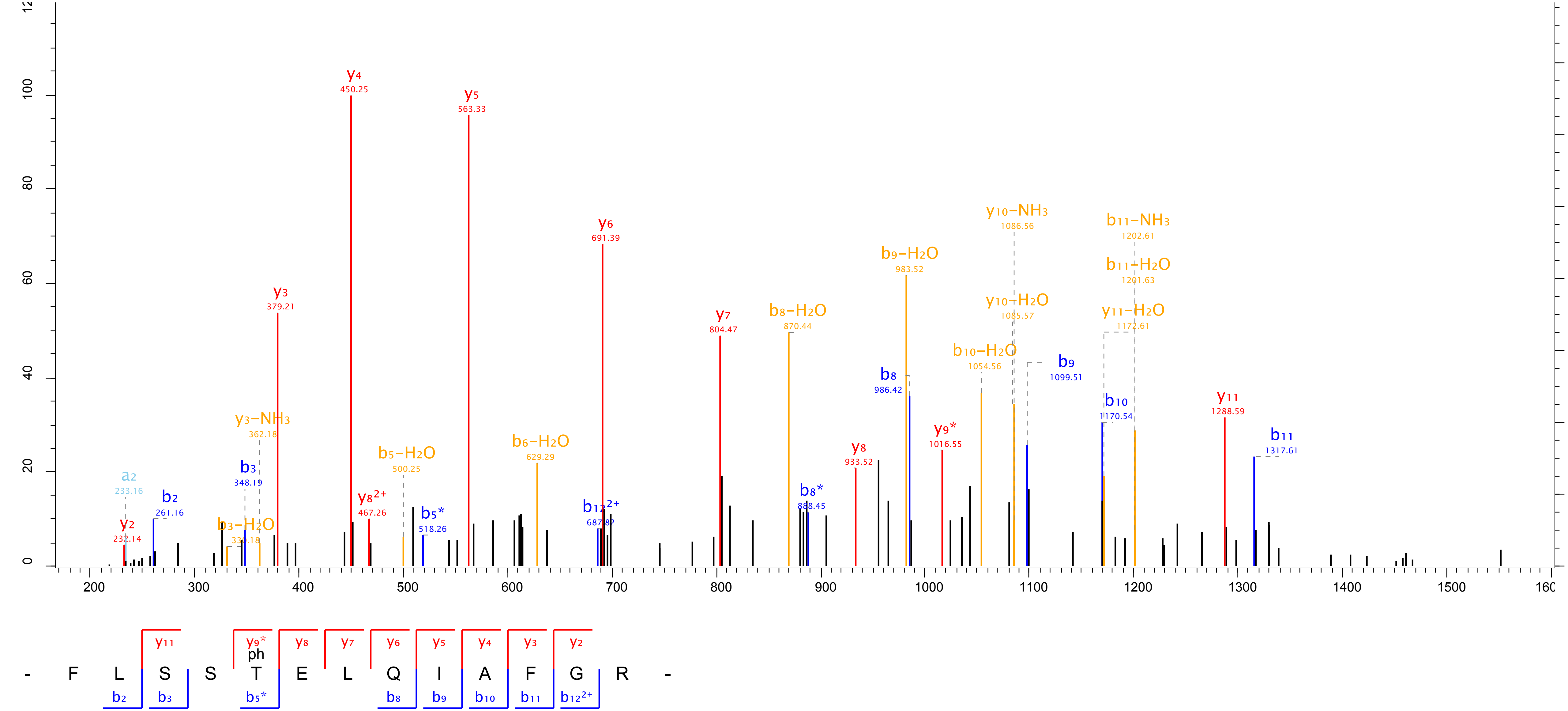

Raw file	Scan	Method	Score	m/z
20130708_PS_Phosphotest_6803wt_SDS01	13383	ITMS; CID	145.94	813.89

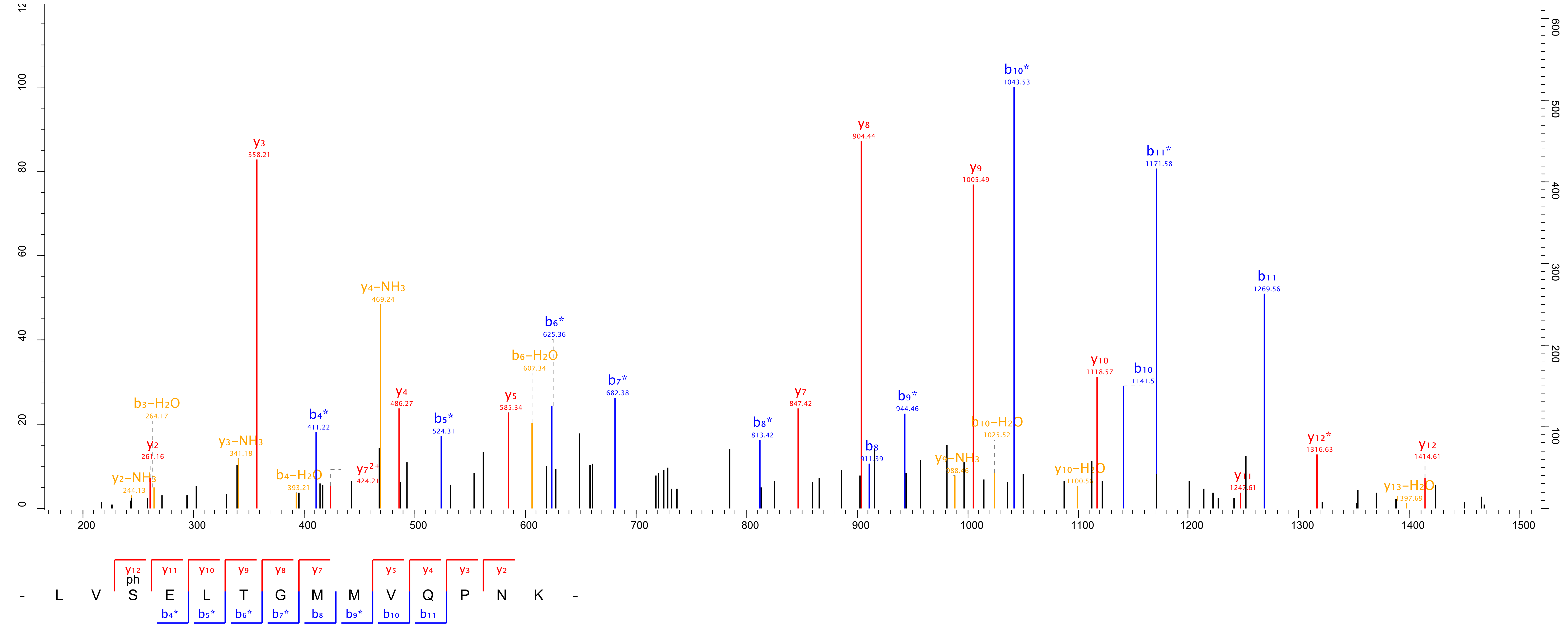

Raw file	Scan	Method	Score	m/z
20130708_PS_Phosphotest_6803wt_SDS01	13419	ITMS; CID	133.9	752.41

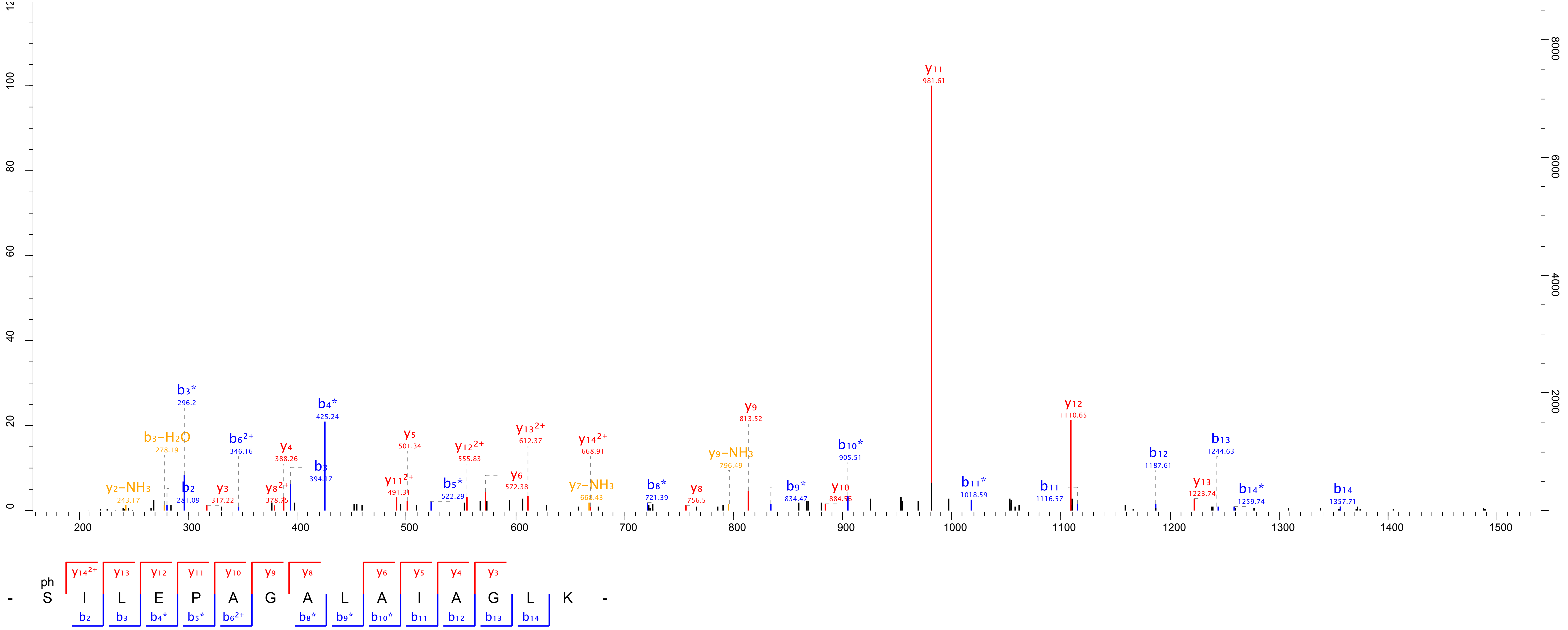

Raw file	Scan	Method	Score	m/z
20130708_PS_Phosphotest_6803wt_SDS01	13527	ITMS; CID	108.9	565.29

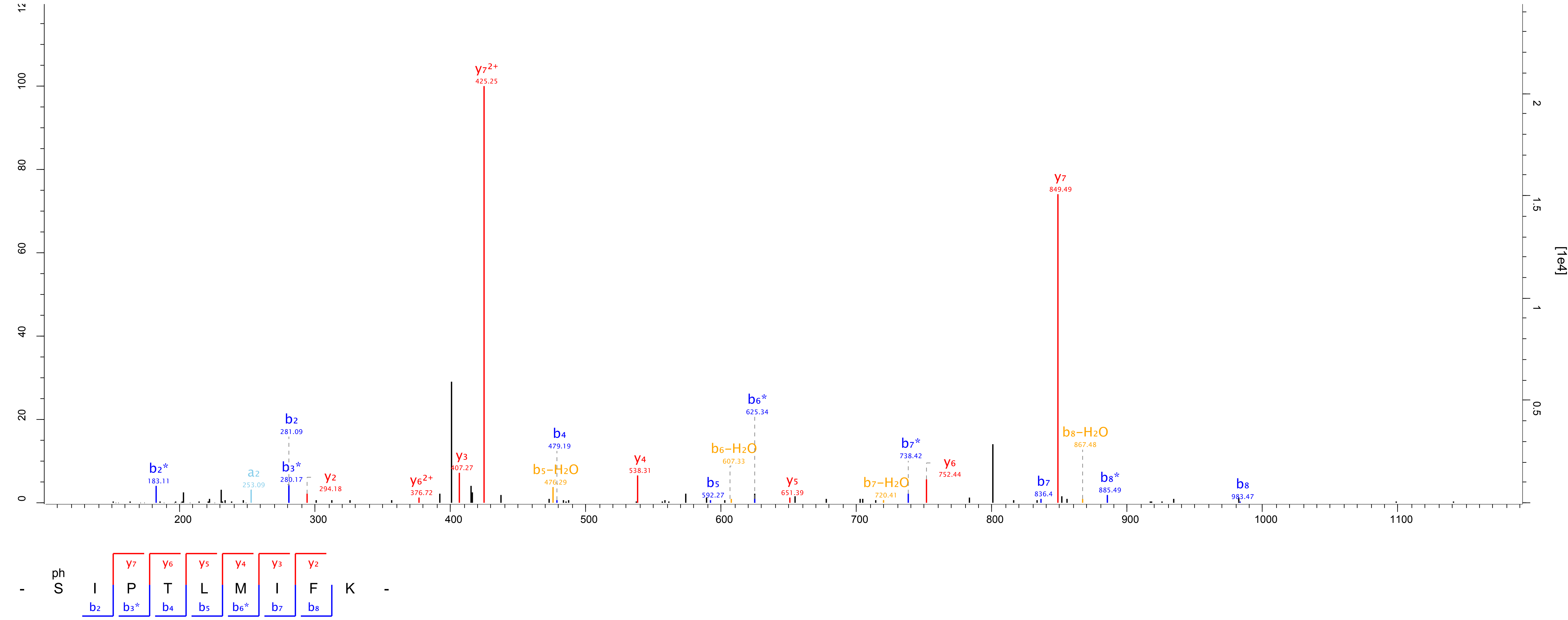

Raw file	Scan	Method	Score	m/z
20130708_PS_Phosphotest_6803wt_YPER01	1367	ITMS; CID	128.12	535.22

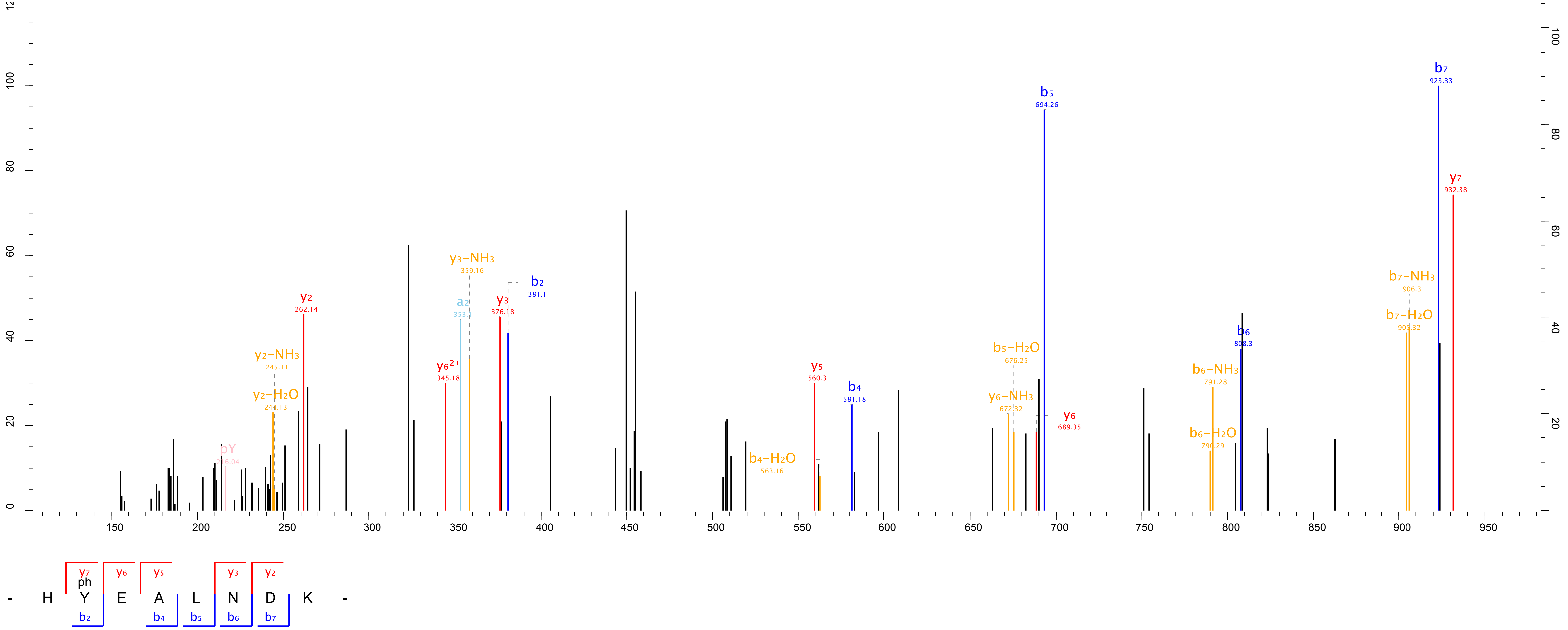

Raw file	Scan	Method	Score	m/z
20130708_PS_Phosphotest_6803wt_SDS01	13752	ITMS; CID	155.06	746.36

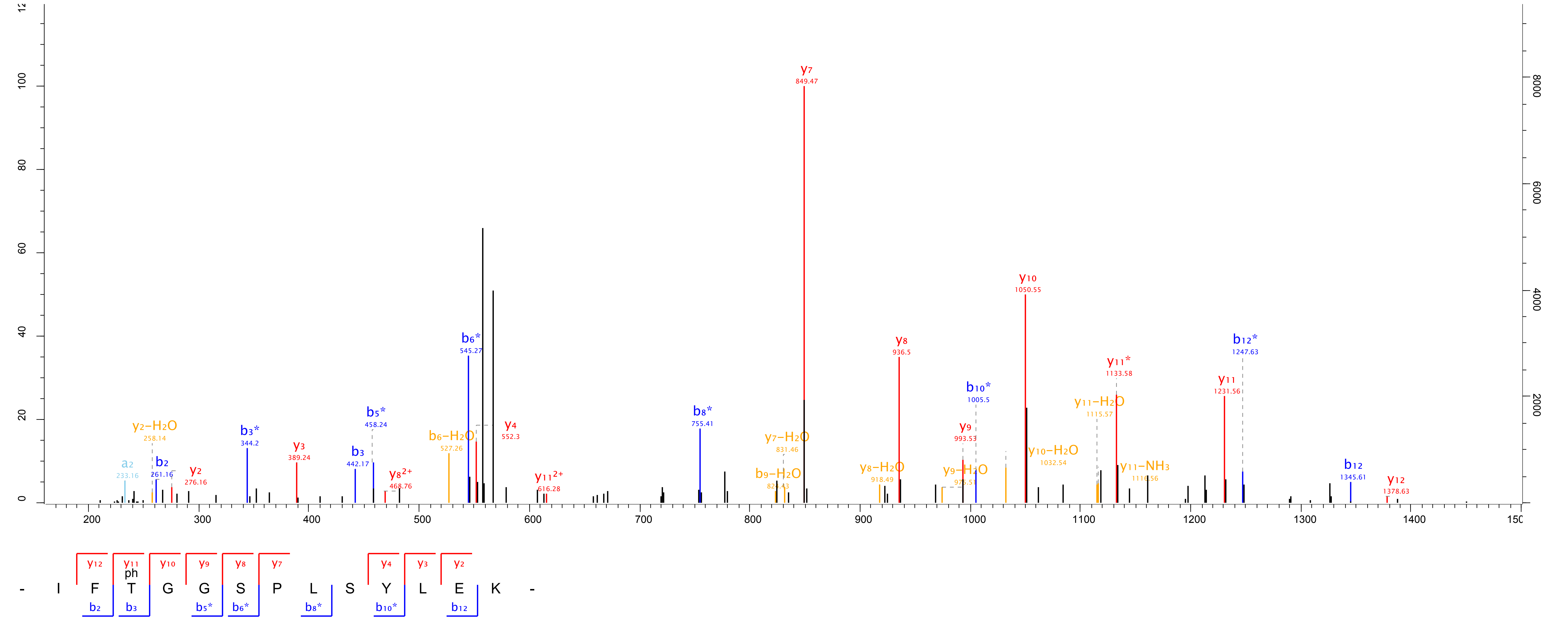

Raw file	Scan	Method	Score	m/z
20130708_PS_Phosphotest_6803wt_SDS01	13932	ITMS; CID	181.51	815.39

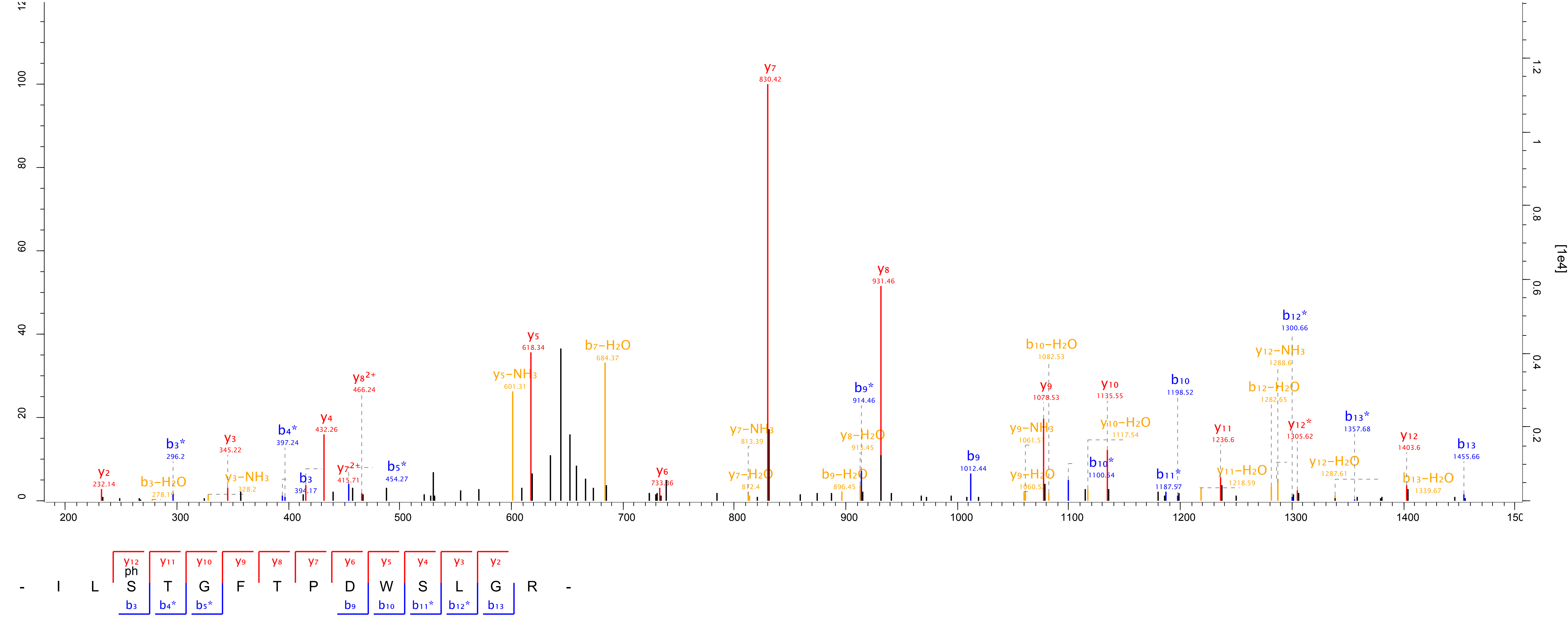

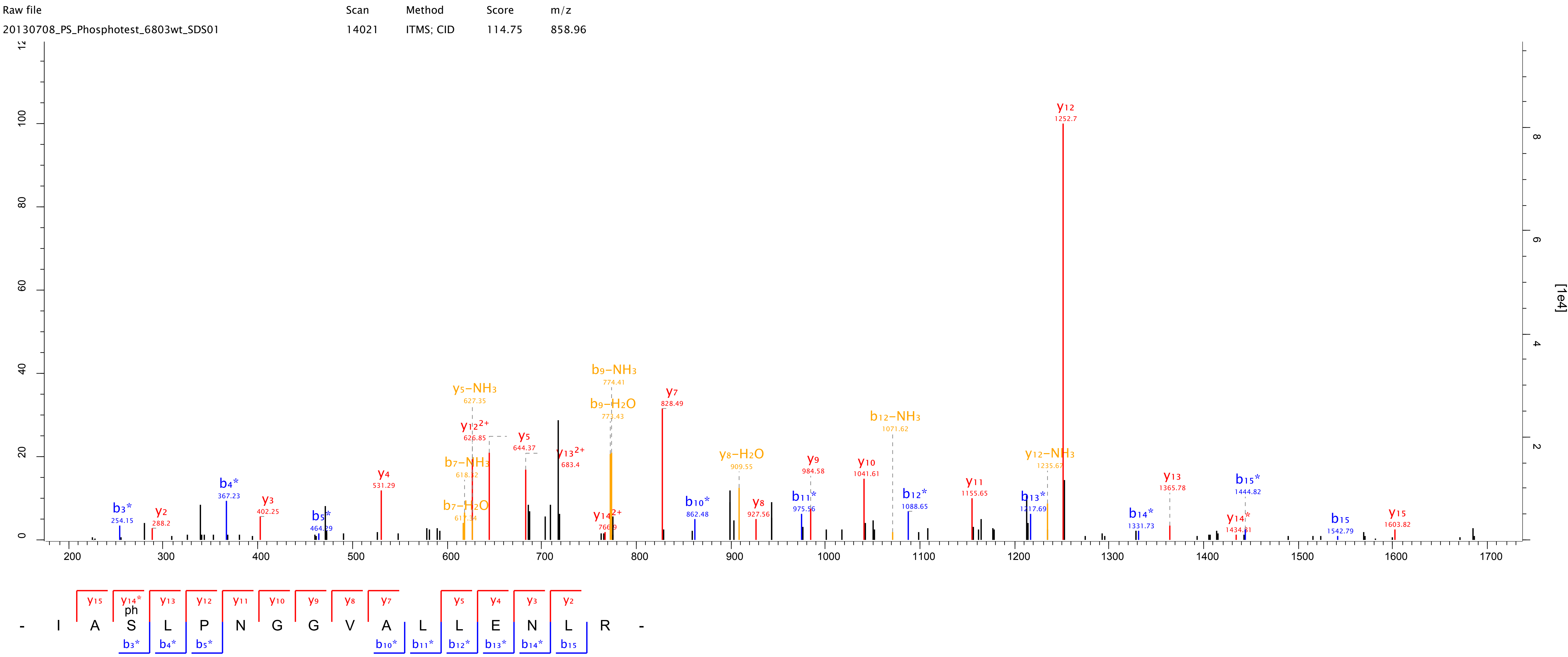
Raw file Scan Method Score m/z
20130708_PS_Phosphotest_6803wt_SDS01 14021 ITMS; CID 114.75 858.96
y12 1252.7
y7 828.49
b9-NH3 774.41
b9-H2O 773.43
y5-NH3 627.35
y12 2+ 626.85
y5 644.37
y13 2+ 683.4
b12-NH3 1071.62
y8-H2O 909.55
y12-NH3 1235.67
- I A S L P N G G V A L L E N L R -
ph
[1e4]

Raw file	Scan	Method	Score	m/z
20130708_PS_Phosphotest_6803wt_SDS01	14341	ITMS; CID	119.77	708.86

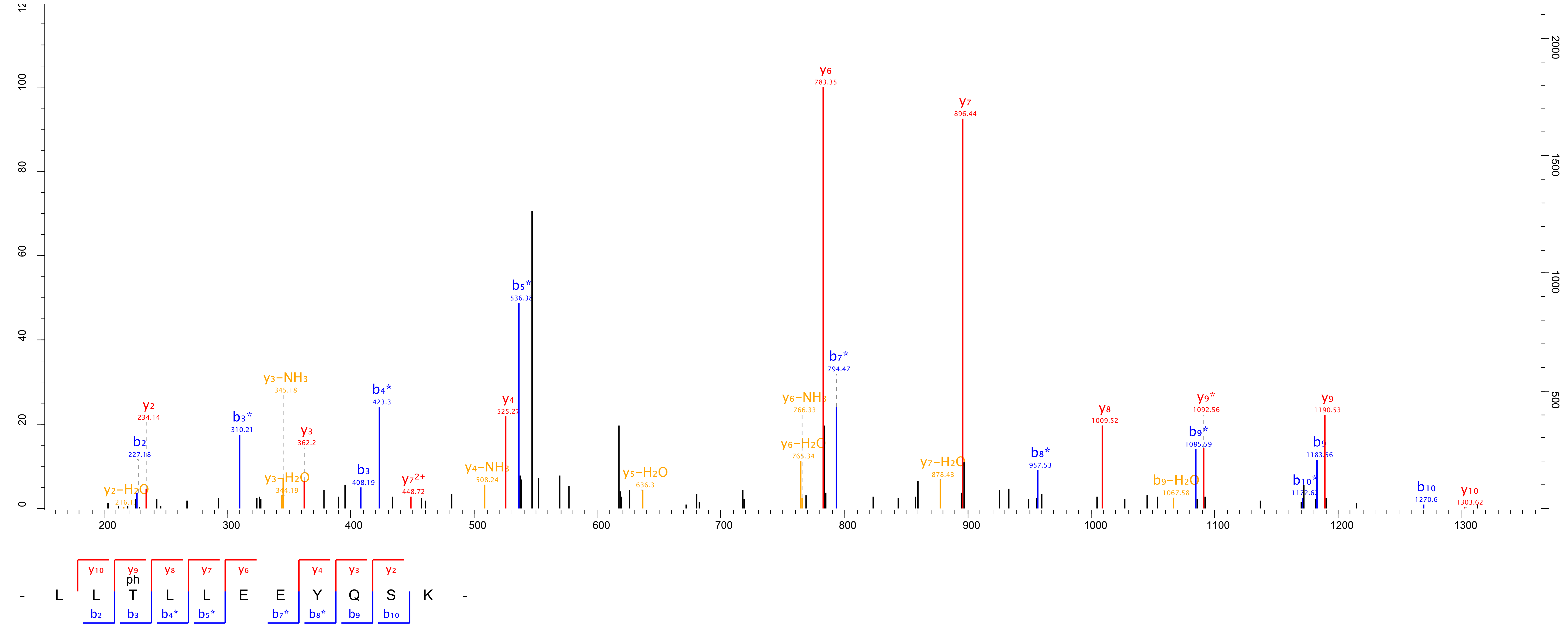

Raw file	Scan	Method	Score	m/z
20130708_PS_Phosphotest_6803wt_SDS01	14402	ITMS; CID	60.49	728.68

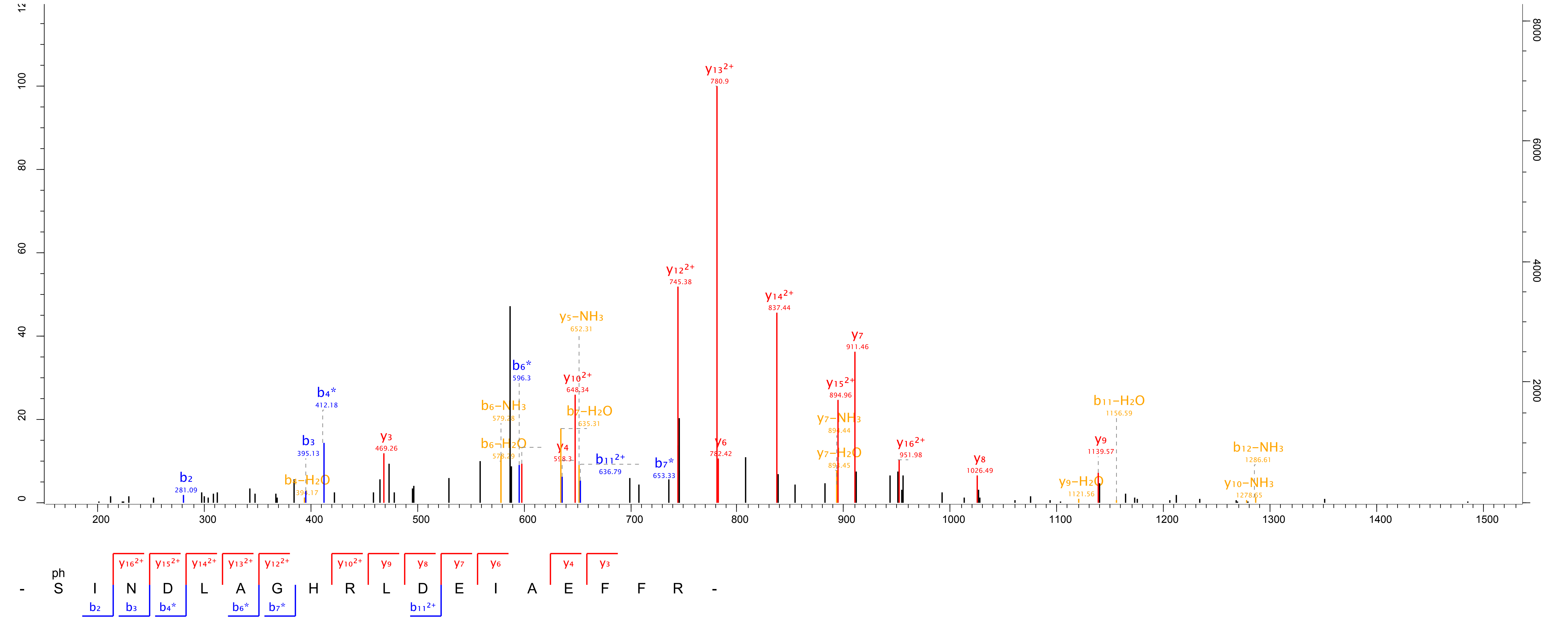

Raw file	Scan	Method	Score	m/z
20130708_PS_Phosphotest_6803wt_YPER01	14454	ITMS; CID	165.53	1027.46

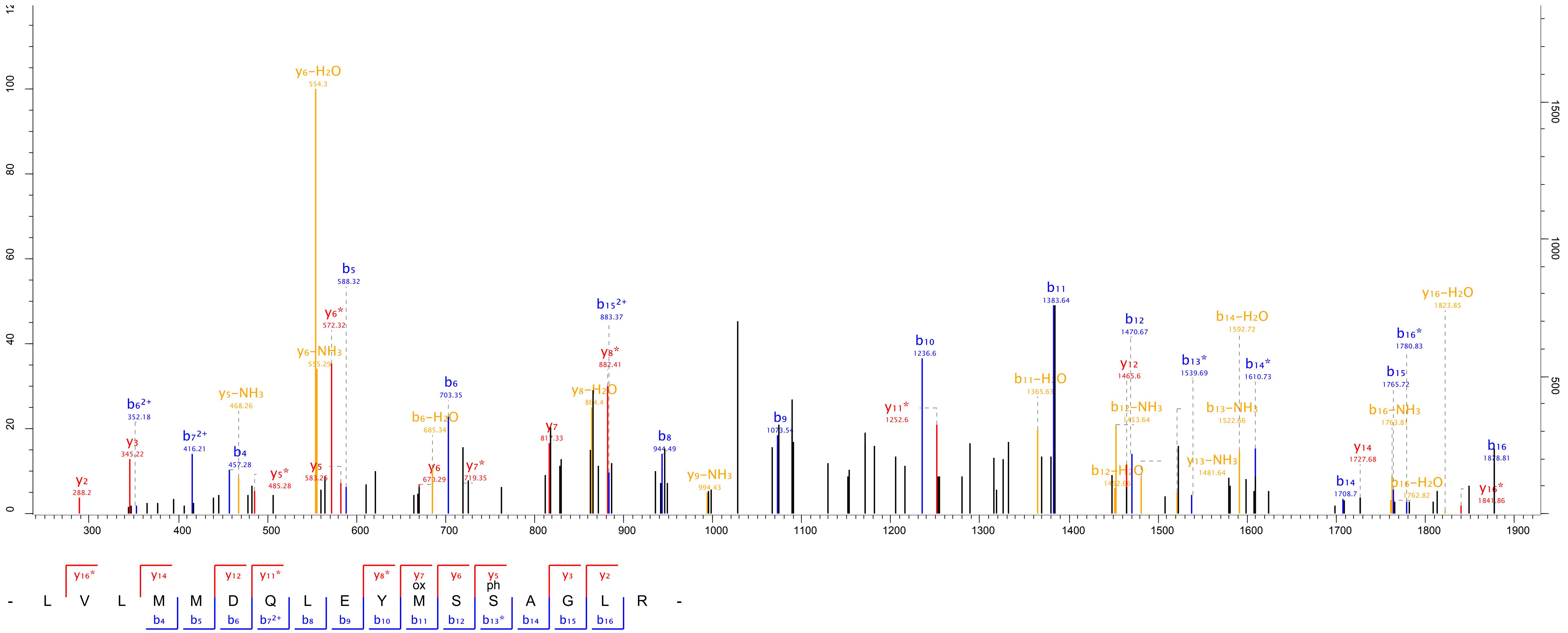

Raw file	Scan	Method	Score	m/z
20130708_PS_Phosphotest_6803wt_SDS02	15006	ITMS; CID	98.16	557.3

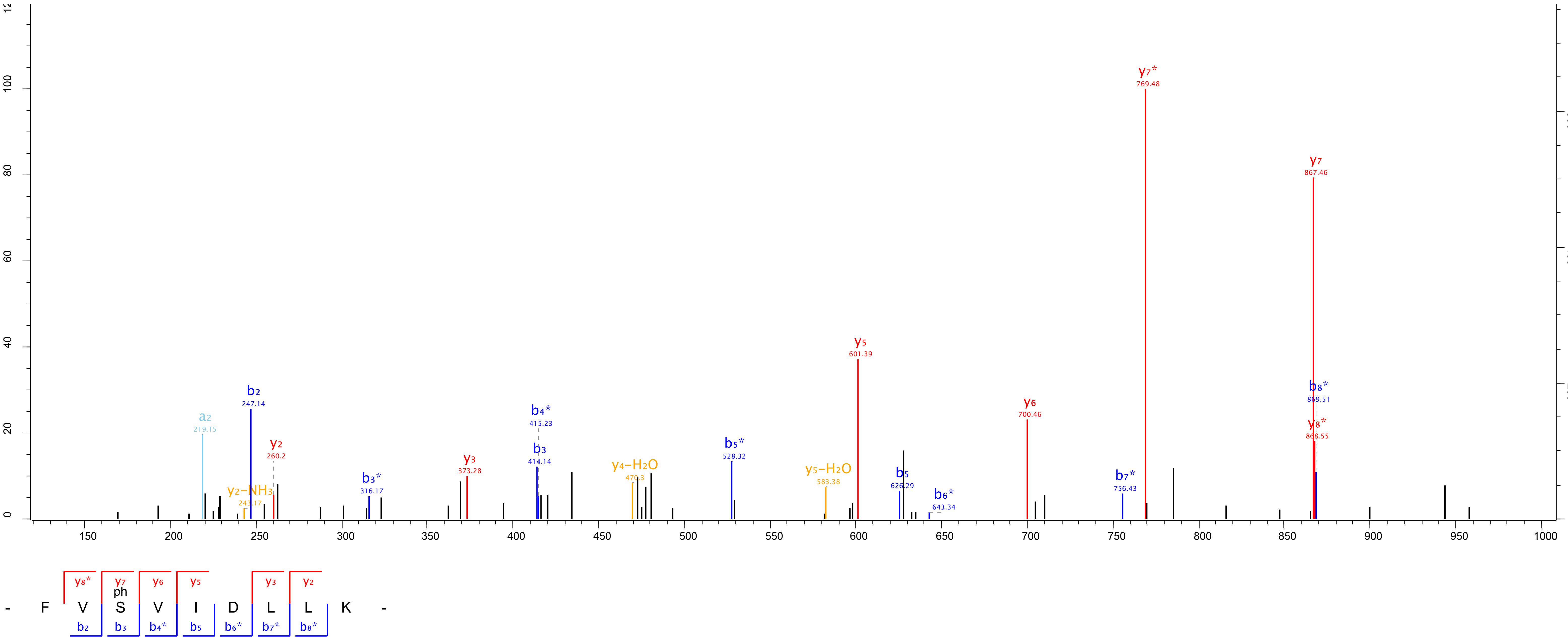

Raw file	Scan	Method	Score	m/z
20130708_PS_Phosphotest_6803wt_SDS01	15025	ITMS; CID	142.1	612.81

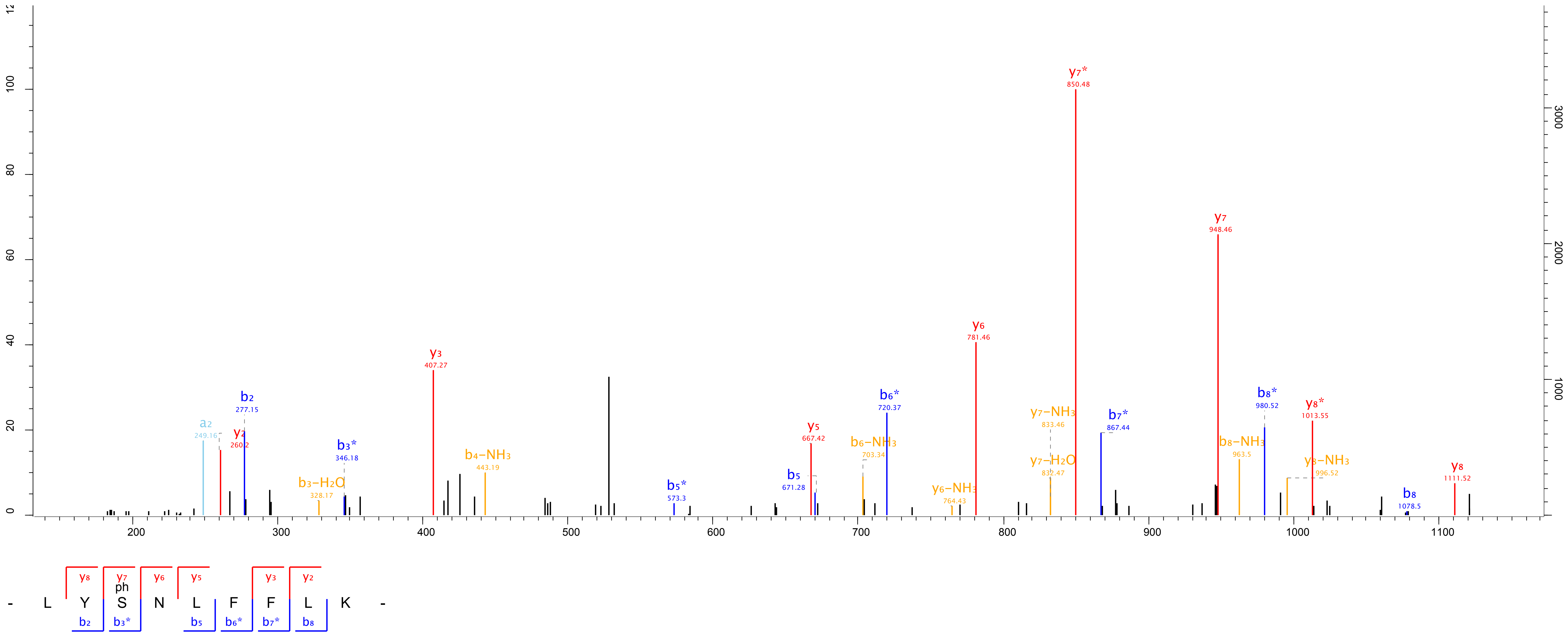

Raw file	Scan	Method	Score	m/z
20130708_PS_Phosphotest_6803wt_SDS01	15087	ITMS; CID	157.74	741.38

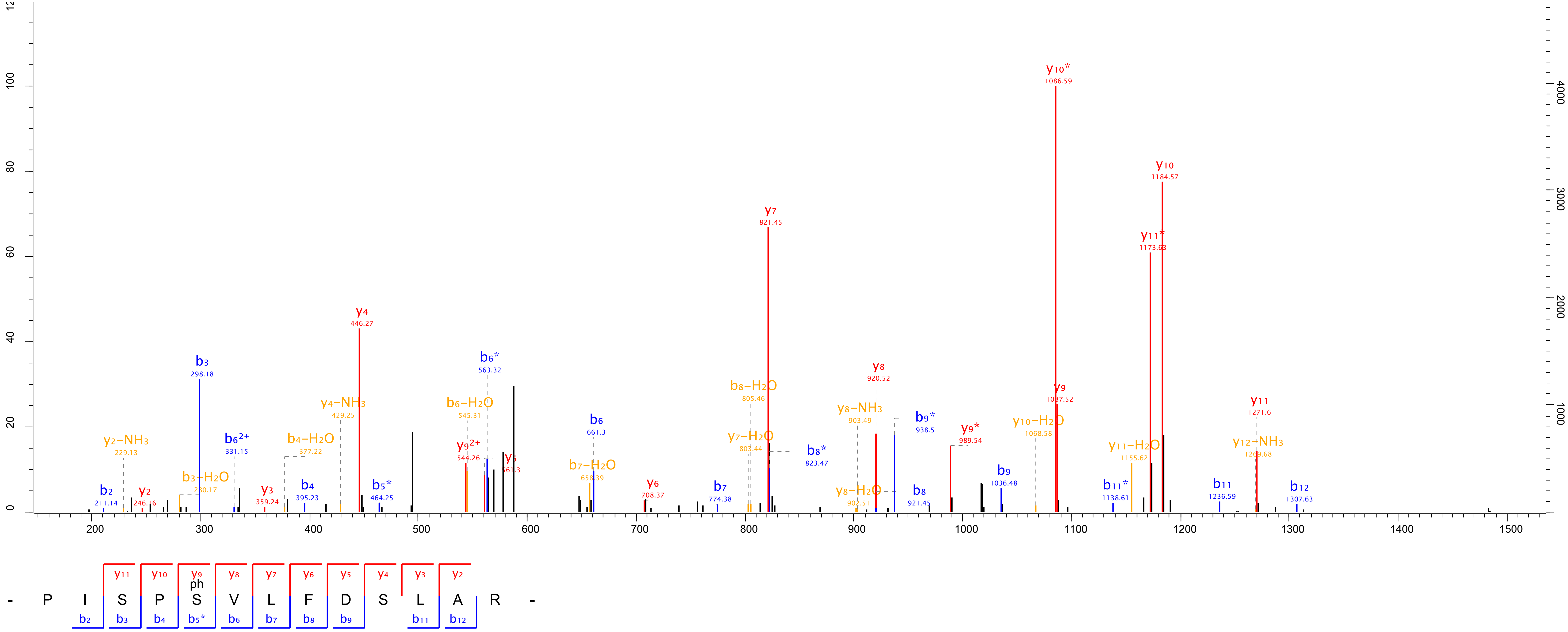

Raw file	Scan	Method	Score	m/z
20130708_PS_Phosphotest_6803wt_SDS01	15132	ITMS; CID	158.93	763.86

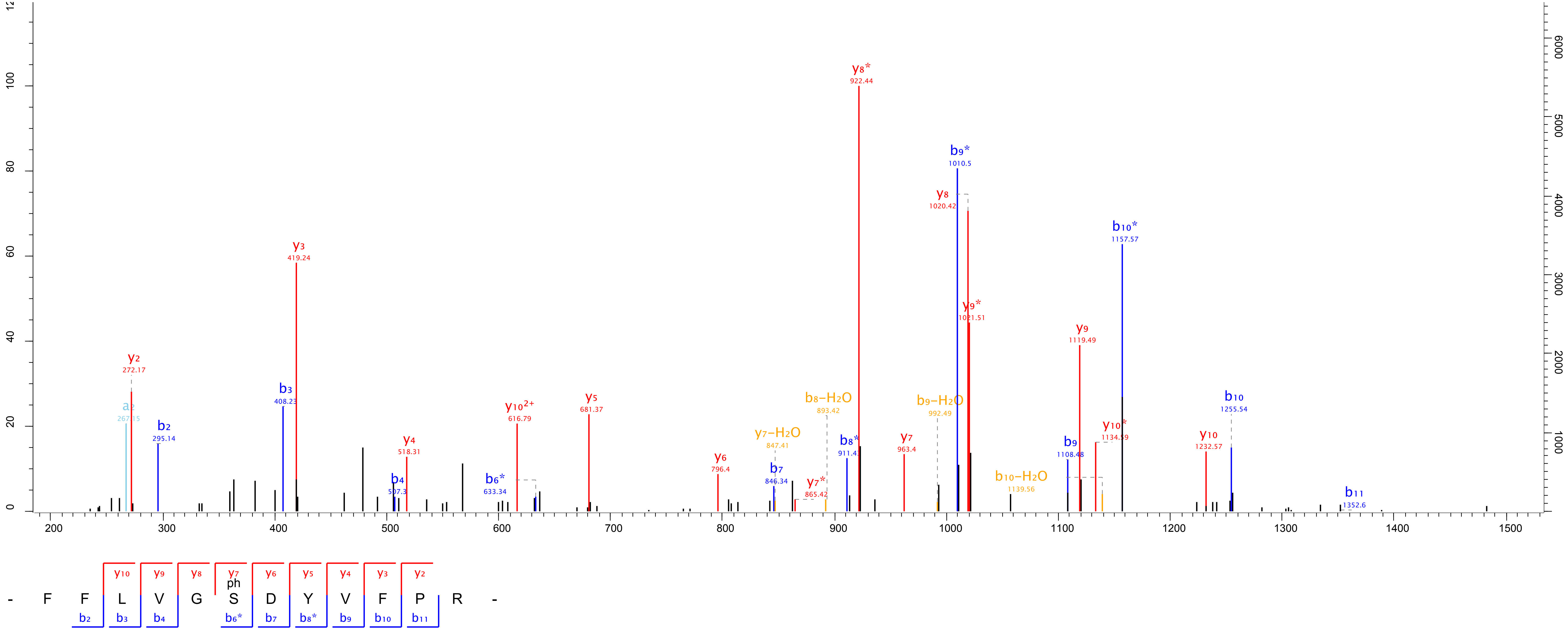

Raw file	Scan	Method	Score	m/z
20130708_PS_Phosphotest_6803wt_SDS05	15157	ITMS; CID	159.81	868.4

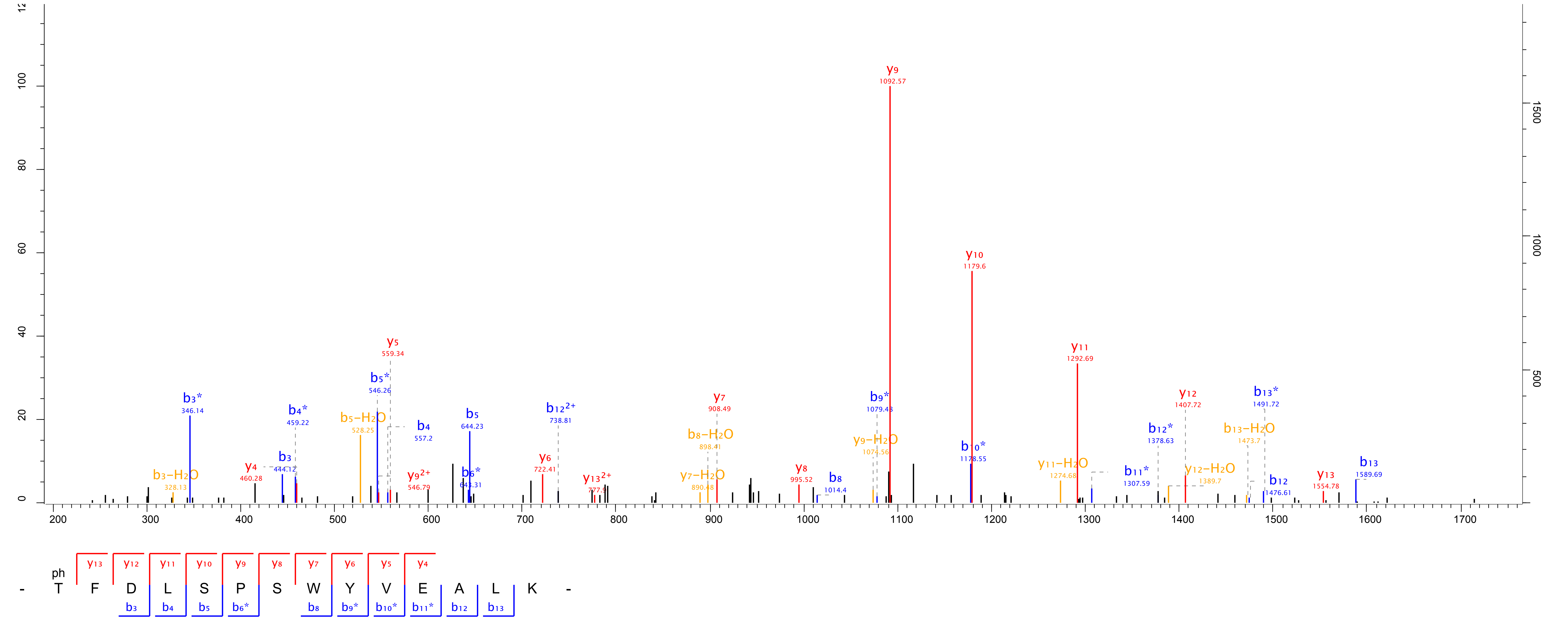

Raw file	Scan	Method	Score	m/z
20130708_PS_Phosphotest_6803wt_SDS01	15374	ITMS; CID	279.12	829.41

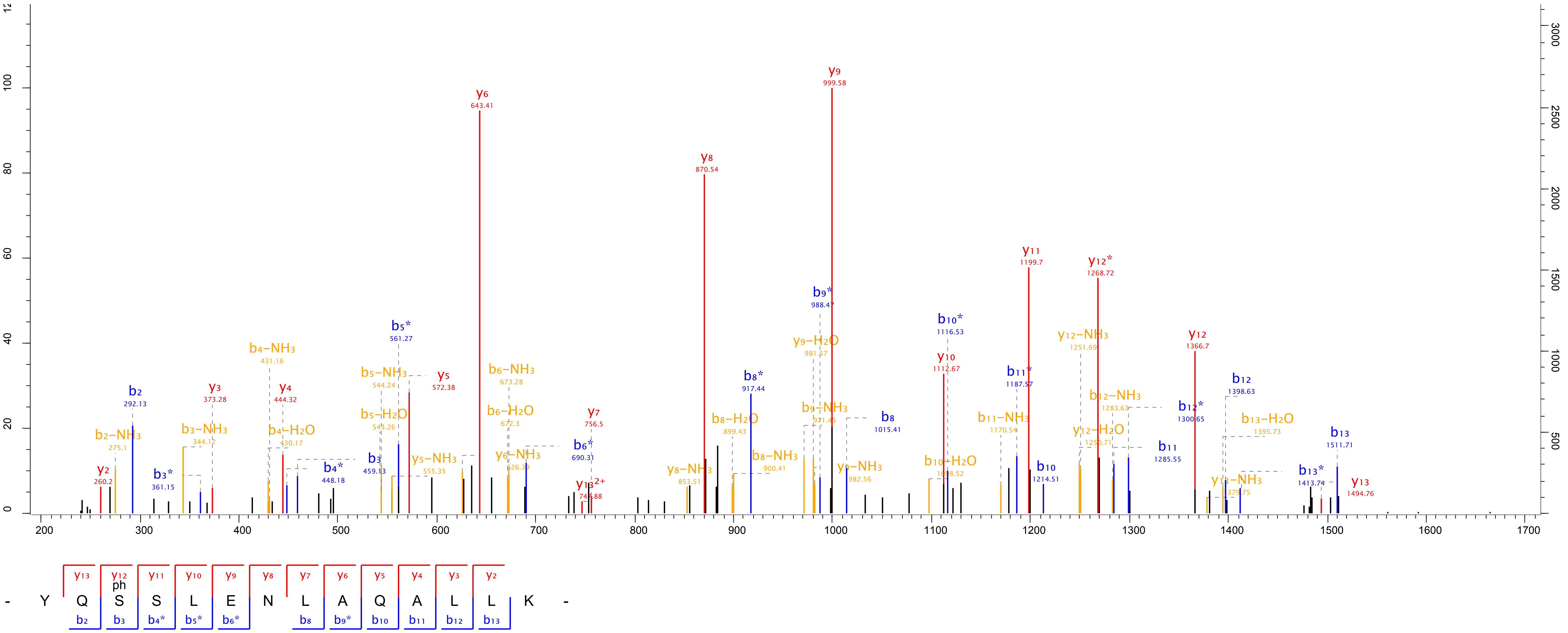

Raw file	Scan	Method	Score	m/z
20130708_PS_Phosphotest_6803wt_SDS03	15392	ITMS; CID	139.76	521.29

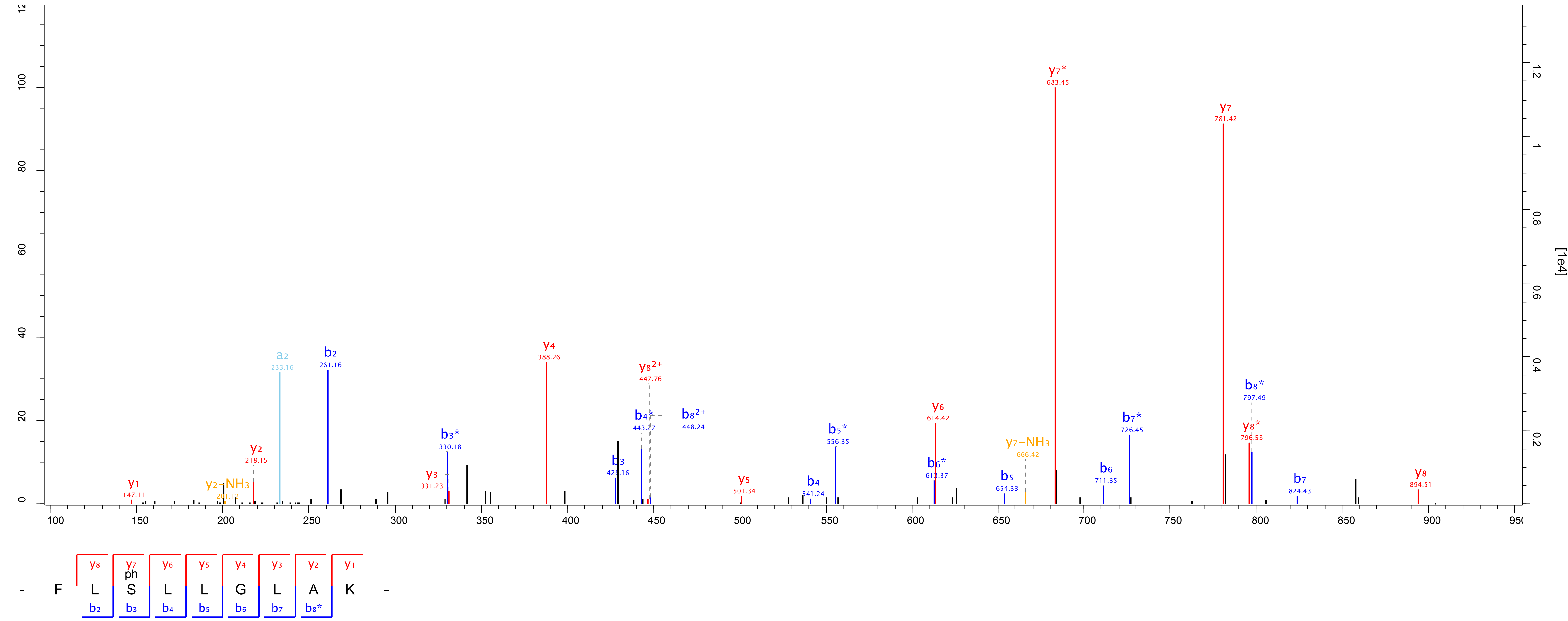

Raw file	Scan	Method	Score	m/z
20130708_PS_Phosphotest_6803wt_SDS02	15468	ITMS; CID	102.39	717.39

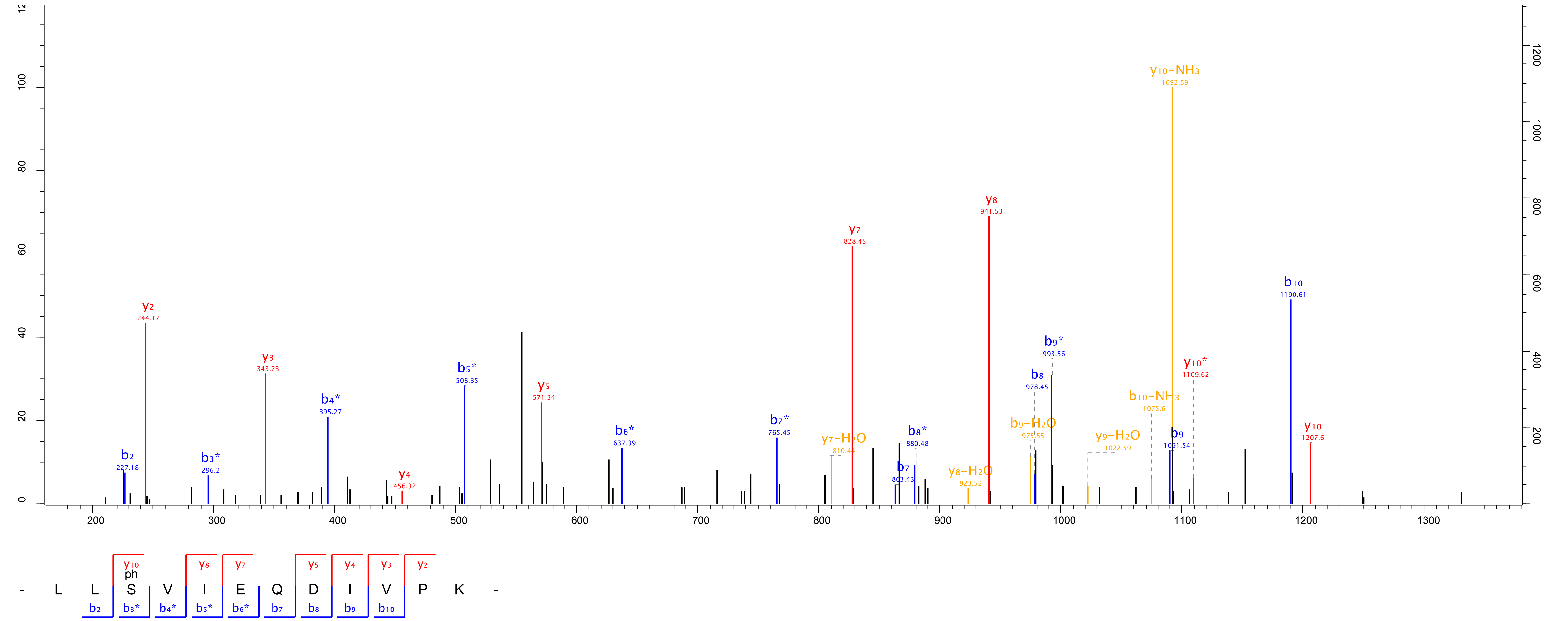

Raw file	Scan	Method	Score	m/z
20130708_PS_Phosphotest_6803wt_SDS01	15843	ITMS; CID	151.99	724.85

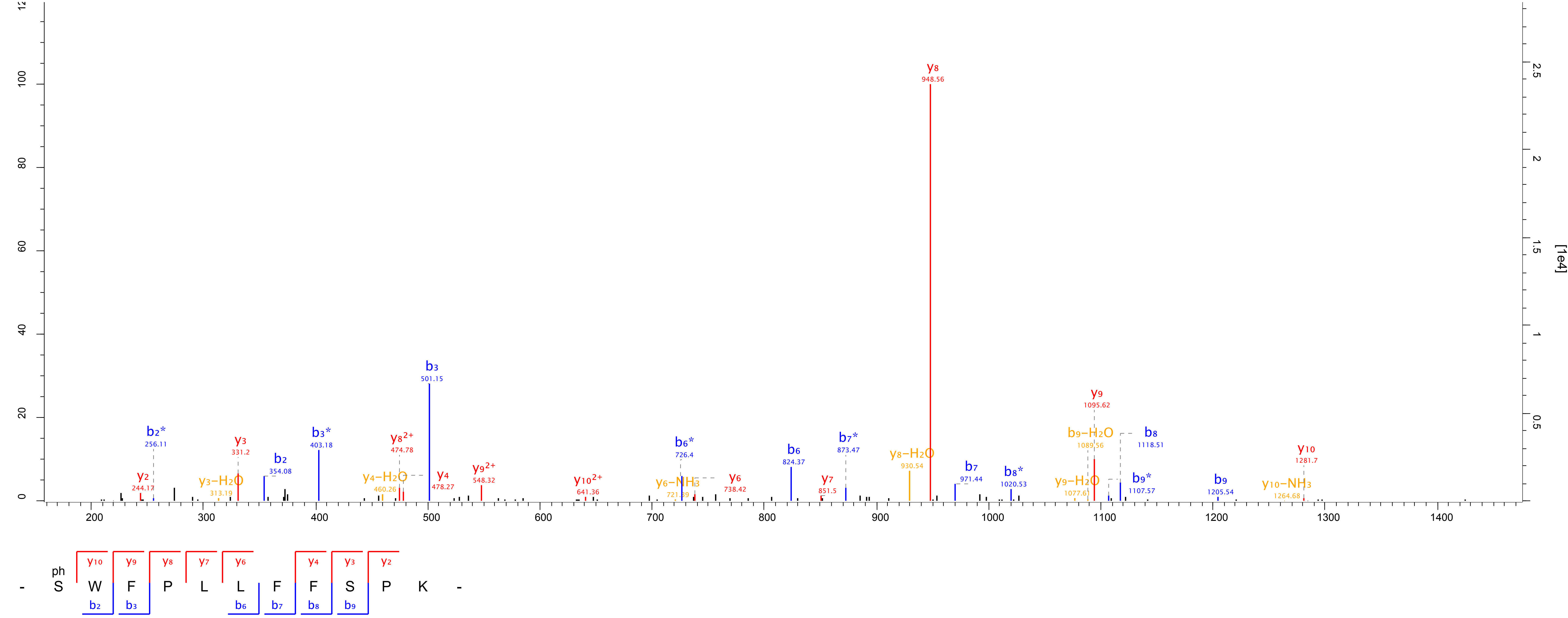

Raw file	Scan	Method	Score	m/z
20130708_PS_Phosphotest_6803wt_YPER03	15951	ITMS; CID	254.68	1061.53

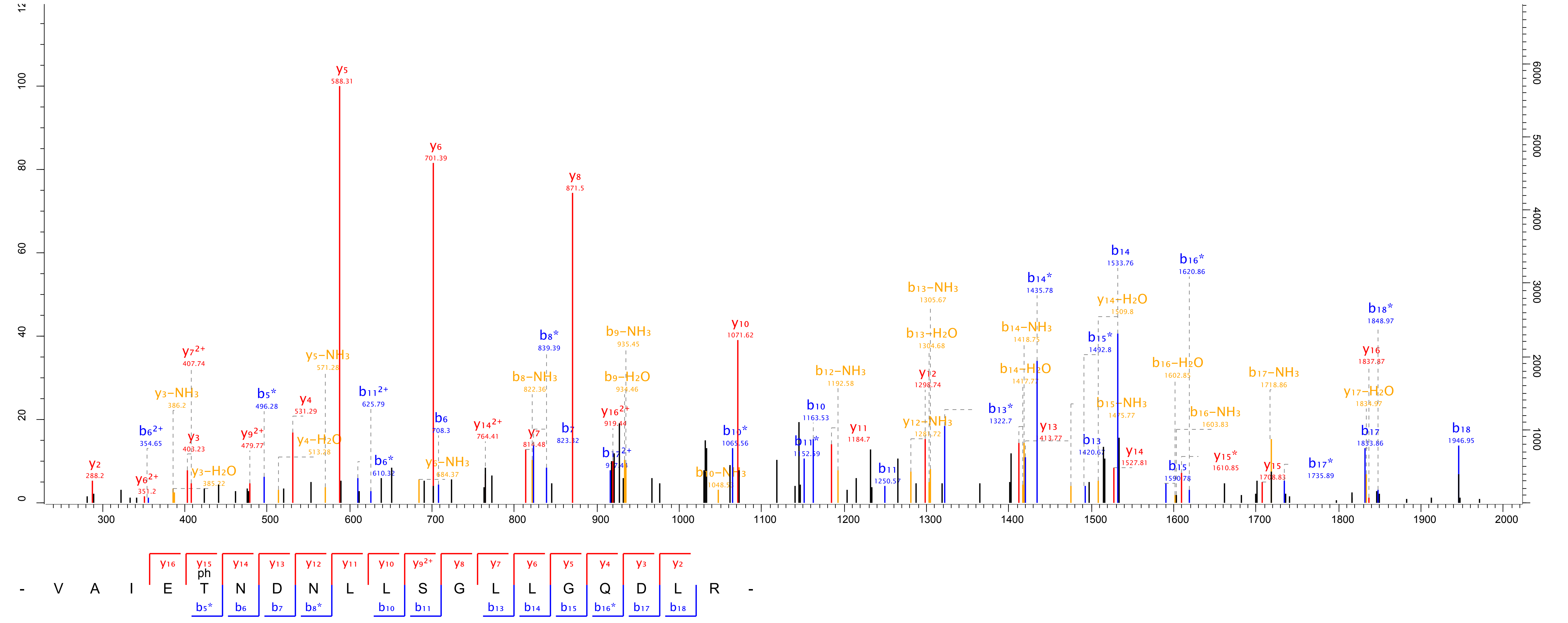

Raw file	Scan	Method	Score	m/z
20130708_PS_Phosphotest_6803wt_SDS02	1662	ITMS; CID	116.74	563.26

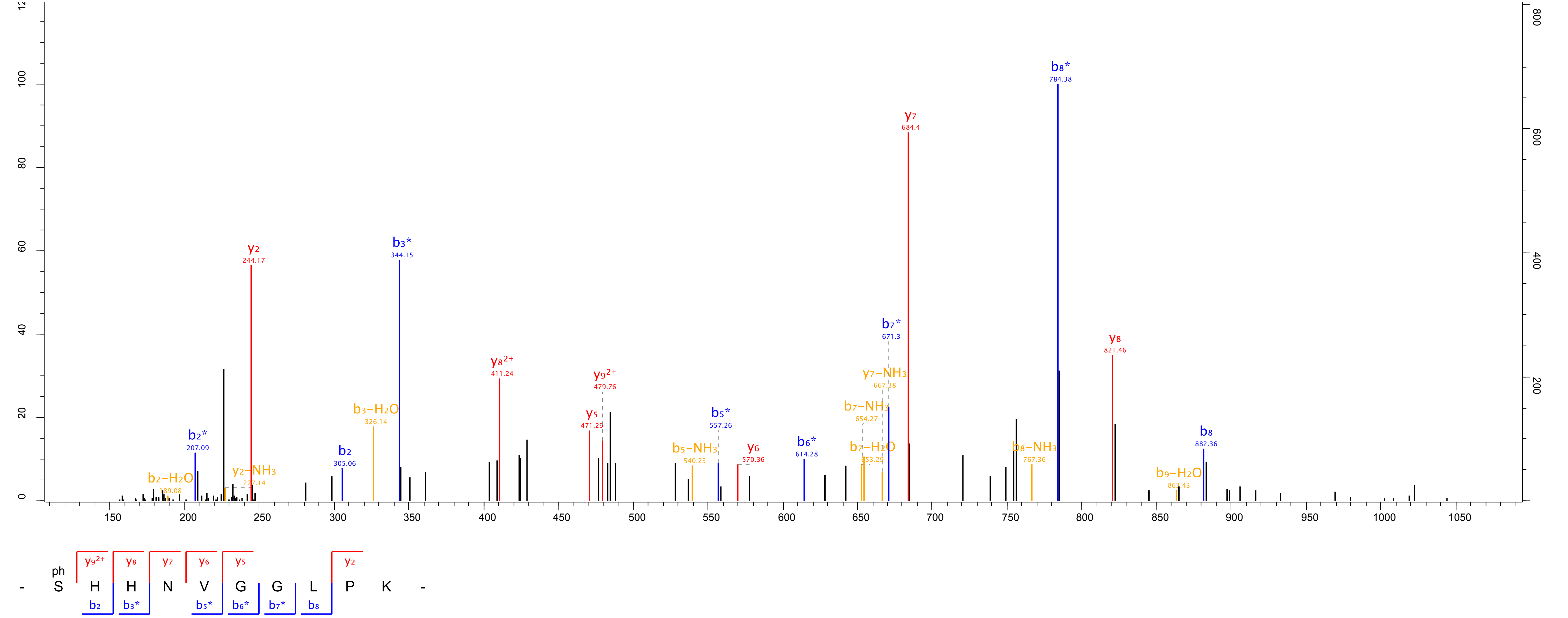

Raw file	Scan	Method	Score	m/z
20130708_PS_Phosphotest_6803wt_SDS01	16652	ITMS; CID	137.61	774.88

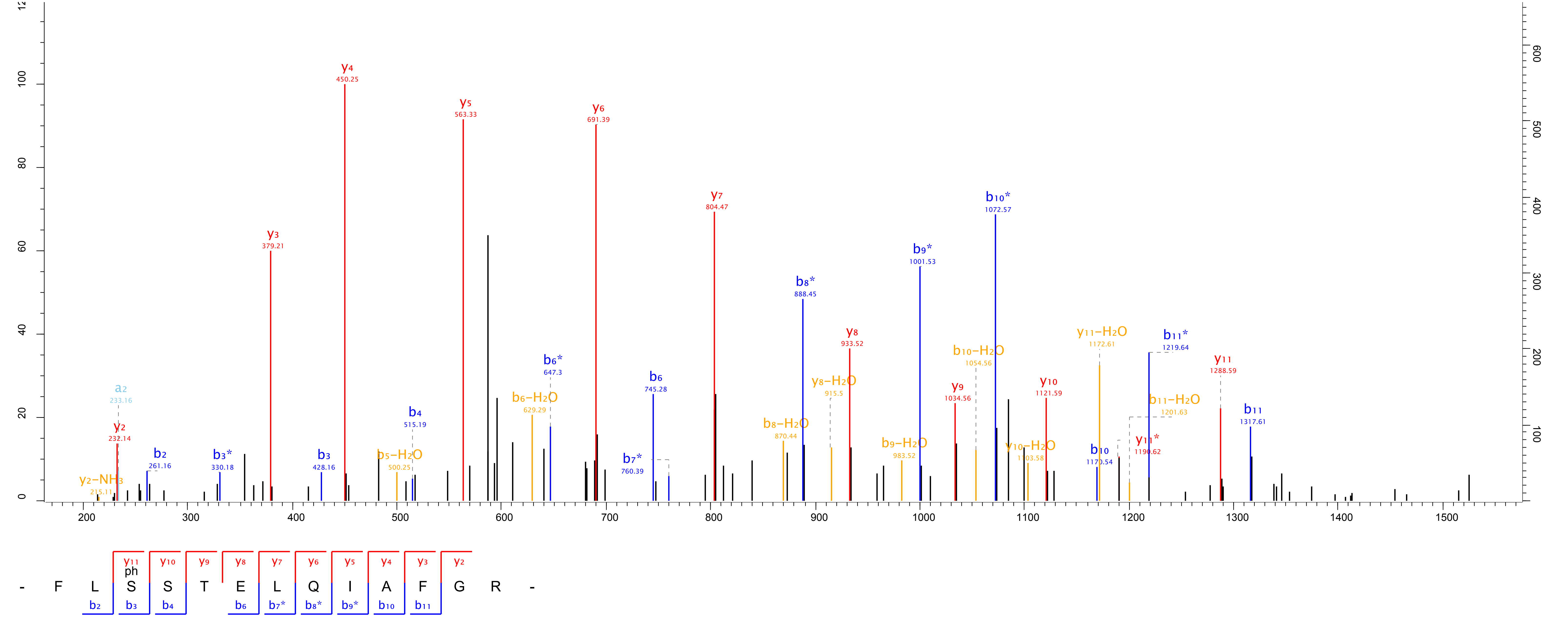

Raw file	Scan	Method	Score	m/z
20130708_PS_Phosphotest_6803wt_SDS01	1974	ITMS; CID	76.23	496.73

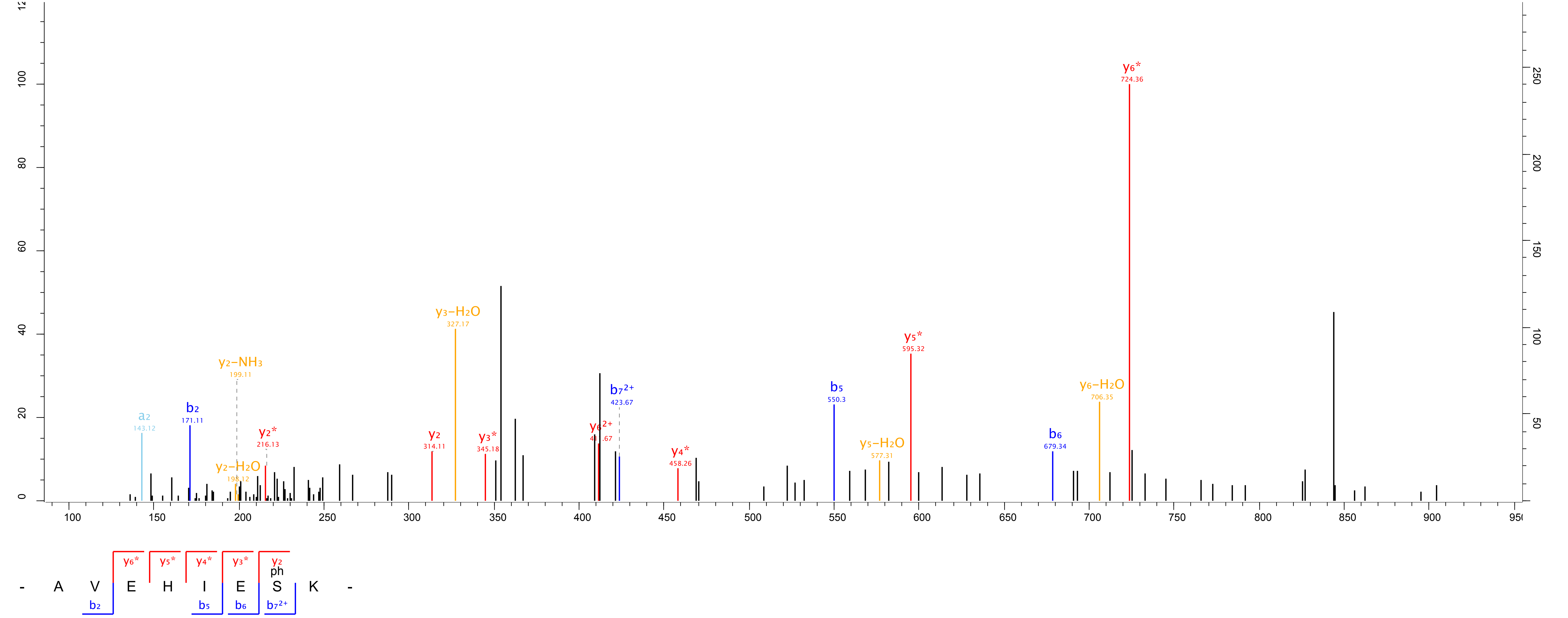

Raw file	Scan	Method	Score	m/z
20130708_PS_Phosphotest_6803wt_YPER01	1983	ITMS; CID	77.74	463.21

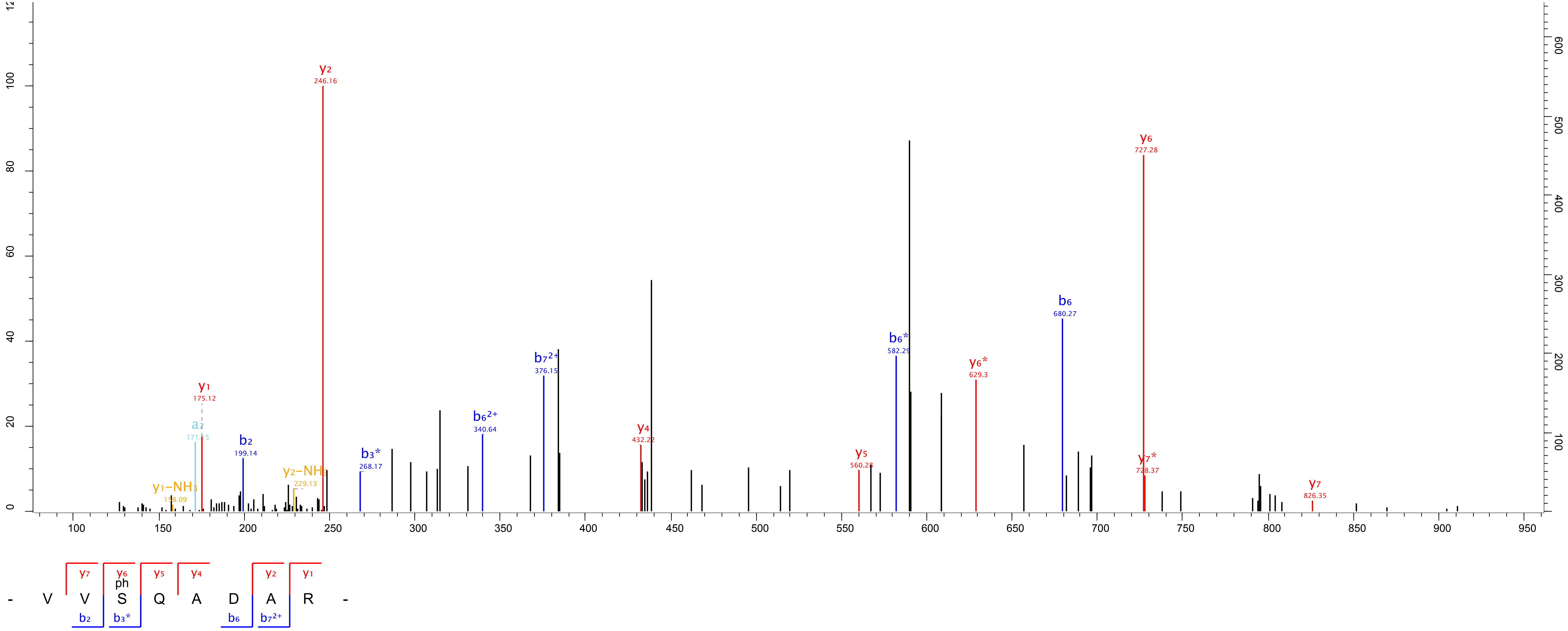

Raw file	Scan	Method	Score	m/z
20130708_PS_Phosphotest_6803wt_FASP03	2081	ITMS; CID	61.34	656.78

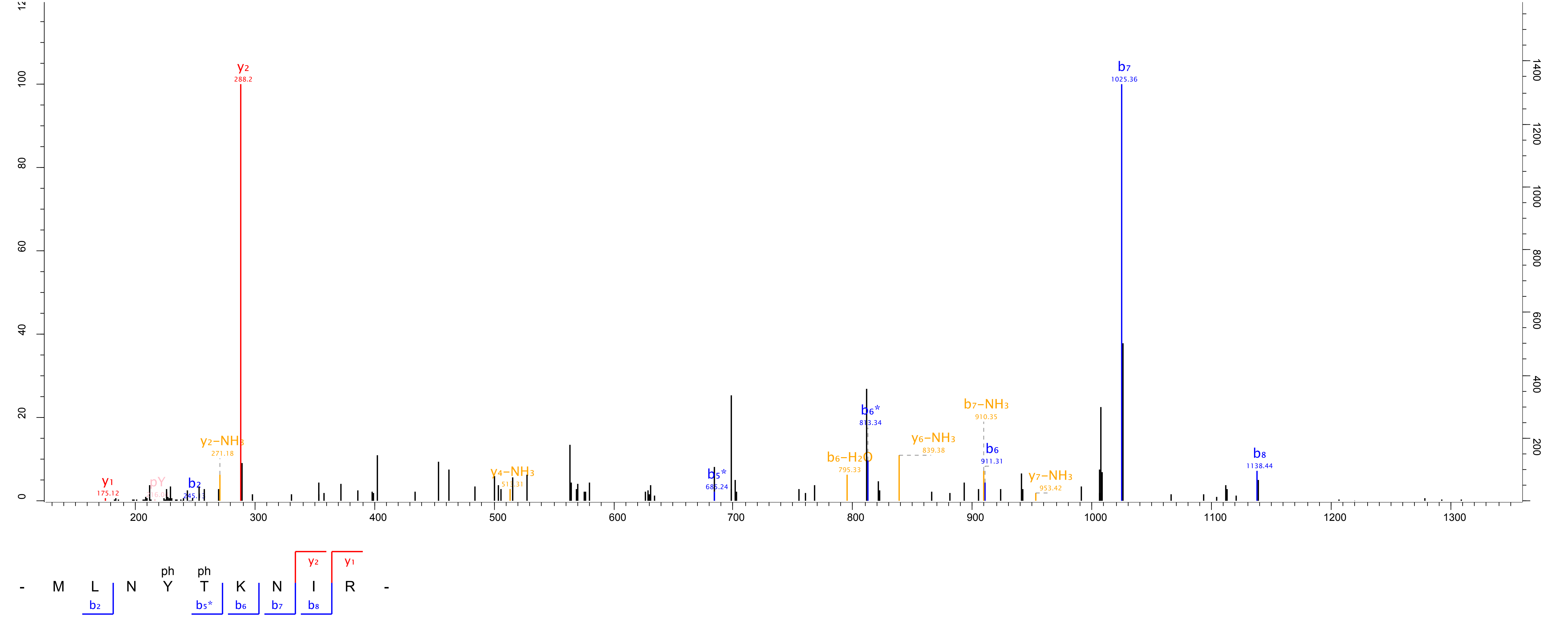

Raw file	Scan	Method	Score	m/z
20130708_PS_Phosphotest_6803wt_YPER01	2170	ITMS; CID	78.96	621.77

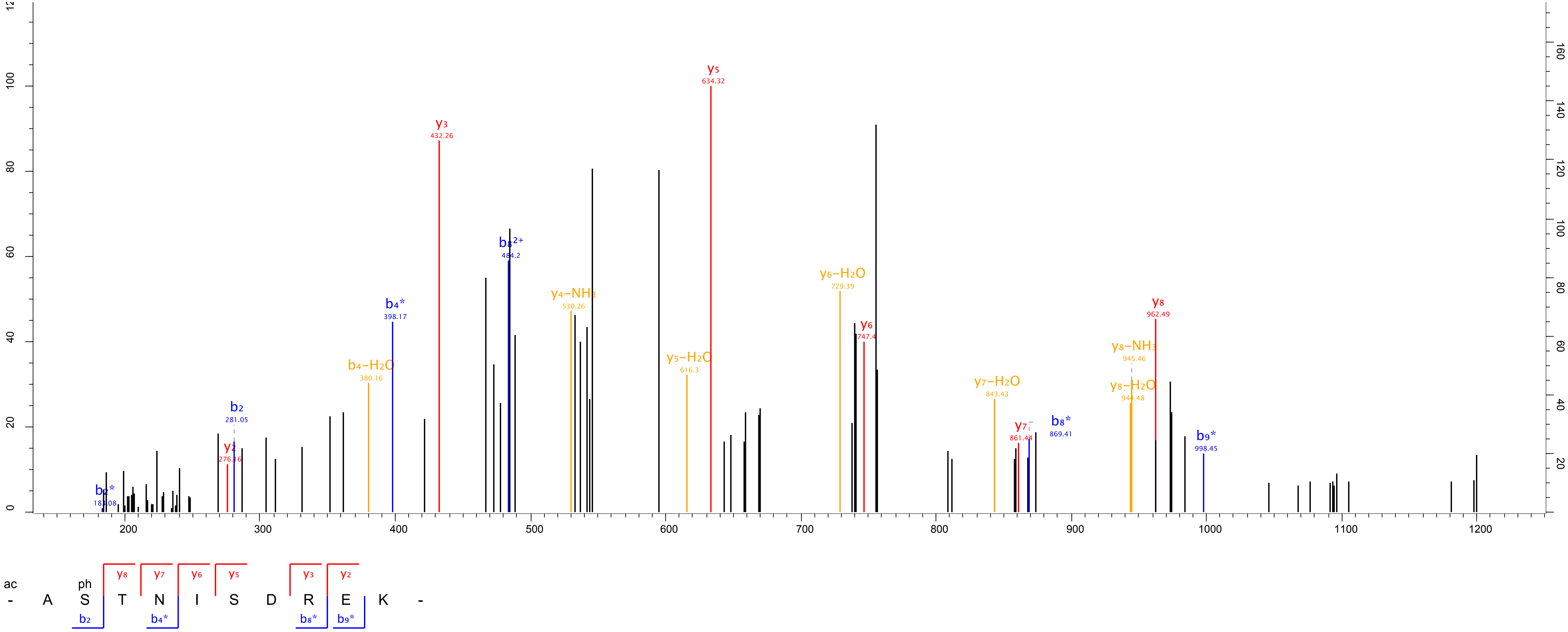

Raw file	Scan	Method	Score	m/z
20130708_PS_Phosphotest_6803wt_SDS01	2481	ITMS; CID	89.36	555.25

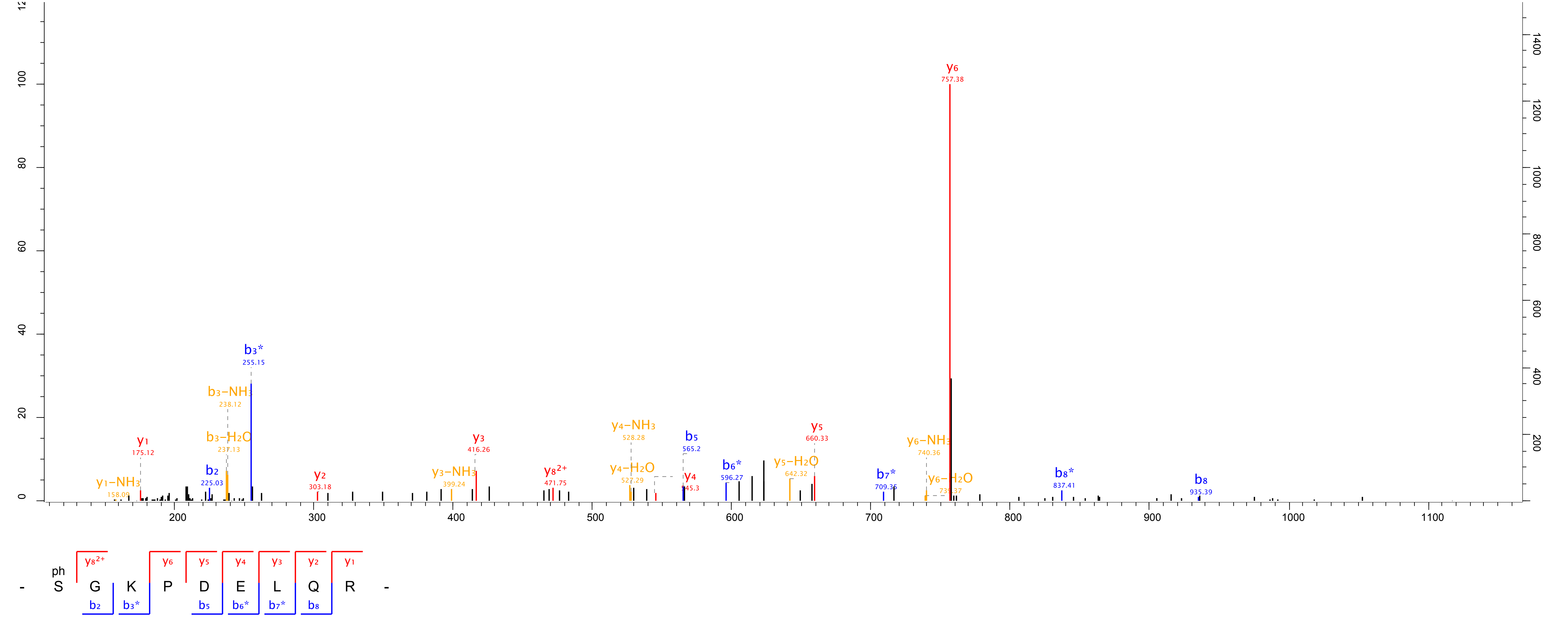

Raw file	Scan	Method	Score	m/z
20130708_PS_Phosphotest_6803wt_SDS01	2702	ITMS; CID	93.67	446.21

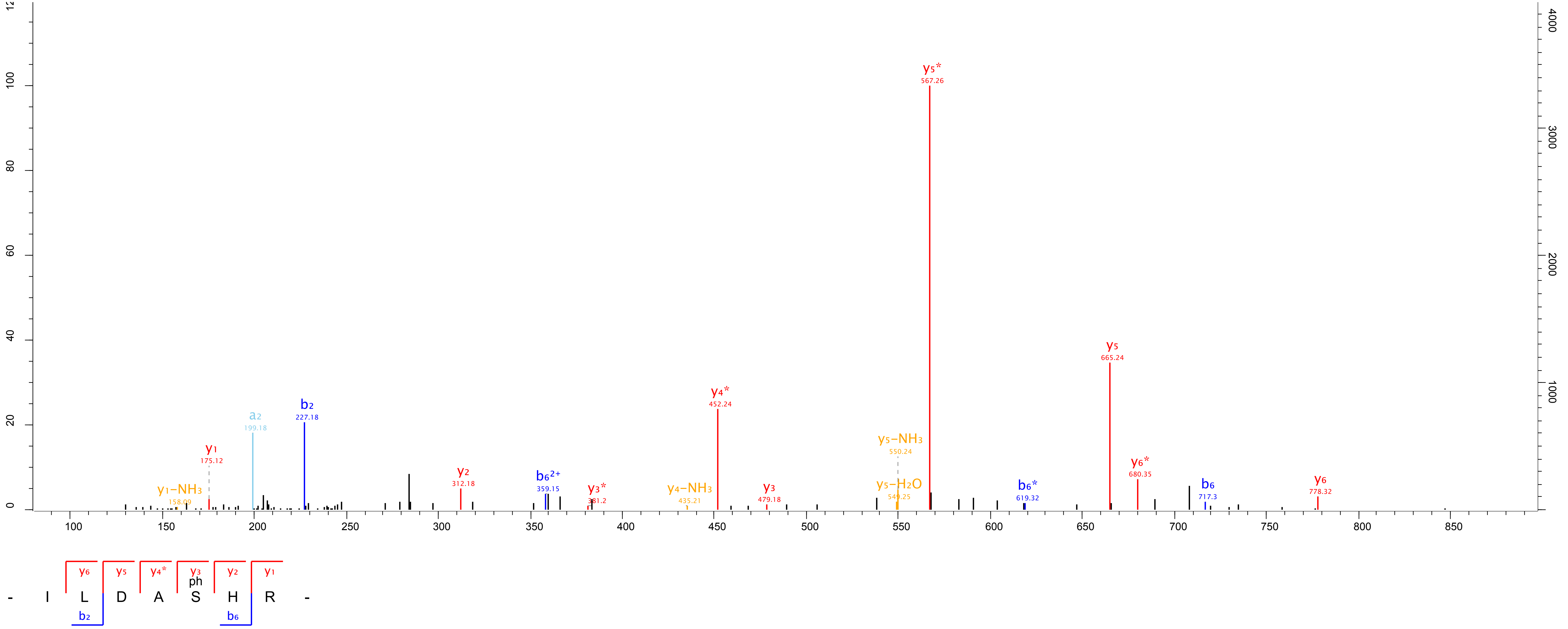

Raw file	Scan	Method	Score	m/z
20130708_PS_Phosphotest_6803wt_SDS01	2863	ITMS; CID	96.21	663.79

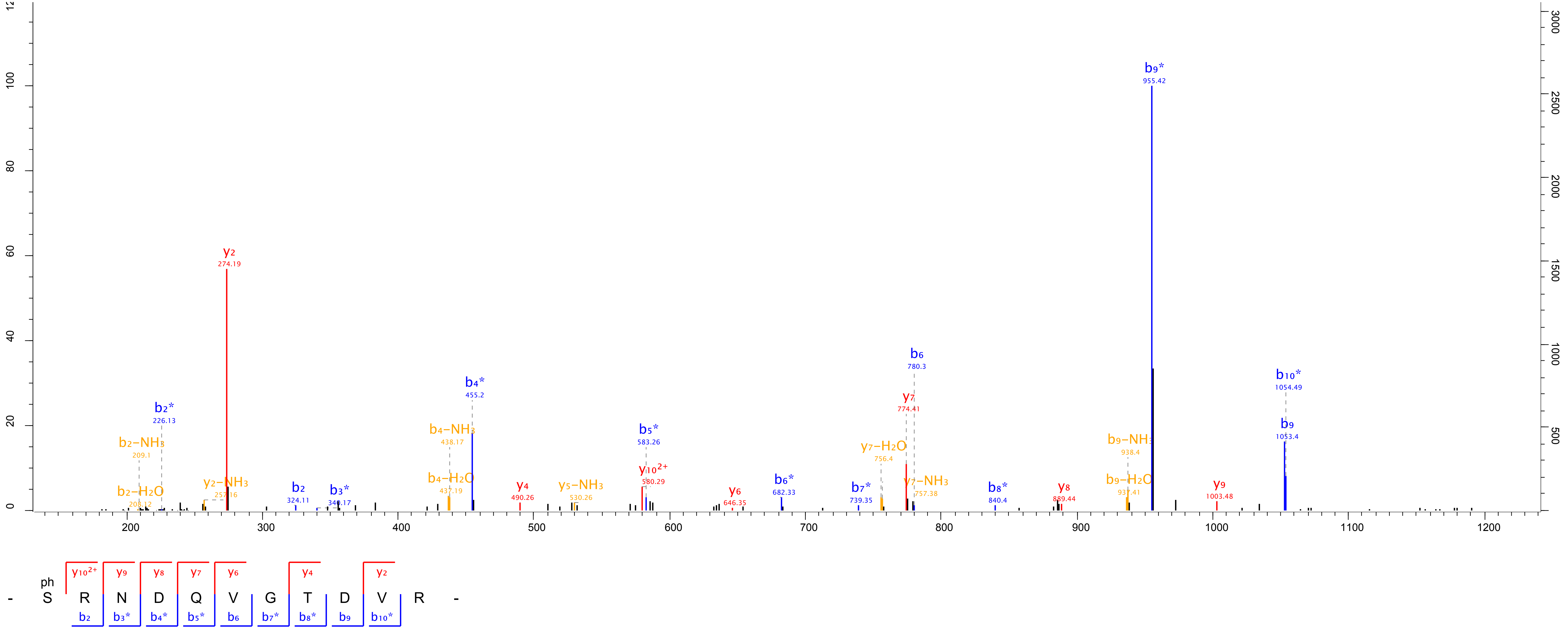

Raw file	Scan	Method	Score	m/z
20130708_PS_Phosphotest_6803wt_SDS01	2867	ITMS; CID	122.52	579.24

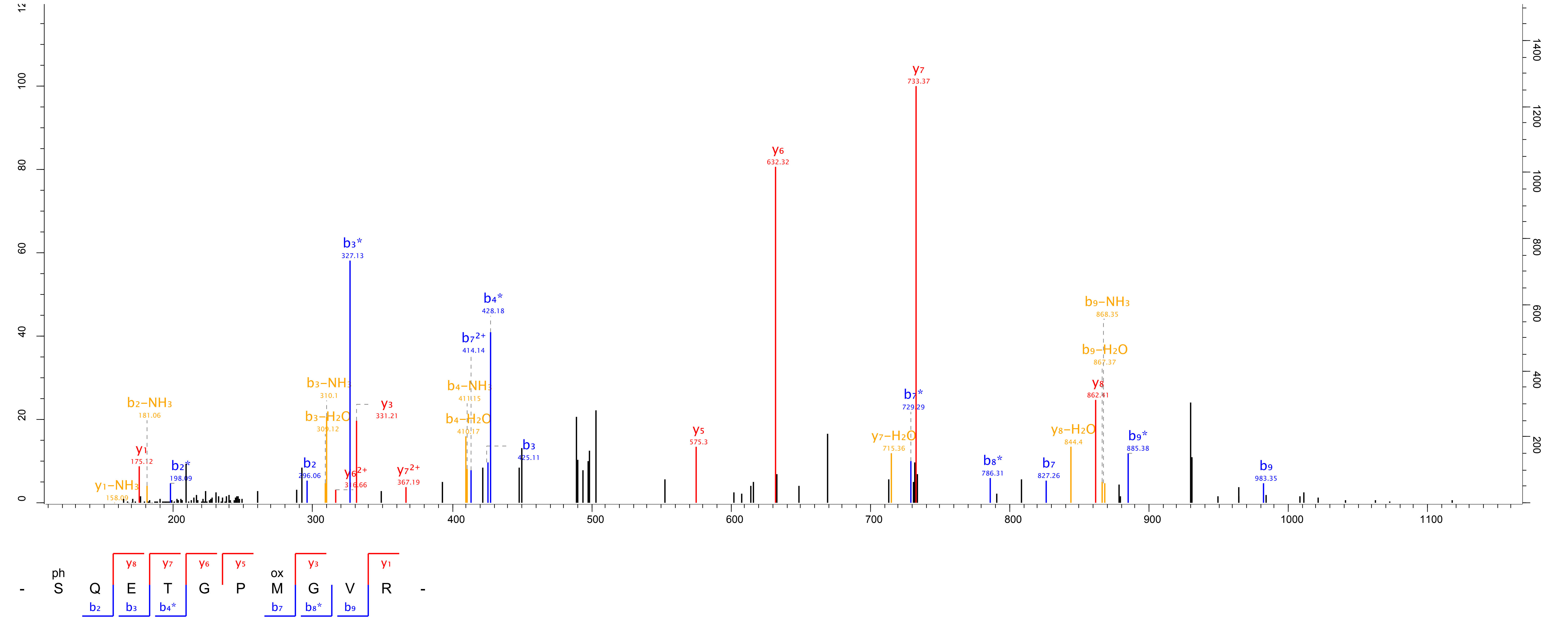

Raw file	Scan	Method	Score	m/z
20130708_PS_Phosphotest_6803wt_SDS01	2912	ITMS; CID	92.94	631.32

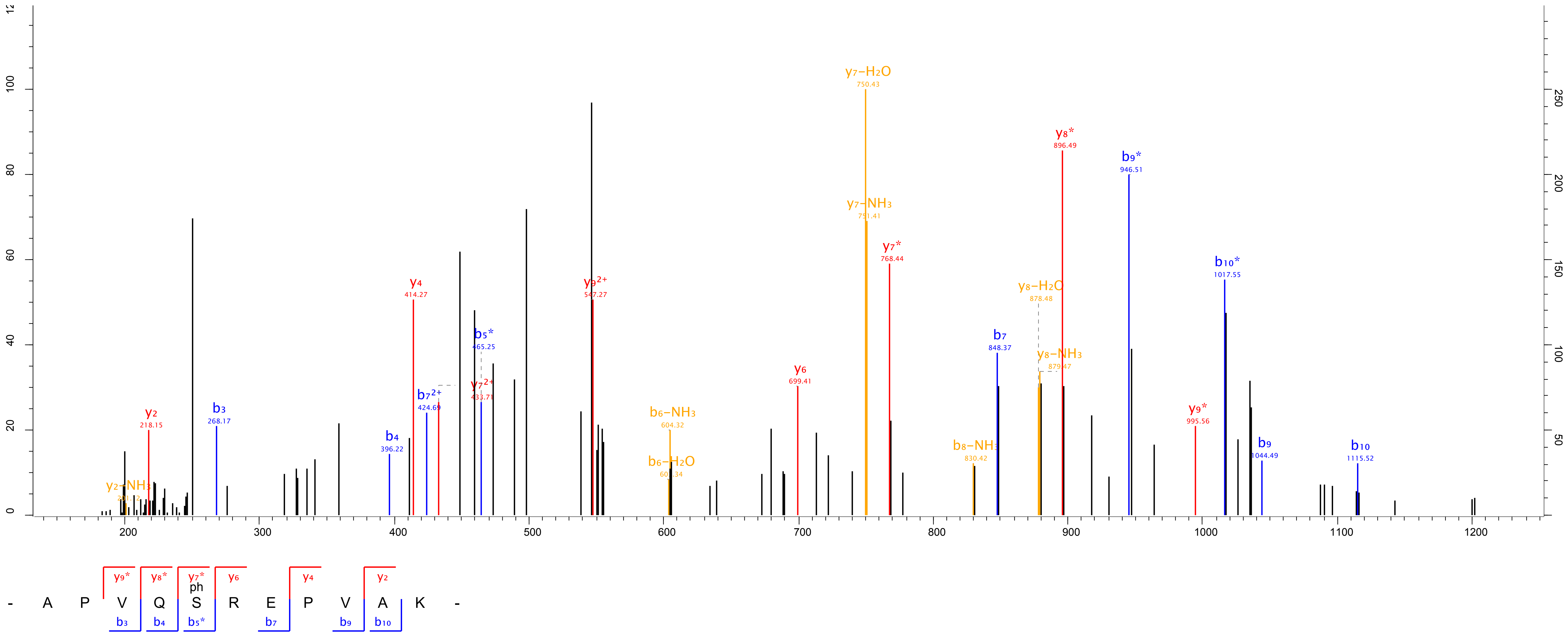

Raw file	Scan	Method	Score	m/z
20130708_PS_Phosphotest_6803wt_SDS01	2943	ITMS; CID	56.87	481.54

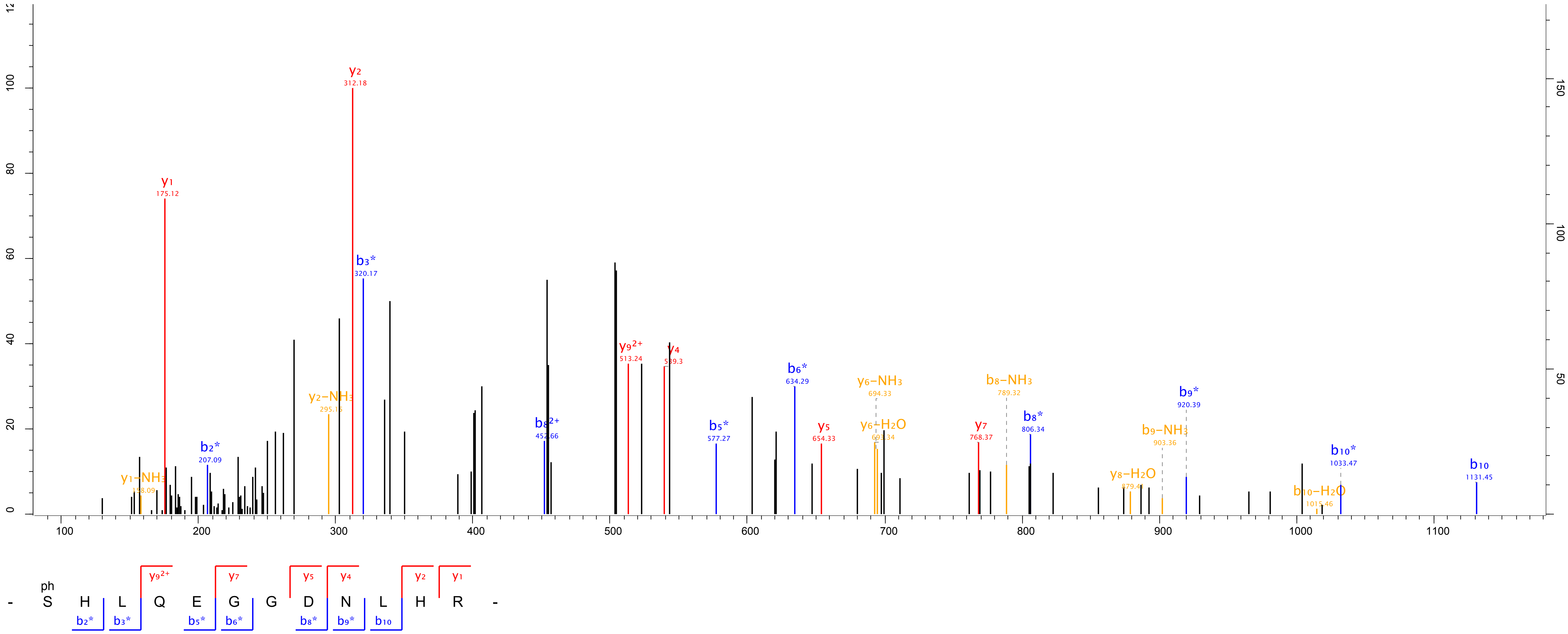

Raw file	Scan	Method	Score	m/z
20130708_PS_Phosphotest_6803wt_SDS01	3210	ITMS; CID	143.24	537.23

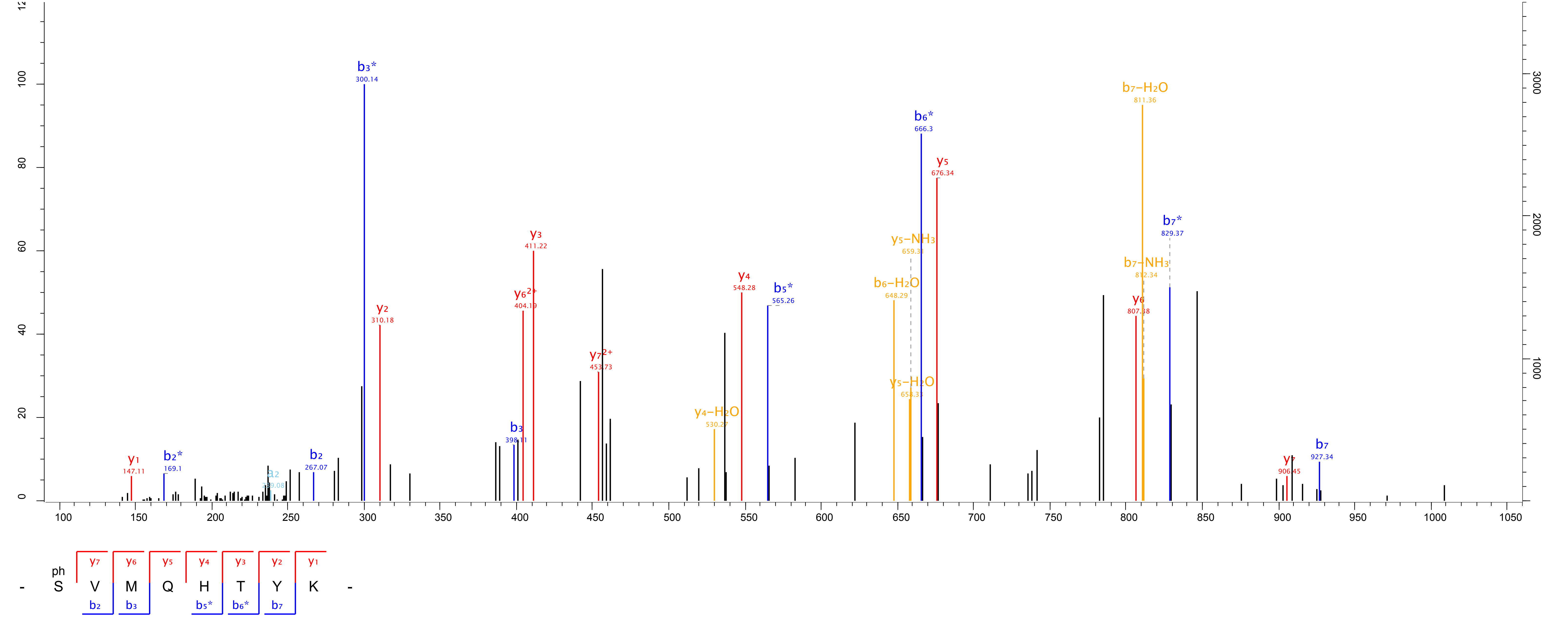

Raw file	Scan	Method	Score	m/z
20130708_PS_Phosphotest_6803wt_YPER01	3274	ITMS; CID	115.2	646.29

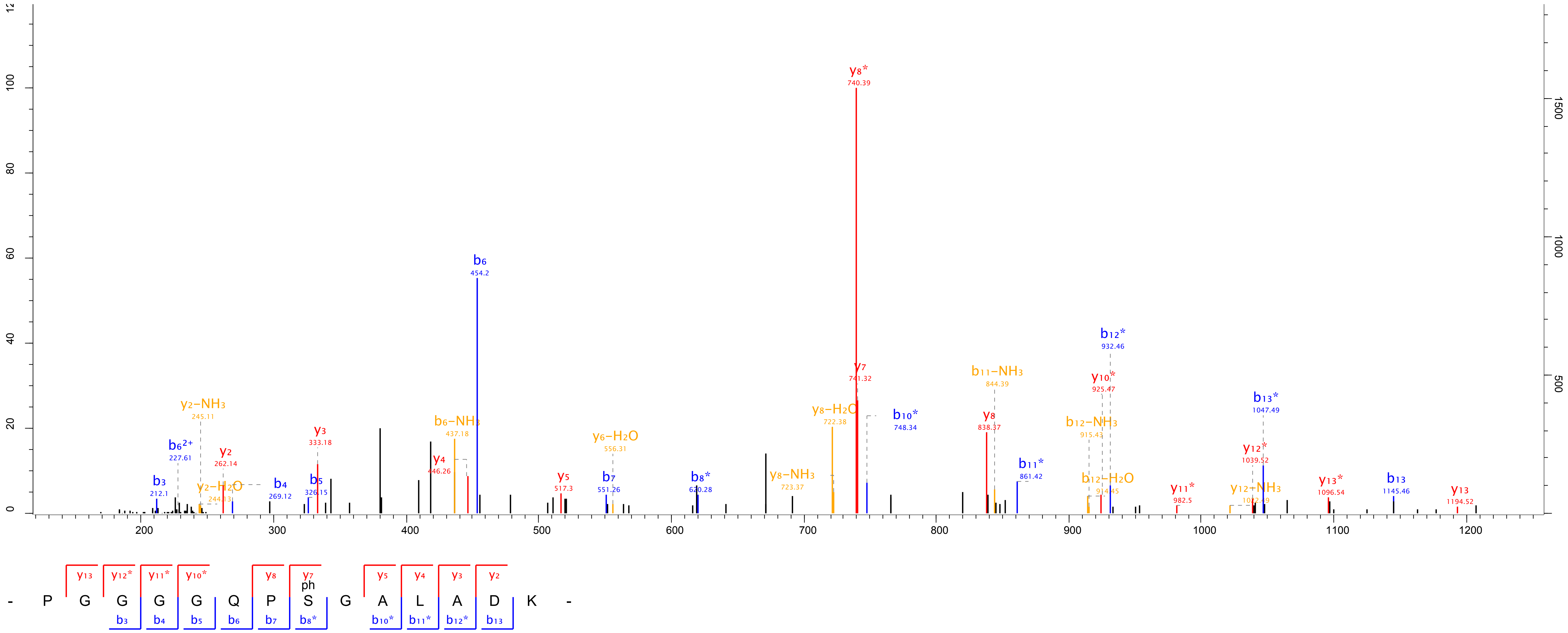

Raw file	Scan	Method	Score	m/z
20130708_PS_Phosphotest_6803wt_SDS01	3287	ITMS; CID	125.23	518.74

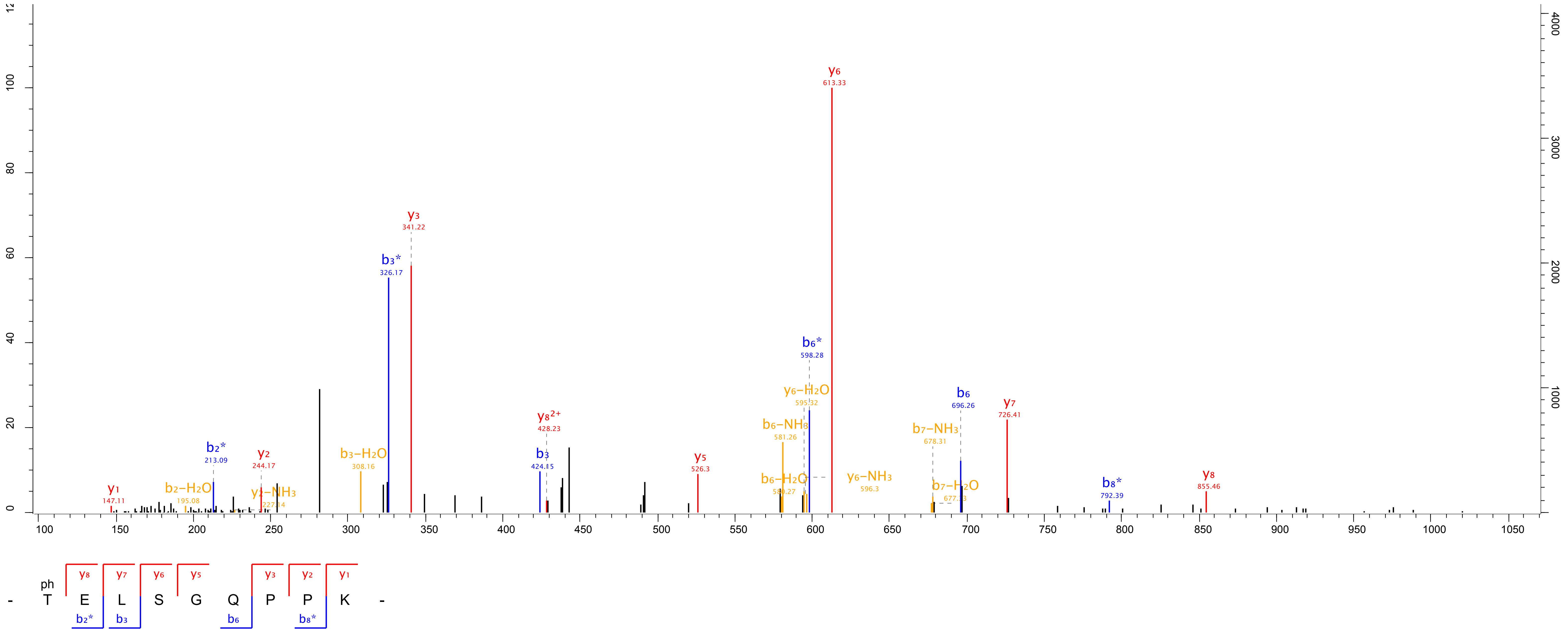

Raw file	Scan	Method	Score	m/z
20130708_PS_Phosphotest_6803wt_SDS01	3289	ITMS; CID	149.93	634.59

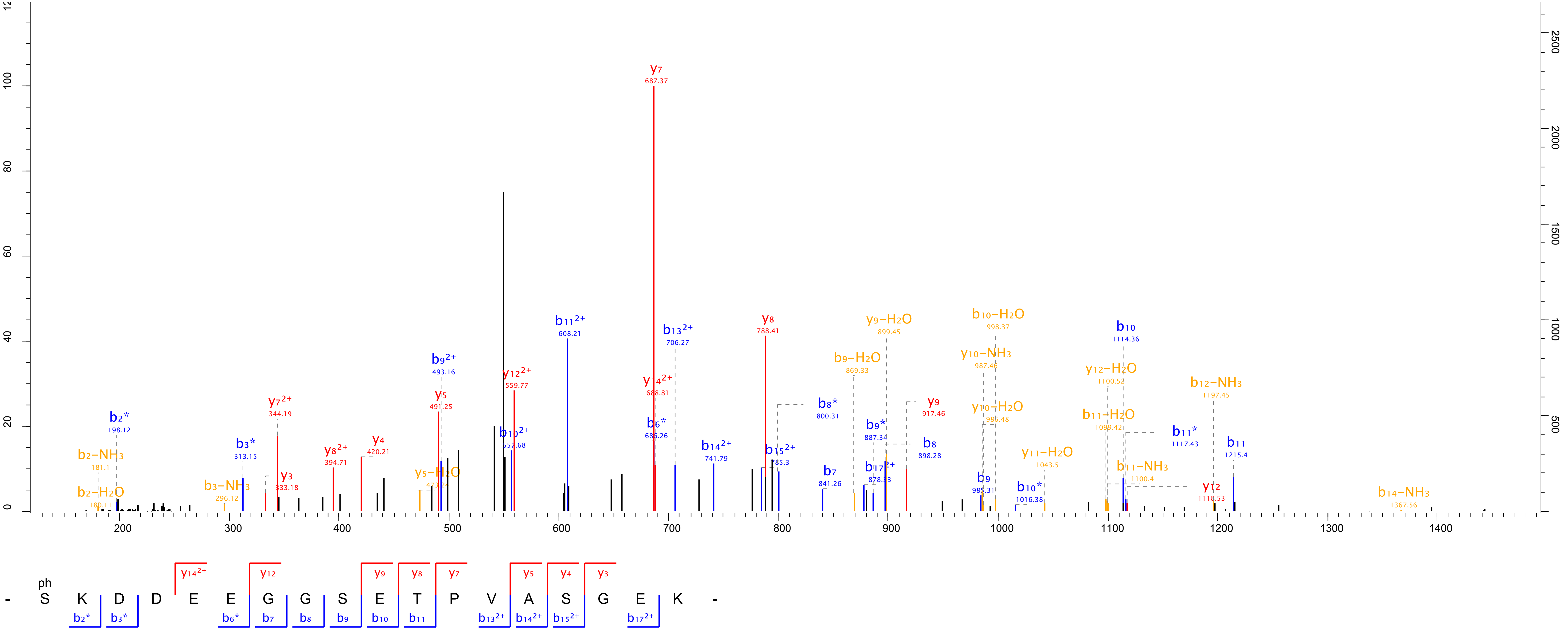

Raw file	Scan	Method	Score	m/z
20130708_PS_Phosphotest_6803wt_YPER01	3292	ITMS; CID	84.82	483.26

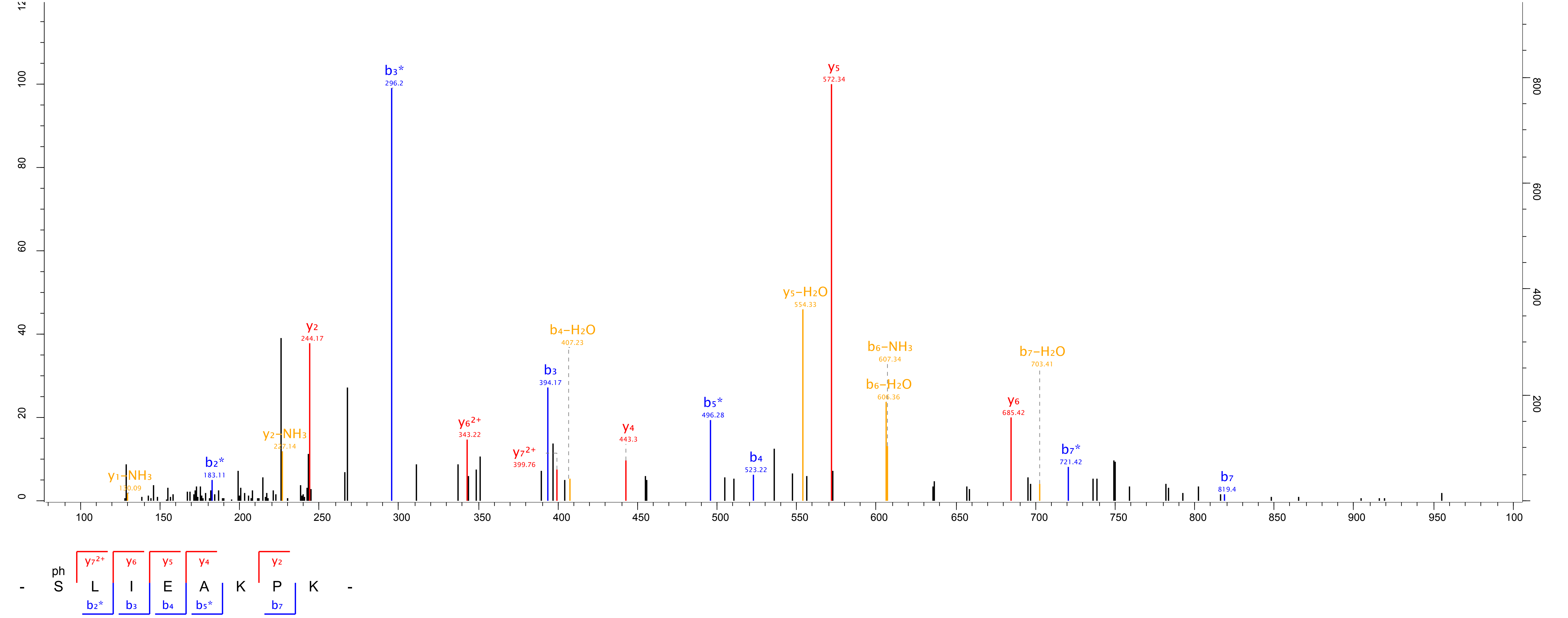

Raw file	Scan	Method	Score	m/z
20130708_PS_Phosphotest_6803wt_SDS01	3305	ITMS; CID	198.48	599.28

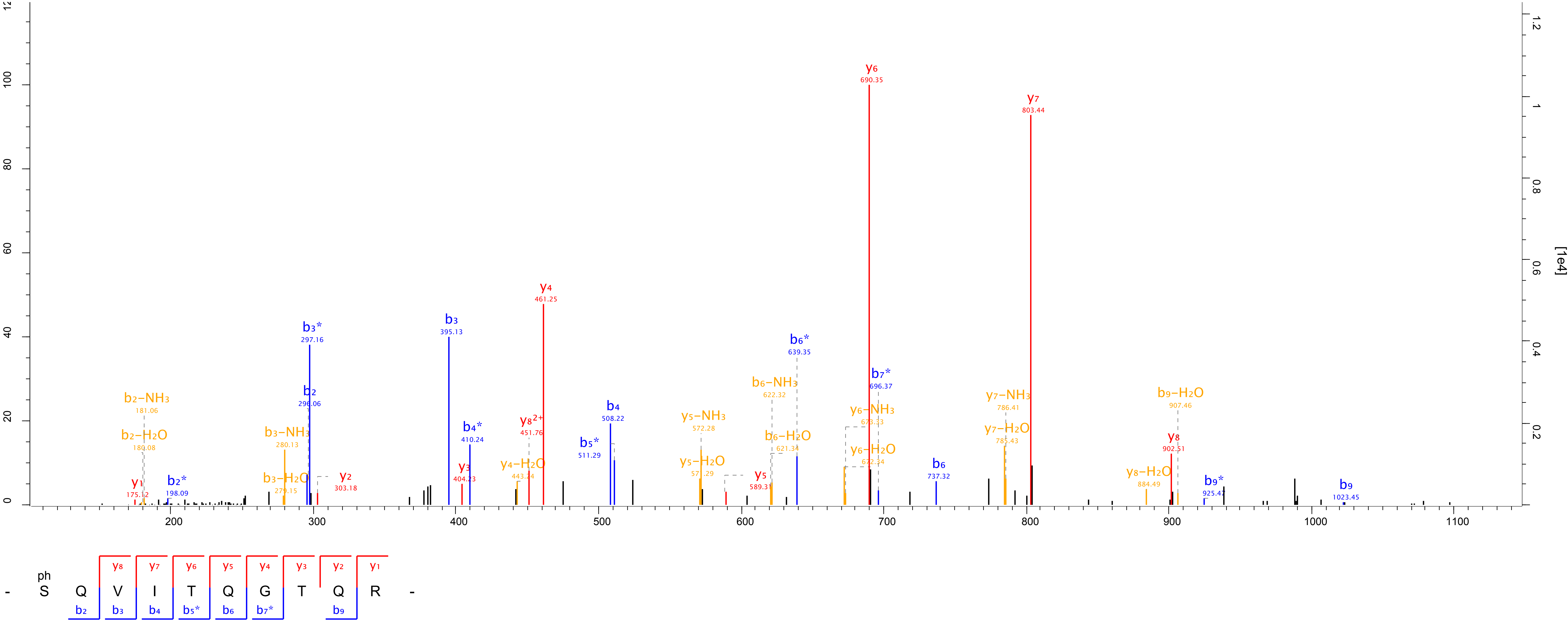

Raw file	Scan	Method	Score	m/z
20130708_PS_Phosphotest_6803wt_SDS01	3311	ITMS; CID	83.08	477.72

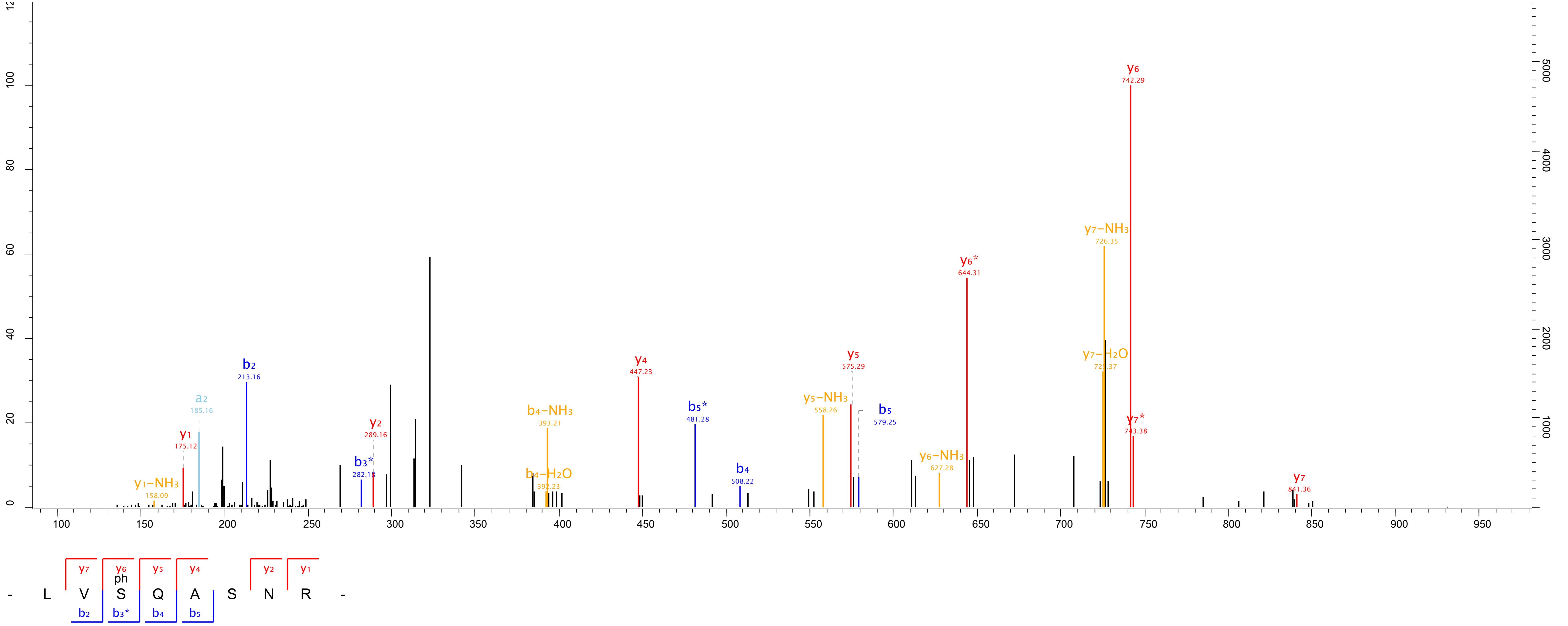

Raw file	Scan	Method	Score	m/z
20130708_PS_Phosphotest_6803wt_SDS05	3410	ITMS; CID	75.22	1022.46

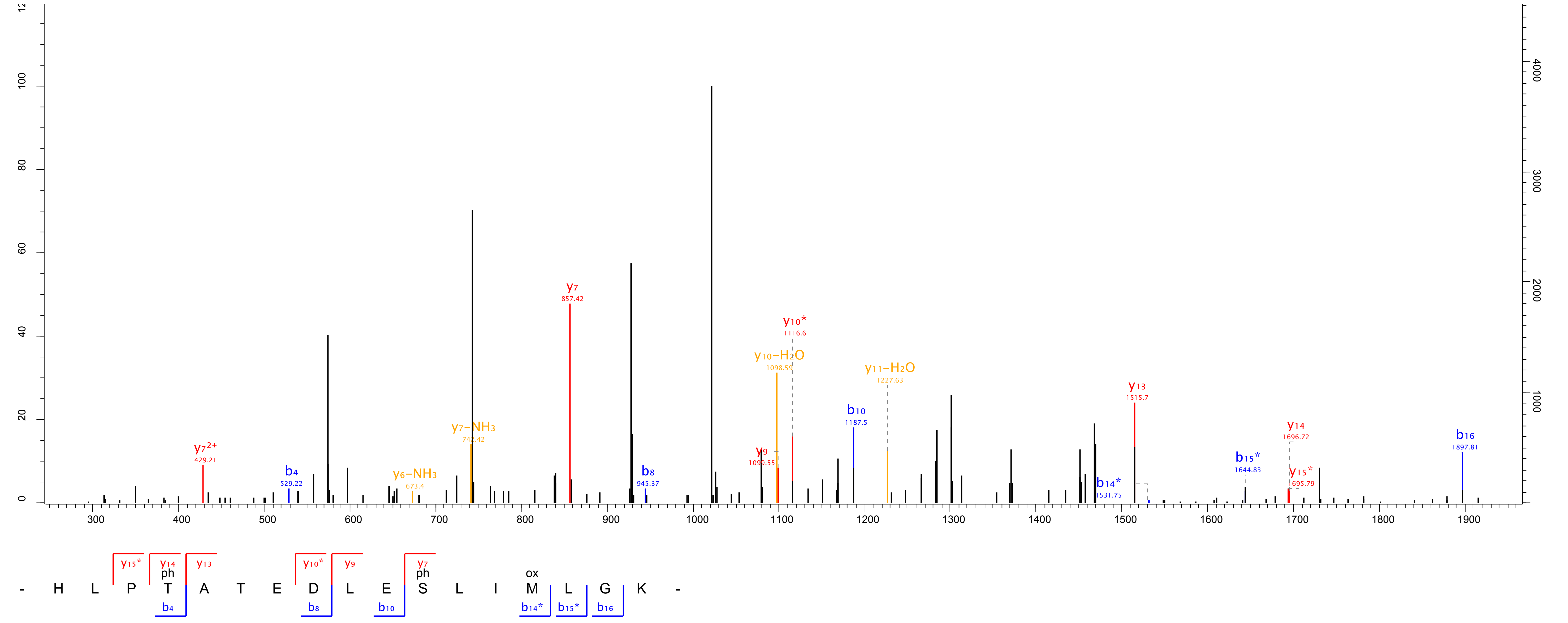

Raw file	Scan	Method	Score	m/z
20130708_PS_Phosphotest_6803wt_SDS01	3422	ITMS; CID	109.6	593.29

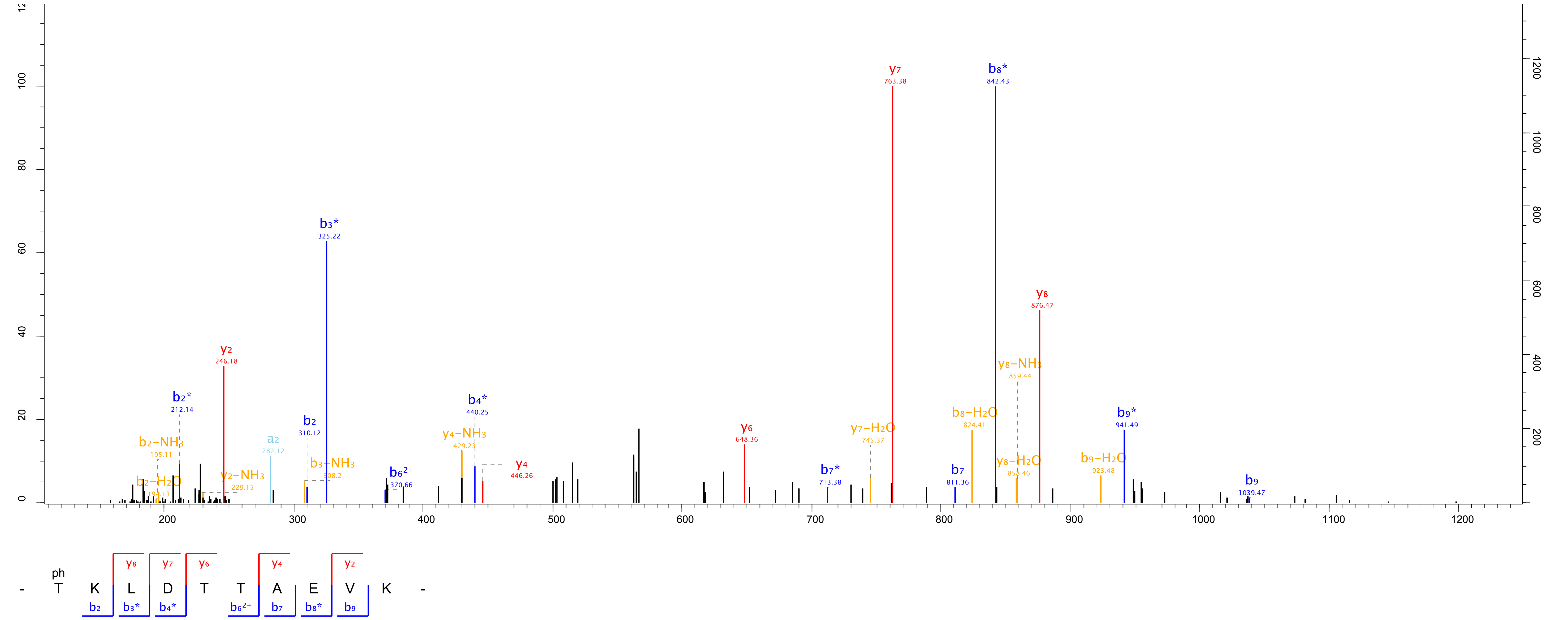

Raw file	Scan	Method	Score	m/z
20130708_PS_Phosphotest_6803wt_FASP01	3434	ITMS; CID	107.69	603.31

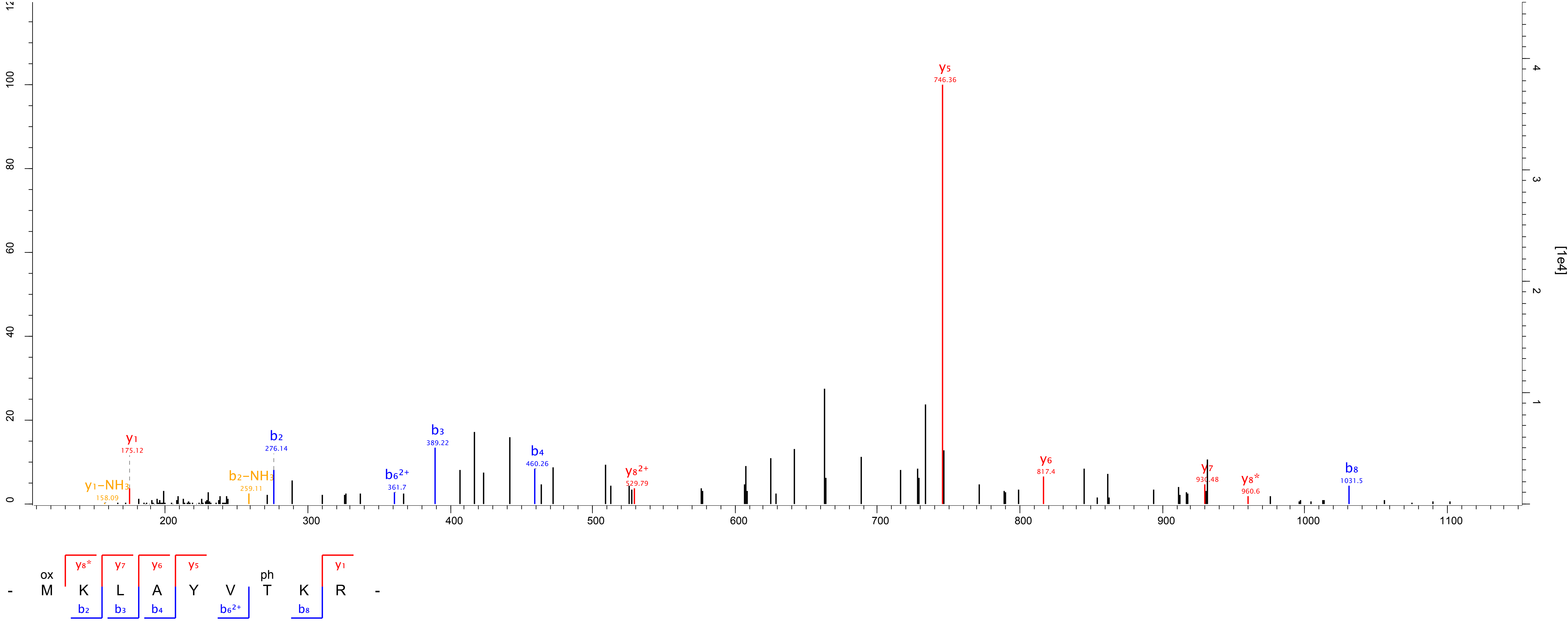

Raw file	Scan	Method	Score	m/z
20130708_PS_Phosphotest_6803wt_SDS02	3576	ITMS; CID	40.37	408.2

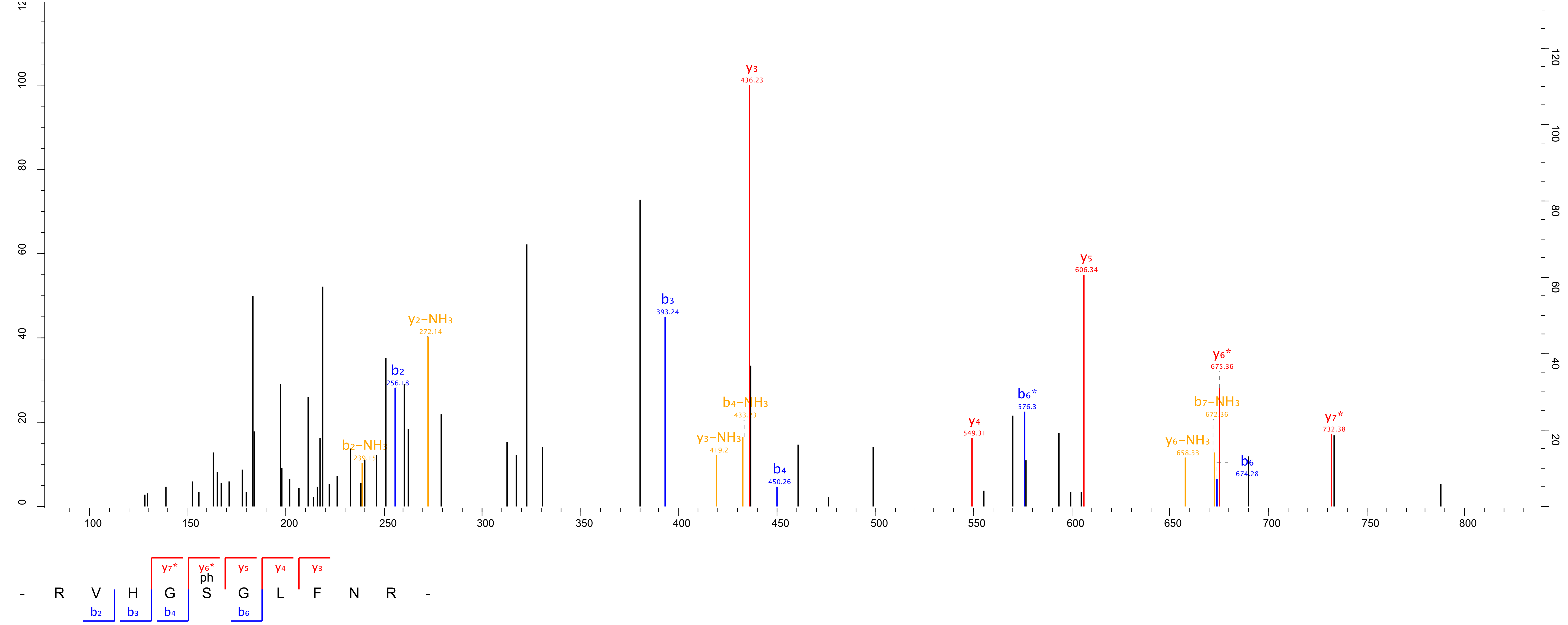

Raw file	Scan	Method	Score	m/z
20130708_PS_Phosphotest_6803wt_YPER01	3587	ITMS; CID	67.76	1022.46

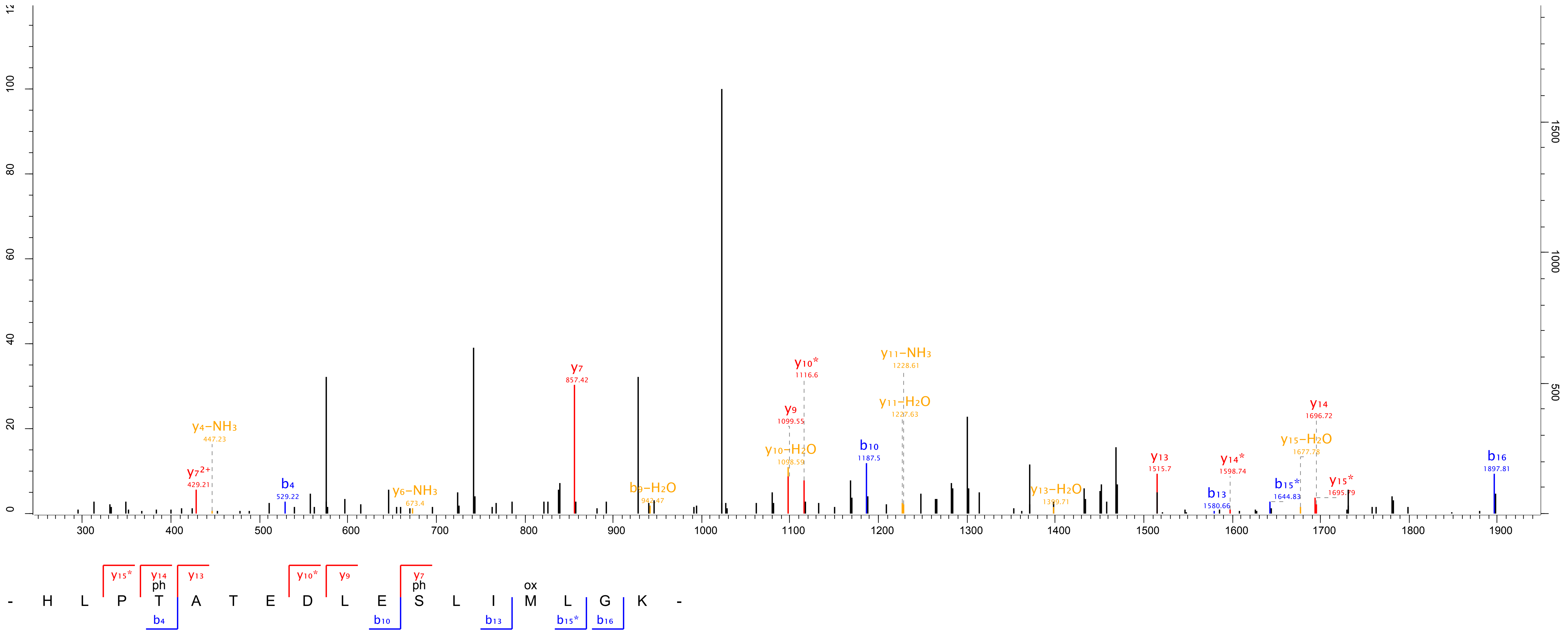

Raw file	Scan	Method	Score	m/z
20130708_PS_Phosphotest_6803wt_YPER01	3617	ITMS; CID	126.5	538.73

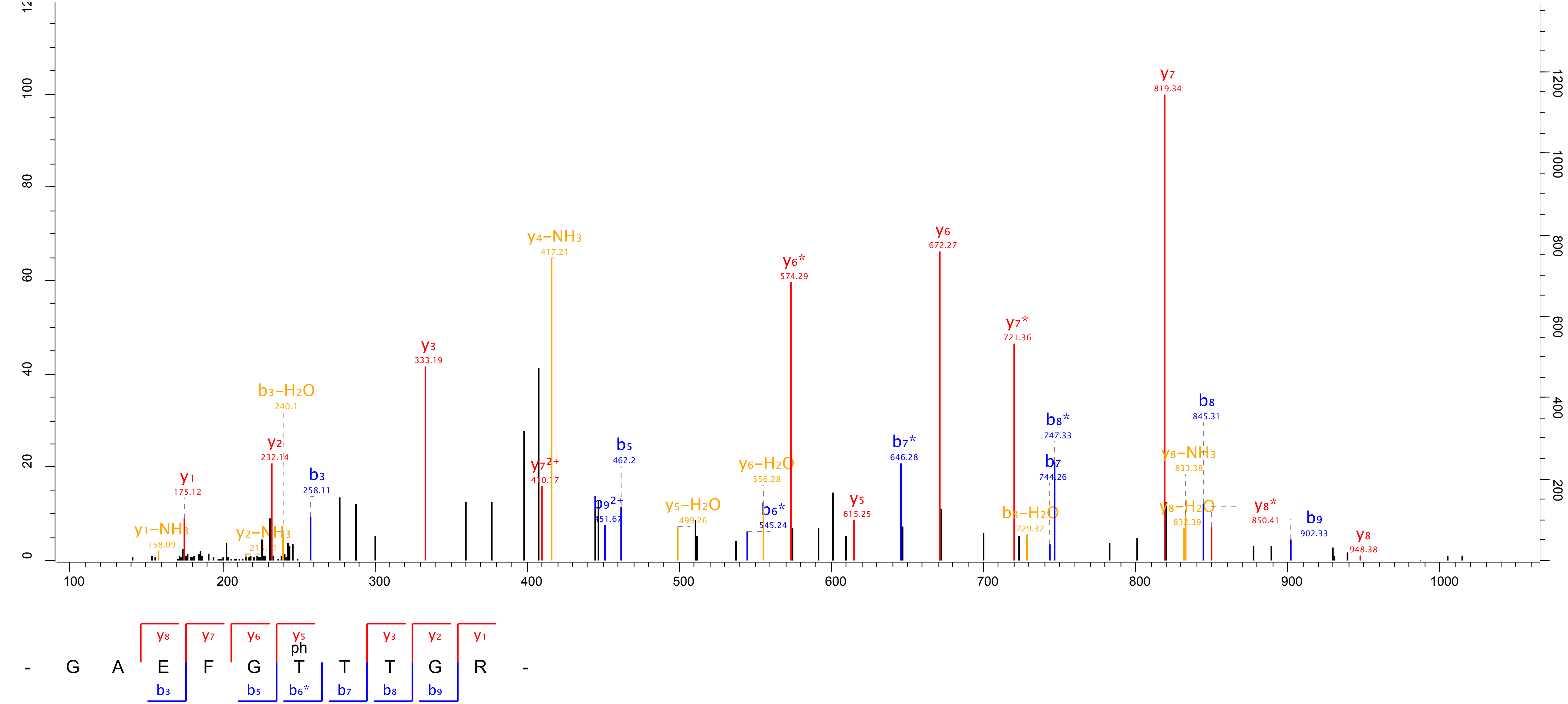

Raw file	Scan	Method	Score	m/z
20130708_PS_Phosphotest_6803wt_YPER01	3620	ITMS; CID	234.87	625.27

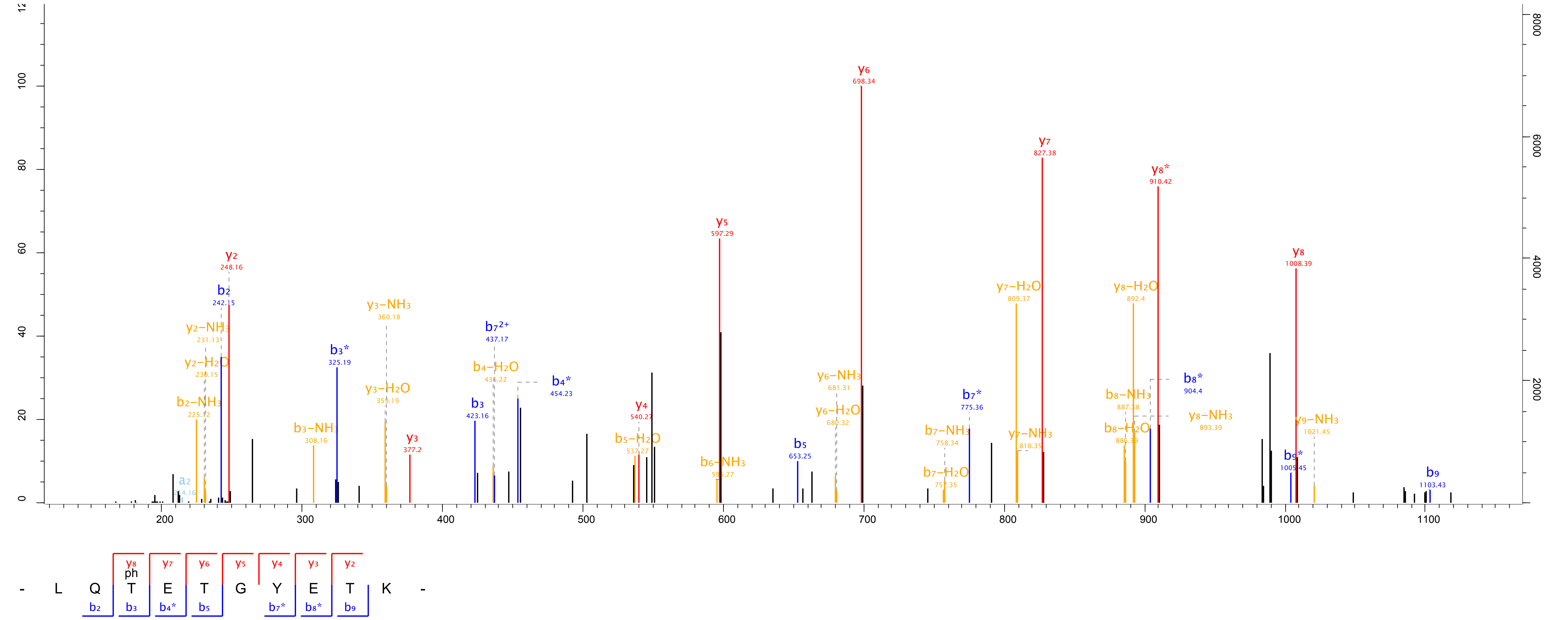

Raw file	Scan	Method	Score	m/z
20130708_PS_Phosphotest_6803wt_SDS01	3632	ITMS; CID	132.31	581.77

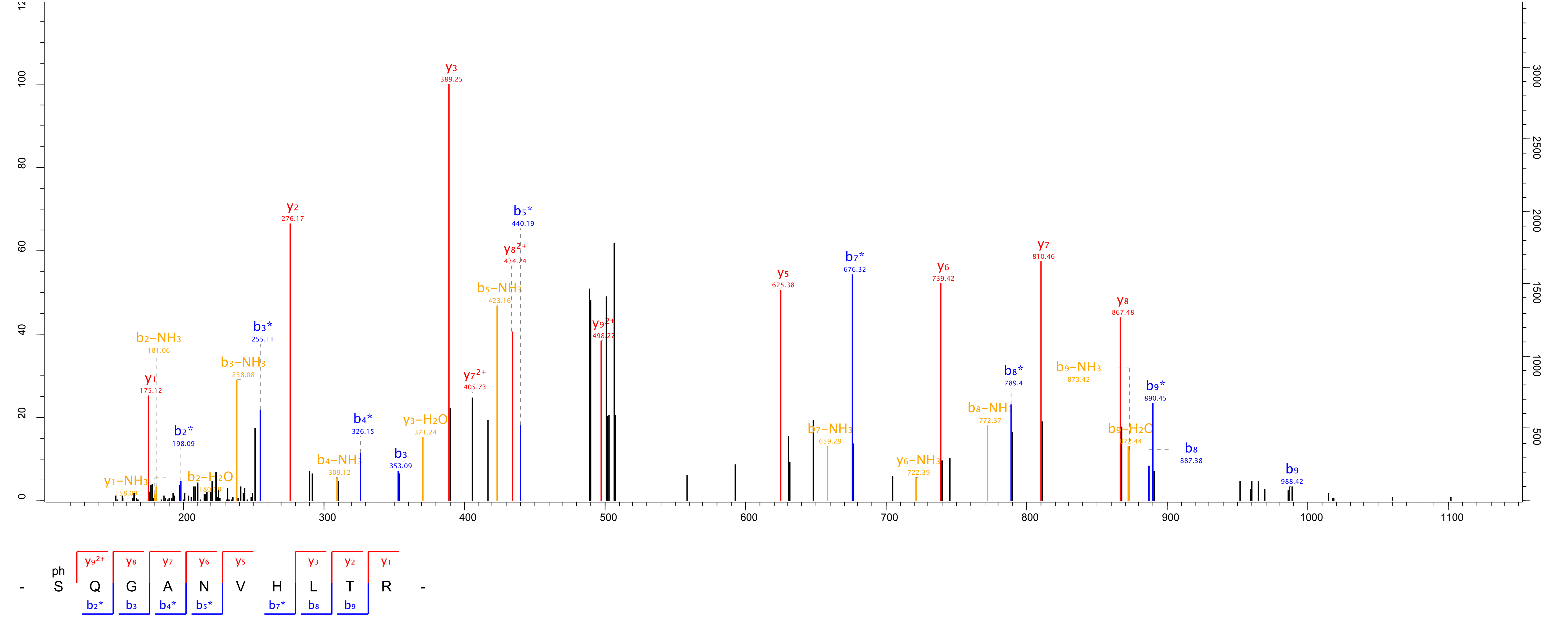

Raw file	Scan	Method	Score	m/z
20130708_PS_Phosphotest_6803wt_SDS01	3685	ITMS; CID	145.61	627.79

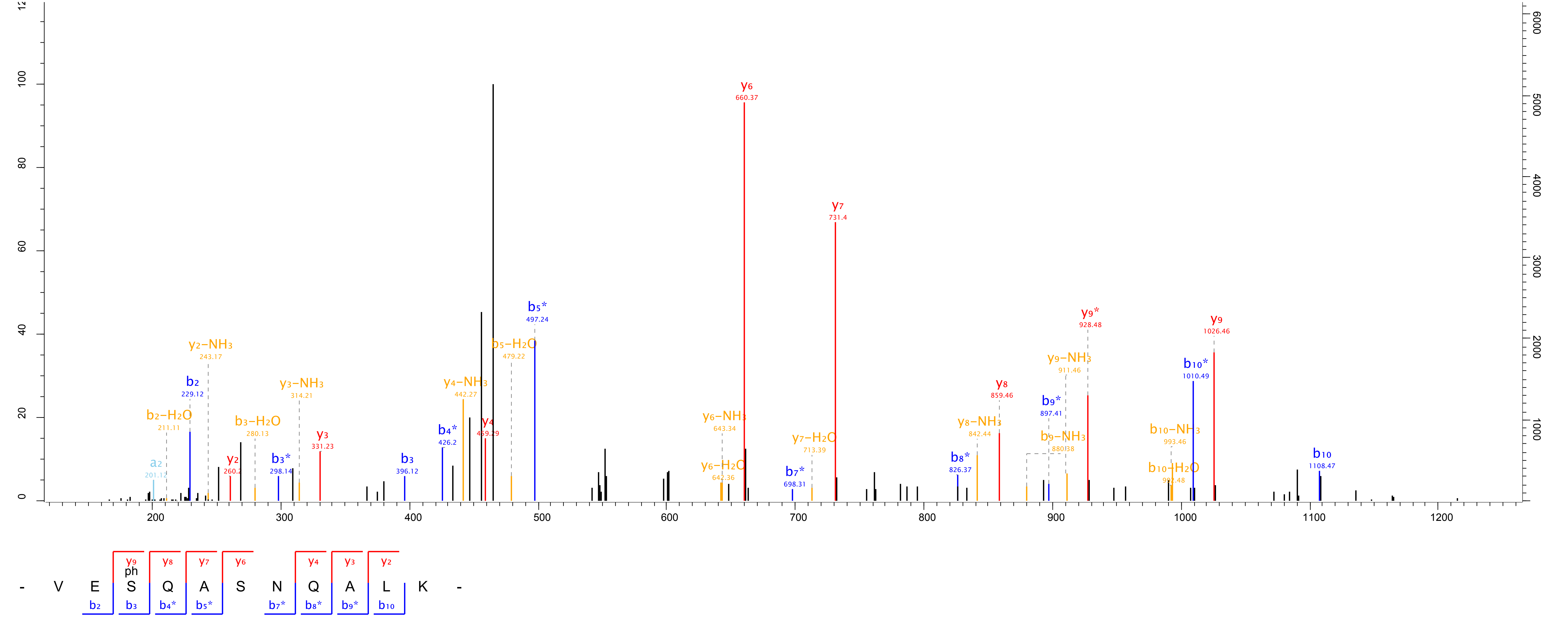

Raw file	Scan	Method	Score	m/z
20130708_PS_Phosphotest_6803wt_SDS01	3708	ITMS; CID	123.51	523.24

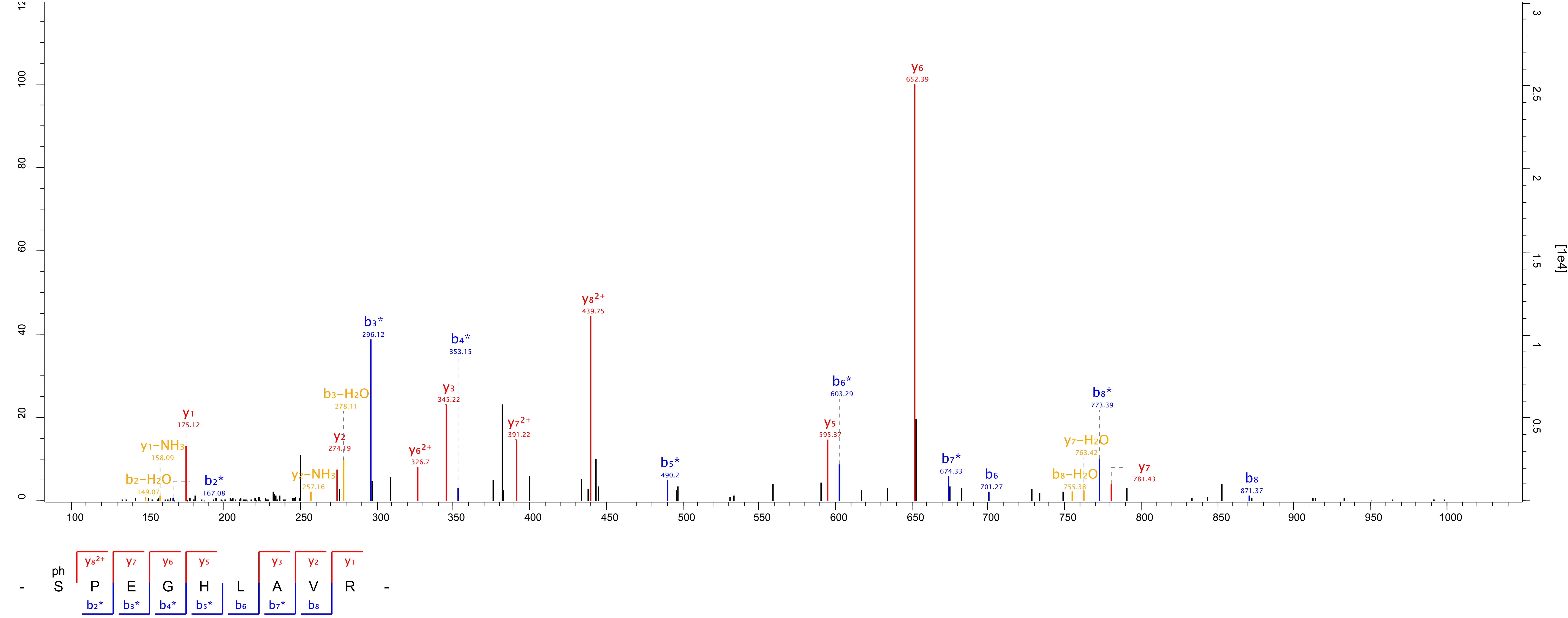

Raw file	Scan	Method	Score	m/z
20130708_PS_Phosphotest_6803wt_SDS01	3722	ITMS; CID	229.02	643.26

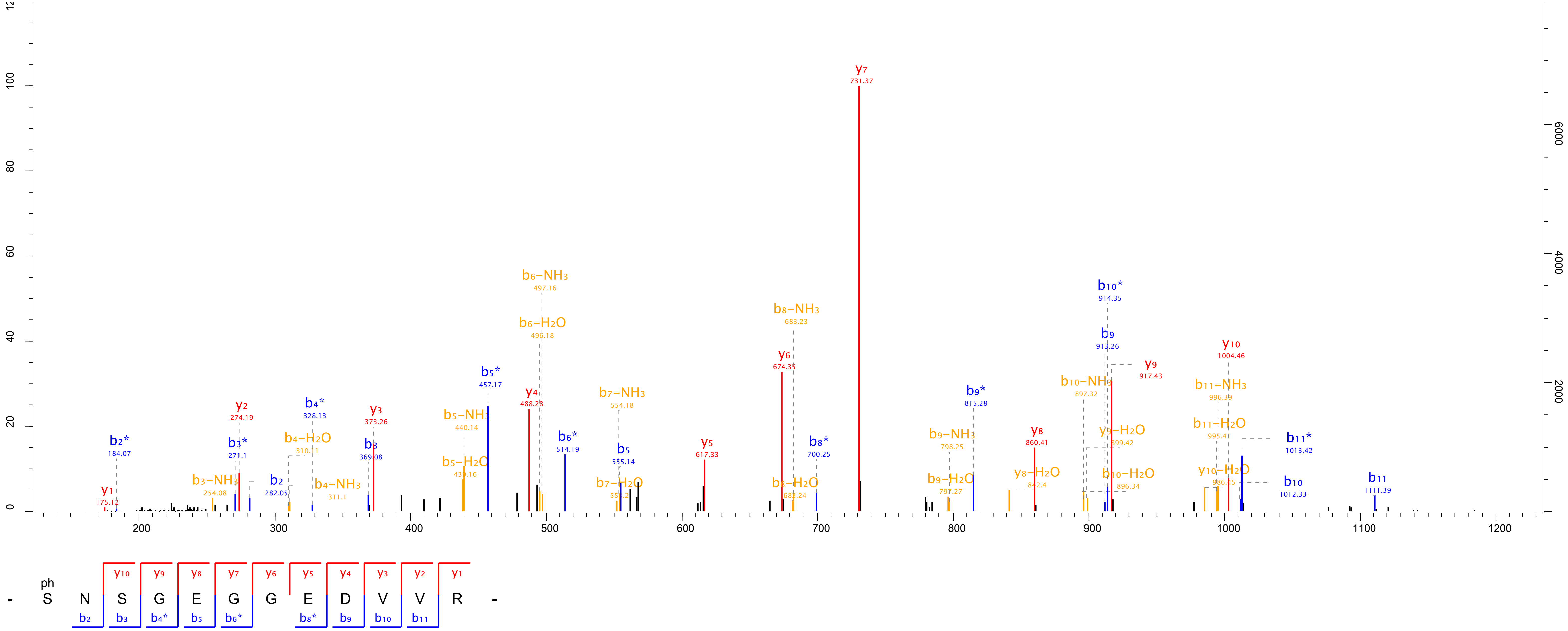

Raw file	Scan	Method	Score	m/z
20130708_PS_Phosphotest_6803wt_SDS01	3747	ITMS; CID	140.68	591.28

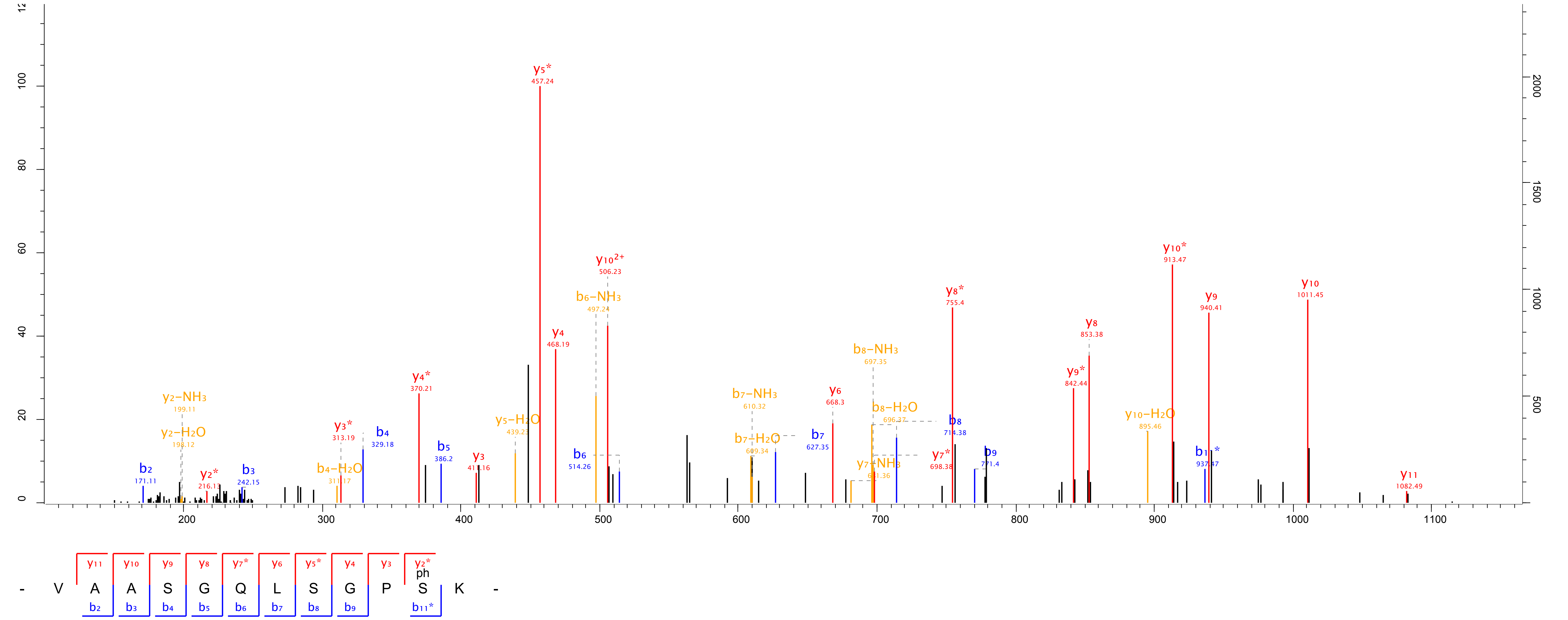

Raw file	Scan	Method	Score	m/z
20130708_PS_Phosphotest_6803wt_YPER01	3795	ITMS; CID	77.19	520.24

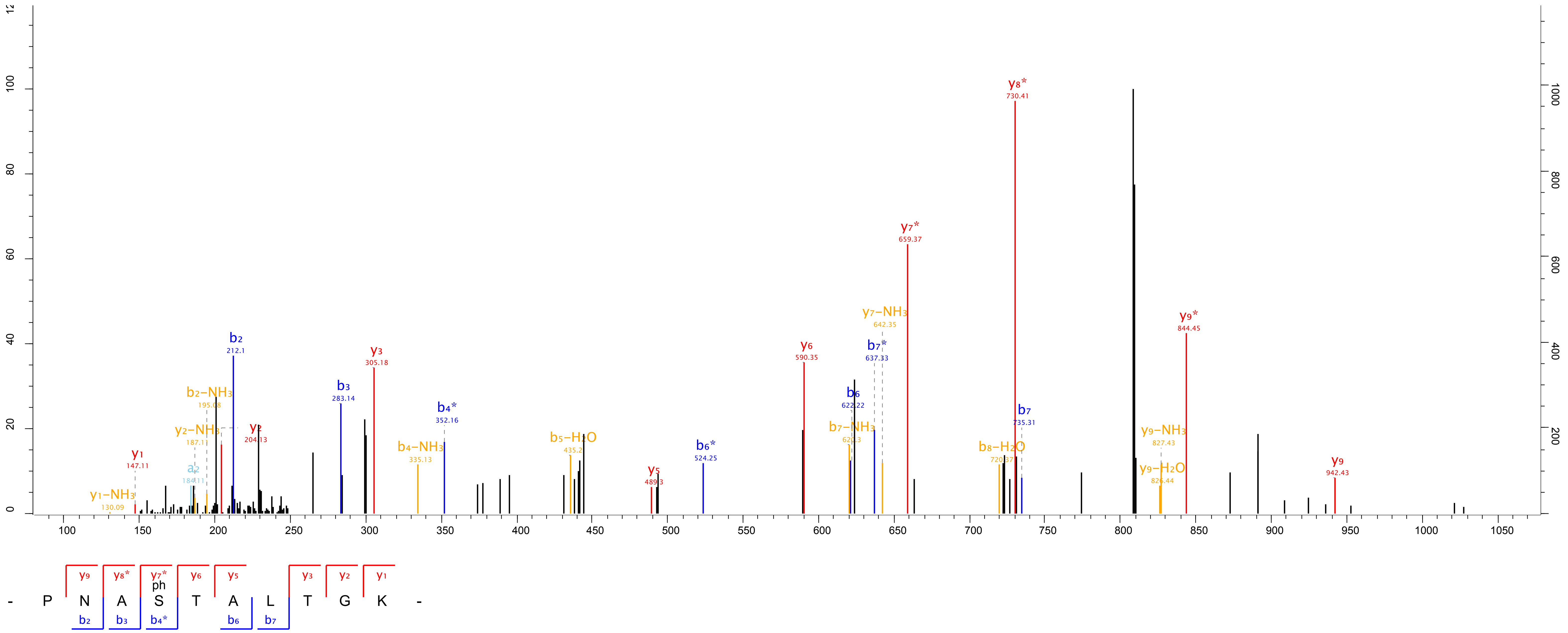

Raw file	Scan	Method	Score	m/z
20130708_PS_Phosphotest_6803wt_YPER01	3888	ITMS; CID	113.52	627.3

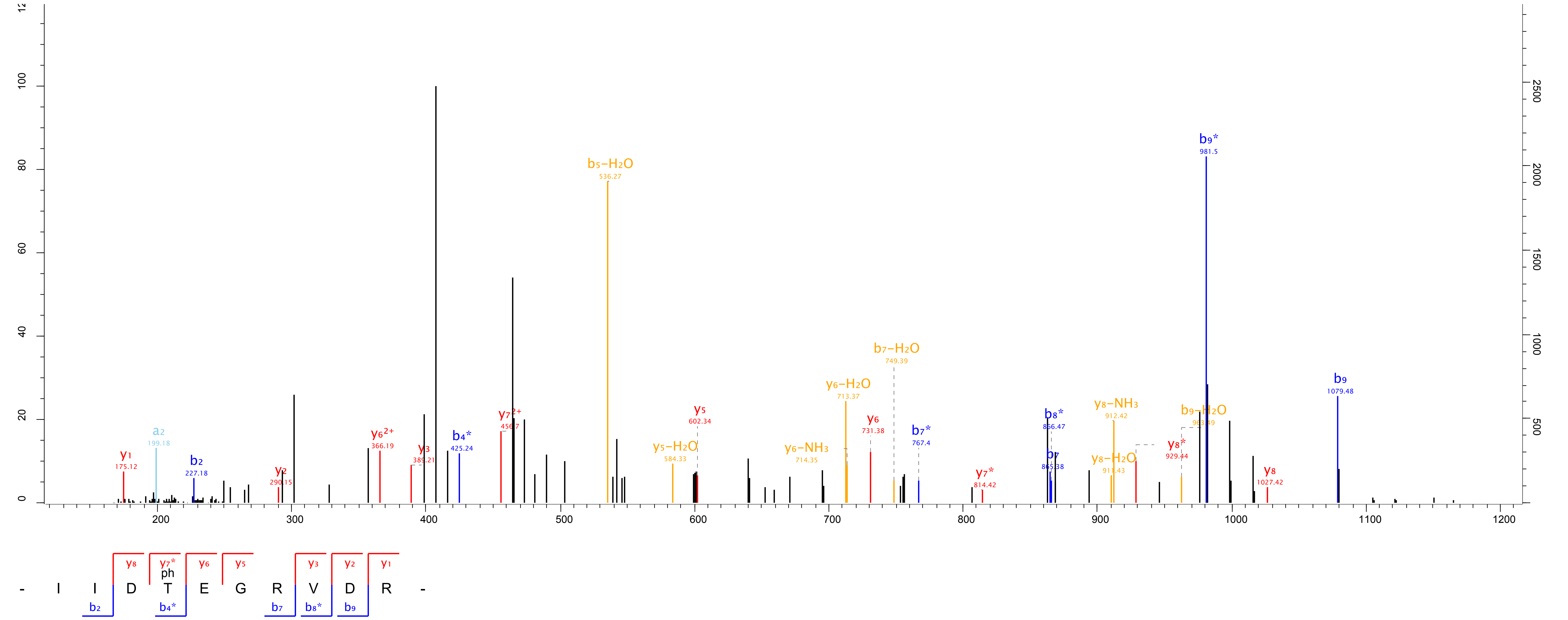

Raw file	Scan	Method	Score	m/z
20130708_PS_Phosphotest_6803wt_SDS01	3900	ITMS; CID	89.36	613.76

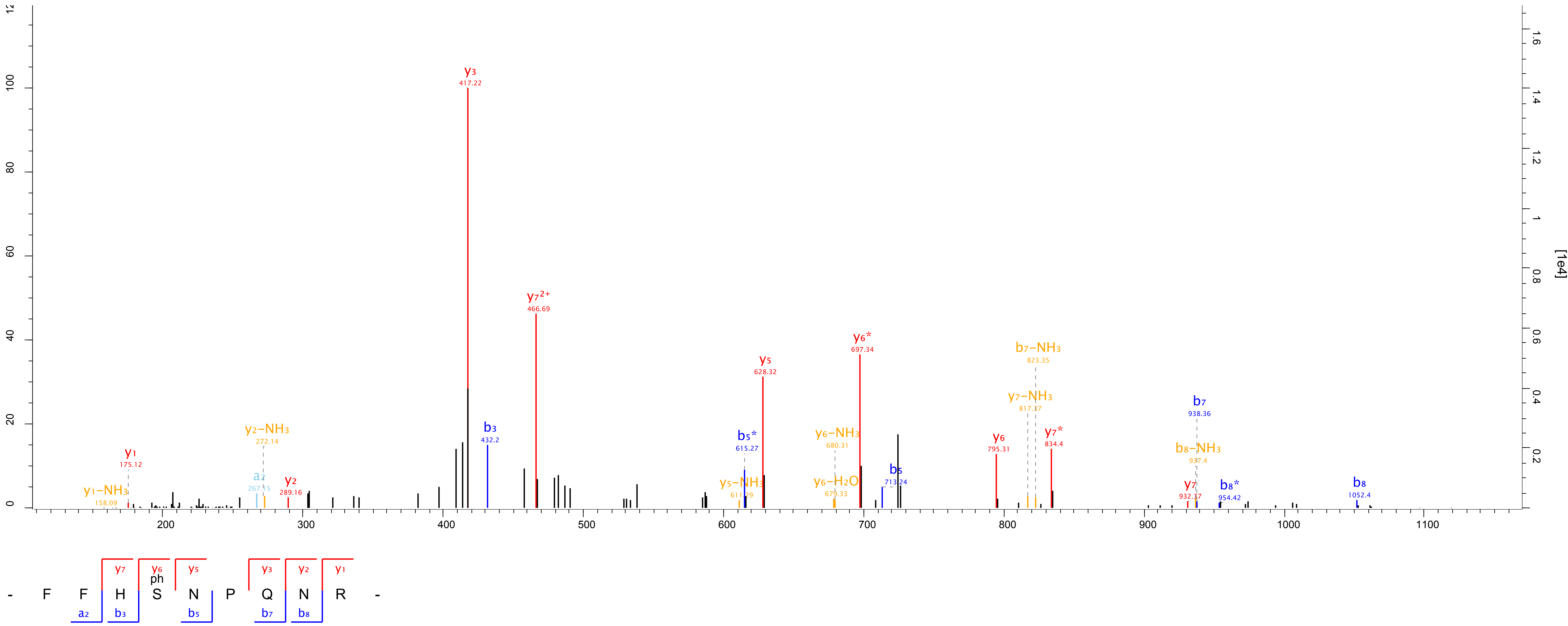

Raw file	Scan	Method	Score	m/z
20130708_PS_Phosphotest_6803wt_SDS01	3949	ITMS; CID	126.71	518.77

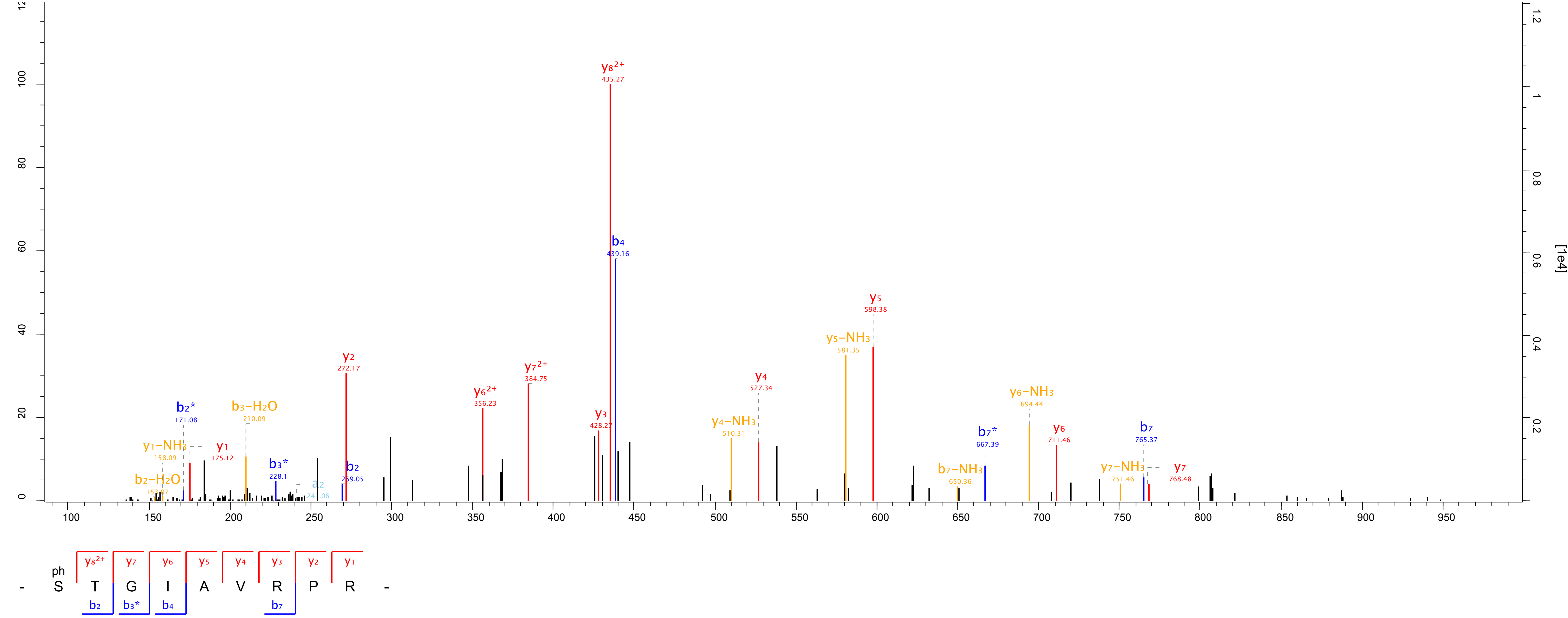

Raw file	Scan	Method	Score	m/z
20130708_PS_Phosphotest_6803wt_SDS01	3989	ITMS; CID	114.71	569.25

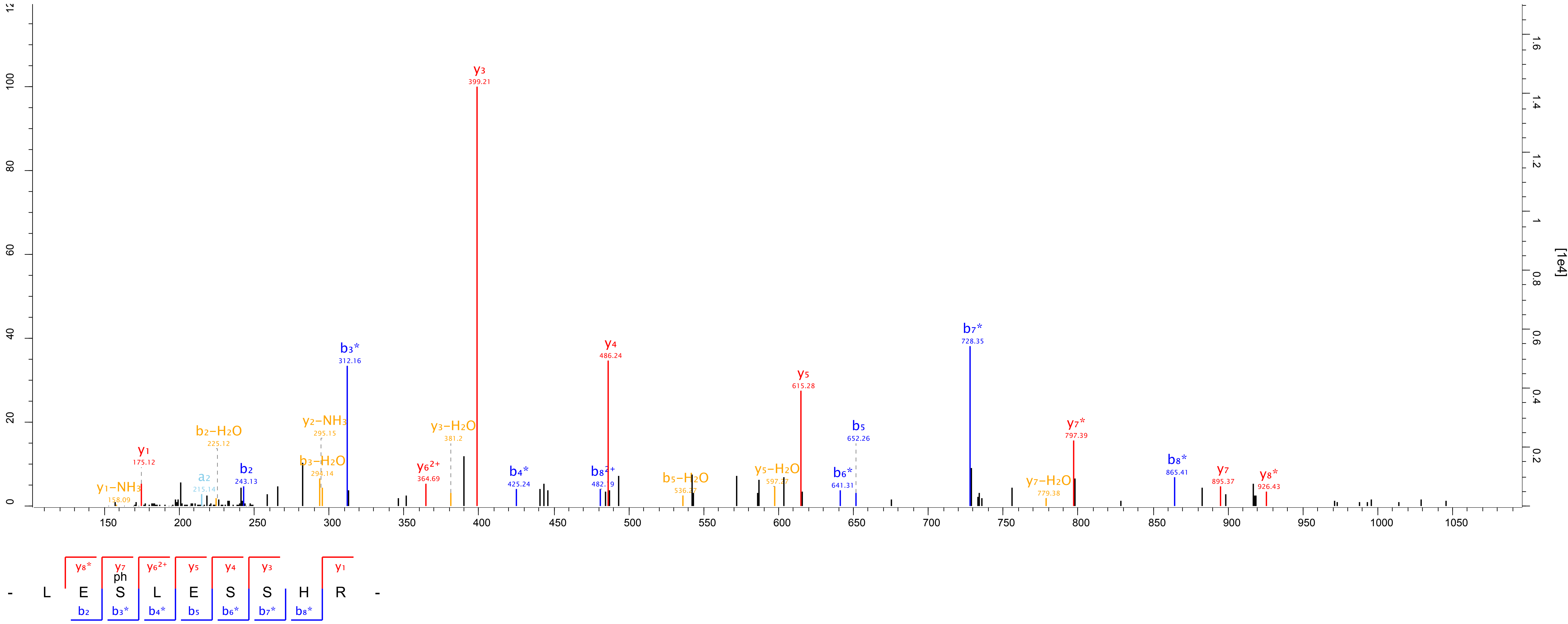

Raw file	Scan	Method	Score	m/z
20130708_PS_Phosphotest_6803wt_YPER01	4032	ITMS; CID	75.1	534.76

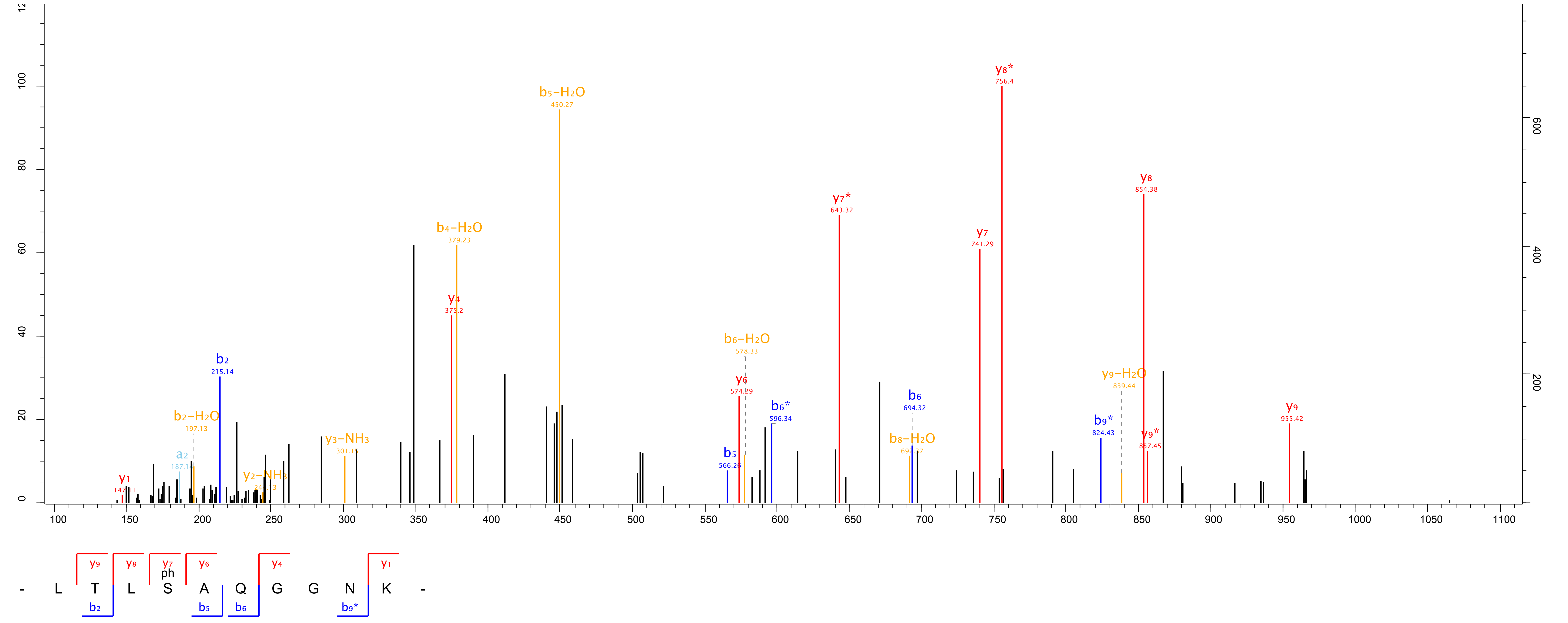

Raw file	Scan	Method	Score	m/z
20130708_PS_Phosphotest_6803wt_SDS01	4039	ITMS; CID	116.98	588.78

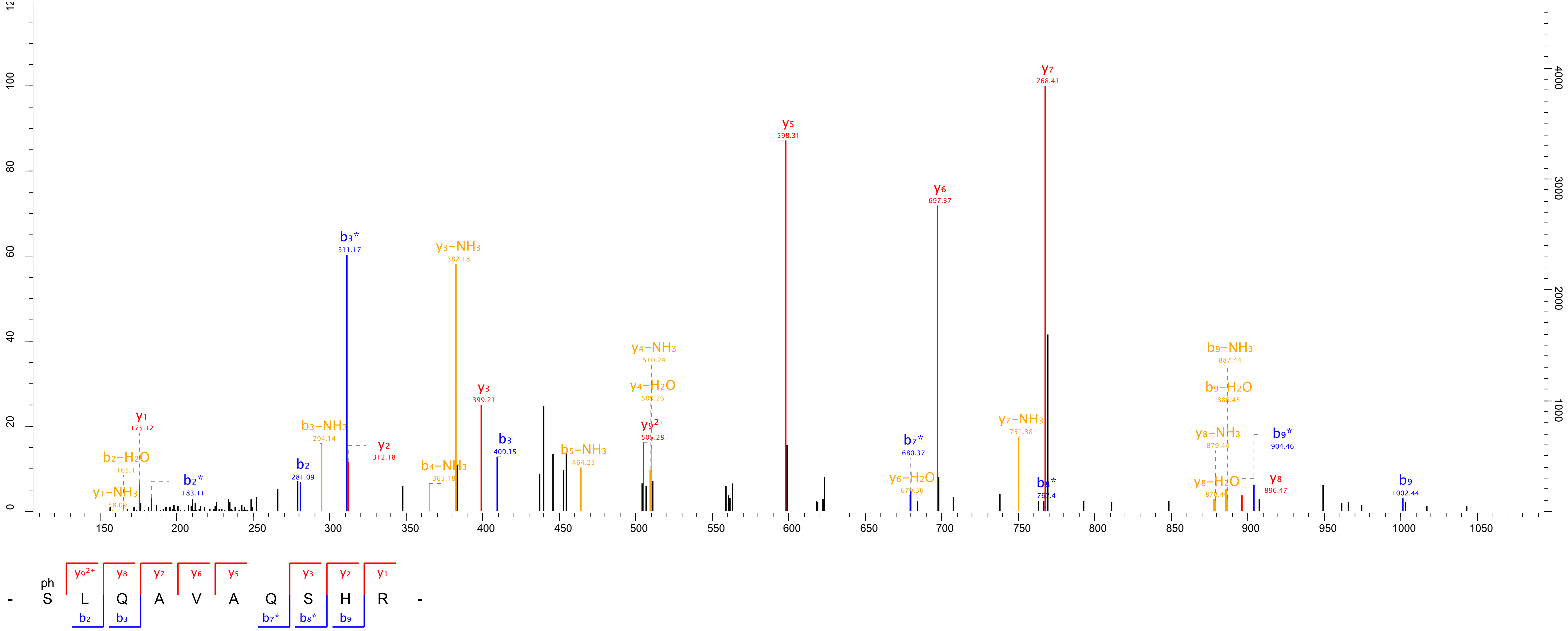

Raw file	Scan	Method	Score	m/z
20130708_PS_Phosphotest_6803wt_YPER01	4113	ITMS; CID	92.3	606.8

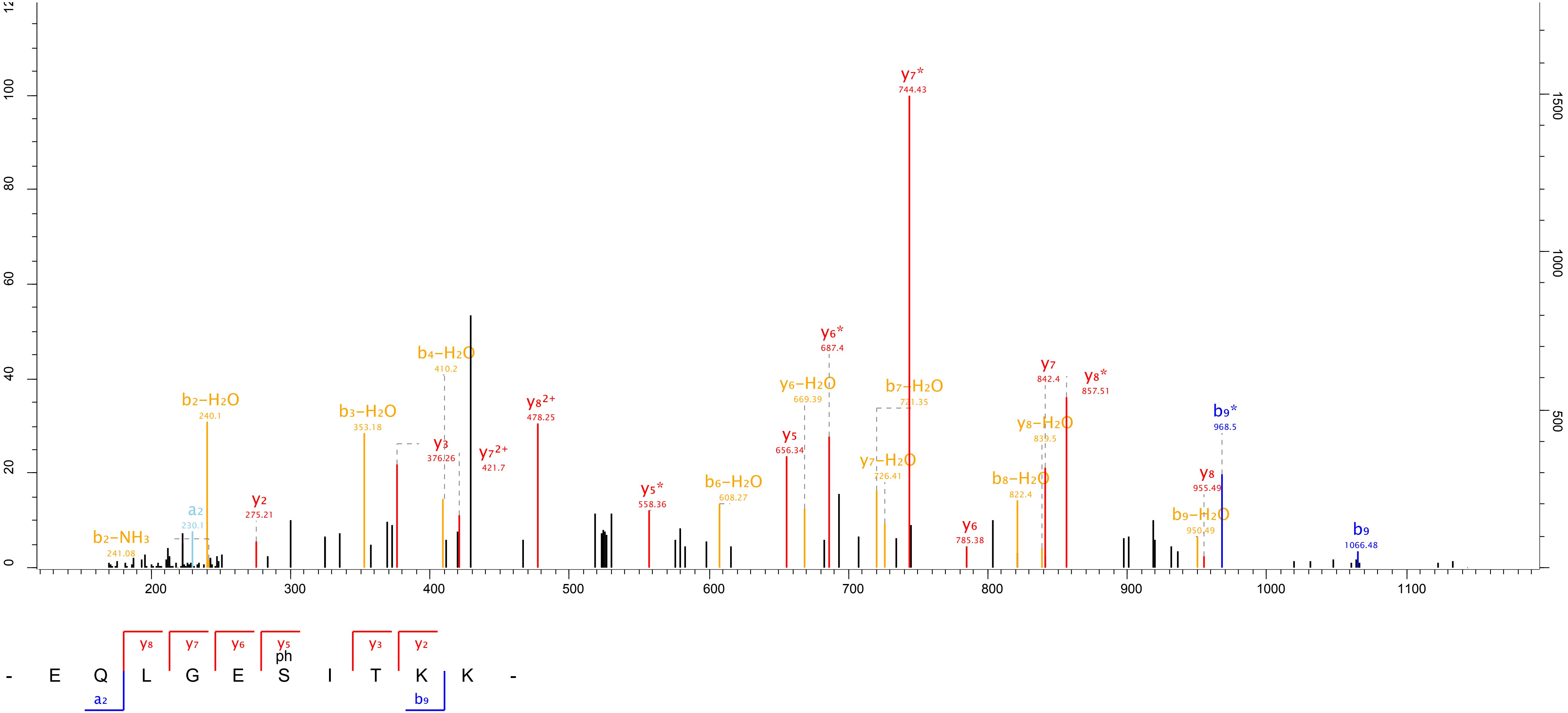

Raw file	Scan	Method	Score	m/z
20130708_PS_Phosphotest_6803wt_SDS01	4140	ITMS; CID	139.19	635.76

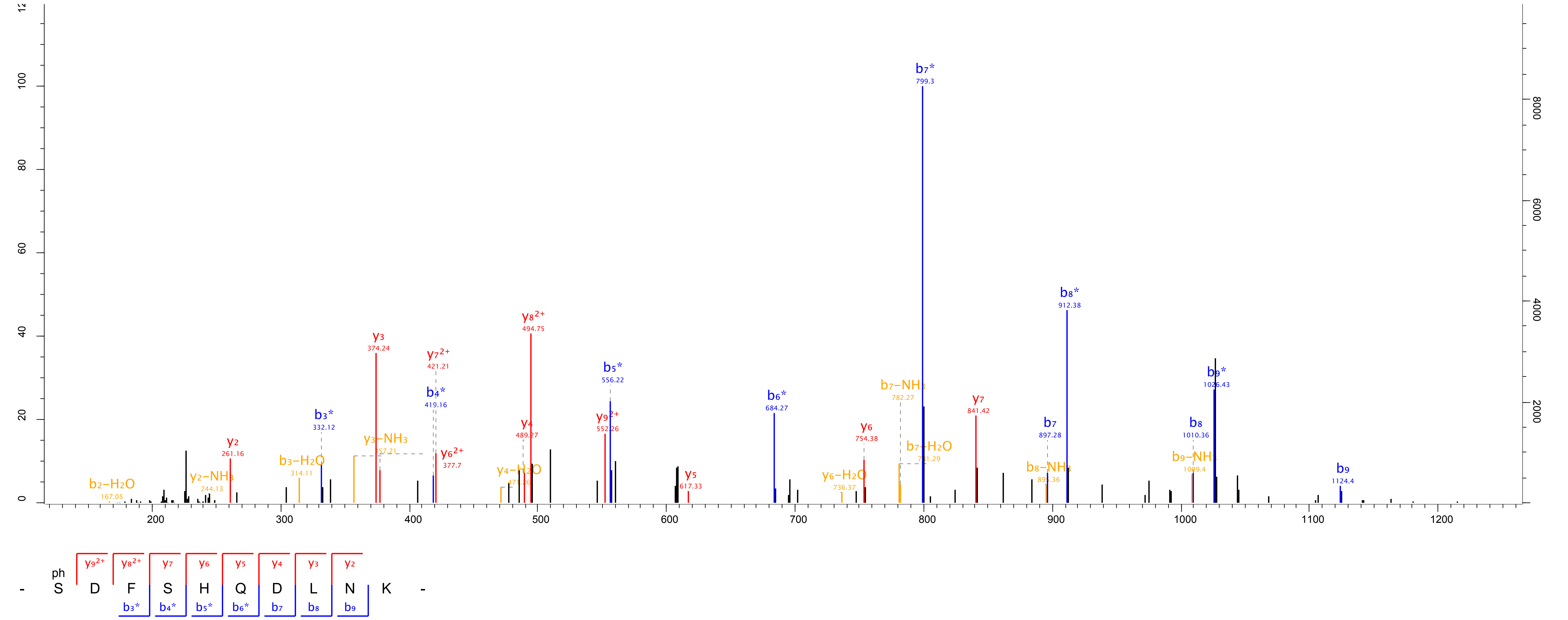

Raw file	Scan	Method	Score	m/z
20130708_PS_Phosphotest_6803wt_SDS04	4164	ITMS; CID	73.88	509.76

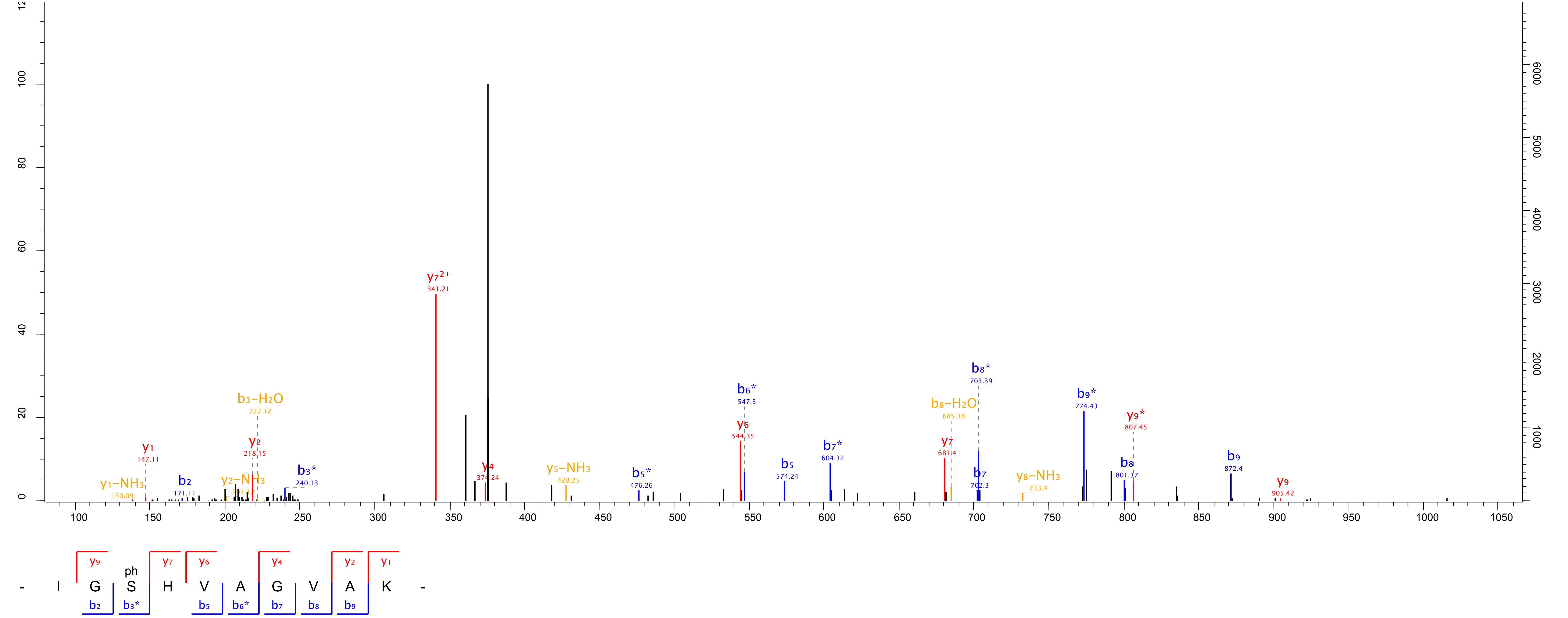

Raw file	Scan	Method	Score	m/z
20130708_PS_Phosphotest_6803wt_SDS01	4200	ITMS; CID	132.01	427.21

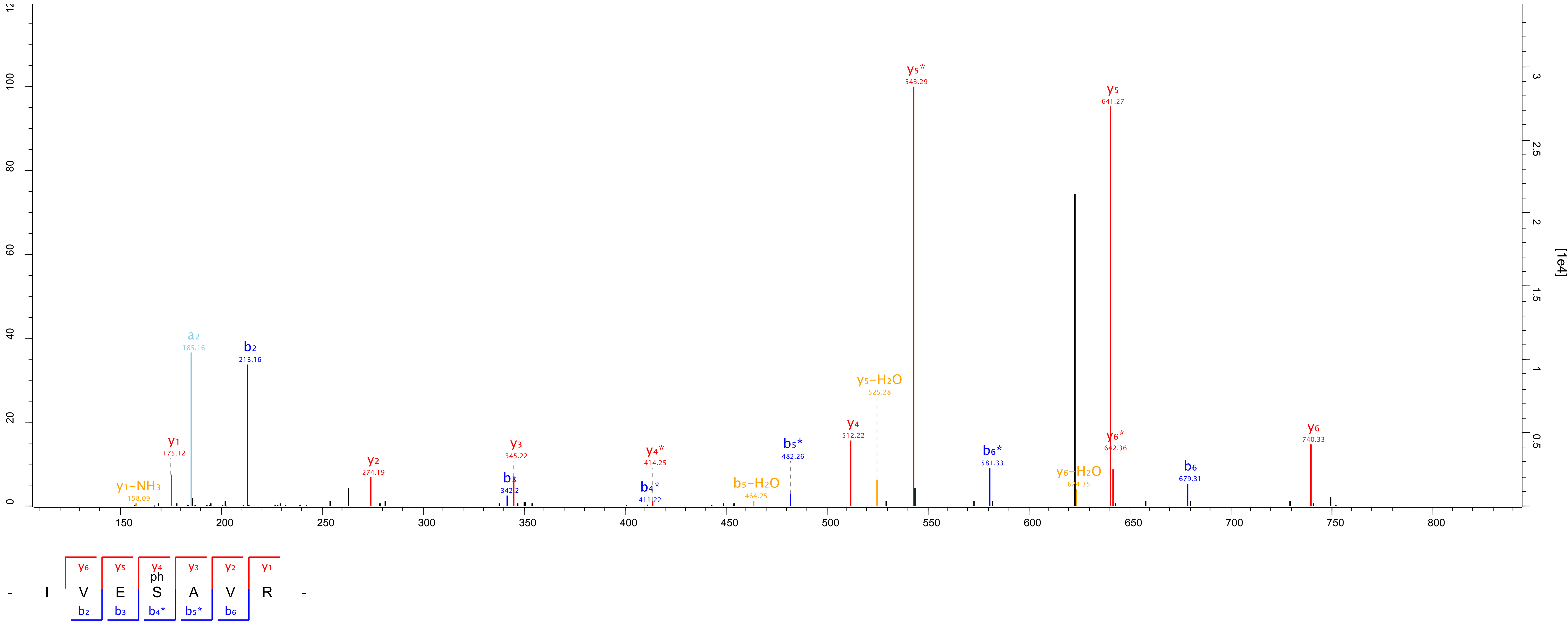

Raw file	Scan	Method	Score	m/z
20130708_PS_Phosphotest_6803wt_YPER01	4214	ITMS; CID	77.63	563.25

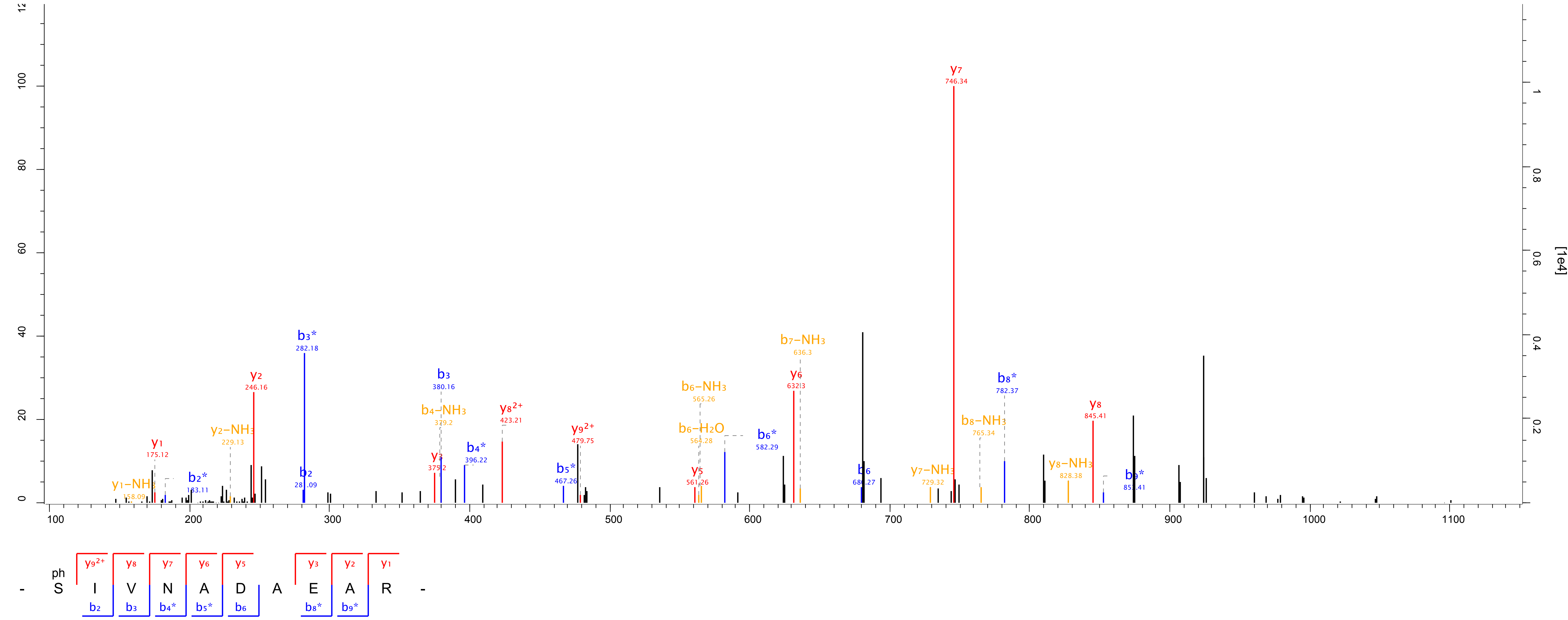

Raw file	Scan	Method	Score	m/z
20130708_PS_Phosphotest_6803wt_SDS02	4278	ITMS; CID	72.34	502.75

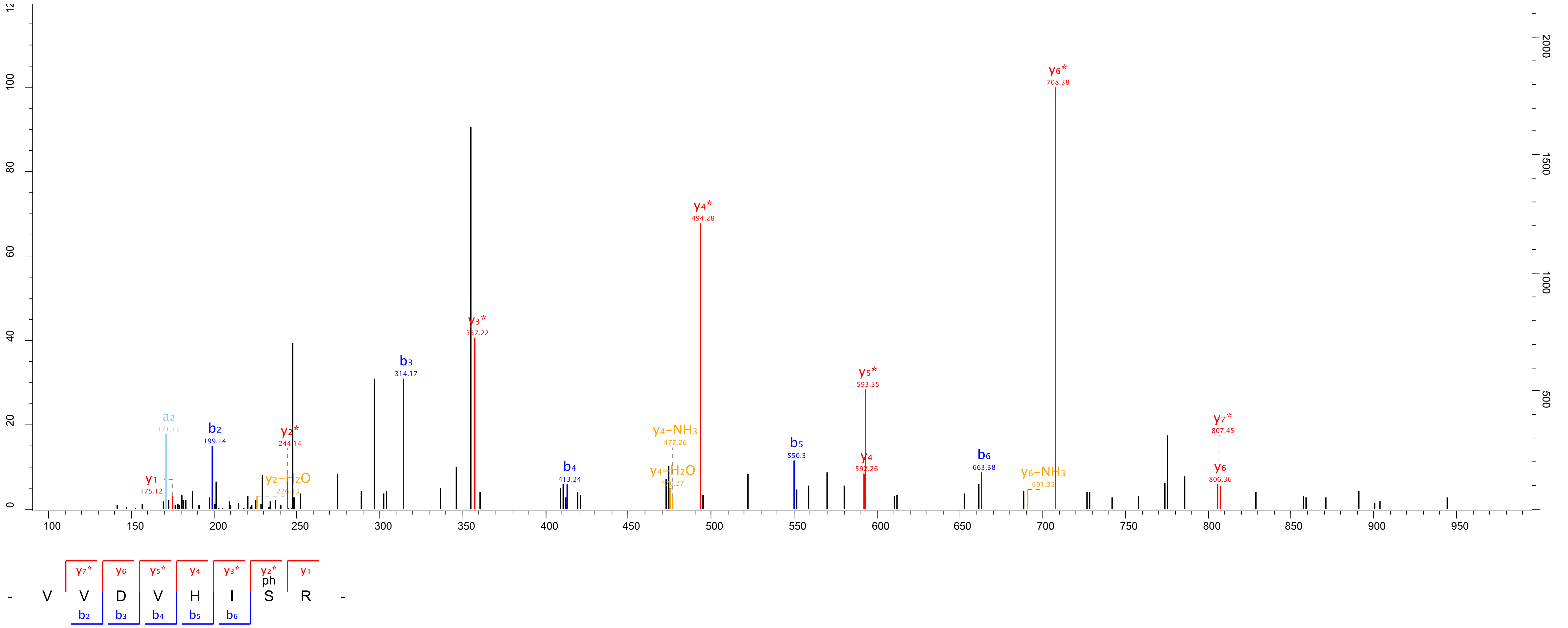

Raw file	Scan	Method	Score	m/z
20130708_PS_Phosphotest_6803wt_SDS01	4307	ITMS; CID	87.71	516.23

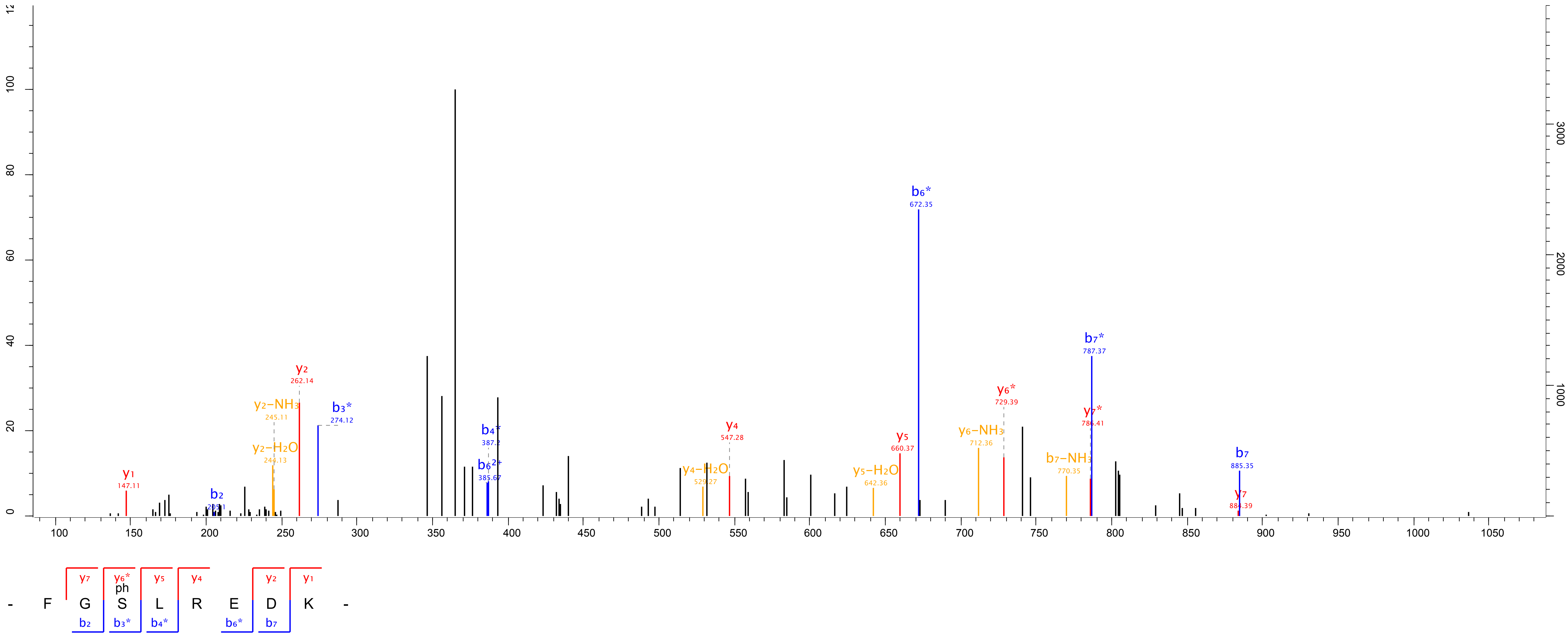

Raw file	Scan	Method	Score	m/z
20130708_PS_Phosphotest_6803wt_YPER02	4337	ITMS; CID	93.84	465.22

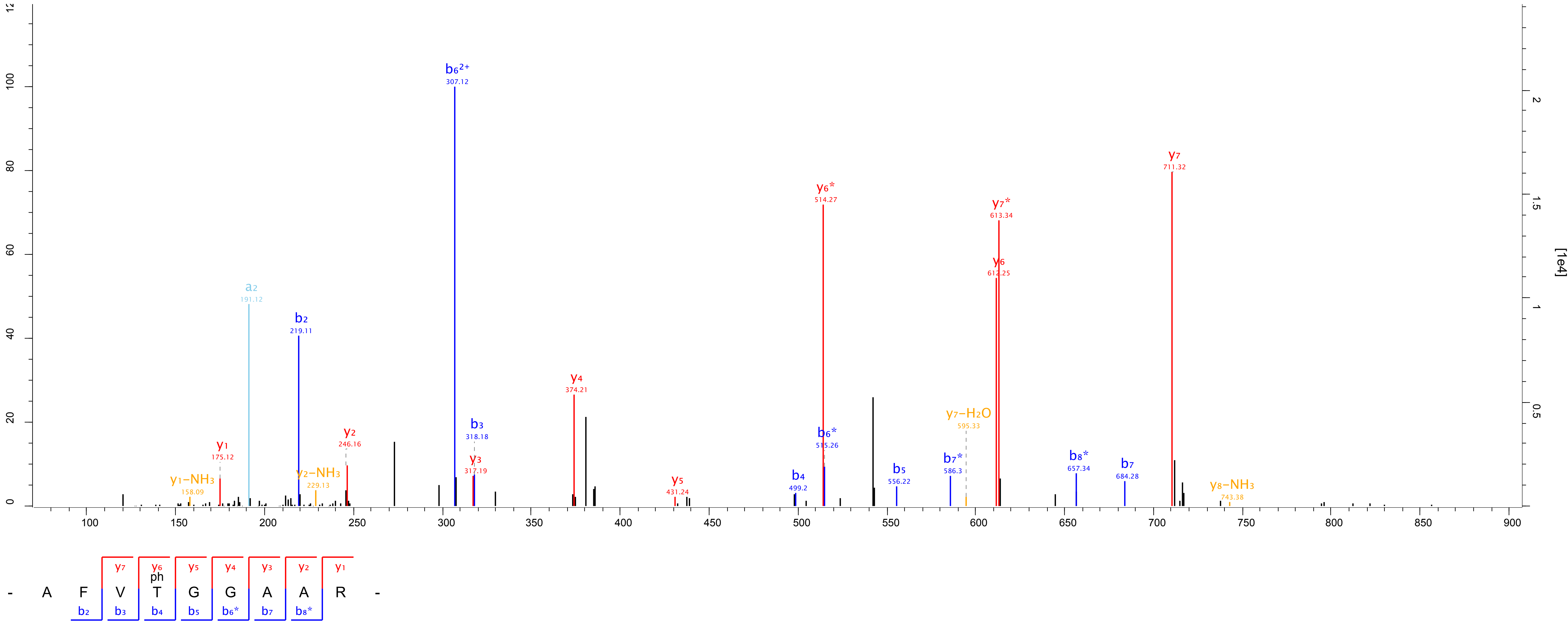

Raw file	Scan	Method	Score	m/z
20130708_PS_Phosphotest_6803wt_SDS01	4601	ITMS; CID	96.15	772.37

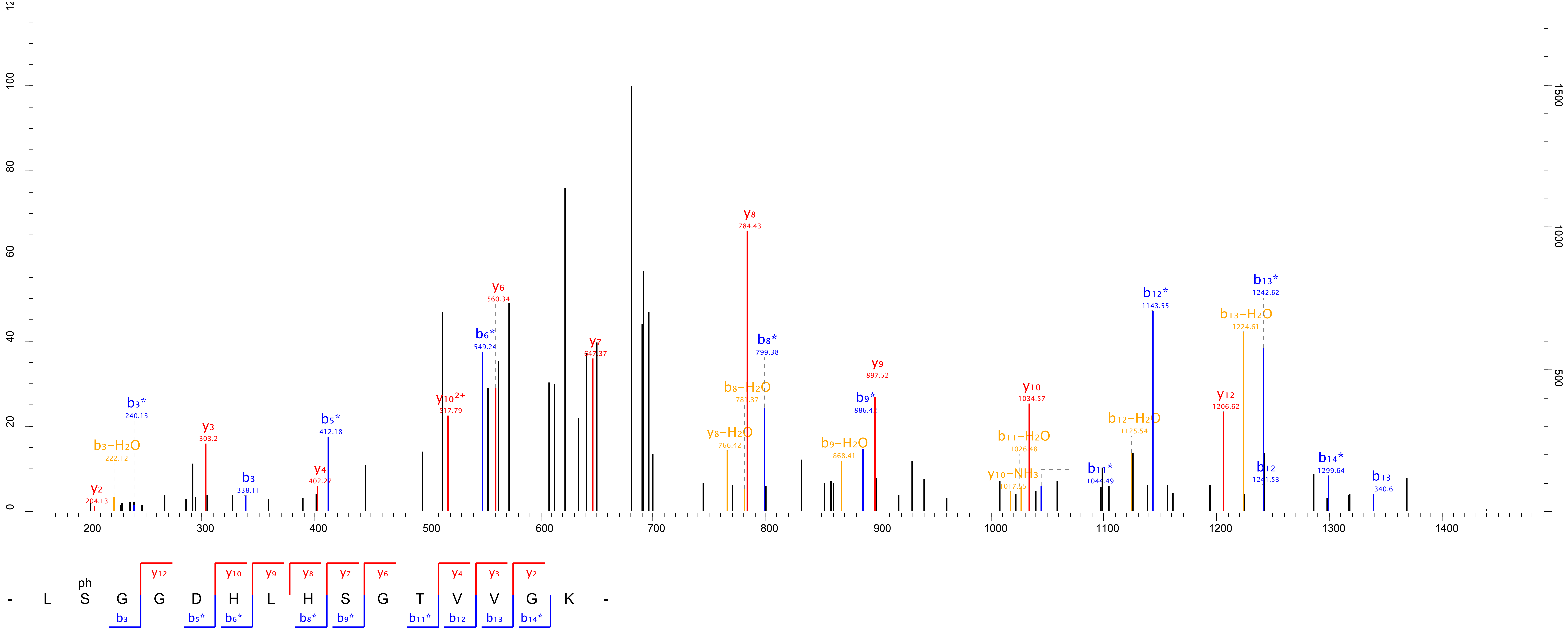

Raw file	Scan	Method	Score	m/z
20130708_PS_Phosphotest_6803wt_SDS01	4652	ITMS; CID	77.53	462.71

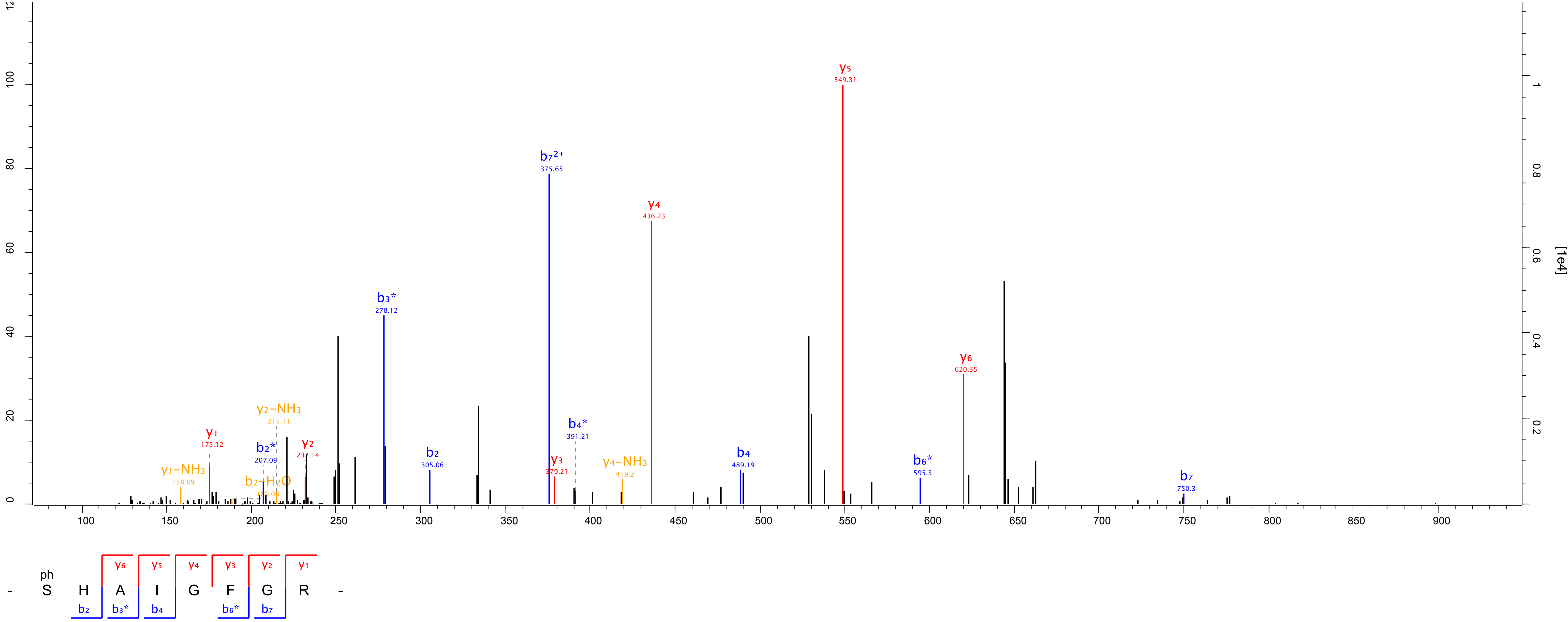

Raw file	Scan	Method	Score	m/z
20130708_PS_Phosphotest_6803wt_YPER01	4772	ITMS; CID	118.51	702.33

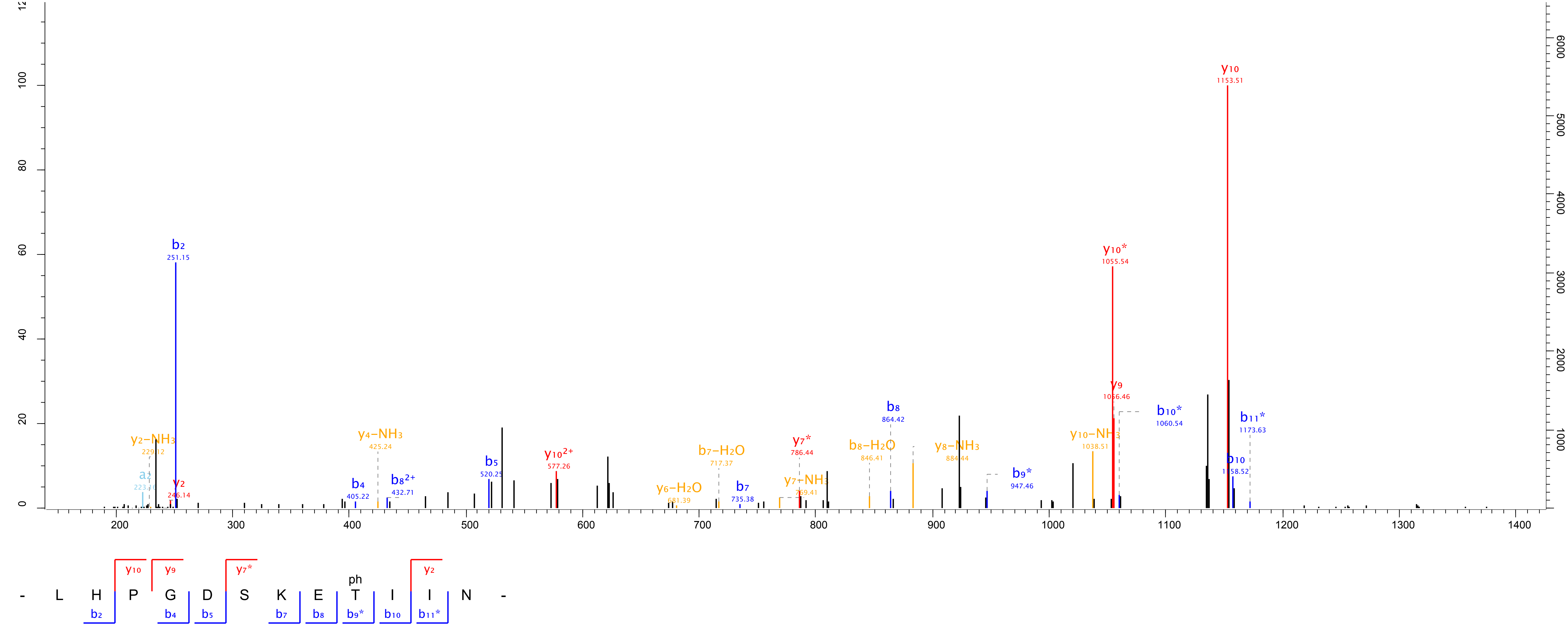

Raw file	Scan	Method	Score	m/z
20130708_PS_Phosphotest_6803wt_SDS01	4917	ITMS; CID	159.44	658.8

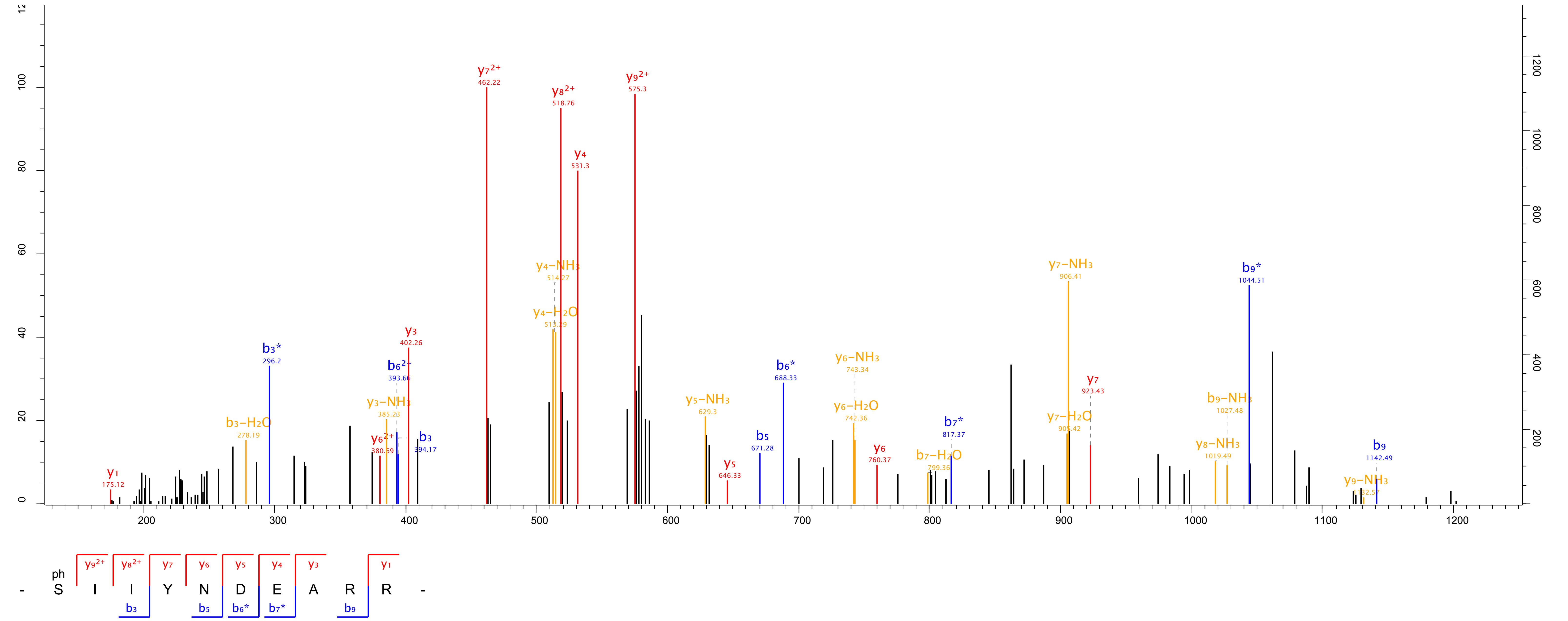

Raw file	Scan	Method	Score	m/z
20130708_PS_Phosphotest_6803wt_YPER01	4973	ITMS; CID	99.24	650.29

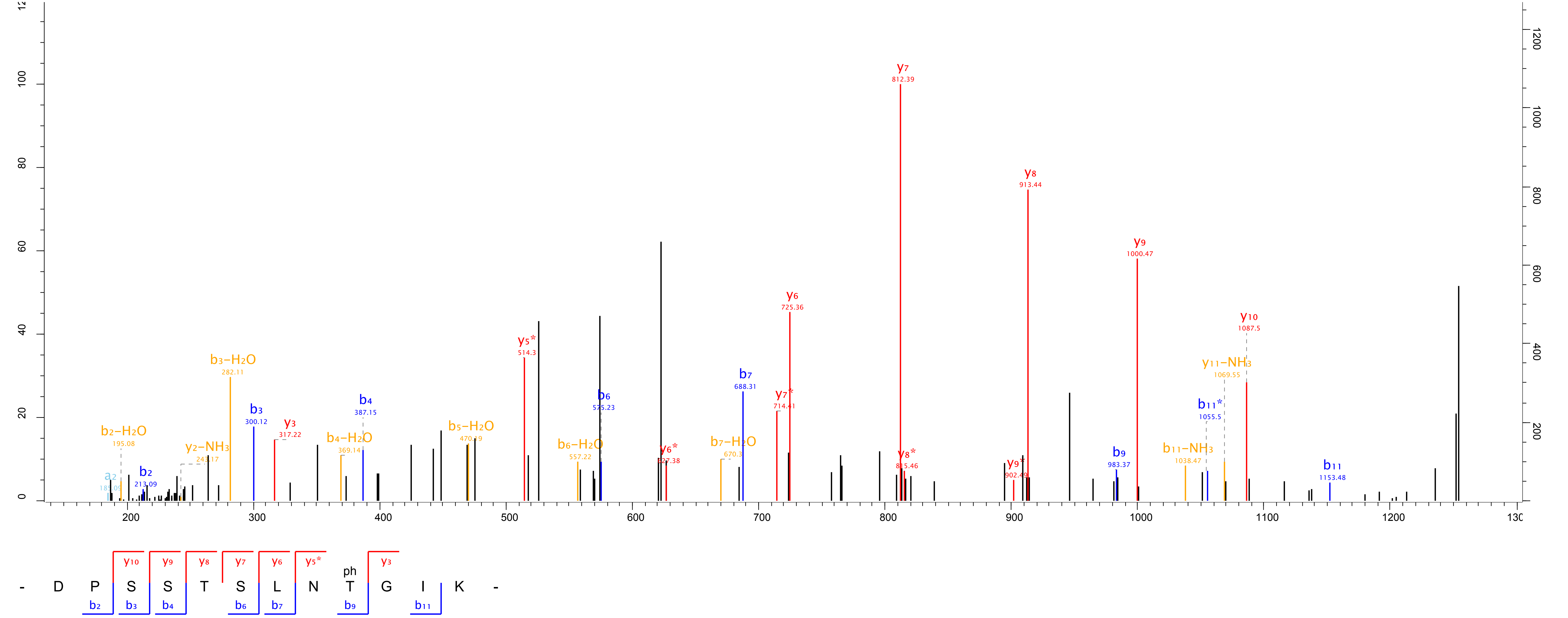

Raw file	Scan	Method	Score	m/z
20130708_PS_Phosphotest_6803wt_SDS02	5080	ITMS; CID	98.58	409.2

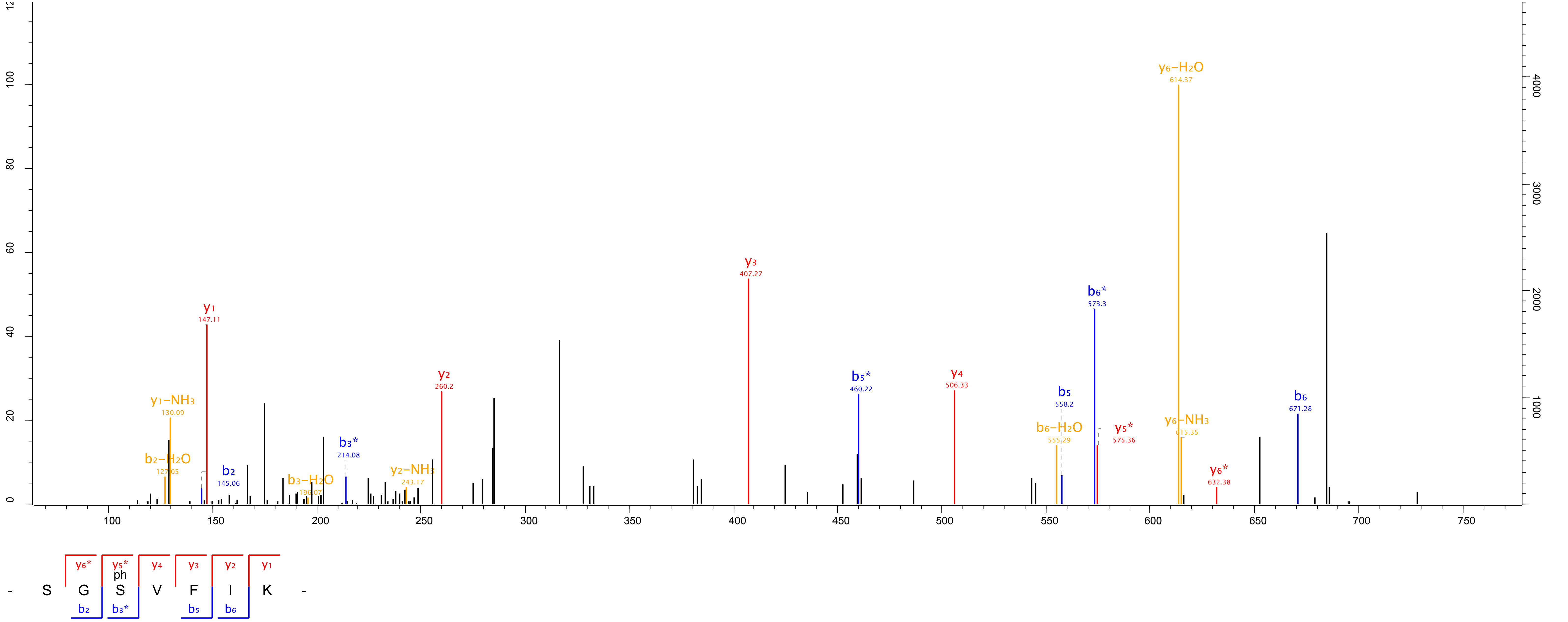

Raw file	Scan	Method	Score	m/z
20130708_PS_Phosphotest_6803wt_SDS01	5087	ITMS; CID	121.6	506.25

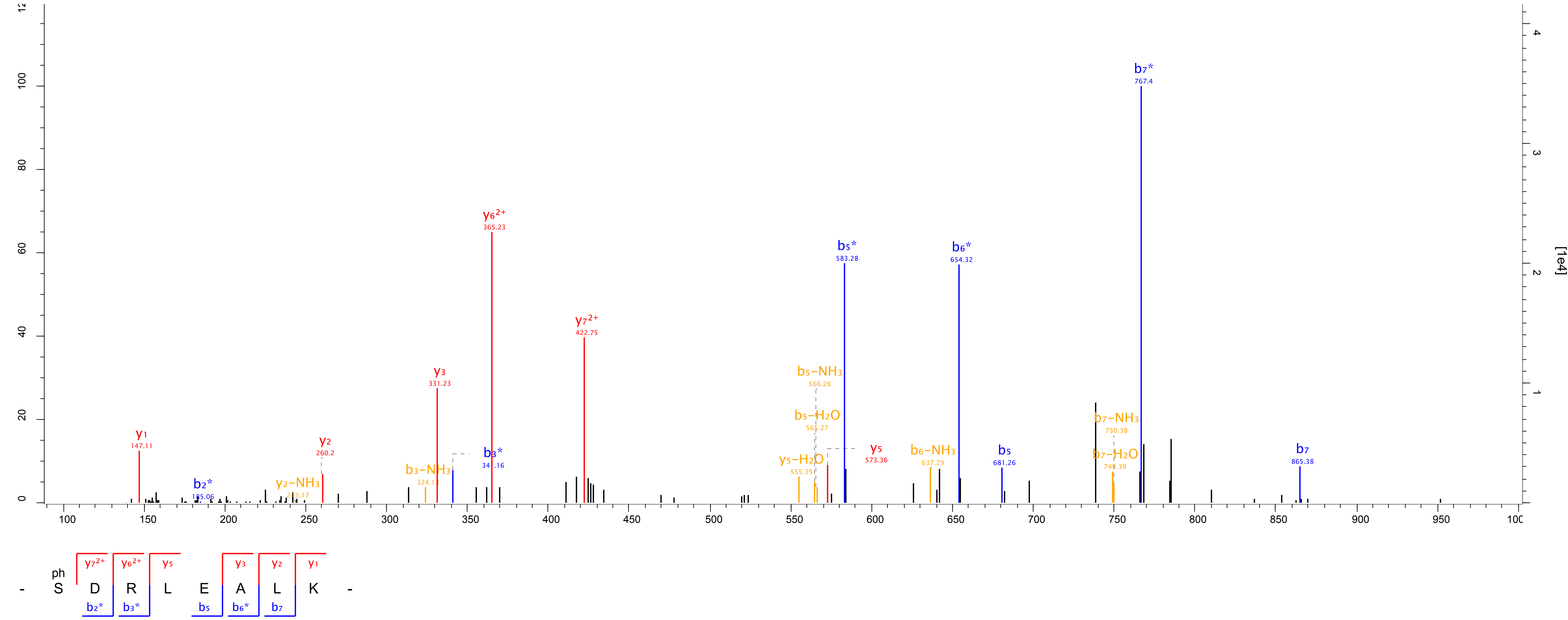

Raw file	Scan	Method	Score	m/z
20130708_PS_Phosphotest_6803wt_SDS01	5162	ITMS; CID	145.23	514.24

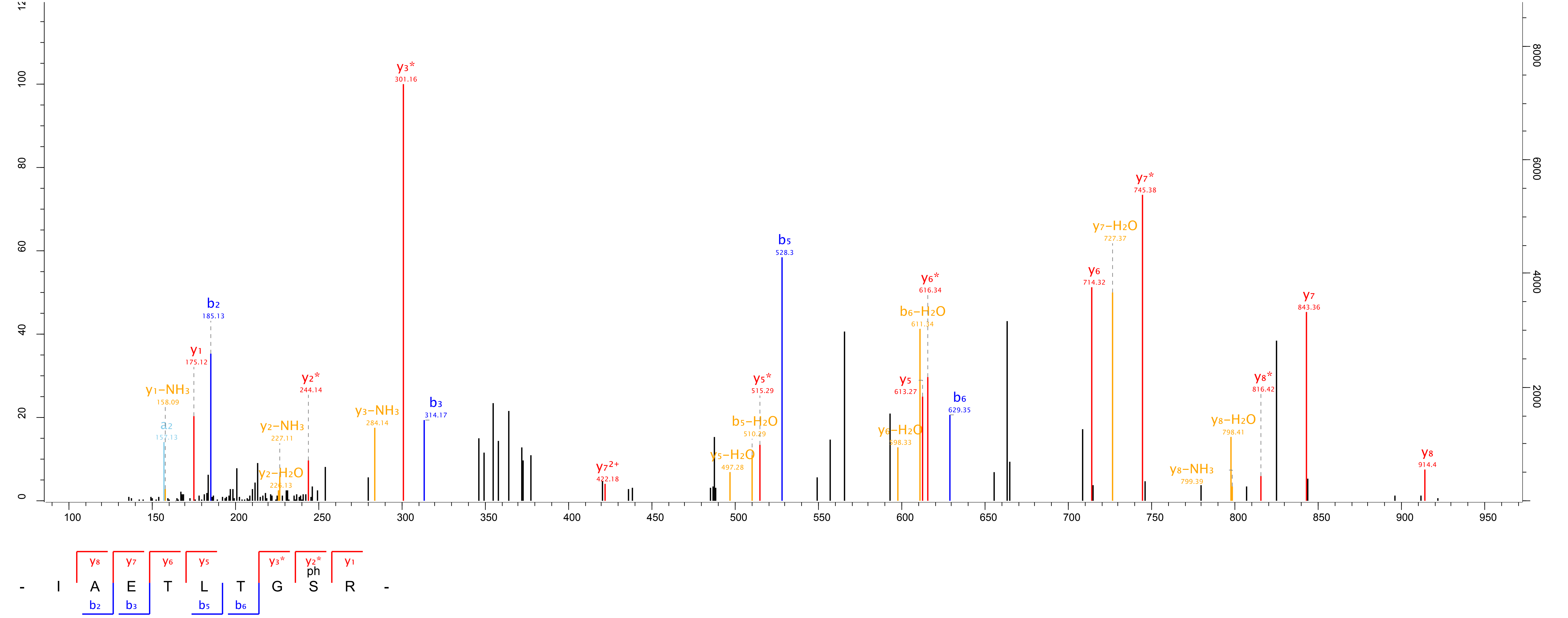

Raw file	Scan	Method	Score	m/z
20130708_PS_Phosphotest_6803wt_SDS02	5165	ITMS; CID	73.39	645.84

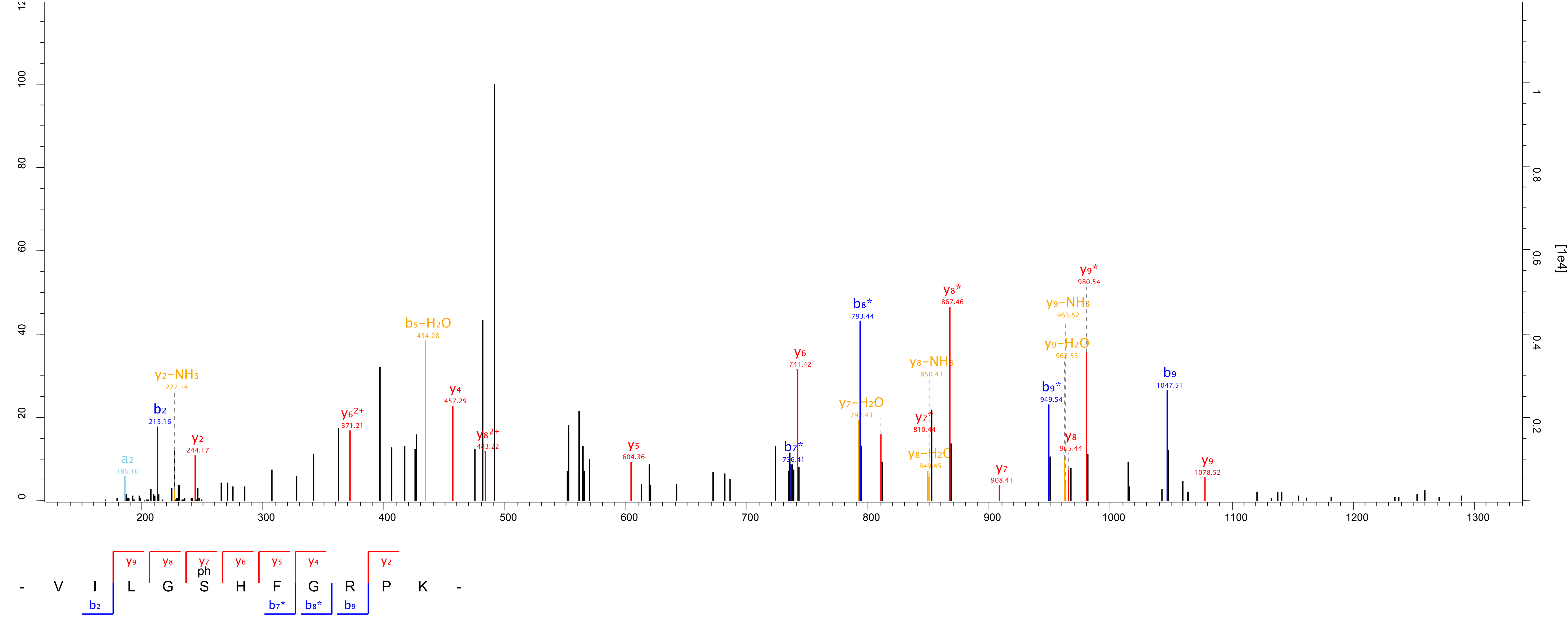

Raw file	Scan	Method	Score	m/z
20130708_PS_Phosphotest_6803wt_SDS02	5183	ITMS; CID	103.55	462.73

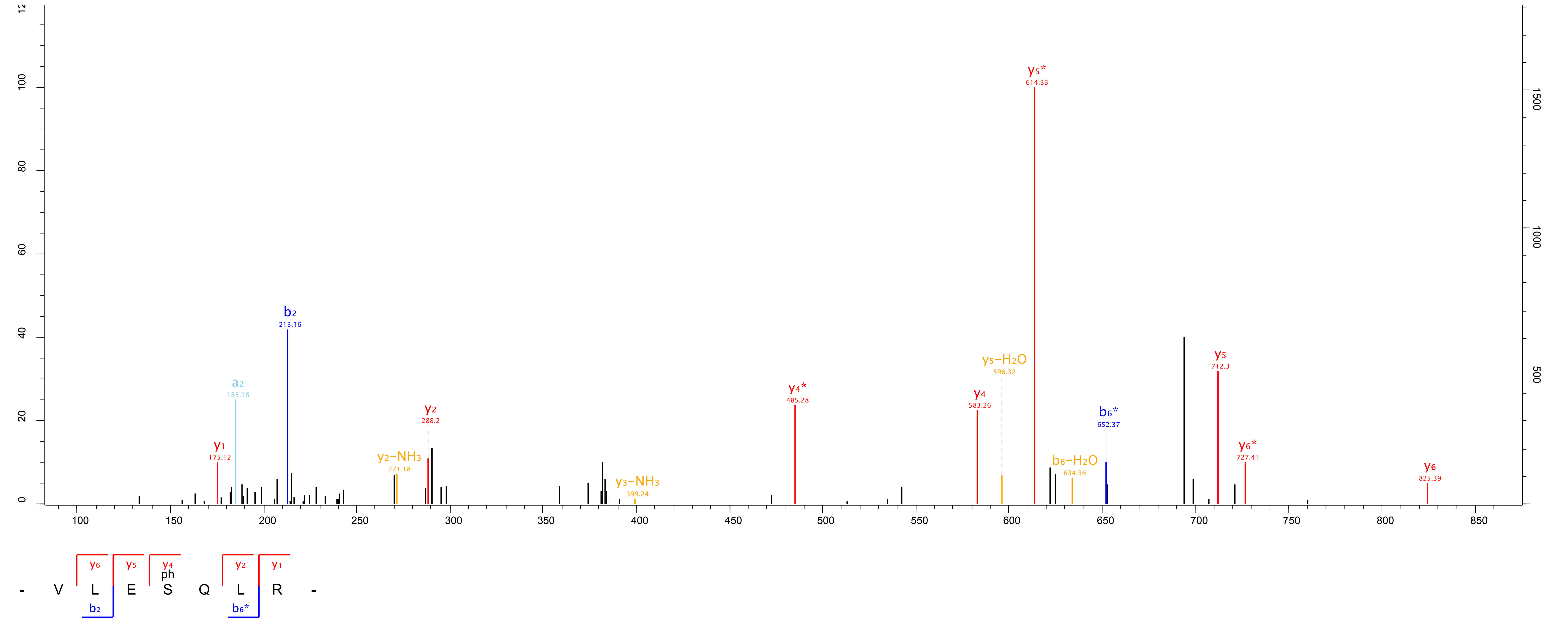

Raw file	Scan	Method	Score	m/z
20130708_PS_Phosphotest_6803wt_YPER01	5235	ITMS; CID	98.06	663.98

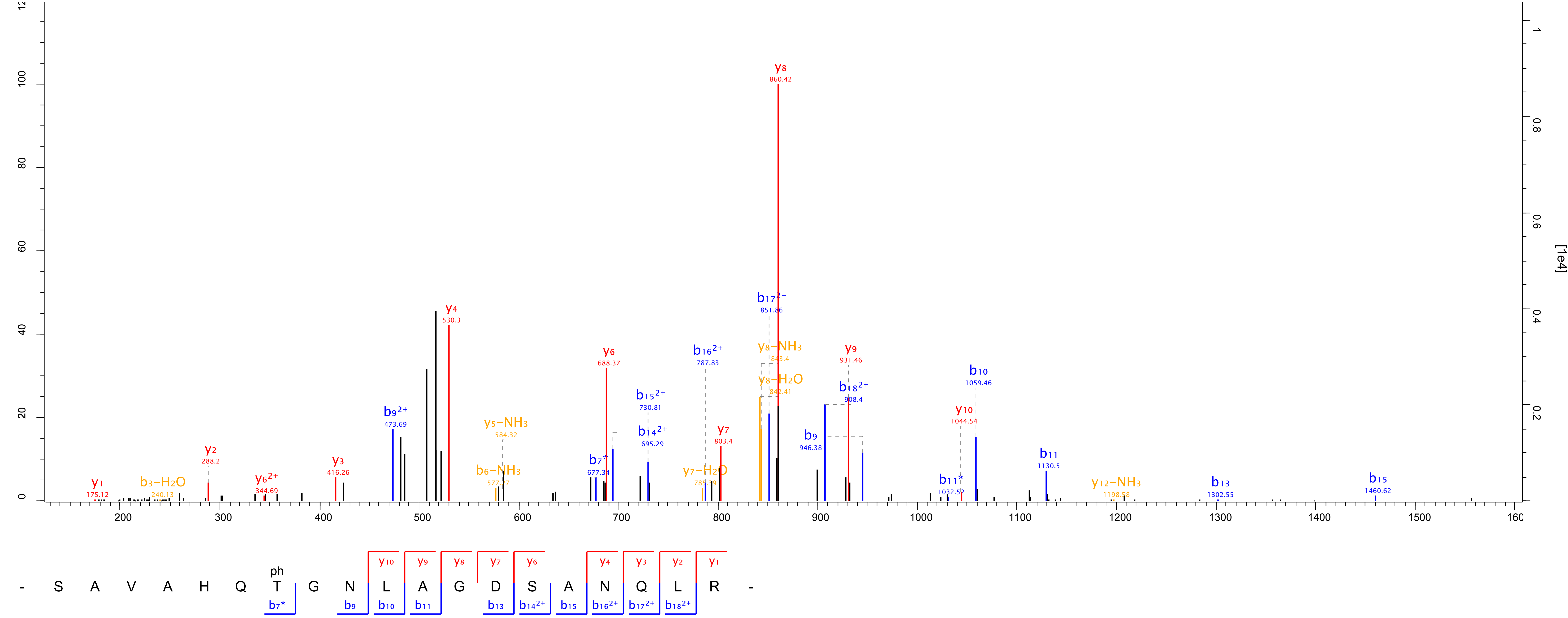

Raw file	Scan	Method	Score	m/z
20130708_PS_Phosphotest_6803wt_SDS01	5307	ITMS; CID	59.15	519.59

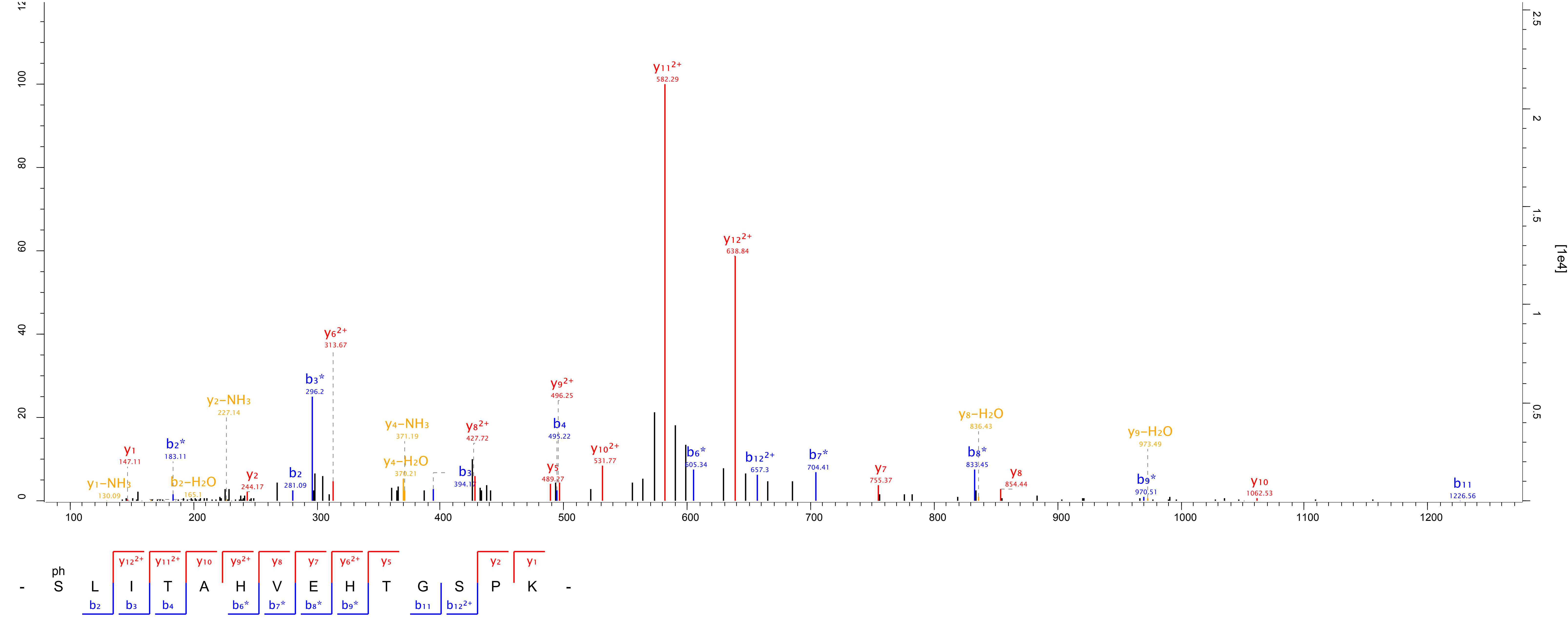

Raw file	Scan	Method	Score	m/z
20130708_PS_Phosphotest_6803wt_SDS01	5316	ITMS; CID	107.79	506.72

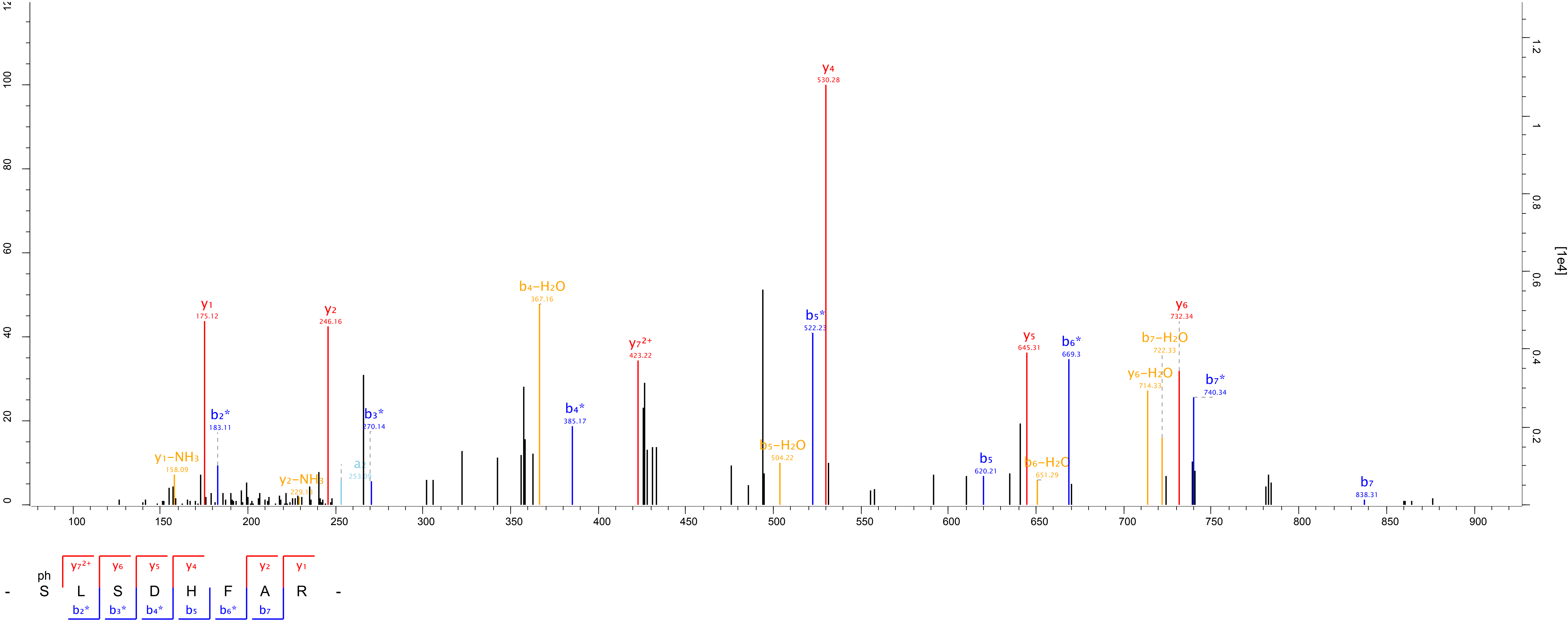

Raw file	Scan	Method	Score	m/z
20130708_PS_Phosphotest_6803wt_SDS01	5339	ITMS; CID	88.6	441.22

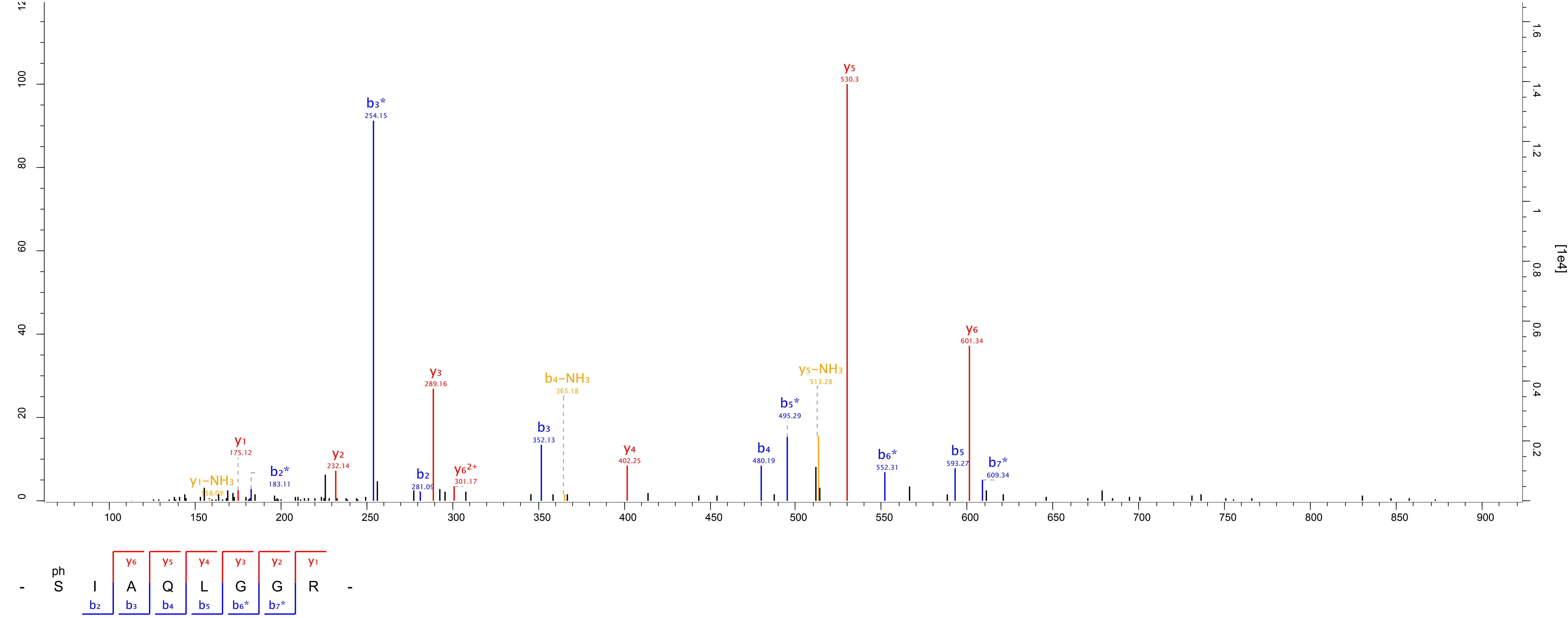

Raw file	Scan	Method	Score	m/z
20130708_PS_Phosphotest_6803wt_SDS01	5353	ITMS; CID	89.8	534.21

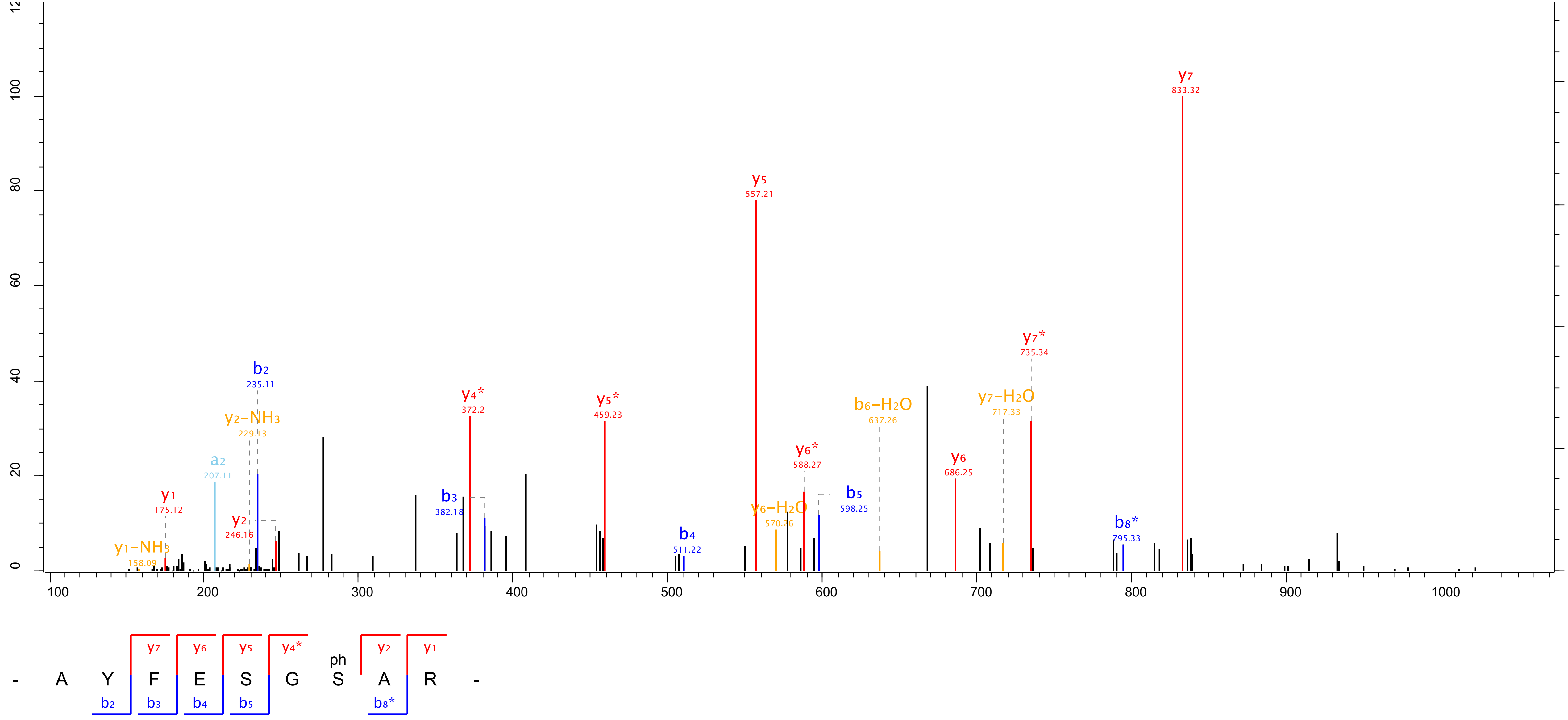

Raw file	Scan	Method	Score	m/z
20130708_PS_Phosphotest_6803wt_SDS01	5421	ITMS; CID	122.39	572.75

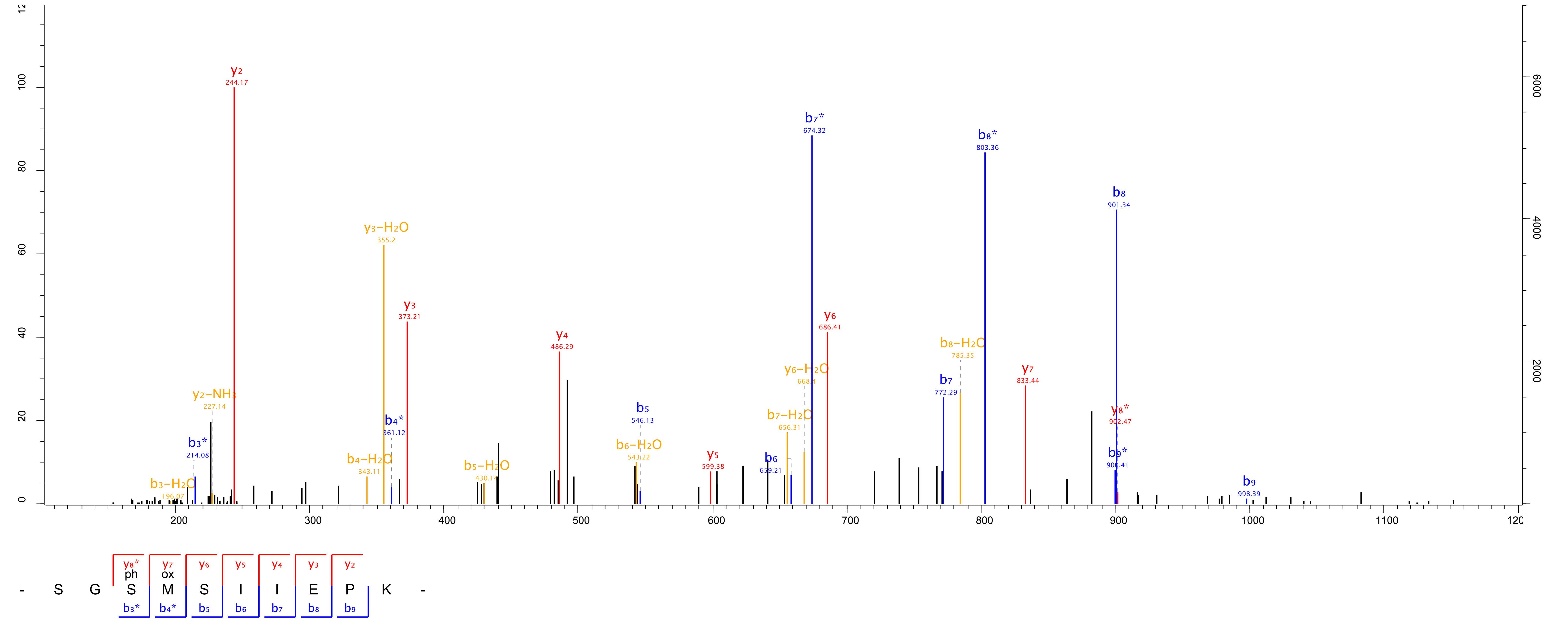

Raw file	Scan	Method	Score	m/z
20130708_PS_Phosphotest_6803wt_YPER01	5469	ITMS; CID	110.92	555.28

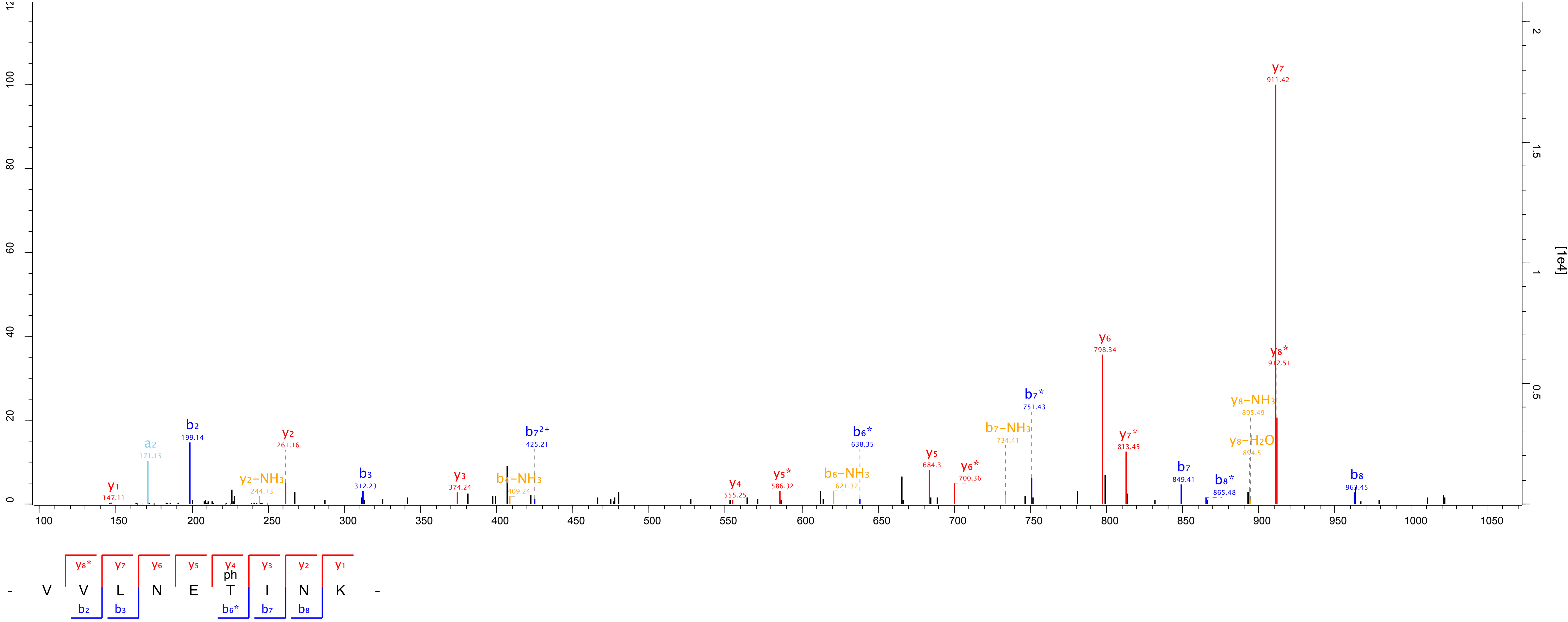

Raw file	Scan	Method	Score	m/z
20130708_PS_Phosphotest_6803wt_SDS01	5522	ITMS; CID	208.51	569.28

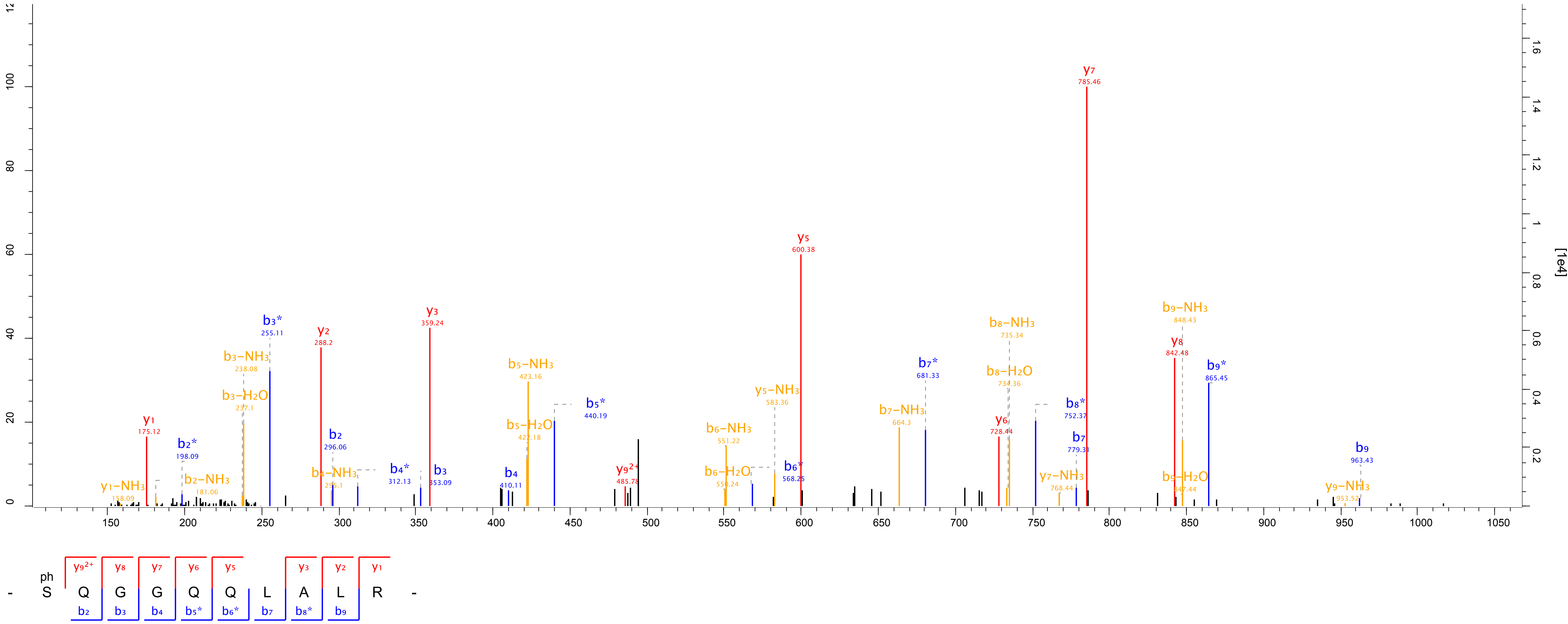

Raw file	Scan	Method	Score	m/z
20130708_PS_Phosphotest_6803wt_SDS01	5681	ITMS; CID	108.56	548.28

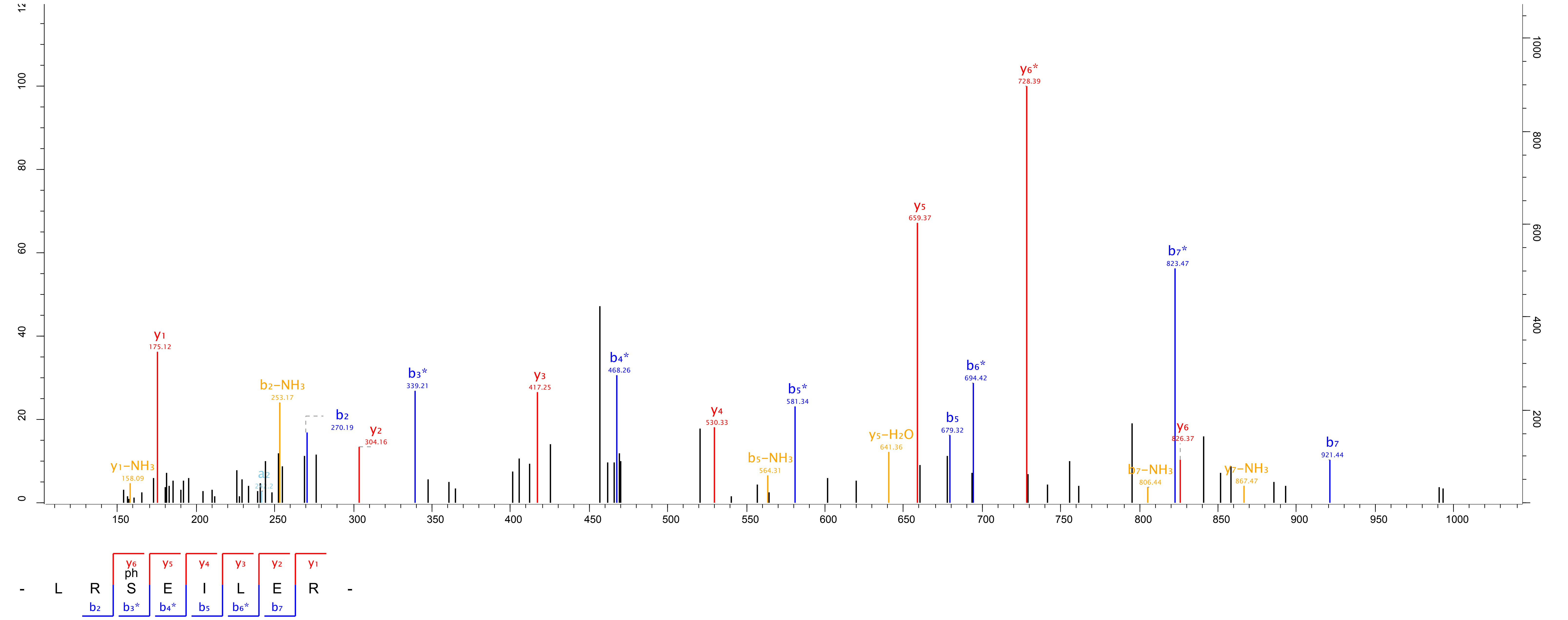

Raw file	Scan	Method	Score	m/z
20130708_PS_Phosphotest_6803wt_SDS01	5721	ITMS; CID	106.58	559.57

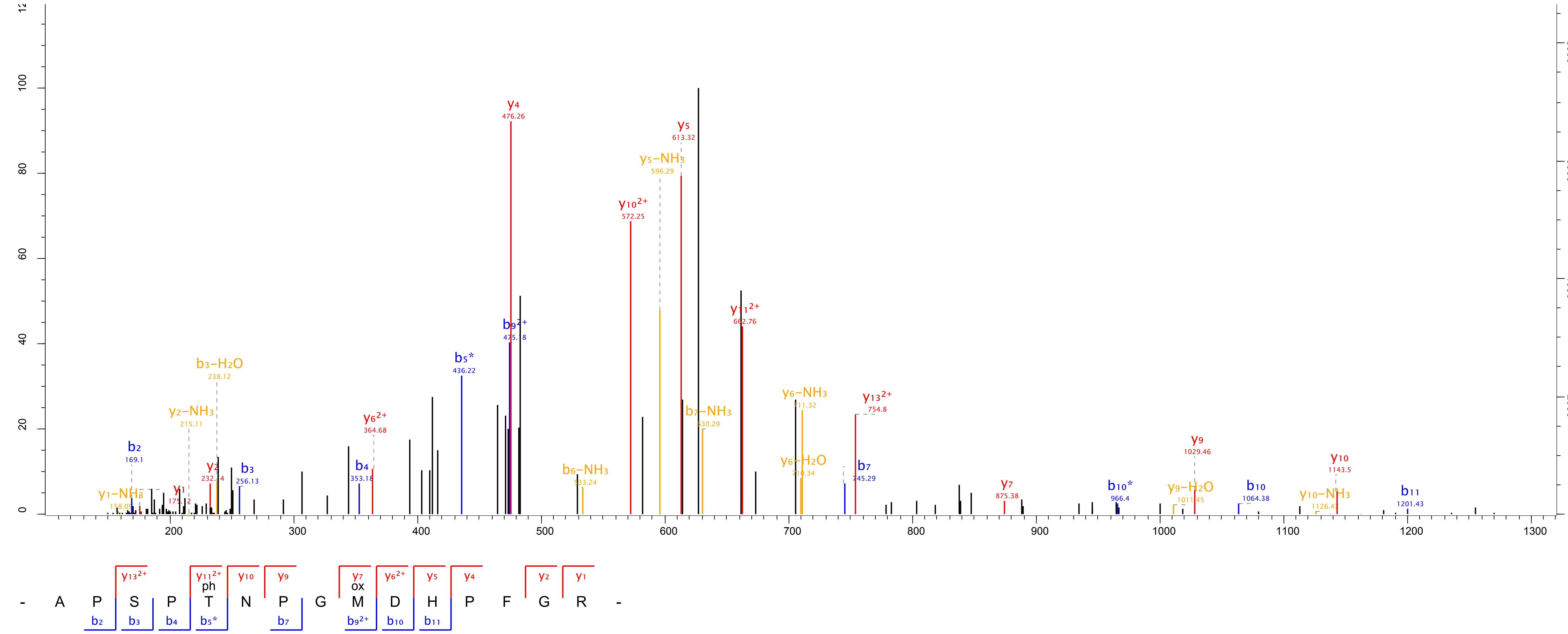

Raw file	Scan	Method	Score	m/z
20130708_PS_Phosphotest_6803wt_SDS01	5791	ITMS; CID	93.48	616.29

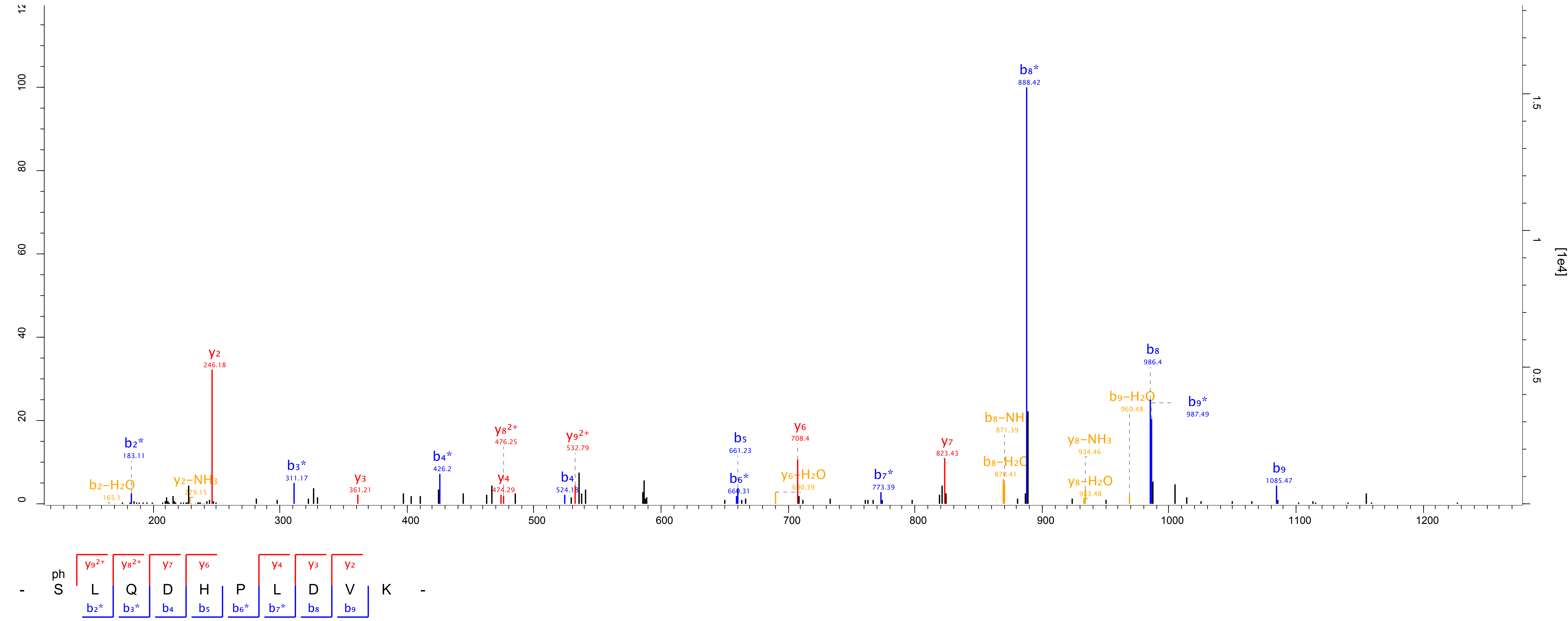

Raw file	Scan	Method	Score	m/z
20130708_PS_Phosphotest_6803wt_SDS02	5818	ITMS; CID	111.09	541.59

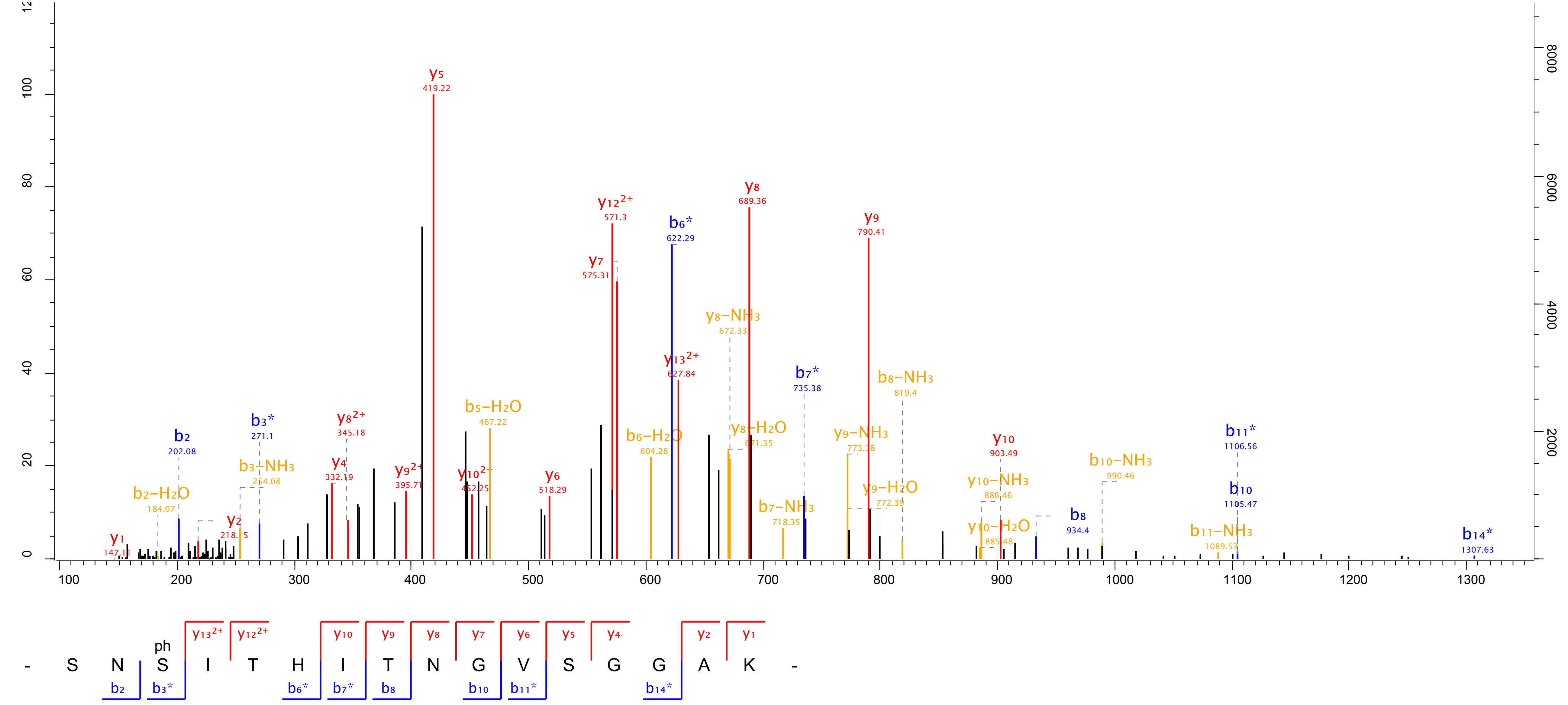

Raw file	Scan	Method	Score	m/z
20130708_PS_Phosphotest_6803wt_SDS01	5829	ITMS; CID	113.61	560.77

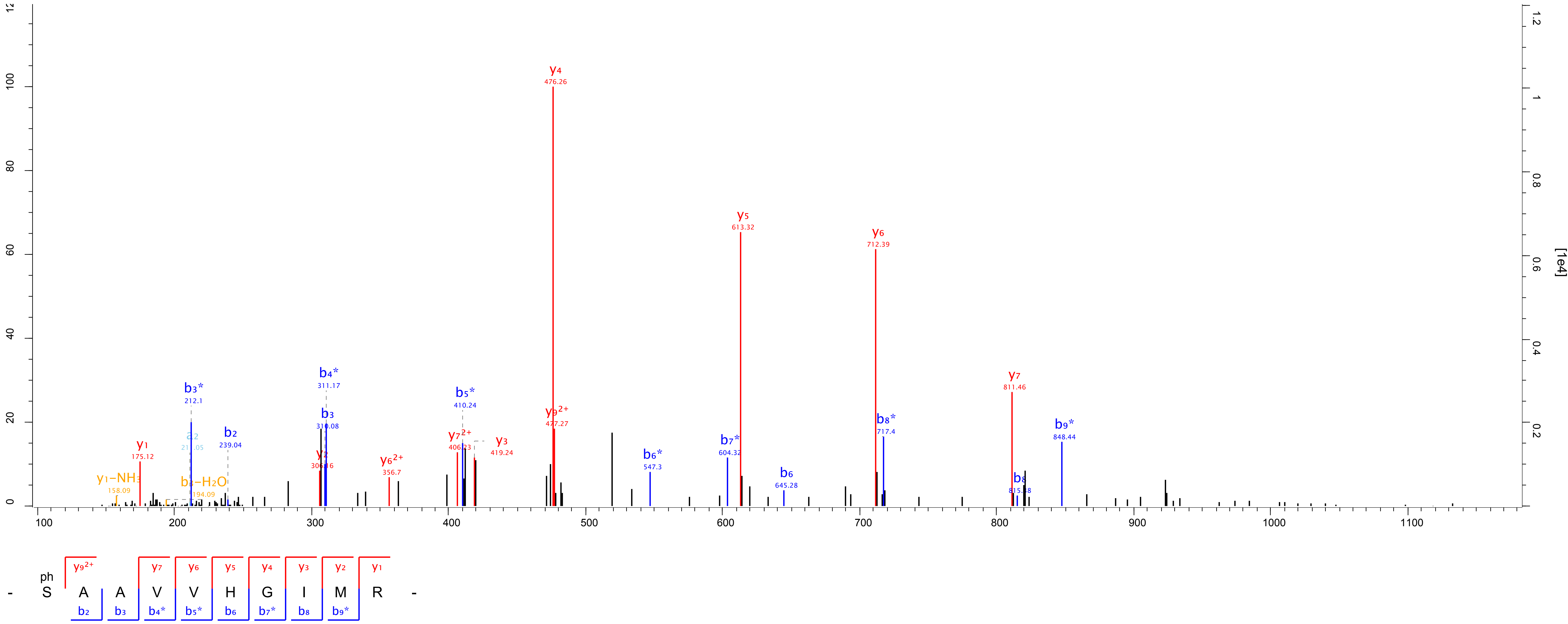

Raw file	Scan	Method	Score	m/z
20130708_PS_Phosphotest_6803wt_SDS02	5831	ITMS; CID	108.58	603.3

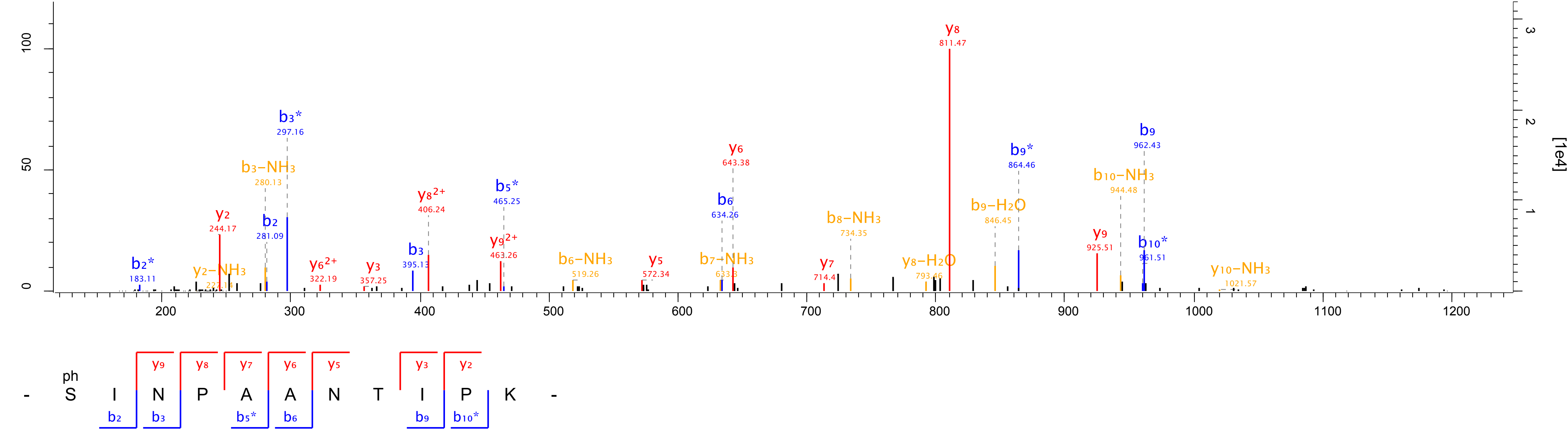

Raw file	Scan	Method	Score	m/z
20130708_PS_Phosphotest_6803wt_SDS01	5834	ITMS; CID	131.66	599.27

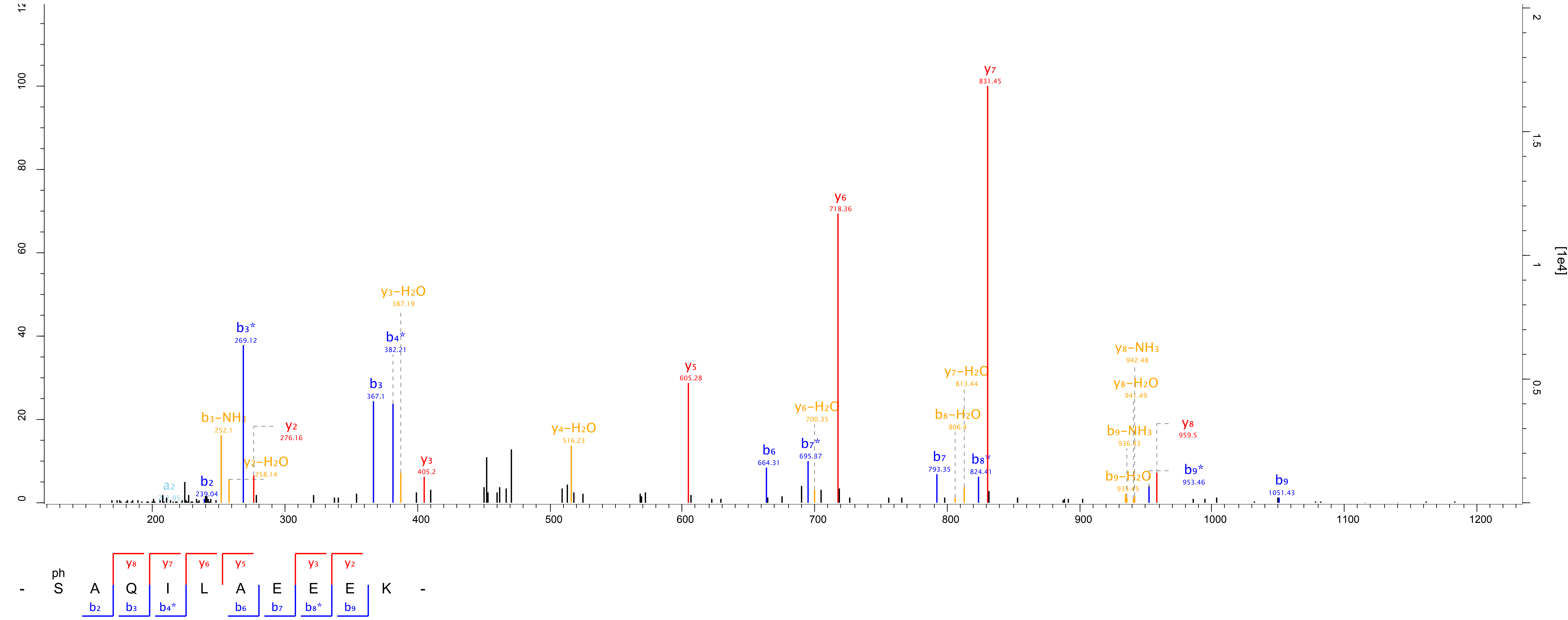

Raw file	Scan	Method	Score	m/z
20130708_PS_Phosphotest_6803wt_SDS01	5847	ITMS; CID	113.37	655.32

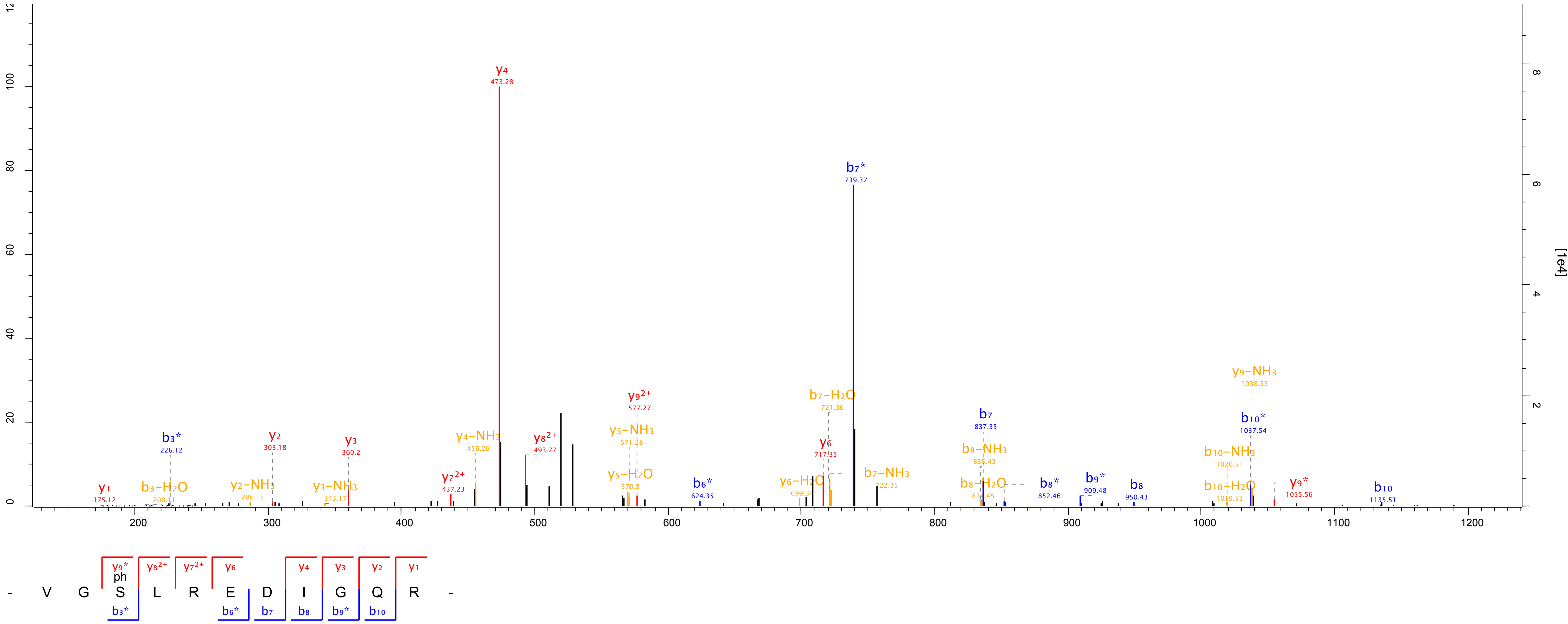

Raw file	Scan	Method	Score	m/z
20130708_PS_Phosphotest_6803wt_YPER01	5865	ITMS; CID	121.82	670.32

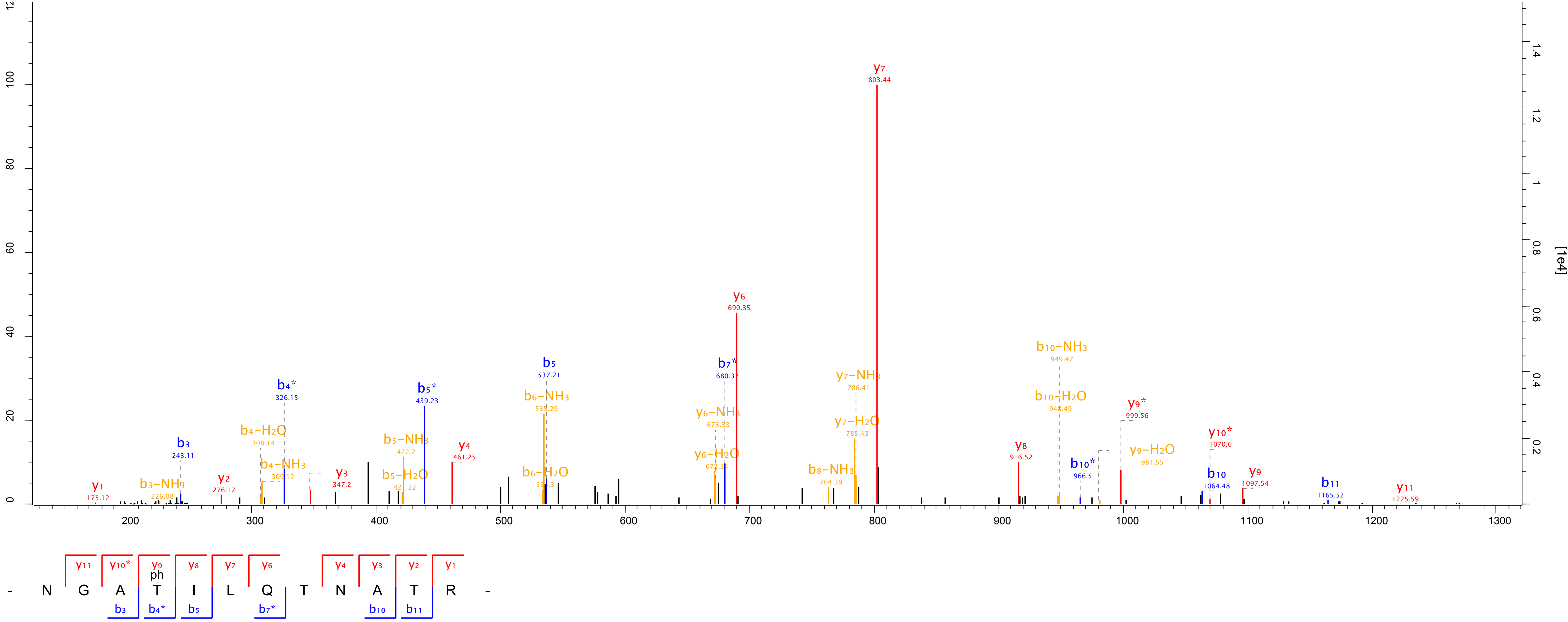

Raw file	Scan	Method	Score	m/z
20130708_PS_Phosphotest_6803wt_SDS01	5867	ITMS; CID	242.86	995.46

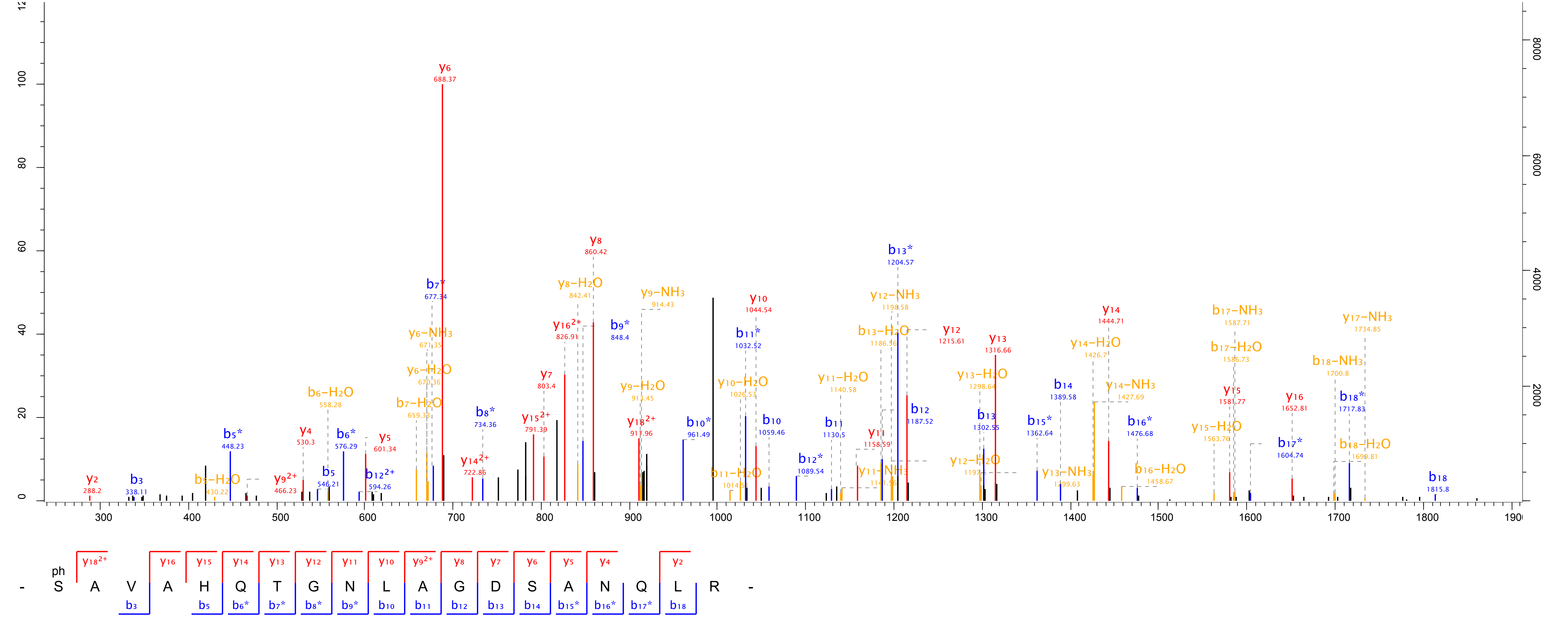

Raw file	Scan	Method	Score	m/z
20130708_PS_Phosphotest_6803wt_SDS01	5874	ITMS; CID	160.67	583.73

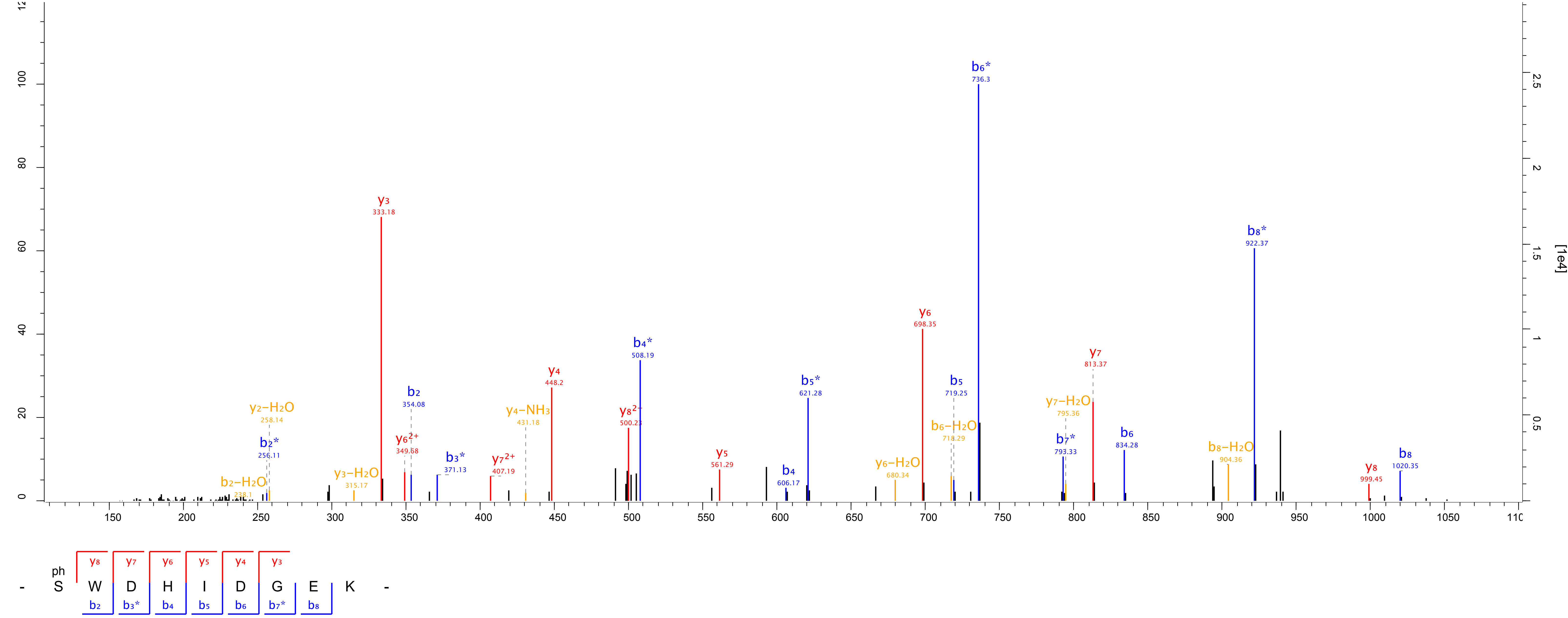

Raw file	Scan	Method	Score	m/z
20130708_PS_Phosphotest_6803wt_SDS01	5904	ITMS; CID	129	516.73

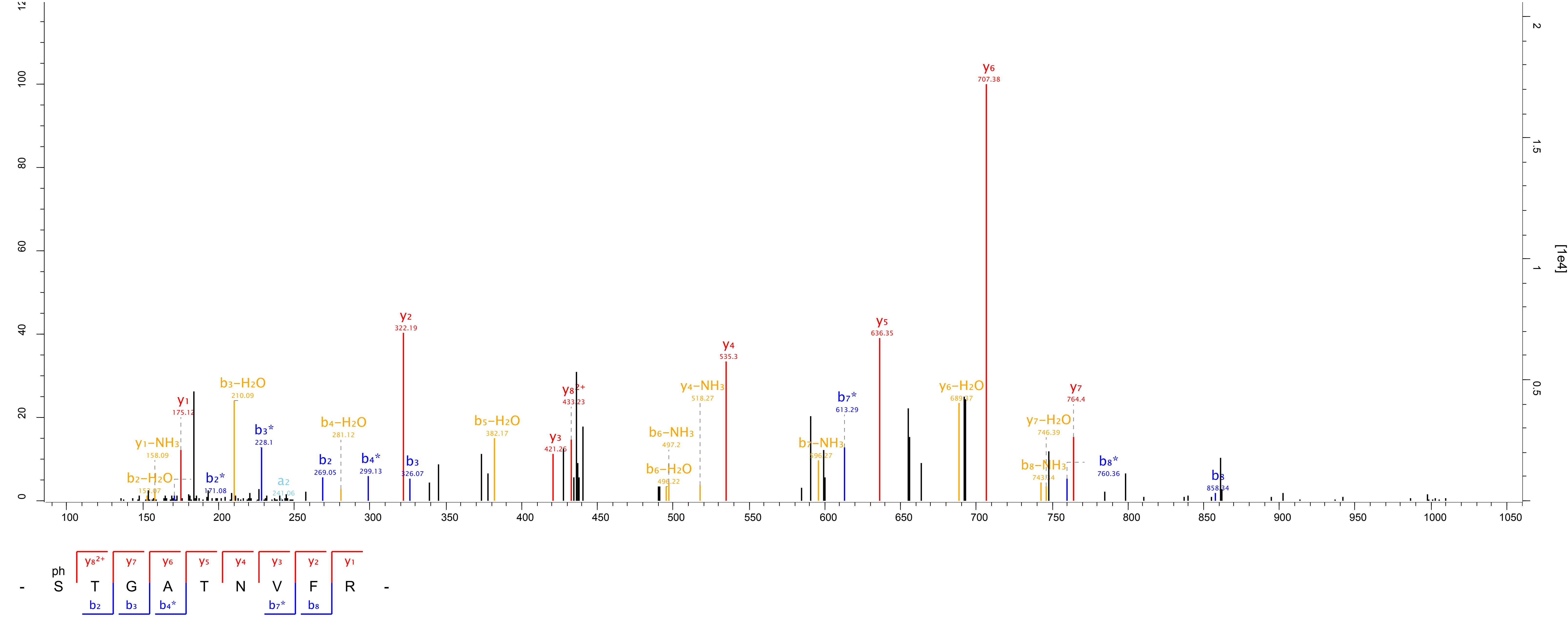

Raw file	Scan	Method	Score	m/z
20130708_PS_Phosphotest_6803wt_SDS01	5964	ITMS; CID	142.1	616.74

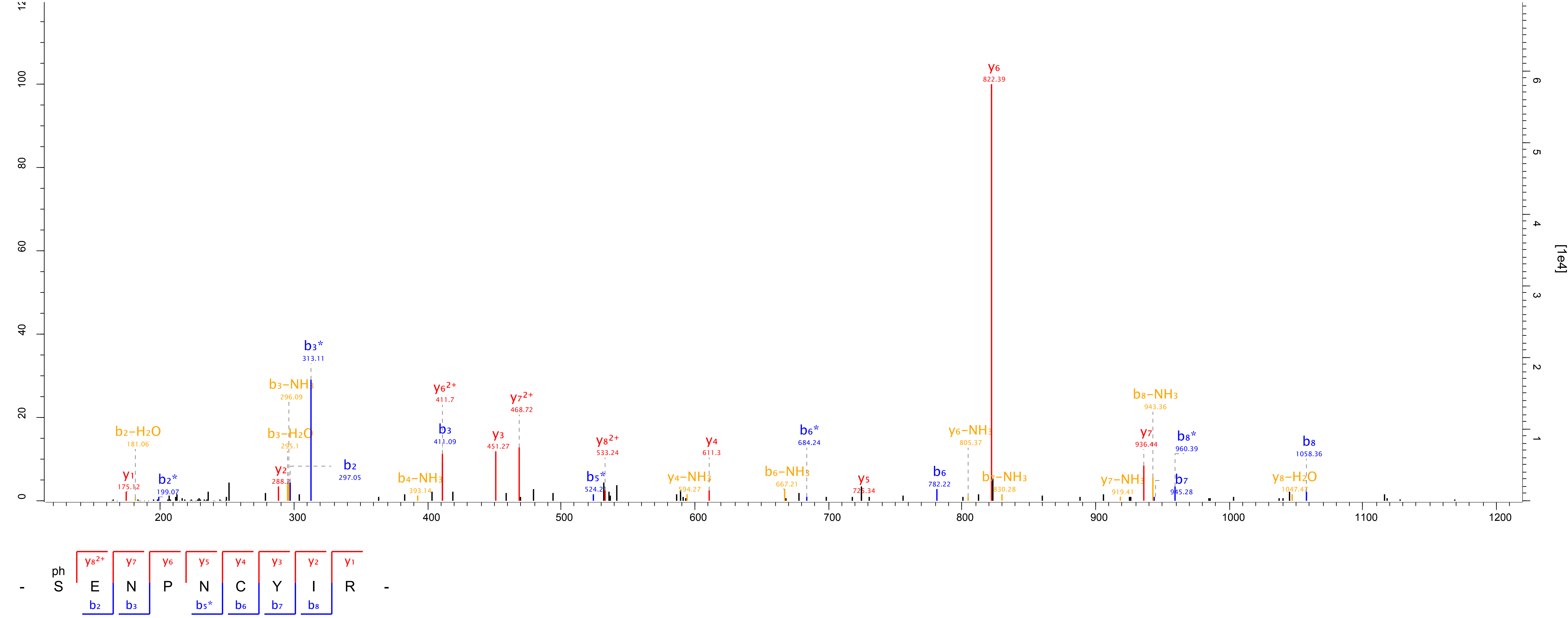

Raw file	Scan	Method	Score	m/z
20130708_PS_Phosphotest_6803wt_SDS01	5966	ITMS; CID	107.73	668.34

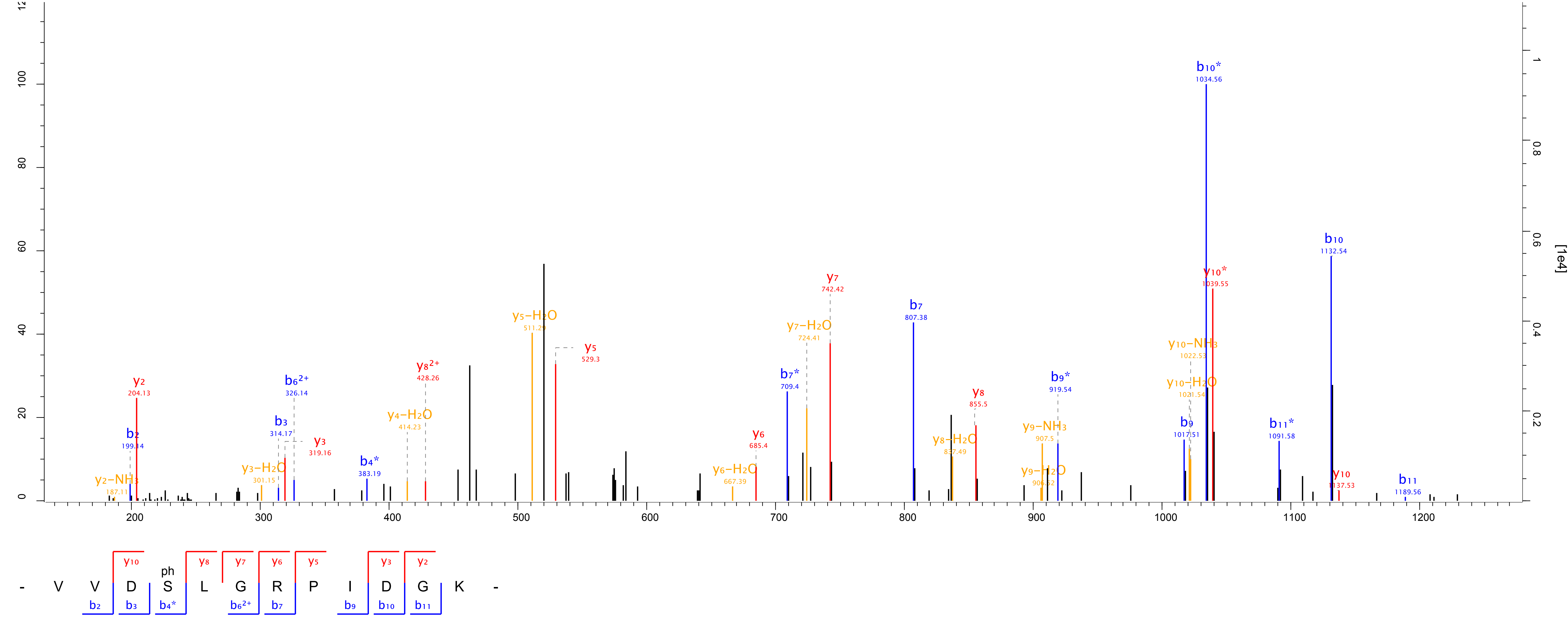

Raw file	Scan	Method	Score	m/z
20130708_PS_Phosphotest_6803wt_SDS01	6023	ITMS; CID	85.16	409.2

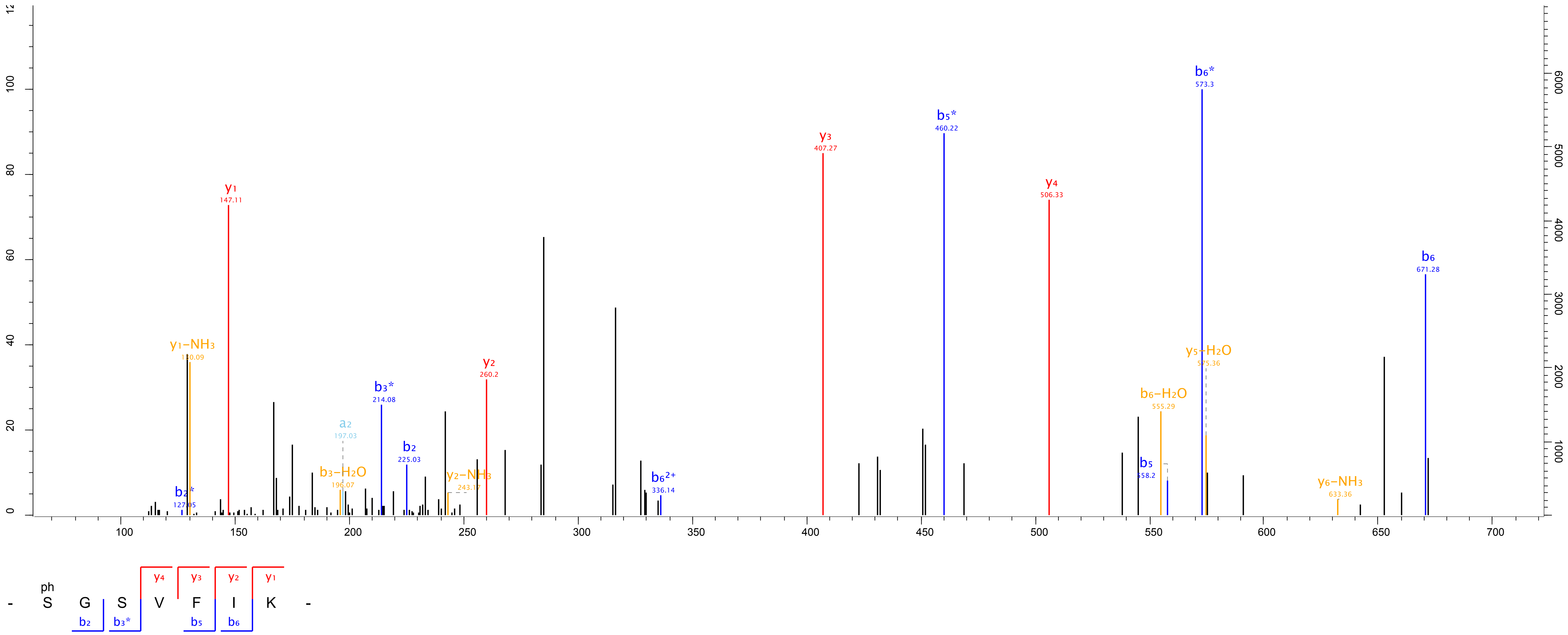

Raw file	Scan	Method	Score	m/z
20130708_PS_Phosphotest_6803wt_SDS03	6061	ITMS; CID	83.95	400.19

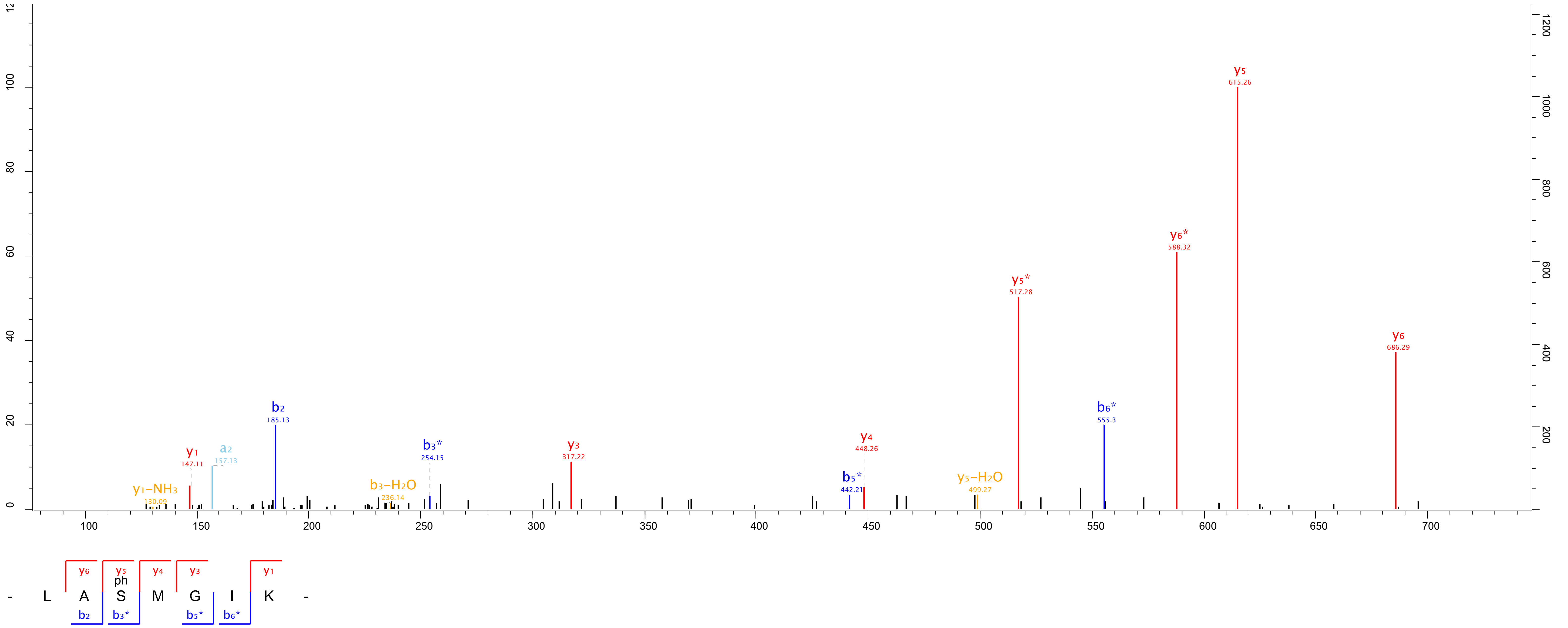

Raw file	Scan	Method	Score	m/z
20130708_PS_Phosphotest_6803wt_SDS01	6185	ITMS; CID	109.83	412.72

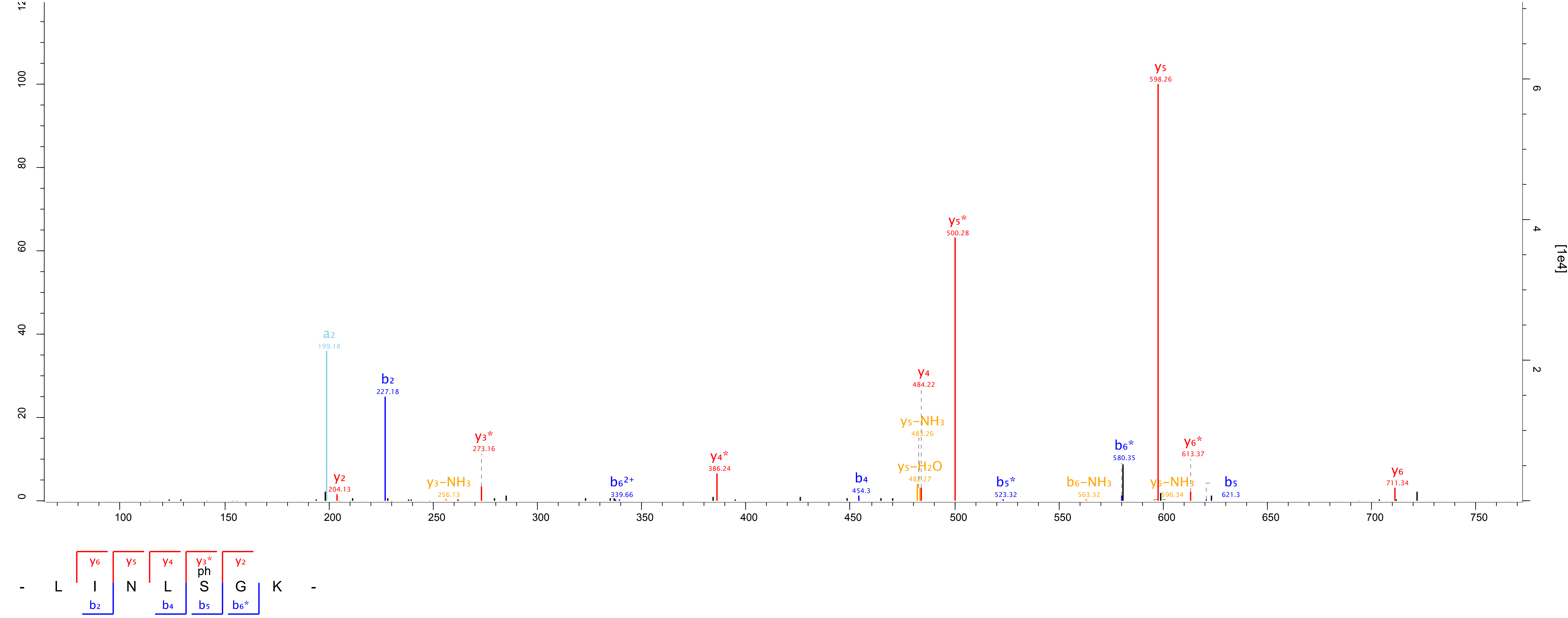

Raw file	Scan	Method	Score	m/z
20130708_PS_Phosphotest_6803wt_YPER01	6198	ITMS; CID	157.07	710.8

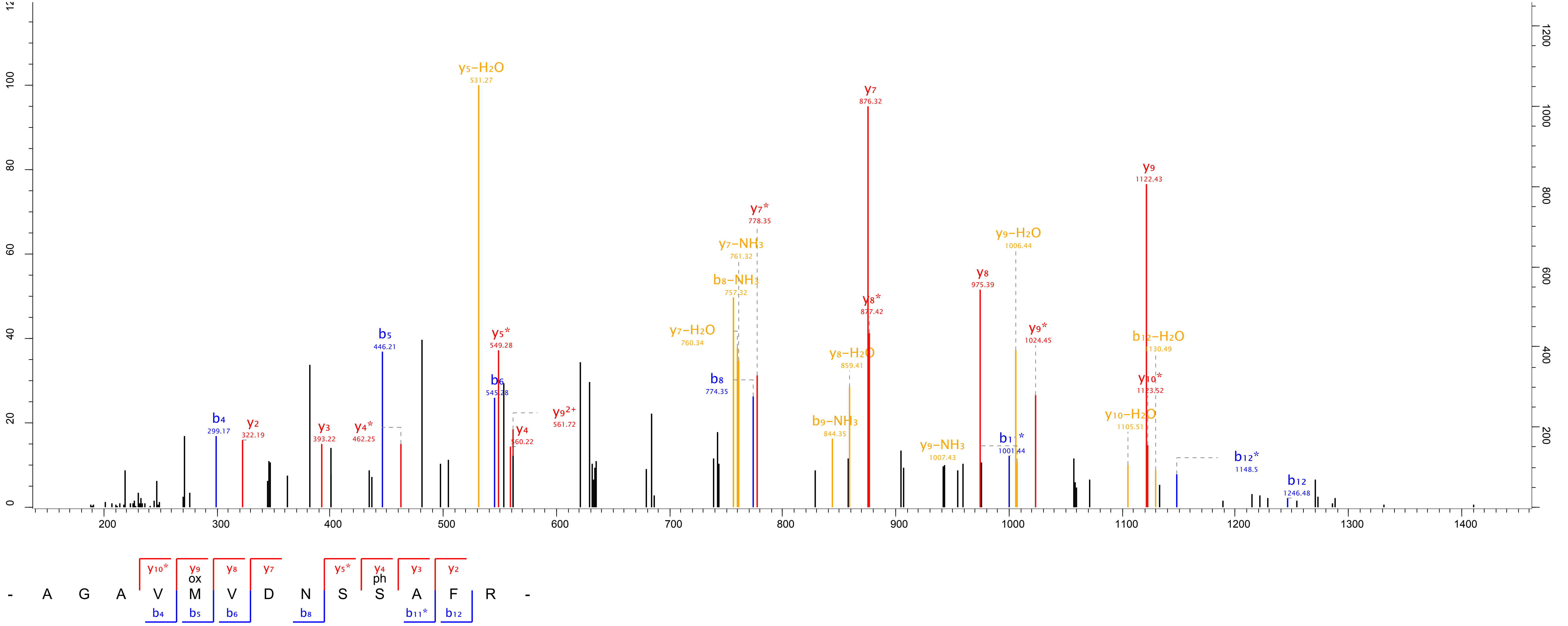

Raw file	Scan	Method	Score	m/z
20130708_PS_Phosphotest_6803wt_SDS01	6217	ITMS; CID	98.55	713.31

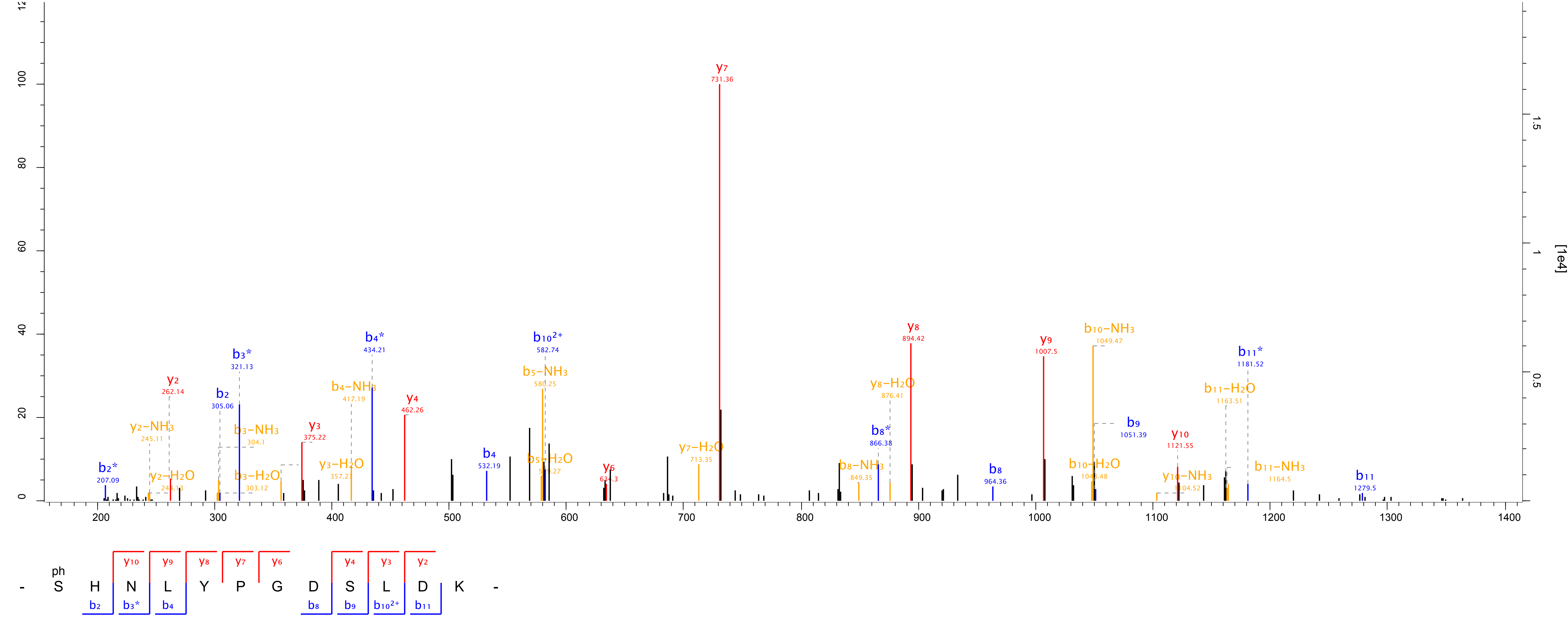

Raw file	Scan	Method	Score	m/z
20130708_PS_Phosphotest_6803wt_SDS01	6221	ITMS; CID	164.93	605.8

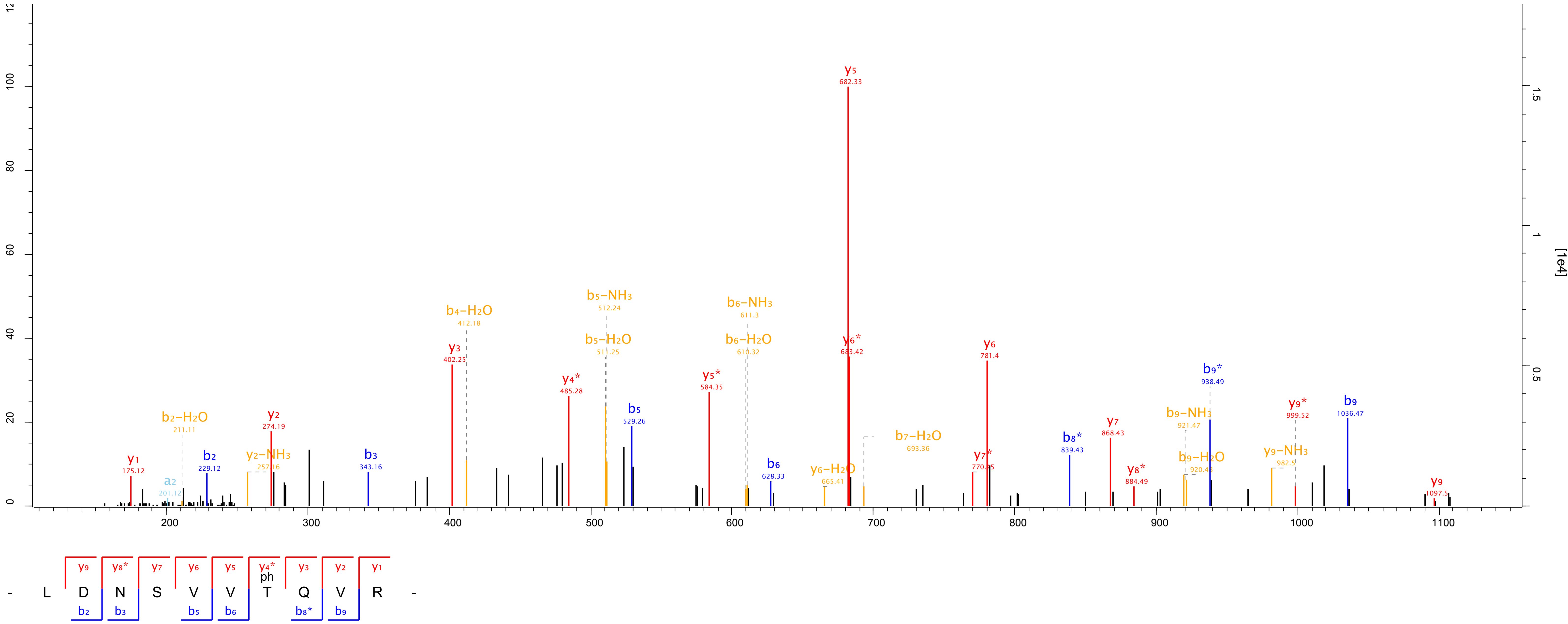

Raw file	Scan	Method	Score	m/z
20130708_PS_Phosphotest_6803wt_YPER01	6296	ITMS; CID	107.09	712.85

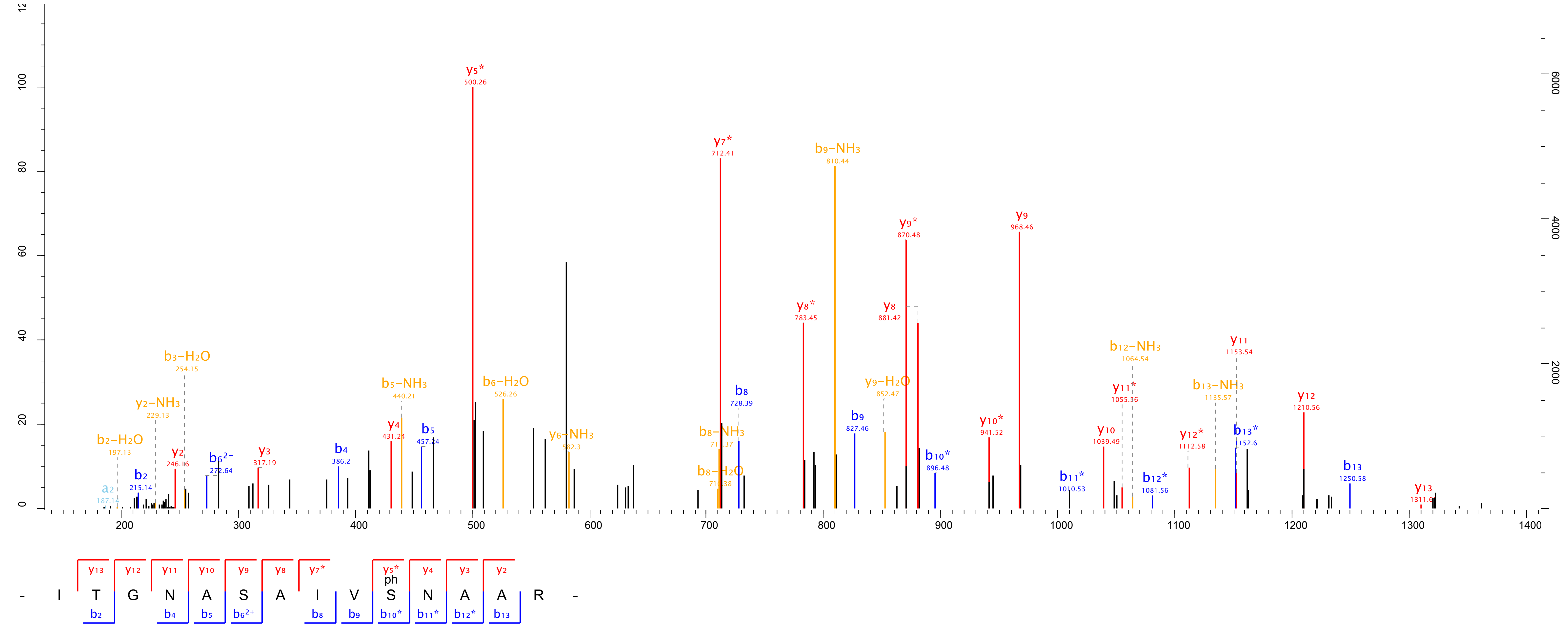

Raw file	Scan	Method	Score	m/z
20130708_PS_Phosphotest_6803wt_SDS01	6305	ITMS; CID	120.9	581.29

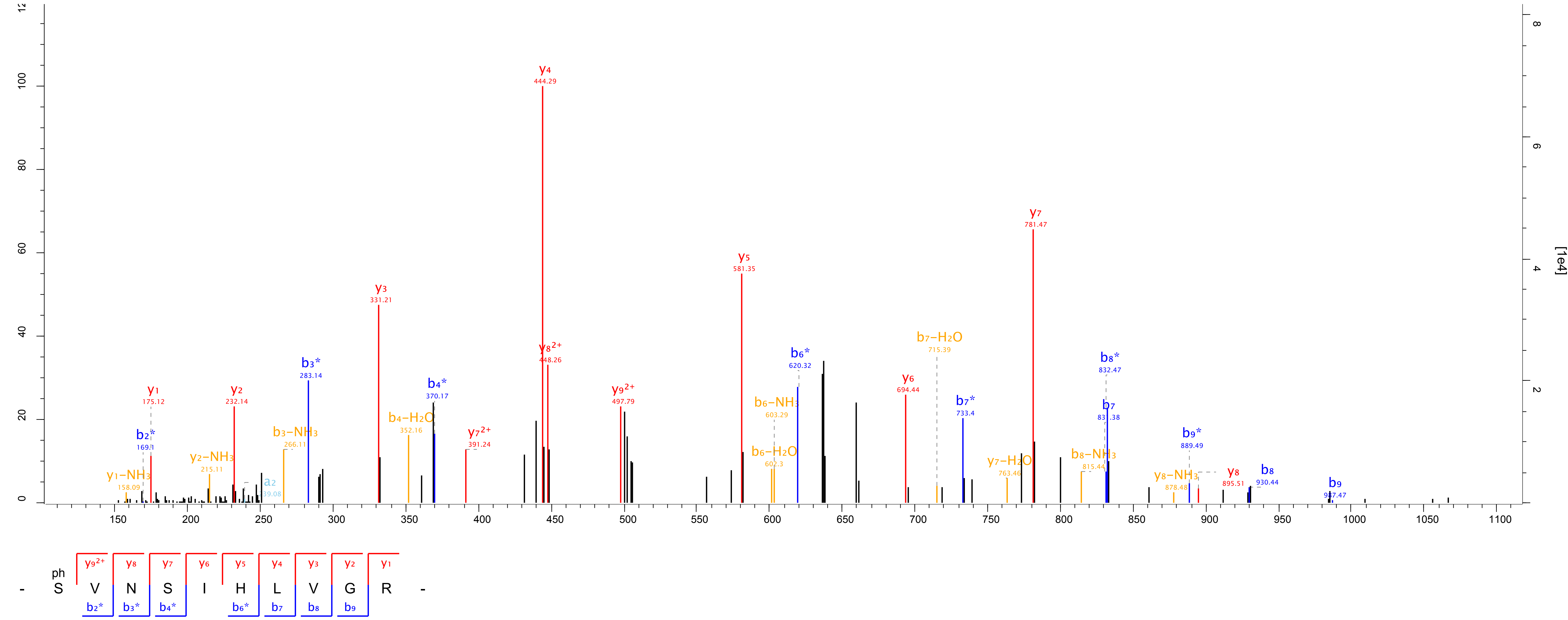

Raw file	Scan	Method	Score	m/z
20130708_PS_Phosphotest_6803wt_SDS01	6327	ITMS; CID	118.52	605.8

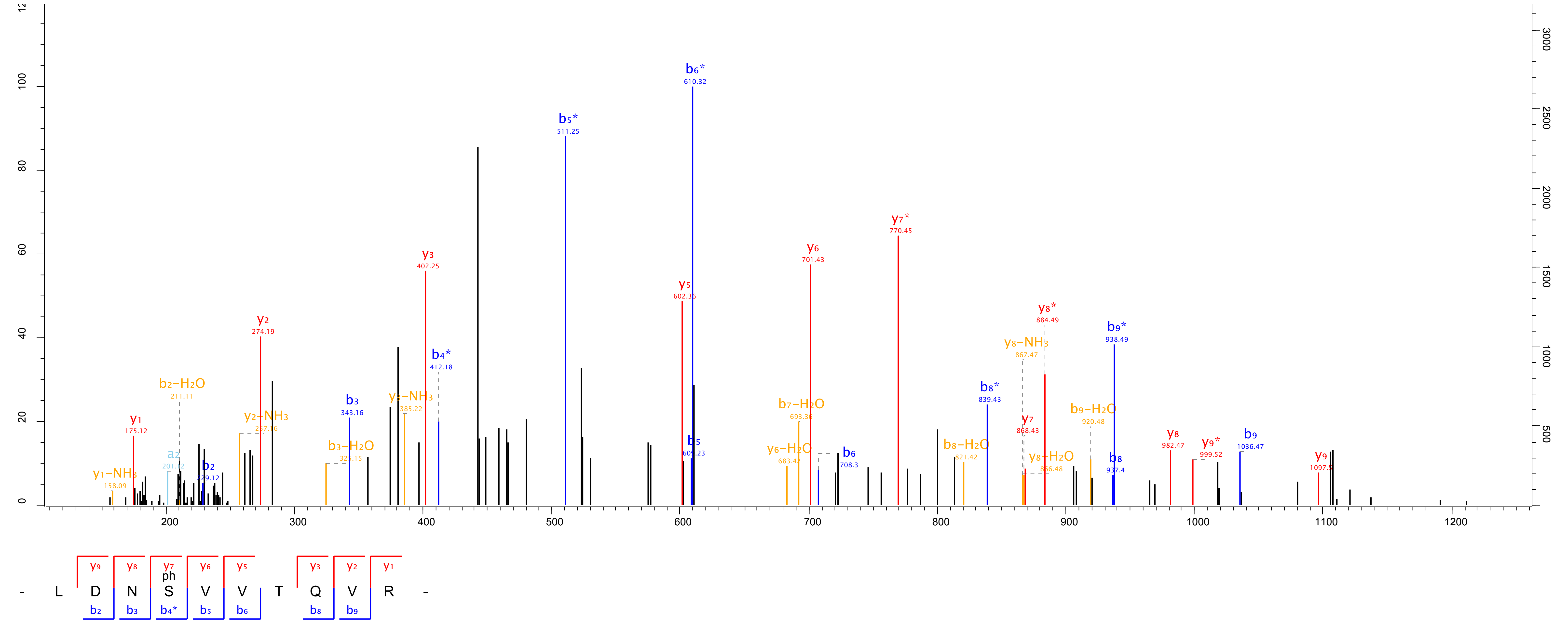

Raw file	Scan	Method	Score	m/z
20130708_PS_Phosphotest_6803wt_SDS01	6399	ITMS; CID	188.34	979.43

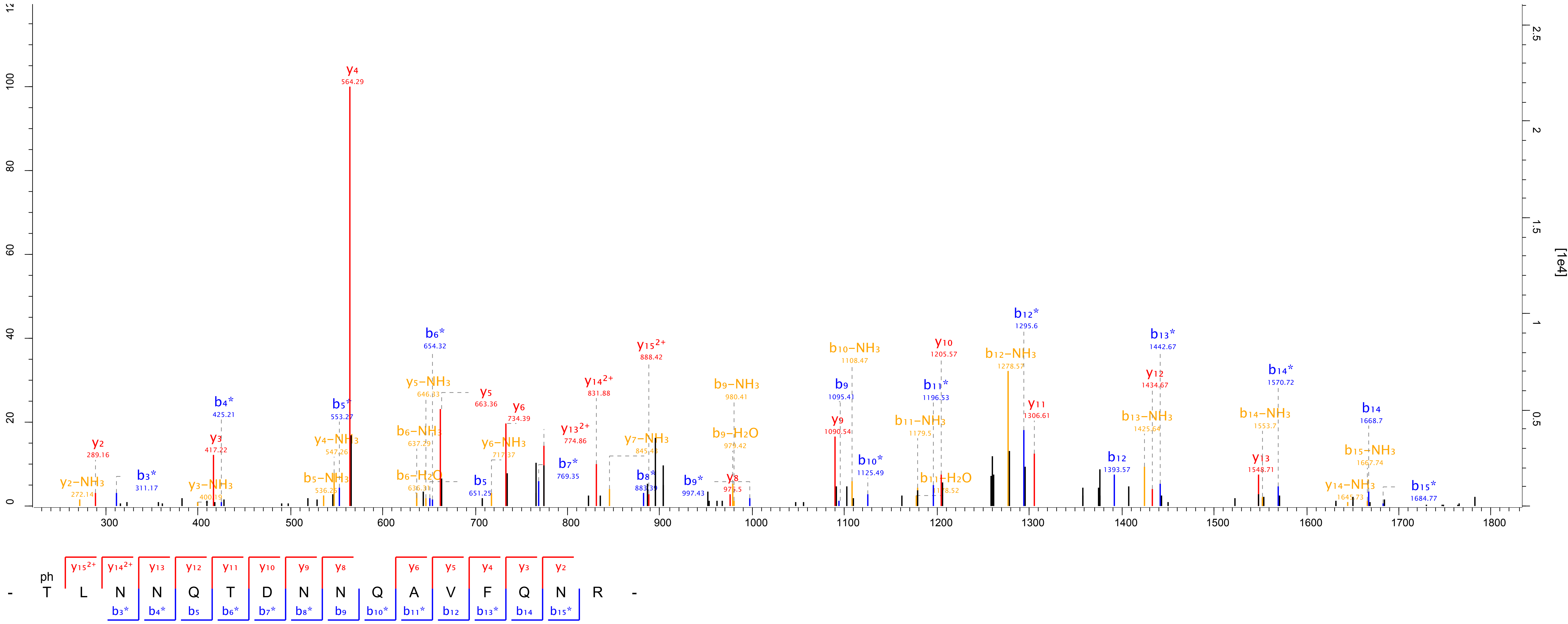

Raw file	Scan	Method	Score	m/z
20130708_PS_Phosphotest_6803wt_YPER01	6414	ITMS; CID	191.41	799.42

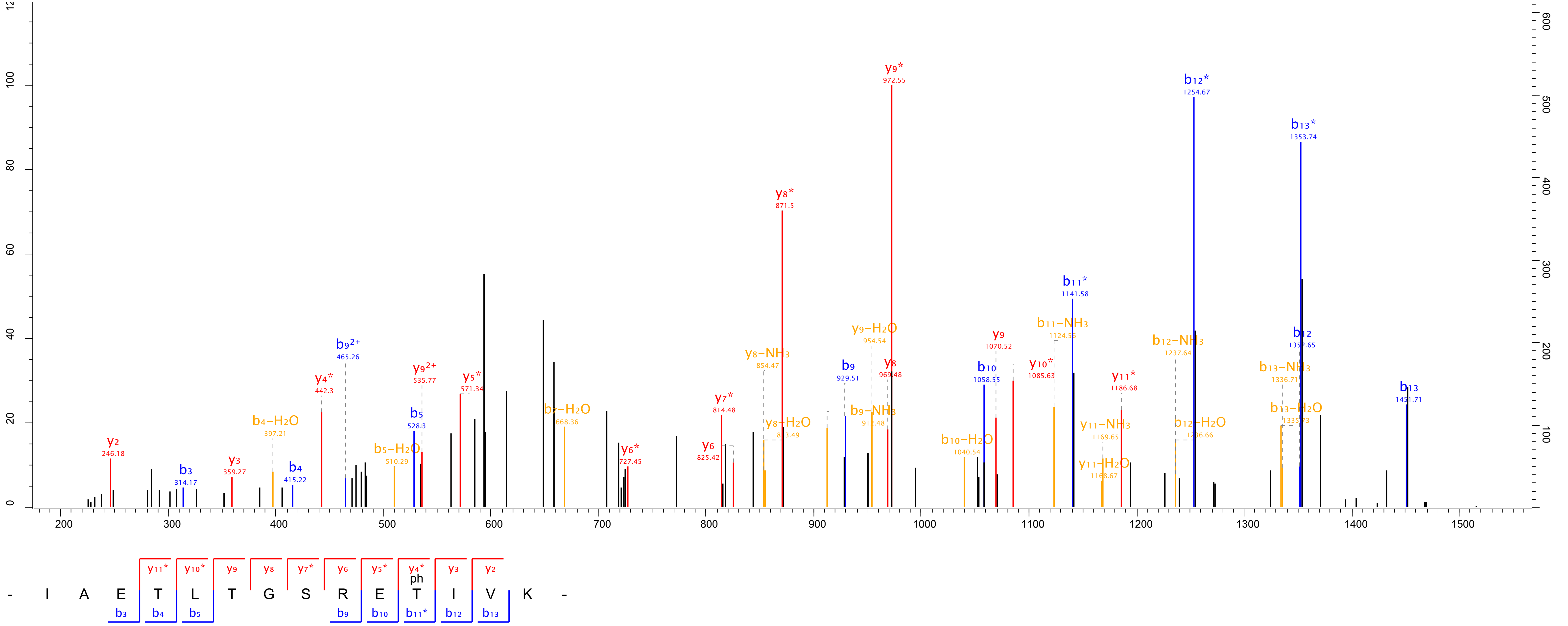

Raw file	Scan	Method	Score	m/z
20130708_PS_Phosphotest_6803wt_SDS01	6457	ITMS; CID	86.46	484.25

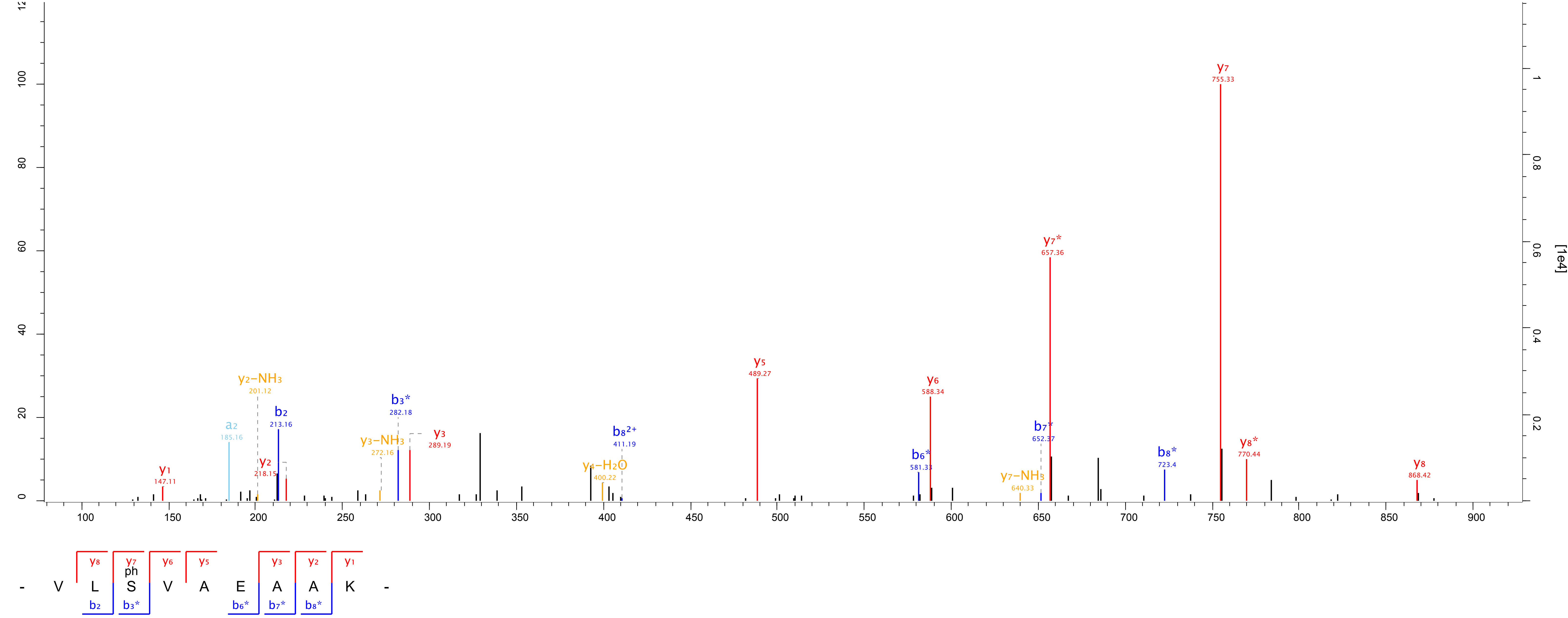

Raw file	Scan	Method	Score	m/z
20130708_PS_Phosphotest_6803wt_SDS01	6481	ITMS; CID	95.43	676.27

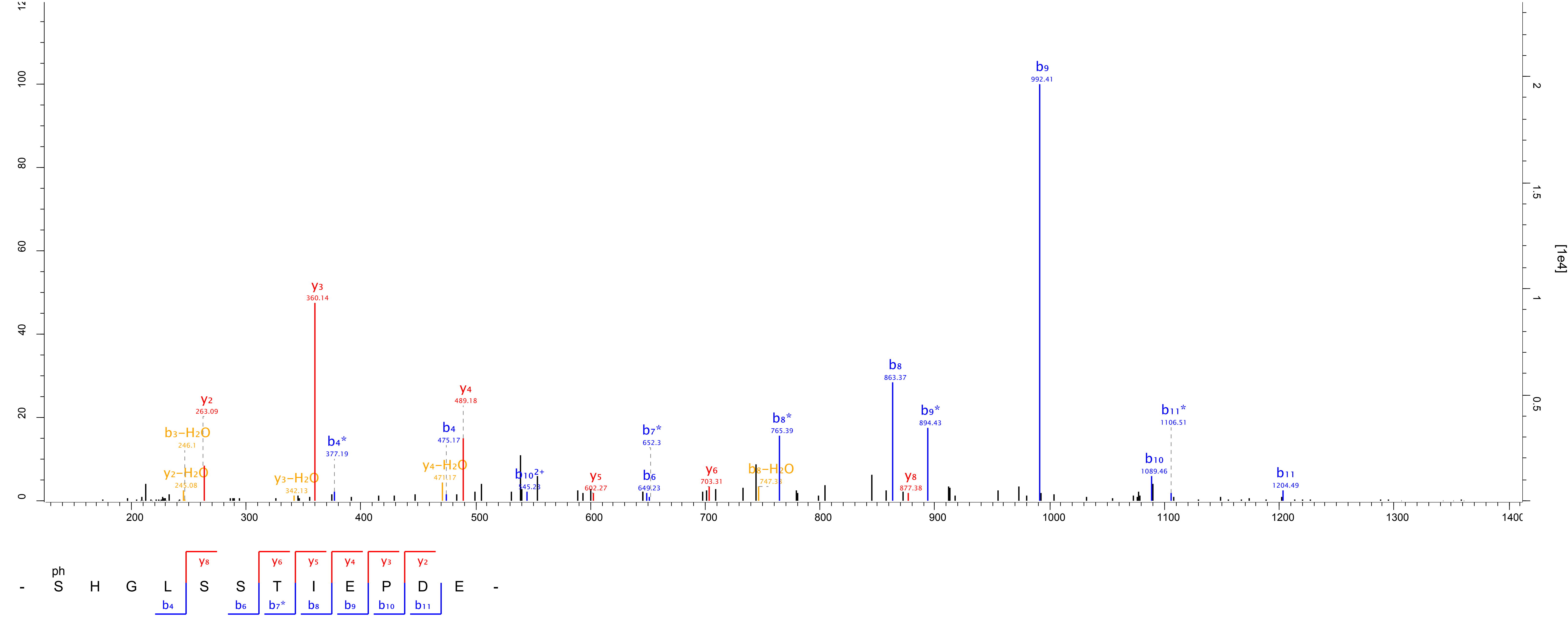

Raw file	Scan	Method	Score	m/z
20130708_PS_Phosphotest_6803wt_SDS01	6503	ITMS; CID	187.52	811.88

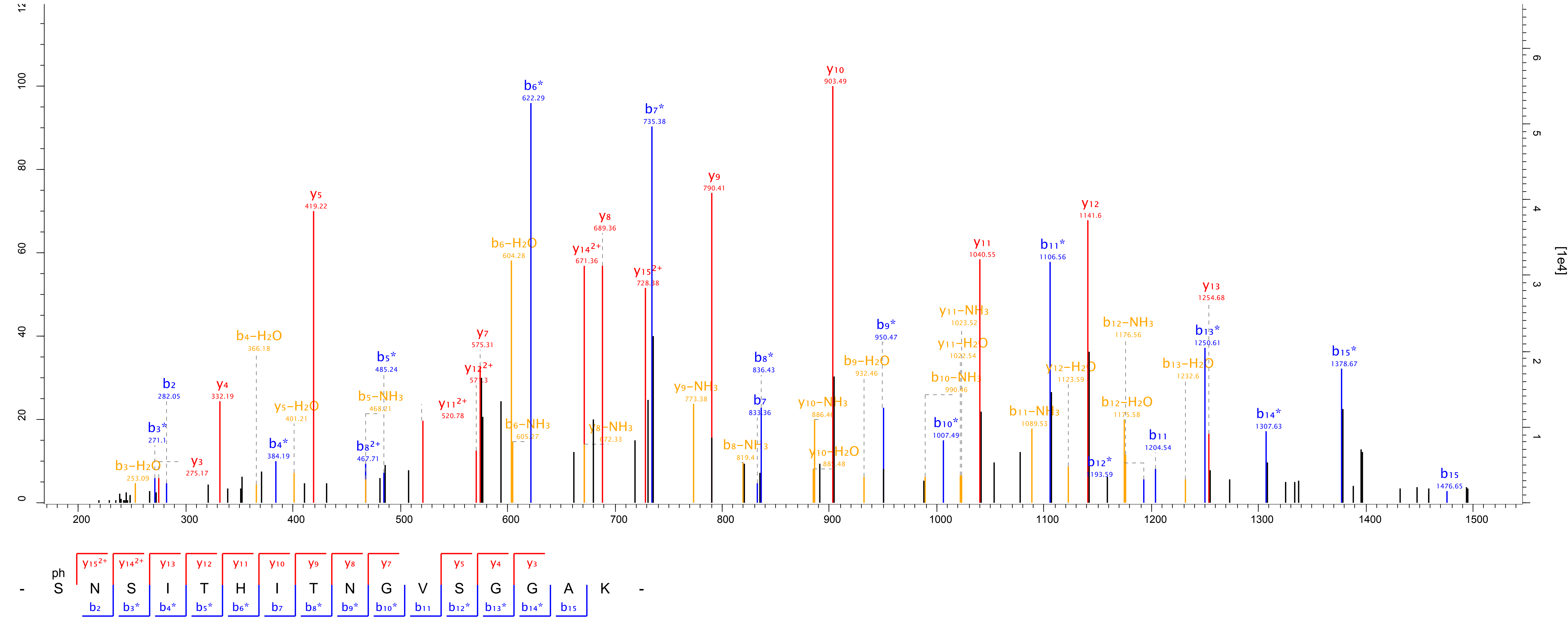

Raw file	Scan	Method	Score	m/z
20130708_PS_Phosphotest_6803wt_SDS01	6565	ITMS; CID	146.59	698.34

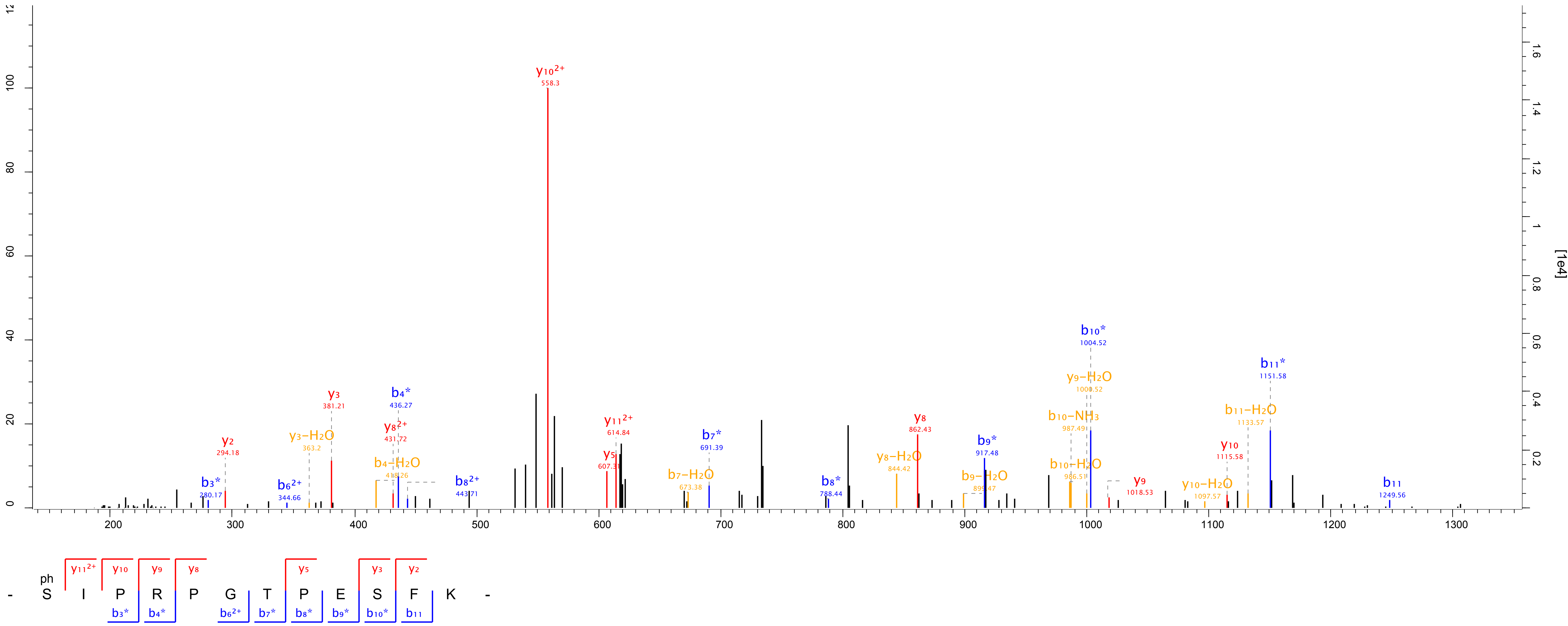

Raw file	Scan	Method	Score	m/z
20130708_PS_Phosphotest_6803wt_SDS01	6608	ITMS; CID	89.75	599.3

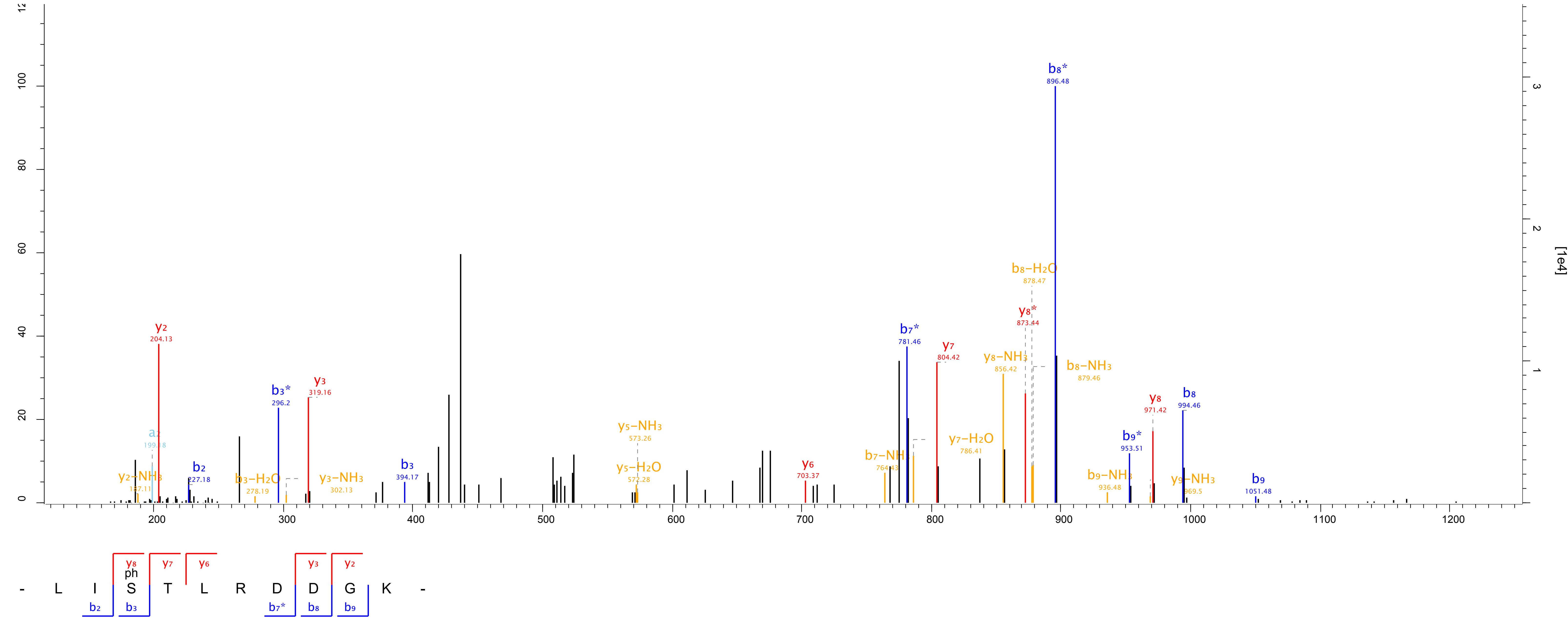

Raw file	Scan	Method	Score	m/z
20130708_PS_Phosphotest_6803wt_SDS01	6620	ITMS; CID	130.47	762.38

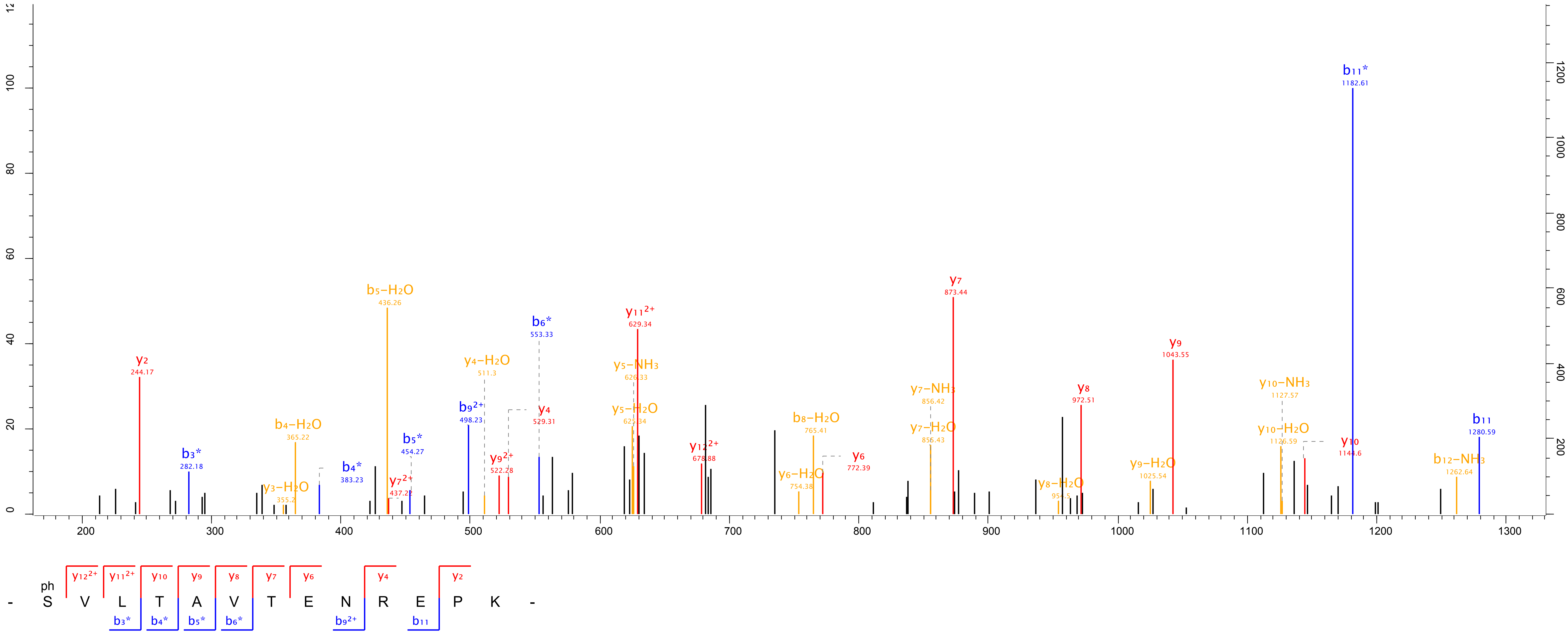

Raw file	Scan	Method	Score	m/z
20130708_PS_Phosphotest_6803wt_SDS01	6656	ITMS; CID	105.99	613.77

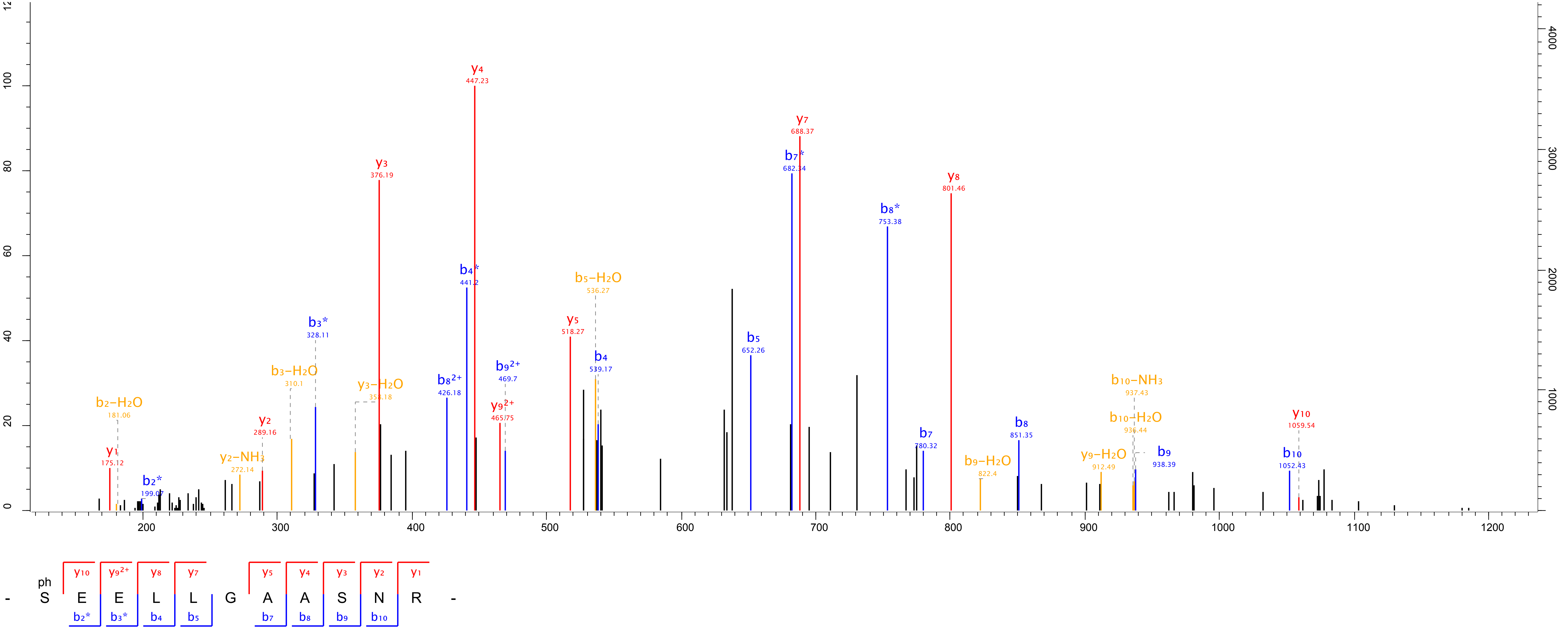

Raw file	Scan	Method	Score	m/z
20130708_PS_Phosphotest_6803wt_SDS01	6657	ITMS; CID	116.19	596.78

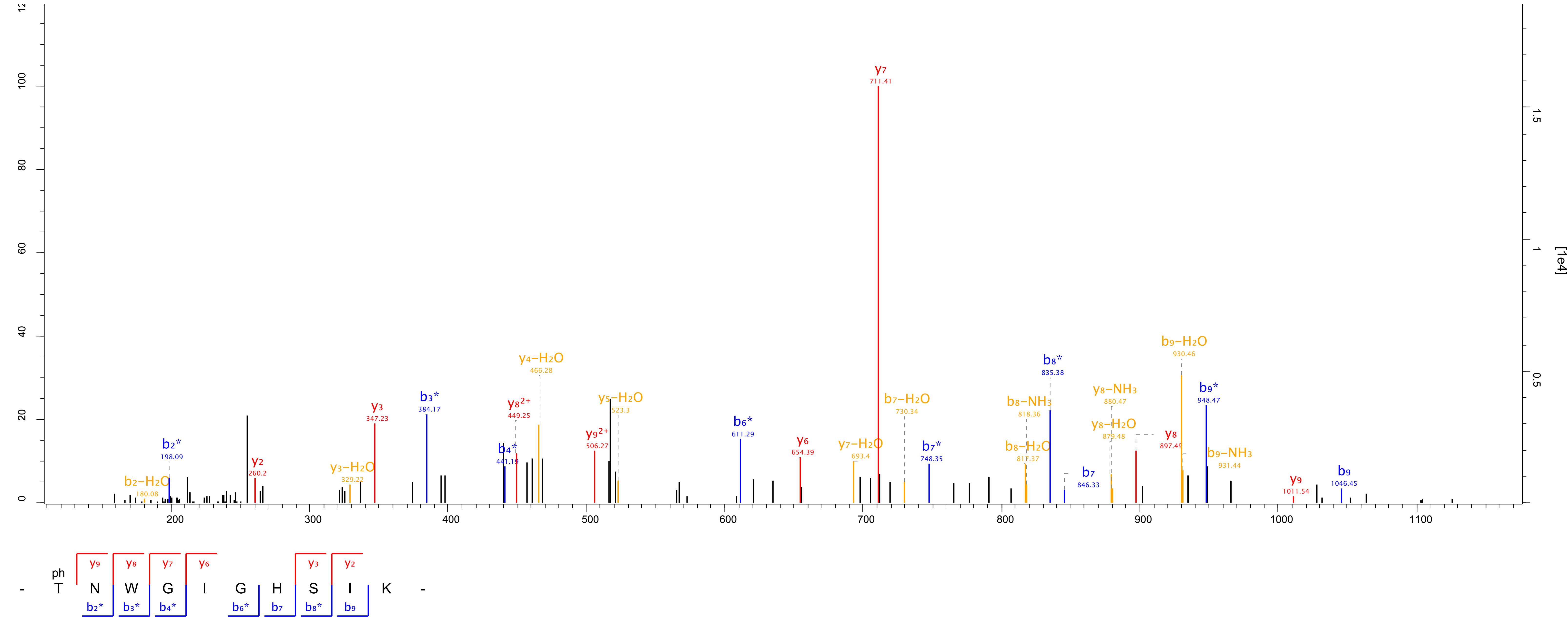

Raw file	Scan	Method	Score	m/z
20130708_PS_Phosphotest_6803wt_SDS01	6665	ITMS; CID	172.09	693.3

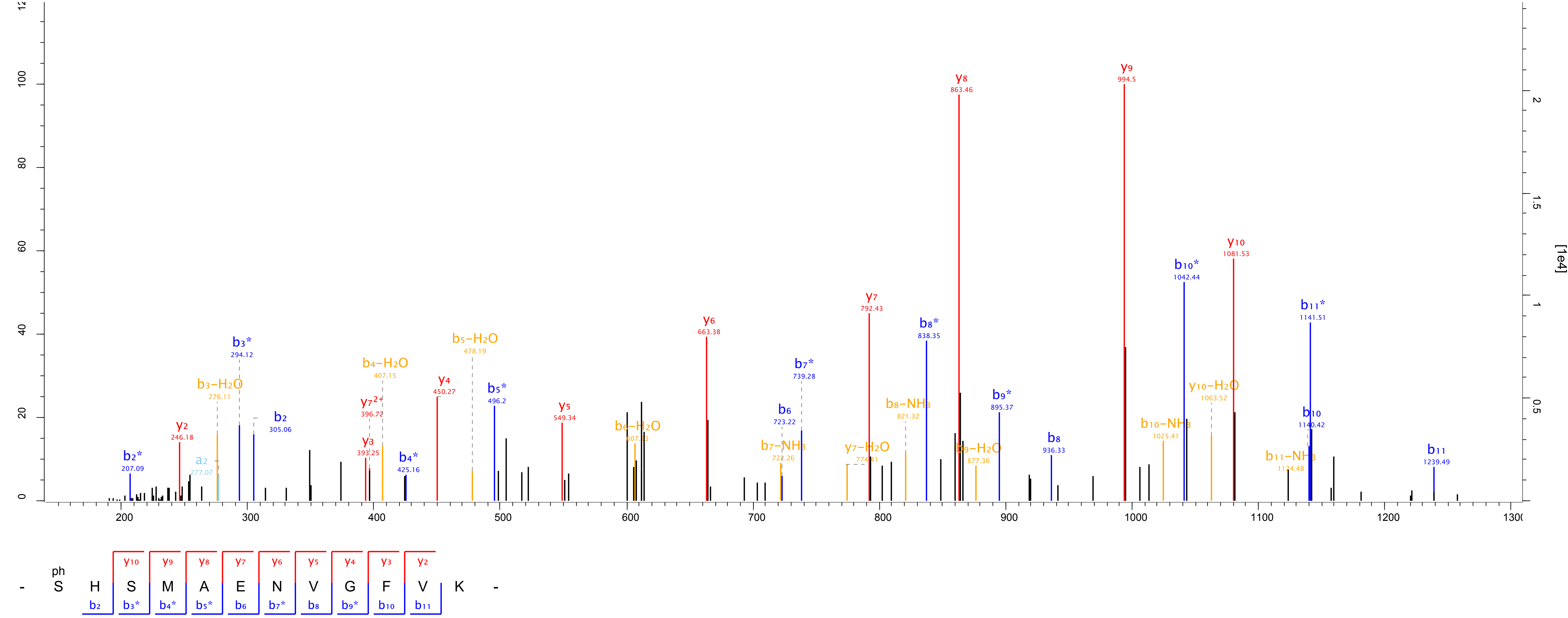

Raw file	Scan	Method	Score	m/z
20130708_PS_Phosphotest_6803wt_YPER01	6696	ITMS; CID	190.66	688.82

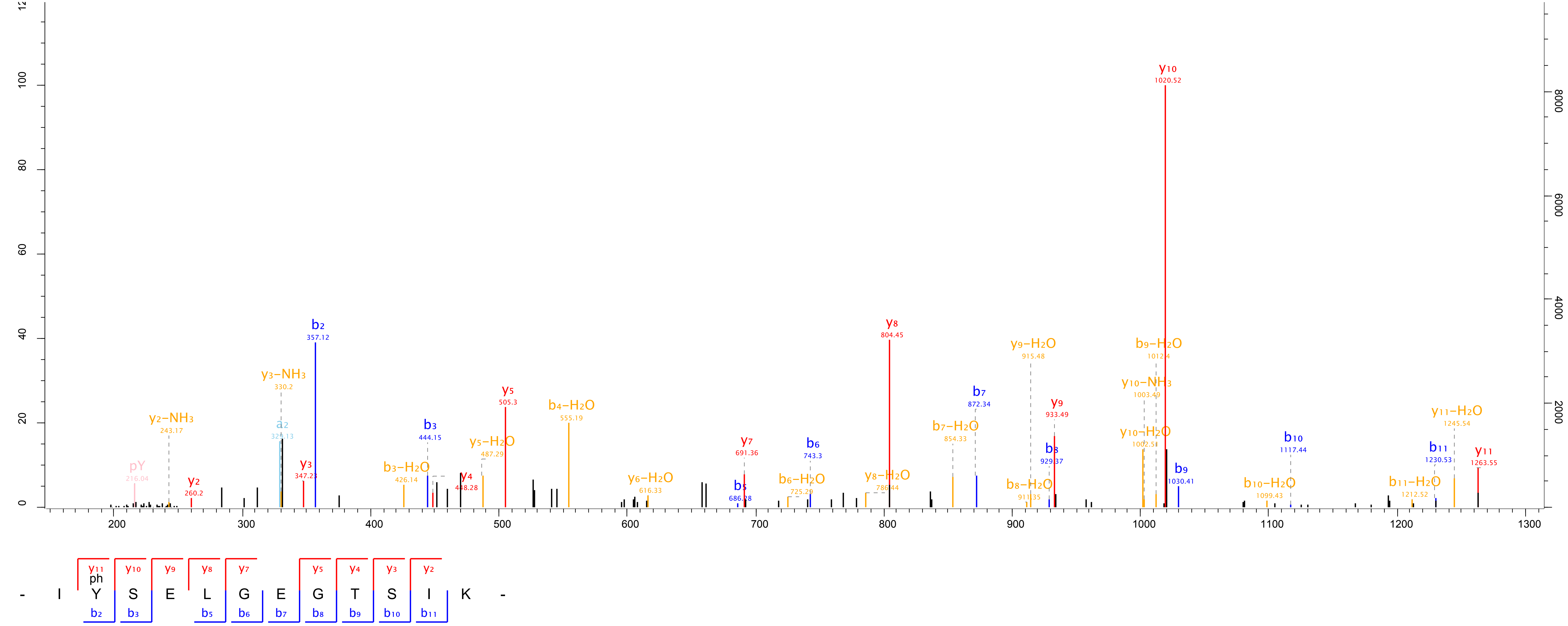

Raw file	Scan	Method	Score	m/z
20130708_PS_Phosphotest_6803wt_SDS01	6716	ITMS; CID	167.18	692.83

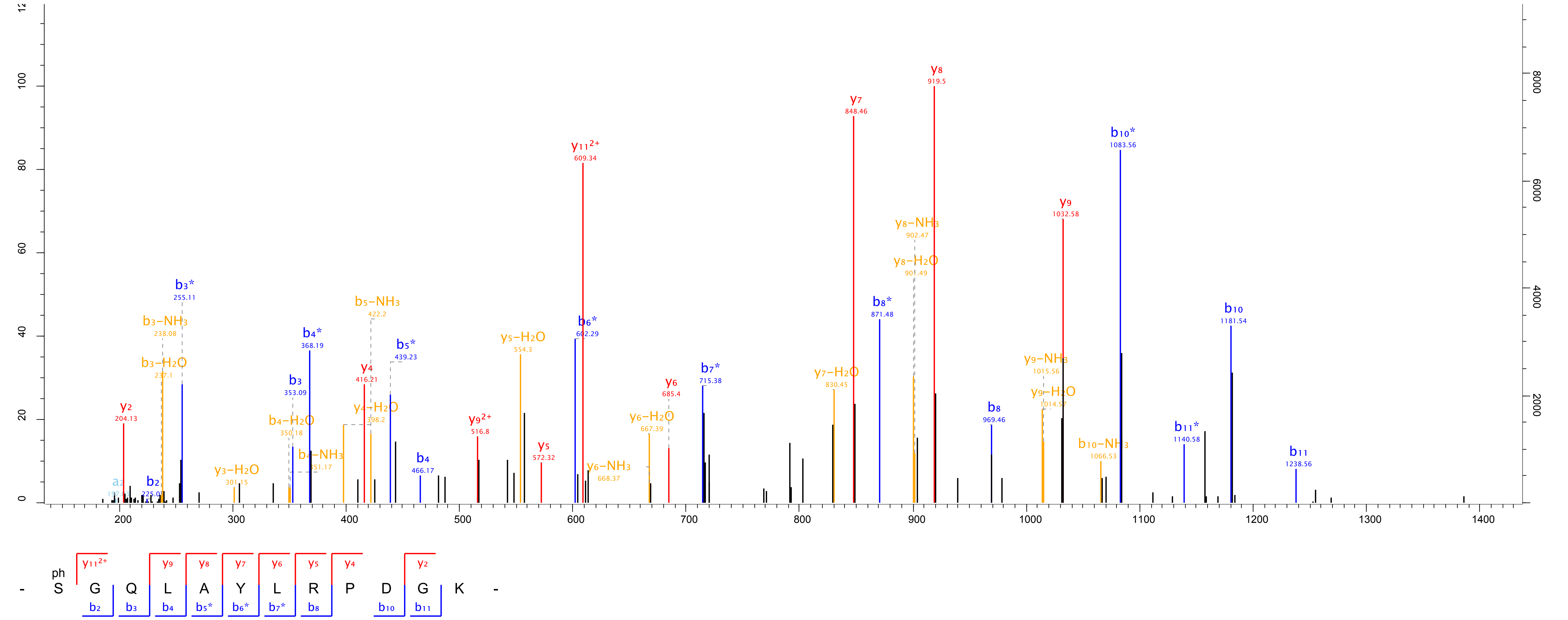

Raw file	Scan	Method	Score	m/z
20130708_PS_Phosphotest_6803wt_SDS01	6733	ITMS; CID	115.7	495.74

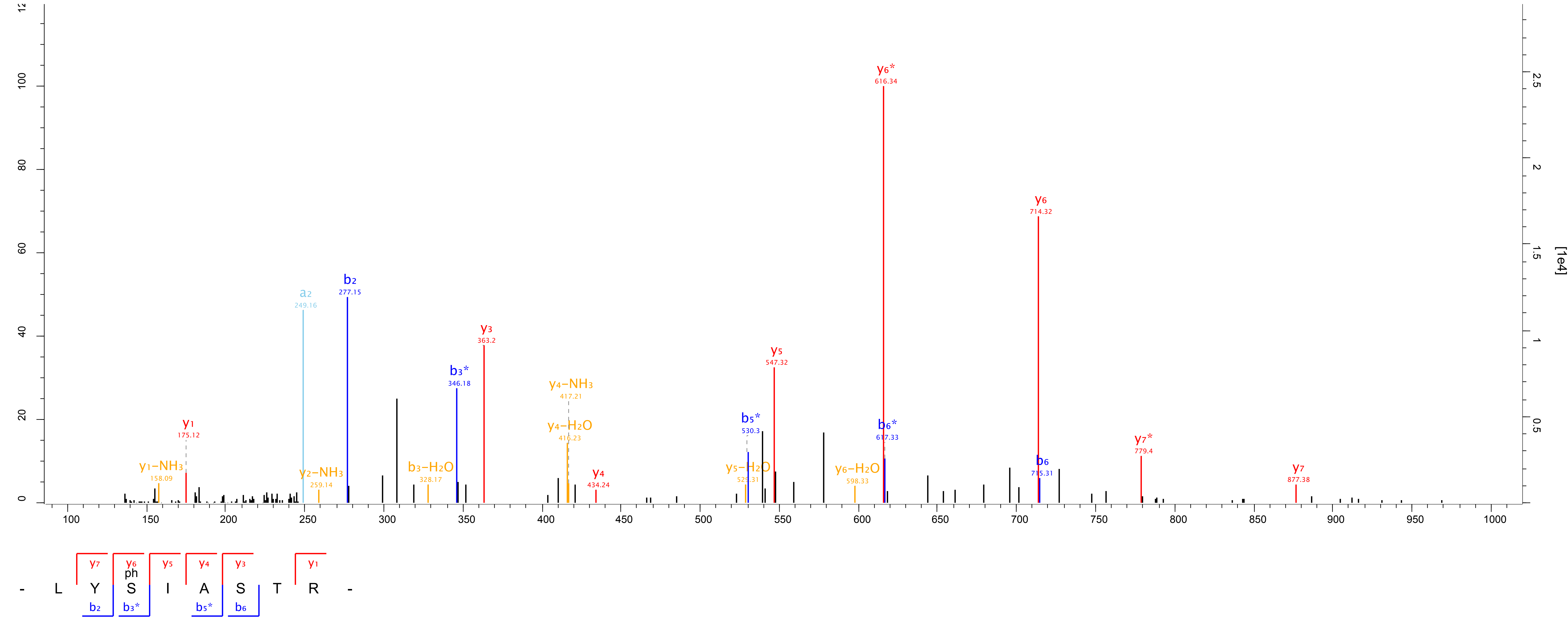

Raw file	Scan	Method	Score	m/z
20130708_PS_Phosphotest_6803wt_YPER01	6748	ITMS; CID	105.66	595.75

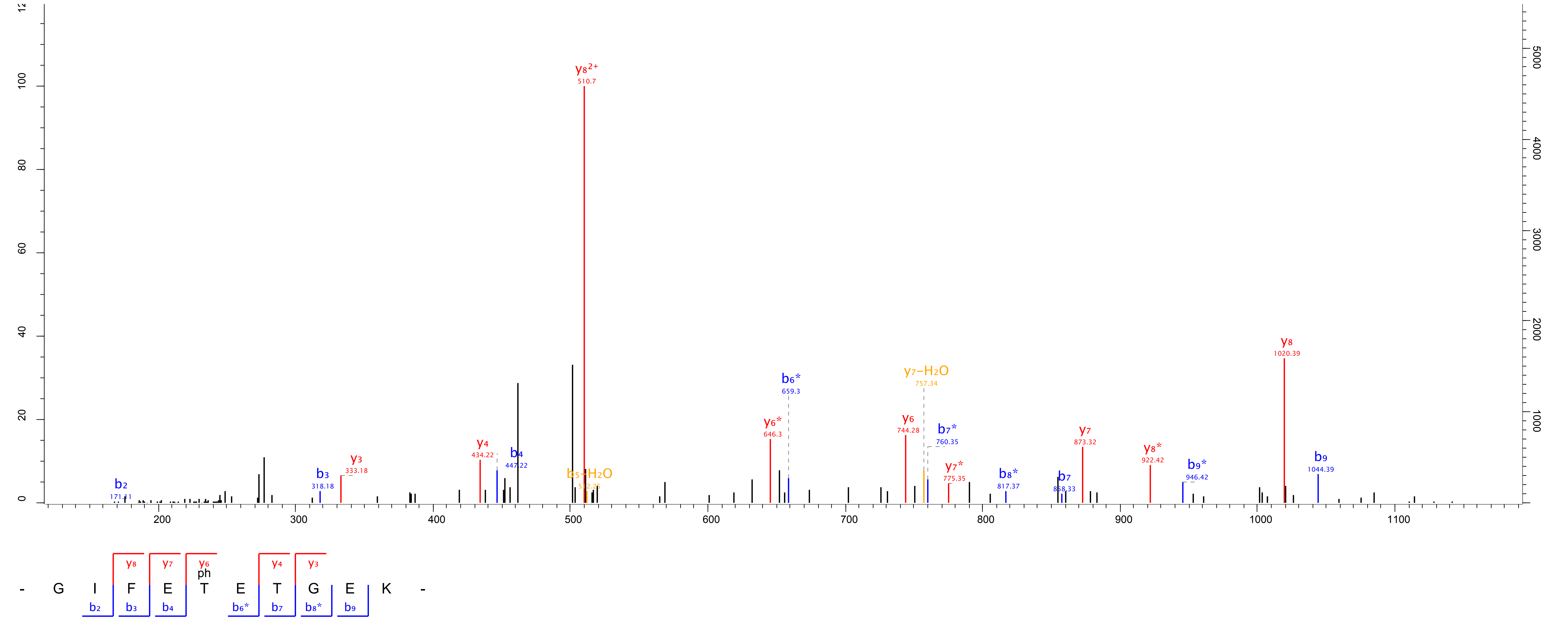

Raw file	Scan	Method	Score	m/z
20130708_PS_Phosphotest_6803wt_YPER01	6760	ITMS; CID	107.46	588.83

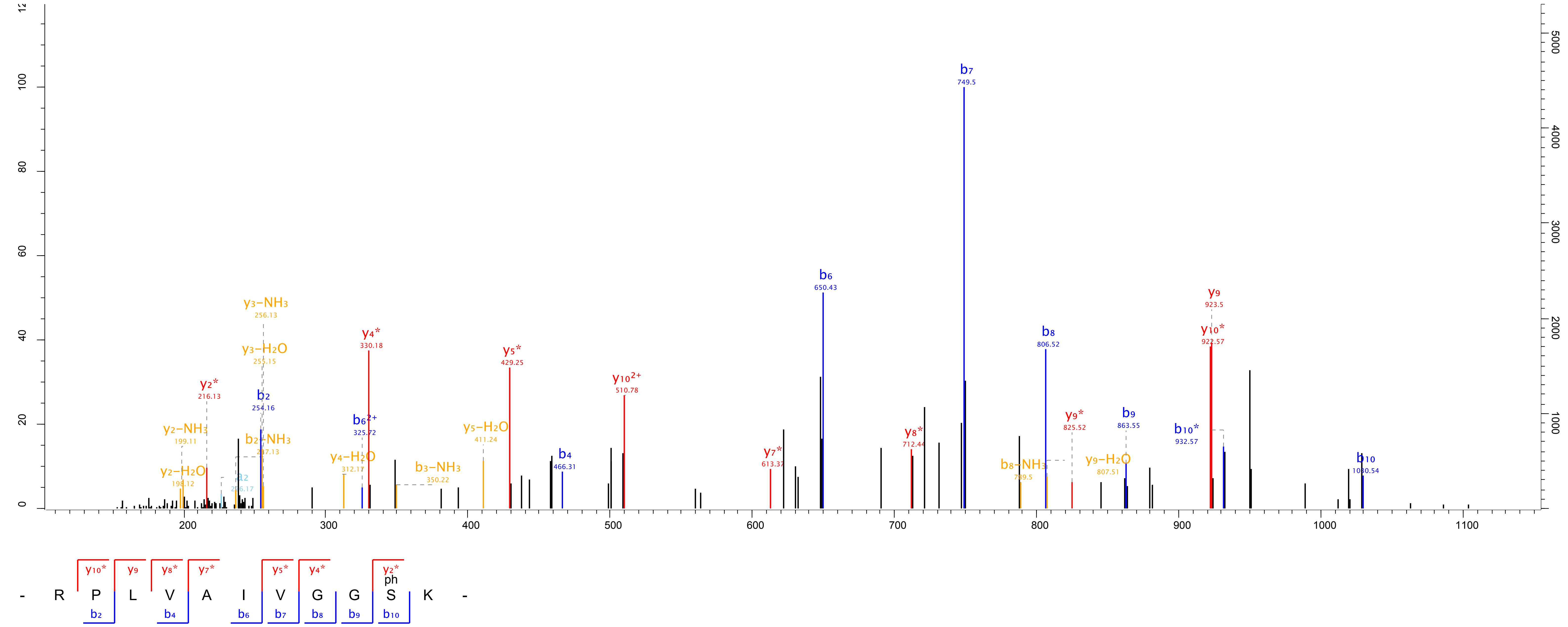

Raw file	Scan	Method	Score	m/z
20130708_PS_Phosphotest_6803wt_YPER02	6792	ITMS; CID	142.97	712.85

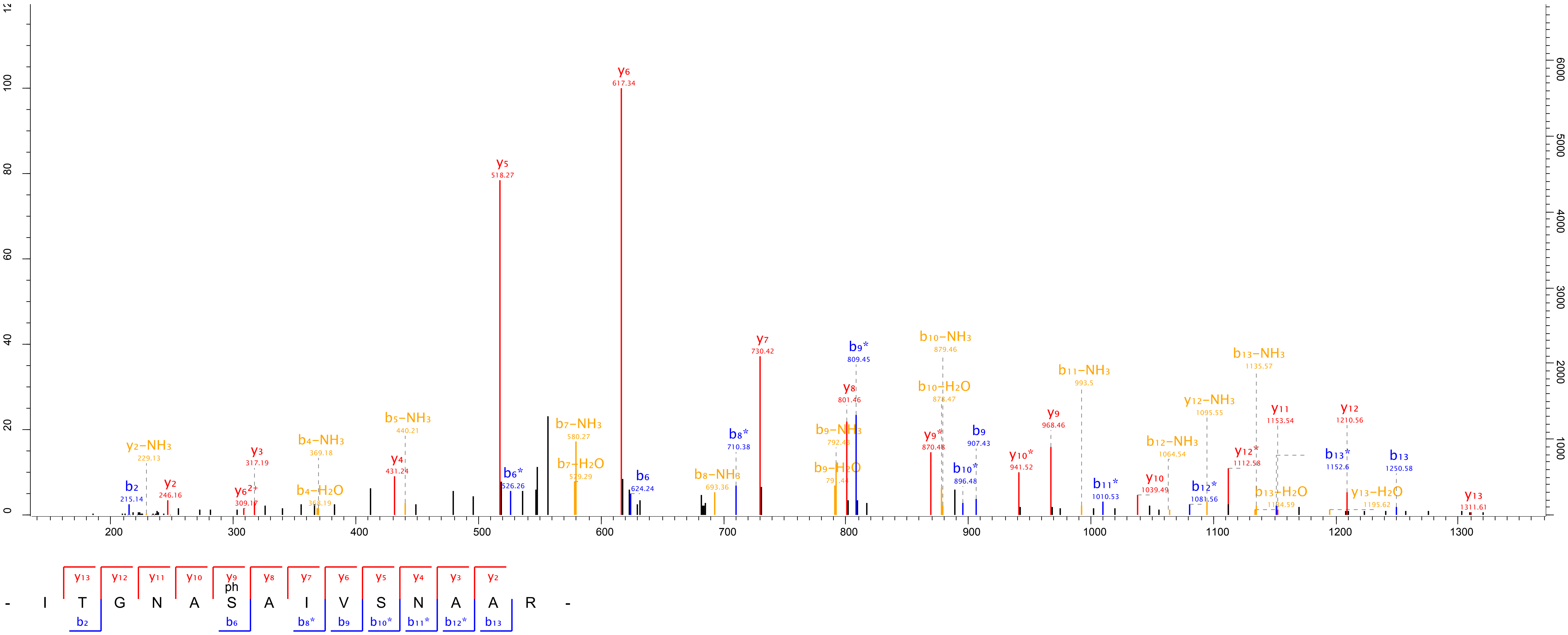

Raw file	Scan	Method	Score	m/z
20130708_PS_Phosphotest_6803wt_YPER01	6850	ITMS; CID	183.28	615.29

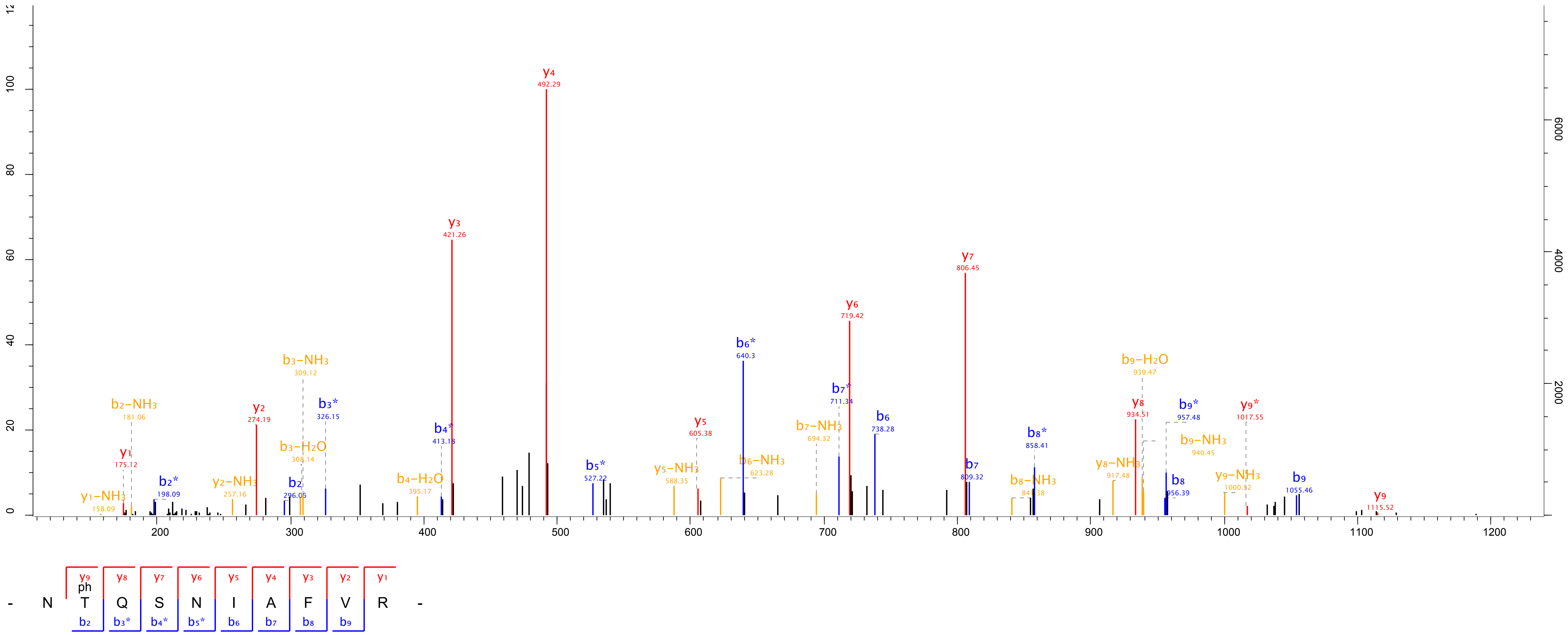

Raw file	Scan	Method	Score	m/z
20130708_PS_Phosphotest_6803wt_SDS01	6884	ITMS; CID	86.77	535.74

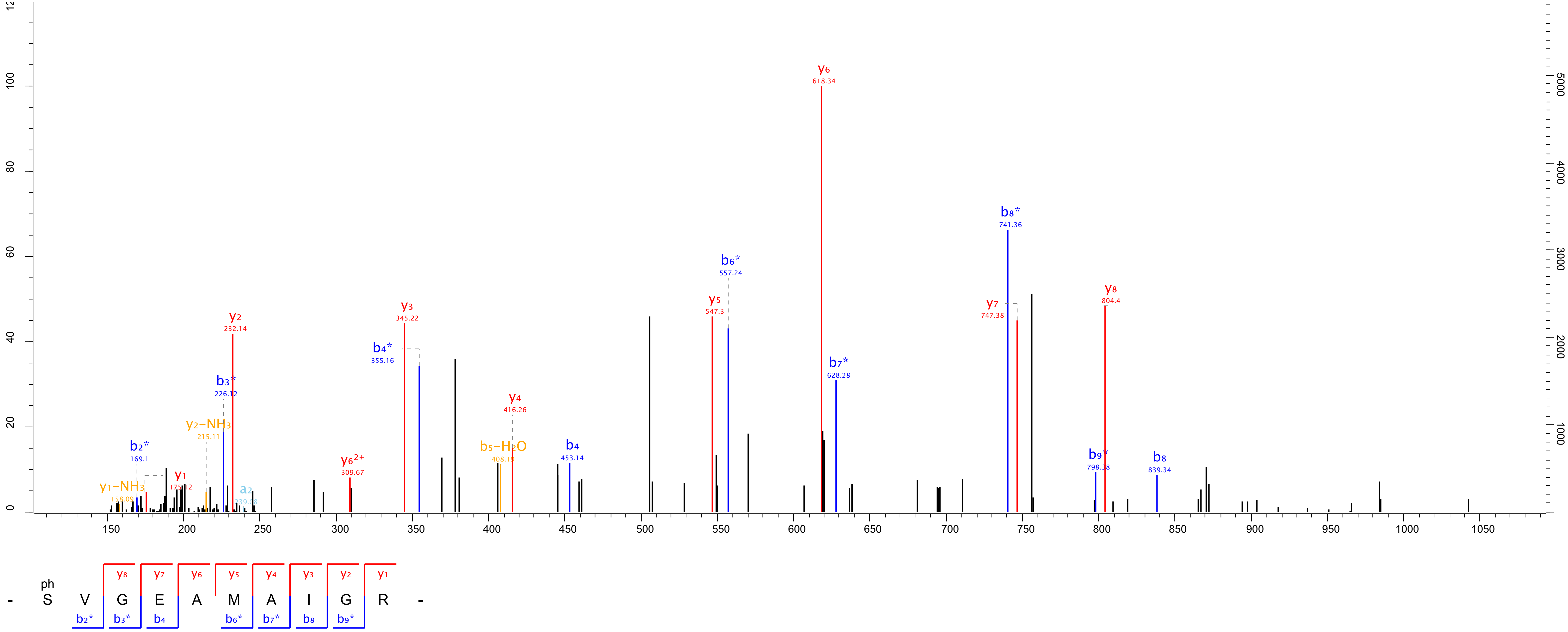

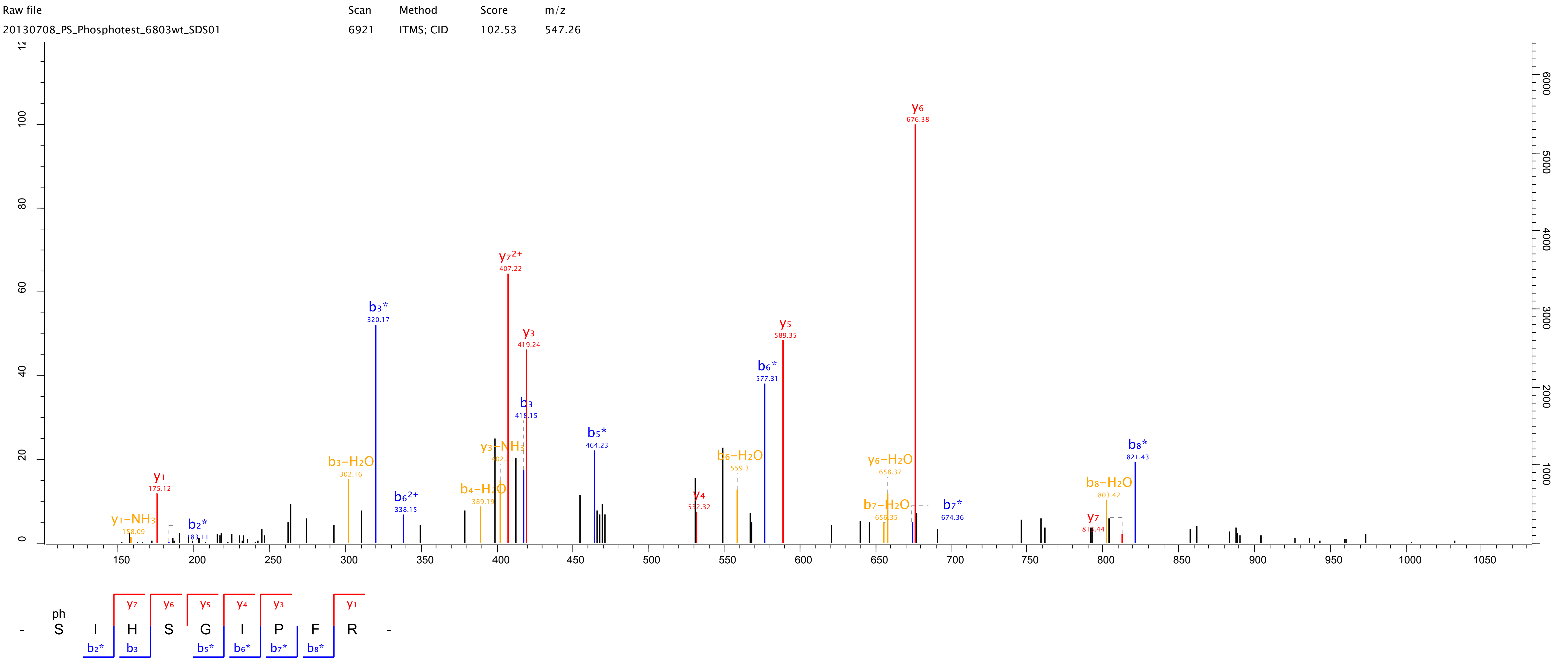

Raw file
20130708_PS_Phosphotest_6803wt_SDS01
Scan
6921
Method
ITMS; CID
Score
102.53
m/z
547.26
y6
676.38
y7 2+
407.22
y3
419.24
b3*
320.17
y5
589.35
b6*
577.31
b3
418.15
b5*
464.23
y3-NH3
402.21
b3-H2O
302.16
b6-H2O
559.3
y6-H2O
658.37
b8*
821.43
y1
175.12
b6 2+
338.15
b4-H2O
389.19
y4
532.32
b8-H2O
803.42
b7-H2O
656.35
b7*
674.36
y1-NH3
158.09
b2*
183.11
y7
813.44
- ph S I H S G I P F R -
y7 y6 y5 y4 y3 y1
b2* b3 b5* b6* b7* b8*

Raw file	Scan	Method	Score	m/z
20130708_PS_Phosphotest_6803wt_SDS01	6938	ITMS; CID	193.72	827.42

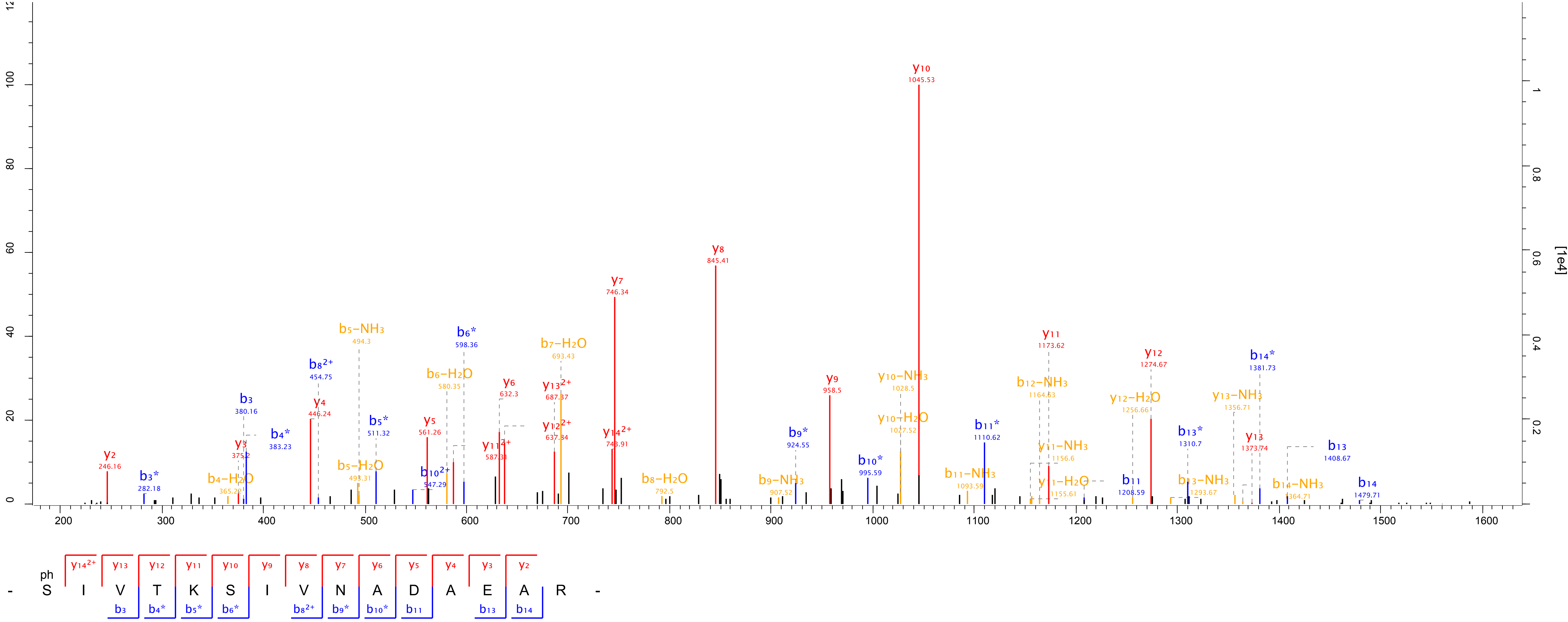

Raw file	Scan	Method	Score	m/z
20130708_PS_Phosphotest_6803wt_SDS01	6944	ITMS; CID	272.72	955.4

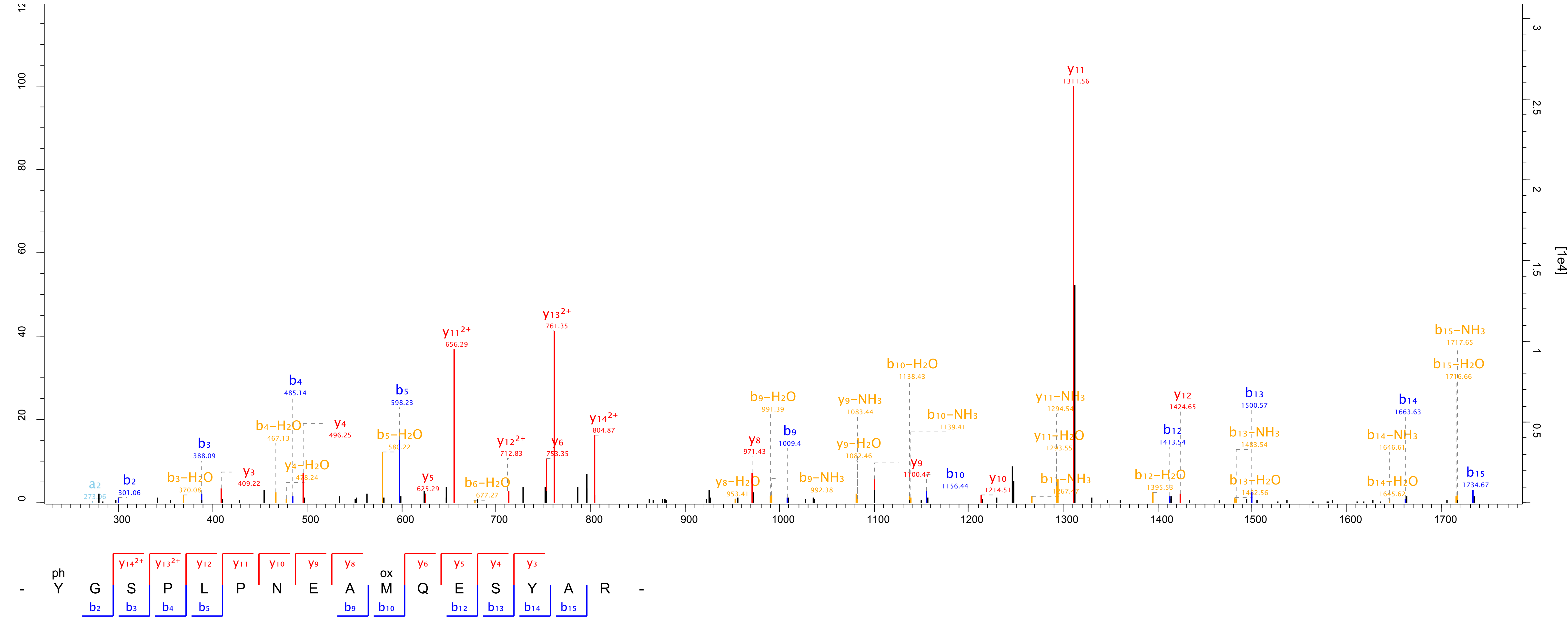

Raw file	Scan	Method	Score	m/z
20130708_PS_Phosphotest_6803wt_SDS01	6951	ITMS; CID	86.54	654.35

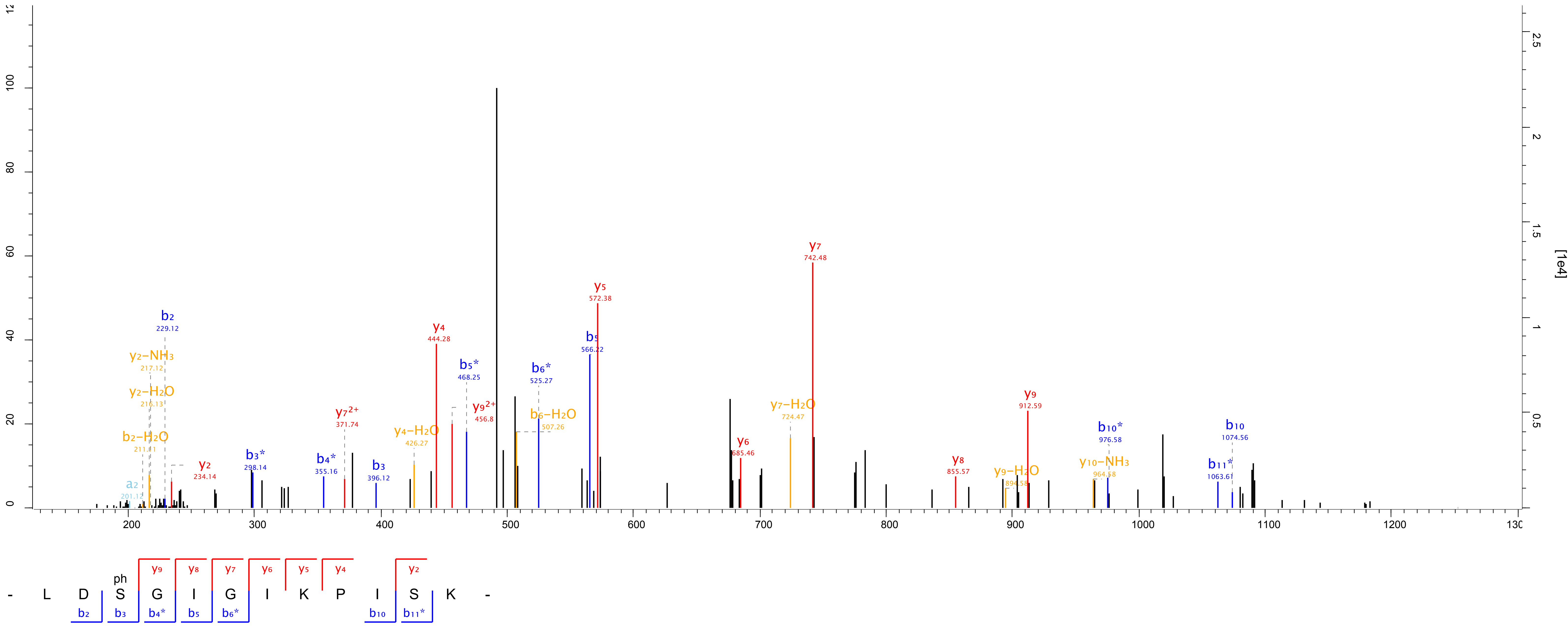

Raw file	Scan	Method	Score	m/z
20130708_PS_Phosphotest_6803wt_SDS01	6954	ITMS; CID	80.24	502.7

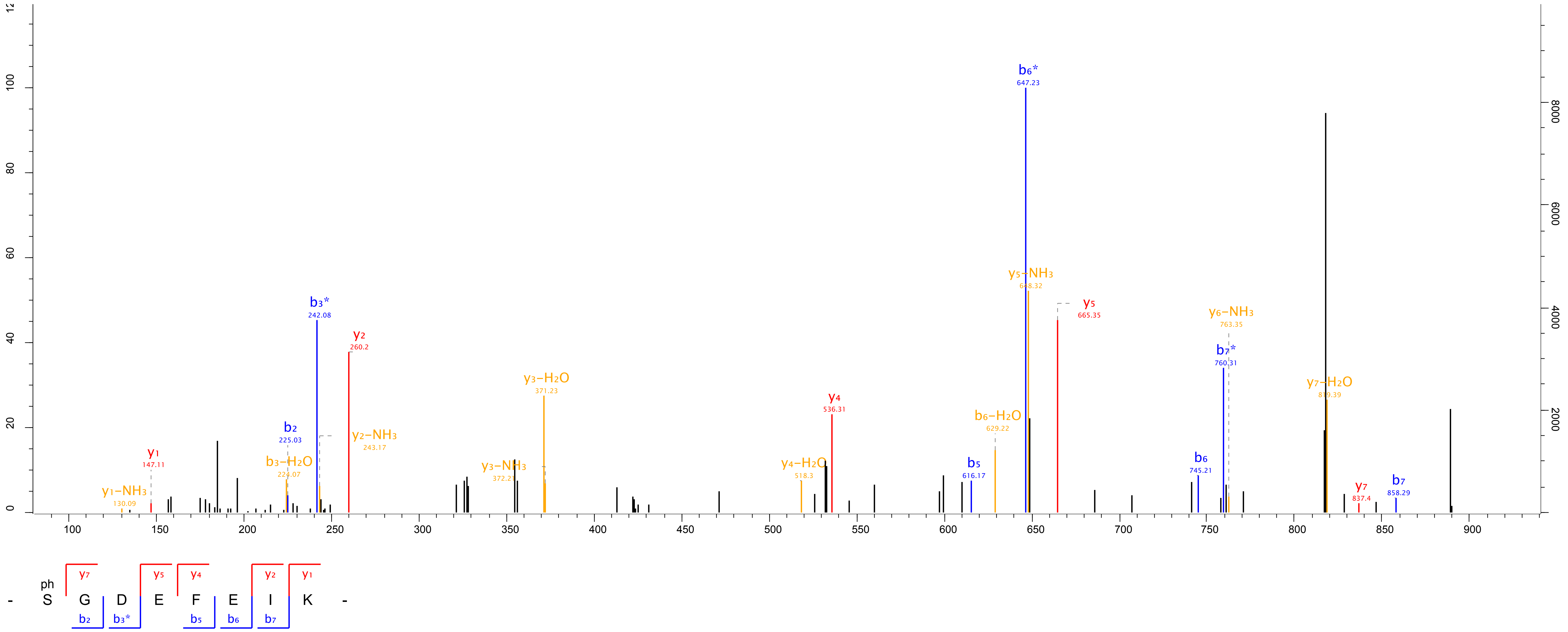

Raw file	Scan	Method	Score	m/z
20130708_PS_Phosphotest_6803wt_SDS01	7044	ITMS; CID	123.95	734.34

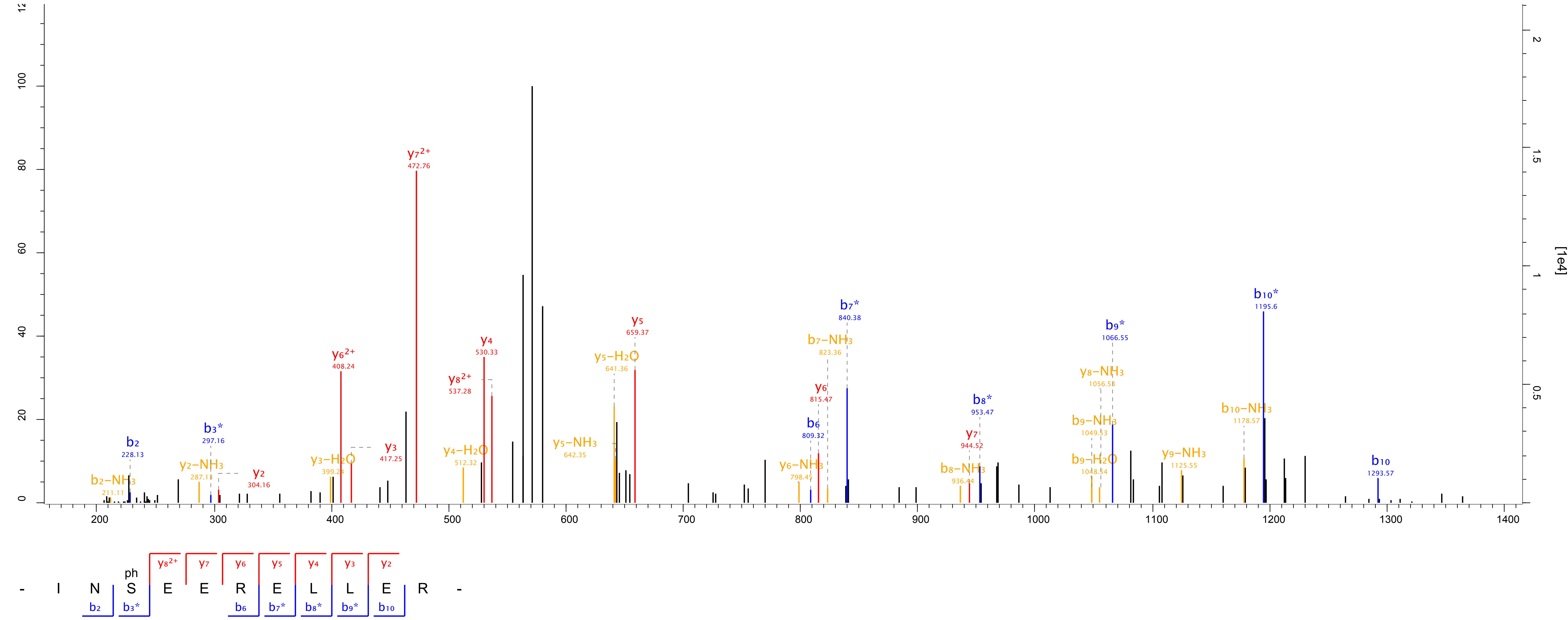

Raw file	Scan	Method	Score	m/z
20130708_PS_Phosphotest_6803wt_SDS01	7050	ITMS; CID	110.98	712.85

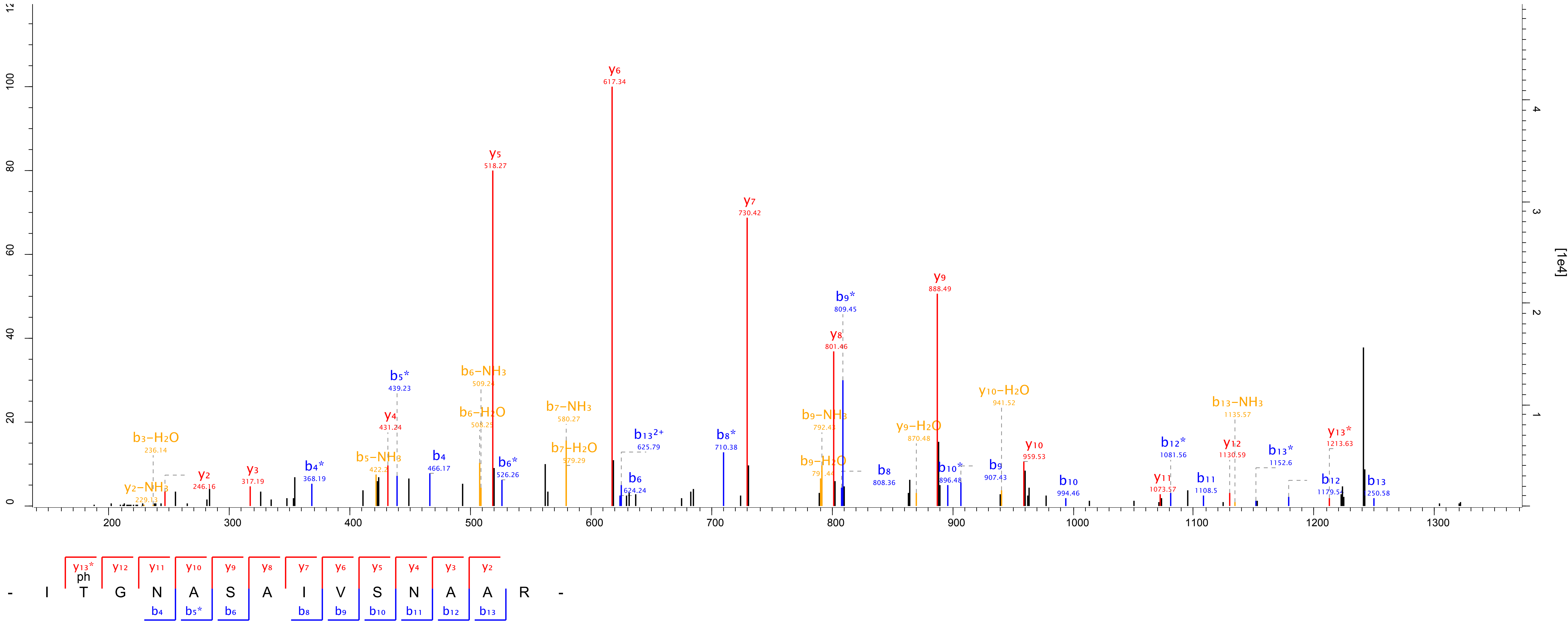

Raw file	Scan	Method	Score	m/z
20130708_PS_Phosphotest_6803wt_SDS01	7058	ITMS; CID	171.62	585.28

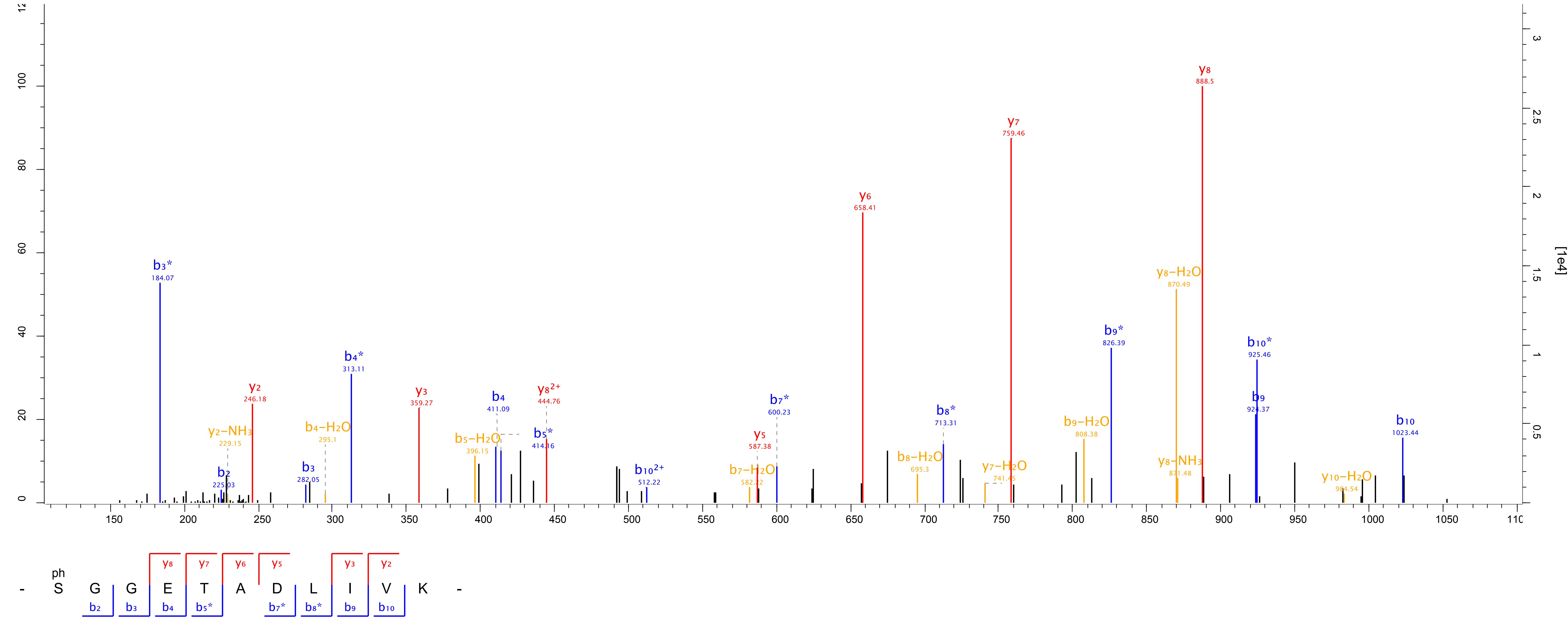

Raw file	Scan	Method	Score	m/z
20130708_PS_Phosphotest_6803wt_SDS01	7079	ITMS; CID	107.09	662.29

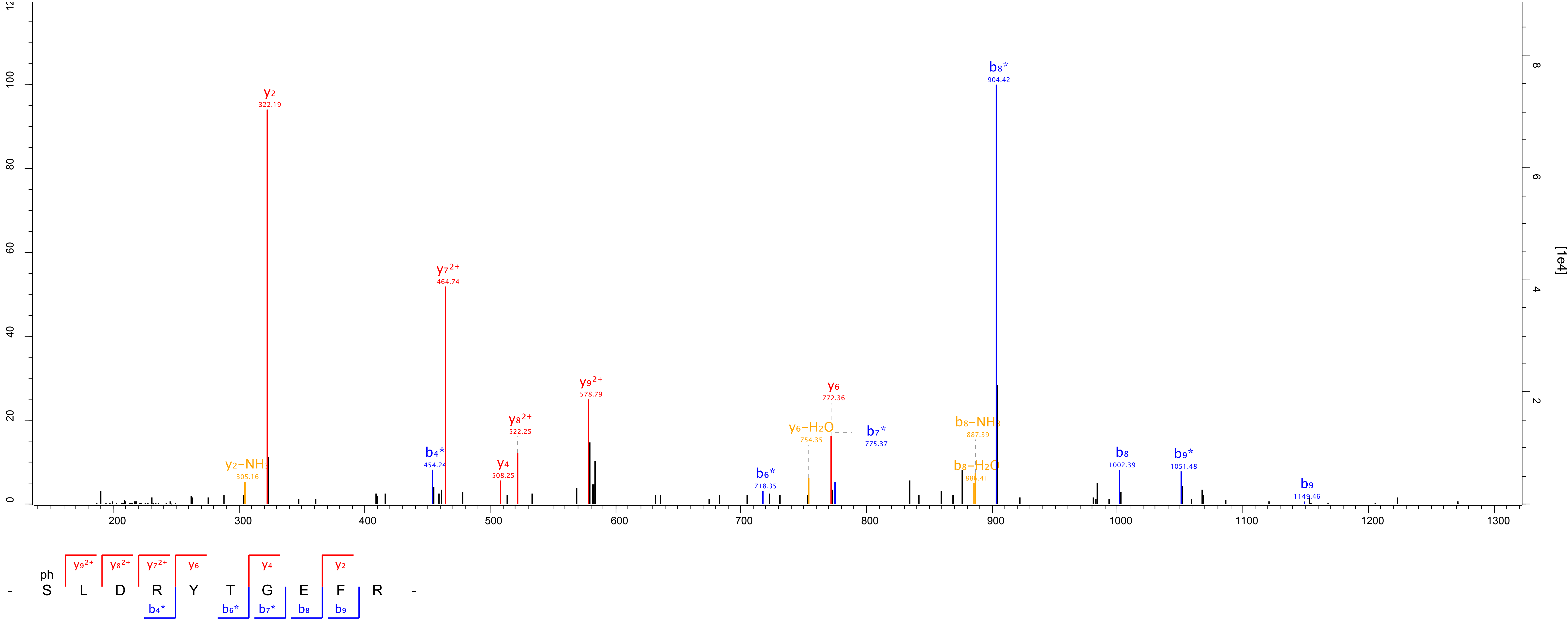

Raw file	Scan	Method	Score	m/z
20130708_PS_Phosphotest_6803wt_SDS01	7086	ITMS; CID	103.91	465.23

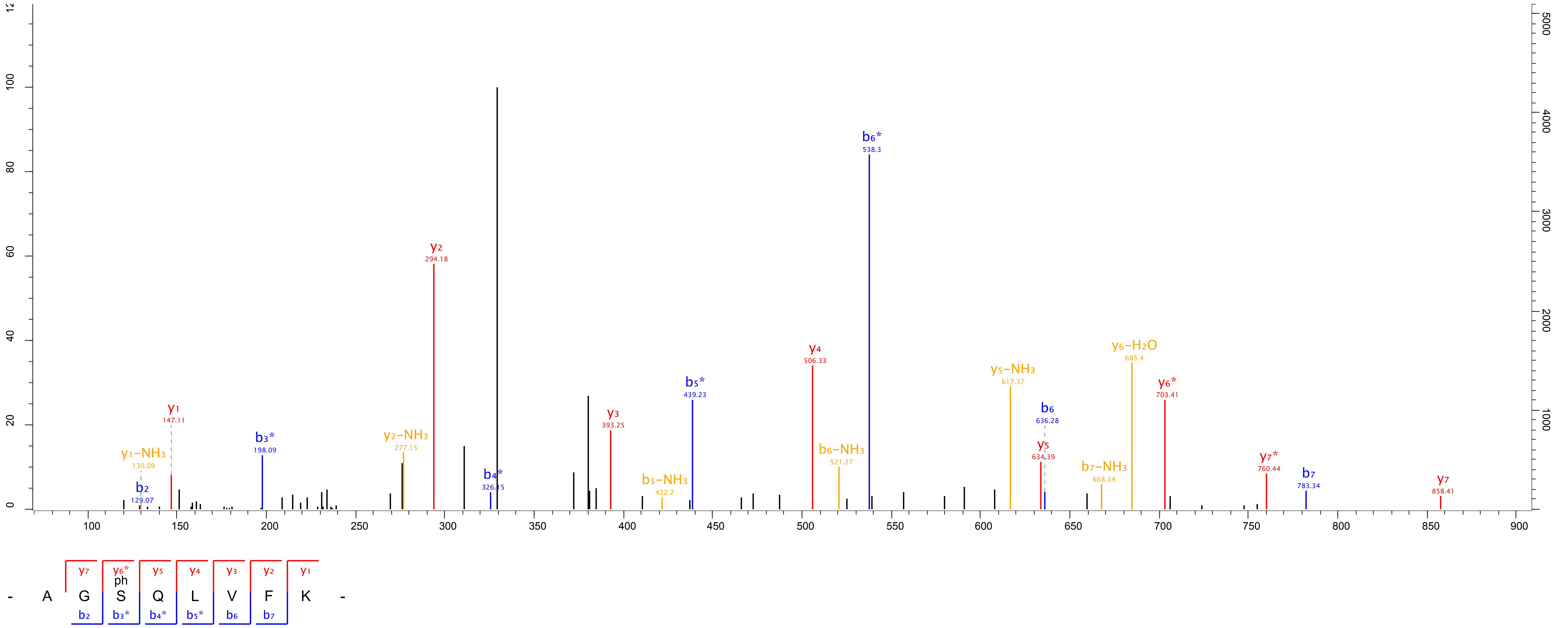

Raw file	Scan	Method	Score	m/z
20130708_PS_Phosphotest_6803wt_SDS01	7089	ITMS; CID	104.9	658.32

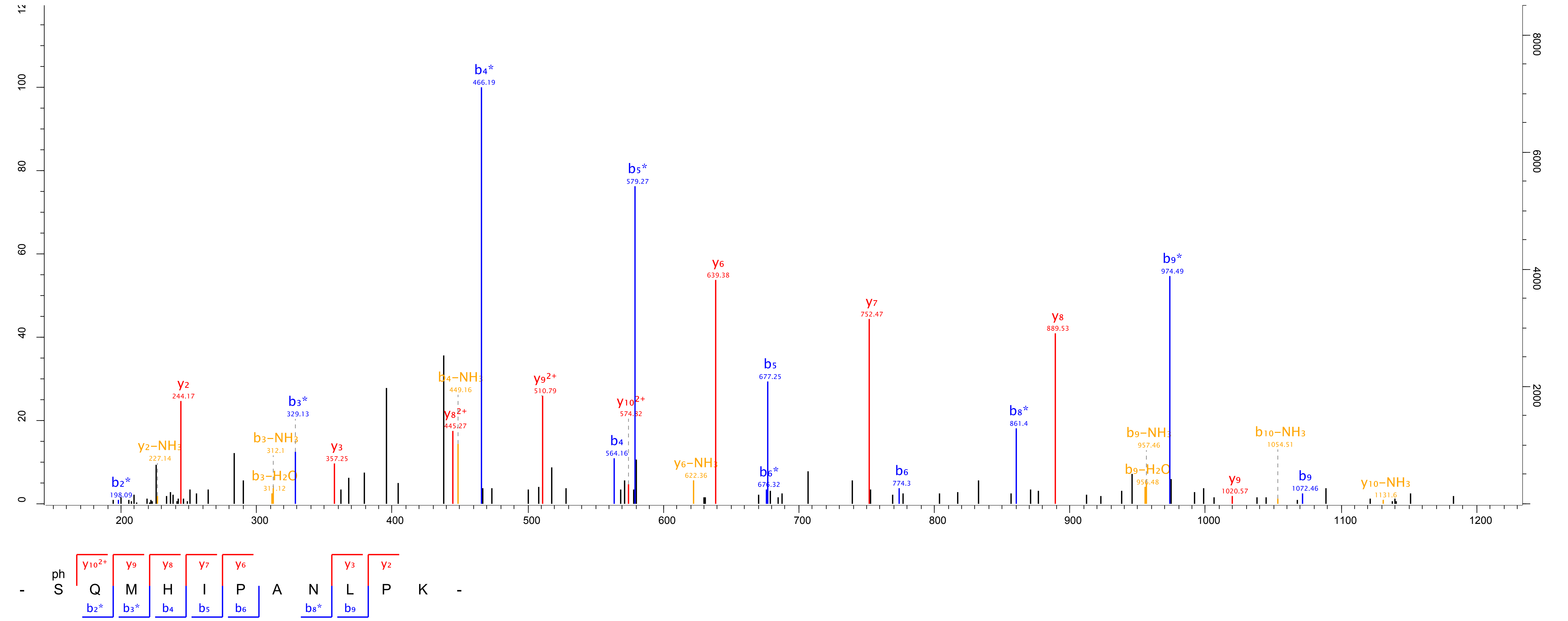

Raw file	Scan	Method	Score	m/z
20130708_PS_Phosphotest_6803wt_SDS01	7176	ITMS; CID	99.27	826.42

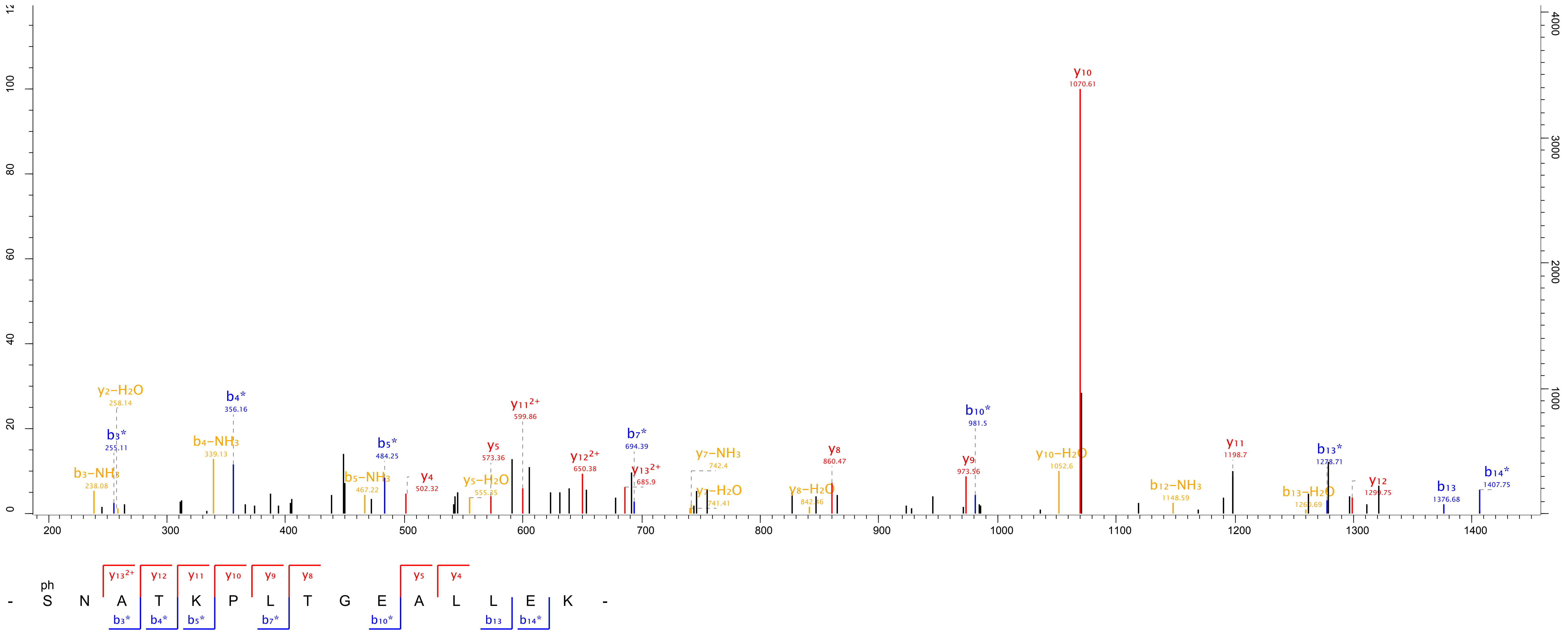

Raw file	Scan	Method	Score	m/z
20130708_PS_Phosphotest_6803wt_YPER01	7204	ITMS; CID	114.75	806.86

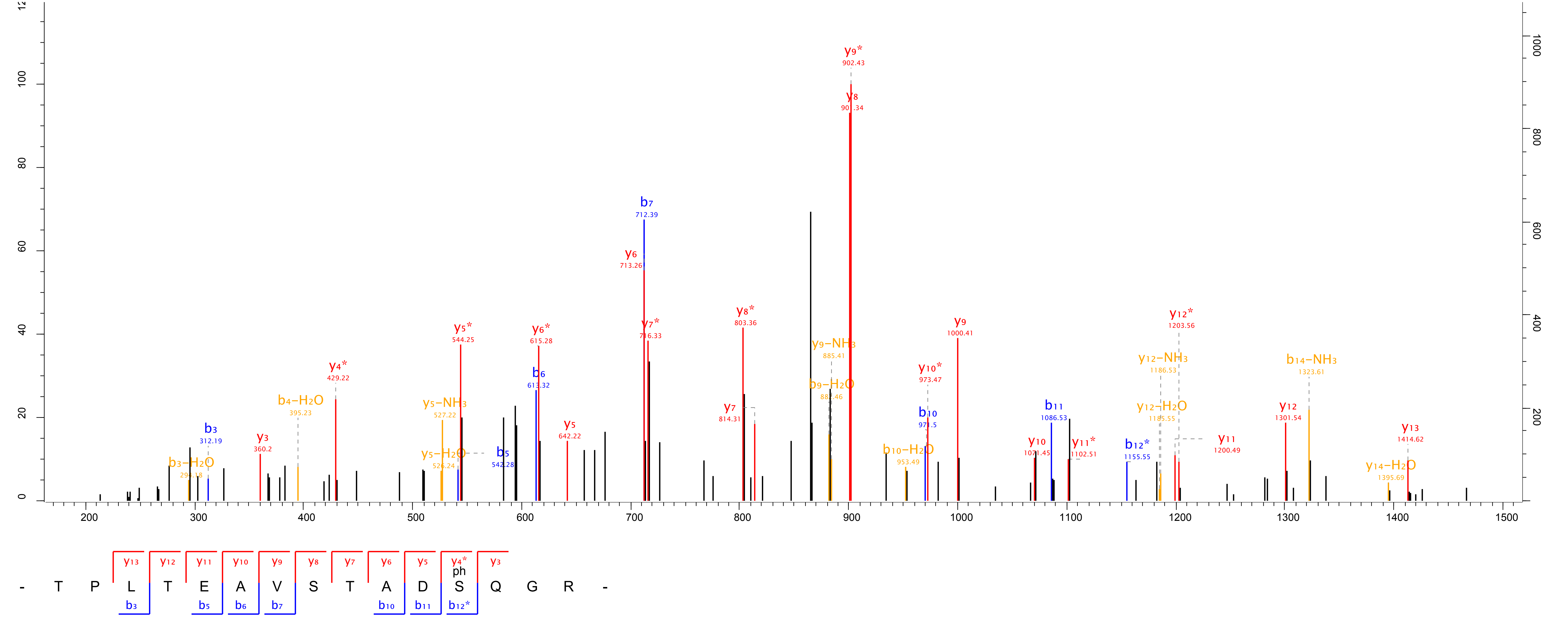

Raw file	Scan	Method	Score	m/z
20130708_PS_Phosphotest_6803wt_YPER01	7208	ITMS; CID	109.6	552.26

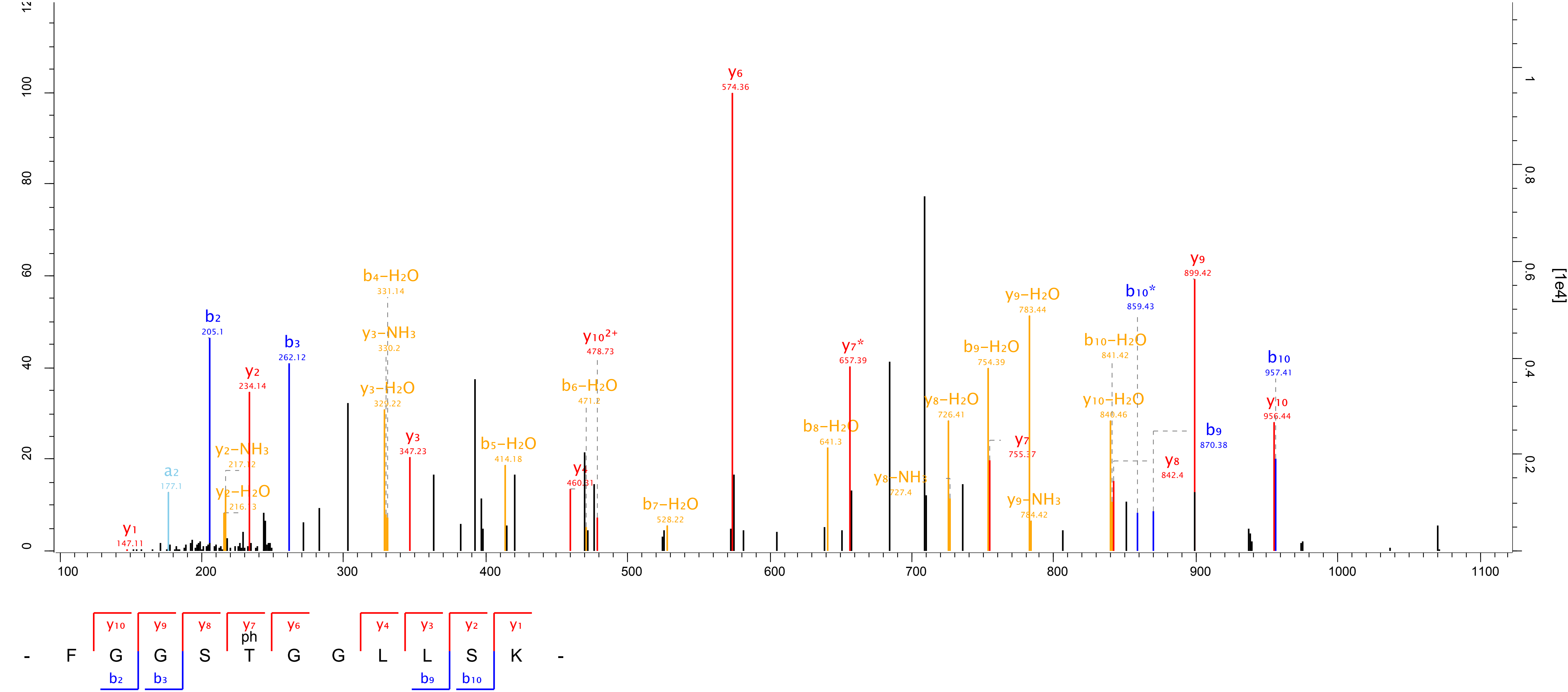

Raw file	Scan	Method	Score	m/z
20130708_PS_Phosphotest_6803wt_SDS01	7230	ITMS; CID	146.27	687.84

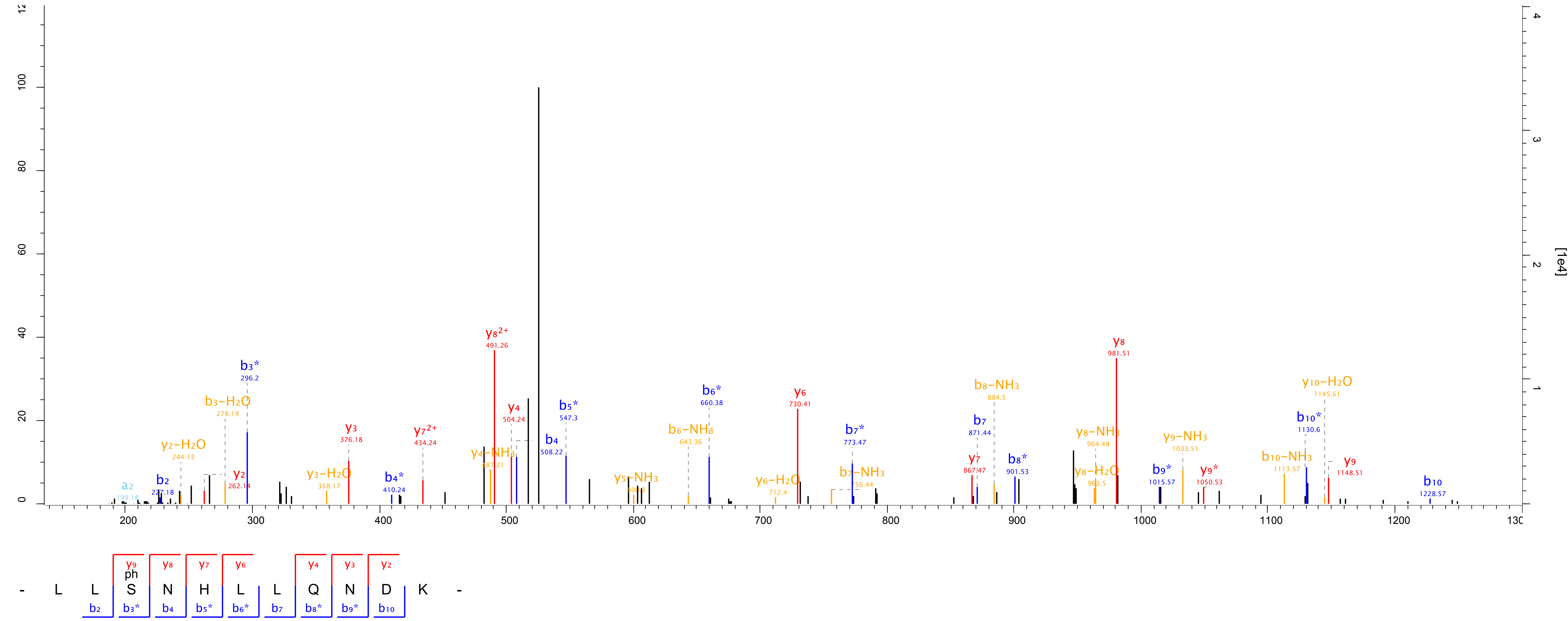

Raw file	Scan	Method	Score	m/z
20130708_PS_Phosphotest_6803wt_SDS03	7311	ITMS; CID	122.1	552.26

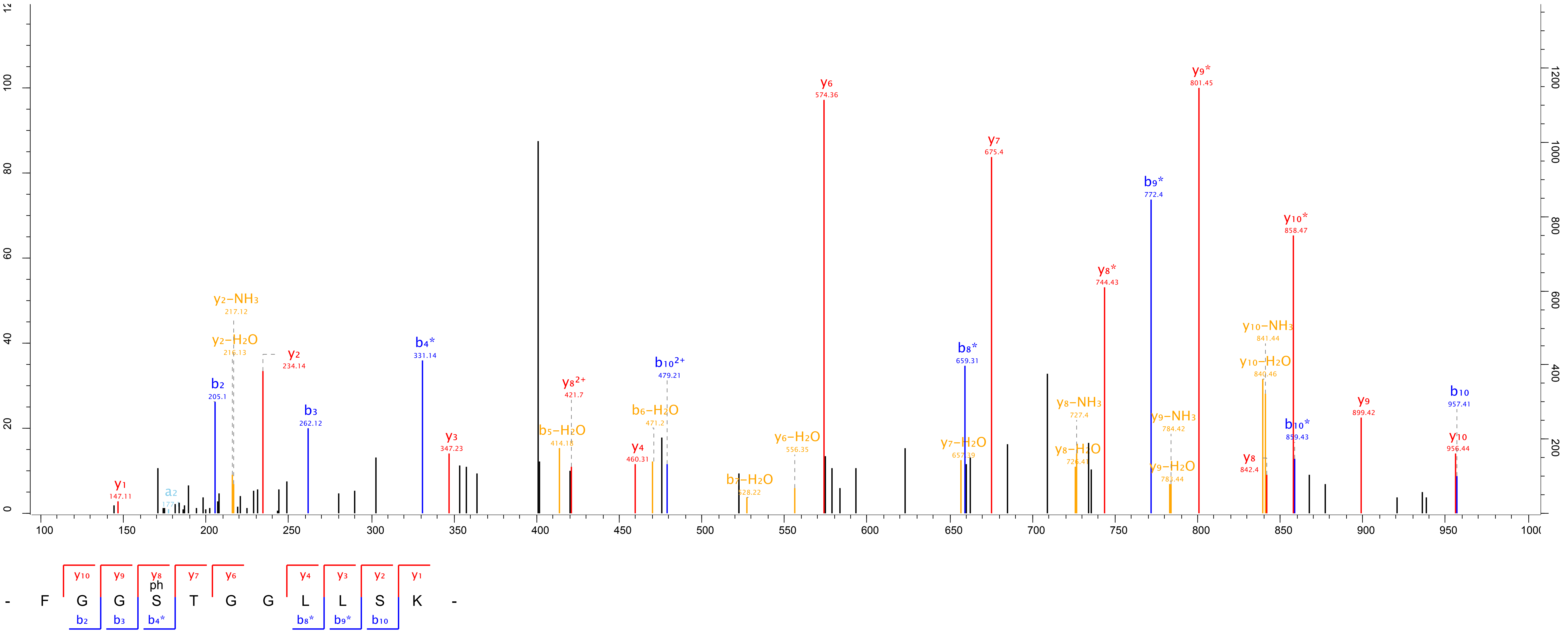

Raw file	Scan	Method	Score	m/z
20130708_PS_Phosphotest_6803wt_SDS01	7338	ITMS; CID	88.5	506.74

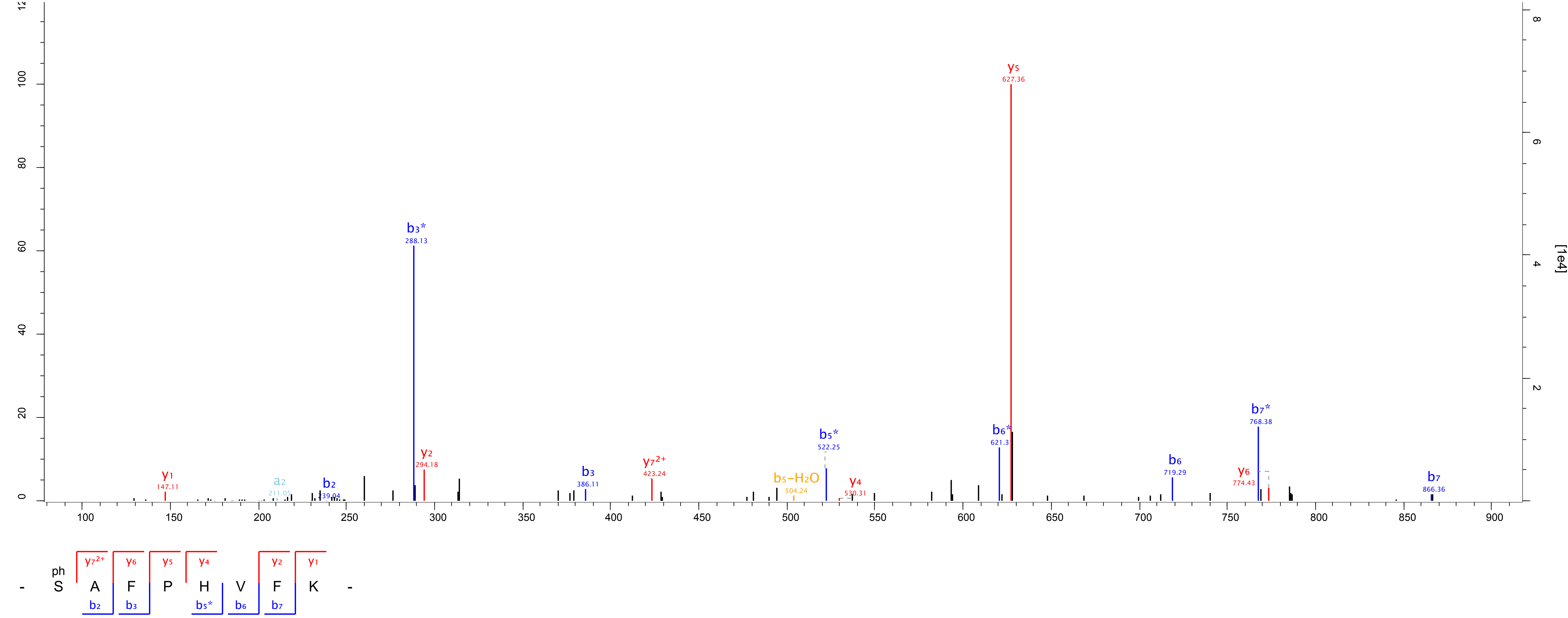

Raw file	Scan	Method	Score	m/z
20130708_PS_Phosphotest_6803wt_YPER01	7354	ITMS; CID	162.68	555.24

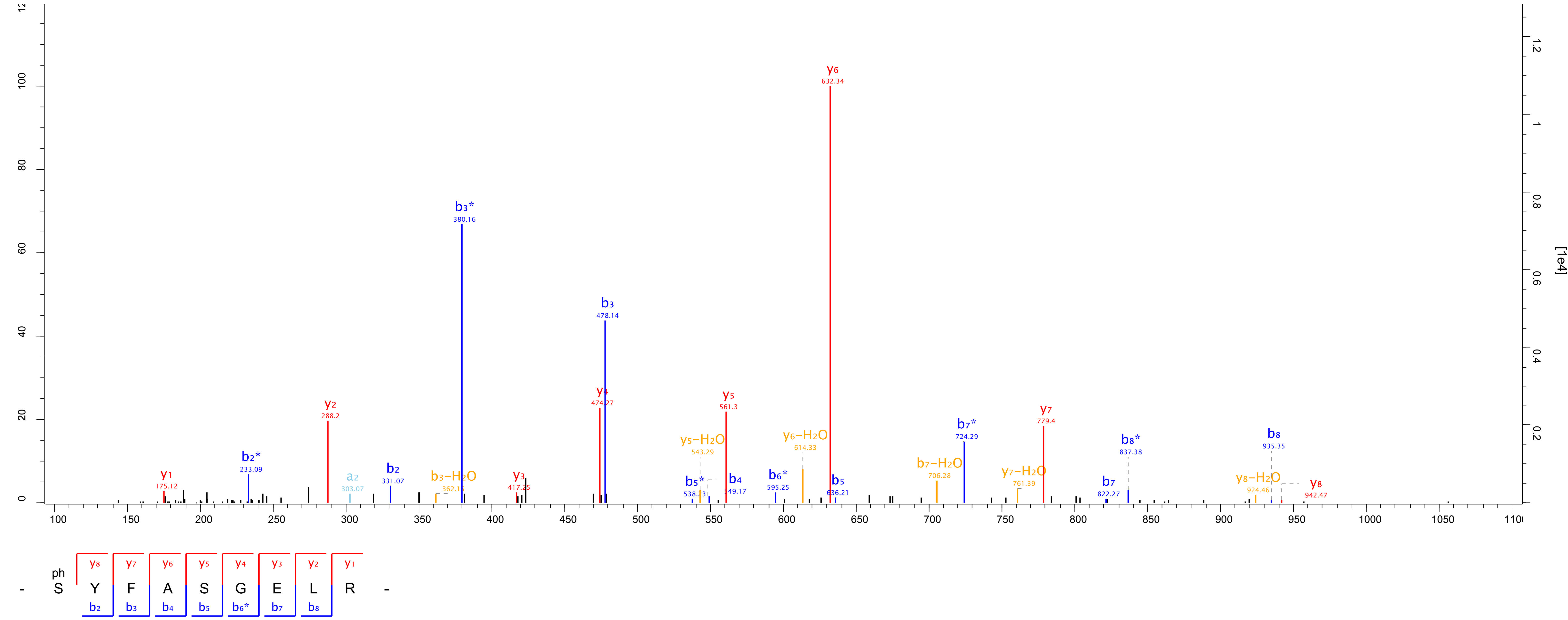

Raw file	Scan	Method	Score	m/z
20130708_PS_Phosphotest_6803wt_SDS01	7407	ITMS; CID	113.76	564.78

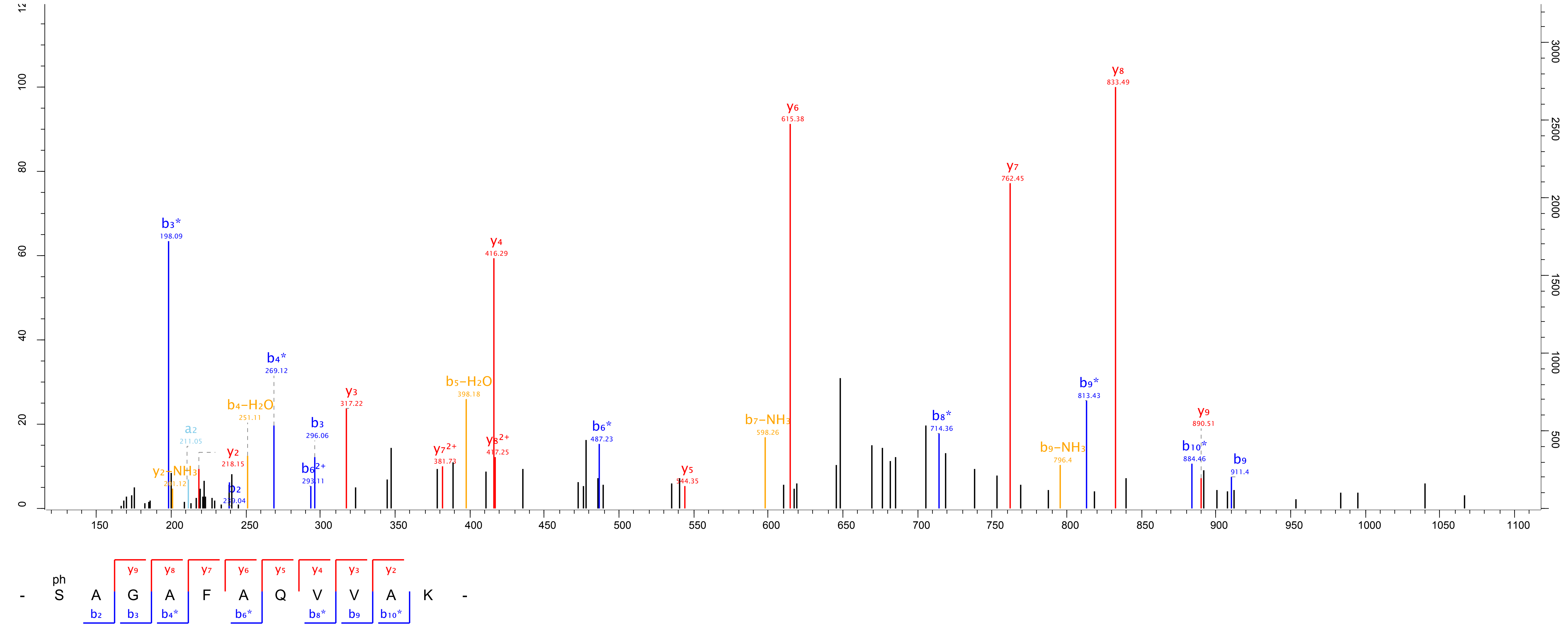

Raw file	Scan	Method	Score	m/z
20130708_PS_Phosphotest_6803wt_SDS01	7418	ITMS; CID	66.26	925.42

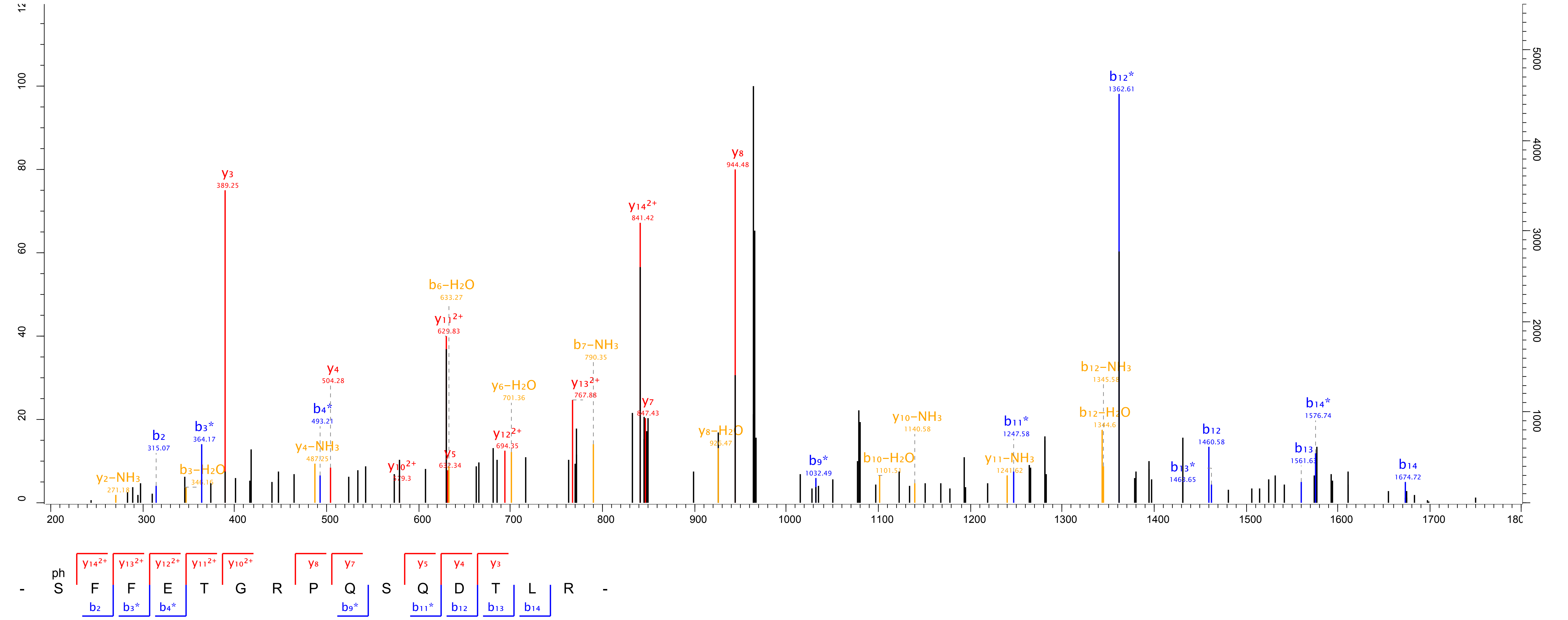

Raw file	Scan	Method	Score	m/z
20130708_PS_Phosphotest_6803wt_SDS01	7430	ITMS; CID	90.73	710.87

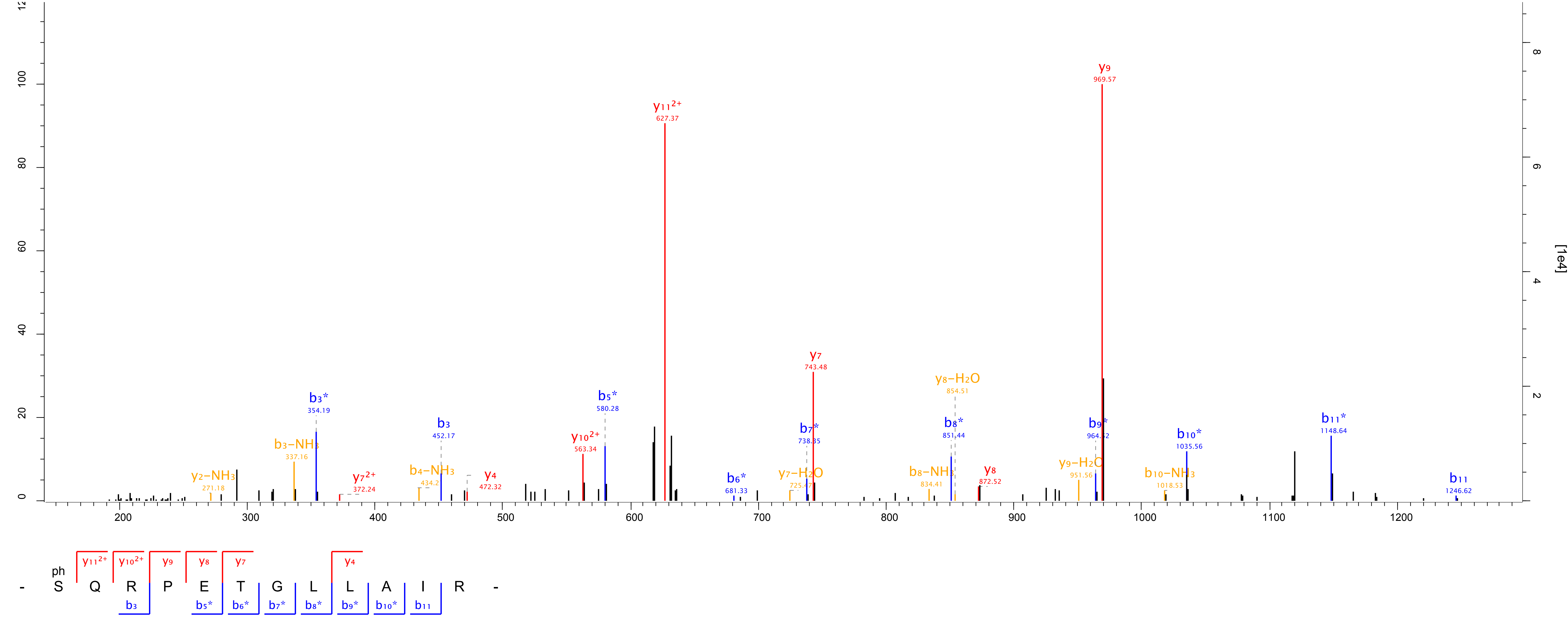

Raw file	Scan	Method	Score	m/z
20130708_PS_Phosphotest_6803wt_SDS01	7442	ITMS; CID	92.27	552.26

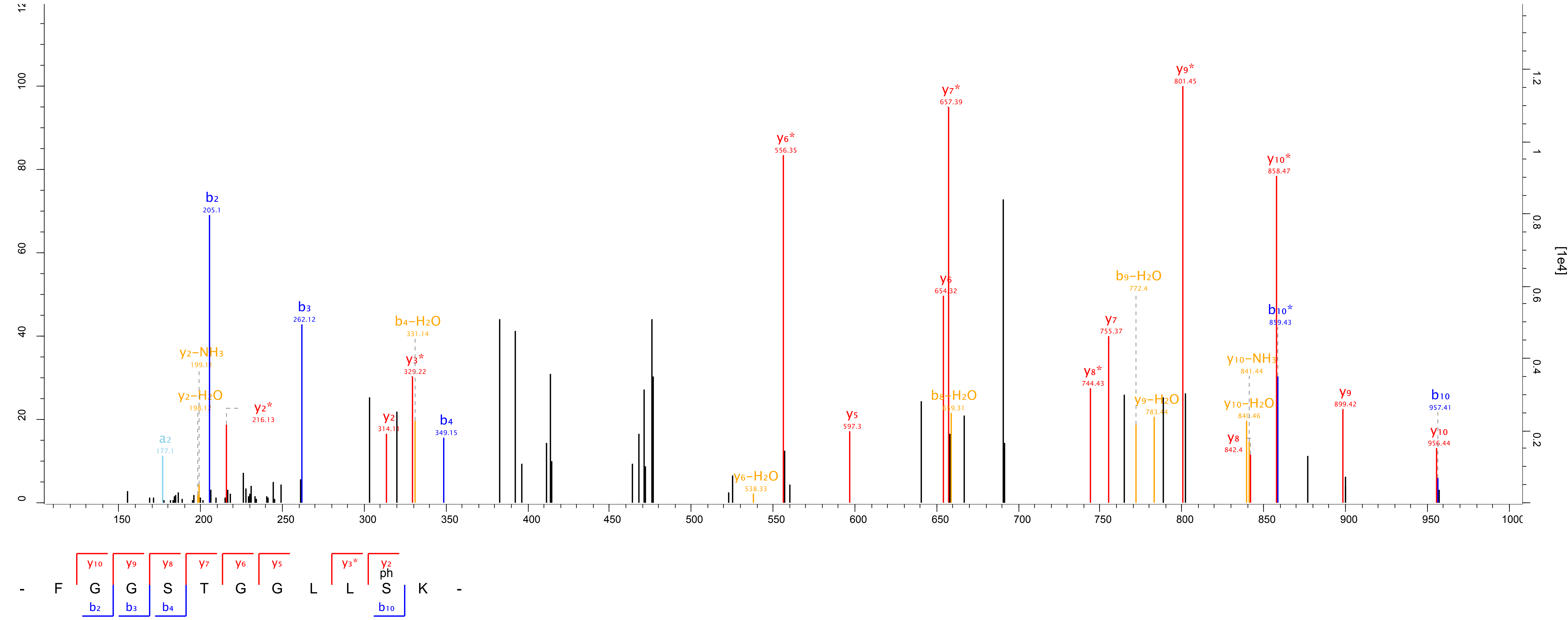

Raw file	Scan	Method	Score	m/z
20130708_PS_Phosphotest_6803wt_SDS01	7457	ITMS; CID	95.42	534.27

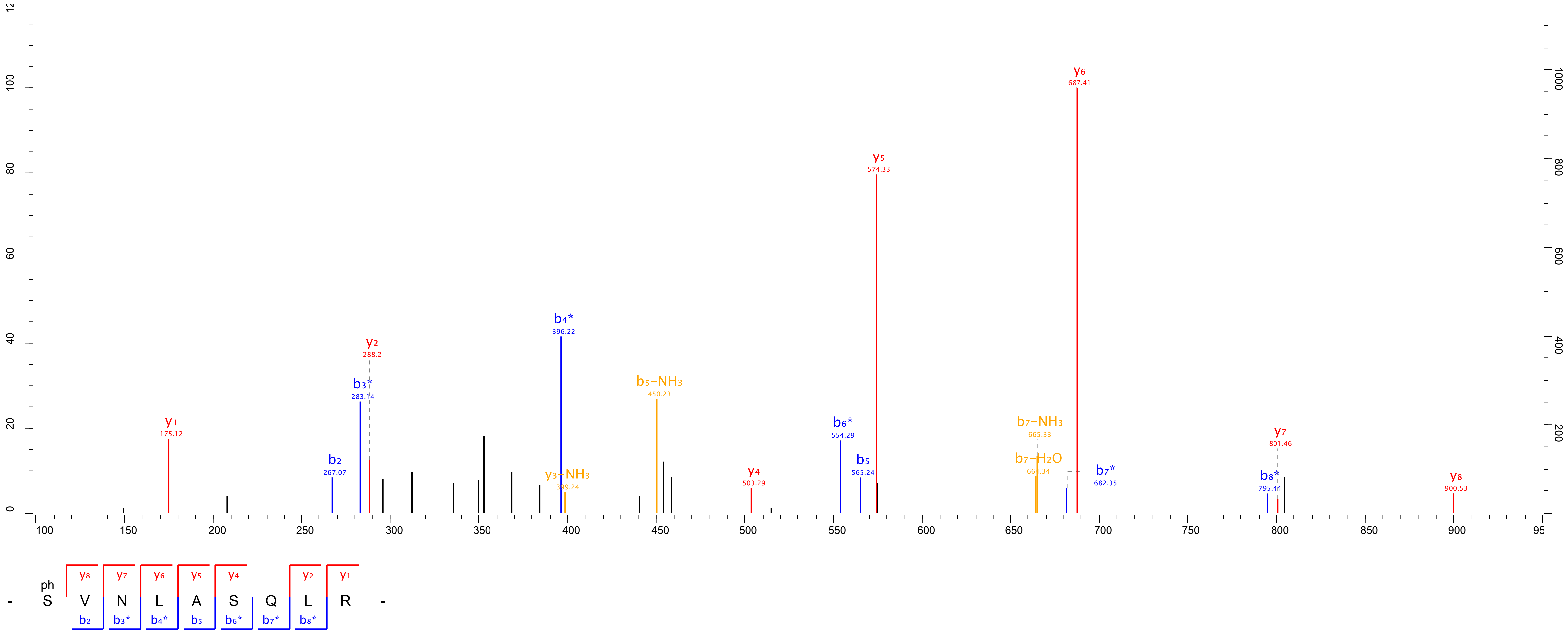

Raw file	Scan	Method	Score	m/z
20130708_PS_Phosphotest_6803wt_SDS01	7463	ITMS; CID	127.87	481.2

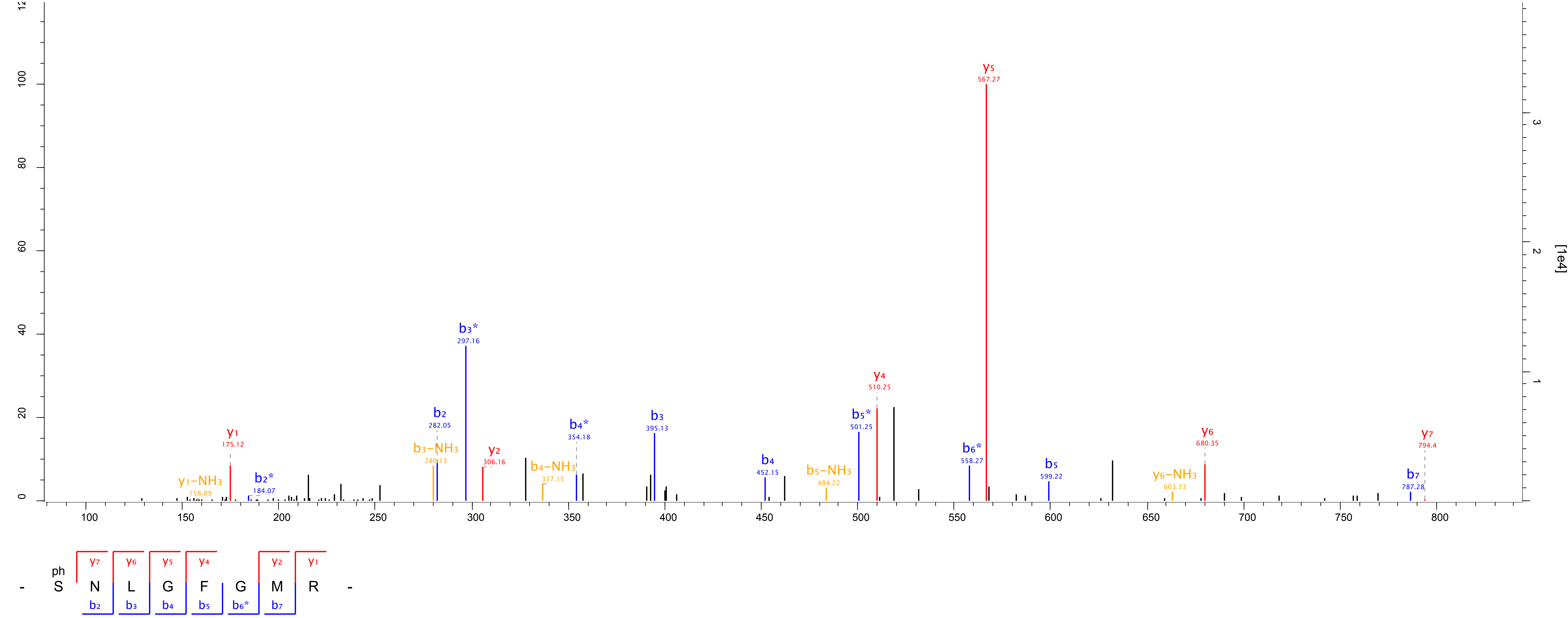

Raw file	Scan	Method	Score	m/z
20130708_PS_Phosphotest_6803wt_SDS01	7592	ITMS; CID	84.51	720.35

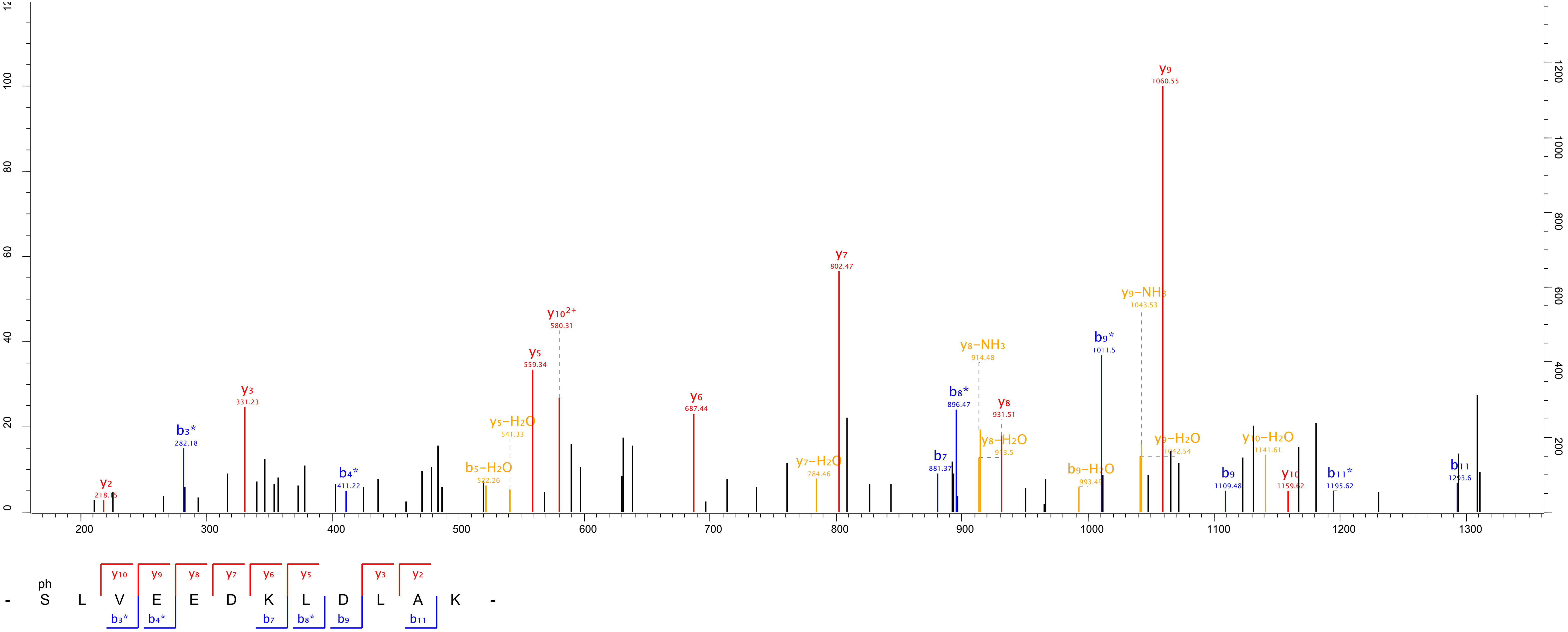

Raw file	Scan	Method	Score	m/z
20130708_PS_Phosphotest_6803wt_SDS01	7602	ITMS; CID	74.54	596.29

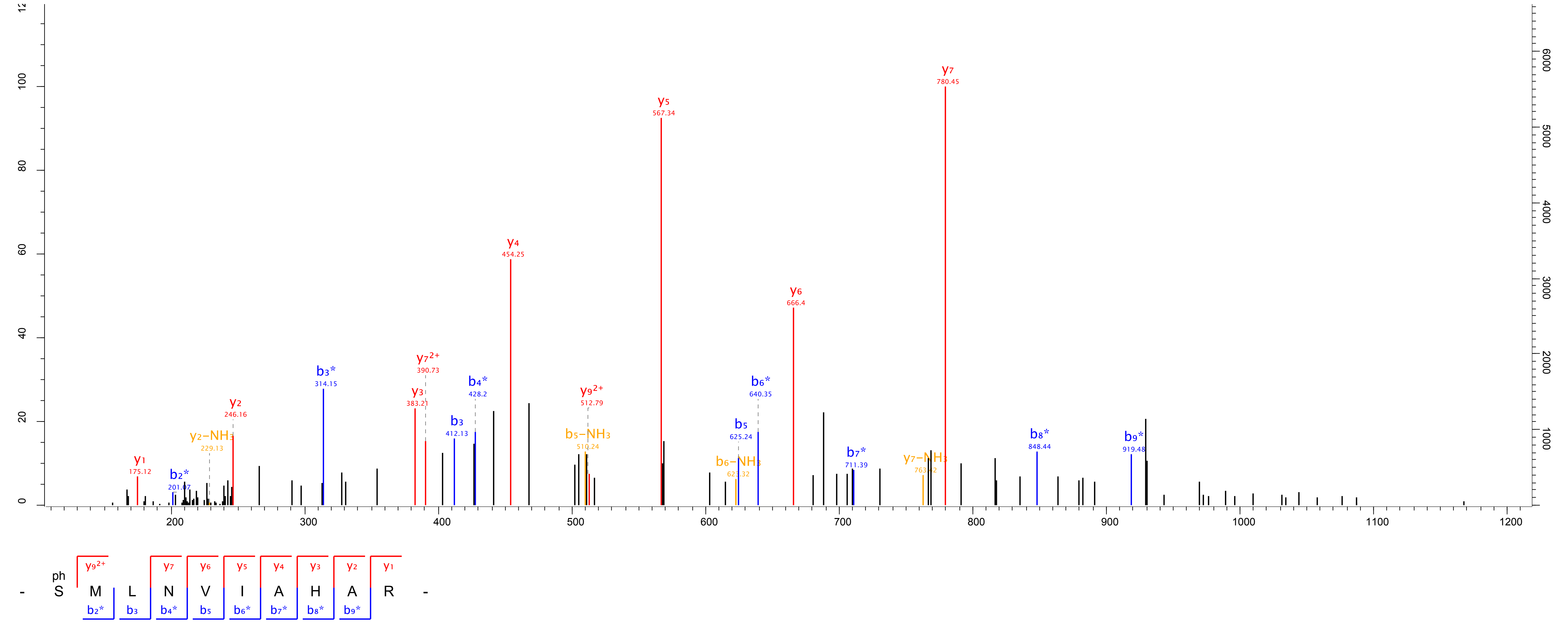

Raw file	Scan	Method	Score	m/z
20130708_PS_Phosphotest_6803wt_SDS01	7615	ITMS; CID	87.57	447.72

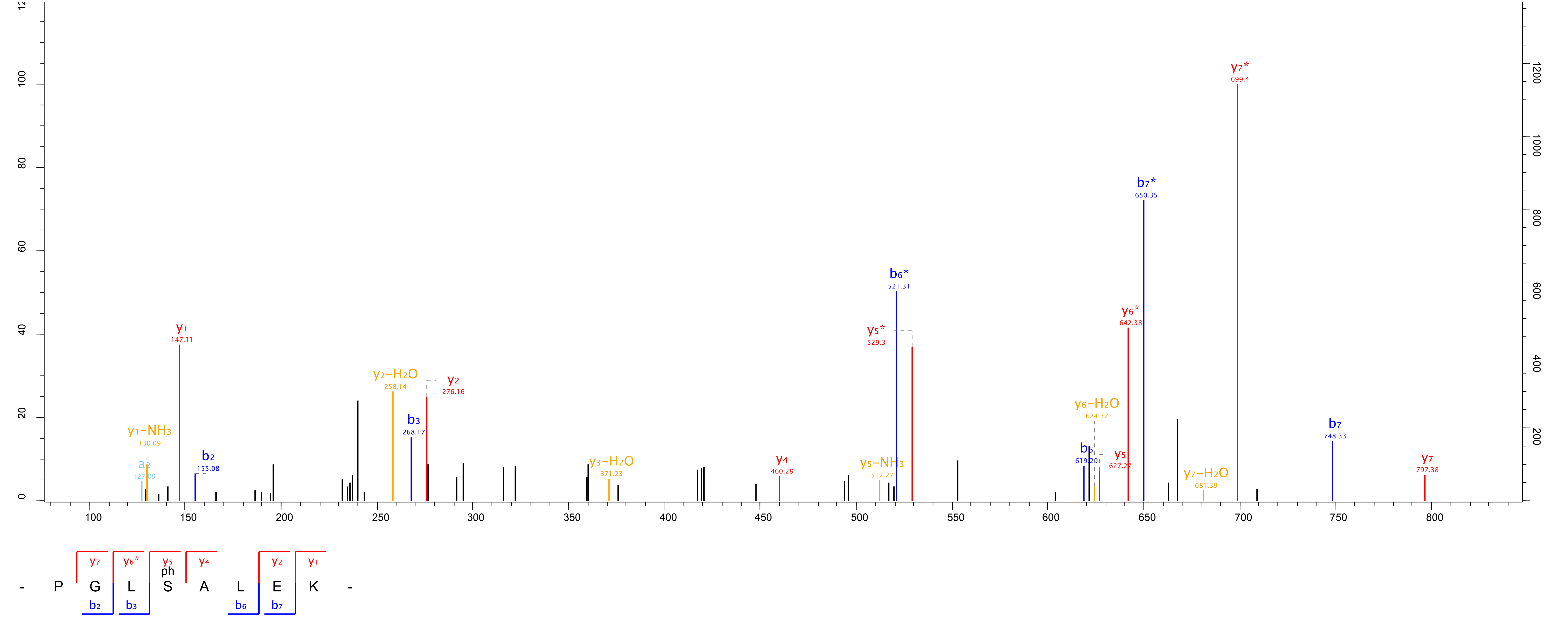

Raw file	Scan	Method	Score	m/z
20130708_PS_Phosphotest_6803wt_SDS01	7626	ITMS; CID	97.78	621.79

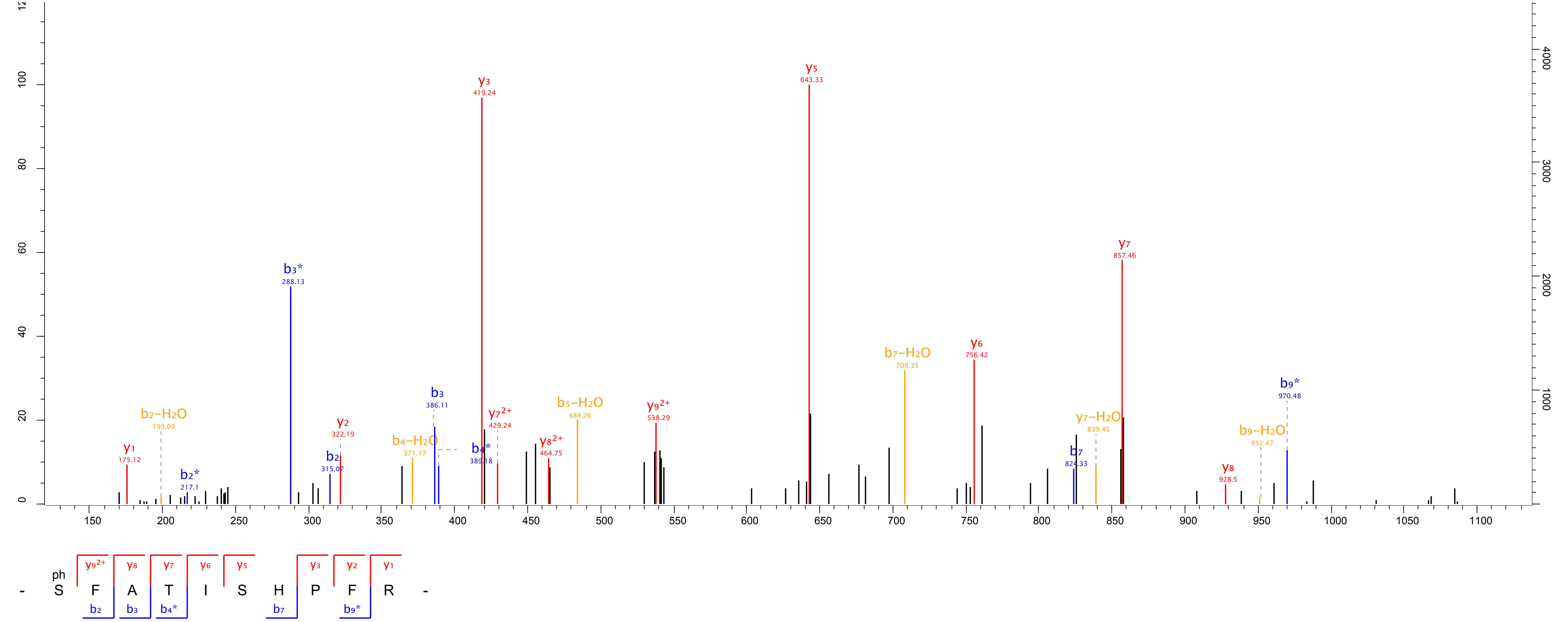

Raw file	Scan	Method	Score	m/z
20130708_PS_Phosphotest_6803wt_YPER01	7667	ITMS; CID	111.63	630.95

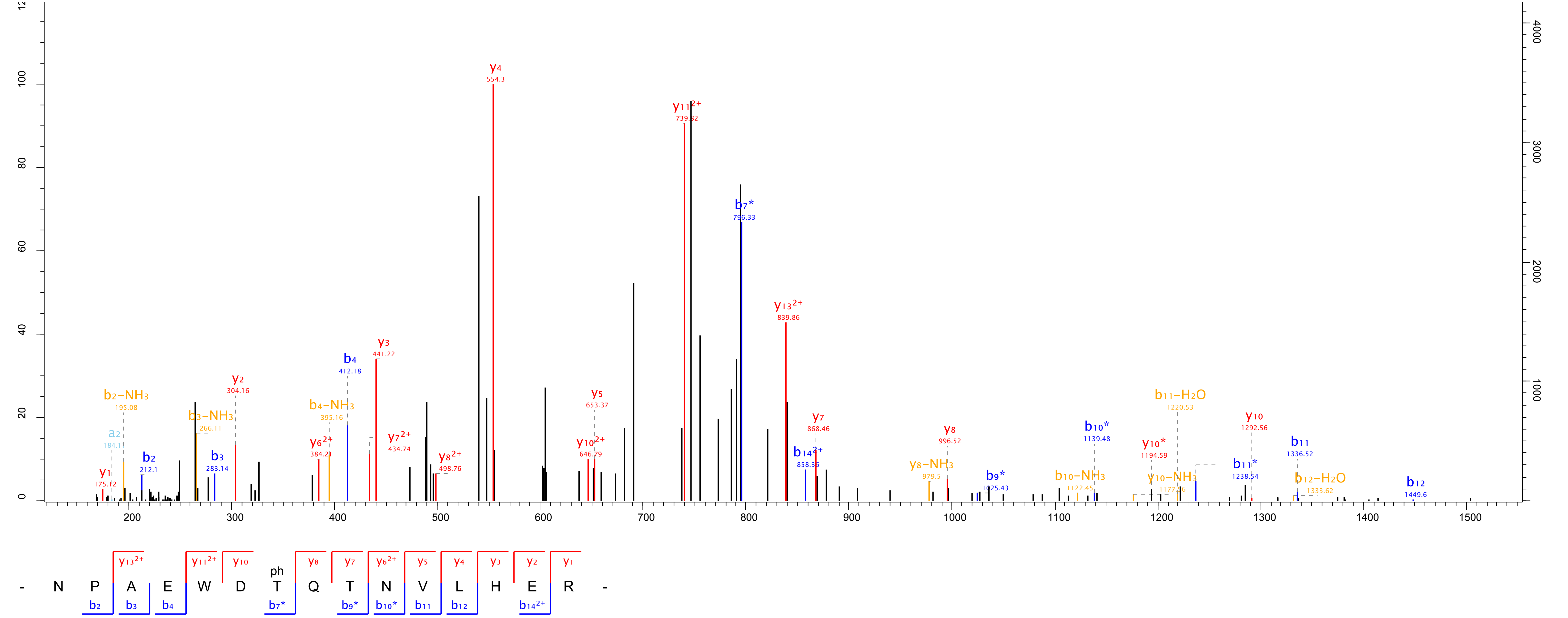

Raw file	Scan	Method	Score	m/z
20130708_PS_Phosphotest_6803wt_SDS01	7681	ITMS; CID	63.08	990.47

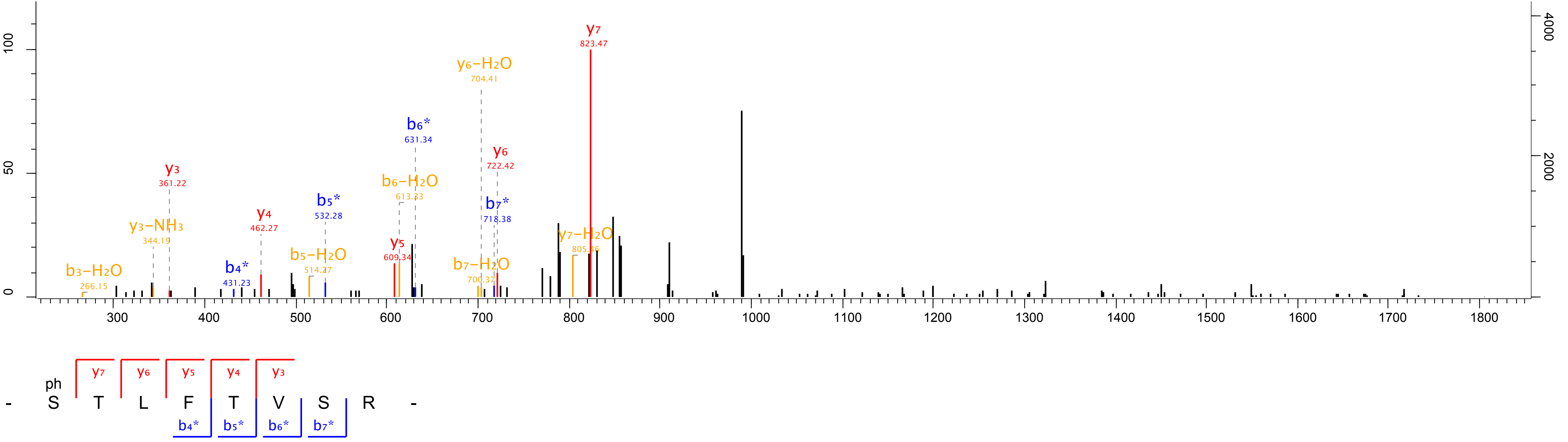

Raw file	Scan	Method	Score	m/z
20130708_PS_Phosphotest_6803wt_SDS01	7723	ITMS; CID	101.71	555.24

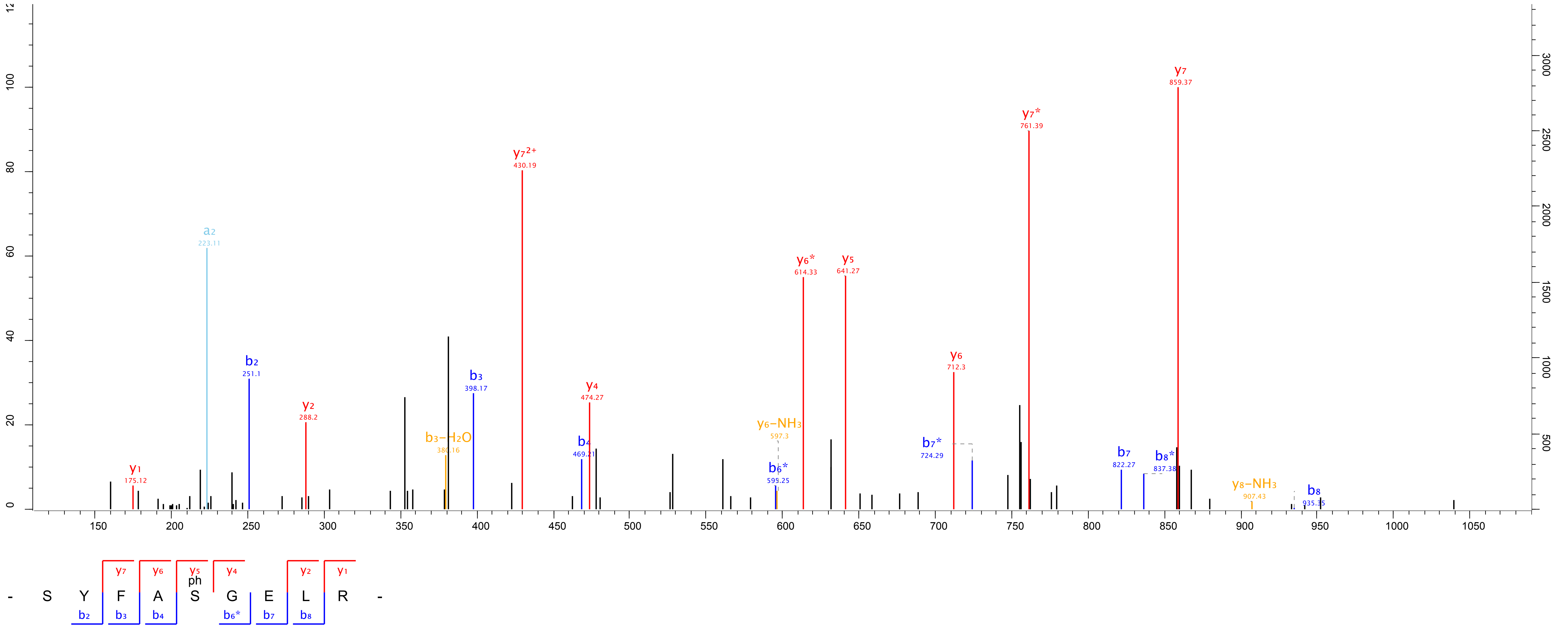

Raw file	Scan	Method	Score	m/z
20130708_PS_Phosphotest_6803wt_SDS01	7777	ITMS; CID	245.04	838.39

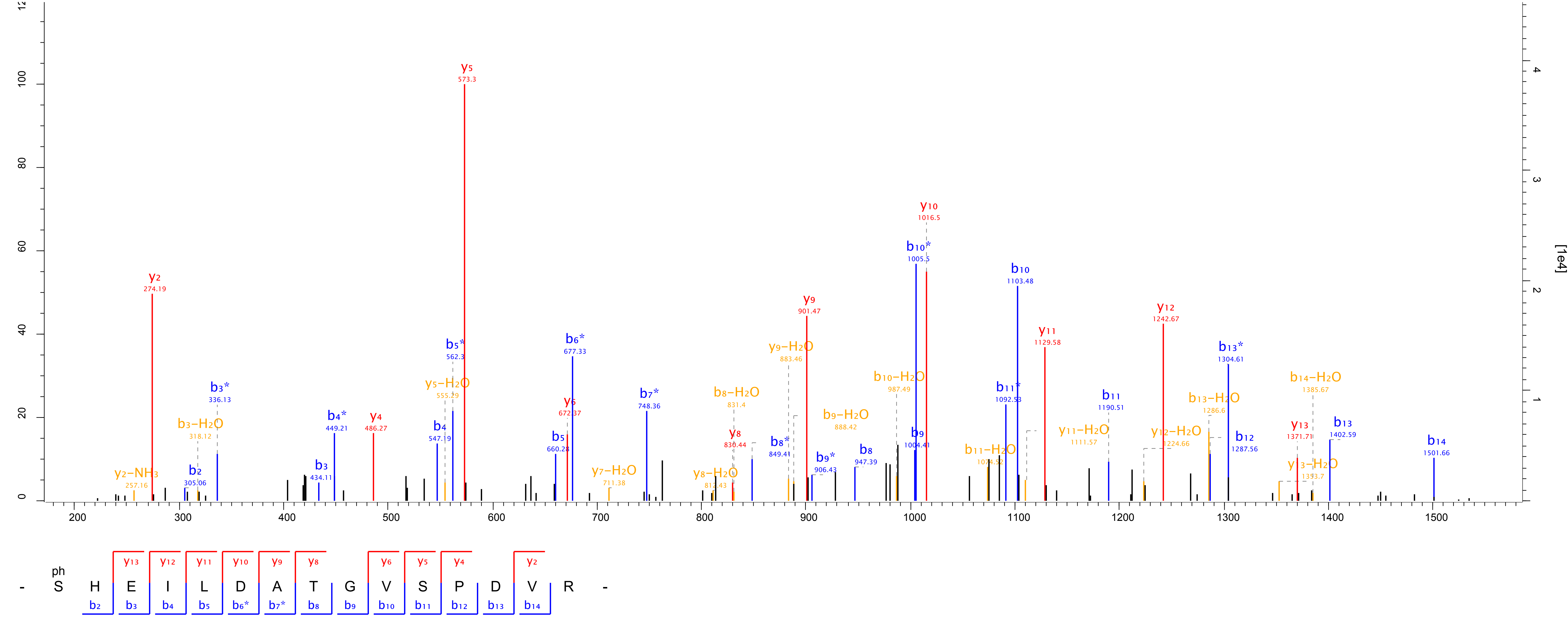

Raw file	Scan	Method	Score	m/z
20130708_PS_Phosphotest_6803wt_SDS01	7857	ITMS; CID	204.78	806.86

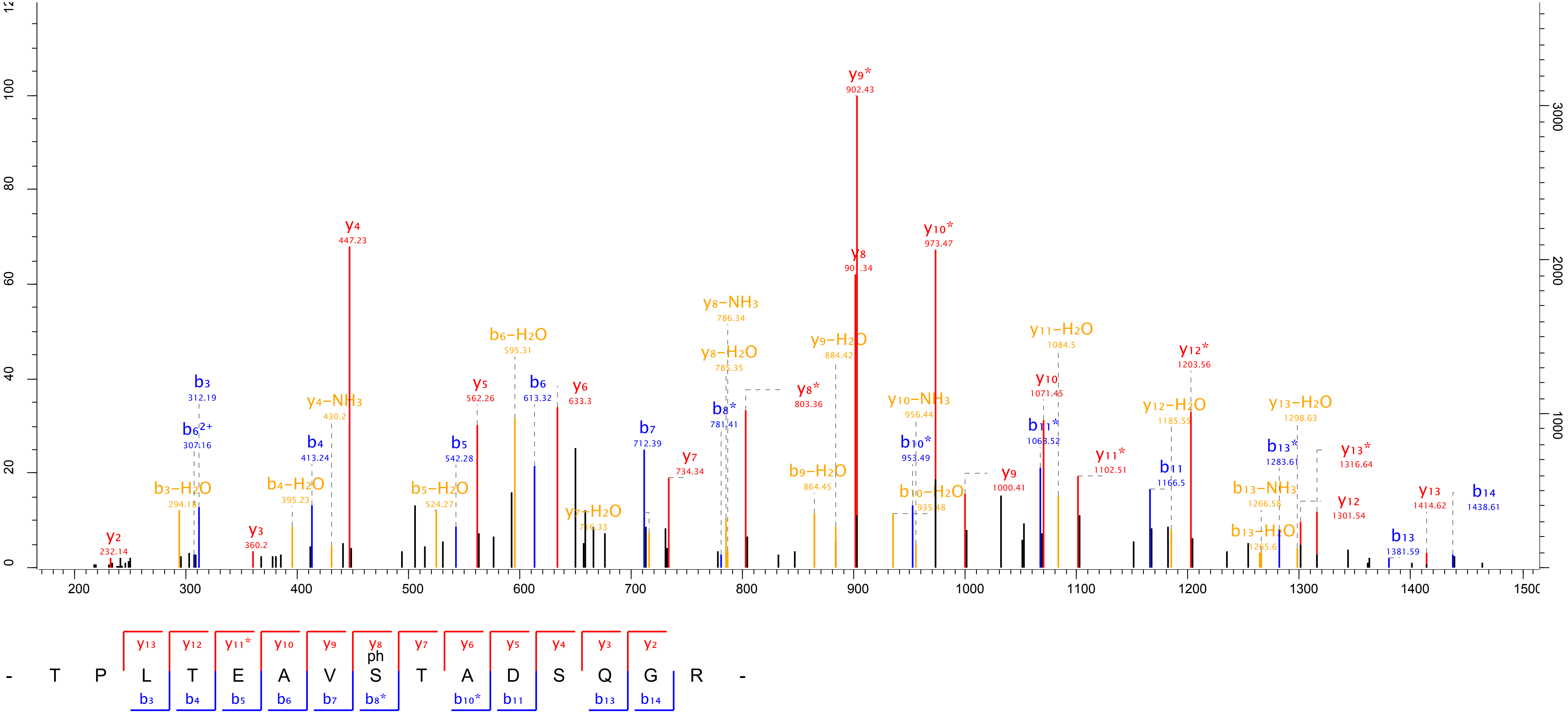

Raw file	Scan	Method	Score	m/z
20130708_PS_Phosphotest_6803wt_YPER01	7916	ITMS; CID	165.24	706.34

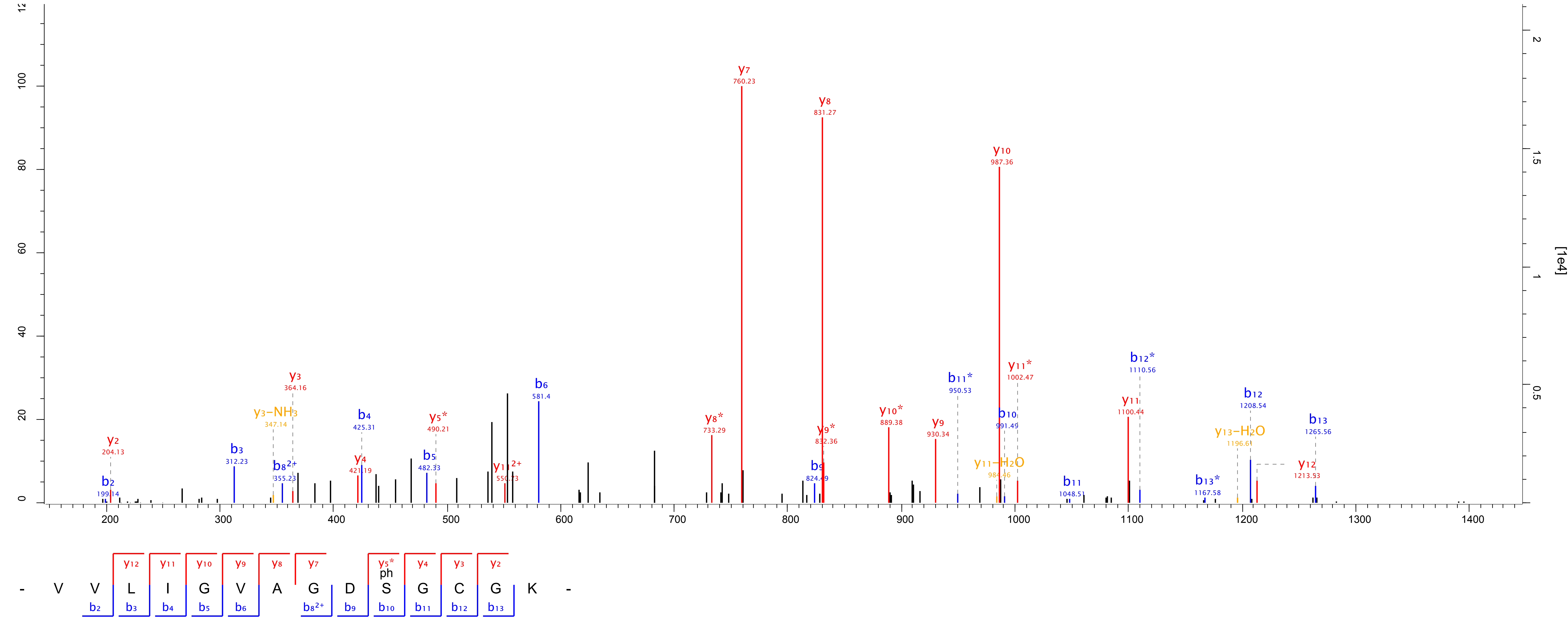

Raw file	Scan	Method	Score	m/z
20130708_PS_Phosphotest_6803wt_SDS01	8034	ITMS; CID	86.46	529.27

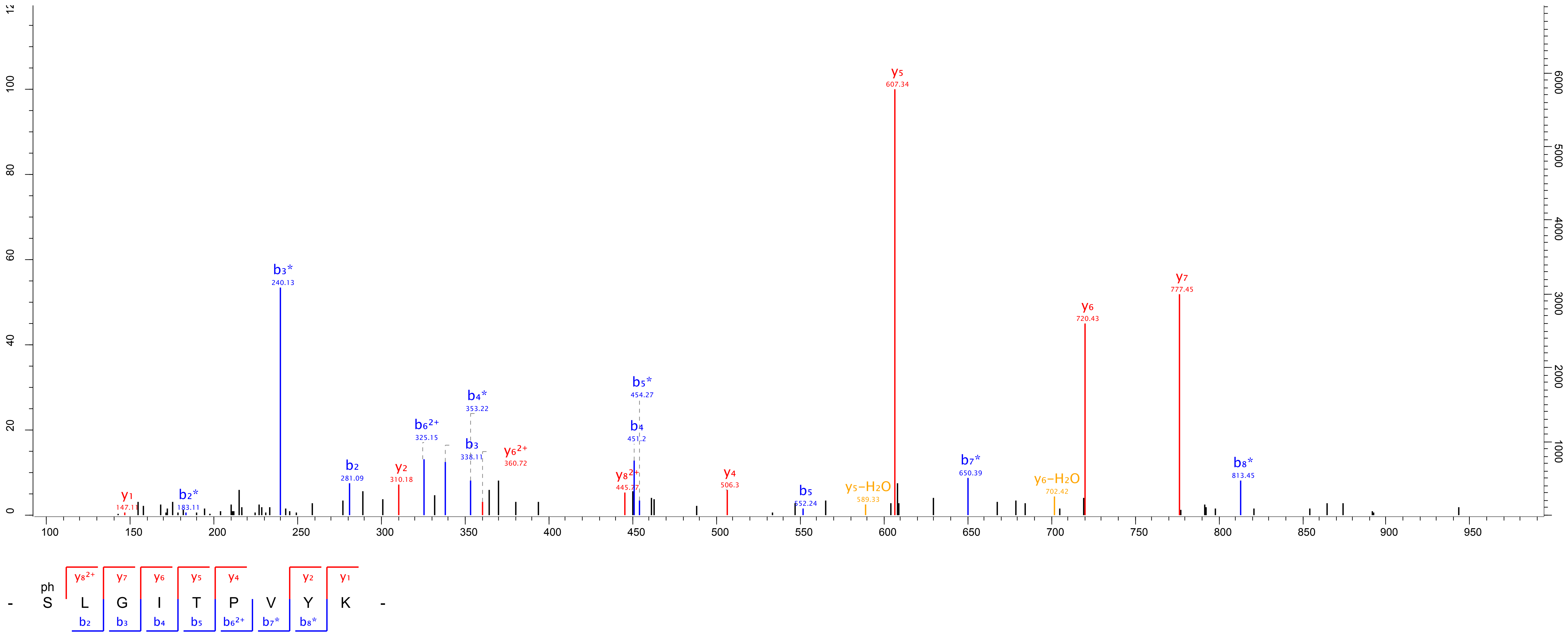

Raw file	Scan	Method	Score	m/z
20130708_PS_Phosphotest_6803wt_SDS03	8108	ITMS; CID	129.74	535.27

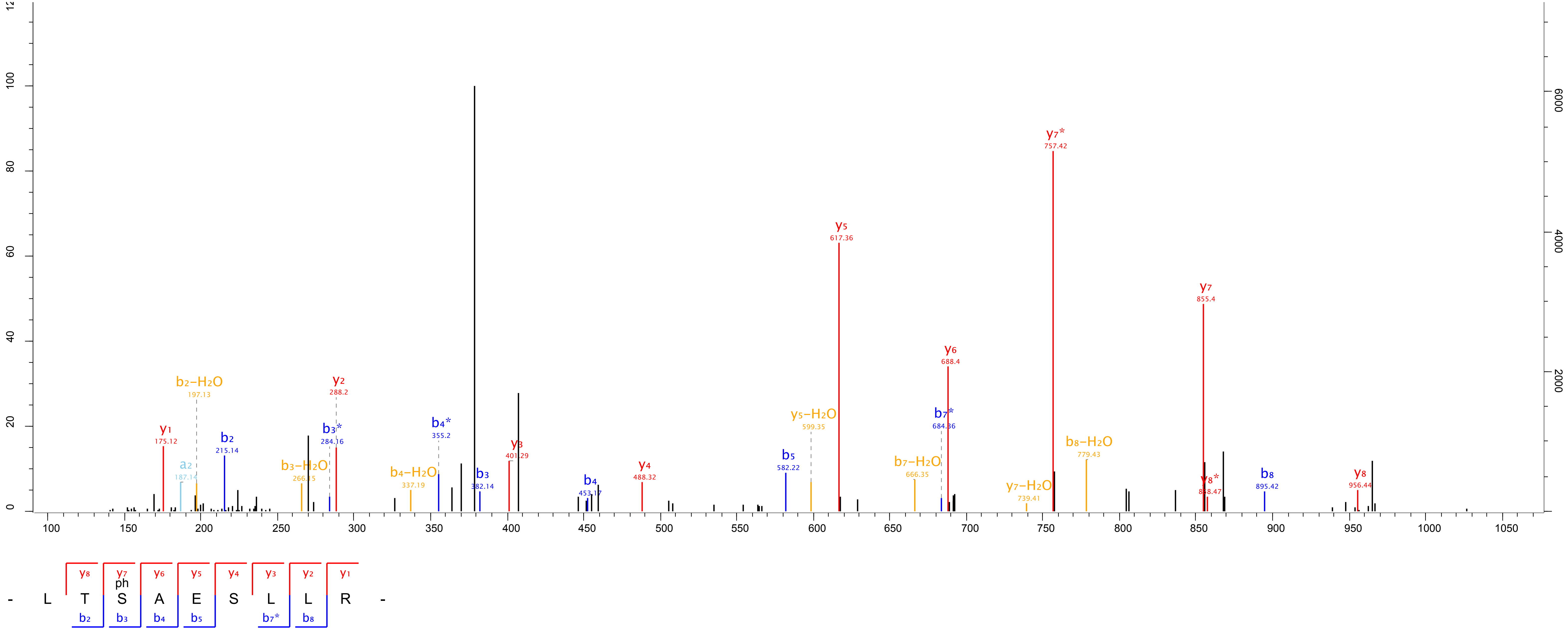

Raw file	Scan	Method	Score	m/z
20130708_PS_Phosphotest_6803wt_SDS01	8153	ITMS; CID	161.2	1019.94

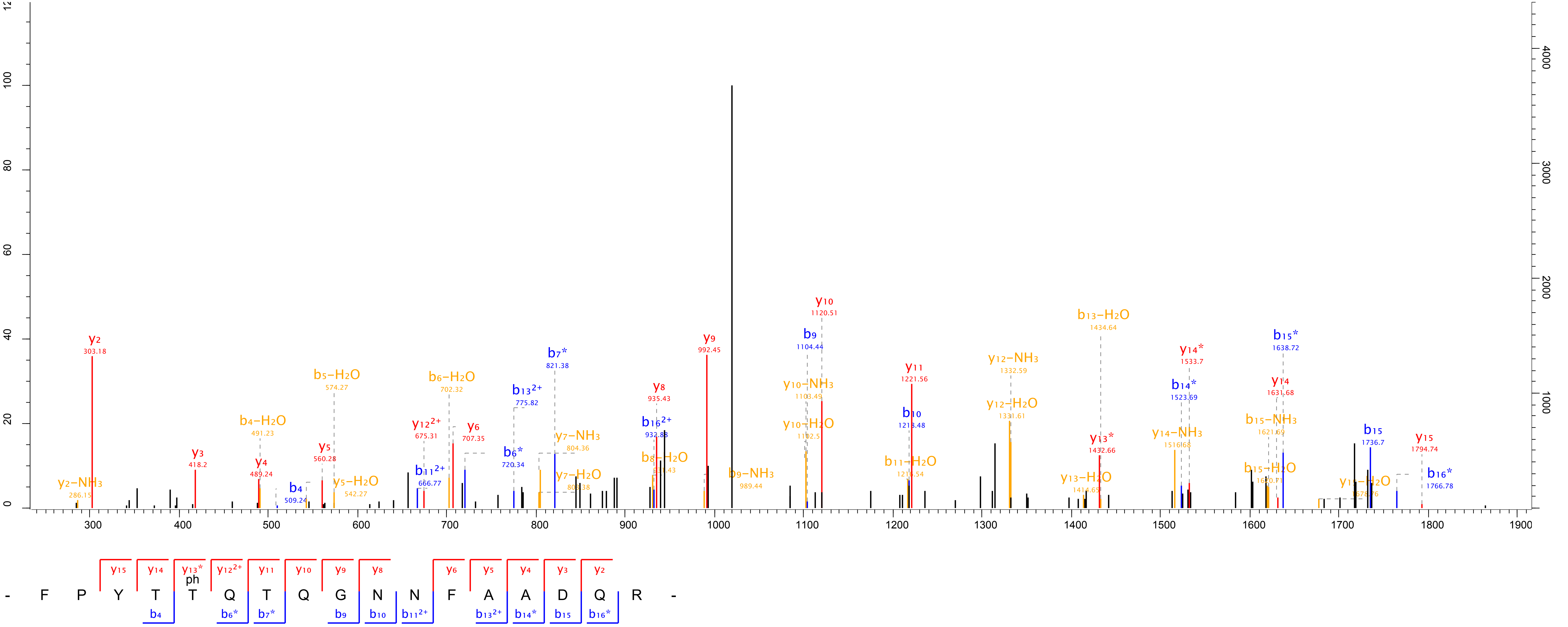

Raw file	Scan	Method	Score	m/z
20130708_PS_Phosphotest_6803wt_SDS01	8187	ITMS; CID	140.27	791.35

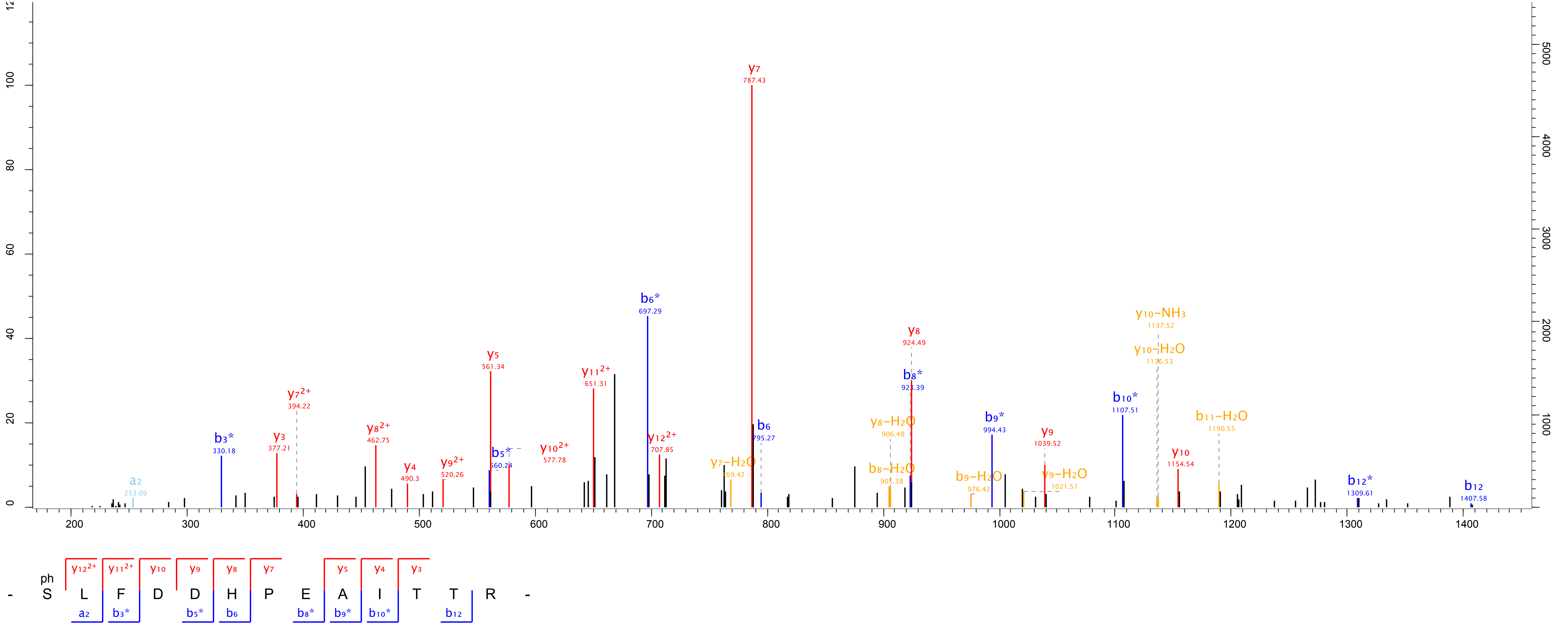

Raw file	Scan	Method	Score	m/z
20130708_PS_Phosphotest_6803wt_SDS01	8348	ITMS; CID	174.19	806.86

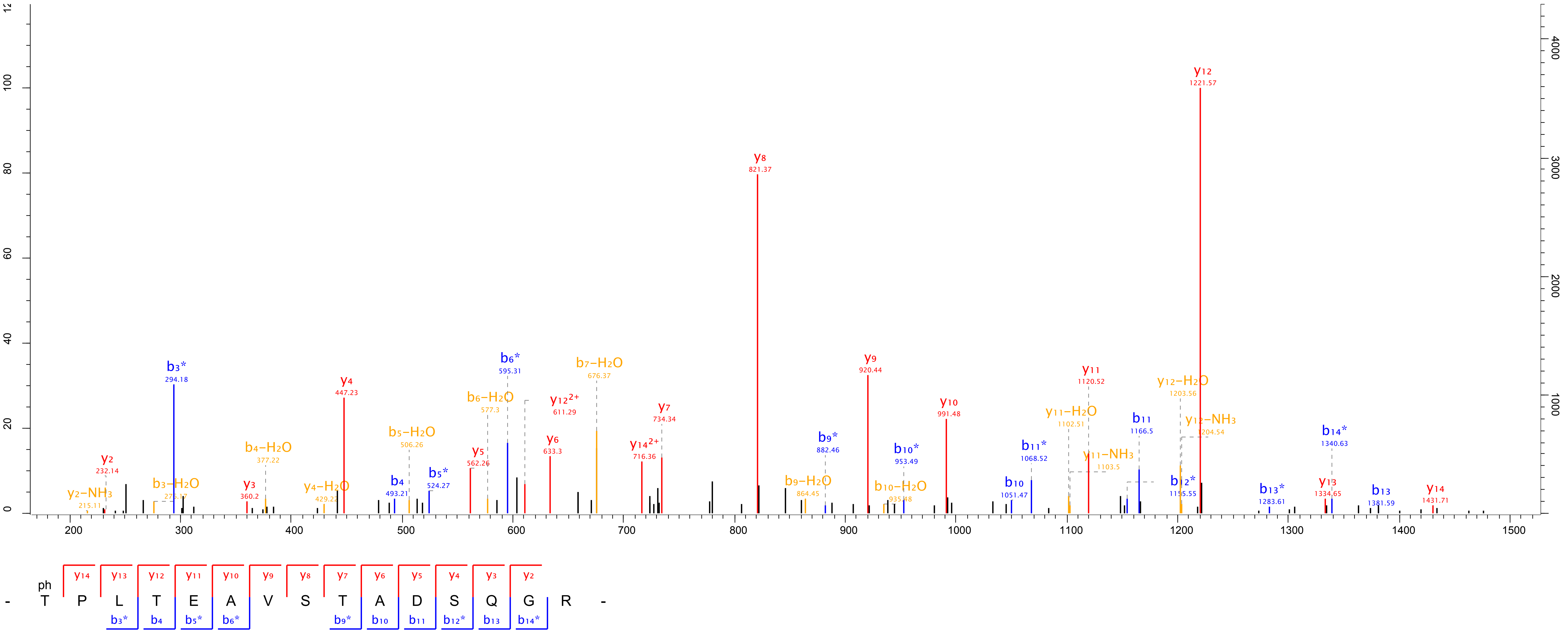

Raw file	Scan	Method	Score	m/z
20130708_PS_Phosphotest_6803wt_SDS01	8355	ITMS; CID	104.17	578.78

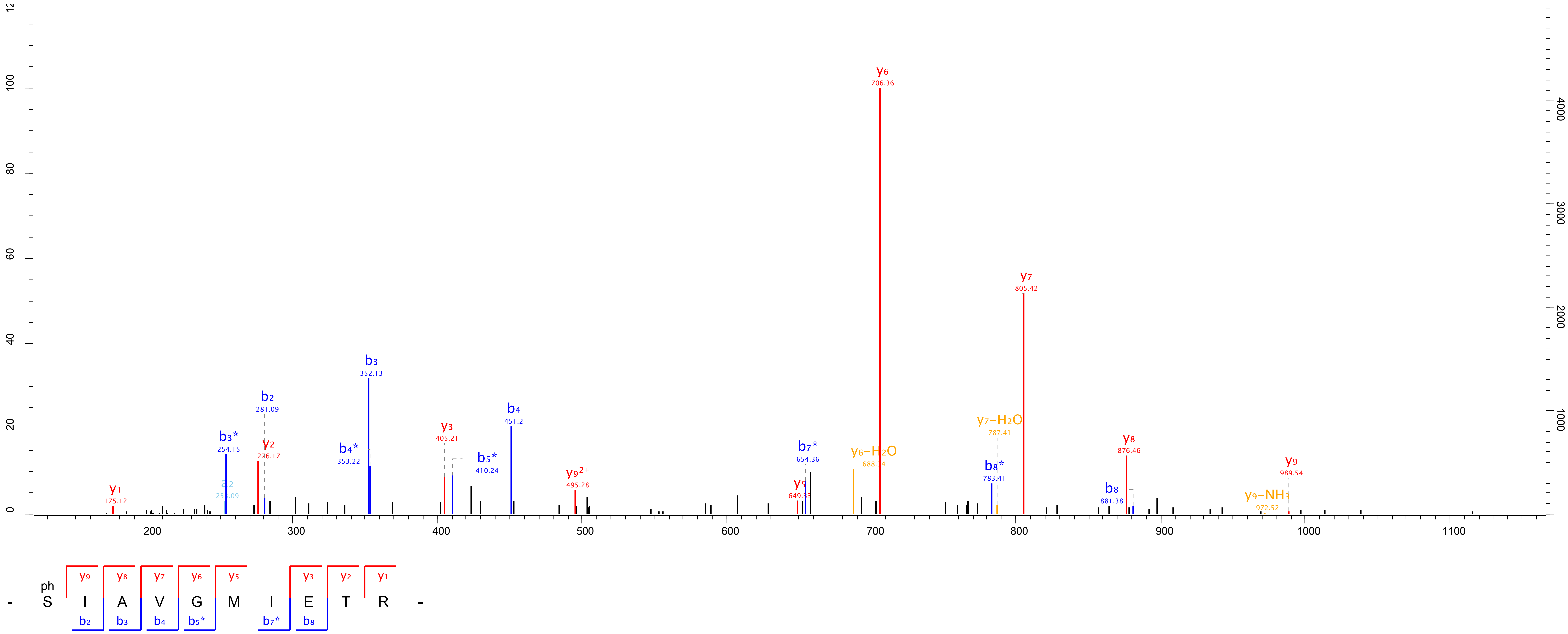

Raw file	Scan	Method	Score	m/z
20130708_PS_Phosphotest_6803wt_SDS01	8376	ITMS; CID	133.98	627.32

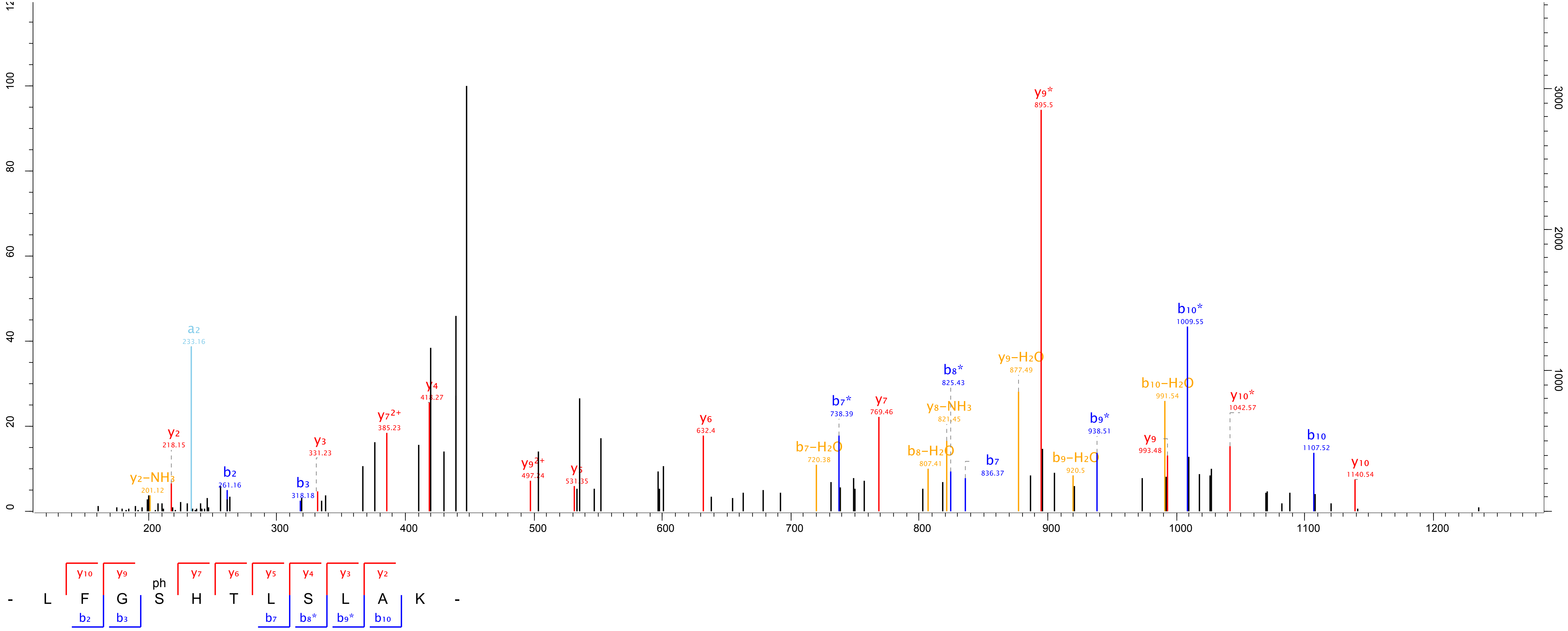

Raw file	Scan	Method	Score	m/z
20130708_PS_Phosphotest_6803wt_SDS01	8385	ITMS; CID	107.57	448.72

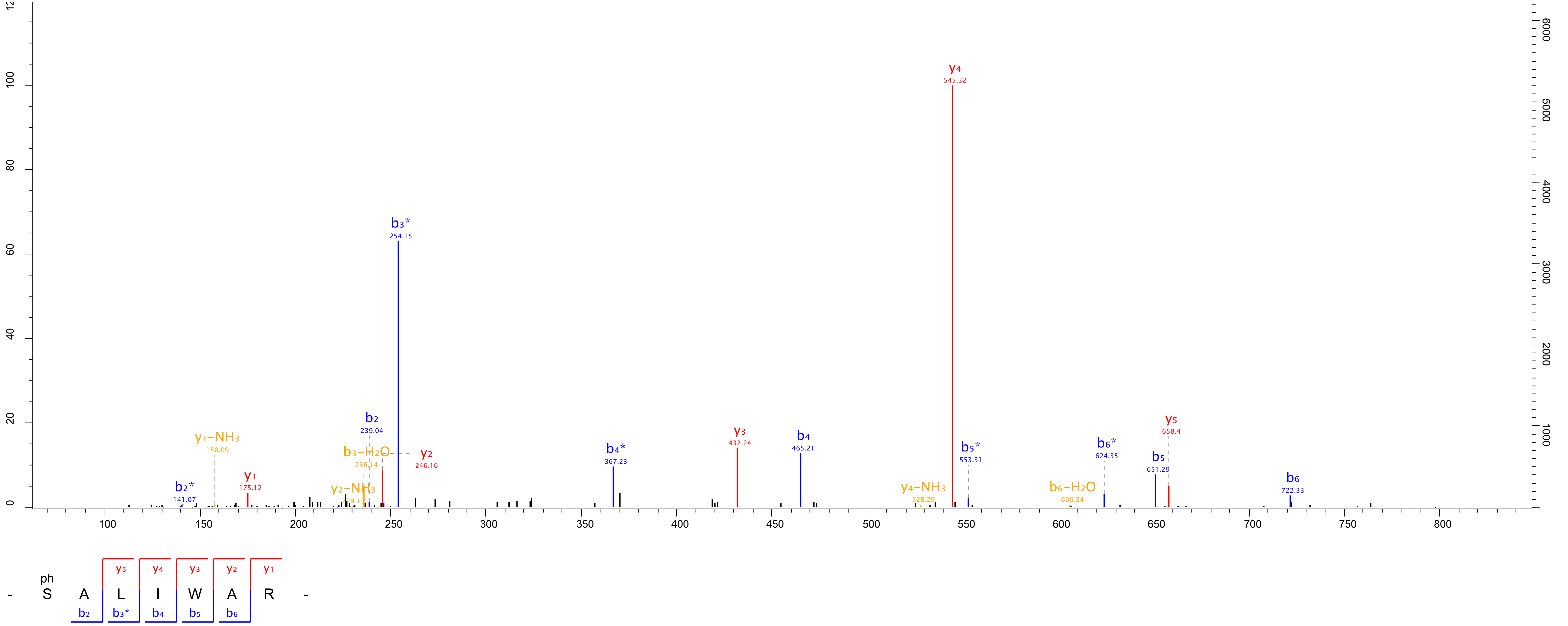

Raw file	Scan	Method	Score	m/z
20130708_PS_Phosphotest_6803wt_SDS01	8443	ITMS; CID	82.88	842.39

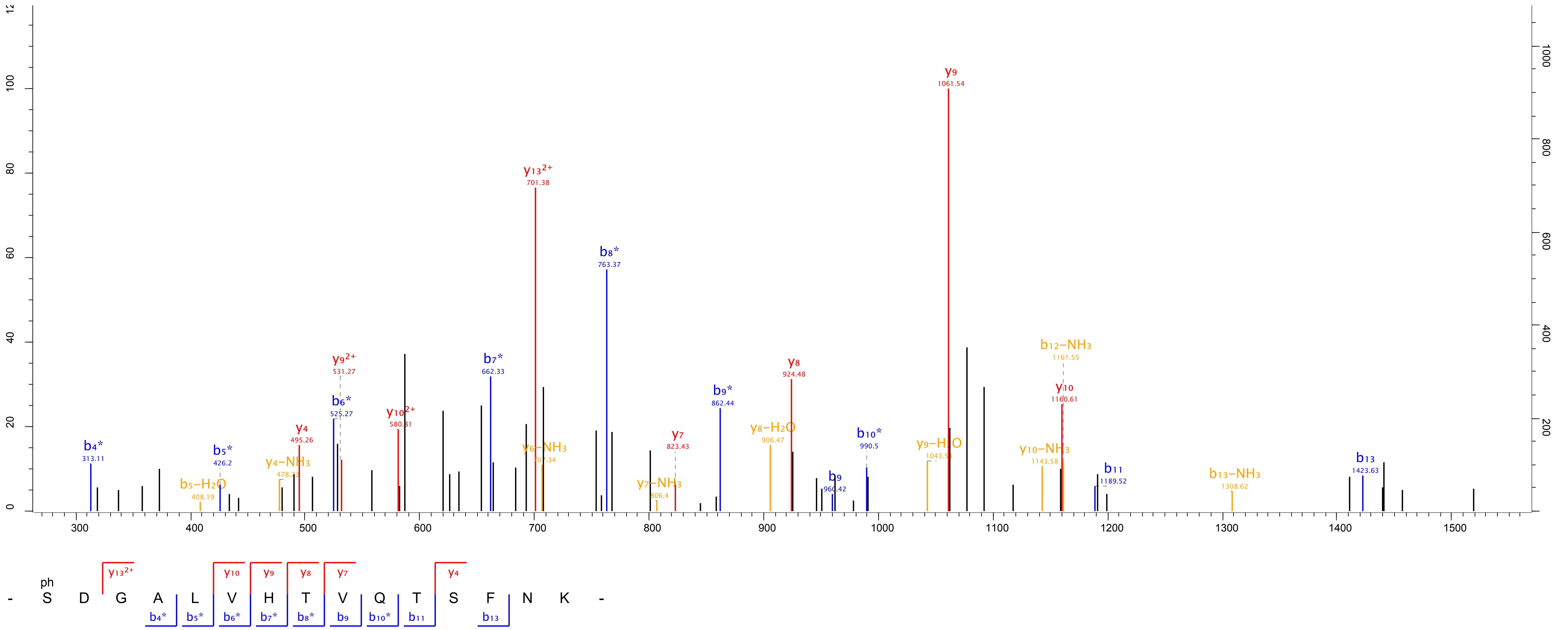

Raw file	Scan	Method	Score	m/z
20130708_PS_Phosphotest_6803wt_SDS01	8447	ITMS; CID	149.23	568.74

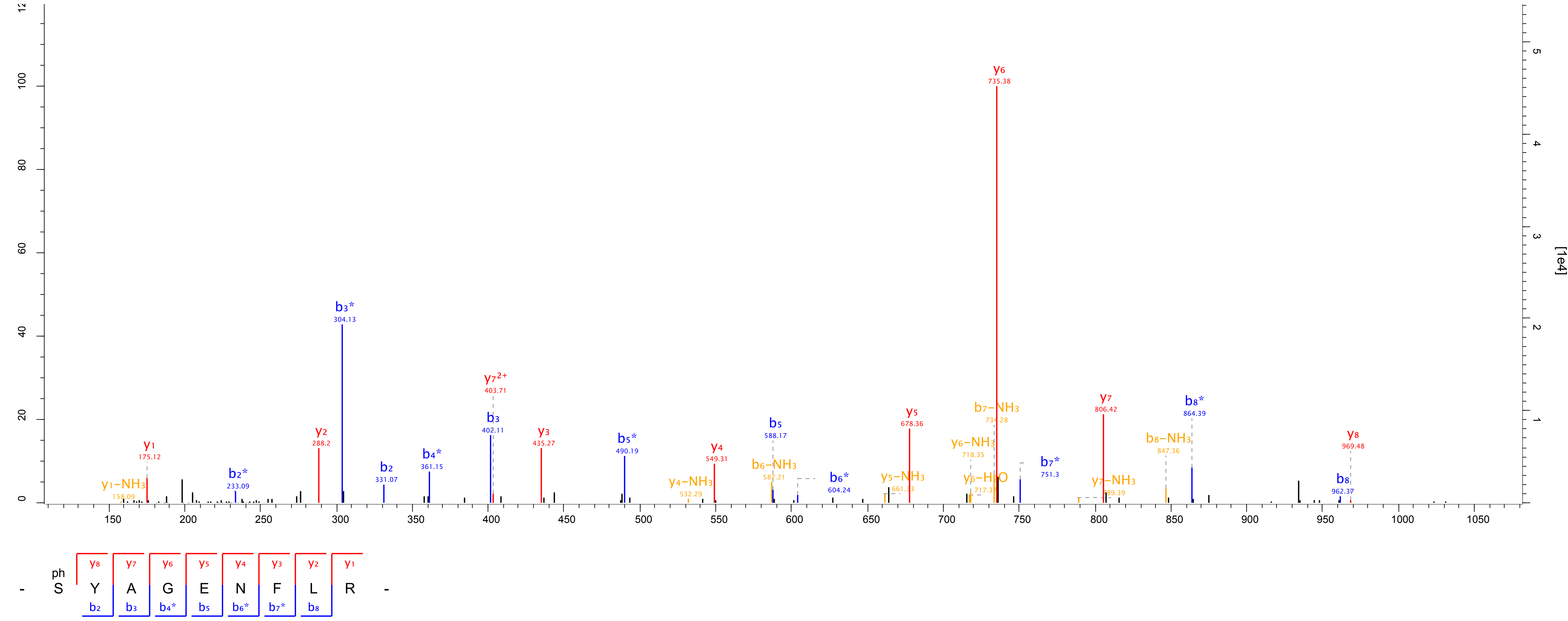

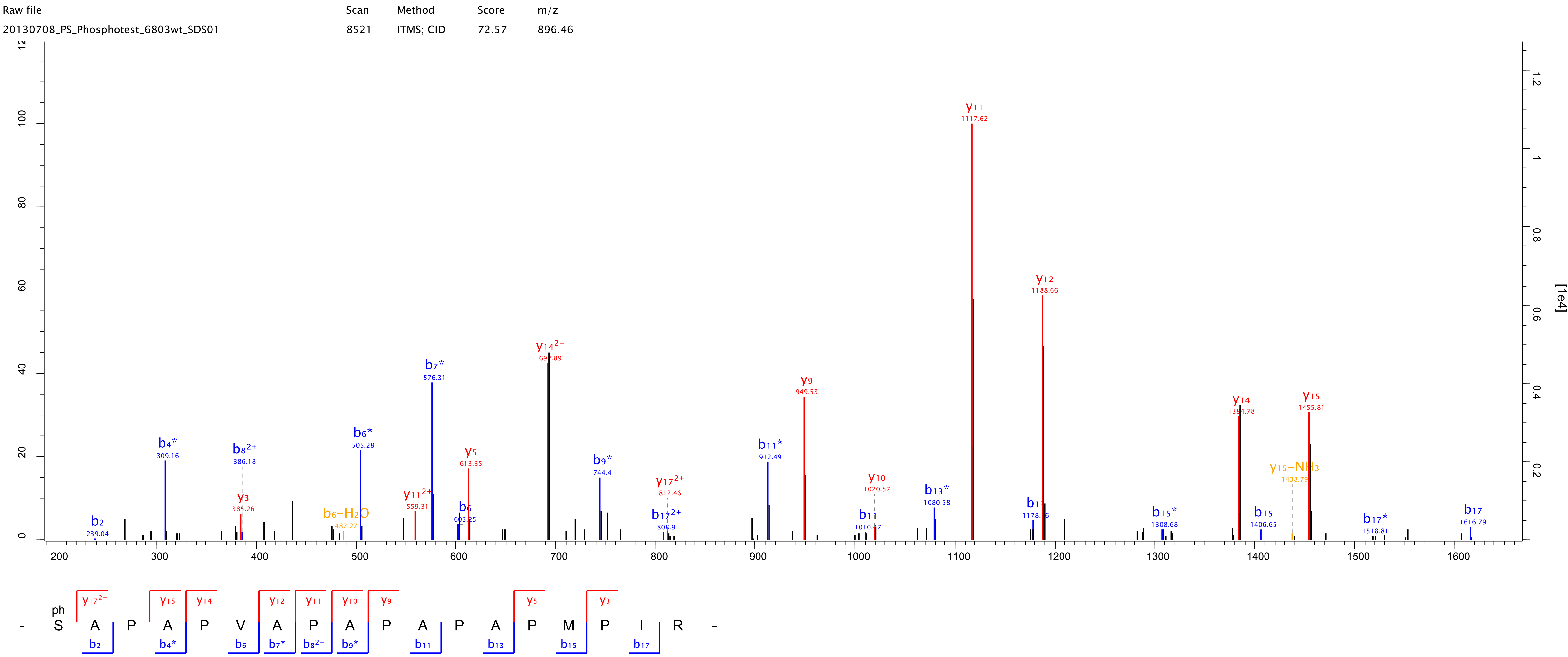
Raw file
20130708_PS_Phosphotest_6803wt_SDS01
Scan
8521
Method
ITMS; CID
Score
72.57
m/z
896.46
[1e4]
b2 239.04
b4* 309.16
y3 385.26
b8²⁺ 386.18
b6−H2O 487.27
b6* 505.28
y11²⁺ 559.31
b7* 576.31
b6 603.25
y5 613.35
y14²⁺ 692.89
b9* 744.4
b17²⁺ 808.9
y17²⁺ 812.46
b11* 912.49
y9 949.53
b11 1010.47
y10 1020.57
b13* 1080.58
y11 1117.62
y12 1188.66
b15* 1308.68
y14 1384.78
b15 1406.65
y15−NH3 1438.79
y15 1455.81
b17* 1518.81
b17 1616.79
- S A P A P V A P A P A P A P M P I R -
ph
y17²⁺ y15 y14 y12 y11 y10 y9 y5 y3
b2 b4* b6 b7* b8²⁺ b9* b11 b13 b15 b17

Raw file	Scan	Method	Score	m/z
20130708_PS_Phosphotest_6803wt_SDS01	8612	ITMS; CID	128.1	849.42

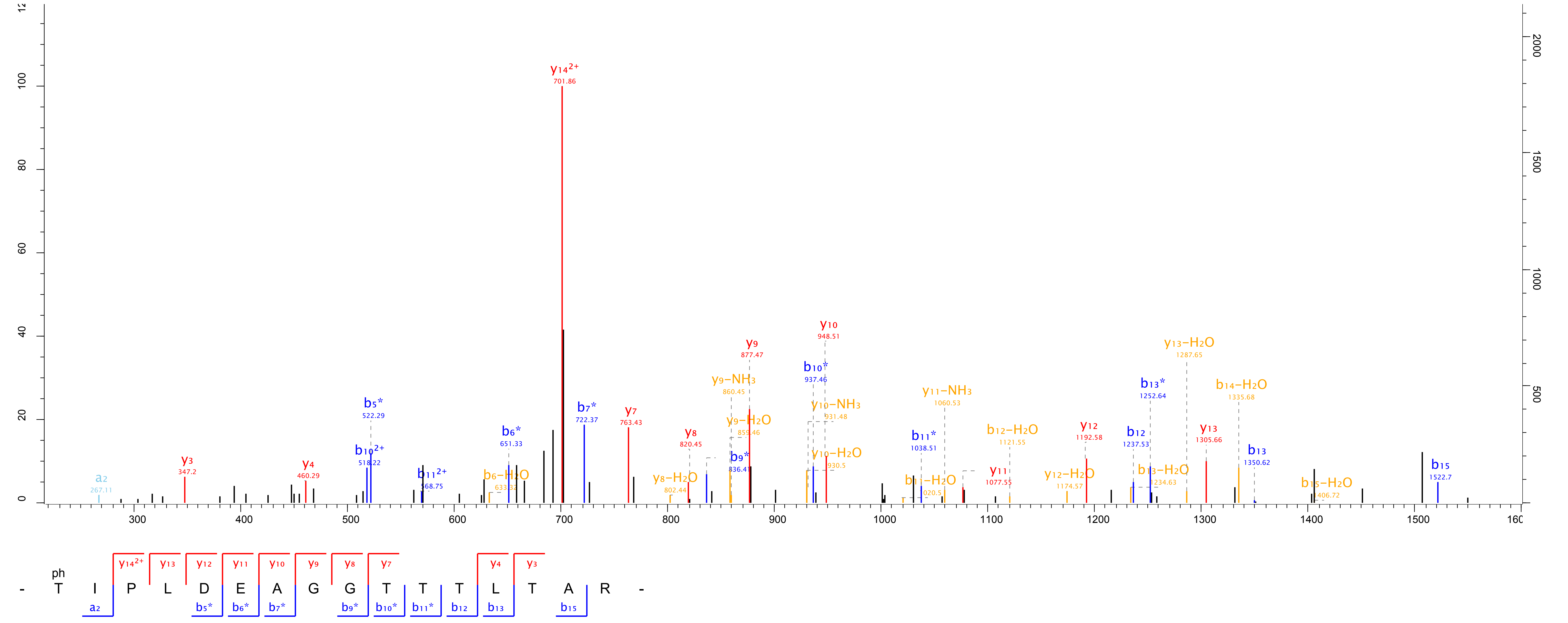

Raw file	Scan	Method	Score	m/z
20130708_PS_Phosphotest_6803wt_SDS01	8798	ITMS; CID	115.04	637.79

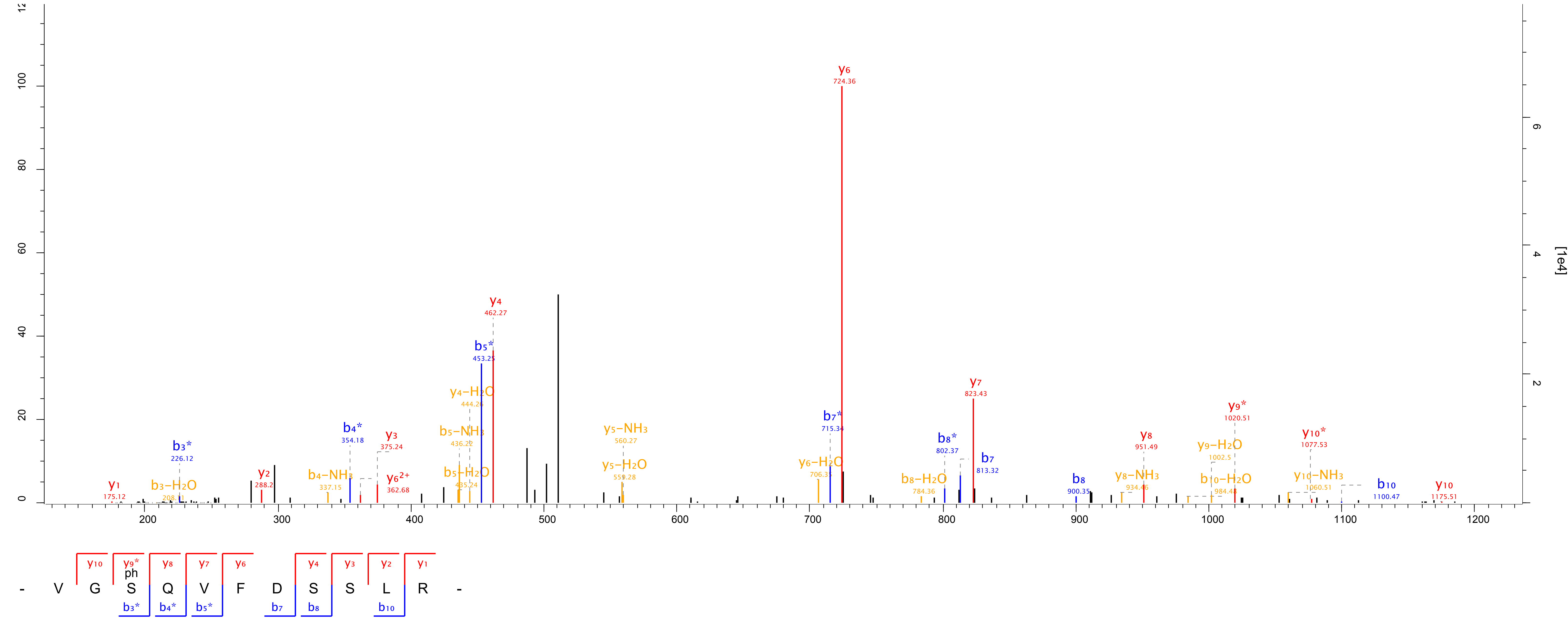

Raw file	Scan	Method	Score	m/z
20130708_PS_Phosphotest_6803wt_SDS01	8813	ITMS; CID	104.7	612.8

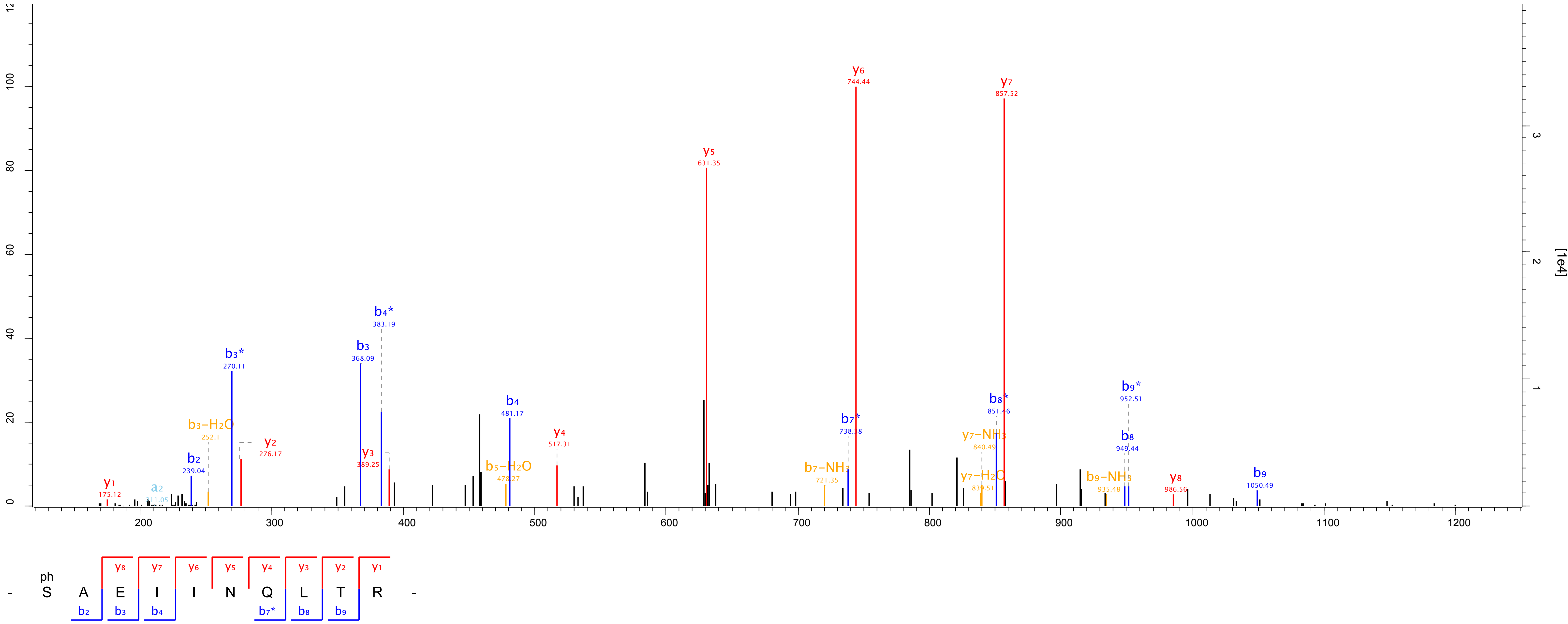

Raw file	Scan	Method	Score	m/z
20130708_PS_Phosphotest_6803wt_SDS01	8941	ITMS; CID	202.63	810.38

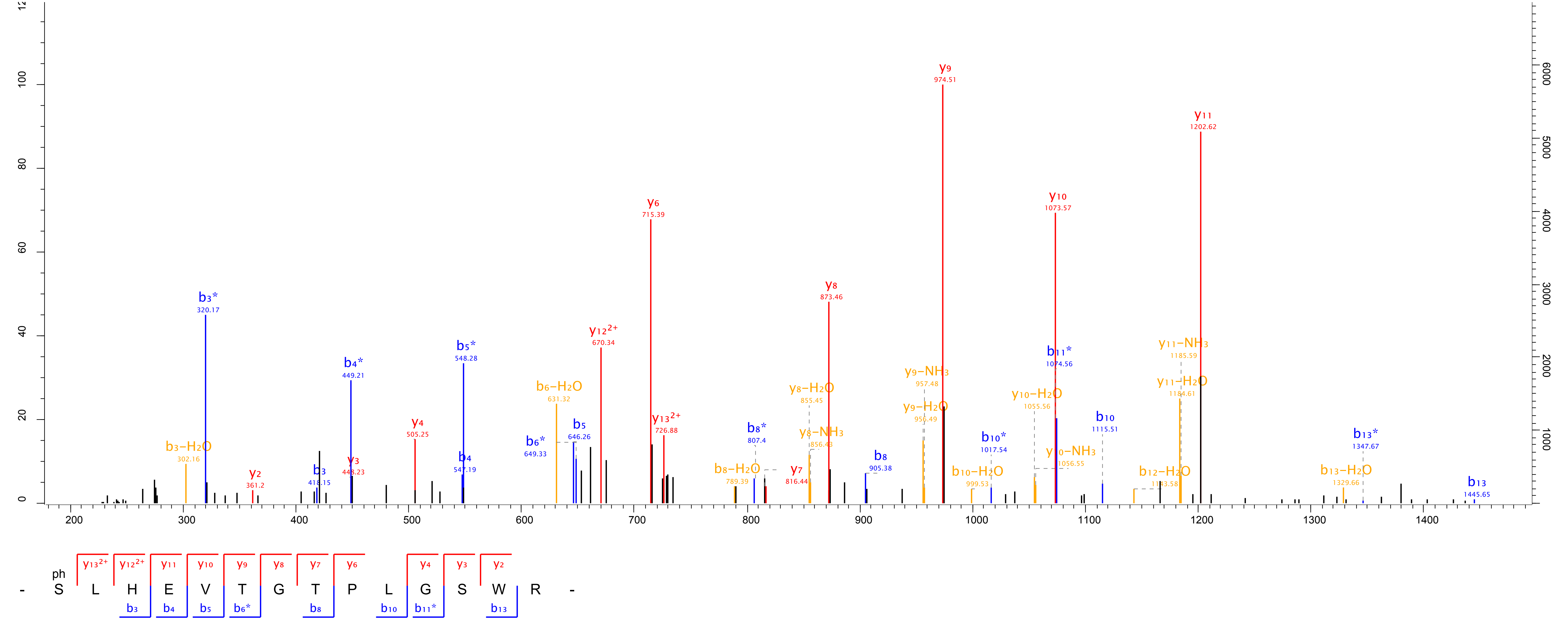

Raw file	Scan	Method	Score	m/z
20130708_PS_Phosphotest_6803wt_SDS01	8991	ITMS; CID	131.86	866.44

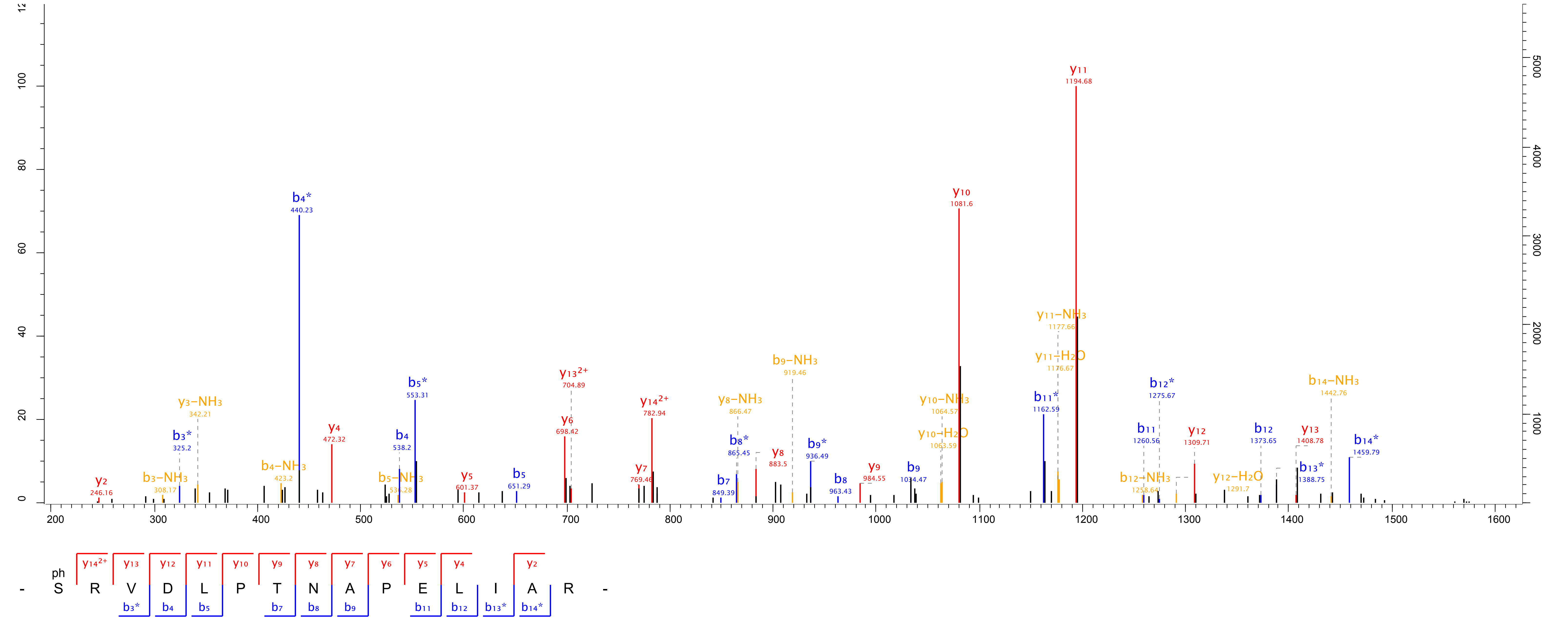

Raw file	Scan	Method	Score	m/z
20130708_PS_Phosphotest_6803wt_SDS01	9005	ITMS; CID	74.96	491.26

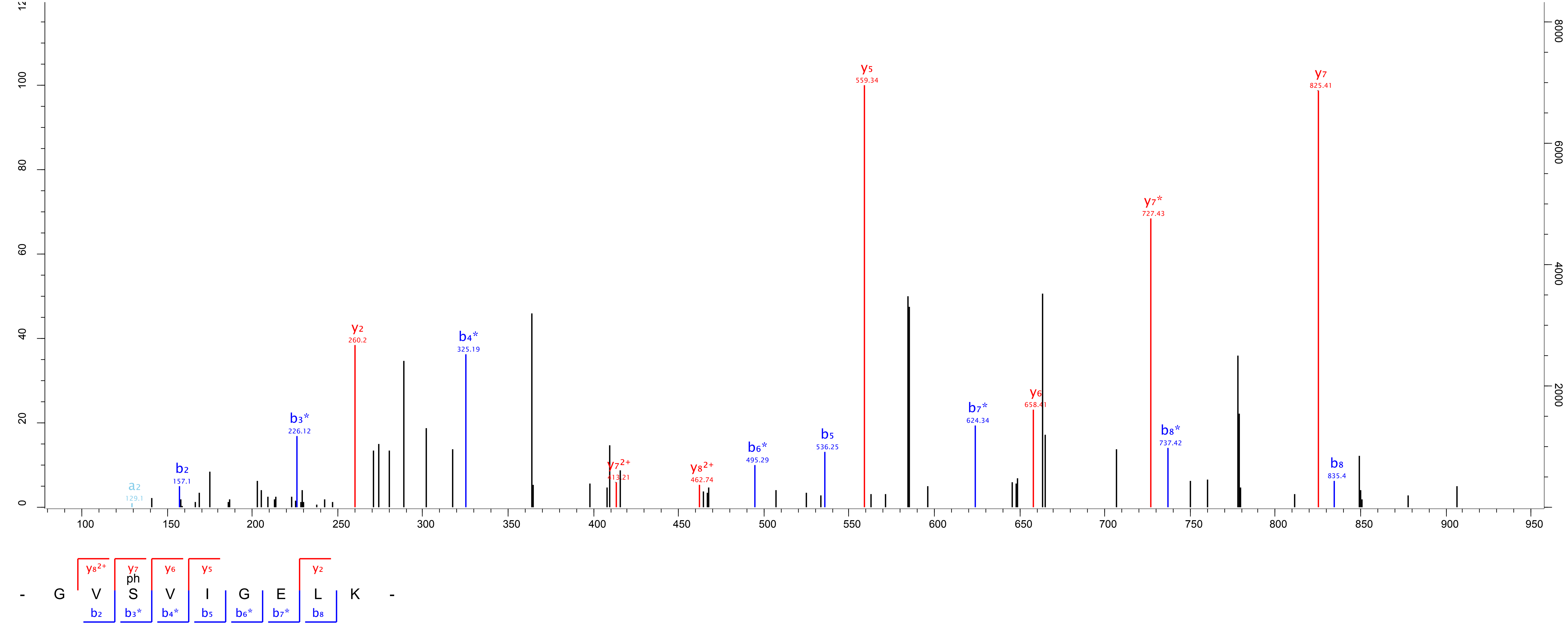

Raw file	Scan	Method	Score	m/z
20130708_PS_Phosphotest_6803wt_YPER01	9020	ITMS; CID	174.78	850.39

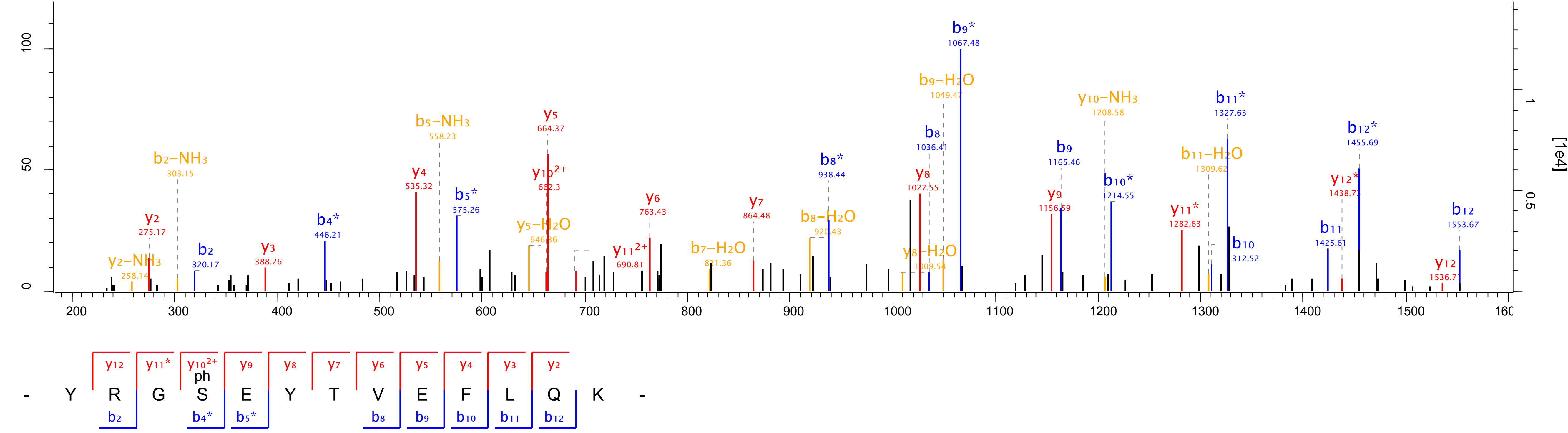

Raw file	Scan	Method	Score	m/z
20130708_PS_Phosphotest_6803wt_YPER01	9020	ITMS; CID	174.78	850.39

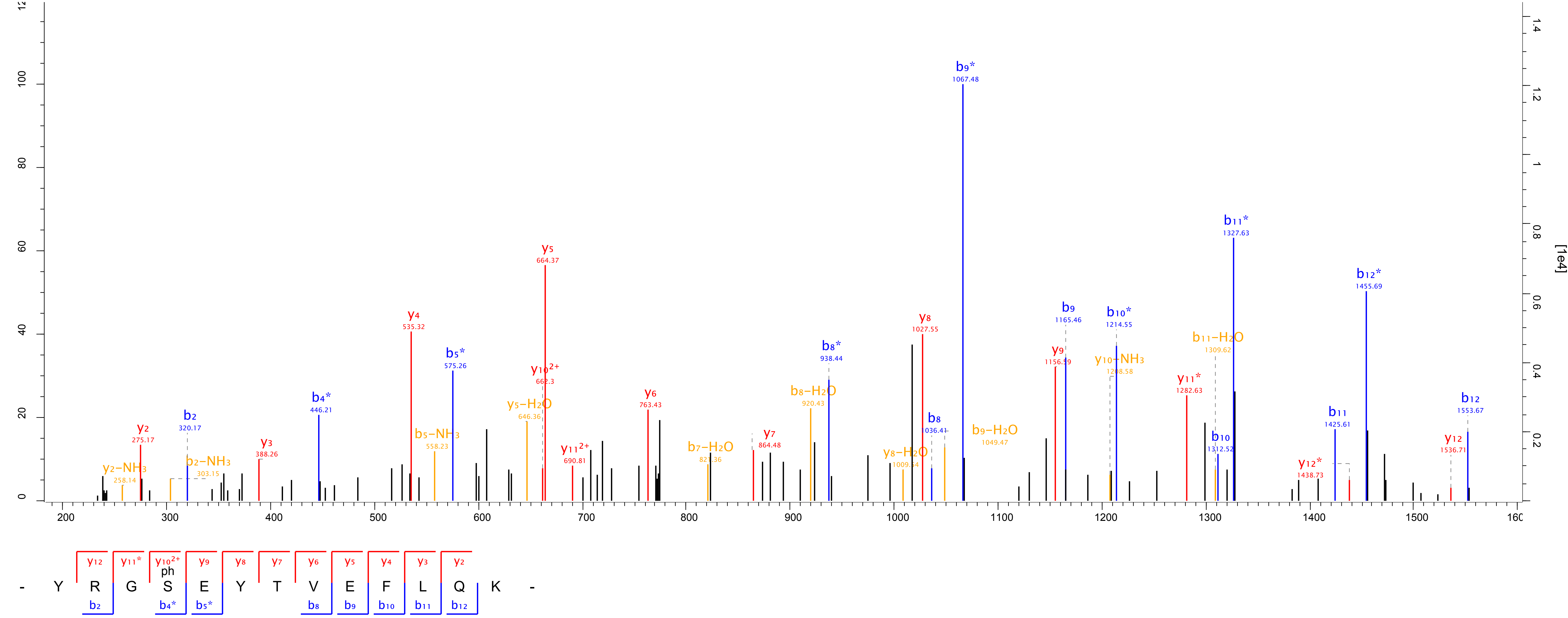

Raw file	Scan	Method	Score	m/z
20130708_PS_Phosphotest_6803wt_SDS01	9061	ITMS; CID	132.65	768.85

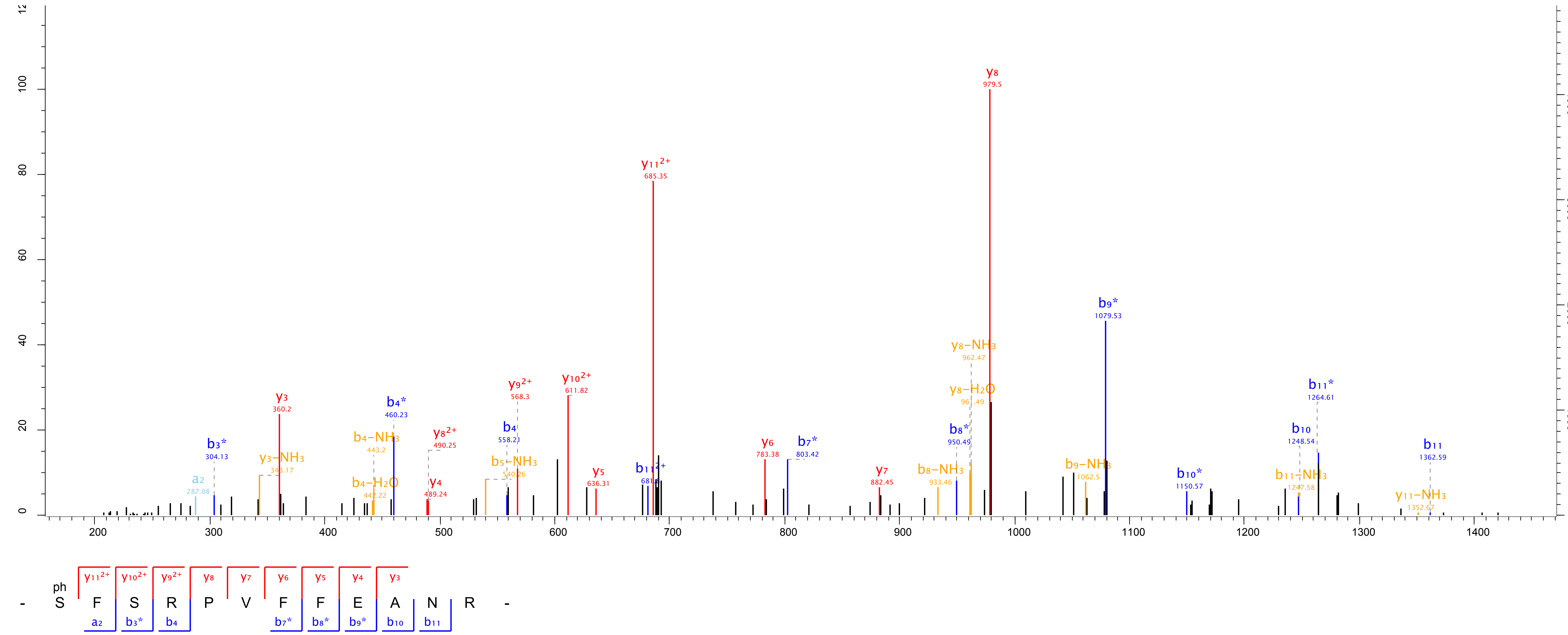

Raw file	Scan	Method	Score	m/z
20130708_PS_Phosphotest_6803wt_SDS01	9084	ITMS; CID	179.42	988.95

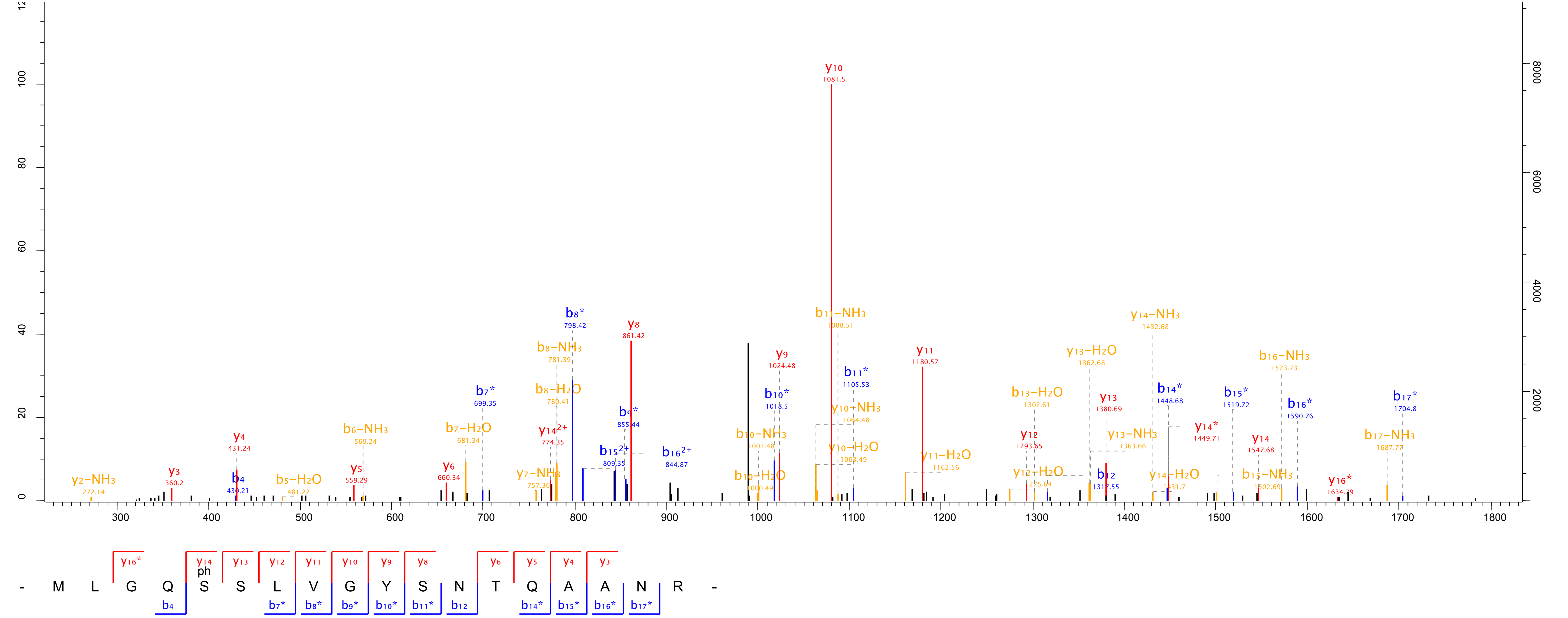

Raw file	Scan	Method	Score	m/z
20130708_PS_Phosphotest_6803wt_SDS01	9086	ITMS; CID	57.48	659.97

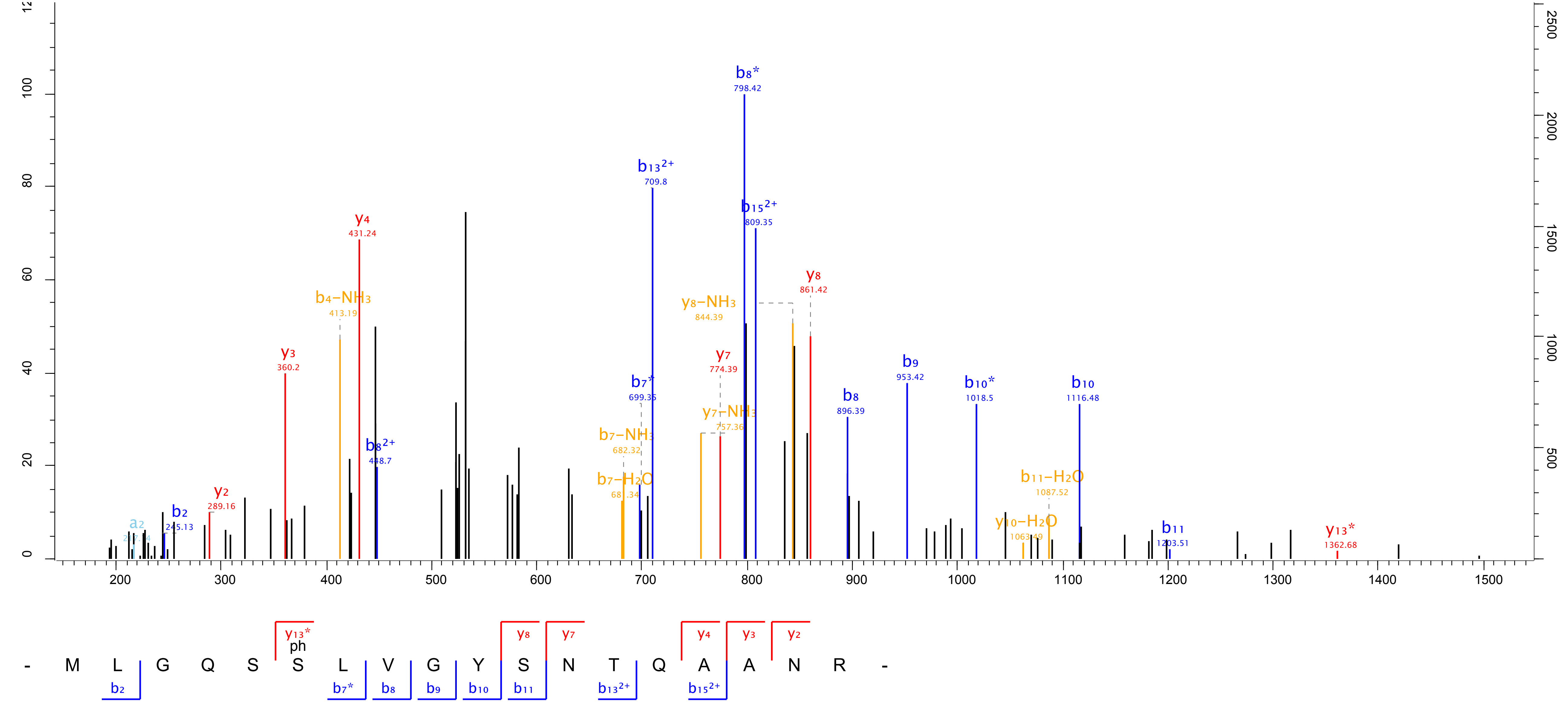

Raw file	Scan	Method	Score	m/z
20130708_PS_Phosphotest_6803wt_SDS02	9205	ITMS; CID	123.28	607.3

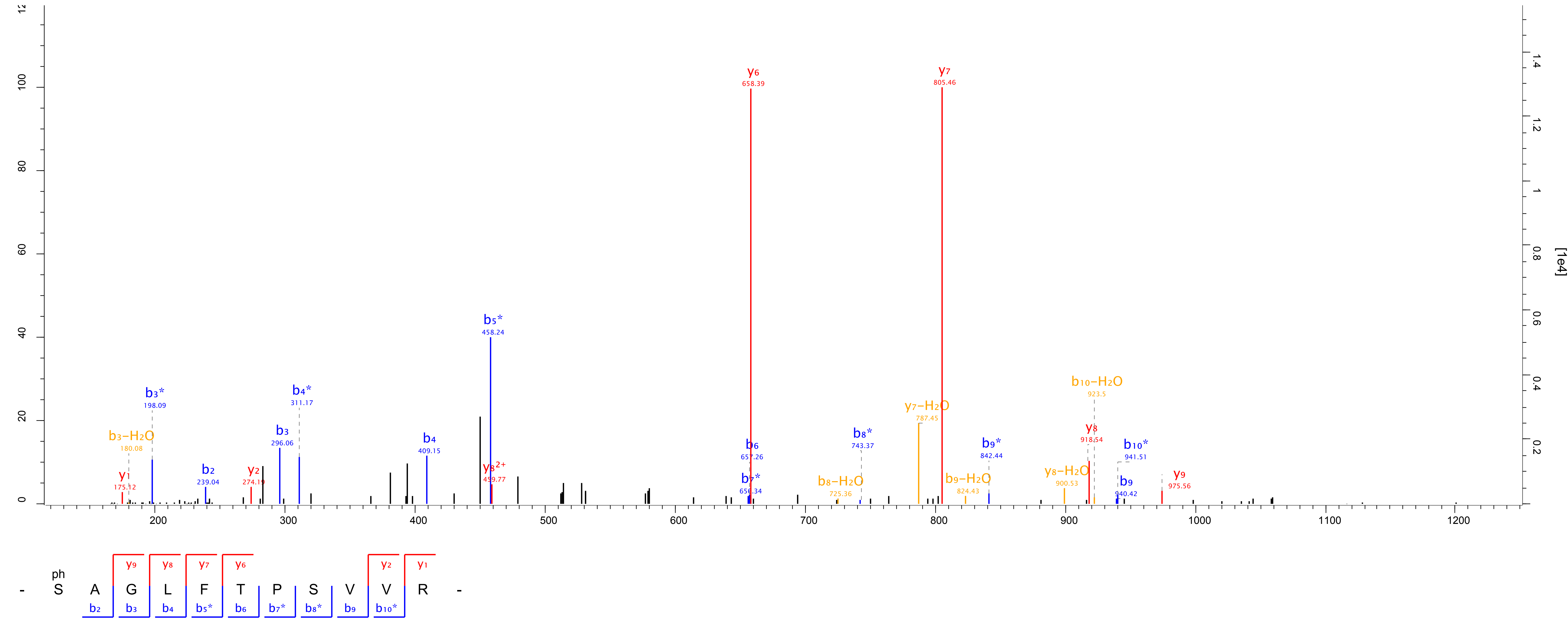

Raw file	Scan	Method	Score	m/z
20130708_PS_Phosphotest_6803wt_SDS01	9245	ITMS; CID	98	593.77

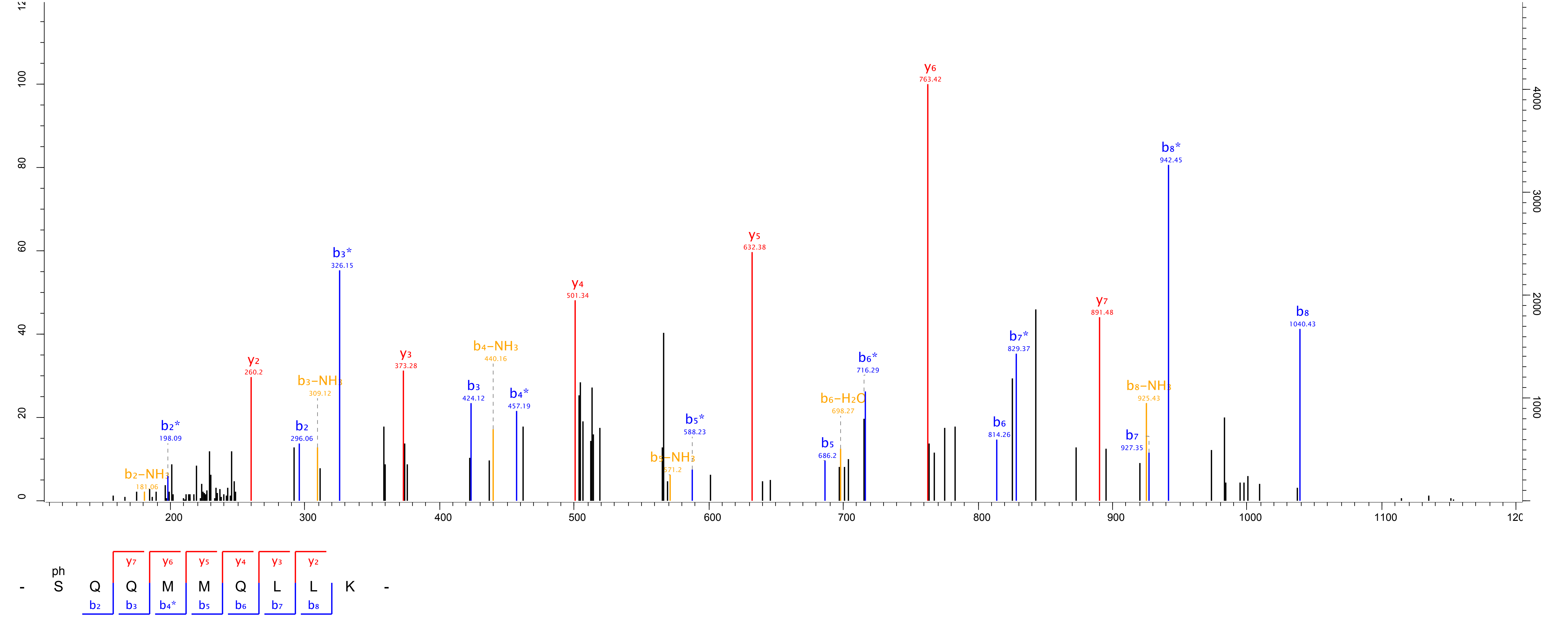

Raw file	Scan	Method	Score	m/z
20130708_PS_Phosphotest_6803wt_SDS01	9258	ITMS; CID	72.29	574.31

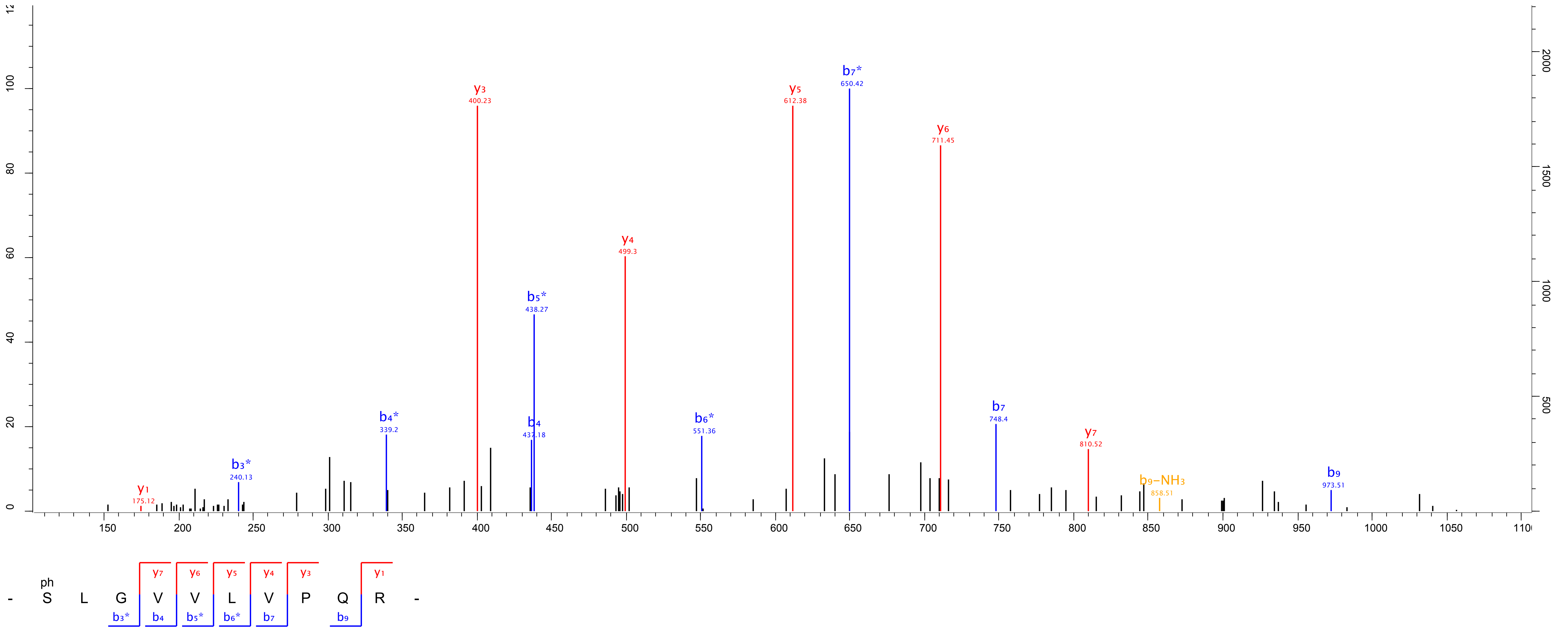

Raw file	Scan	Method	Score	m/z
20130708_PS_Phosphotest_6803wt_SDS01	9308	ITMS; CID	119.68	734.87

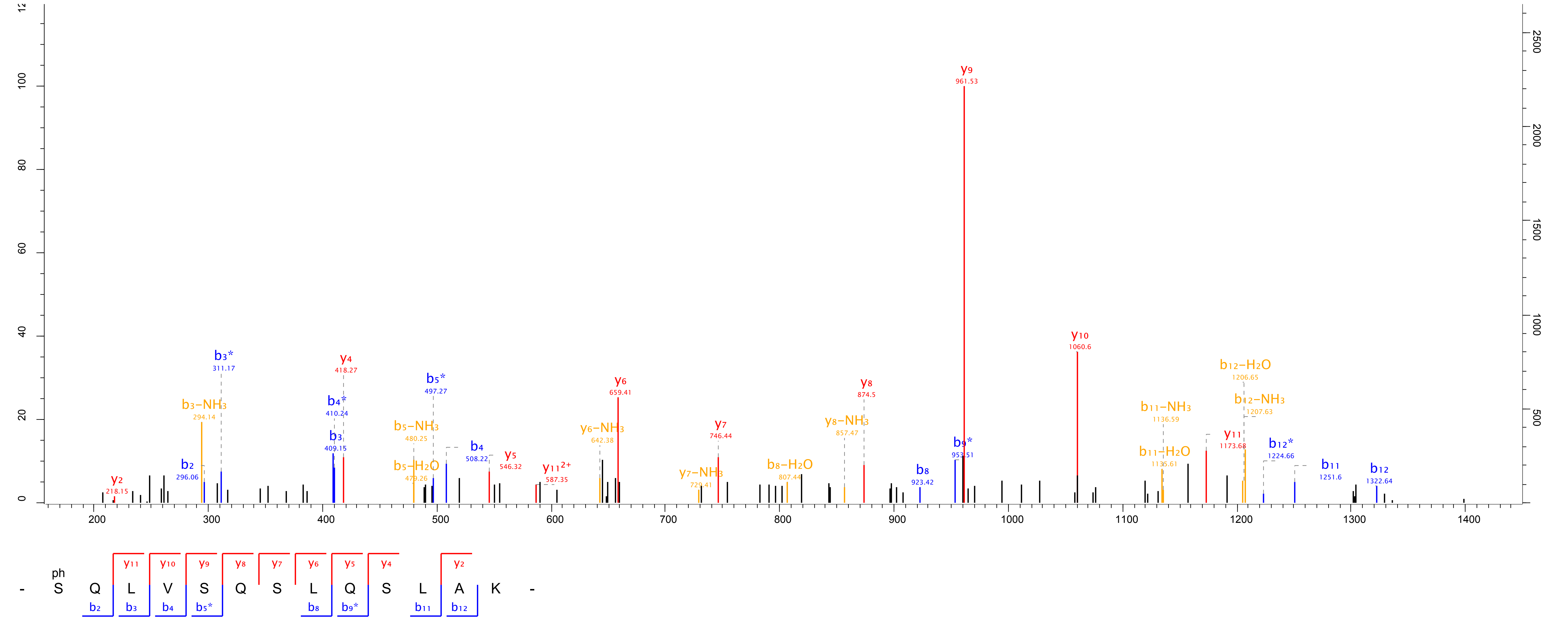

Raw file	Scan	Method	Score	m/z
20130708_PS_Phosphotest_6803wt_YPER01	9442	ITMS; CID	78.9	763.88

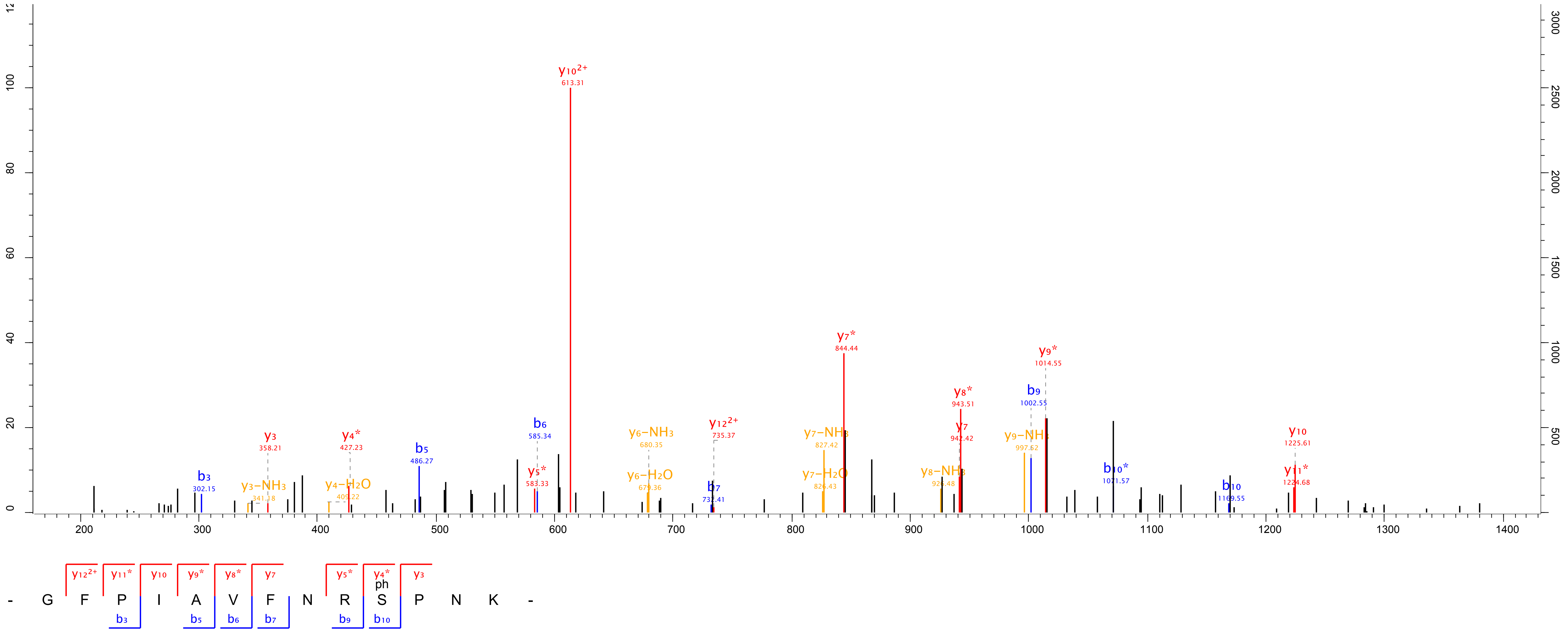

Raw file	Scan	Method	Score	m/z
20130708_PS_Phosphotest_6803wt_YPER01	9478	ITMS; CID	89.8	574.81

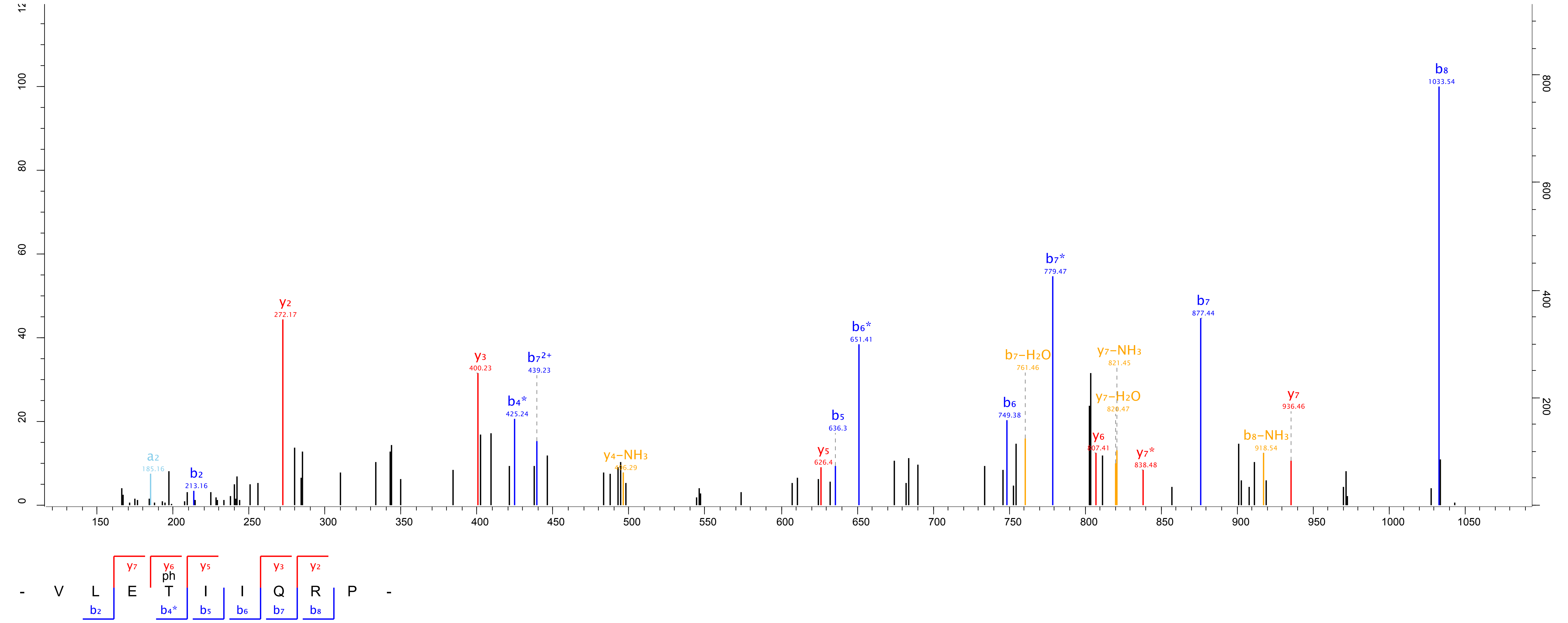

Raw file	Scan	Method	Score	m/z
20130708_PS_Phosphotest_6803wt_SDS01	9503	ITMS; CID	86.54	690.81

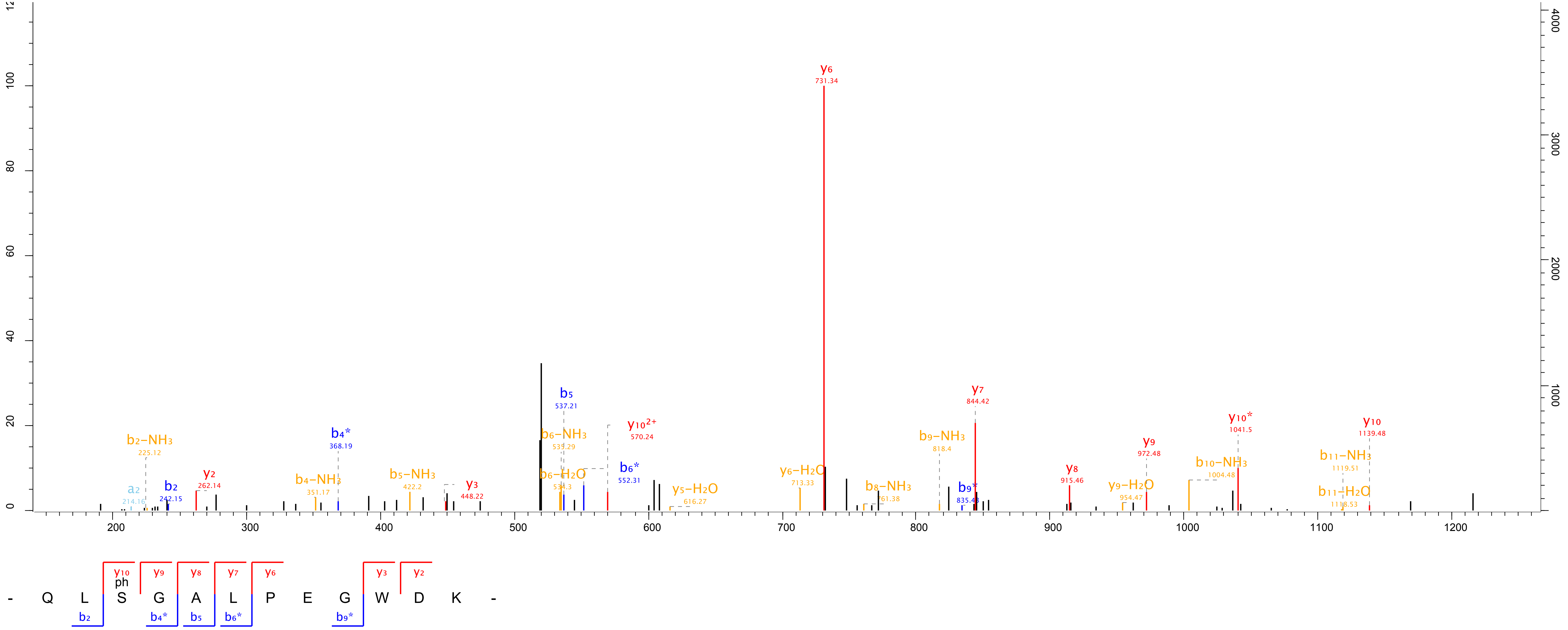

Raw file	Scan	Method	Score	m/z
20130708_PS_Phosphotest_6803wt_SDS01	9513	ITMS; CID	117	1046.99

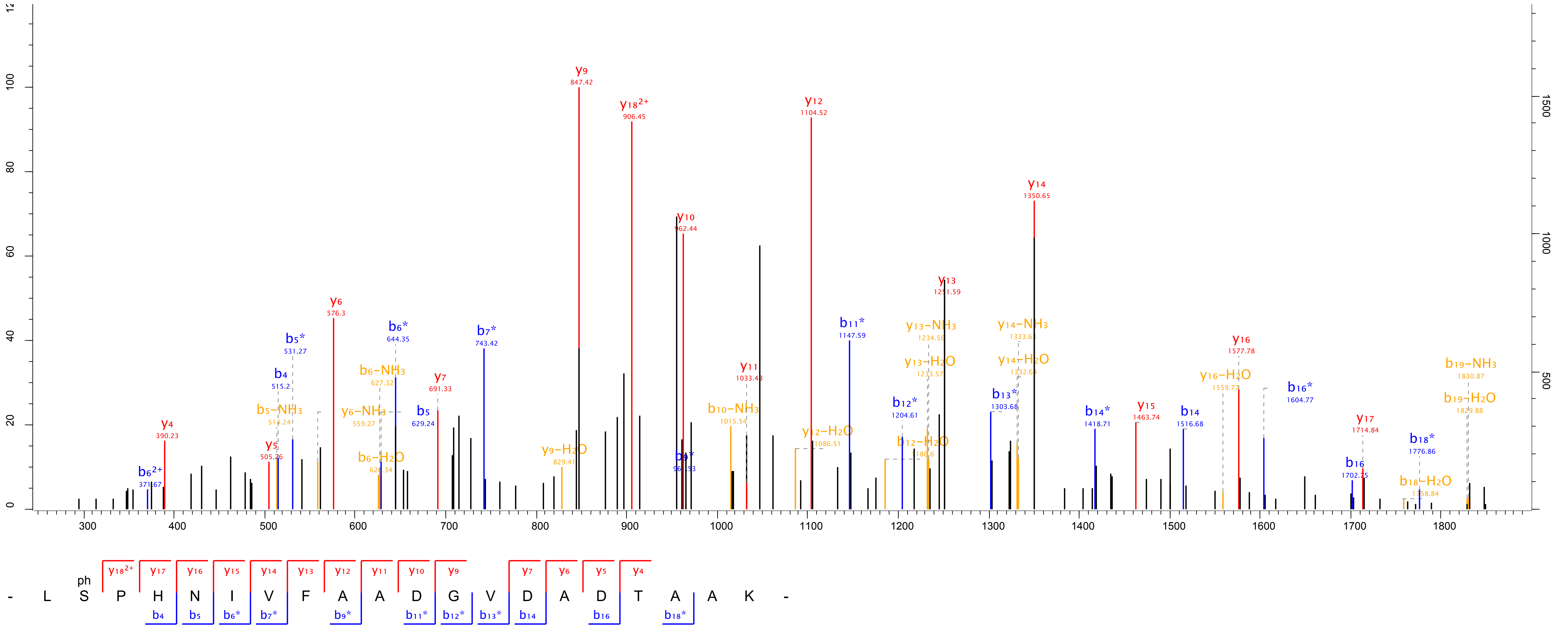

Raw file	Scan	Method	Score	m/z
20130708_PS_Phosphotest_6803wt_SDS01	9535	ITMS; CID	148.41	668.83

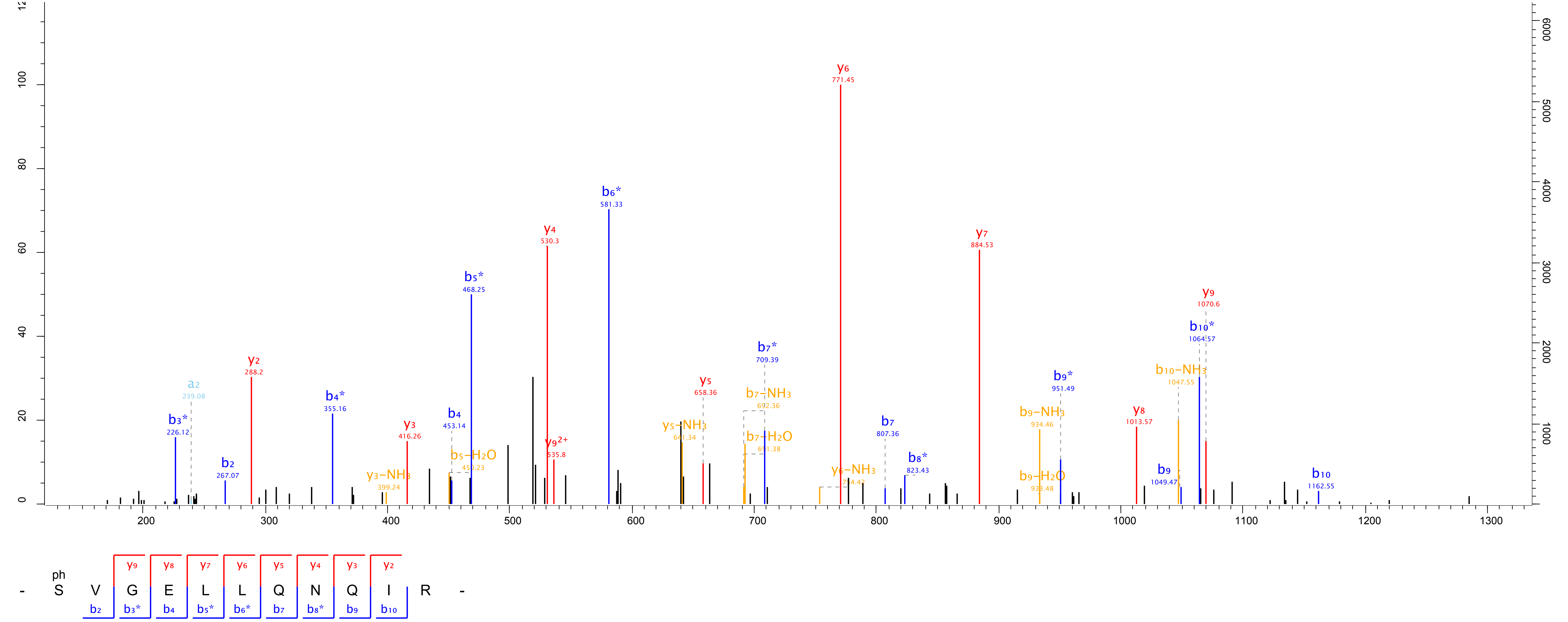

Raw file	Scan	Method	Score	m/z
20130708_PS_Phosphotest_6803wt_SDS01	9537	ITMS; CID	197.28	856.92

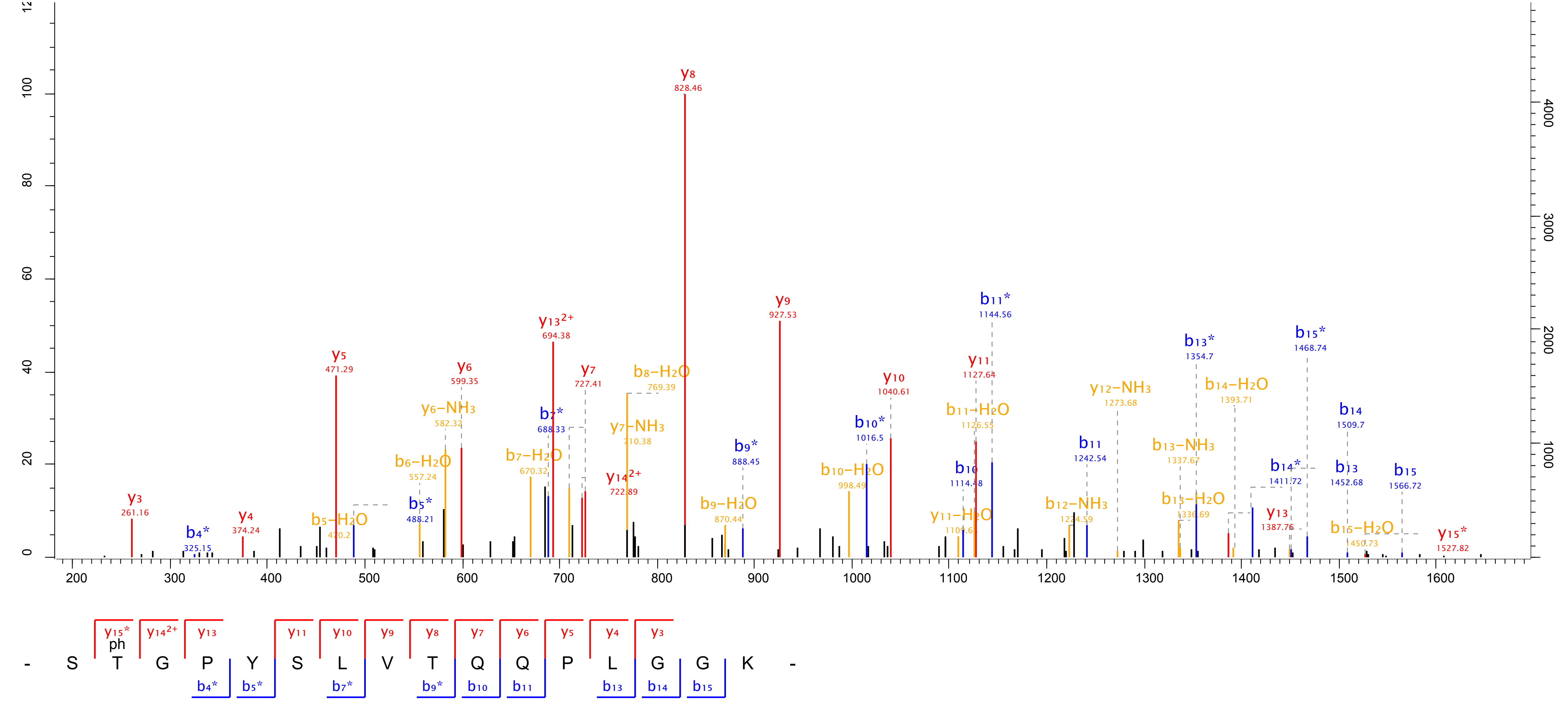

Raw file	Scan	Method	Score	m/z
20130708_PS_Phosphotest_6803wt_SDS01	9590	ITMS; CID	96.03	454.21

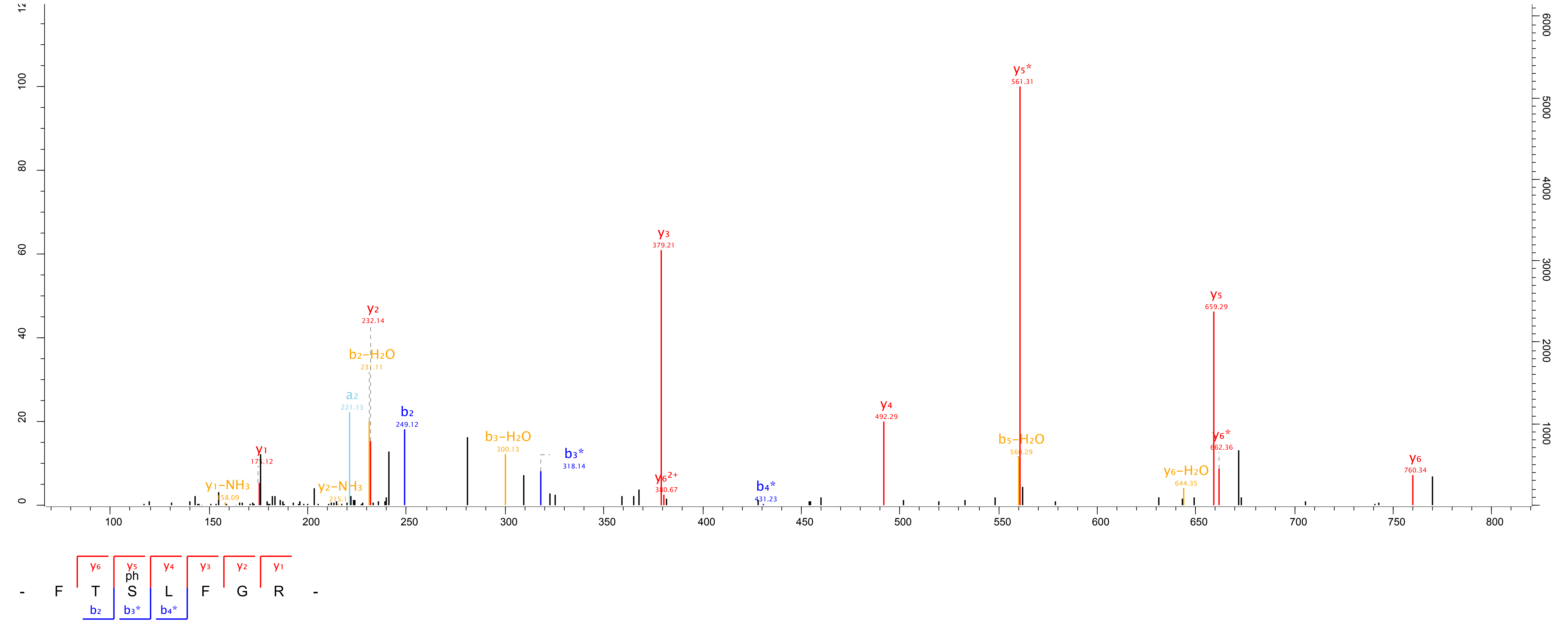

Raw file	Scan	Method	Score	m/z
20130708_PS_Phosphotest_6803wt_SDS01	9685	ITMS; CID	231.39	961.96

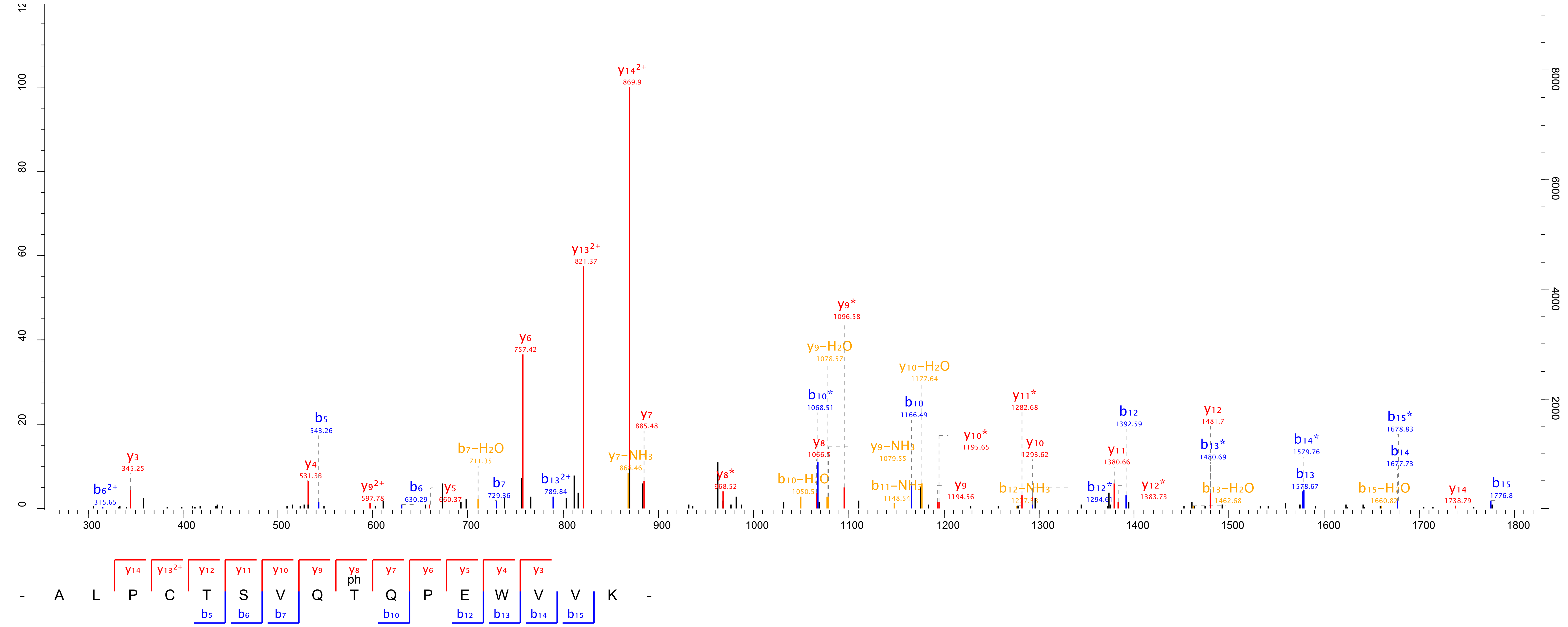

Raw file	Scan	Method	Score	m/z
20130708_PS_Phosphotest_6803wt_YPER02	9727	ITMS; CID	54.77	810.39

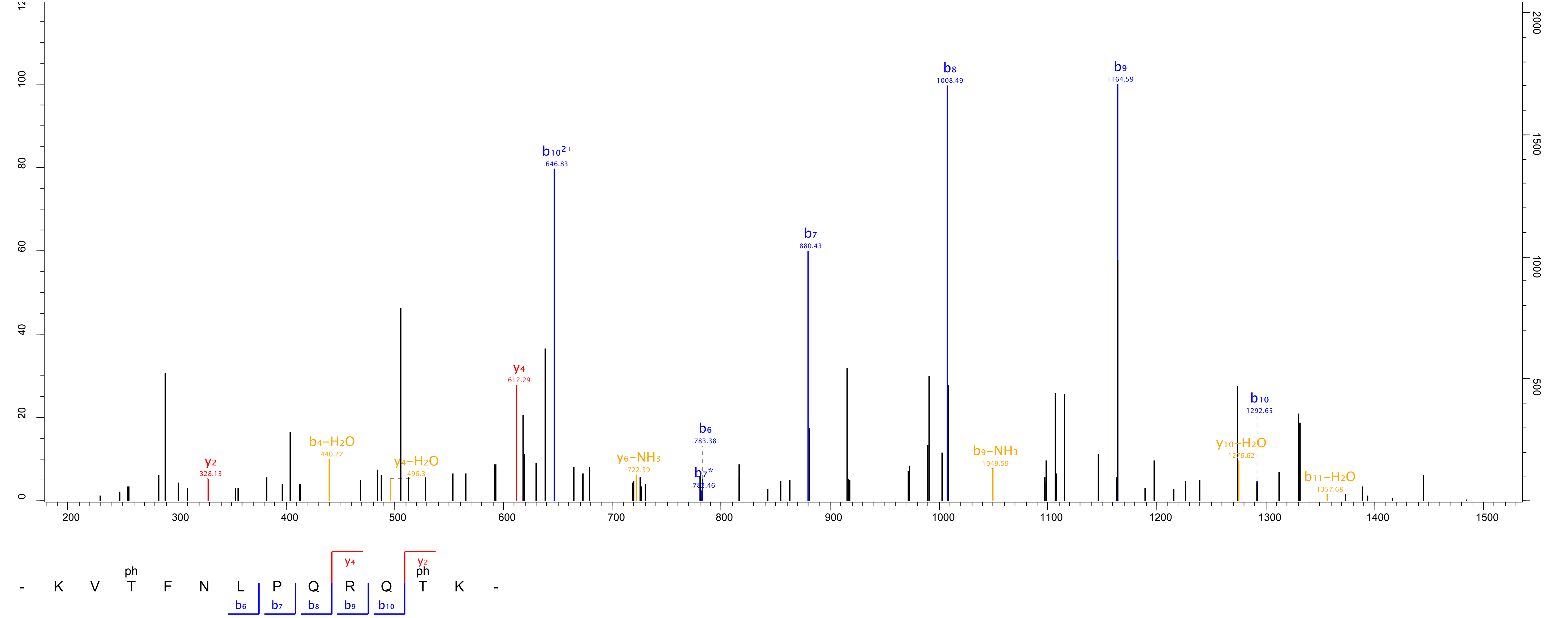

Raw file	Scan	Method	Score	m/z
20130708_PS_Phosphotest_6803wt_SDS01	9803	ITMS; CID	98.9	606.31

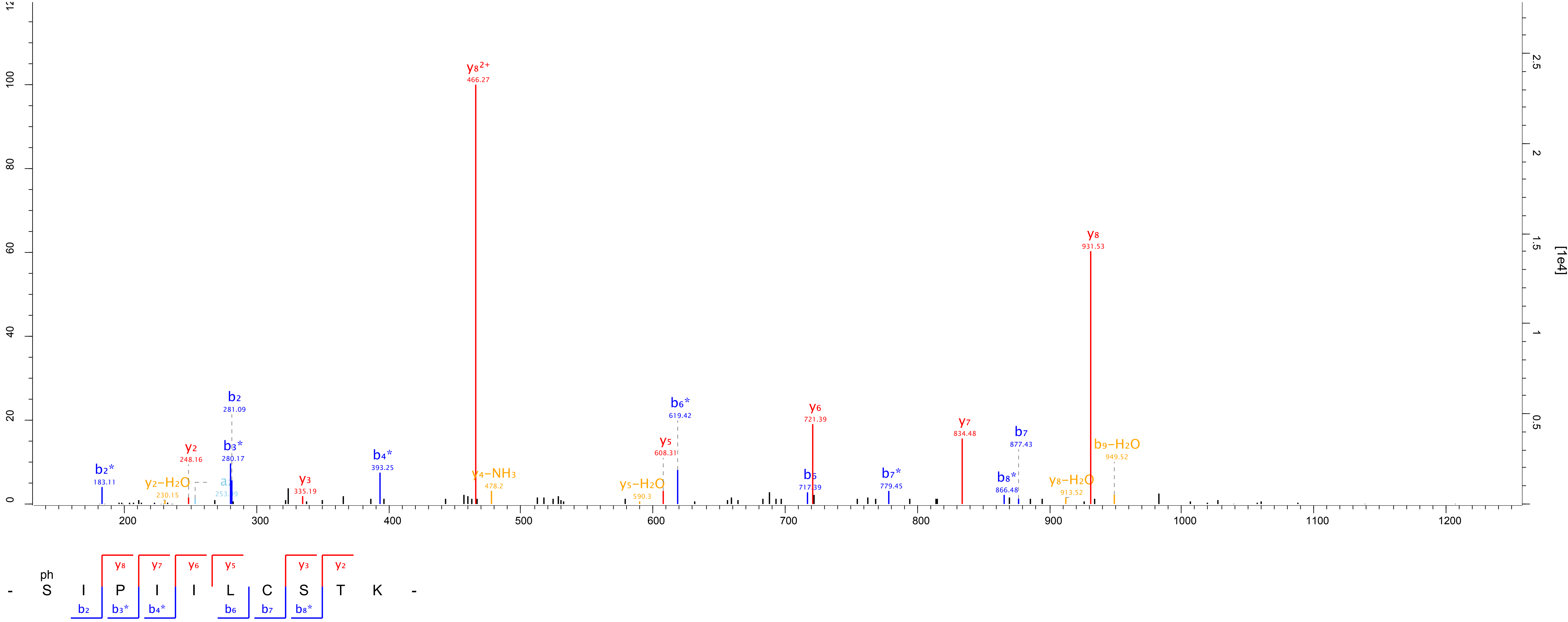

Raw file	Scan	Method	Score	m/z
20130708_PS_Phosphotest_6803wt_SDS02	9864	ITMS; CID	118.5	572.31

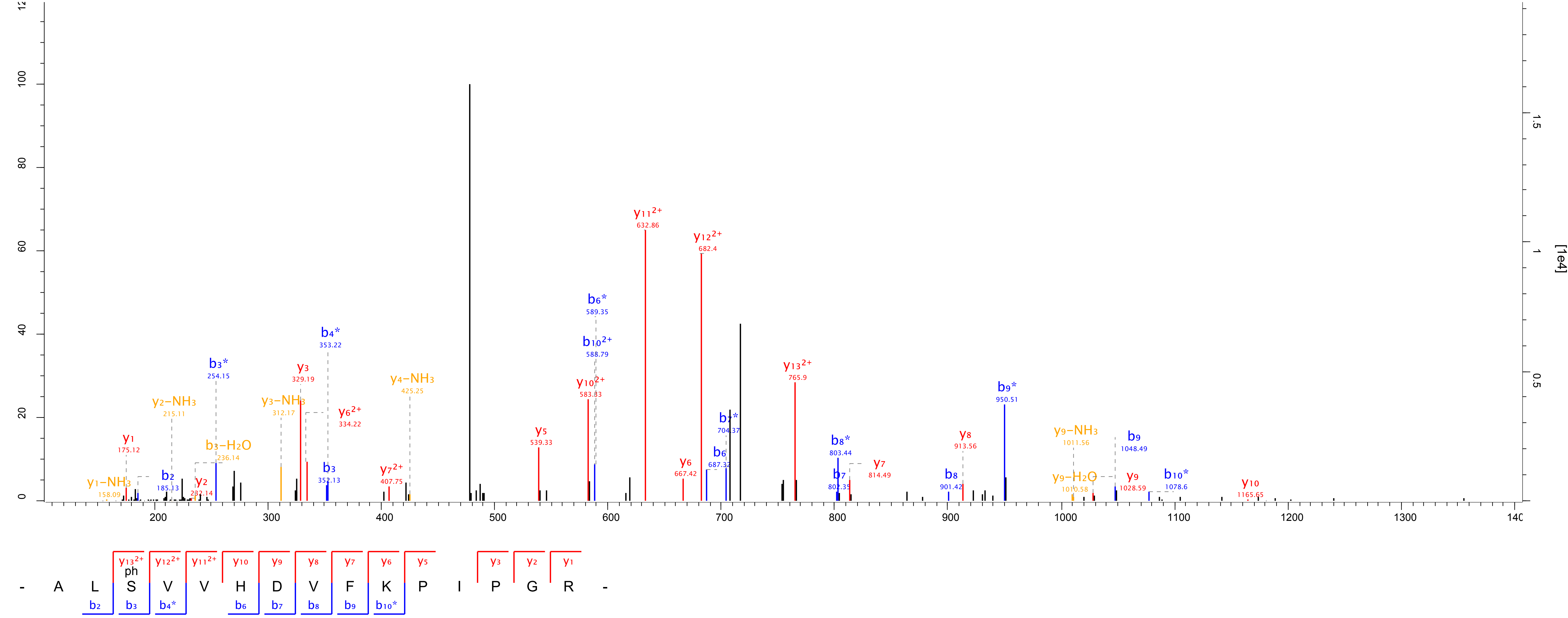

Raw file	Scan	Method	Score	m/z
20130708_PS_Phosphotest_6803wt_SDS01	9882	ITMS; CID	155.88	616.28

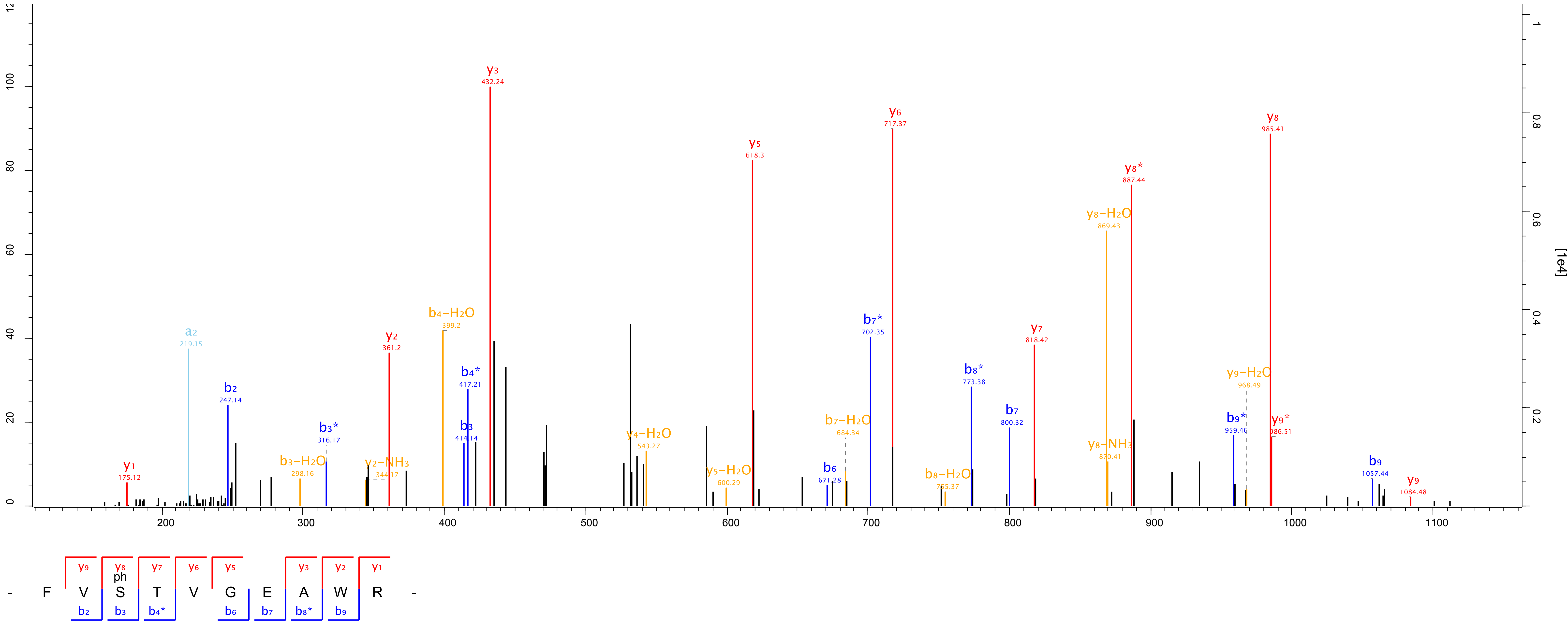

Raw file	Scan	Method	Score	m/z
20130708_PS_Phosphotest_6803wt_YPER01	9912	ITMS; CID	157.38	920.93

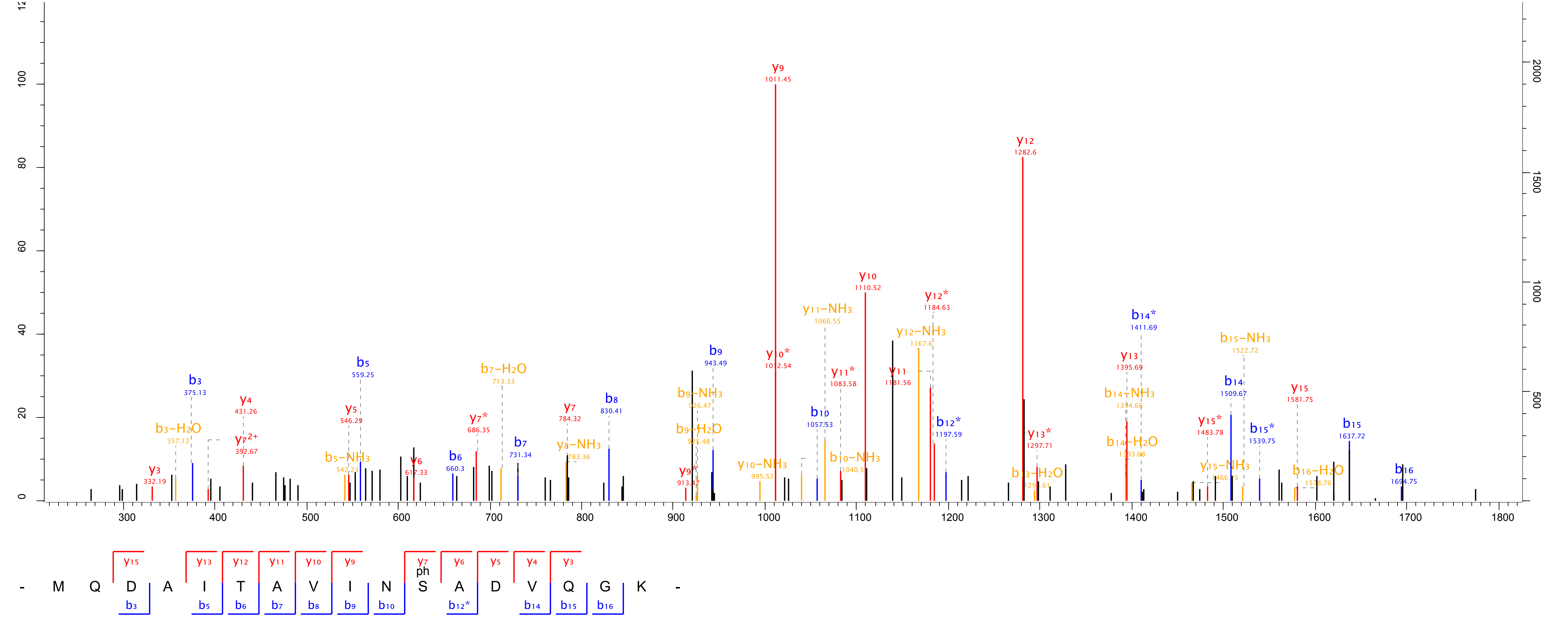

Raw file	Scan	Method	Score	m/z
20130708_PS_Phosphotest_6803wt_SDS01	9942	ITMS; CID	162.49	585.31

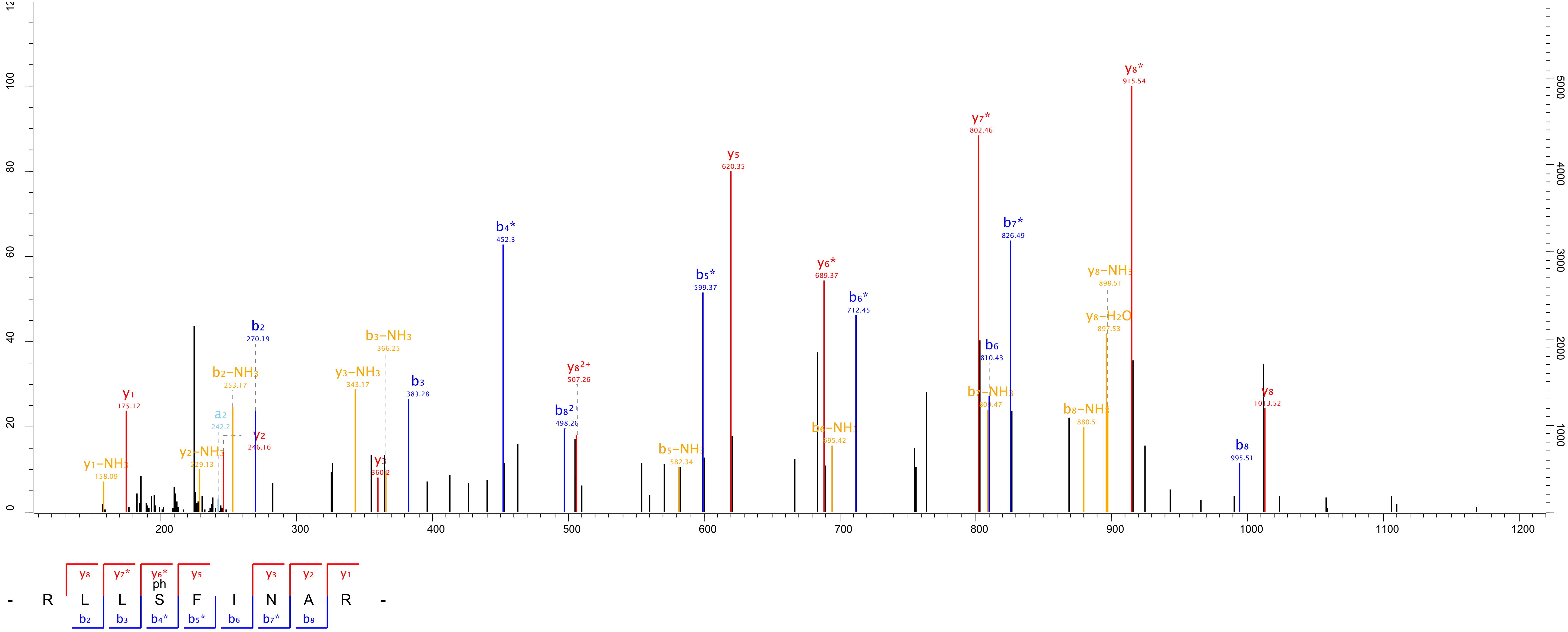

Raw file	Scan	Method	Score	m/z
20130708_PS_Phosphotest_6803wt_SDS01	9987	ITMS; CID	138.89	624.81

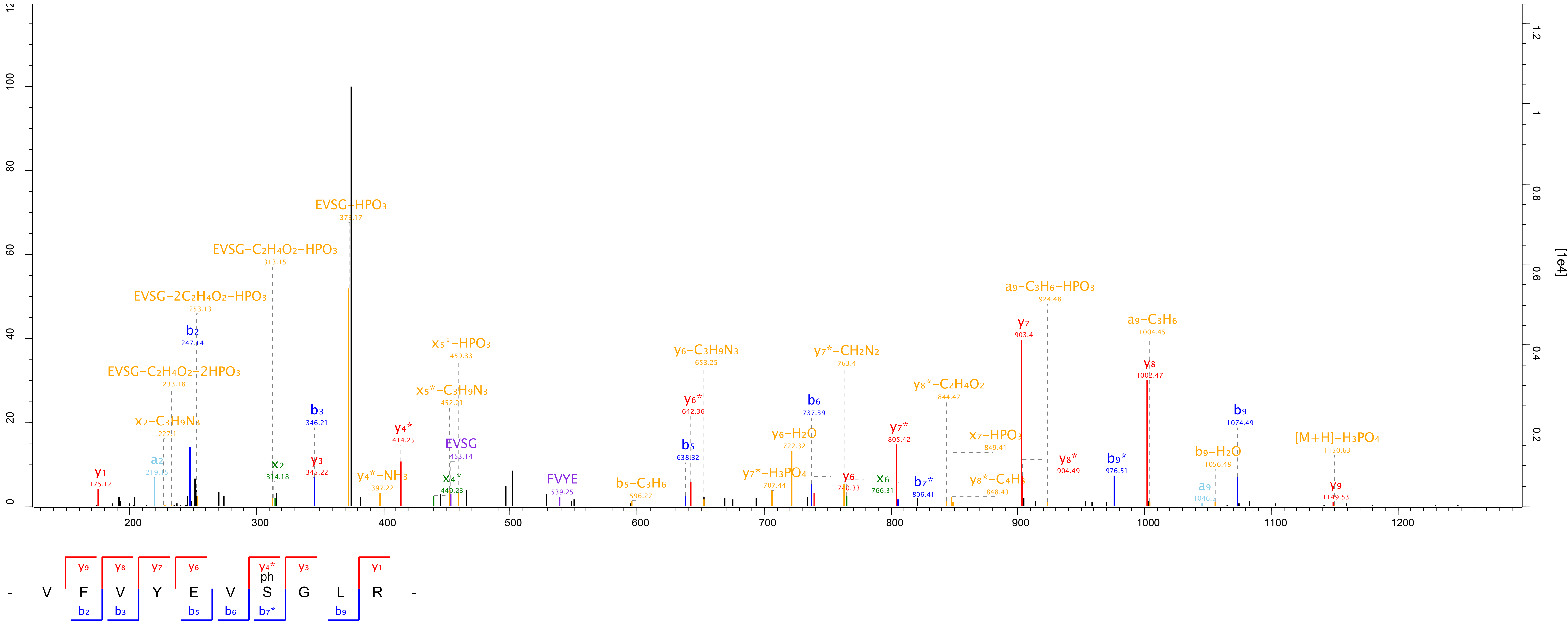

Qualitative Dataset

Phosphopeptide MS/MS Spectra (CID)

Unlocalized phosphorylation events

Raw file	Scan	Method	Score	m/z
20130708_PS_Phosphotest_6803wt_SDS02	10086	ITMS; CID	177.66	780.84

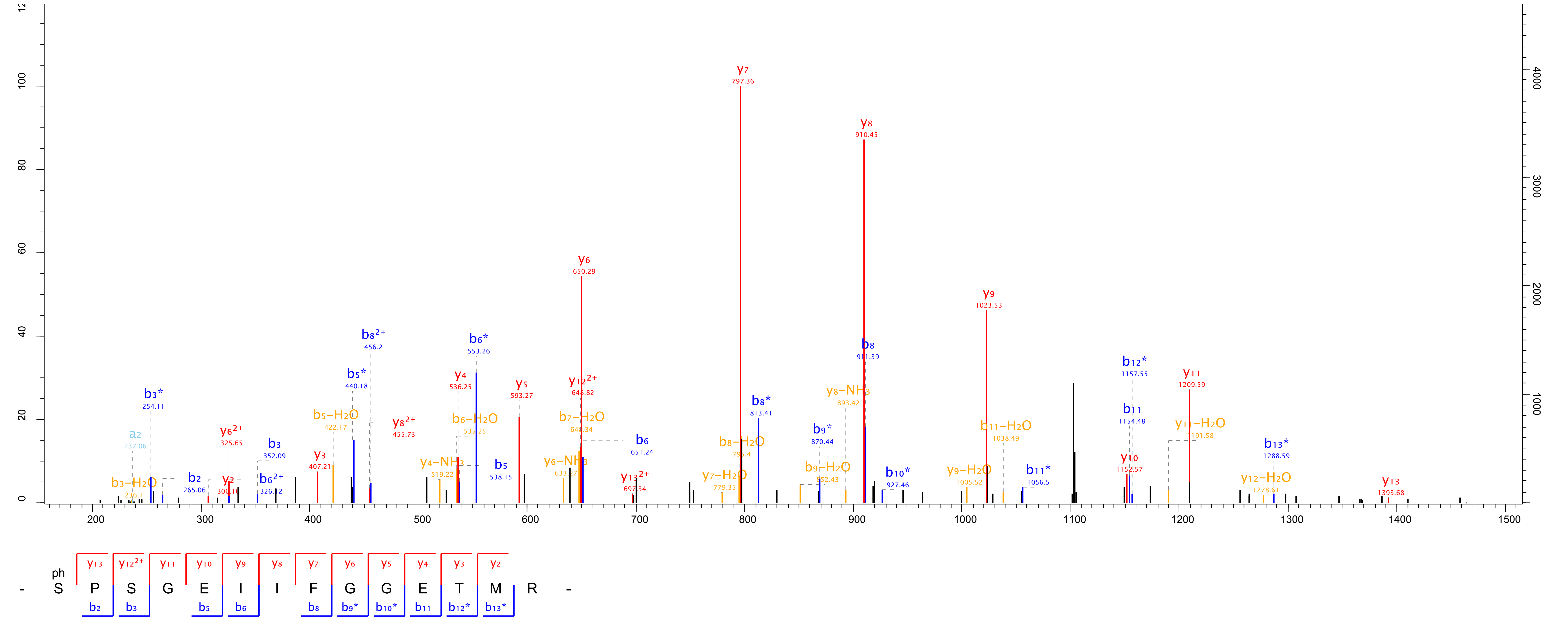

Raw file	Scan	Method	Score	m/z
20130708_PS_Phosphotest_6803wt_YPER01	10250	ITMS; CID	73.92	773.04

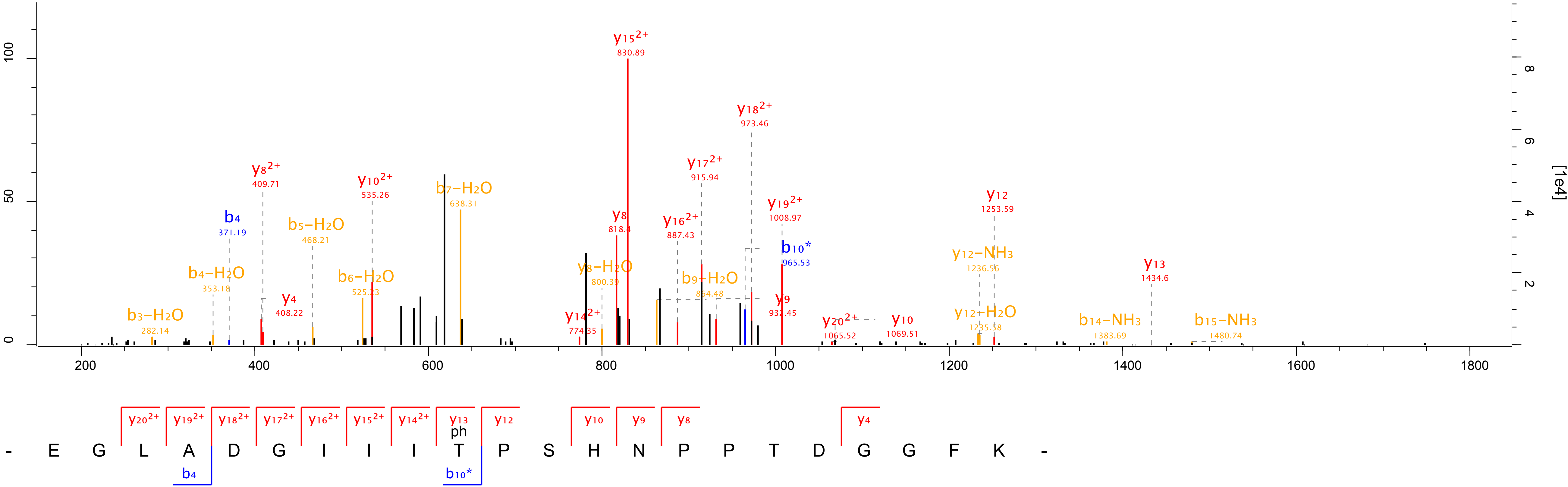

Raw file	Scan	Method	Score	m/z
20130708_PS_Phosphotest_6803wt_SDS02	10253	ITMS; CID	100.02	615.31

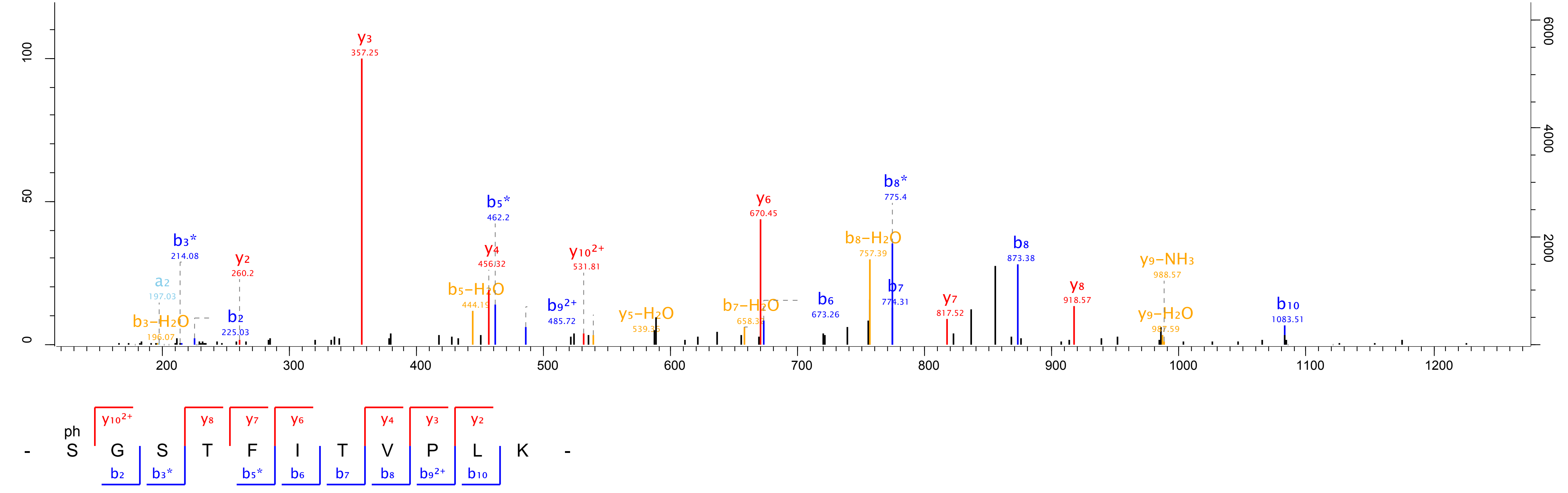

Raw file	Scan	Method	Score	m/z
20130708_PS_Phosphotest_6803wt_SDS01	10513	ITMS; CID	141.86	756.37

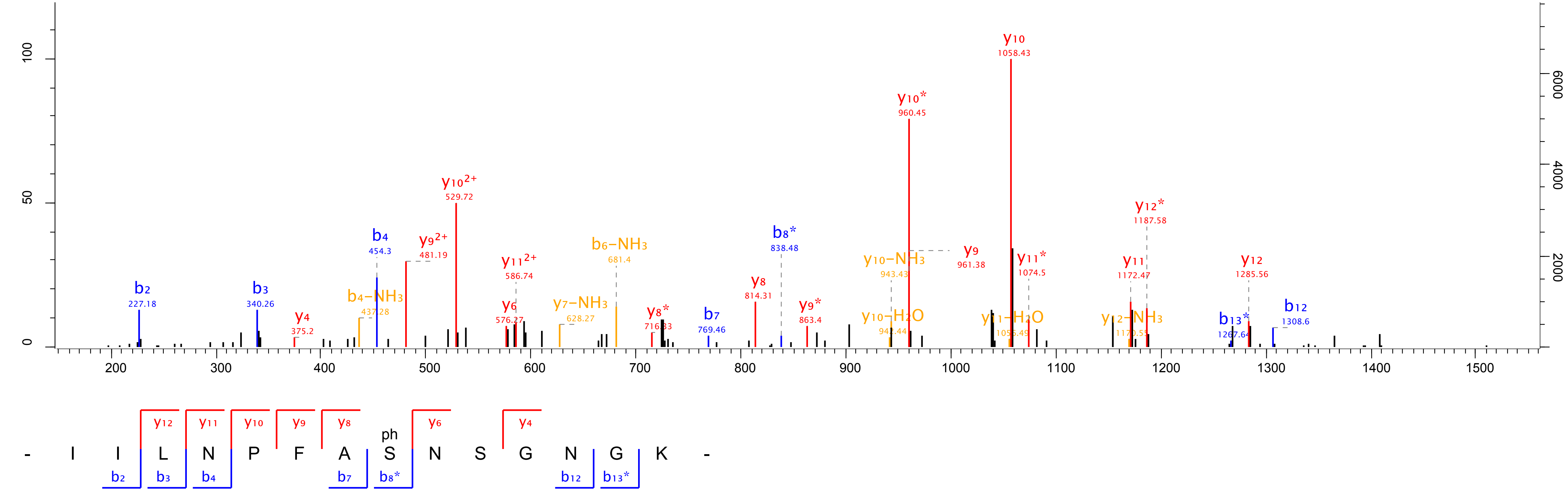

Raw file	Scan	Method	Score	m/z
20130708_PS_Phosphotest_6803wt_SDS01	11072	ITMS; CID	130.07	904.94

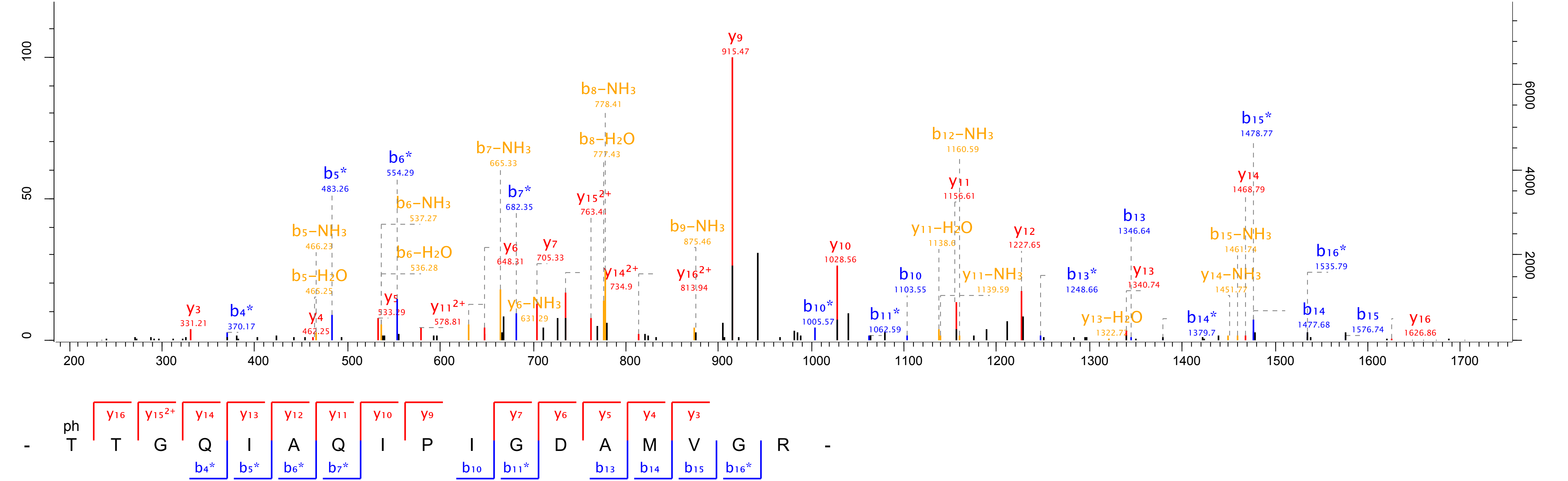

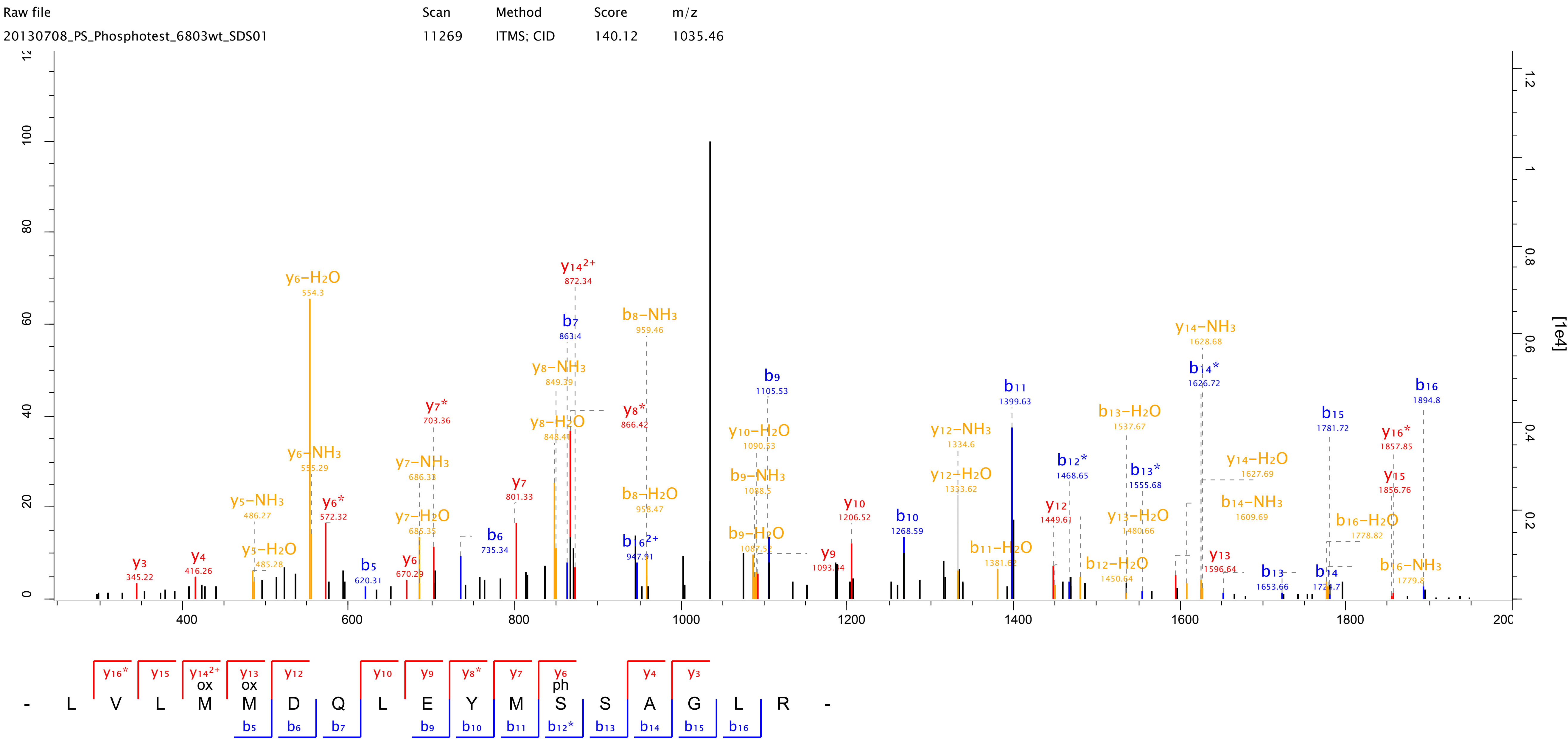
Raw file
20130708_PS_Phosphotest_6803wt_SDS01
Scan
11269
Method
ITMS; CID
Score
140.12
m/z
1035.46
[1e4]
- L V L M M D Q L E Y M S S A G L R -
ox
ox
ph

Raw file	Scan	Method	Score	m/z
20130708_PS_Phosphotest_6803wt_SDS01	11304	ITMS; CID	139.48	667.79

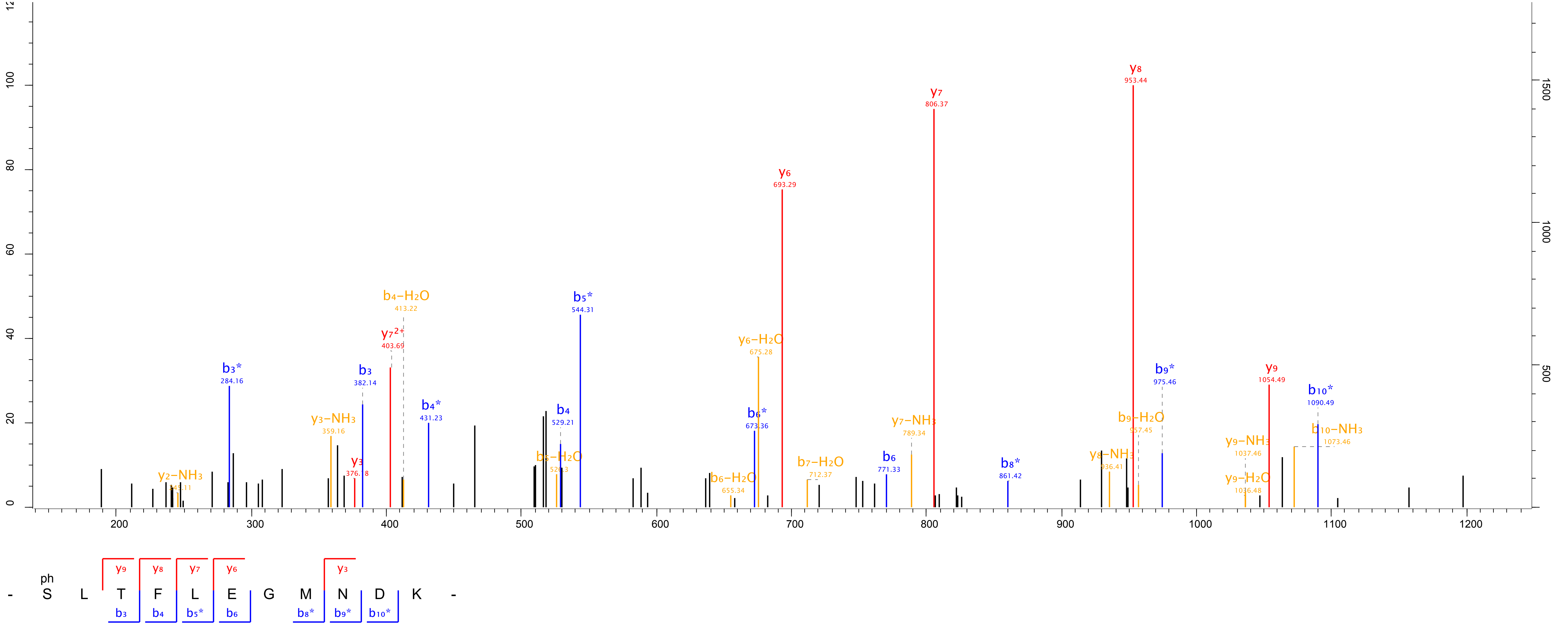

Raw file	Scan	Method	Score	m/z
20130708_PS_Phosphotest_6803wt_SDS01	11652	ITMS; CID	109.85	959.95

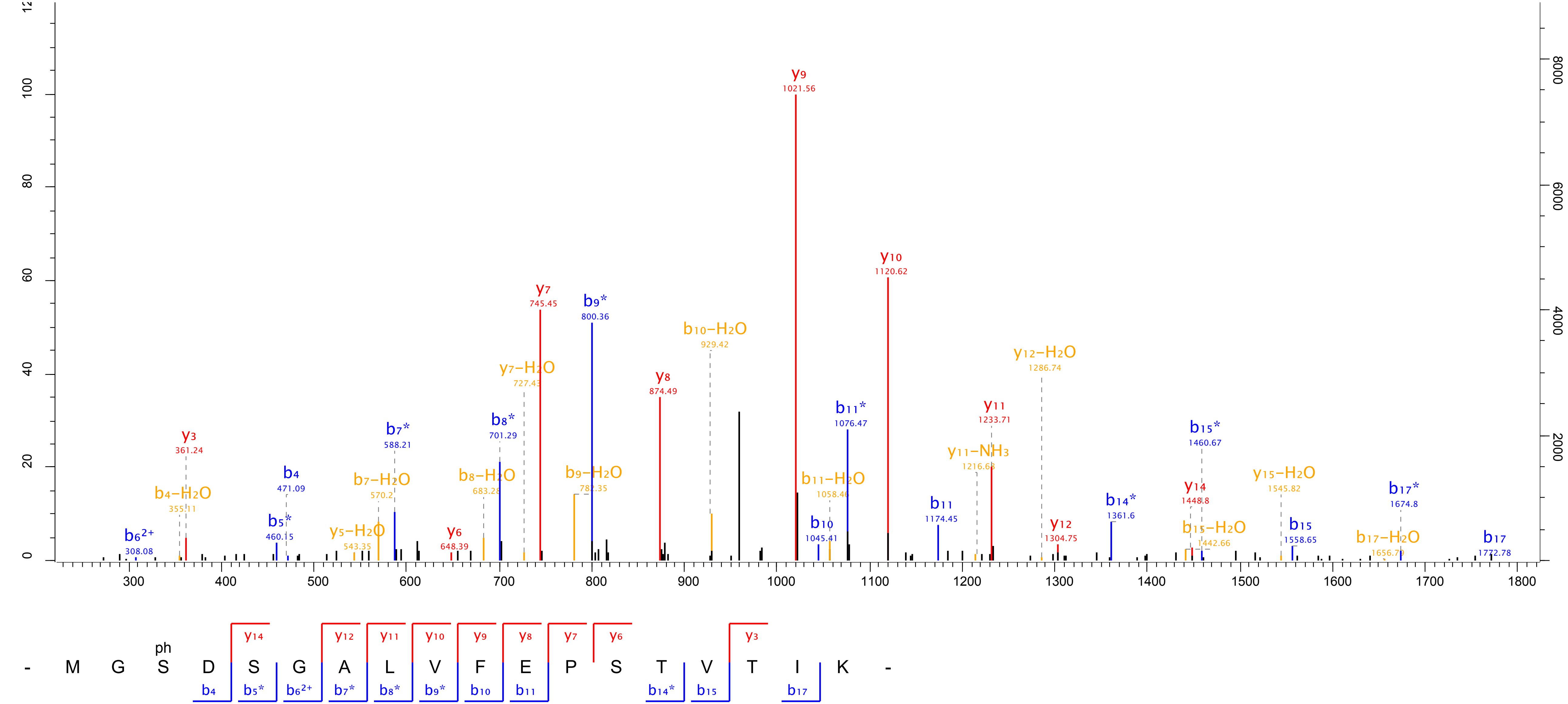

Raw file	Scan	Method	Score	m/z
20130708_PS_Phosphotest_6803wt_SDS01	11827	ITMS; CID	159.18	664.83

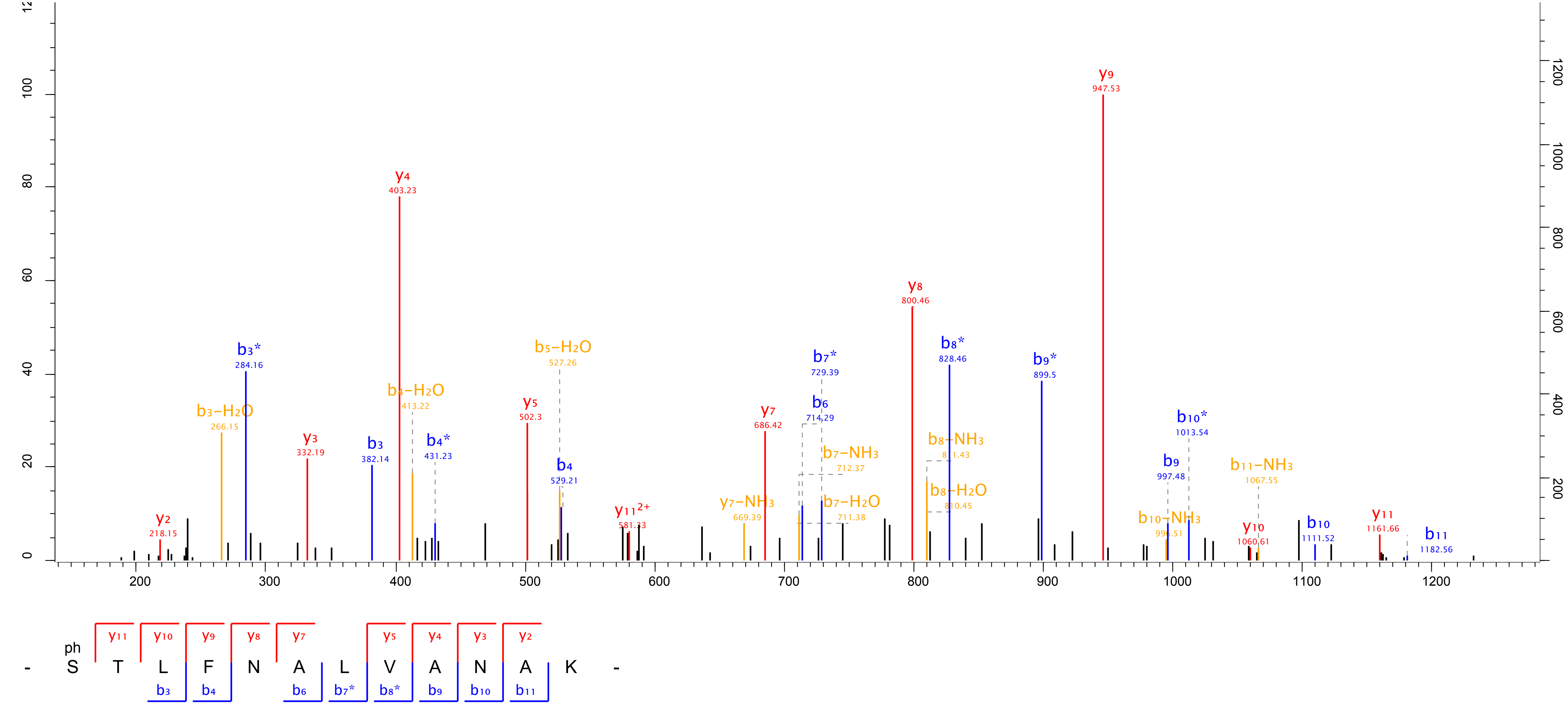

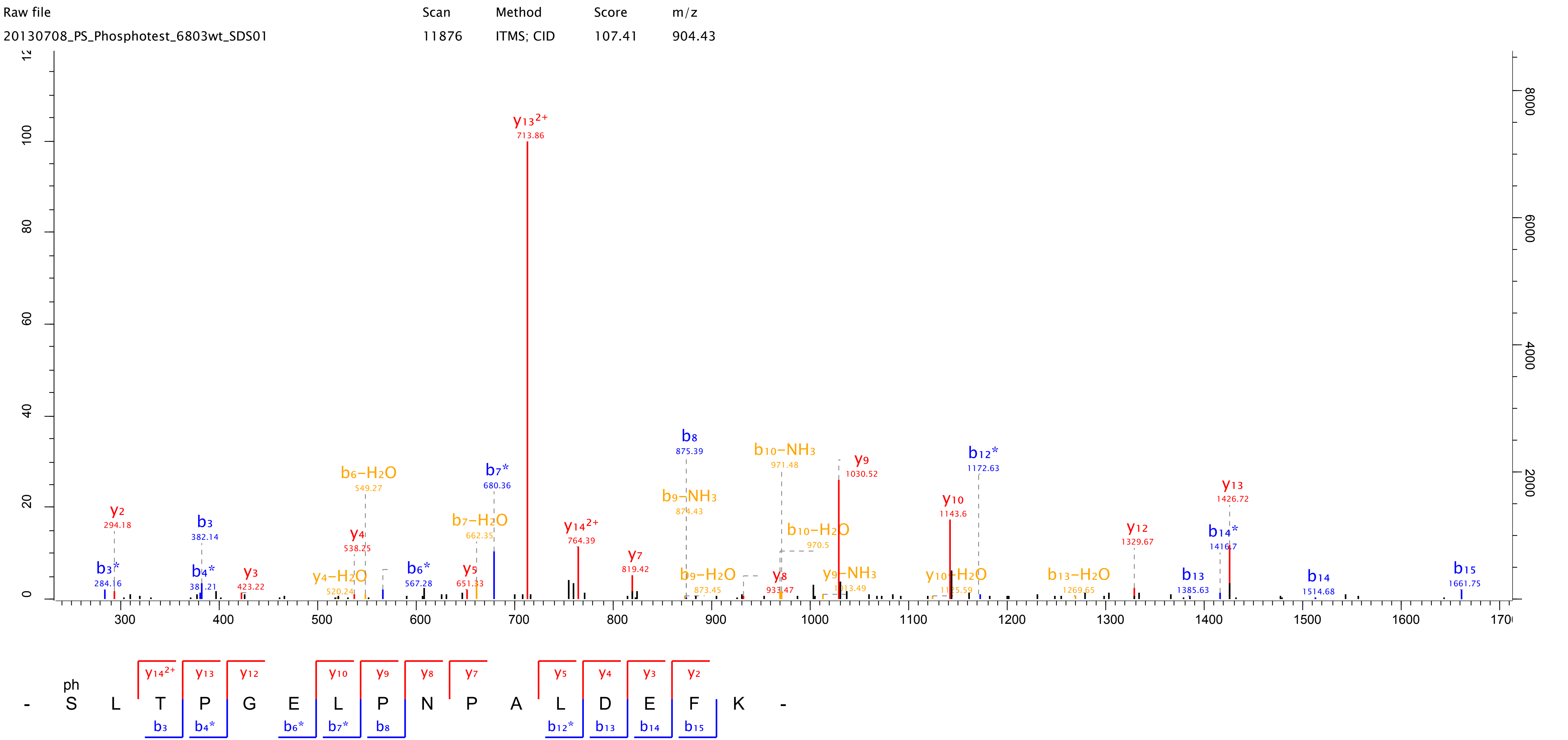

Raw file
20130708_PS_Phosphotest_6803wt_SDS01
Scan
11876
Method
ITMS; CID
Score
107.41
m/z
904.43
y13 2+ 713.86
y2 294.18
b3 382.14
b3* 284.16
b4*
y3 423.22
y4 538.25
y4-H2O 520.24
b6-H2O 549.27
b6* 567.28
y5 651.33
b7-H2O 662.35
b7* 680.36
y14 2+ 764.39
y7 819.42
b8 875.39
b9-NH3
b9-H2O 873.45
y8
b10-NH3 971.48
b10-H2O 970.5
y9 1030.52
y9-NH3
y10 1143.6
y10-H2O
b12* 1172.63
b13-H2O 1269.65
y12 1329.67
b13 1385.63
b14*
y13 1426.72
b14 1514.68
b15 1661.75
- ph S L T P G E L P N P A L D E F K -

Raw file	Scan	Method	Score	m/z
20130708_PS_Phosphotest_6803wt_SDS01	12327	ITMS; CID	85.09	894.92

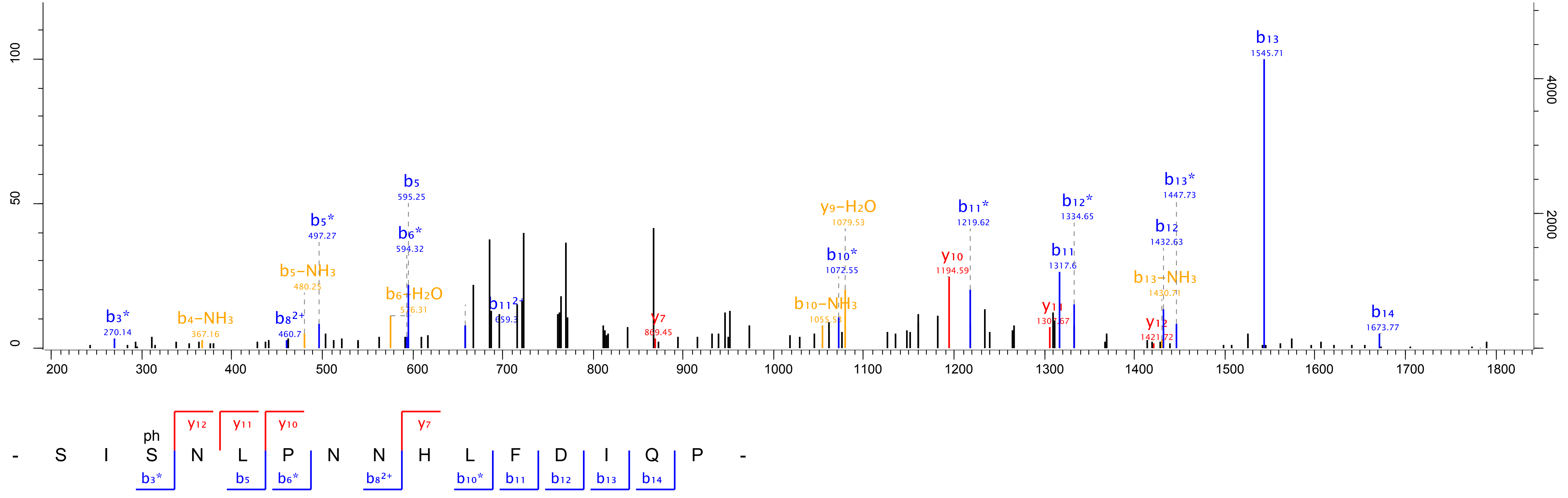

Raw file	Scan	Method	Score	m/z
20130708_PS_Phosphotest_6803wt_SDS01	13093	ITMS; CID	54.01	753.38

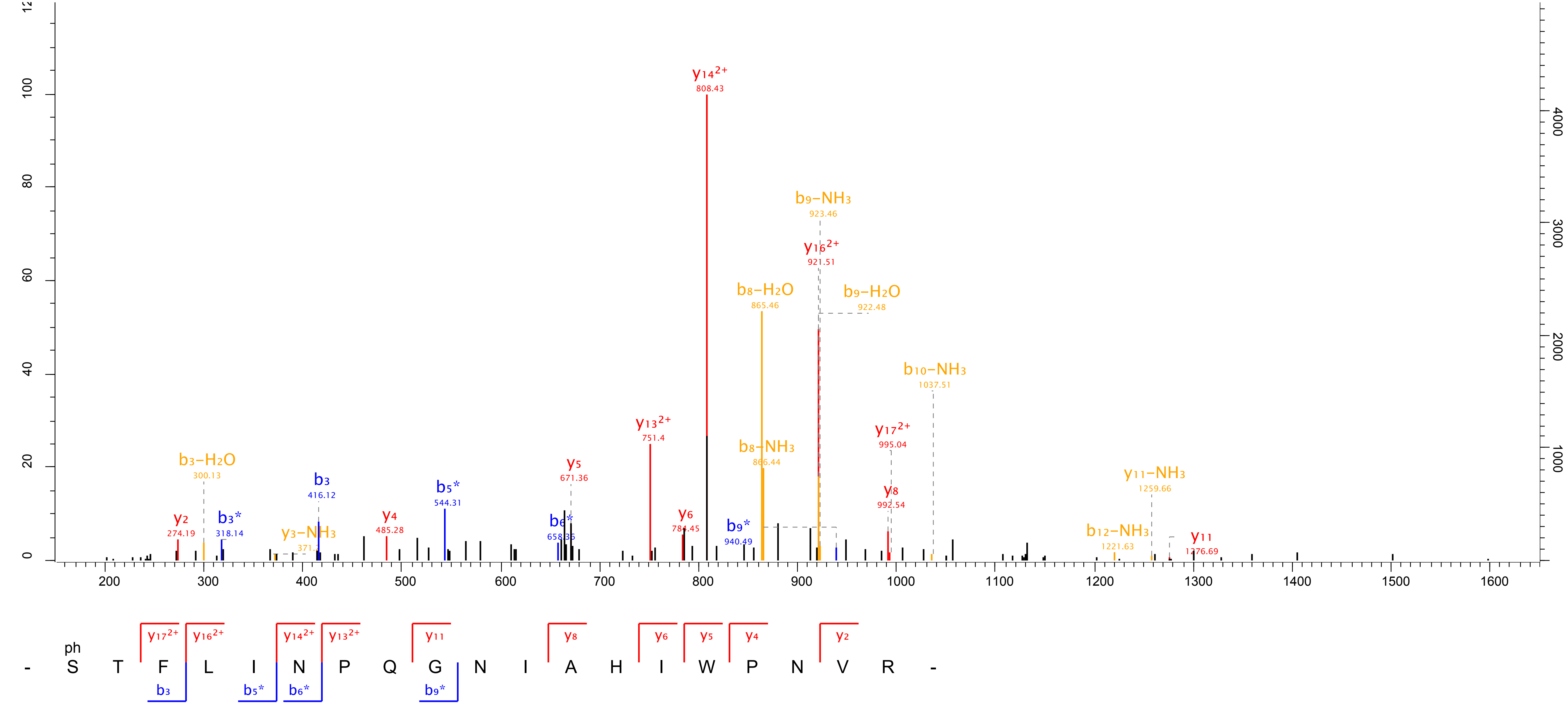

Raw file	Scan	Method	Score	m/z
20130708_PS_Phosphotest_6803wt_SDS01	13554	ITMS; CID	179.66	774.88

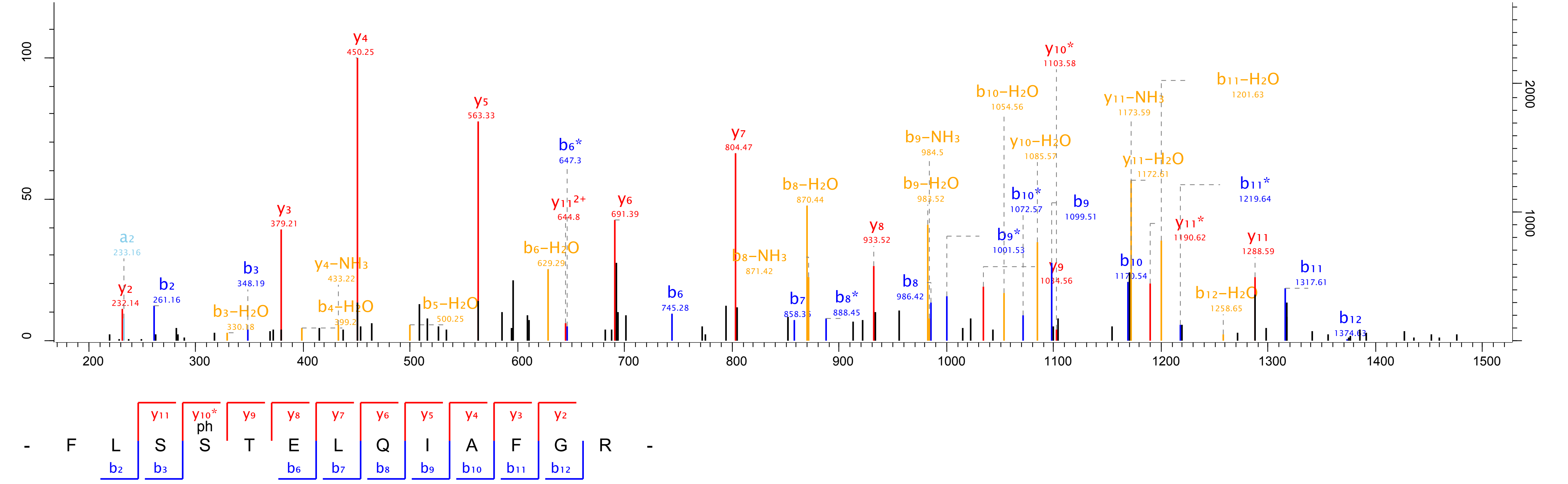

Raw file	Scan	Method	Score	m/z
20130708_PS_Phosphotest_6803wt_SDS01	13733	ITMS; CID	62.78	765.4

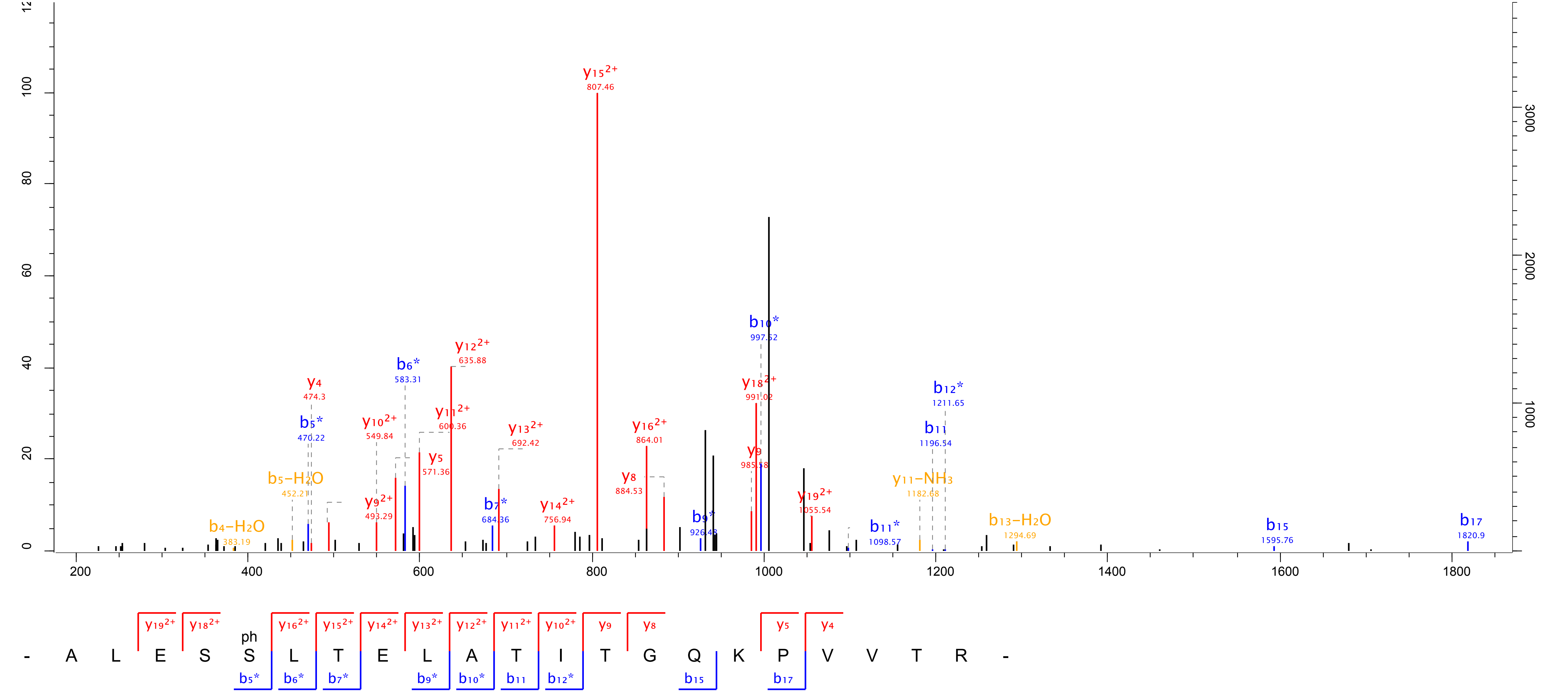

Raw file	Scan	Method	Score	m/z
20130708_PS_Phosphotest_6803wt_SDS01	14006	ITMS; CID	84.95	1150.58

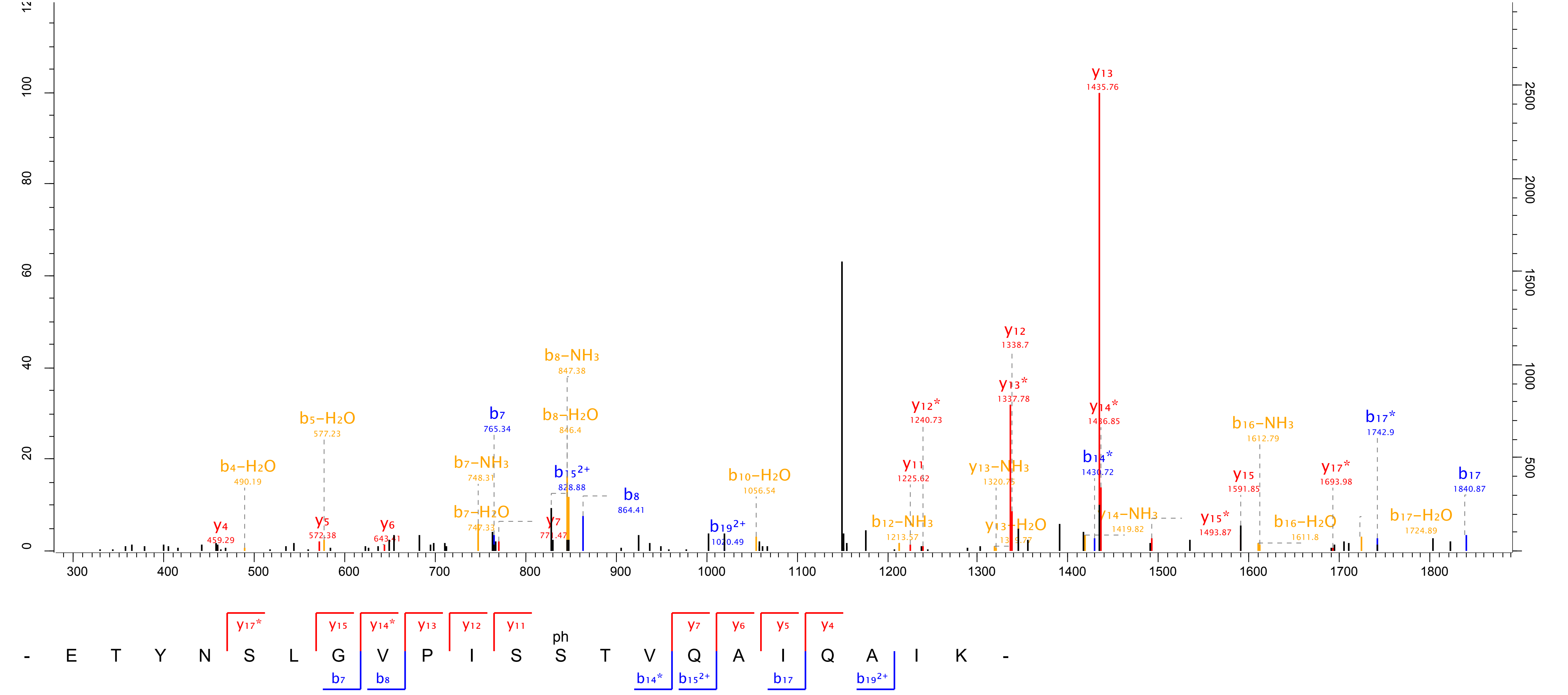

Raw file	Scan	Method	Score	m/z
20130708_PS_Phosphotest_6803wt_SDS02	2430	ITMS; CID	119.34	597.25

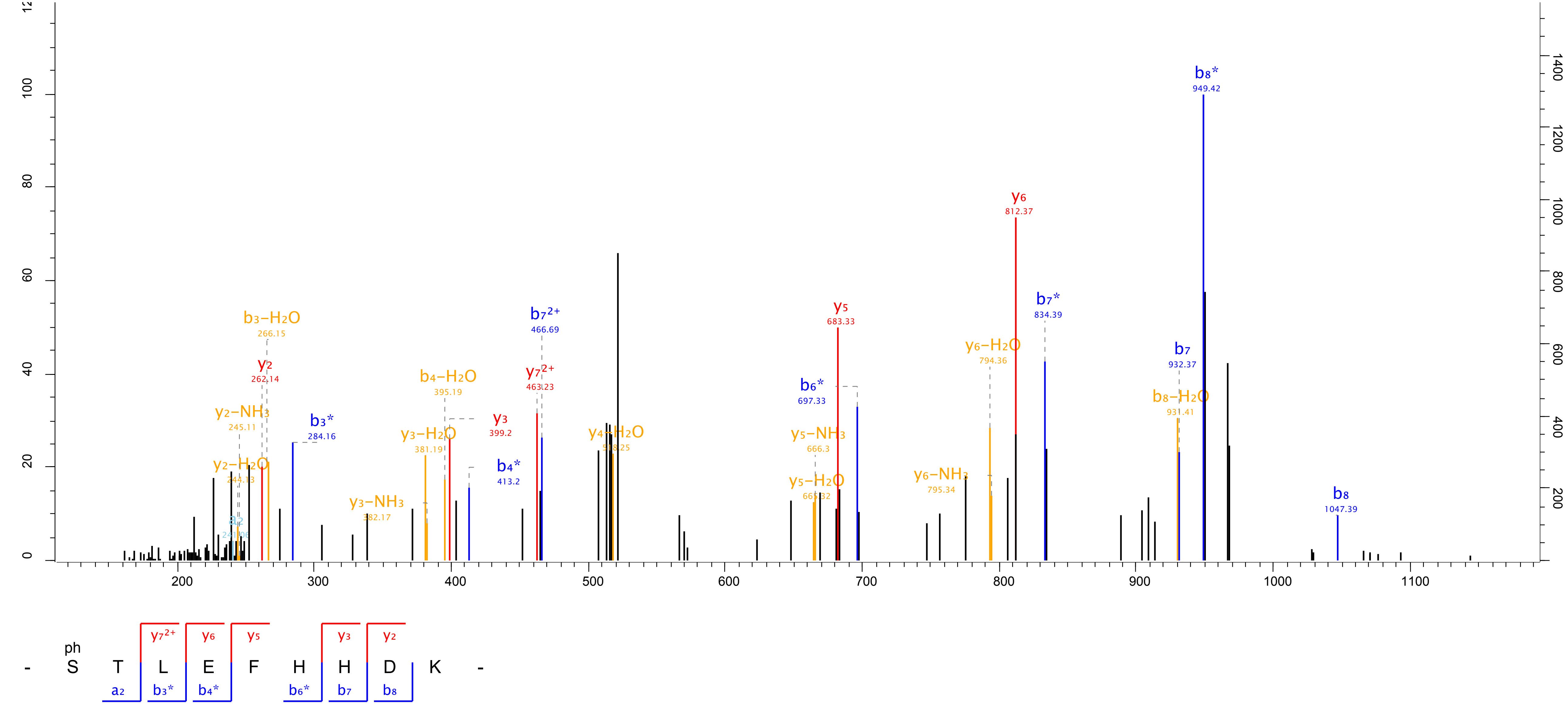

Raw file	Scan	Method	Score	m/z
20130708_PS_Phosphotest_6803wt_SDS01	3954	ITMS; CID	83.08	485.22

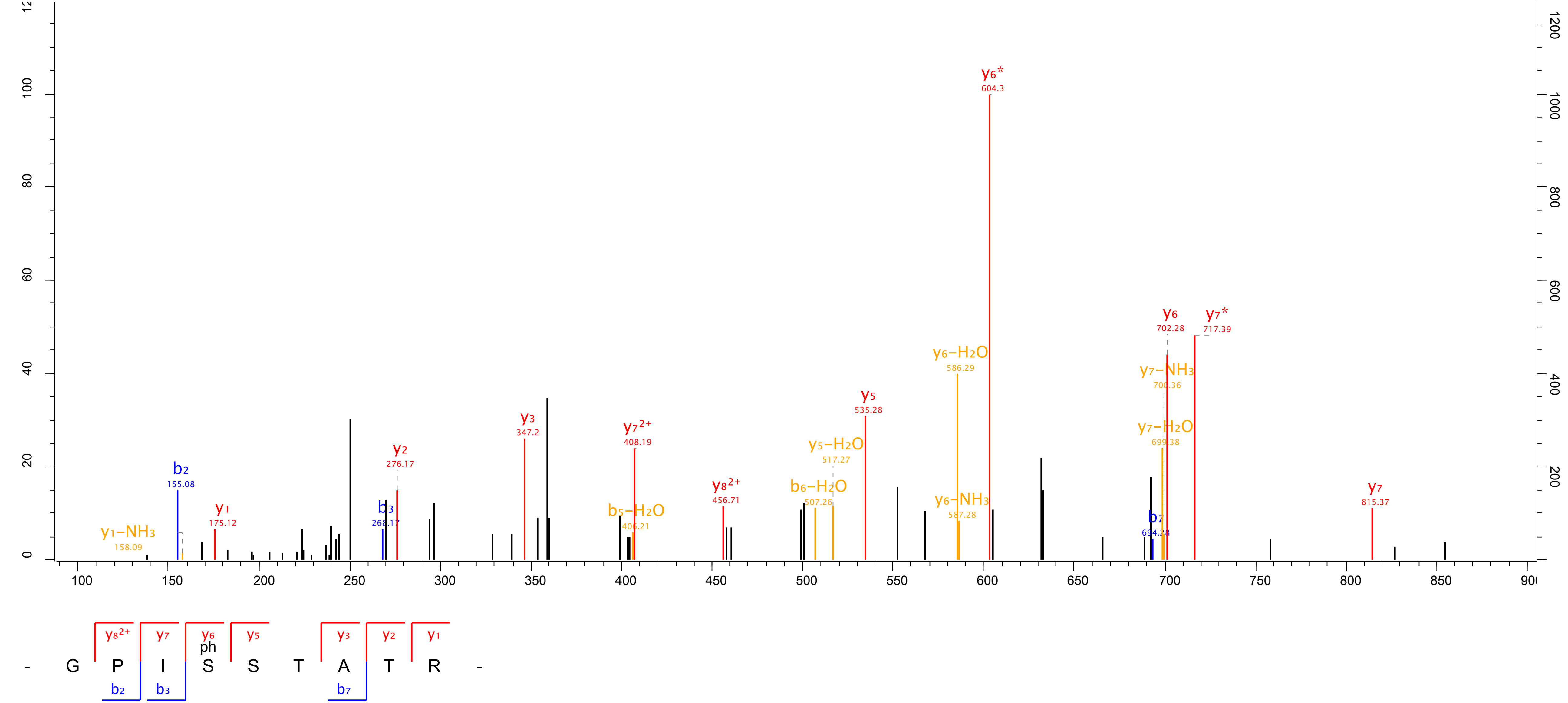

Raw file	Scan	Method	Score	m/z
20130708_PS_Phosphotest_6803wt_SDS01	4362	ITMS; CID	62.29	515.25

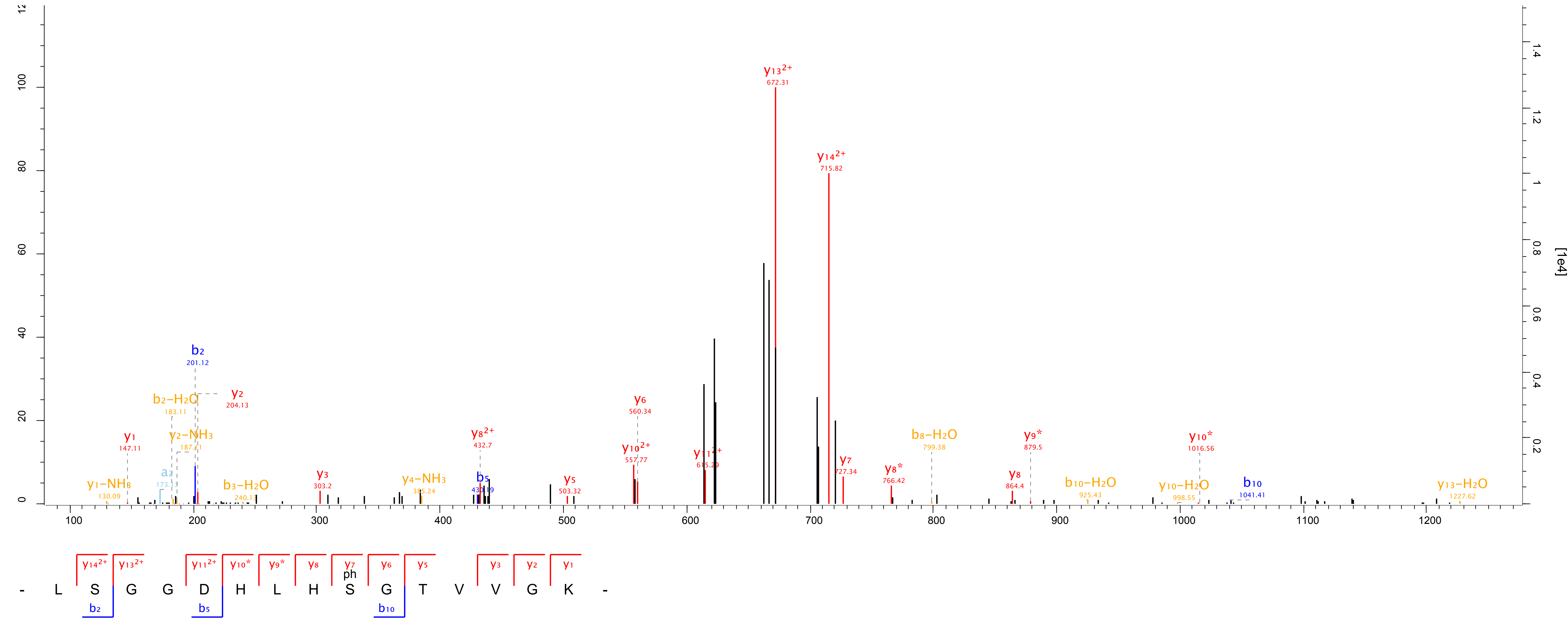

Raw file	Scan	Method	Score	m/z
20130708_PS_Phosphotest_6803wt_SDS01	4400	ITMS; CID	119.03	530.23

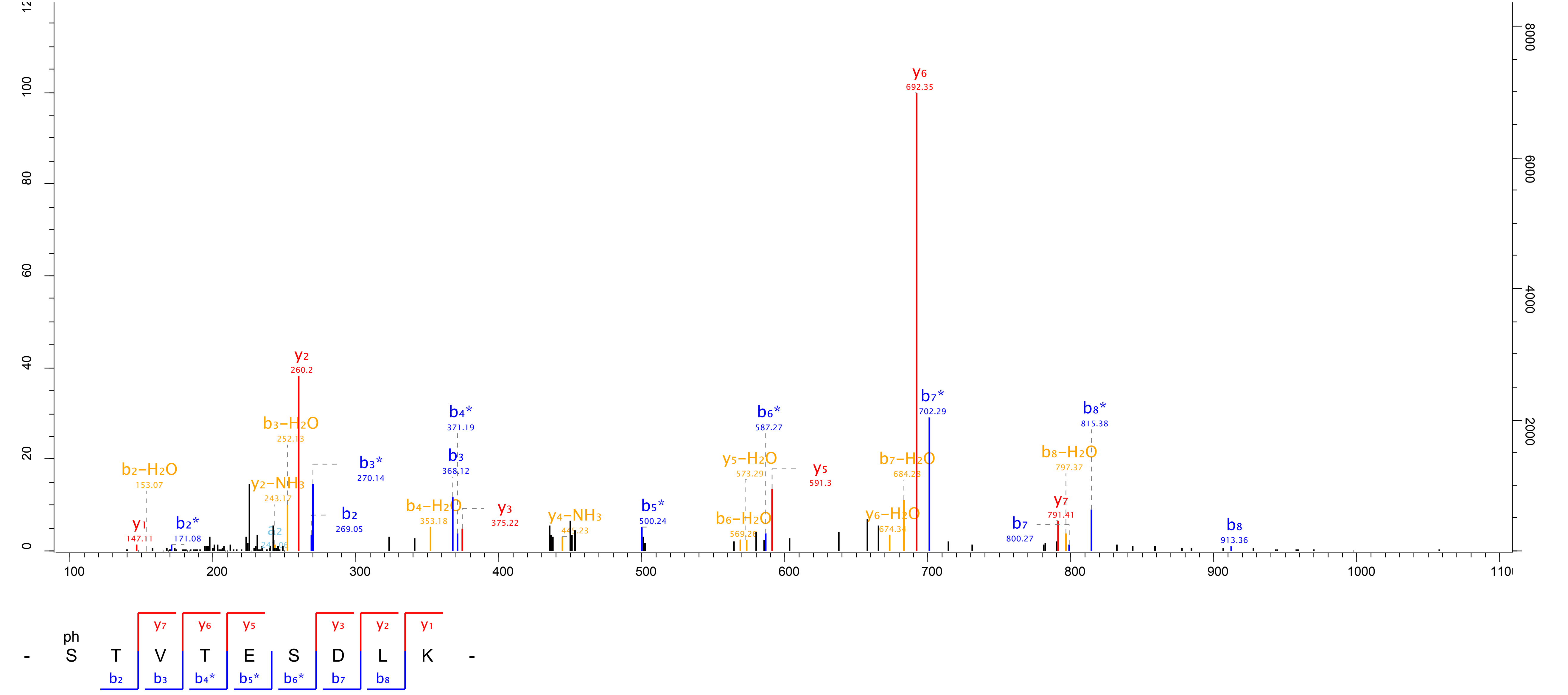

Raw file	Scan	Method	Score	m/z
20130708_PS_Phosphotest_6803wt_YPER01	4982	ITMS; CID	92.94	545.25

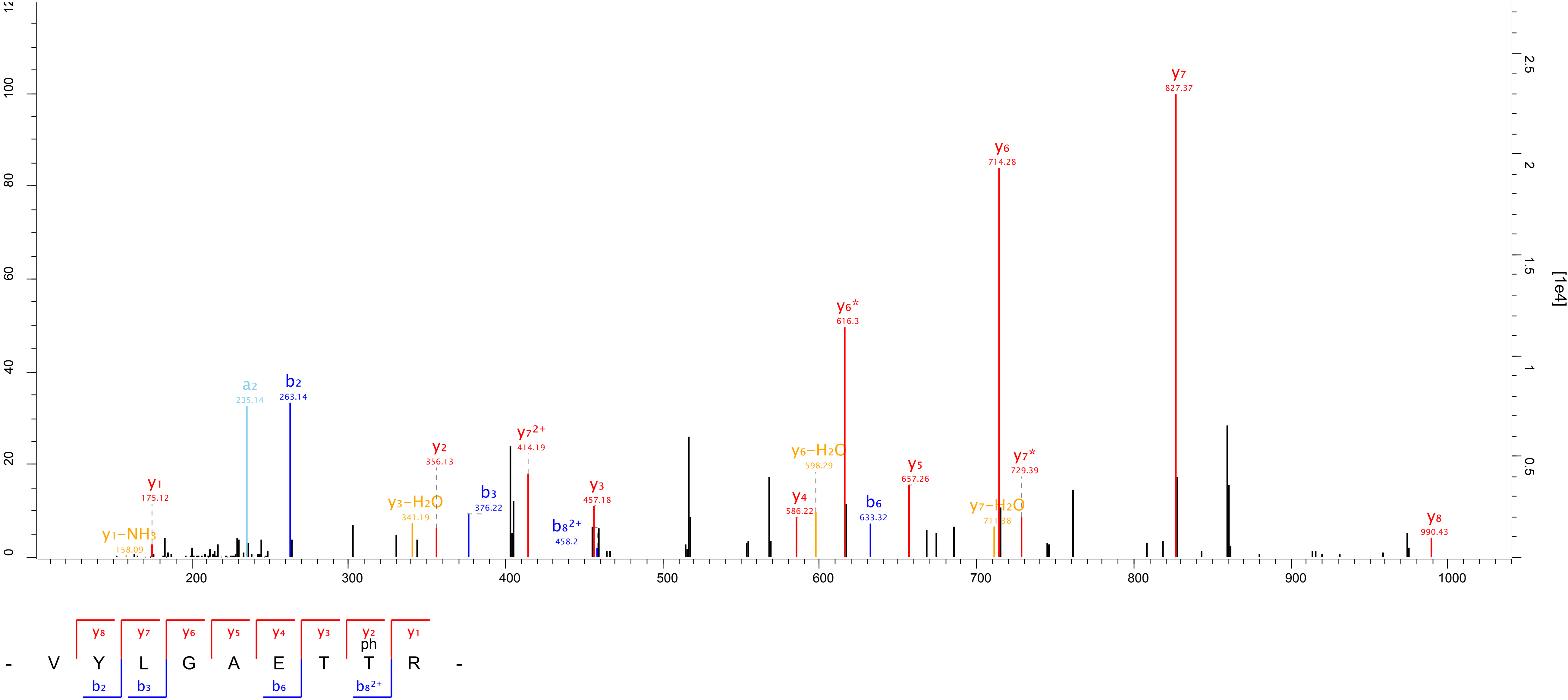

Raw file	Scan	Method	Score	m/z
20130708_PS_Phosphotest_6803wt_SDS01	5082	ITMS; CID	93.67	456.22

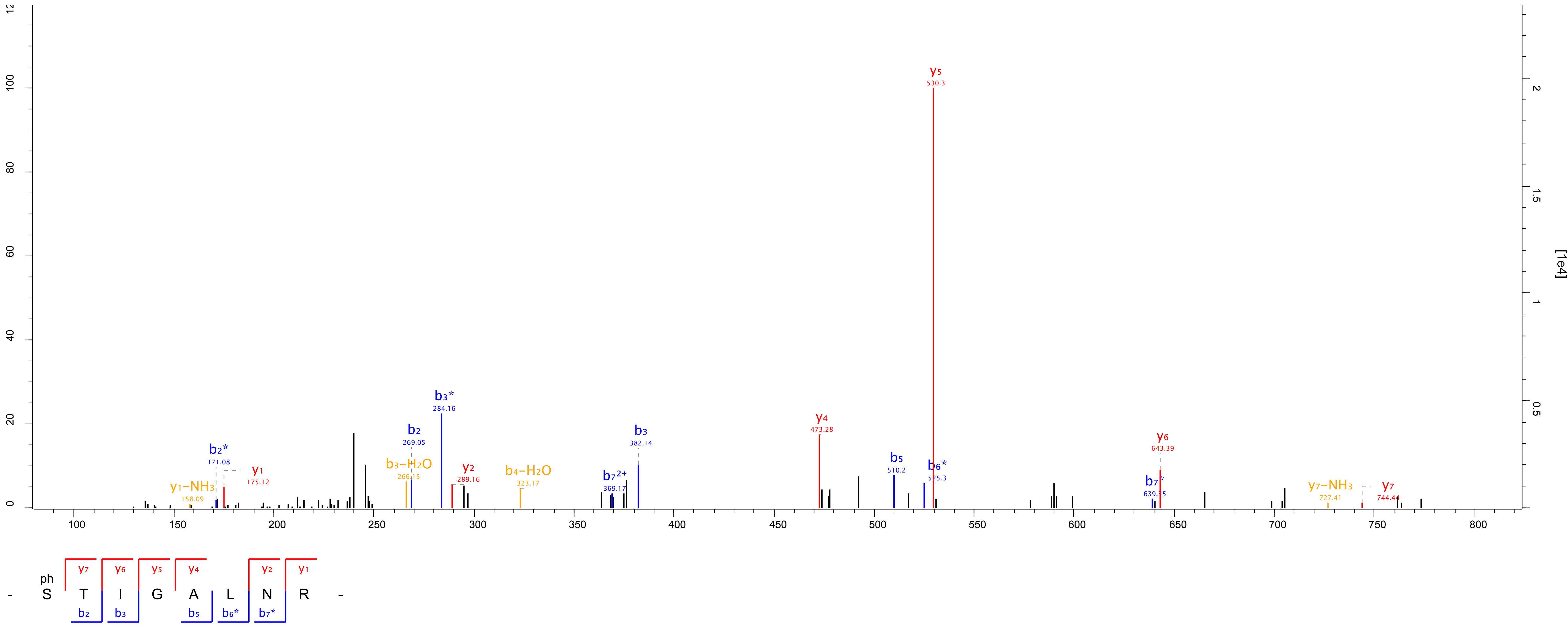

Raw file	Scan	Method	Score	m/z
20130708_PS_Phosphotest_6803wt_YPER01	5445	ITMS; CID	192.23	823.34

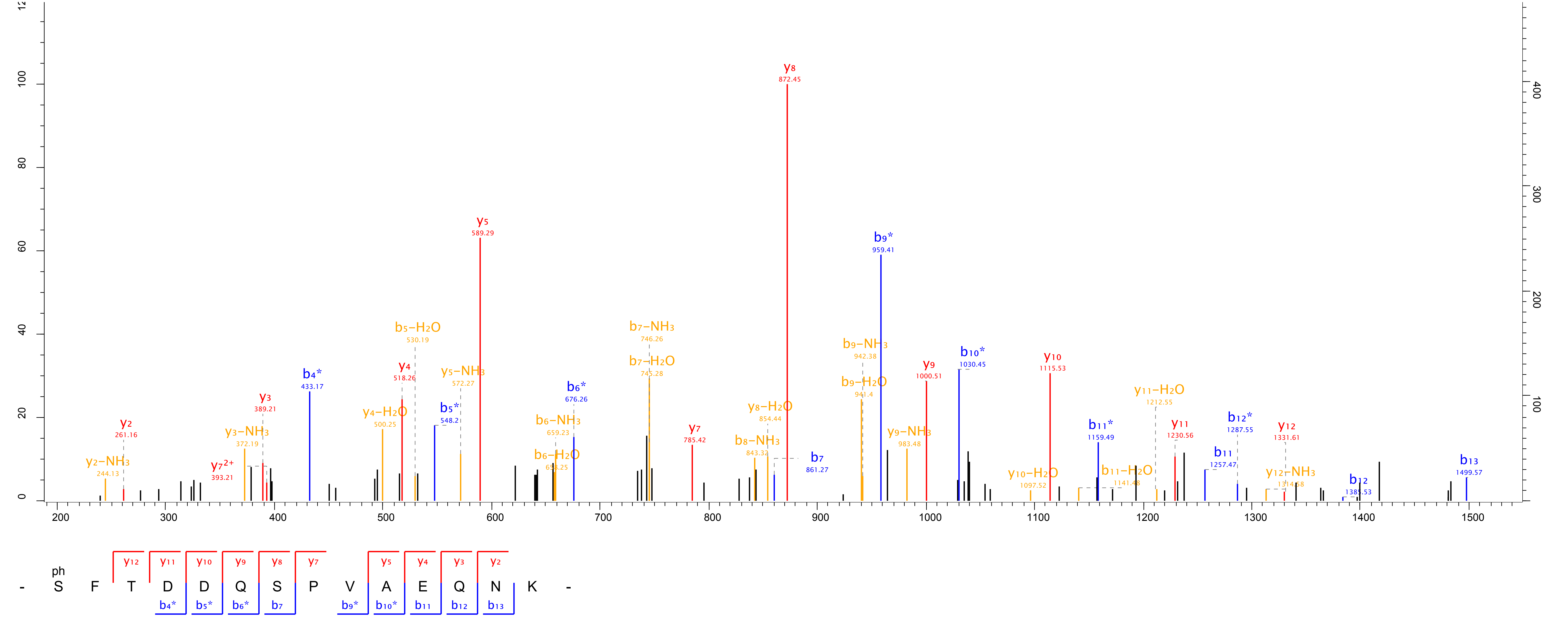

Raw file	Scan	Method	Score	m/z
20130708_PS_Phosphotest_6803wt_SDS01	5587	ITMS; CID	85.86	413.22

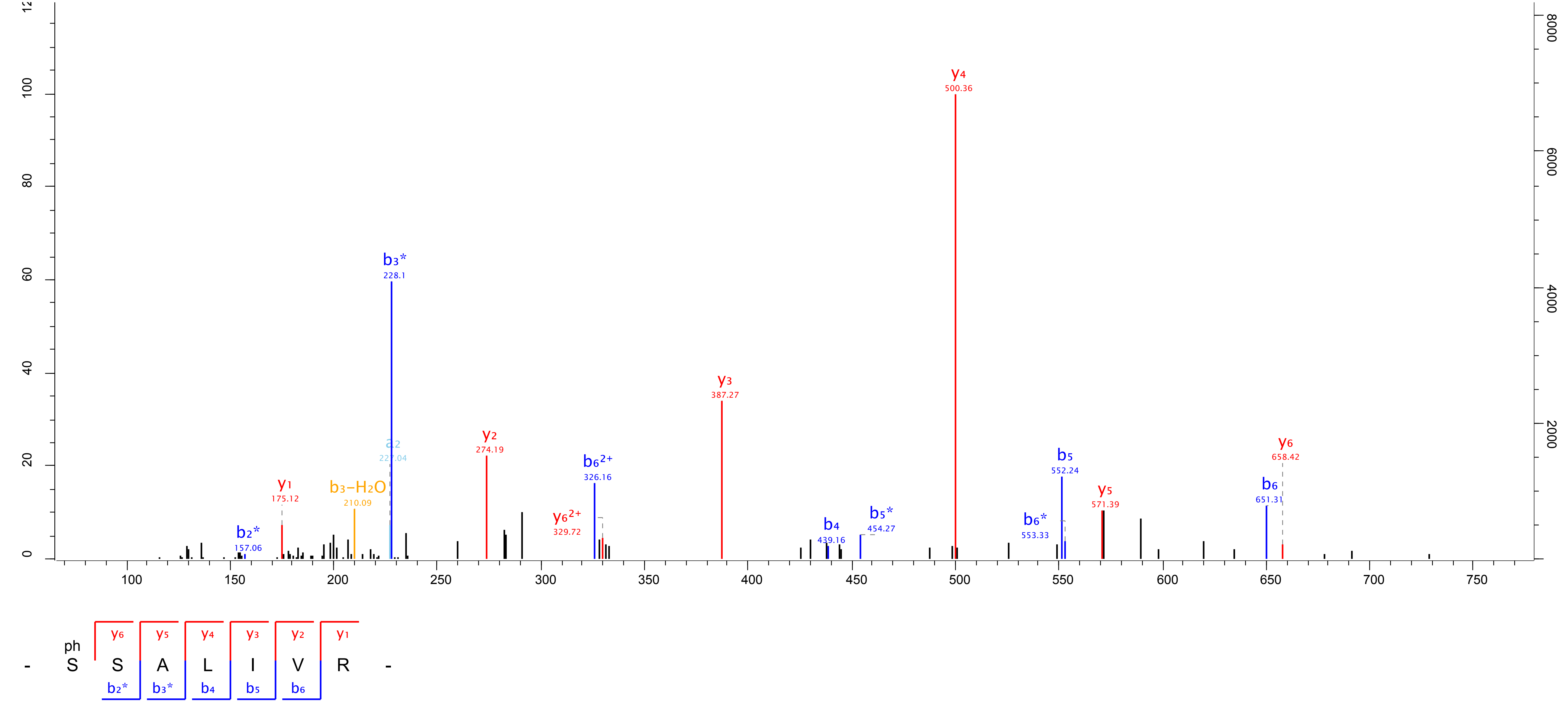

Raw file	Scan	Method	Score	m/z
20130708_PS_Phosphotest_6803wt_SDS02	5742	ITMS; CID	165.88	707.67

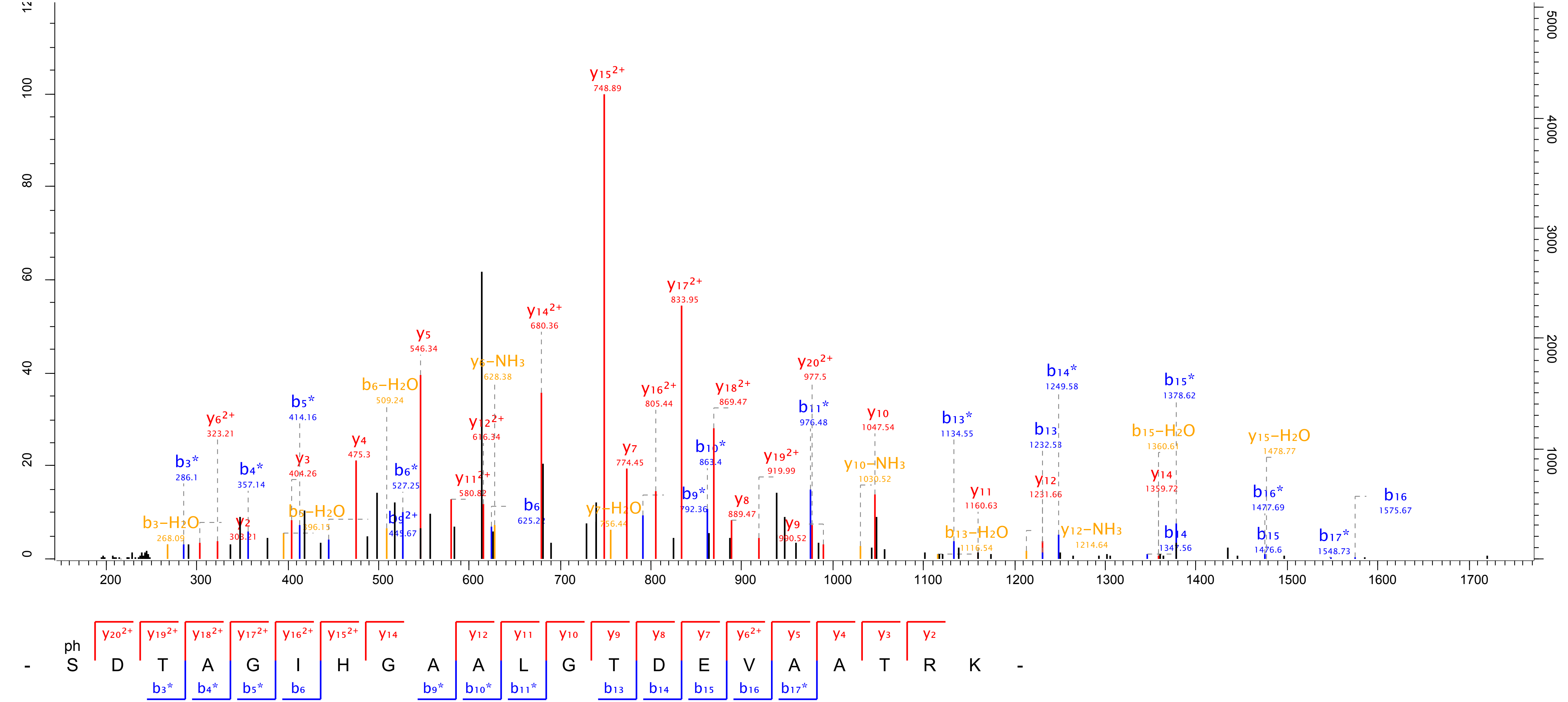

Raw file	Scan	Method	Score	m/z
20130708_PS_Phosphotest_6803wt_SDS01	6345	ITMS; CID	133.48	673.76

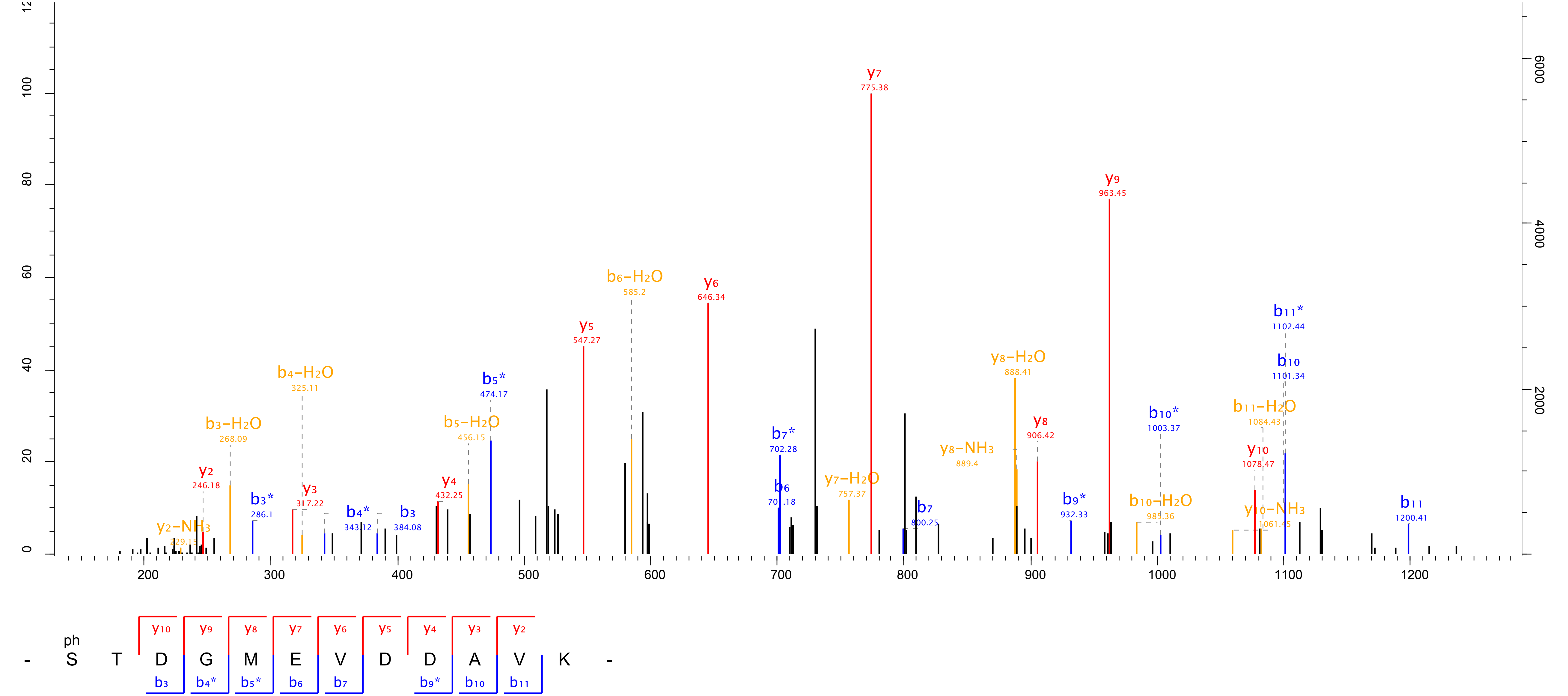

Raw file	Scan	Method	Score	m/z
20130708_PS_Phosphotest_6803wt_YPER01	6387	ITMS; CID	115.26	649.31

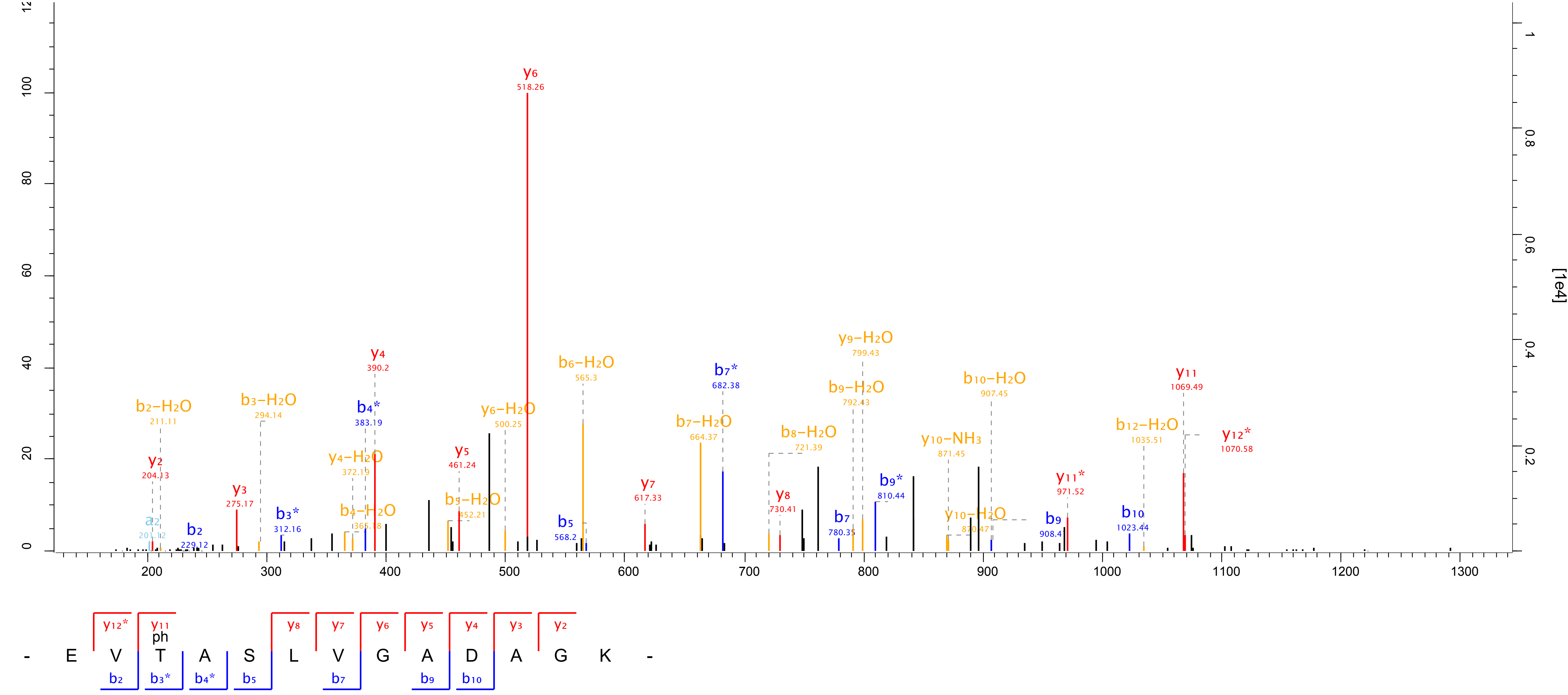

Raw file	Scan	Method	Score	m/z
20130708_PS_Phosphotest_6803wt_SDS01	6403	ITMS; CID	62.08	597.61

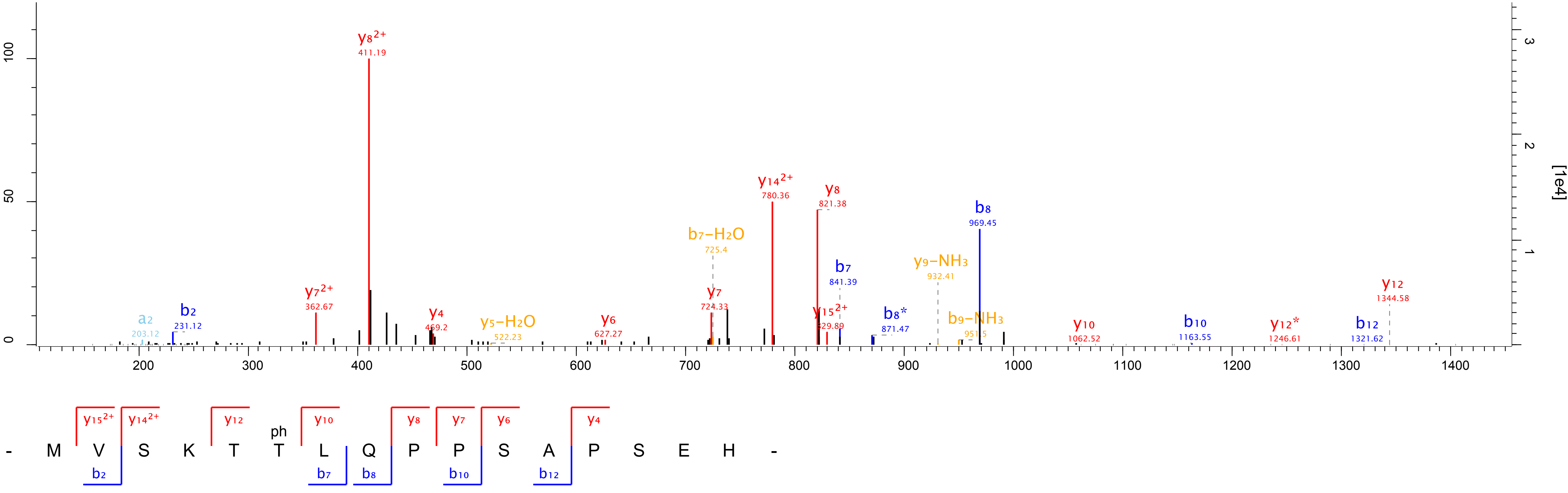

Raw file	Scan	Method	Score	m/z
20130708_PS_Phosphotest_6803wt_SDS01	6619	ITMS; CID	76.68	483.22

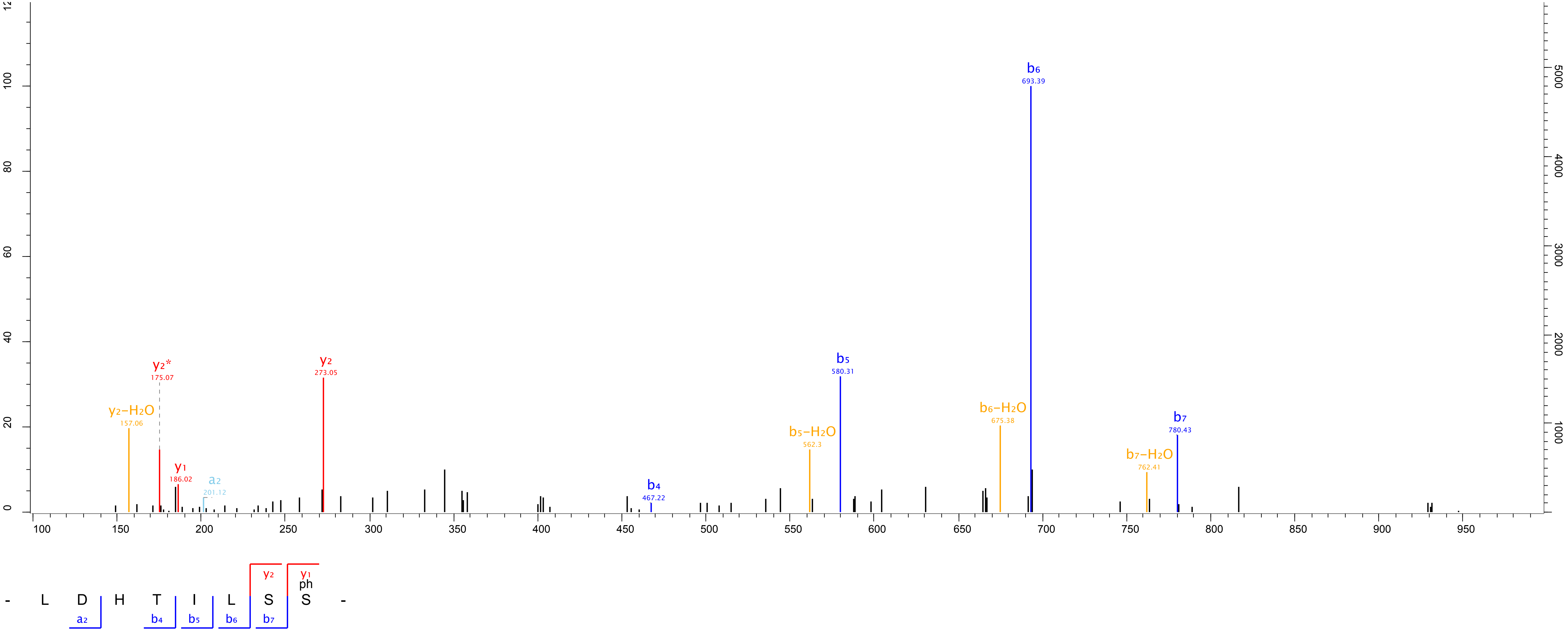

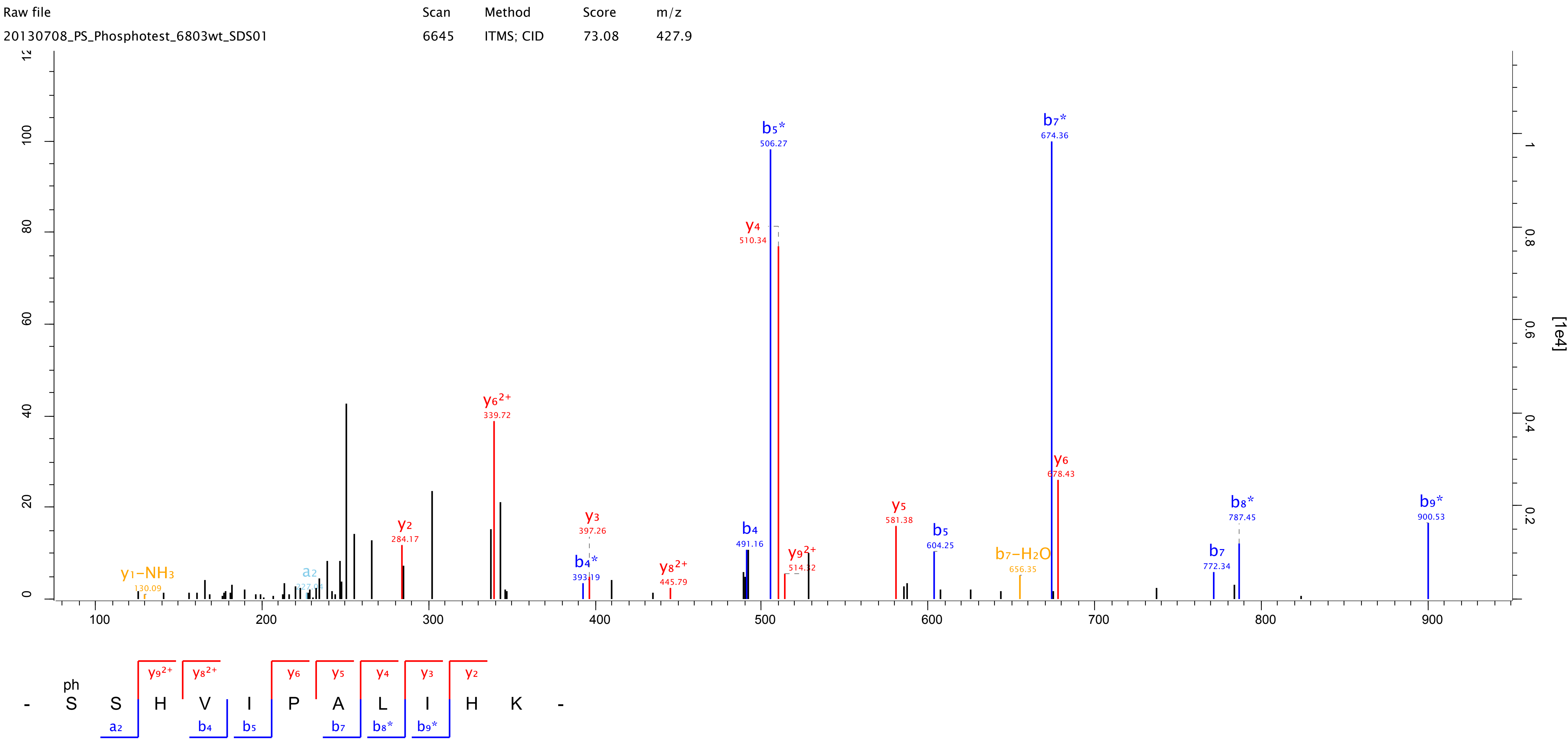

Raw file
20130708_PS_Phosphotest_6803wt_SDS01
Scan
6645
Method
ITMS; CID
Score
73.08
m/z
427.9
[1e4]
y1−NH3 130.09
a2 227.04
y2 284.17
y6 2+ 339.72
b4* 393.19
y3 397.26
y8 2+ 445.79
b4 491.16
b5* 506.27
y4 510.34
y9 2+ 514.32
y5 581.38
b5 604.25
b7−H2O 656.35
b7* 674.36
y6 678.43
b7 772.34
b8* 787.45
b9* 900.53
- ph S S H V I P A L I H K -

Raw file	Scan	Method	Score	m/z
20130708_PS_Phosphotest_6803wt_SDS01	6662	ITMS; CID	112.84	641.34

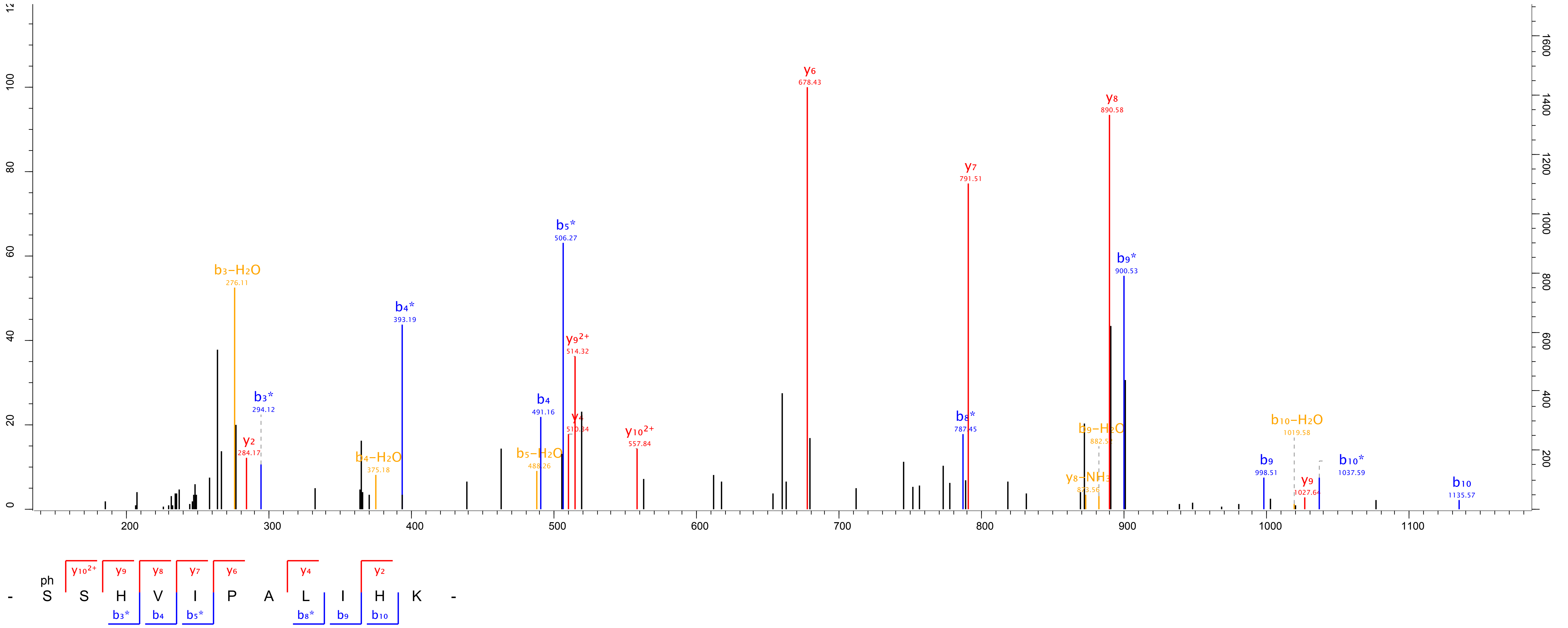

Raw file	Scan	Method	Score	m/z
20130708_PS_Phosphotest_6803wt_SDS01	6841	ITMS; CID	183.41	805.39

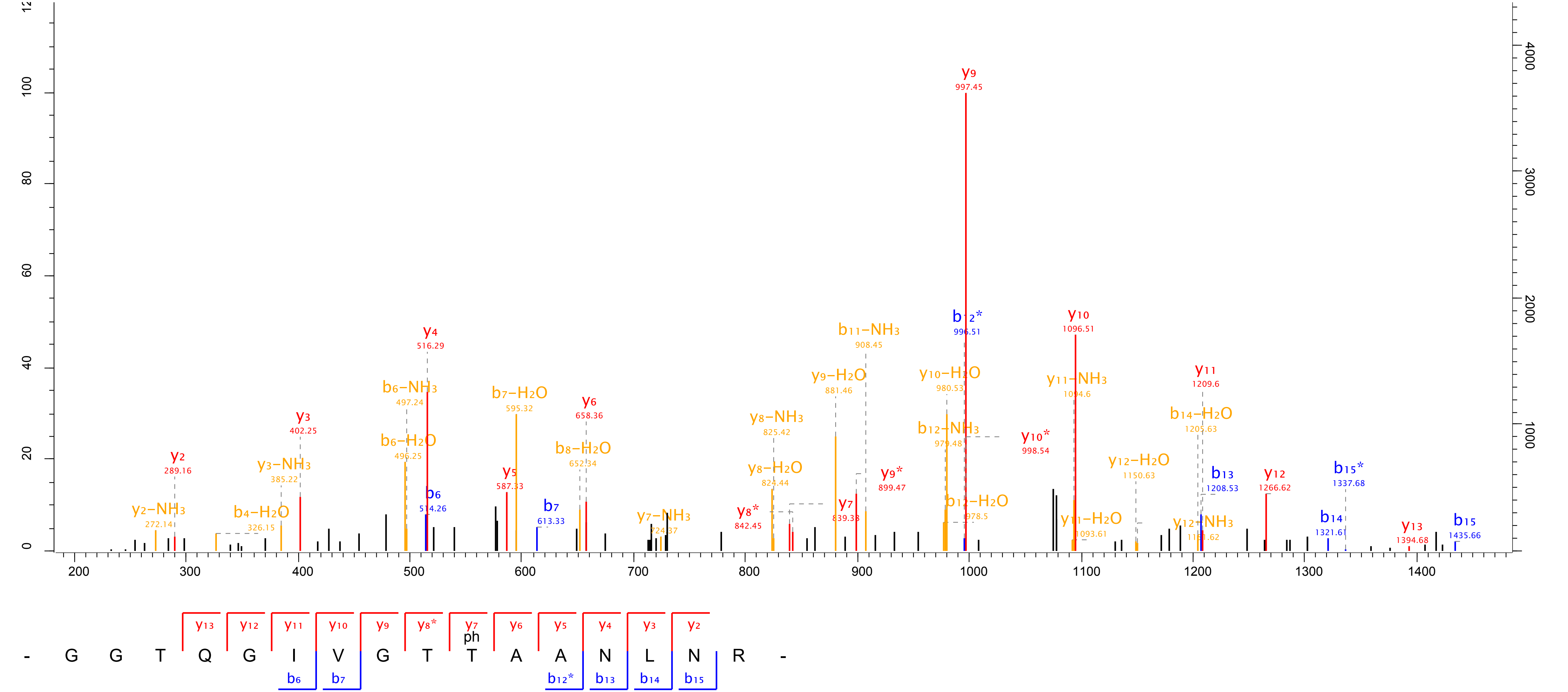

Raw file	Scan	Method	Score	m/z
20130708_PS_Phosphotest_6803wt_SDS01	6911	ITMS; CID	192.4	723.27

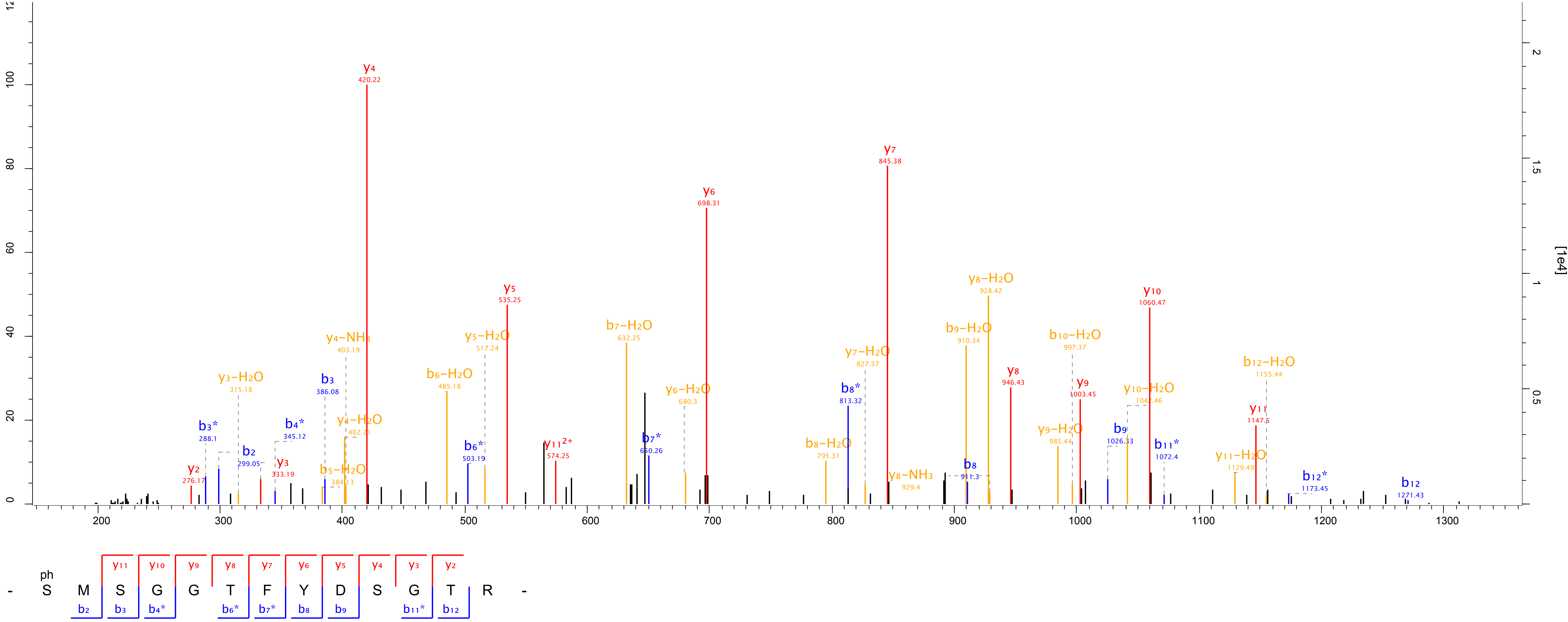

Raw file	Scan	Method	Score	m/z
20130708_PS_Phosphotest_6803wt_SDS01	6992	ITMS; CID	158.15	823.37

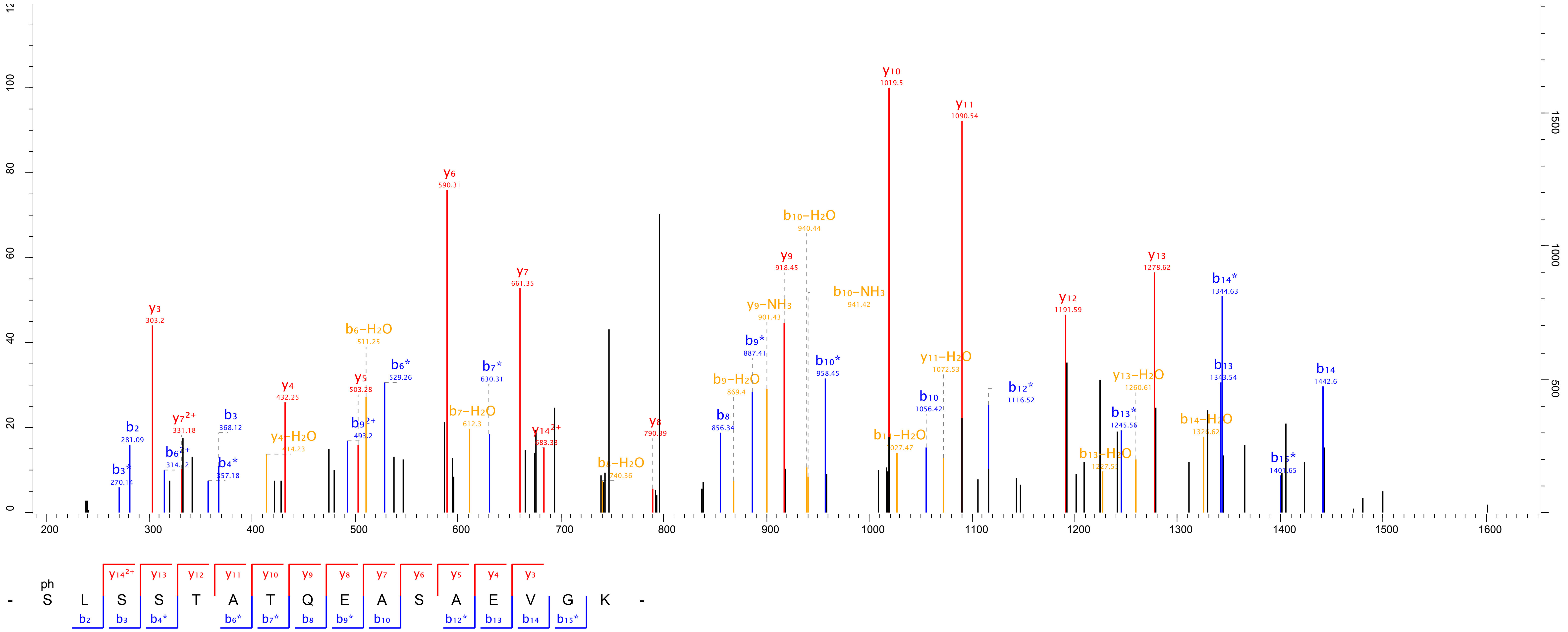

Raw file	Scan	Method	Score	m/z
20130708_PS_Phosphotest_6803wt_YPER03	7804	ITMS; CID	100.23	680.63

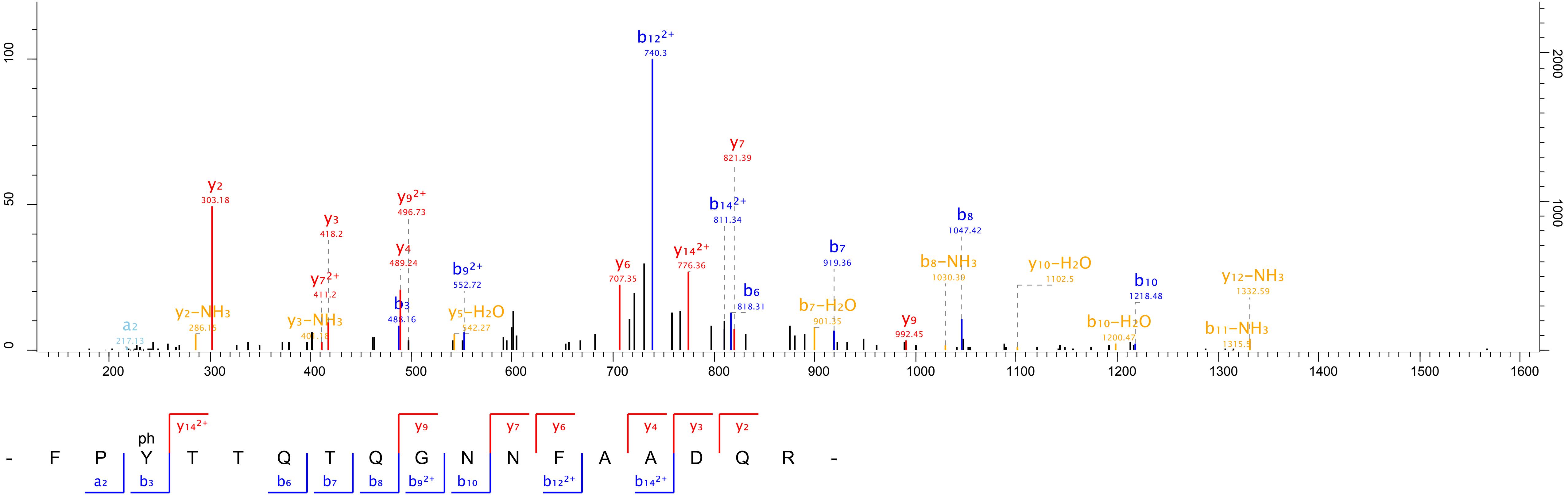

Raw file	Scan	Method	Score	m/z
20130708_PS_Phosphotest_6803wt_YPER01	7990	ITMS; CID	112.11	430.21

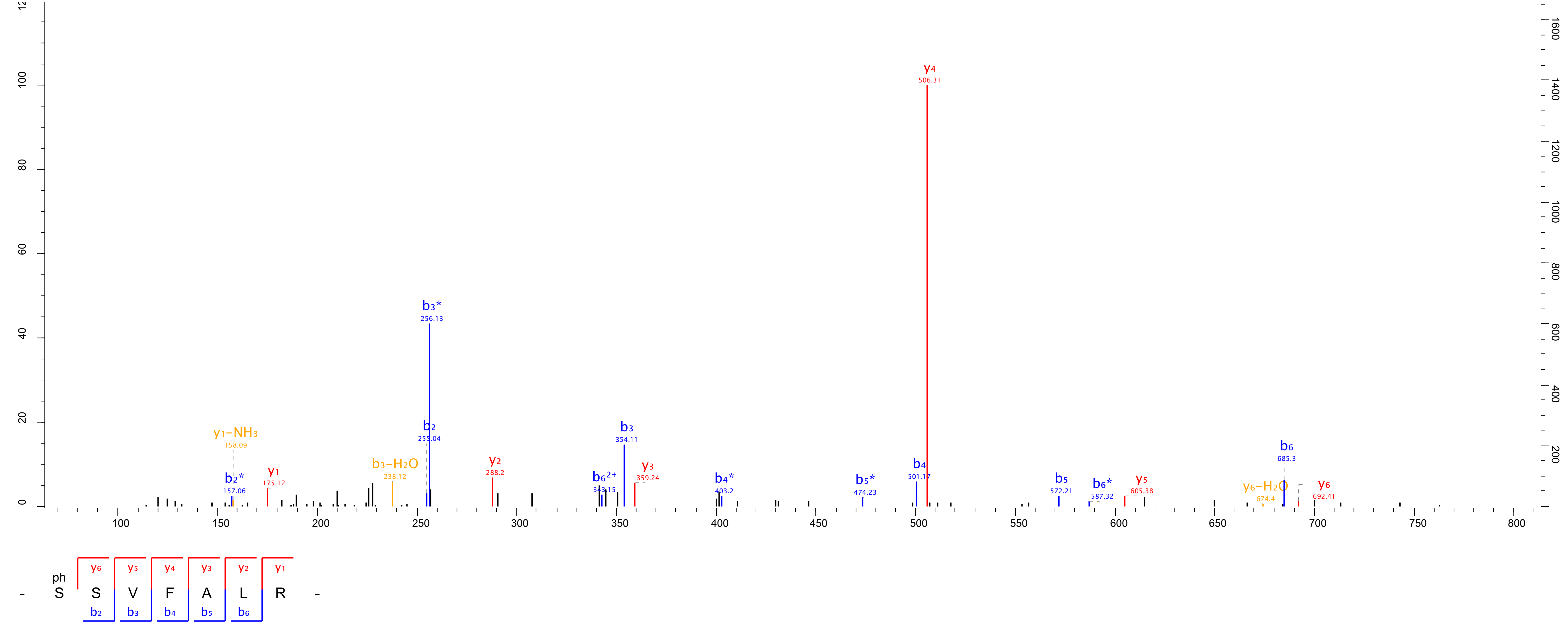

Raw file	Scan	Method	Score	m/z
20130708_PS_Phosphotest_6803wt_SDS01	8379	ITMS; CID	90.58	634.31

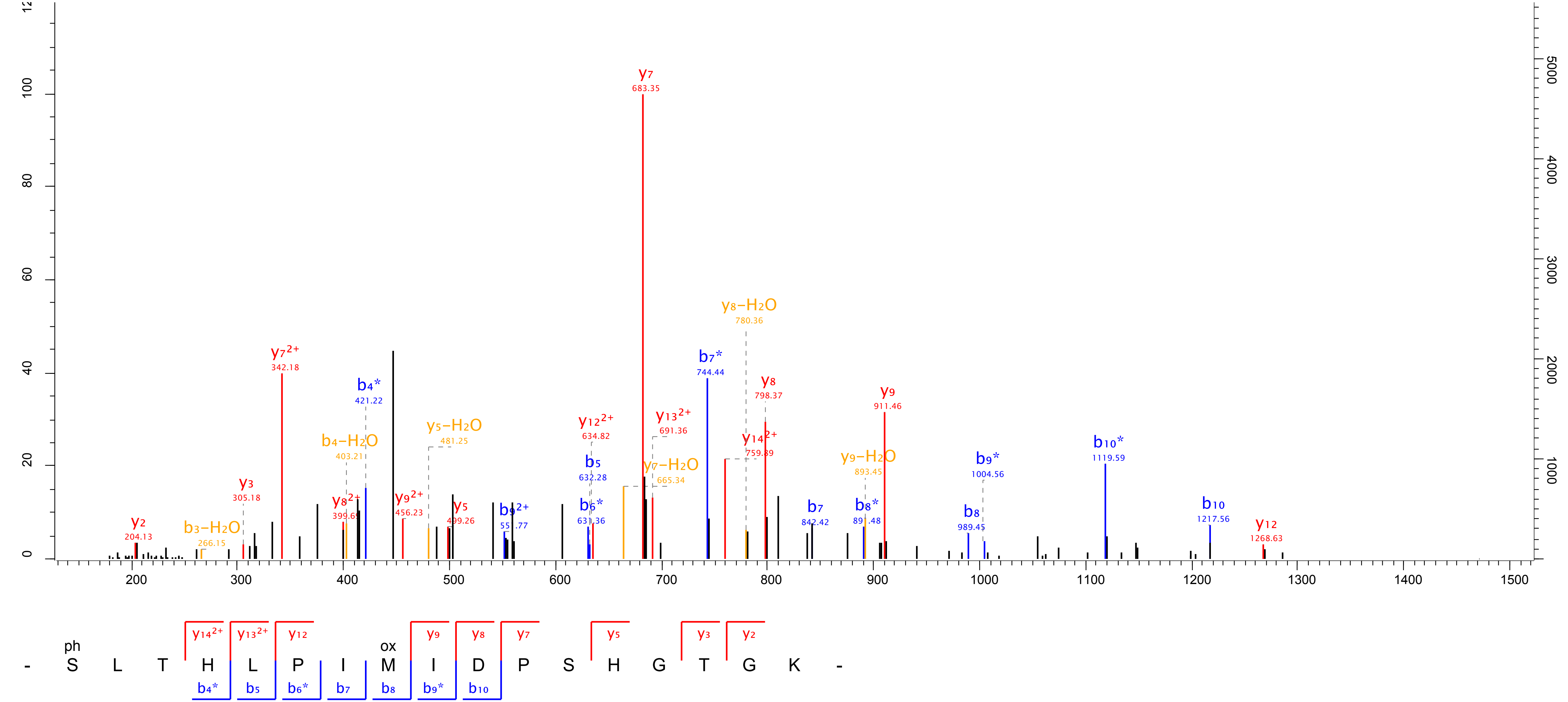

Raw file	Scan	Method	Score	m/z
20130708_PS_Phosphotest_6803wt_SDS01	9899	ITMS; CID	80.75	607.33

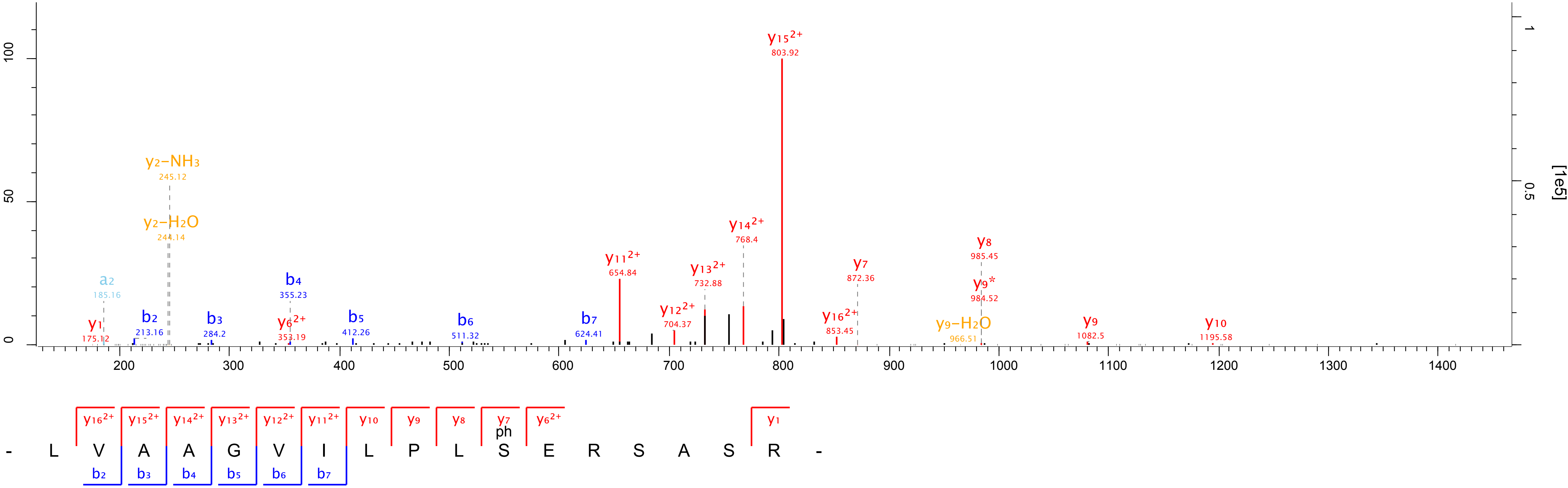

Quantitative Dataset

Phosphopeptide MS/MS Spectra (HCD)

Localized phosphorylation events

Raw file	Scan	Method	Score	m/z
20141031_PS_Syn6803wt_dimethyl_Mix_Phospho1	10035	FTMS; HCD	69.98	728.88

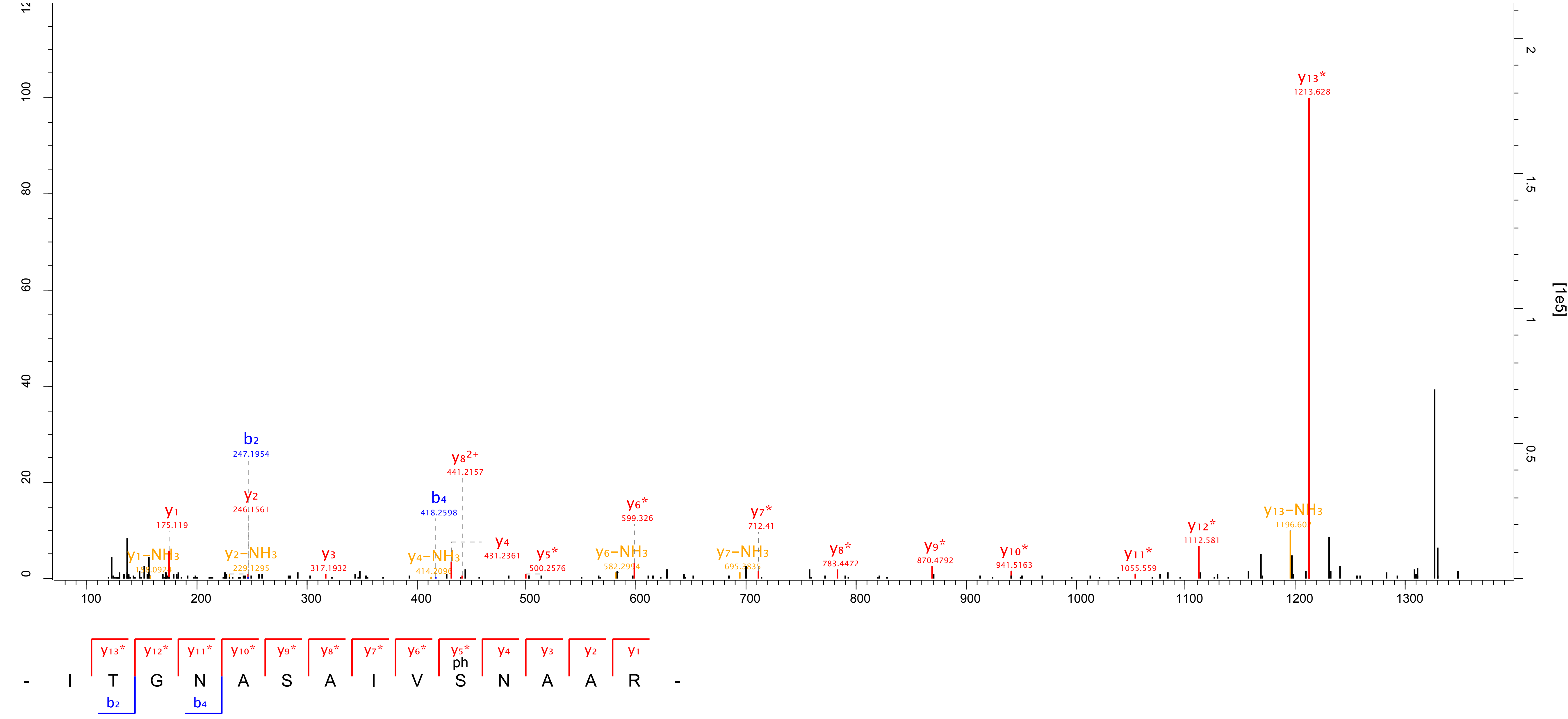

Raw file	Scan	Method	Score	m/z
20141031_PS_Syn6803wt_dimethyl_Mix_Phospho1	10249	FTMS; HCD	62.03	742.36

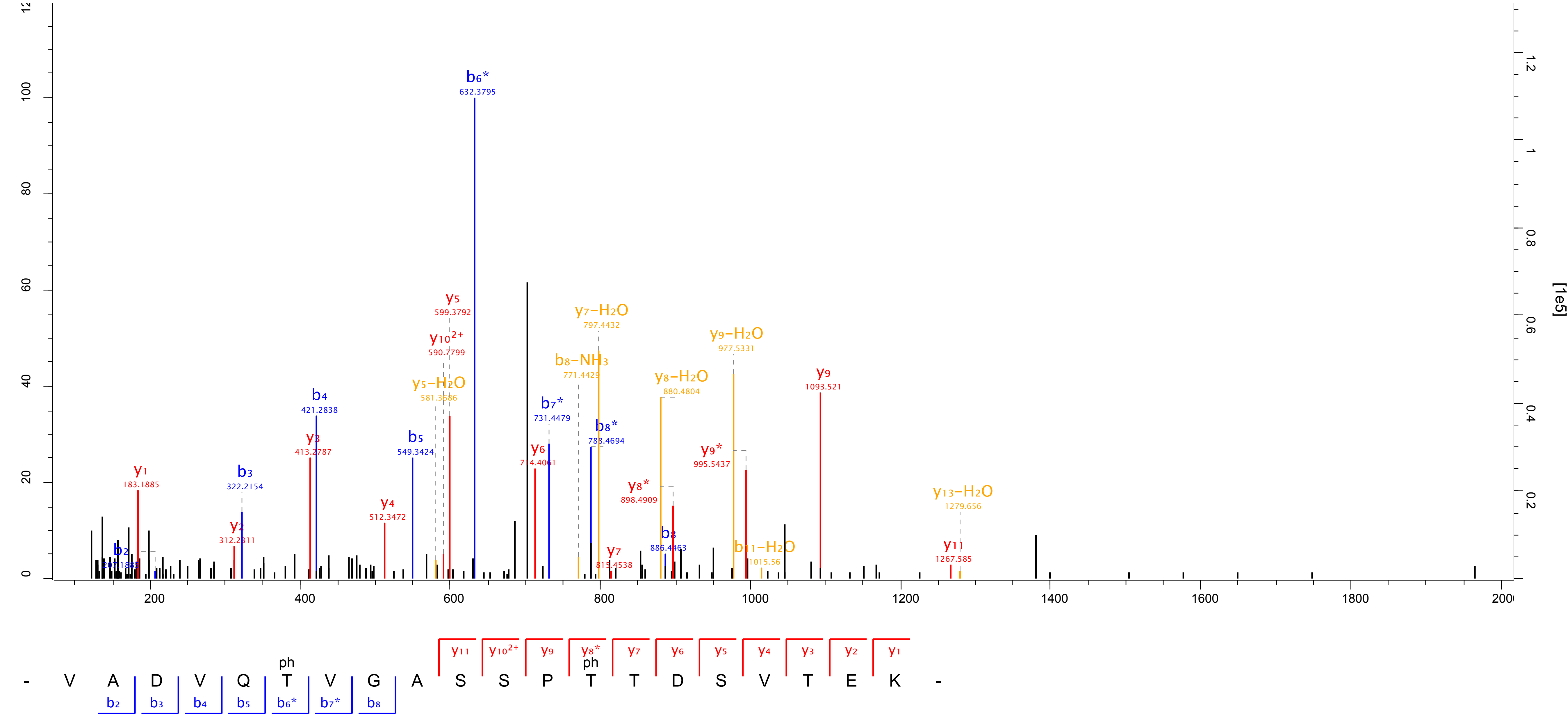

Raw file	Scan	Method	Score	m/z
20141031_PS_Syn6803wt_dimethyl_Mix_Phospho1	10558	FTMS; HCD	140.89	856.94

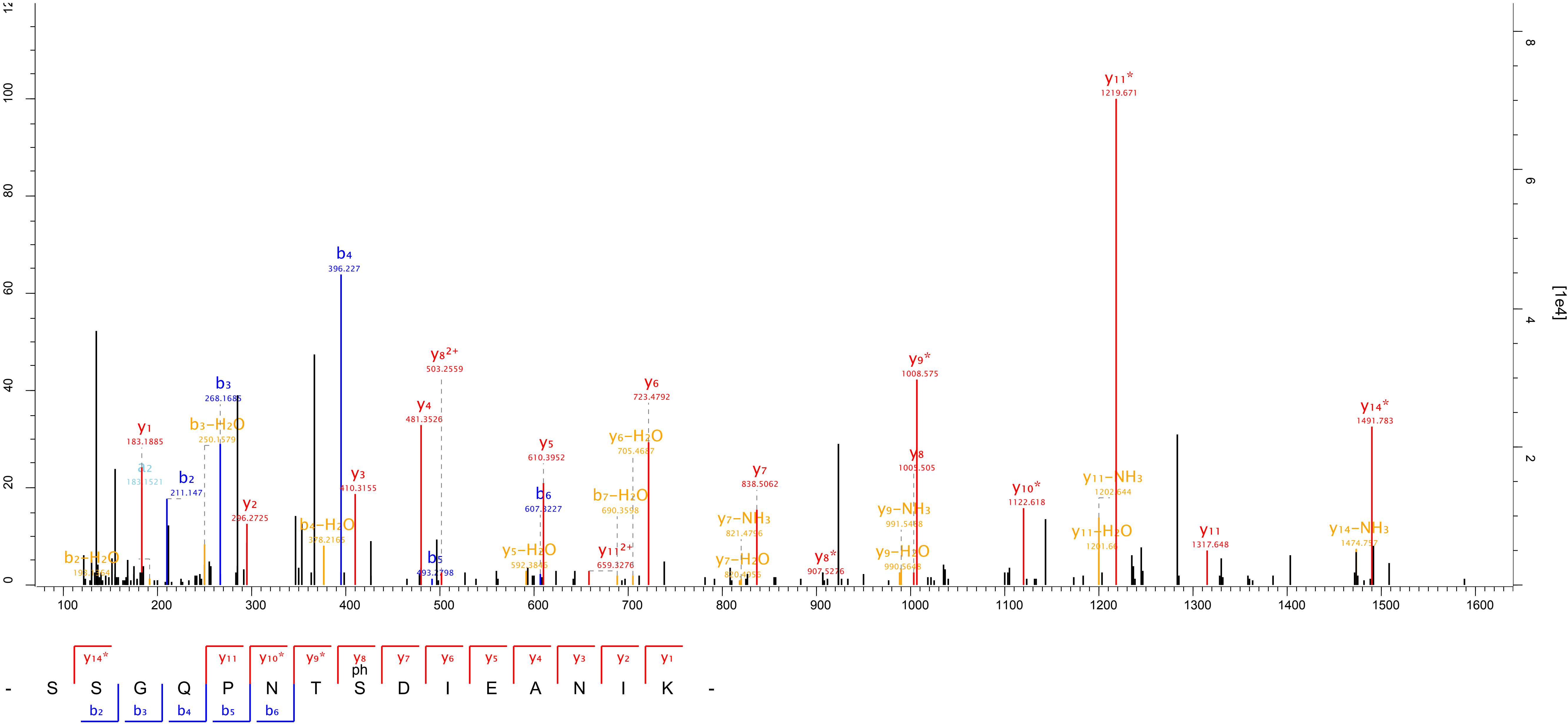

Raw file	Scan	Method	Score	m/z
20141031_PS_Syn6803wt_dimethyl_Mix_Phospho4	10679	FTMS; HCD	62.86	583.67

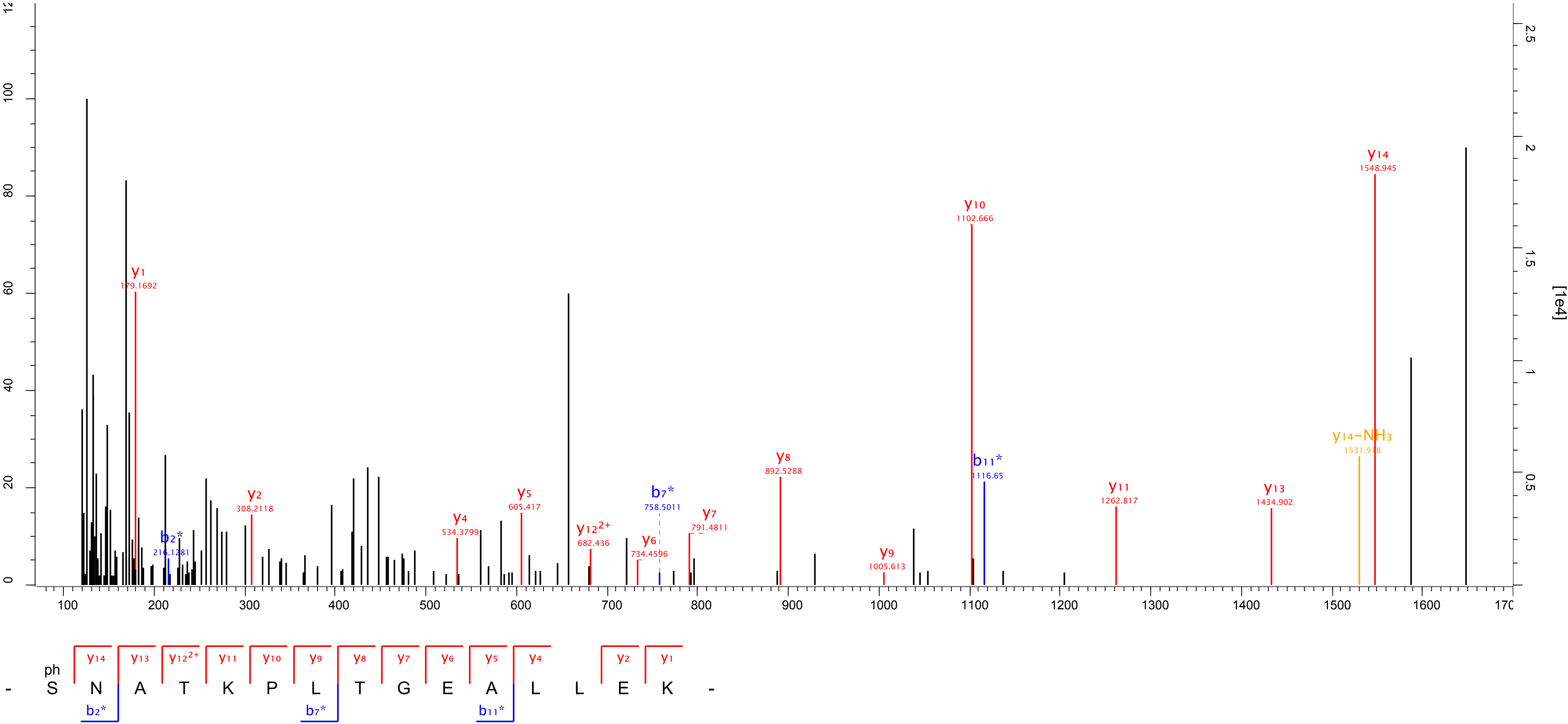

Raw file	Scan	Method	Score	m/z
20141031_PS_Syn6803wt_dimethyl_Mix_Phospho8	10735	FTMS; HCD	71.53	588.34

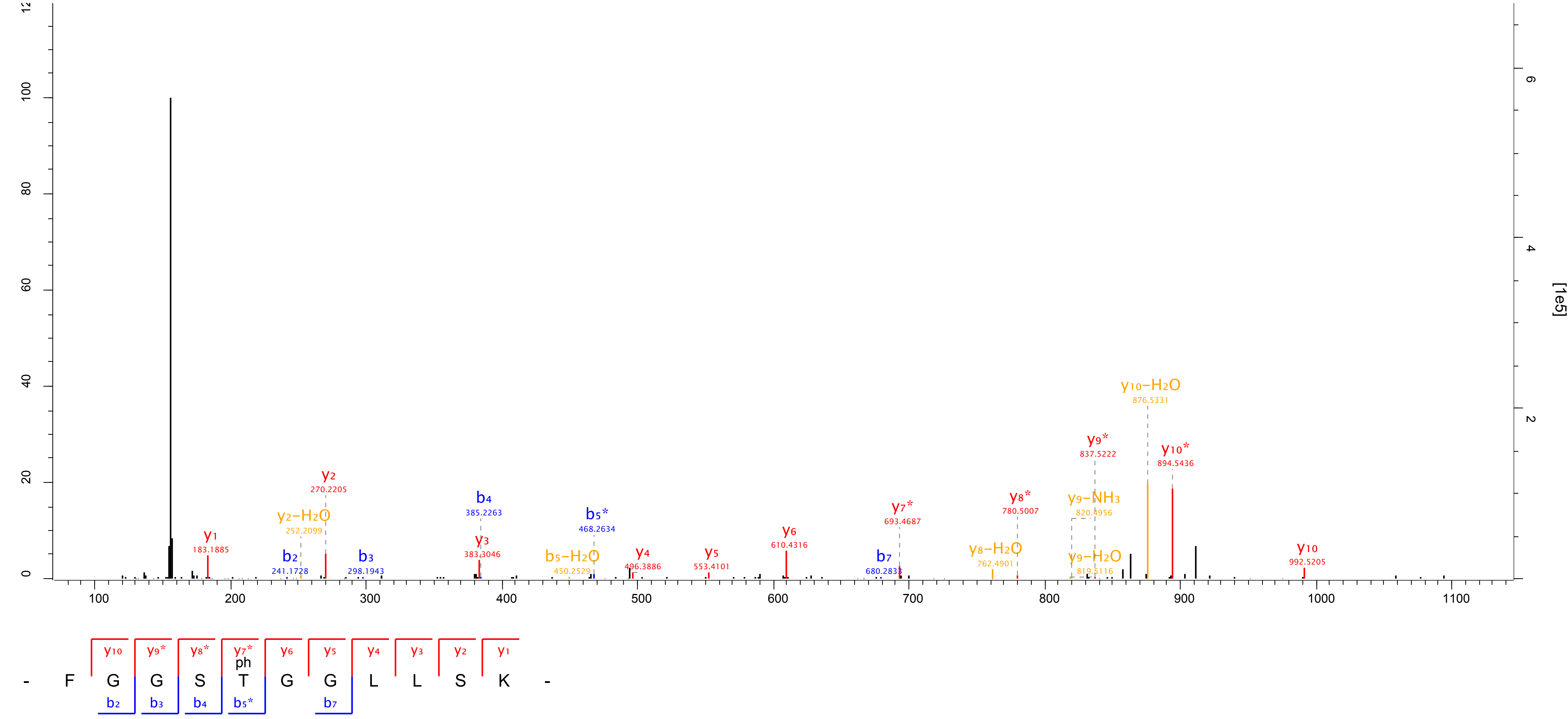

Raw file	Scan	Method	Score	m/z
201417_PS_Syn6803wt_dimethylMix_TiO2_1	10756	FTMS; HCD	64.73	901.79

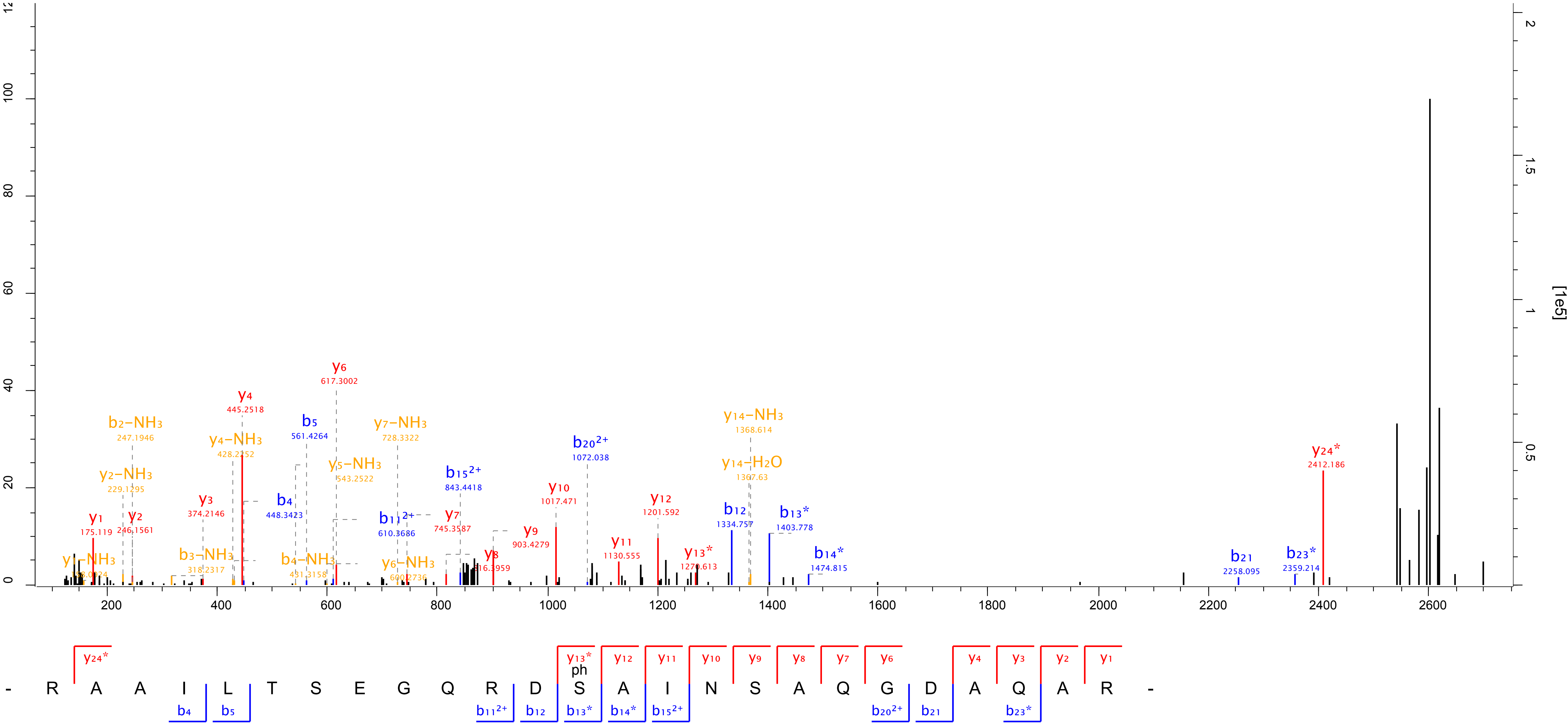

Raw file	Scan	Method	Score	m/z
201417_PS_Syn6803wt_dimethylMix_TiO2_4	10912	FTMS; HCD	105.08	690.83

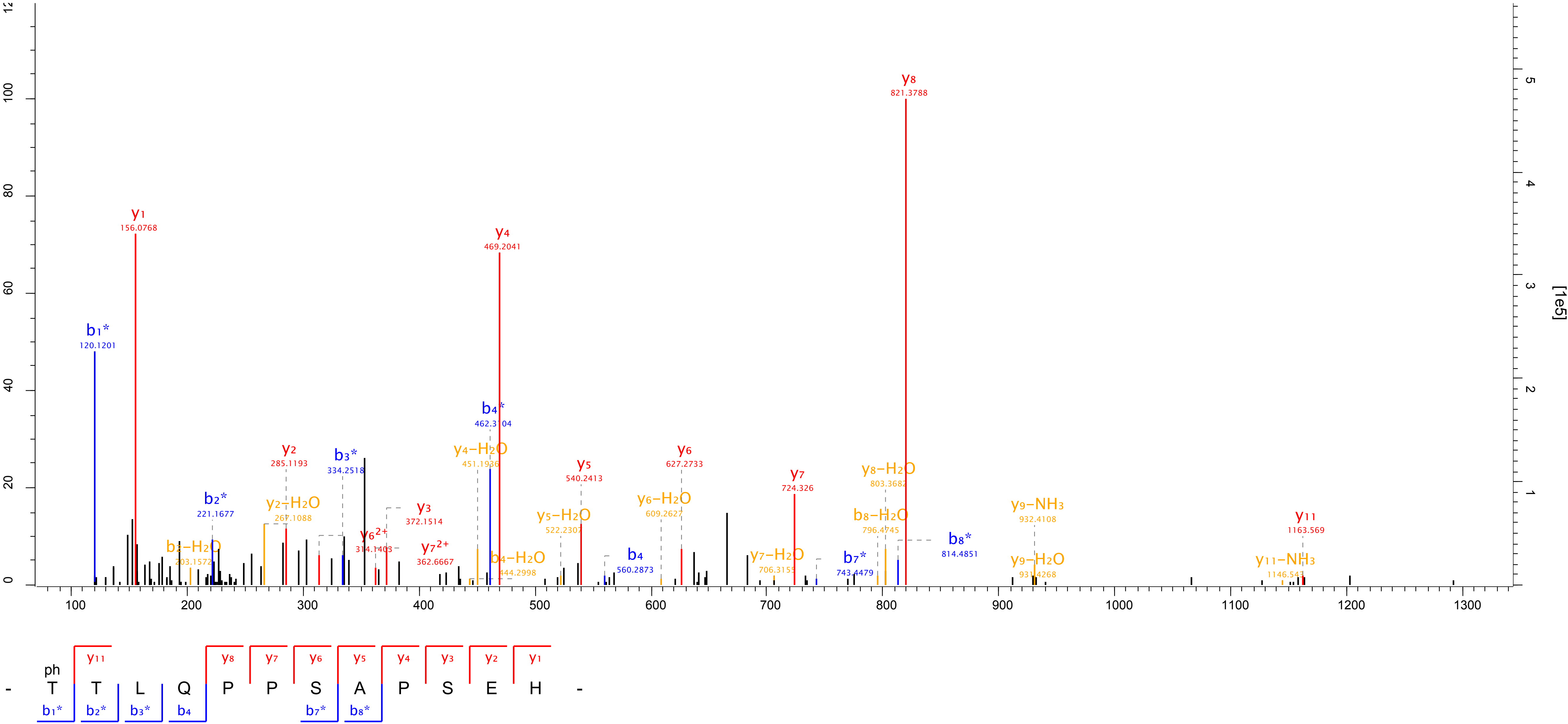

Raw file	Scan	Method	Score	m/z
20150122_PS_Syn_DimethylMix_Rep2_Phospho01	10990	FTMS; HCD	139.9	689.64

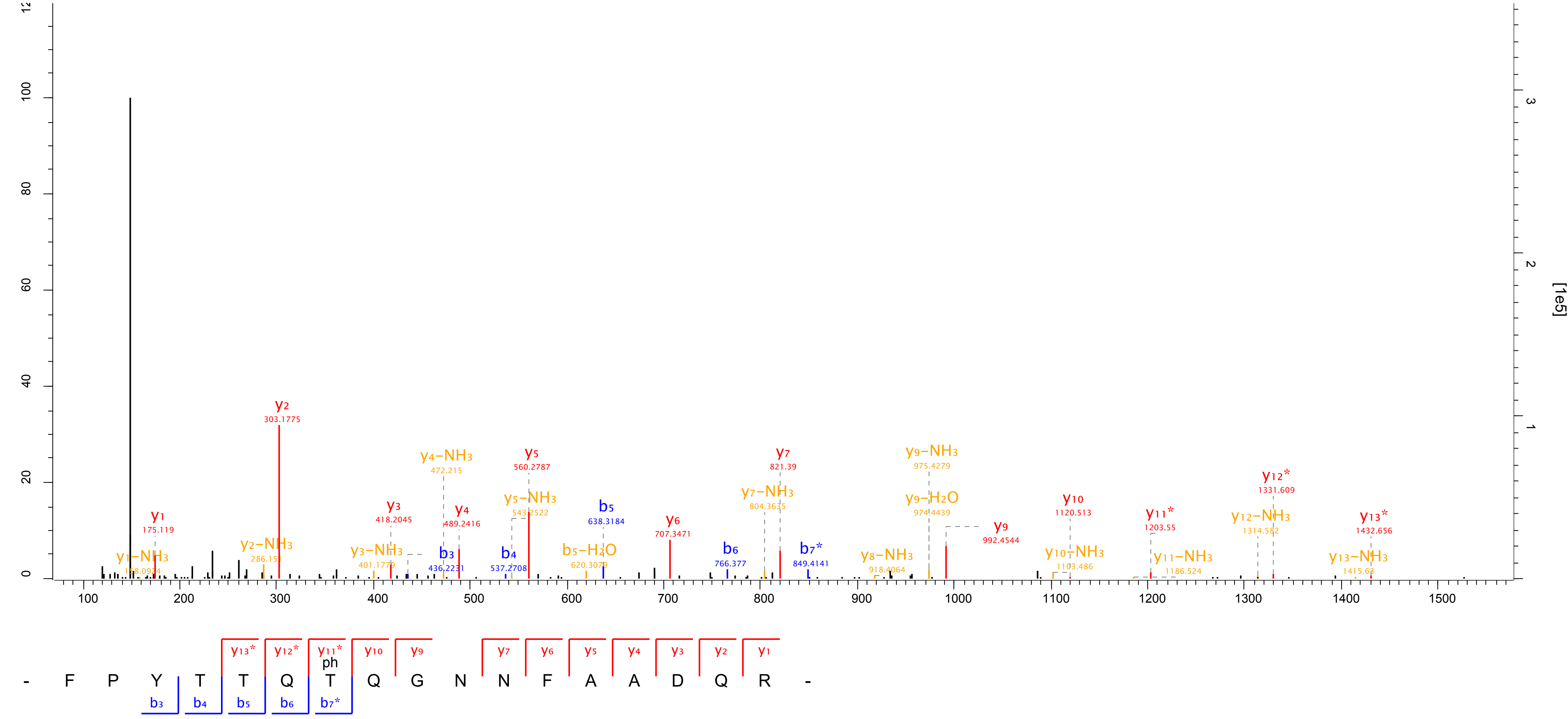

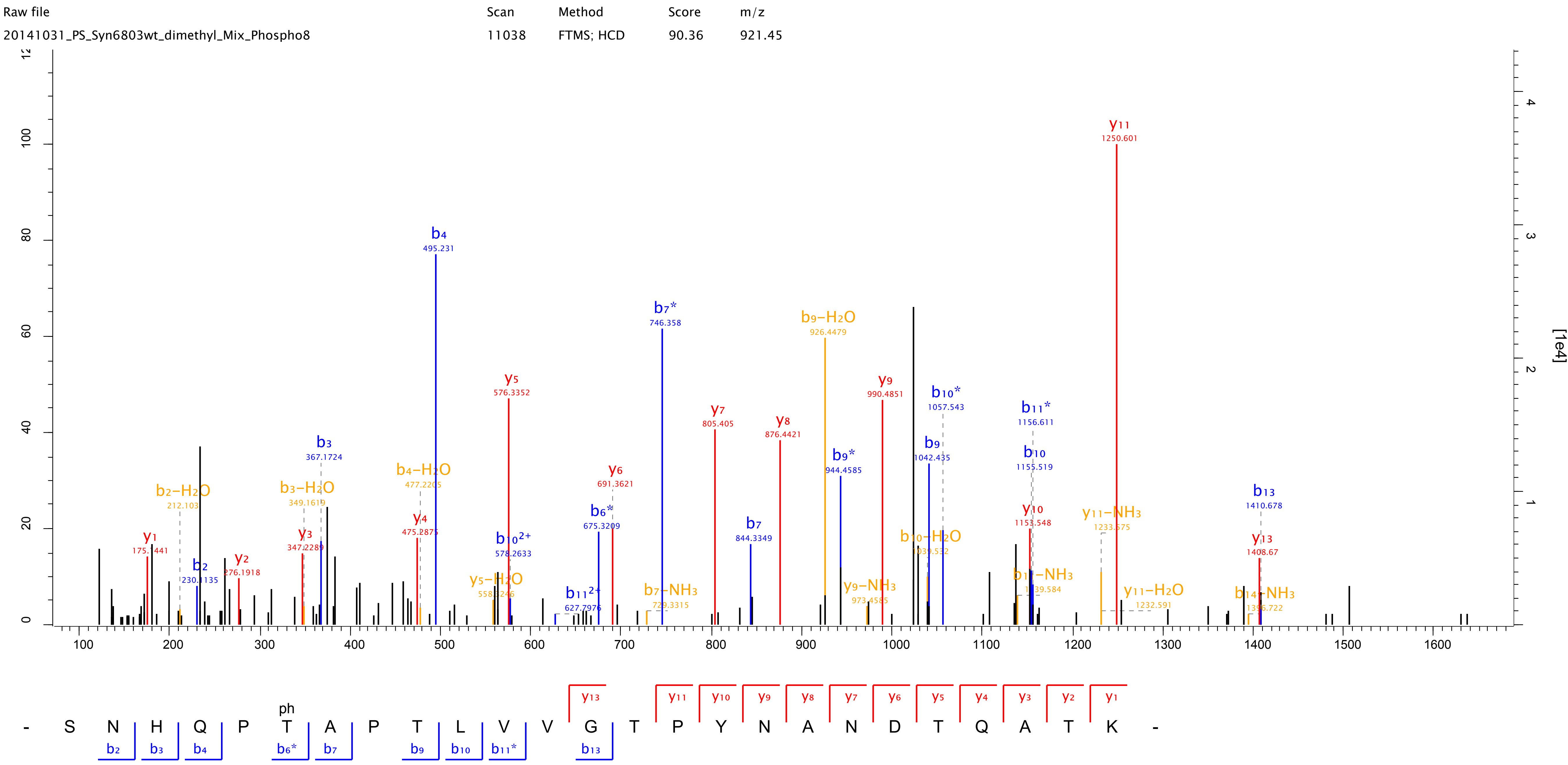
Raw file
20141031_PS_Syn6803wt_dimethyl_Mix_Phospho8
Scan
11038
Method
FTMS; HCD
Score
90.36
m/z
921.45
[1e4]
y11
1250.601
b4
495.231
b7*
746.358
b9-H2O
926.4479
y5
576.3352
y9
990.4851
b10*
1057.543
b11*
1156.611
y7
805.405
y8
876.4421
b3
367.1724
b9*
944.4585
b9
1042.435
b10
1155.519
b4-H2O
477.2205
y6
691.3621
b2-H2O
212.103
b3-H2O
b6*
675.3209
y4
475.2875
b7
844.3349
y11-NH3
y10
b13
1410.678
y13
1408.67
b10-H2O
b10 2+
578.2633
y3
y1
y2
276.1918
b2
b11-NH3
y5-H2O
b11 2+
627.7976
y9-NH3
b7-NH3
y11-H2O
1232.591
b14-NH3
- S N H Q P T A P T L V V G T P Y N A N D T Q A T K -
ph

Raw file	Scan	Method	Score	m/z
20150122_PS_Syn_DimethylMix_Rep2_Phospho01	11143	FTMS; HCD	51.77	653.35

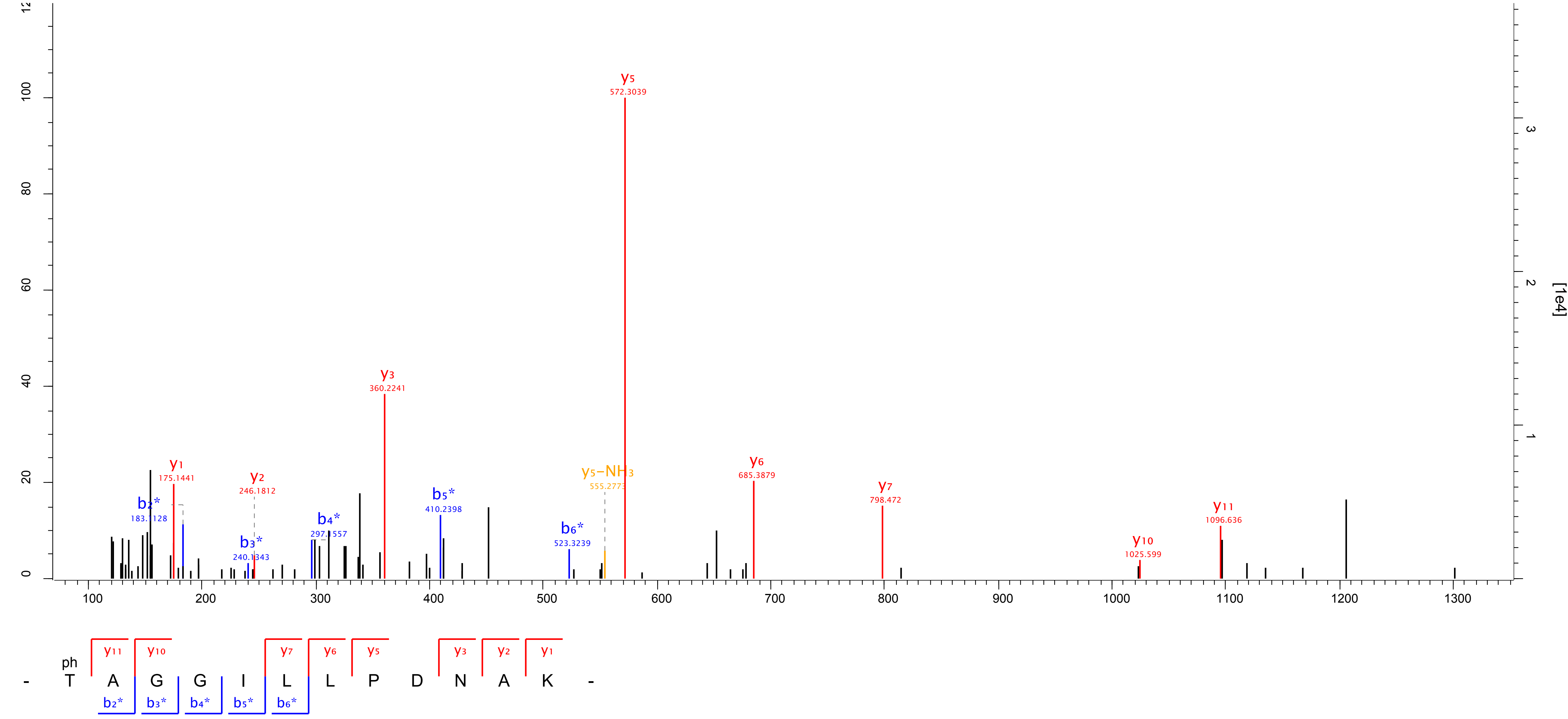

Raw file	Scan	Method	Score	m/z
20141031_PS_Syn6803wt_dimethyl_Mix_Phospho6	11190	FTMS; HCD	52.58	573.27

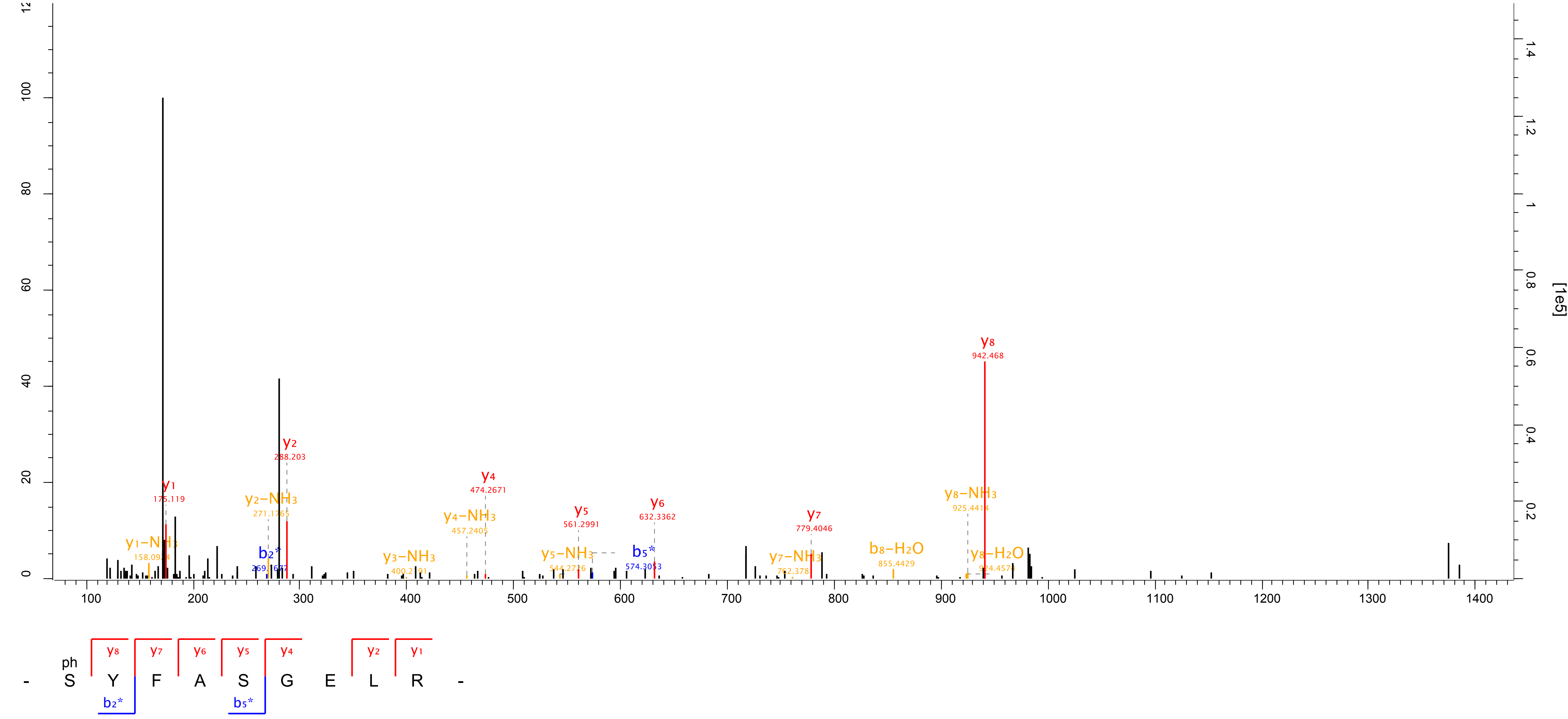

Raw file	Scan	Method	Score	m/z
20141031_PS_Syn6803wt_dimethyl_Mix_Phospho3	11302	FTMS; HCD	77.42	717.35

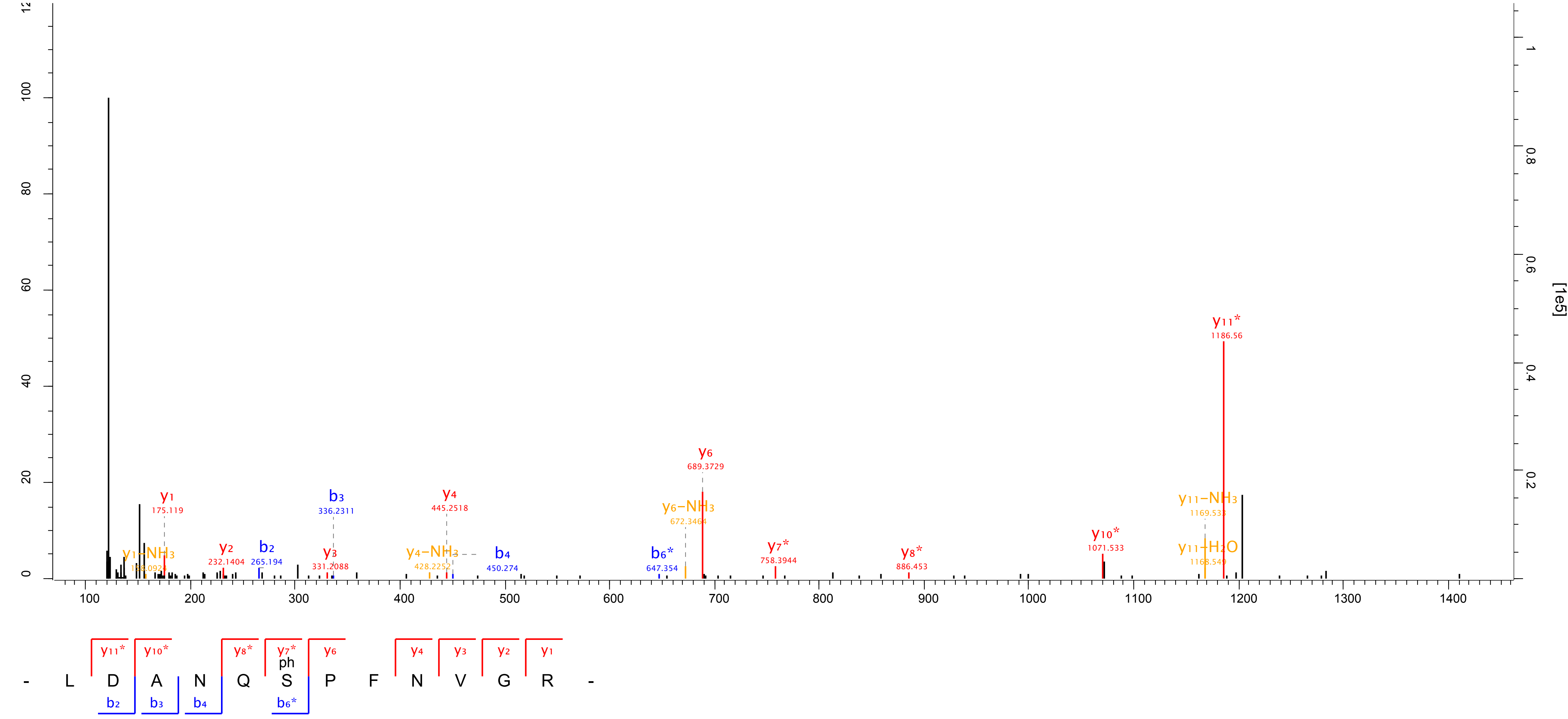

Raw file	Scan	Method	Score	m/z
20141031_PS_Syn6803wt_dimethyl_Mix_Phospho4	11517	FTMS; HCD	74.44	448.25

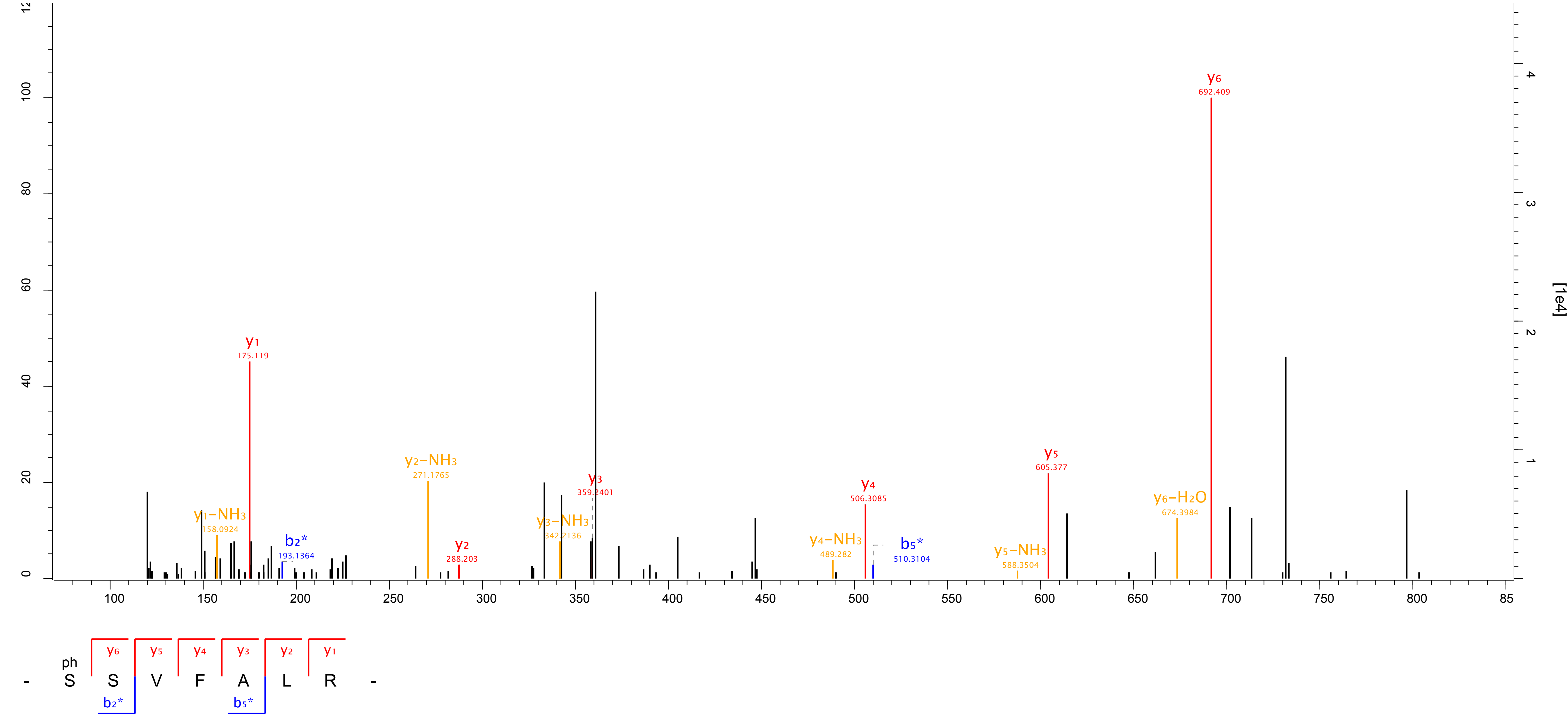

Raw file	Scan	Method	Score	m/z
20141031_PS_Syn6803wt_dimethyl_Mix_Phospho4	11560	FTMS; HCD	126.77	964.94

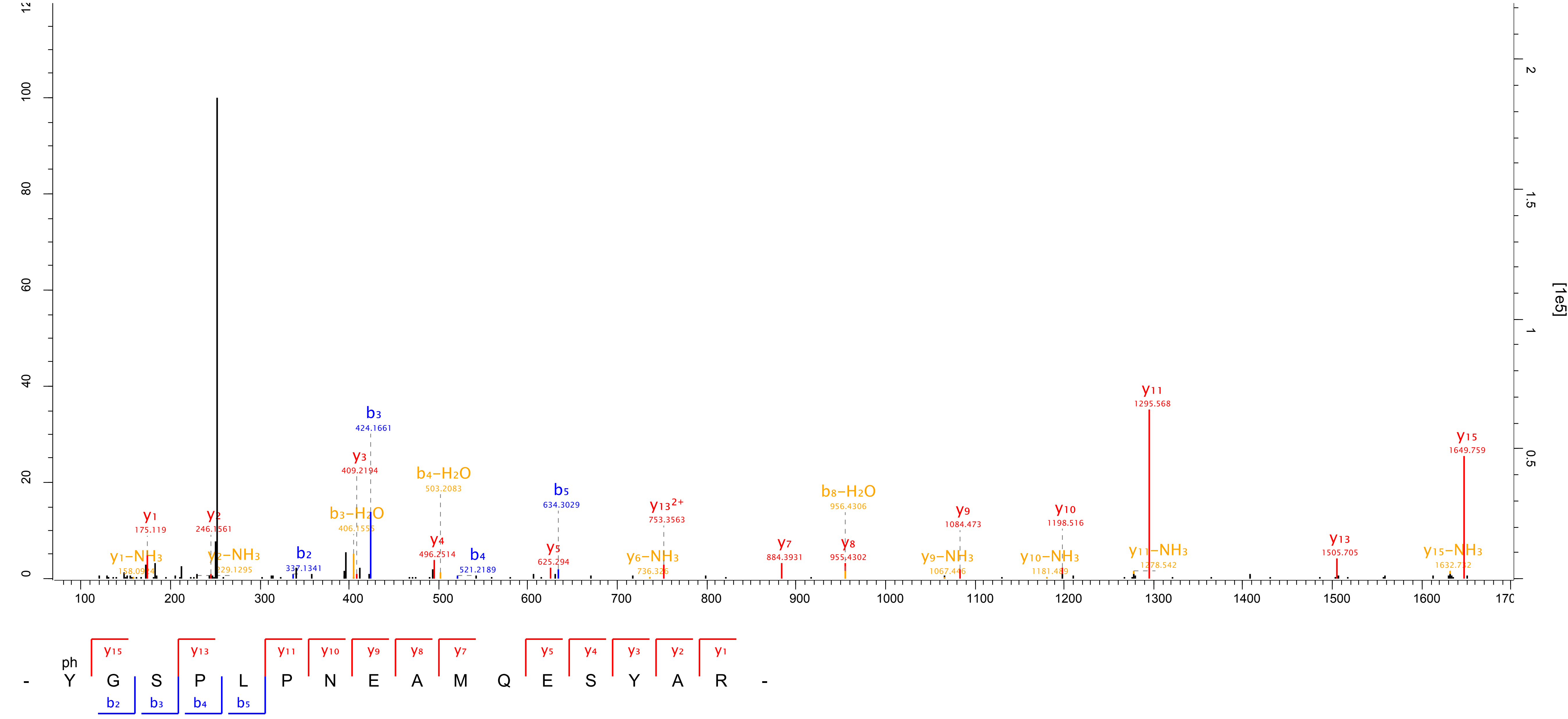

Raw file	Scan	Method	Score	m/z
20141031_PS_Syn6803wt_dimethyl_Mix_Phospho2	11598	FTMS; HCD	56.72	601.7

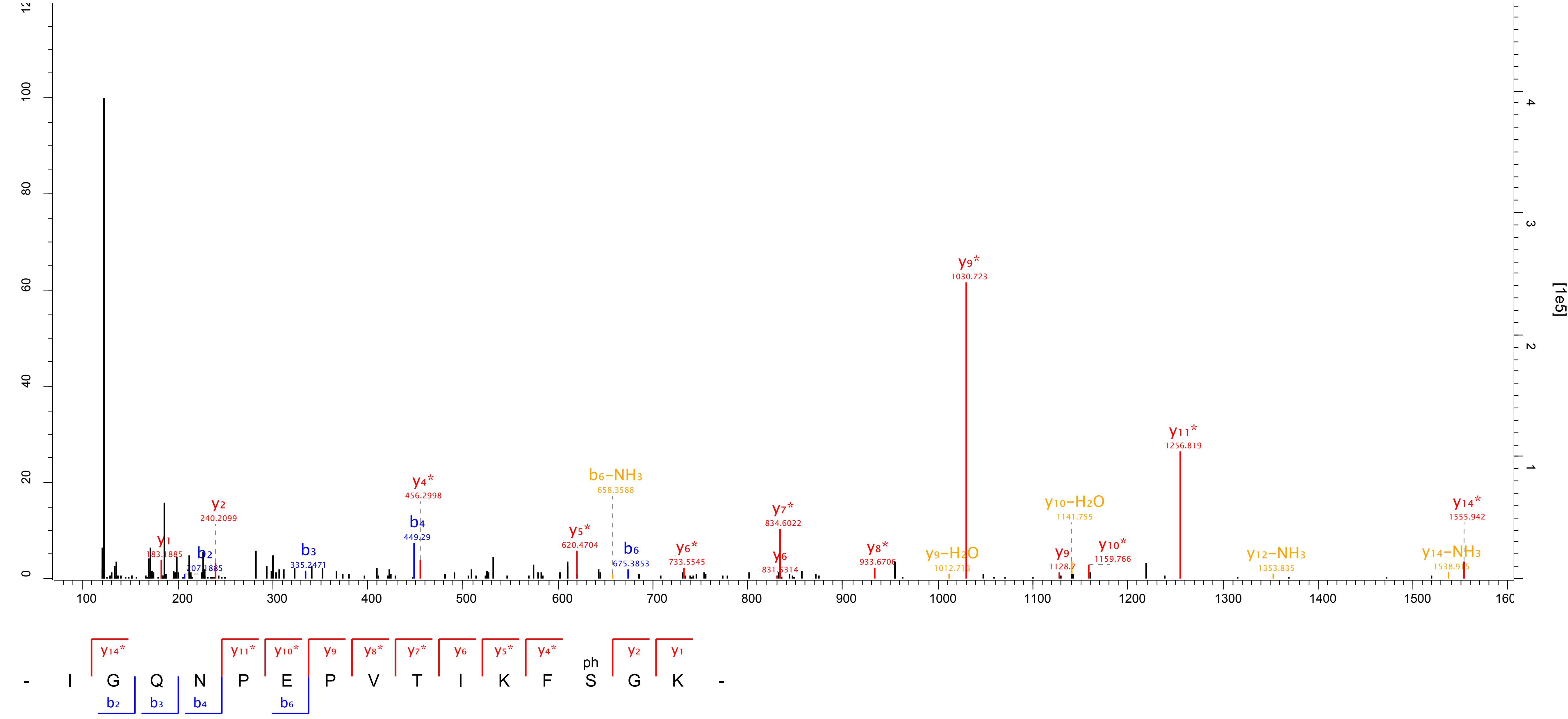

Raw file	Scan	Method	Score	m/z
20150122_PS_Syn_DimethylMix_Rep1_Phospho04	11726	FTMS; HCD	68.41	593.99

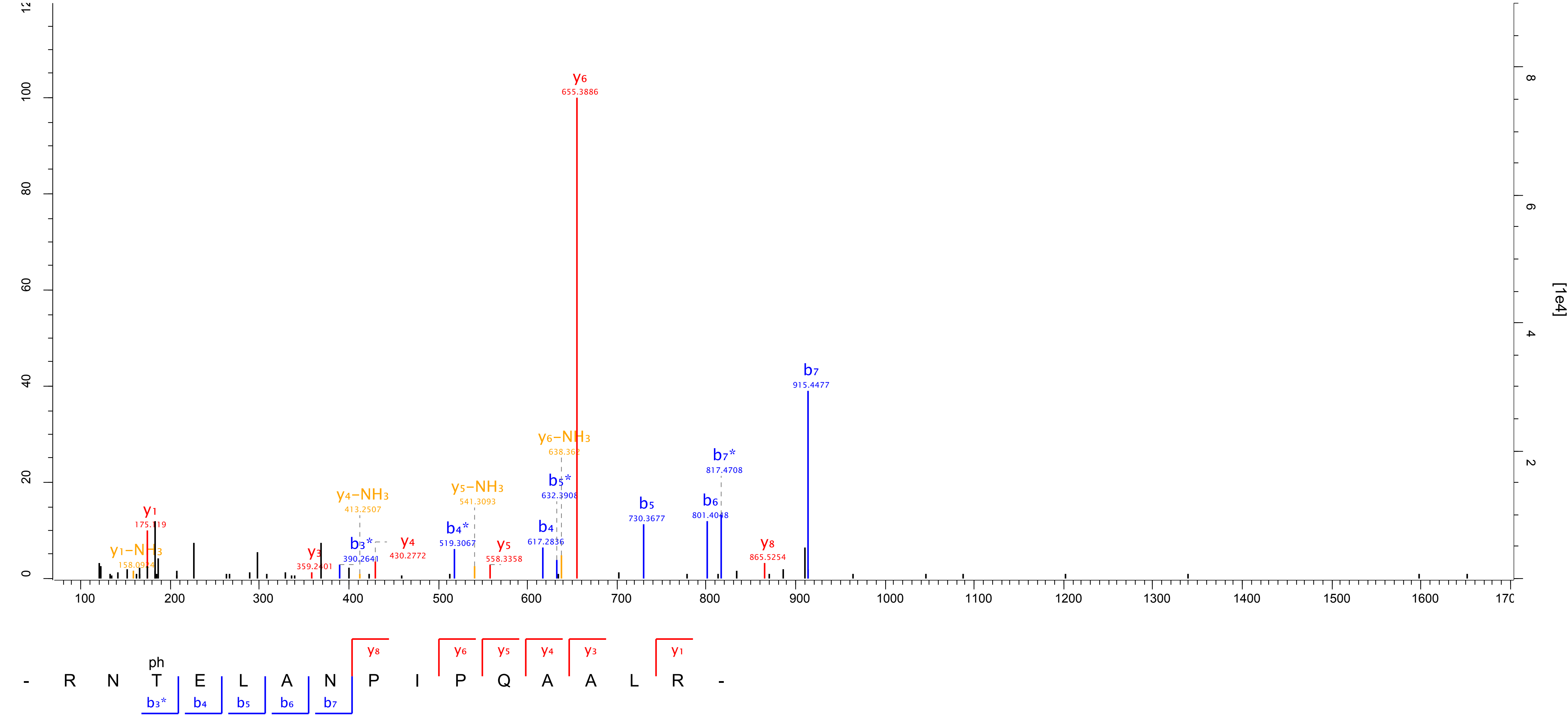

Raw file	Scan	Method	Score	m/z
20150122_PS_Syn_DimethylMix_Rep2_Phospho03	11757	FTMS; HCD	92.26	742.41

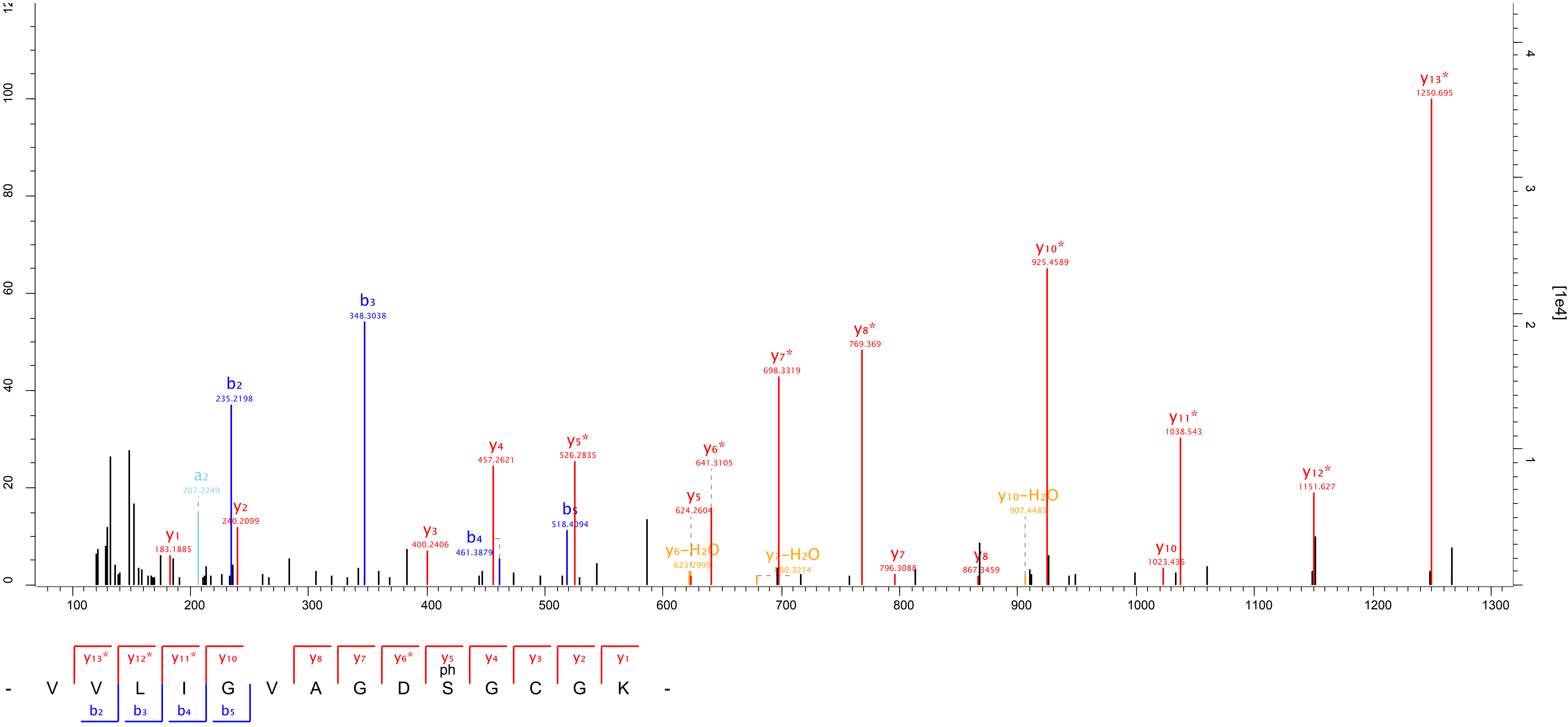

Raw file	Scan	Method	Score	m/z
20141031_PS_Syn6803wt_dimethyl_Mix_Phospho8	11761	FTMS; HCD	56.57	674.86

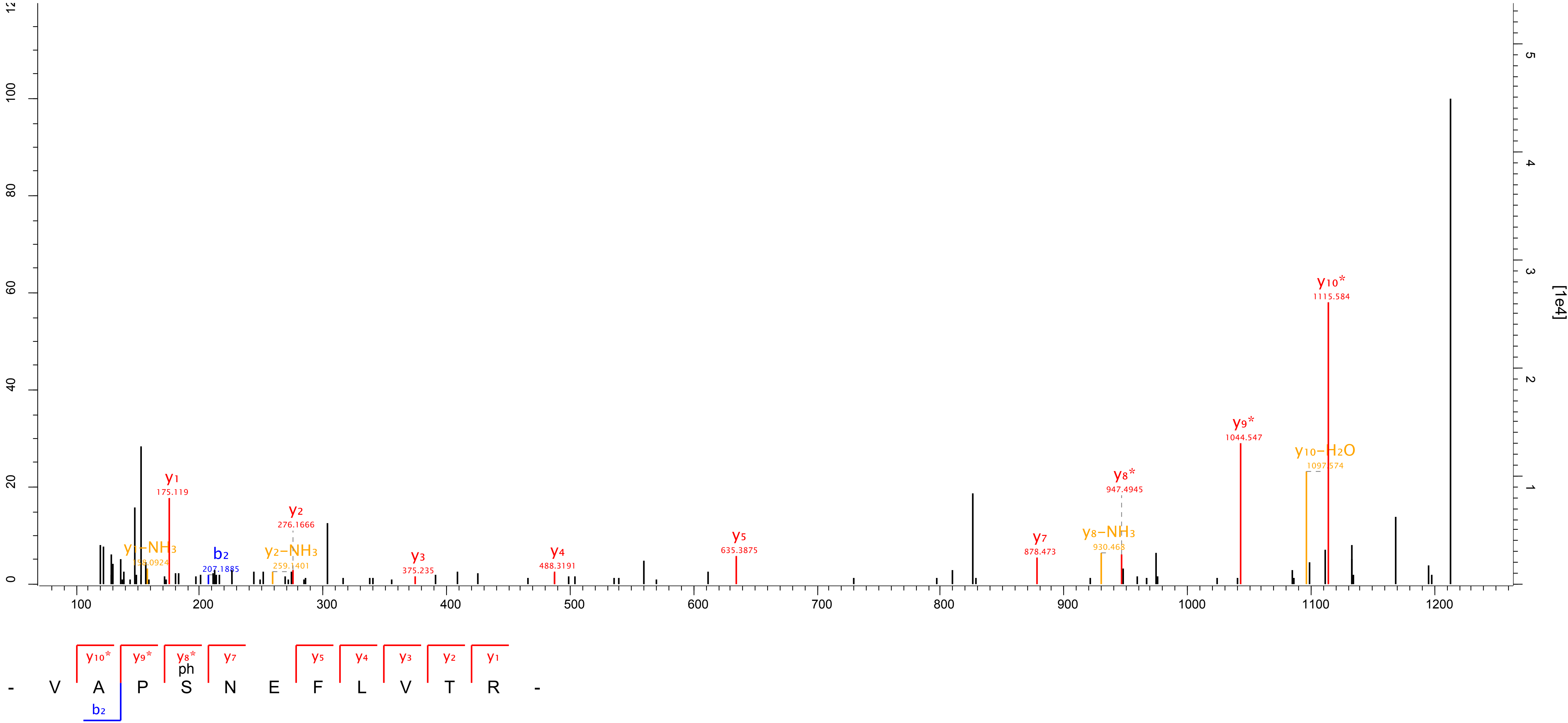

Raw file	Scan	Method	Score	m/z
20150122_PS_Syn_DimethylMix_Rep1_Phospho02	11994	FTMS; HCD	128.85	531.94

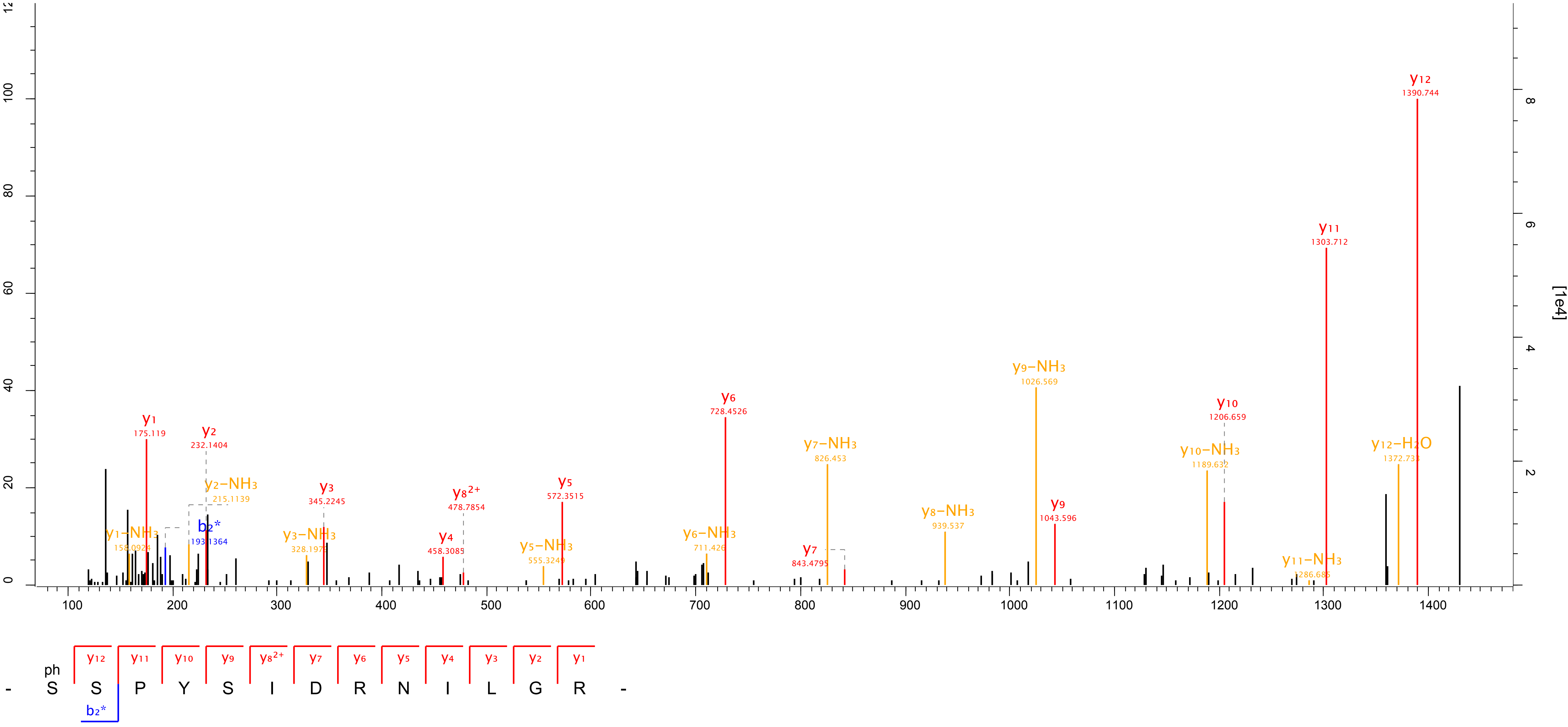

Raw file	Scan	Method	Score	m/z
201417_PS_Syn6803wt_dimethylMix_TiO2_1	12011	FTMS; HCD	72.21	997.47

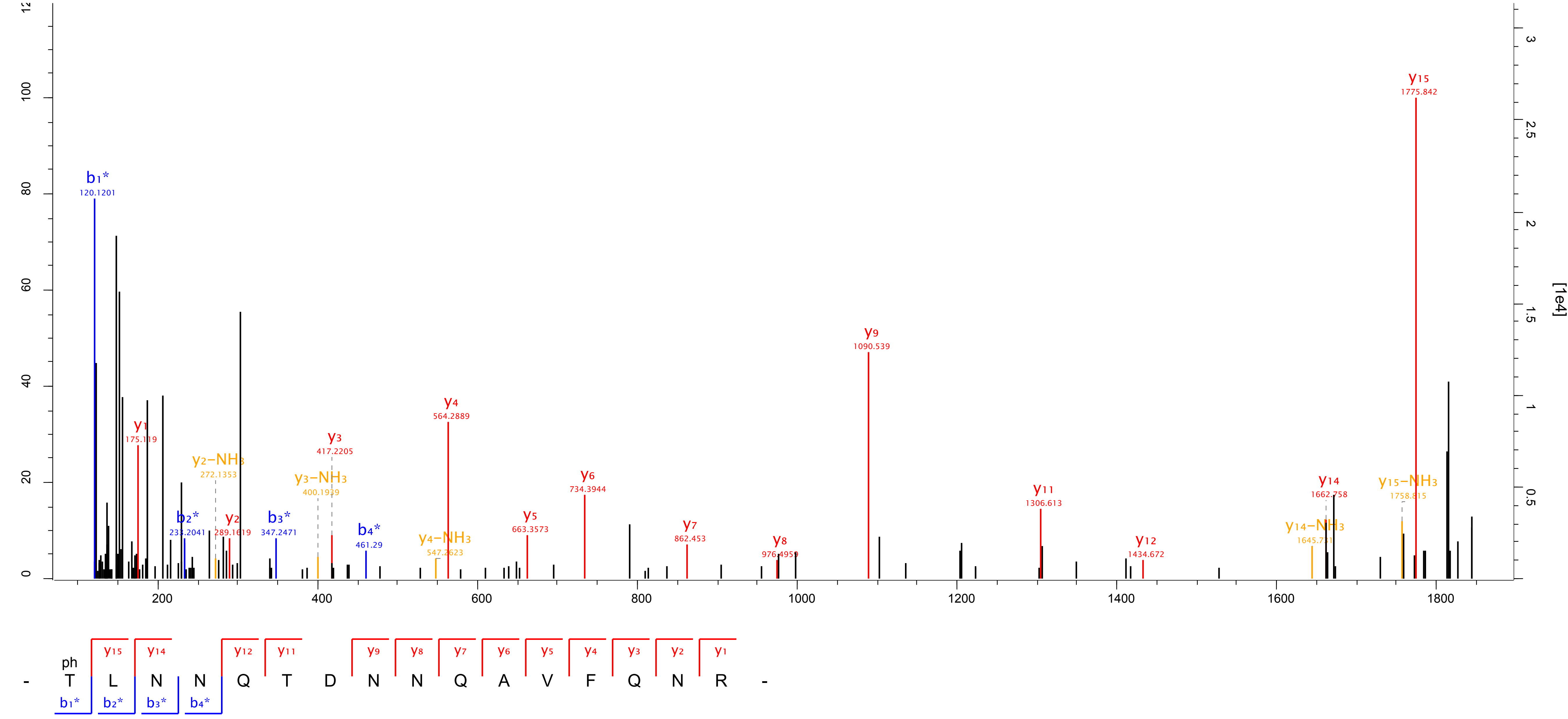

Raw file	Scan	Method	Score	m/z
20150122_PS_Syn_DimethylMix_Rep1_Phospho03	12154	FTMS; HCD	54.67	988.94

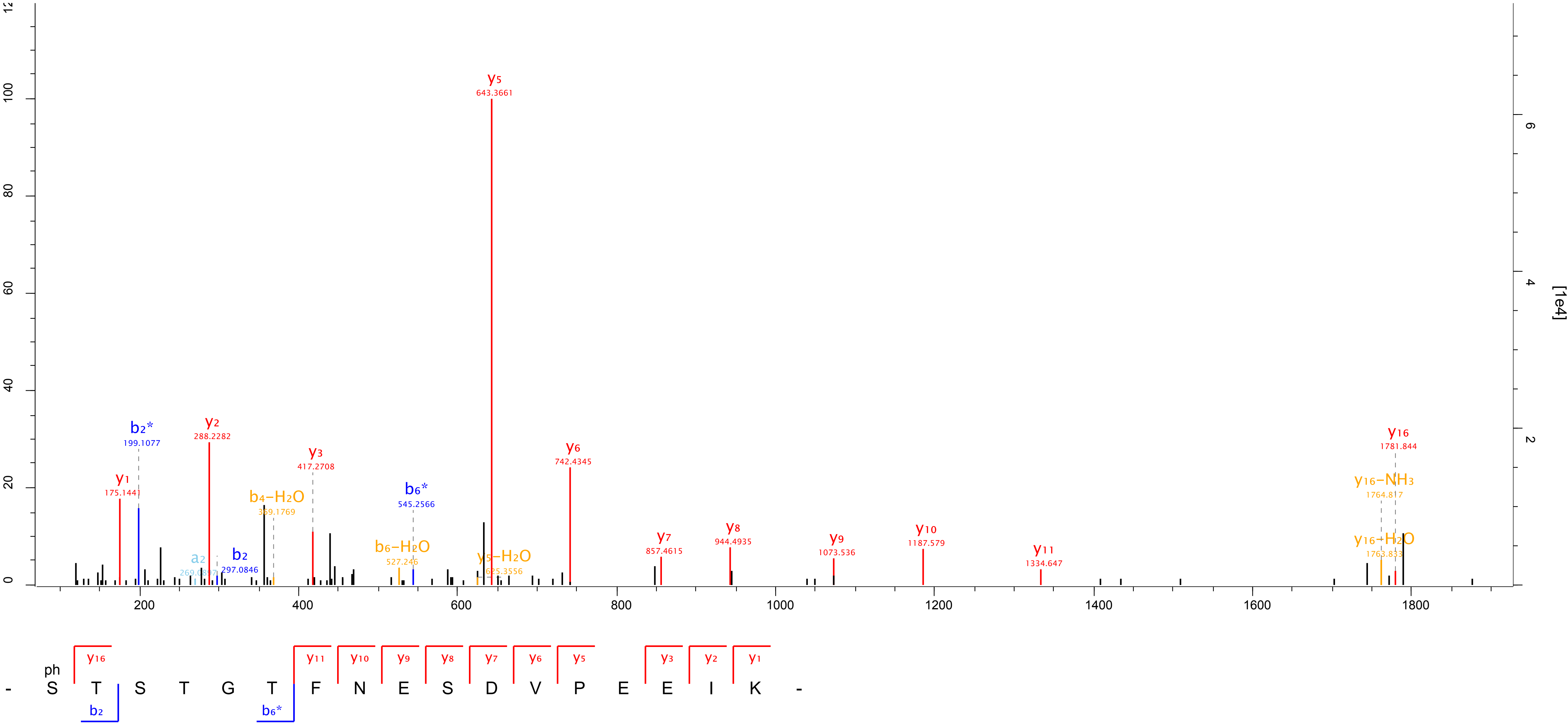

Raw file	Scan	Method	Score	m/z
20141031_PS_Syn6803wt_dimethyl_Mix_Phospho3	12154	FTMS; HCD	113.73	720.72

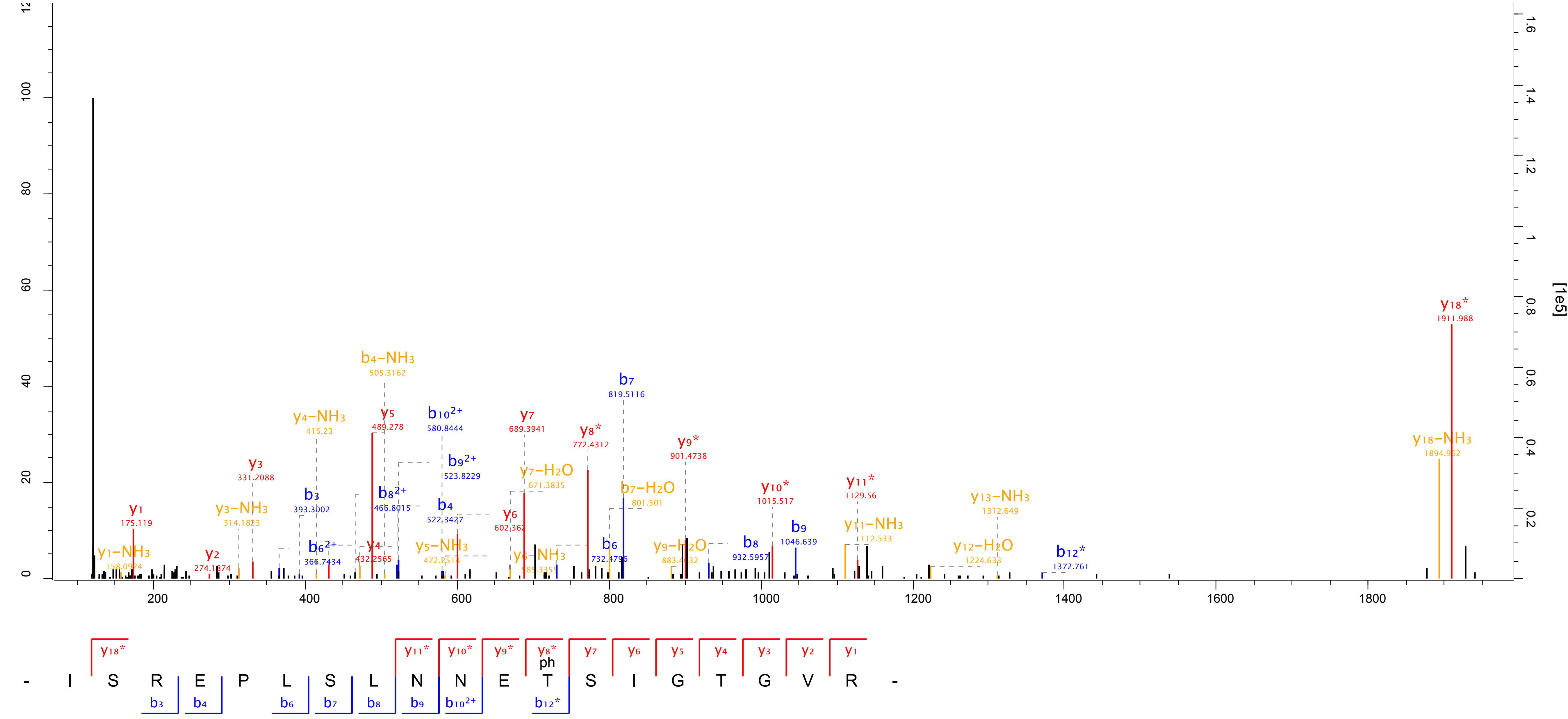

Raw file	Scan	Method	Score	m/z
20150122_PS_Syn_DimethylMix_Rep1_Phospho05	12252	FTMS; HCD	71.85	992.97

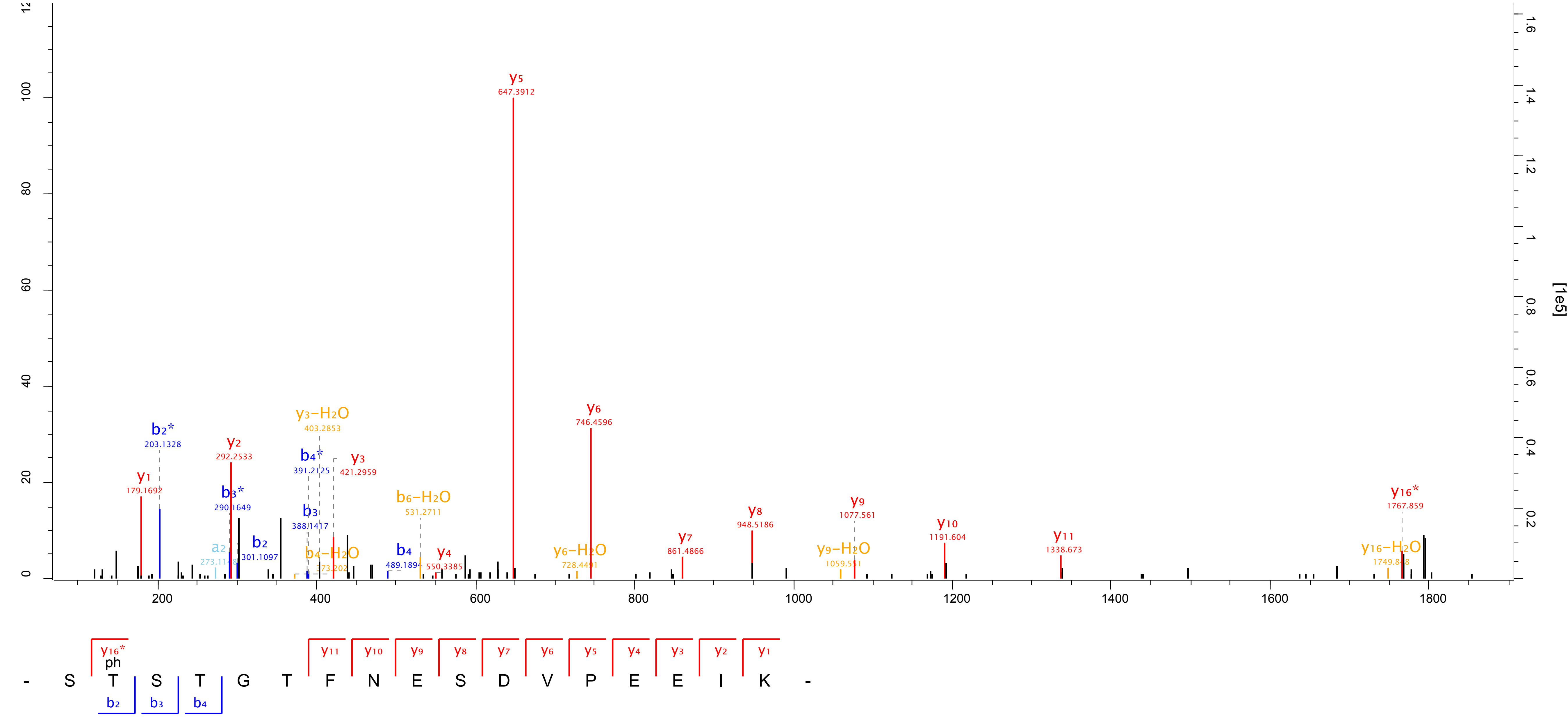

Raw file	Scan	Method	Score	m/z
201417_PS_Syn6803wt_dimethylMix_TiO2_1	12258	FTMS; HCD	95.21	728.34

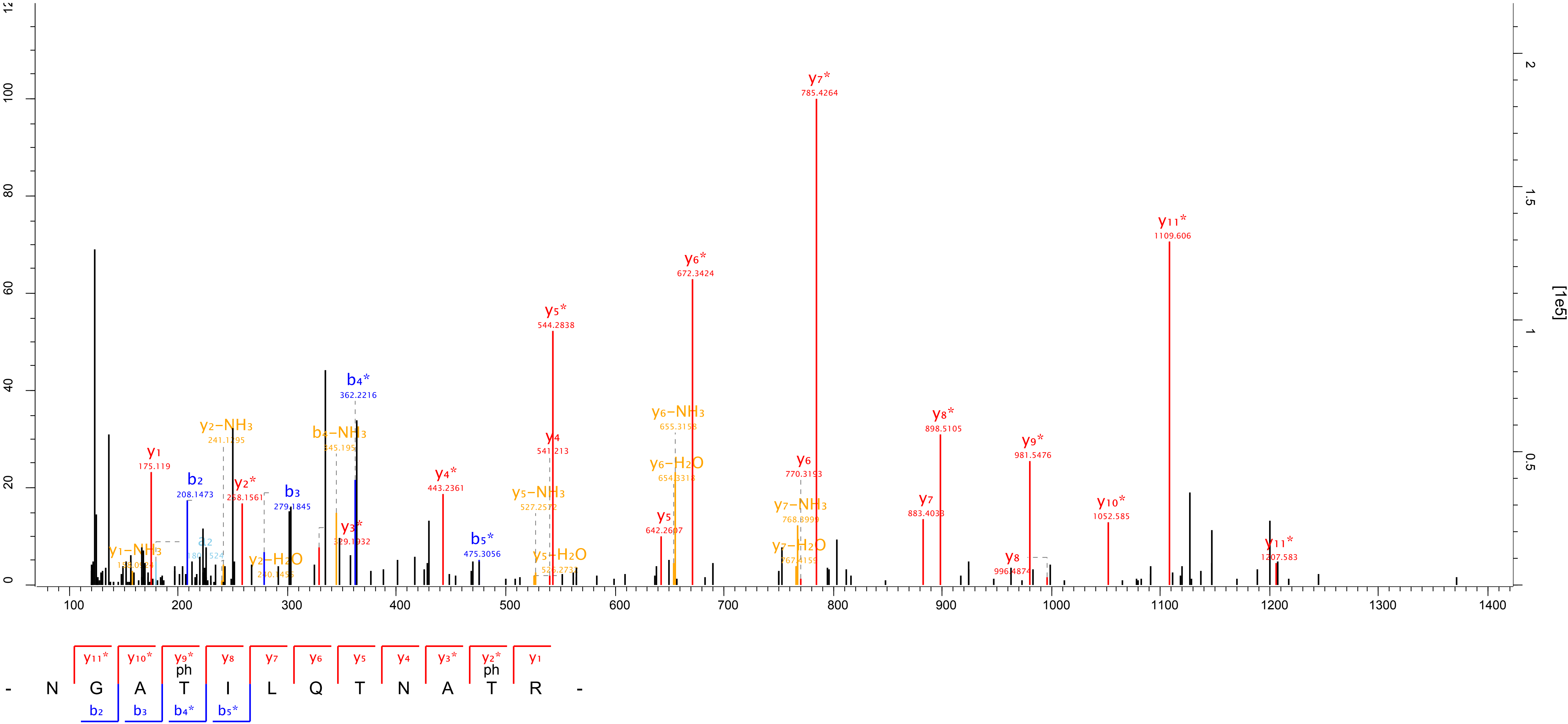

Raw file	Scan	Method	Score	m/z
20141031_PS_Syn6803wt_dimethyl_Mix_Phospho4	12390	FTMS; HCD	47.5	639.35

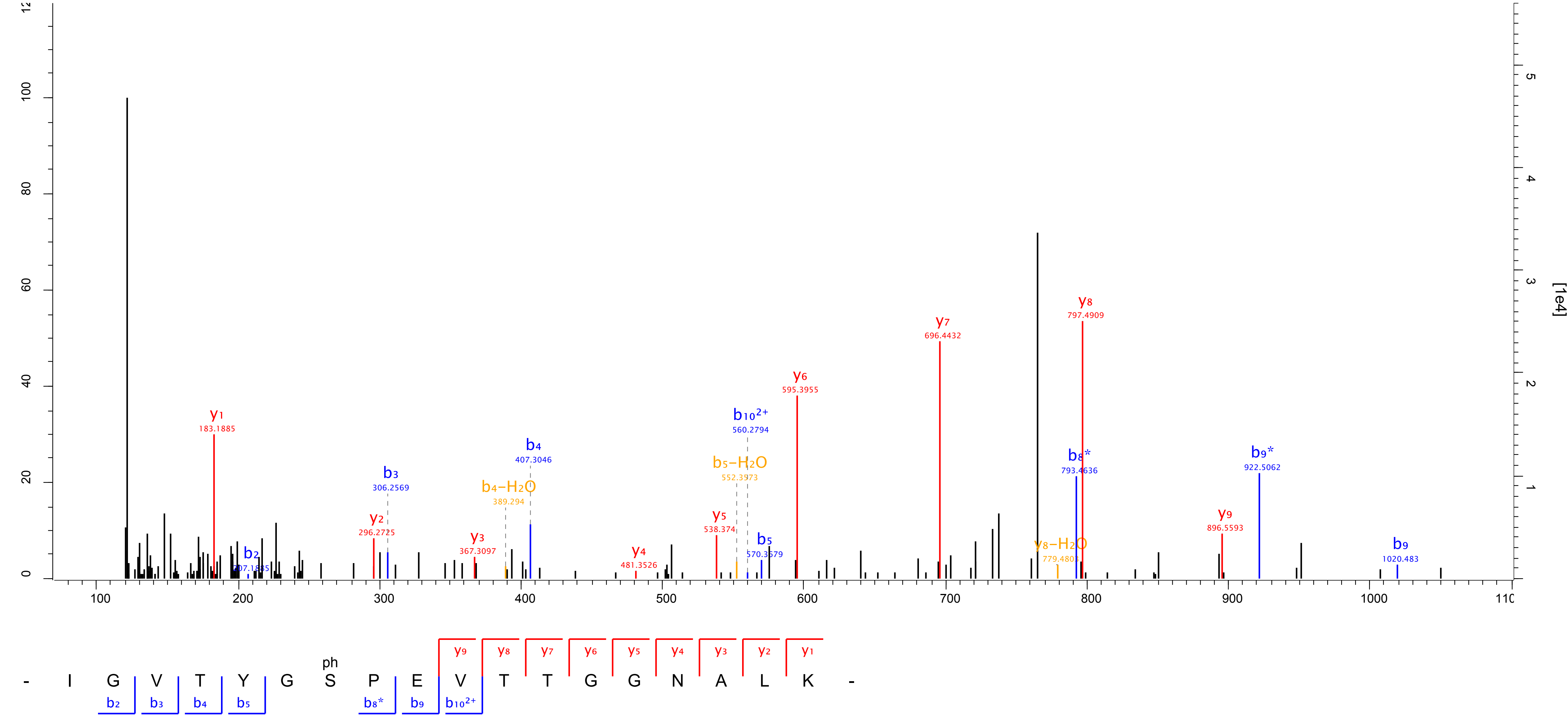

Raw file	Scan	Method	Score	m/z
20141031_PS_Syn6803wt_dimethyl_Mix_Phospho7	12446	FTMS; HCD	121.13	671.87

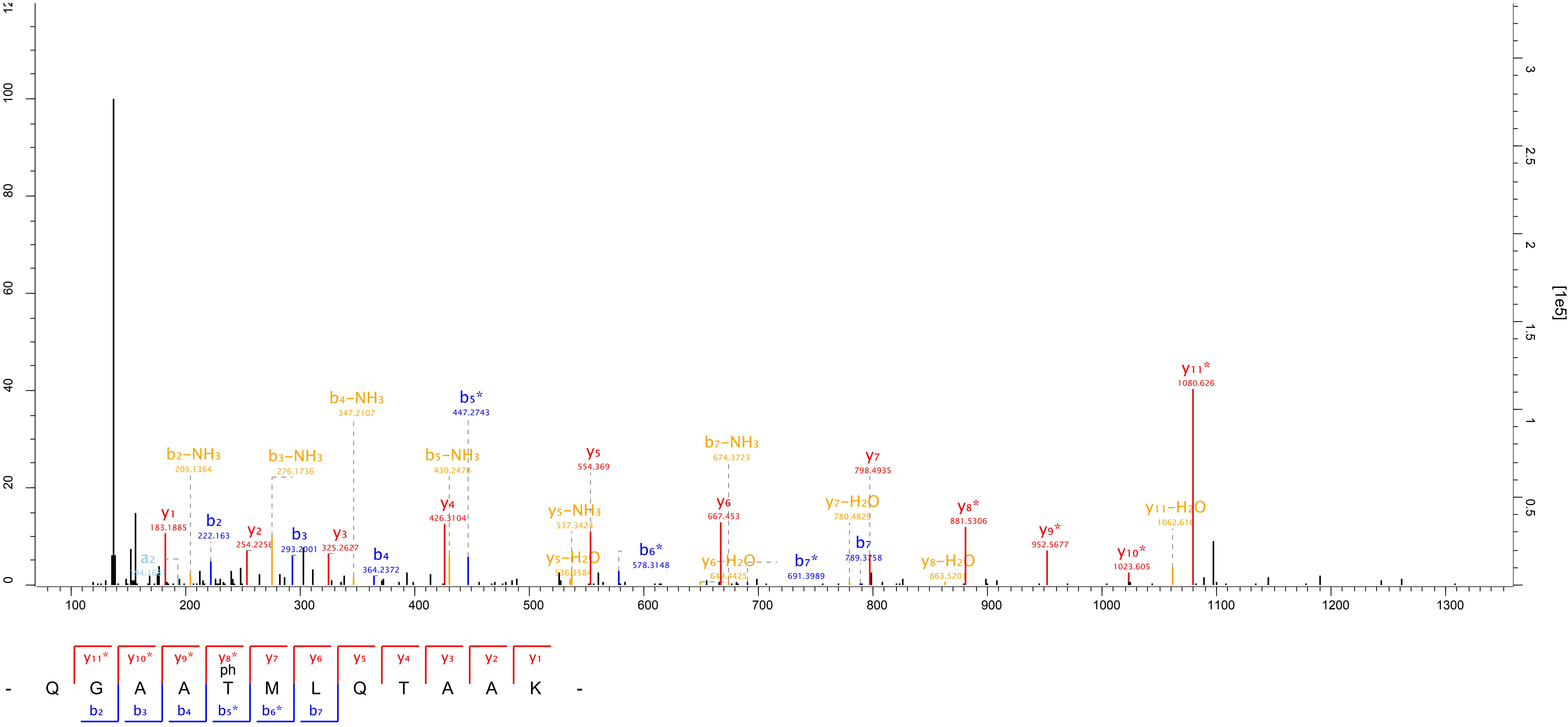

Raw file	Scan	Method	Score	m/z
20141031_PS_Syn6803wt_dimethyl_Mix_Phospho5	12571	FTMS; HCD	124.68	860.41

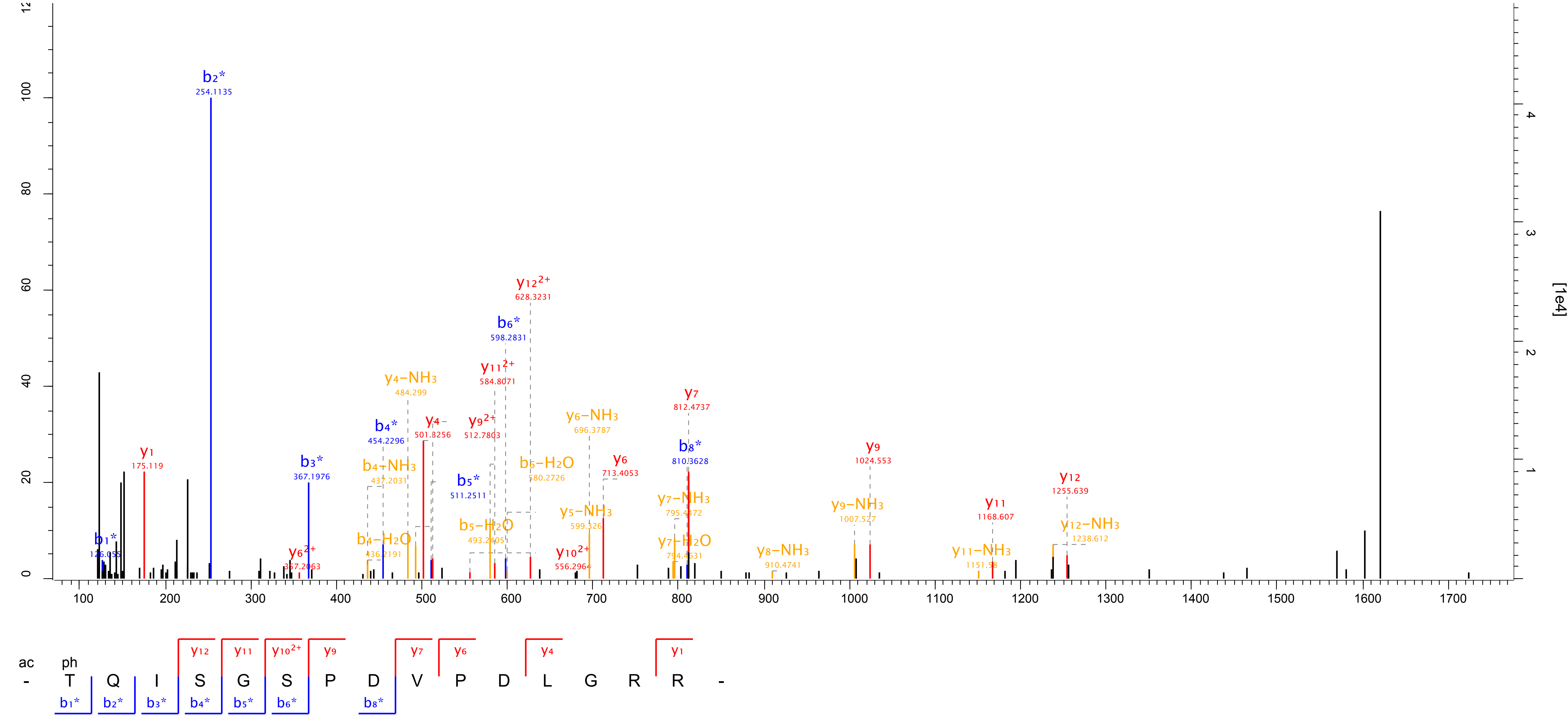

Raw file	Scan	Method	Score	m/z
20141031_PS_Syn6803wt_dimethyl_Mix_Phospho5	12601	FTMS; HCD	76.21	720.38

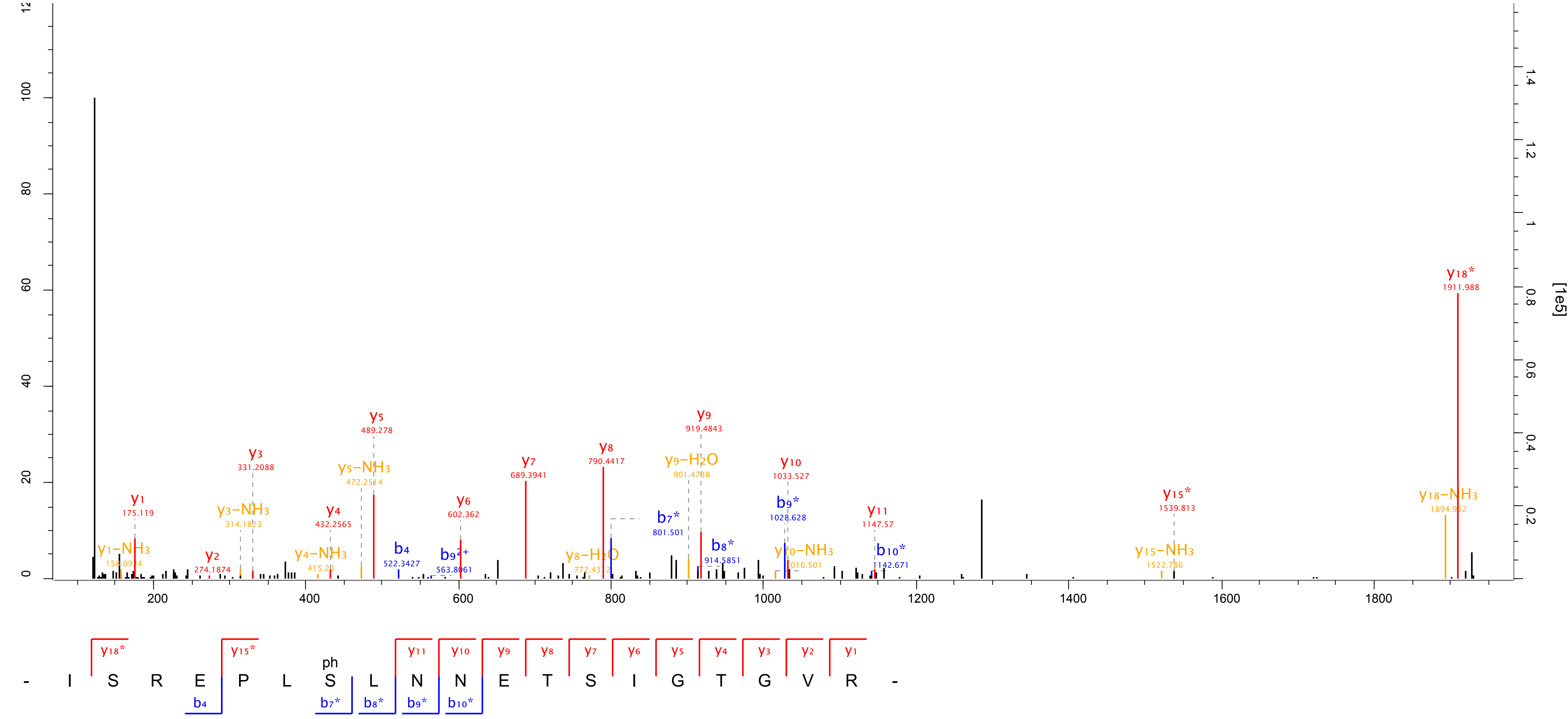

Raw file	Scan	Method	Score	m/z
20141031_PS_Syn6803wt_dimethyl_Mix_Phospho1	13563	FTMS; HCD	154.34	997.49

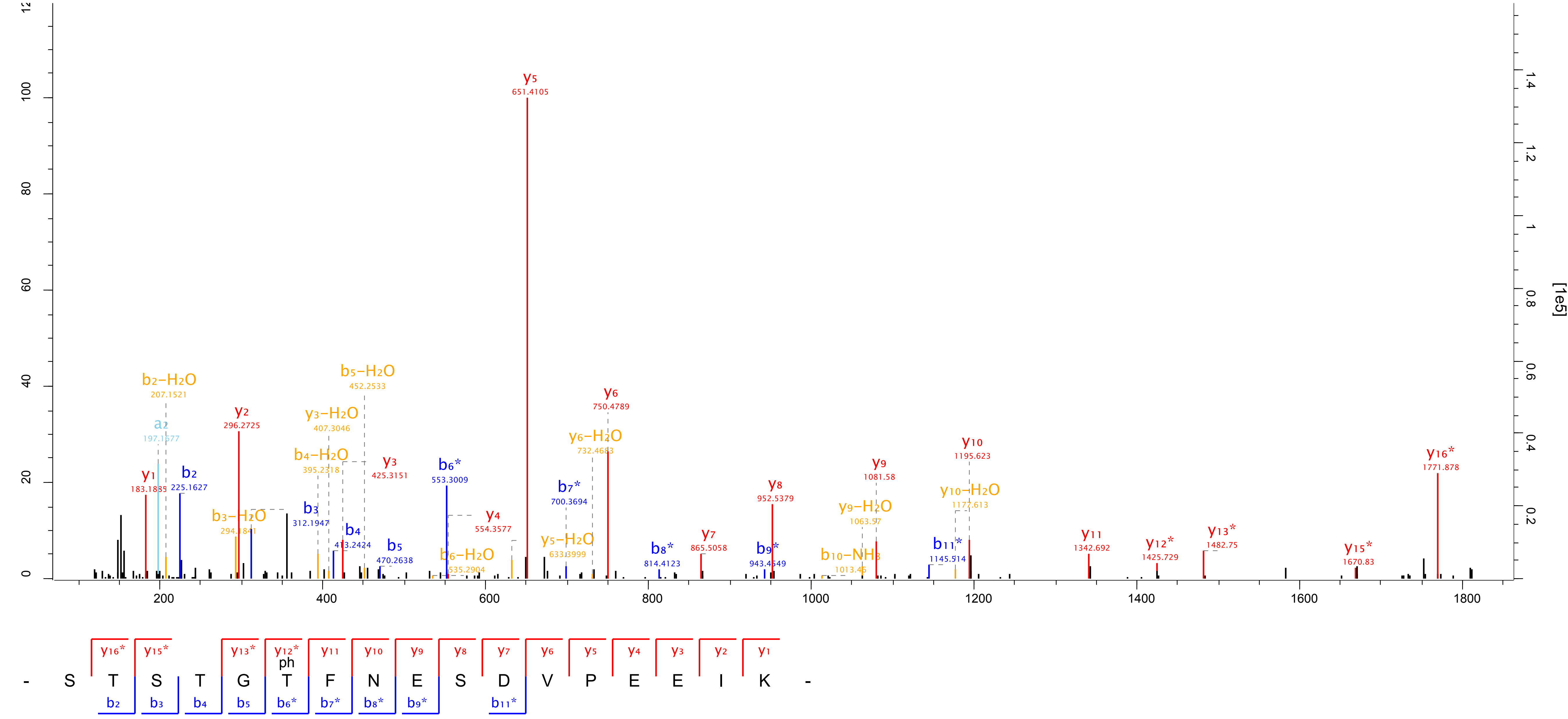

Raw file	Scan	Method	Score	m/z
20141031_PS_Syn6803wt_dimethyl_Mix_Phospho1	13840	FTMS; HCD	100.28	996.98

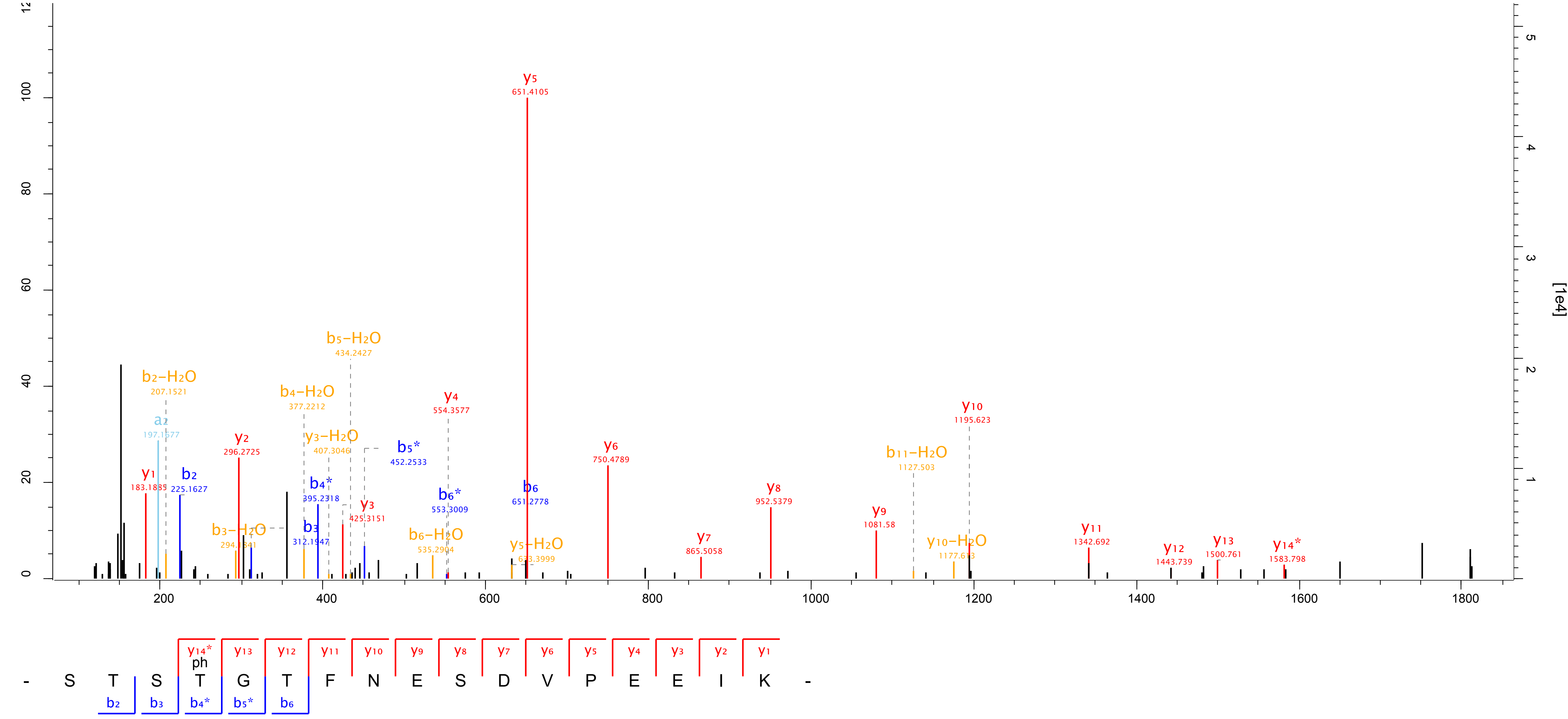

Raw file	Scan	Method	Score	m/z
20141031_PS_Syn6803wt_dimethyl_Mix_Phospho1	13851	FTMS; HCD	92.79	758.4

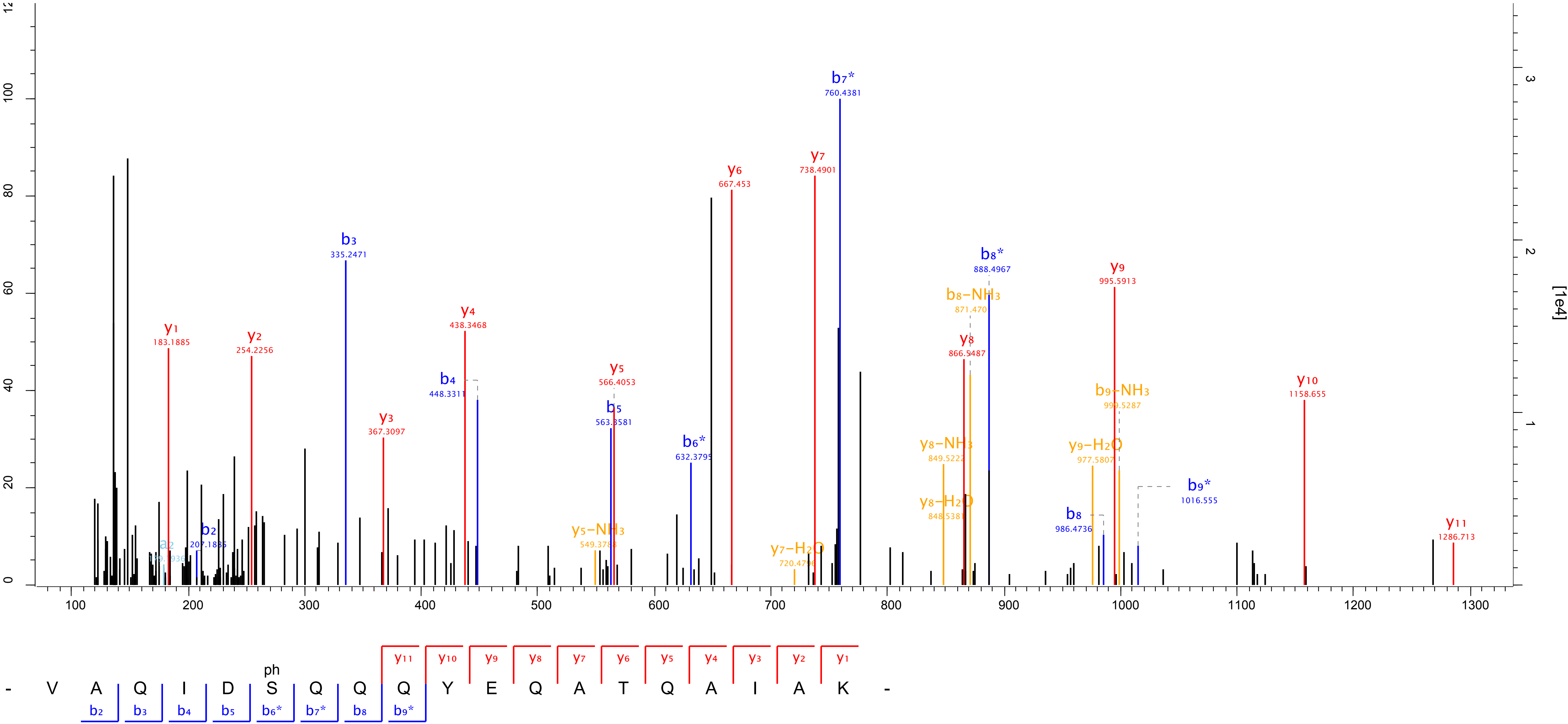

Raw file	Scan	Method	Score	m/z
20141031_PS_Syn6803wt_dimethyl_Mix_Phospho1	13854	FTMS; HCD	110.78	953.46

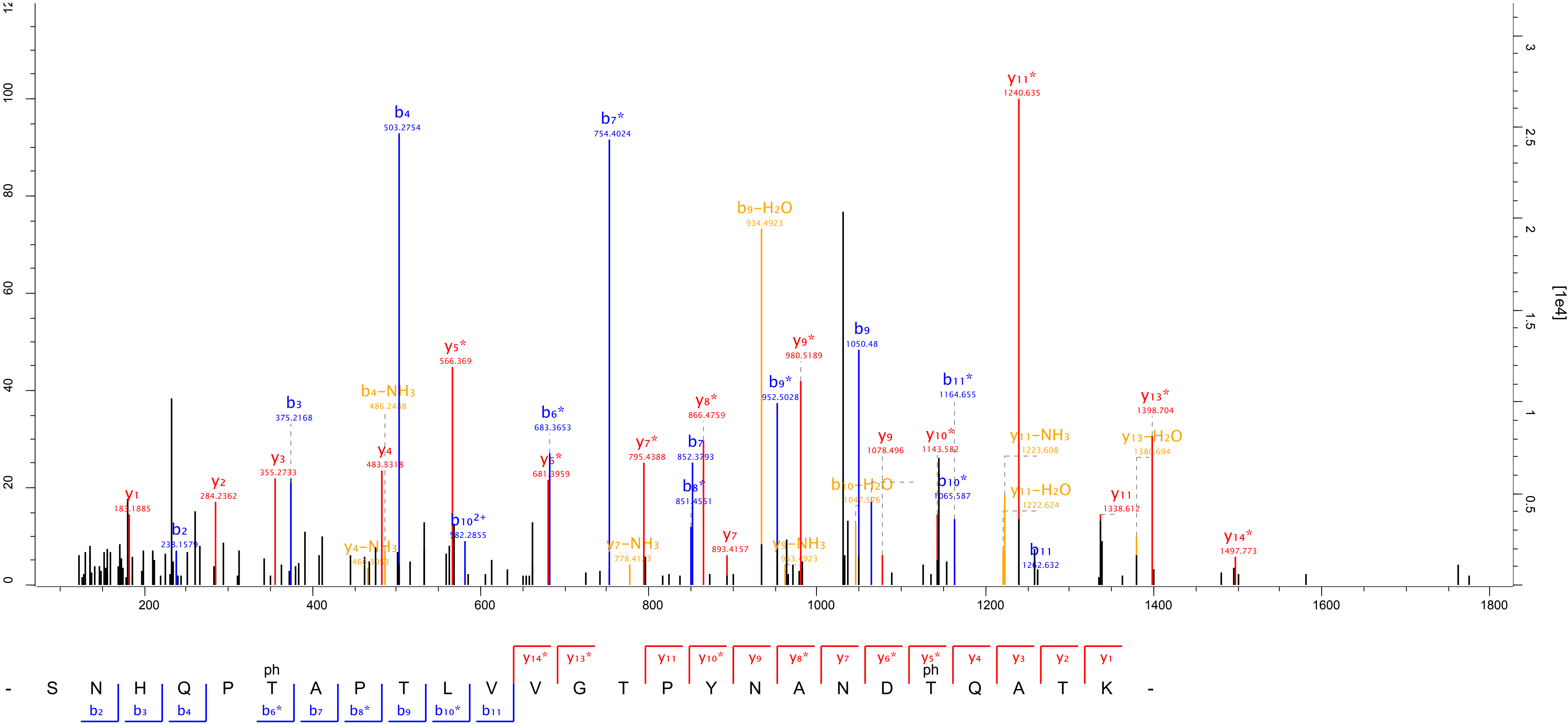

Raw file	Scan	Method	Score	m/z
201417_PS_Syn6803wt_dimethylMix_TiO2_2	13920	FTMS; HCD	77.07	713.38

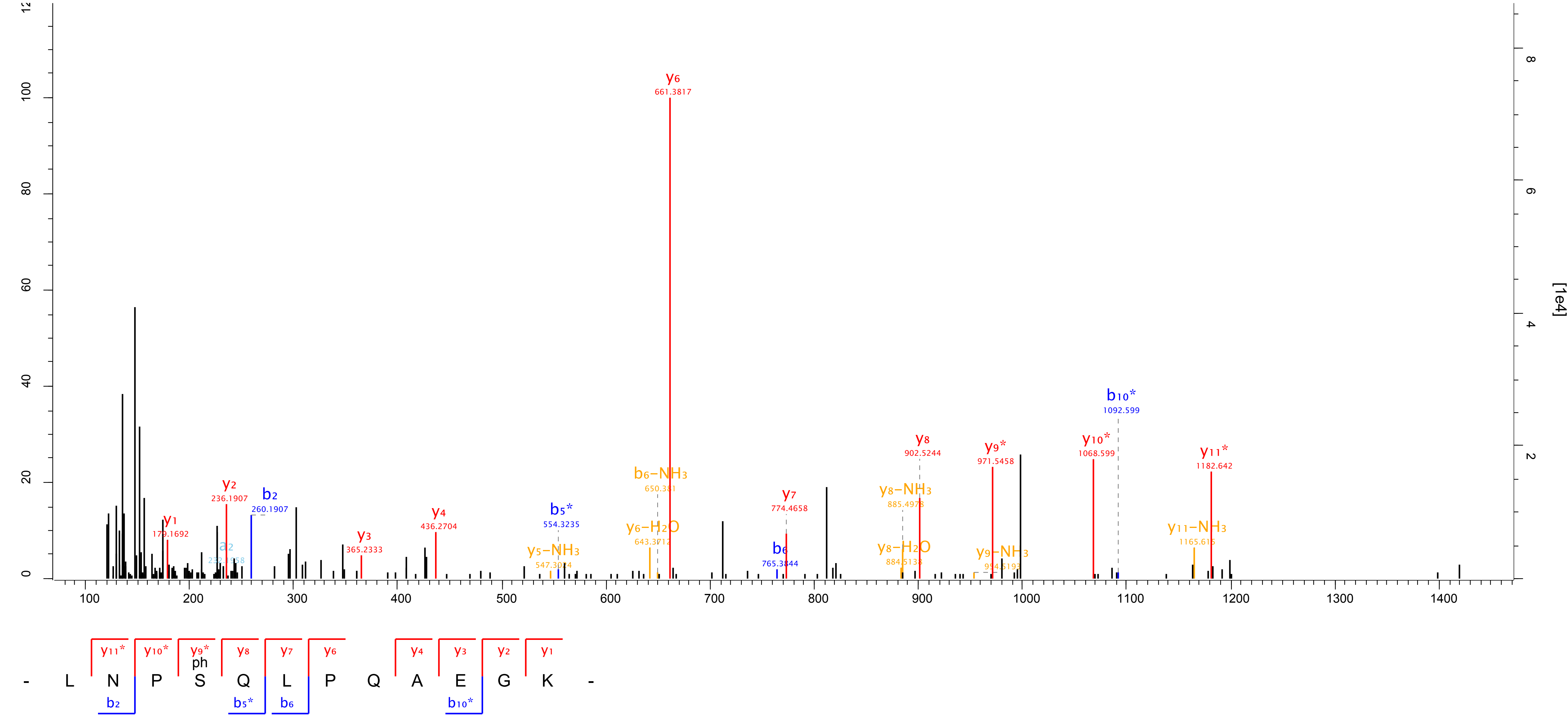

Raw file	Scan	Method	Score	m/z
201417_PS_Syn6803wt_dimethylMix_TiO2_1	14224	FTMS; HCD	97.2	778.86

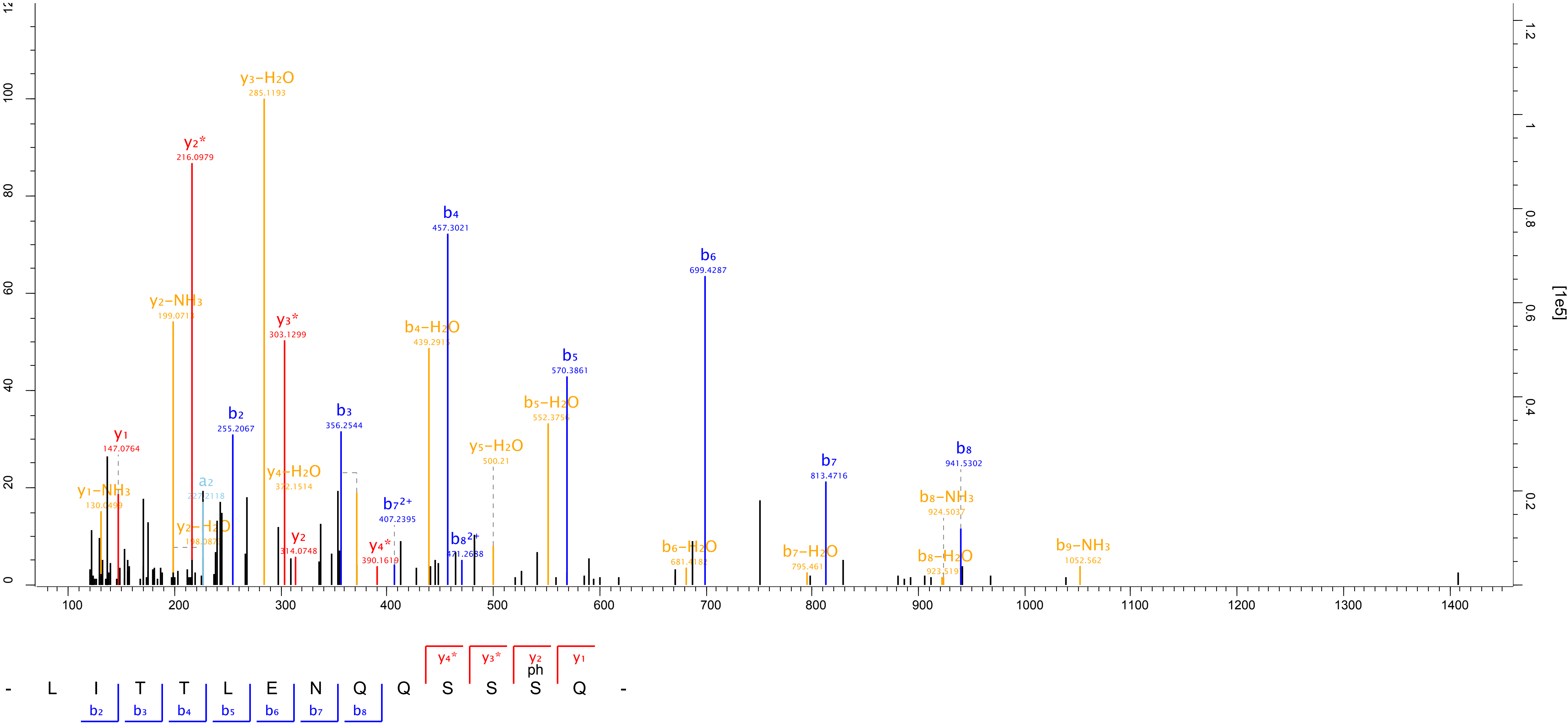

Raw file	Scan	Method	Score	m/z
20141031_PS_Syn6803wt_dimethyl_Mix_Phospho5	14336	FTMS; HCD	56.71	693.02

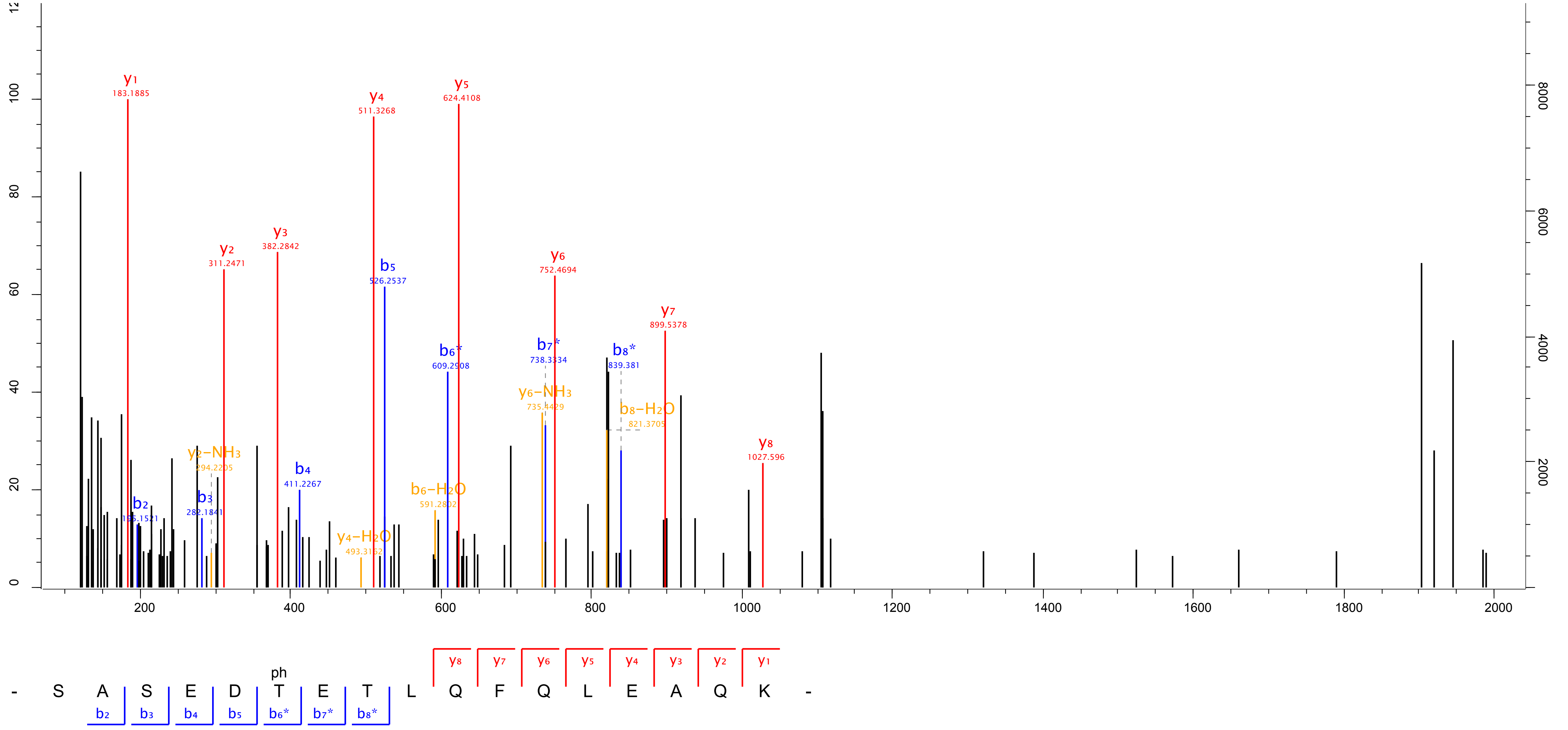

Raw file	Scan	Method	Score	m/z
20141031_PS_Syn6803wt_dimethyl_Mix_Phospho7	14710	FTMS; HCD	66.25	491.97

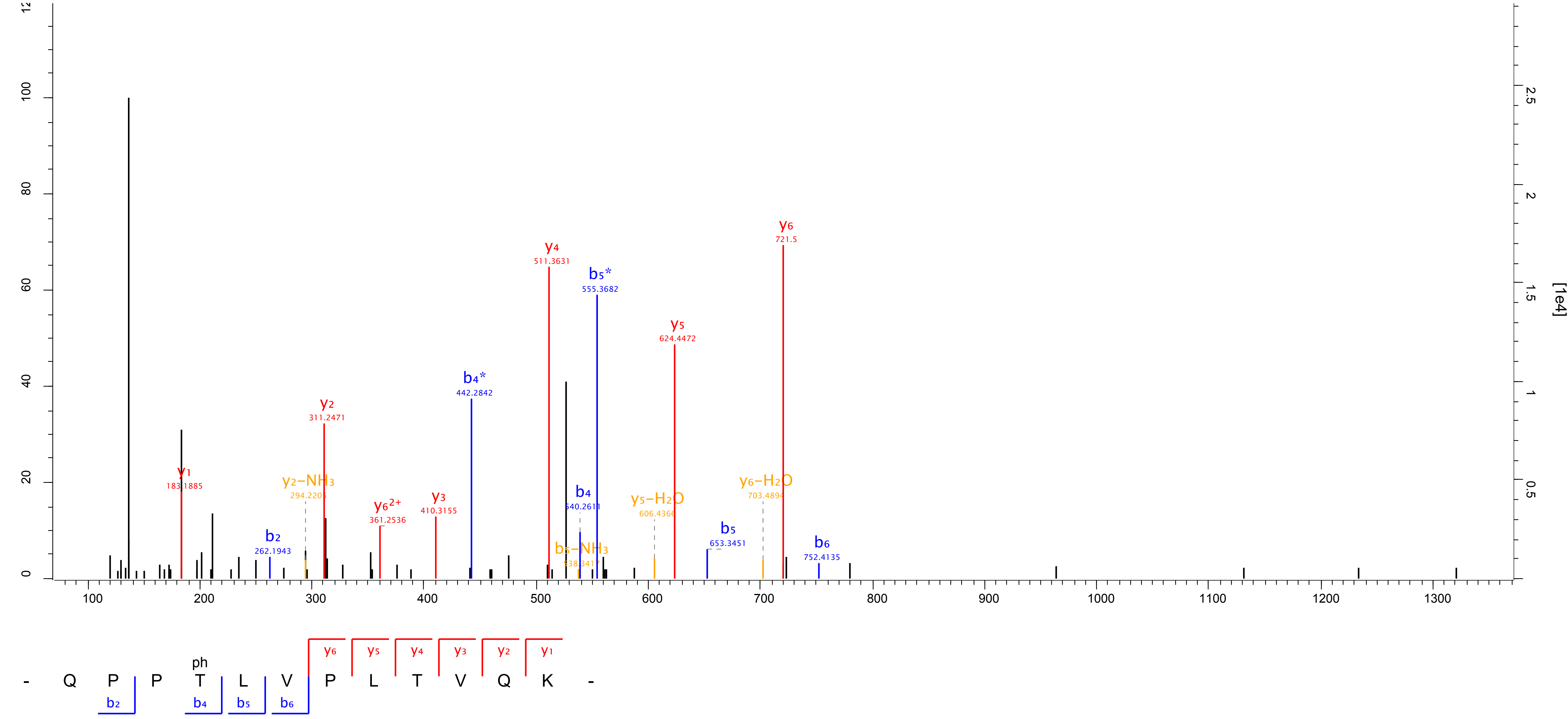

Raw file	Scan	Method	Score	m/z
20141031_PS_Syn6803wt_dimethyl_Mix_Phospho3	14721	FTMS; HCD	48.6	728.4

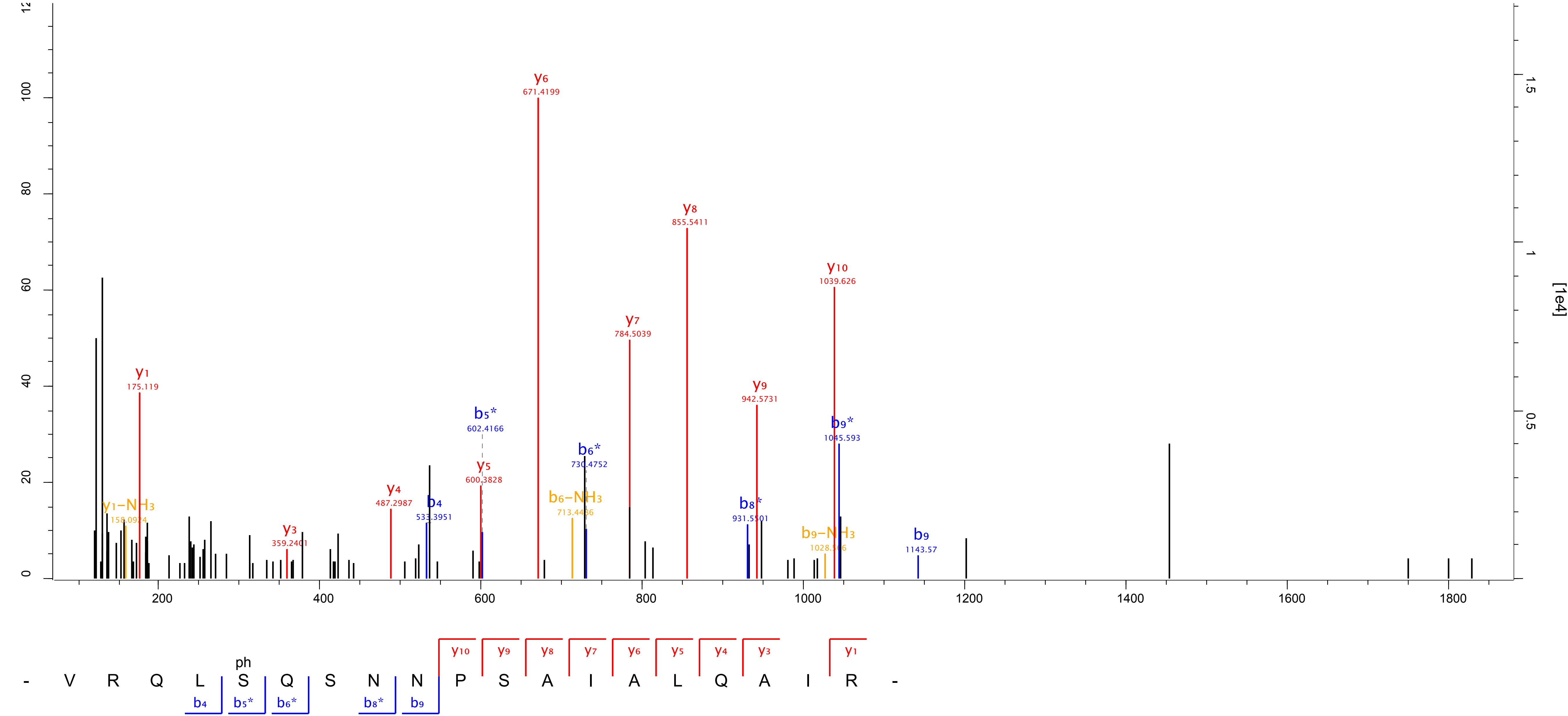

Raw file	Scan	Method	Score	m/z
20141031_PS_Syn6803wt_dimethyl_Mix_Phospho4	14741	FTMS; HCD	70.09	707.67

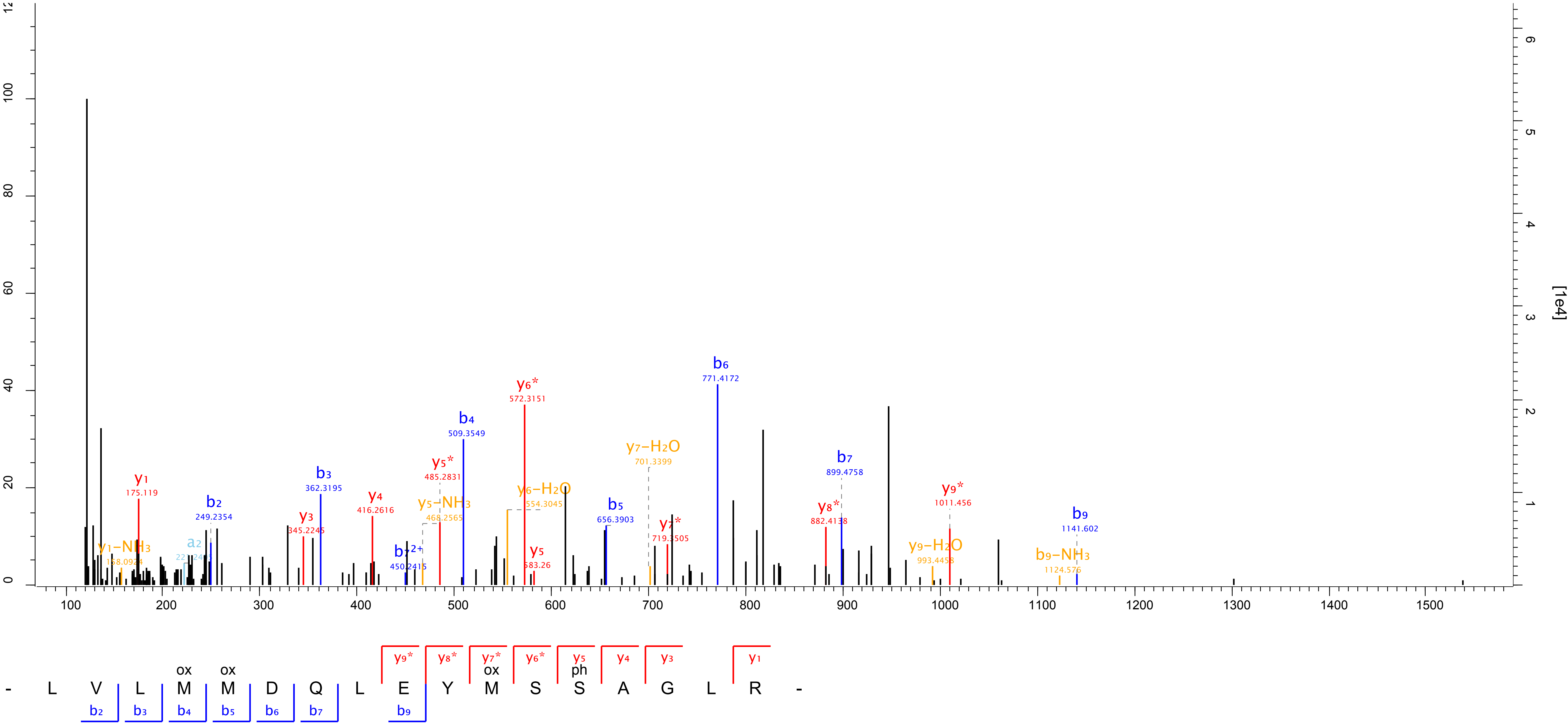

Raw file	Scan	Method	Score	m/z
20141031_PS_Syn6803wt_dimethyl_Mix_Phospho3	14758	FTMS; HCD	123.02	819.4

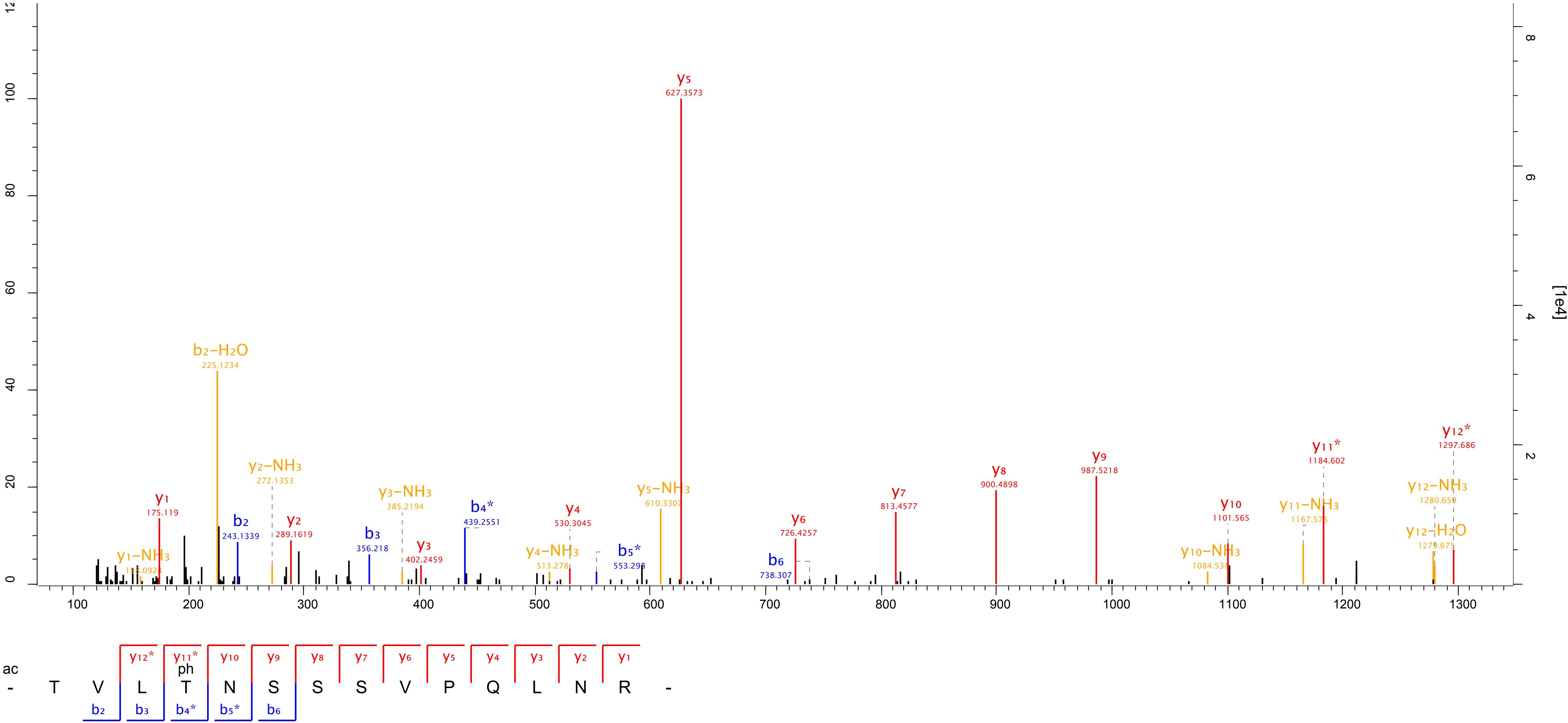

Raw file	Scan	Method	Score	m/z
20141031_PS_Syn6803wt_dimethyl_Mix_Phospho4	14883	FTMS; HCD	75.59	967.08

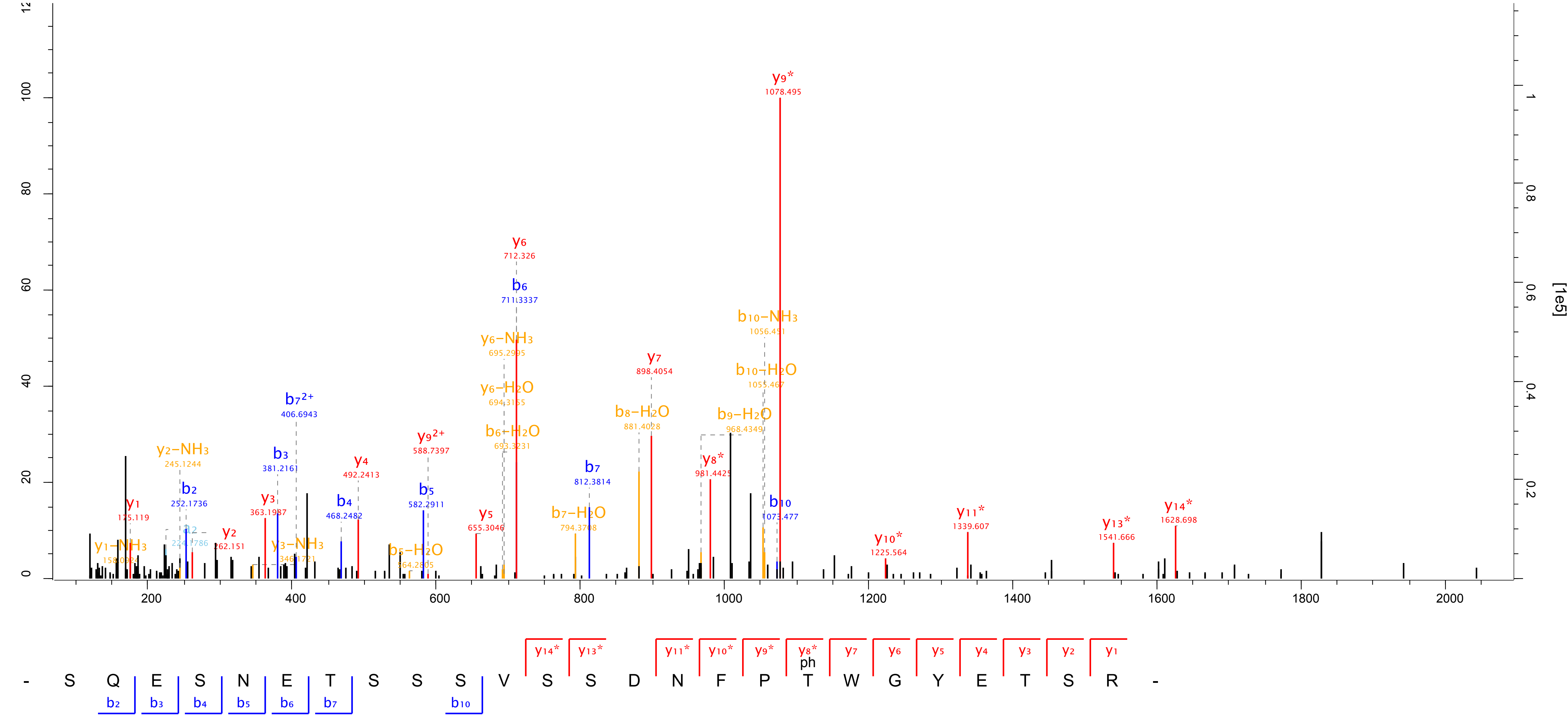

Raw file	Scan	Method	Score	m/z
201417_PS_Syn6803wt_dimethylMix_TiO2_2	15098	FTMS; HCD	110.6	924.13

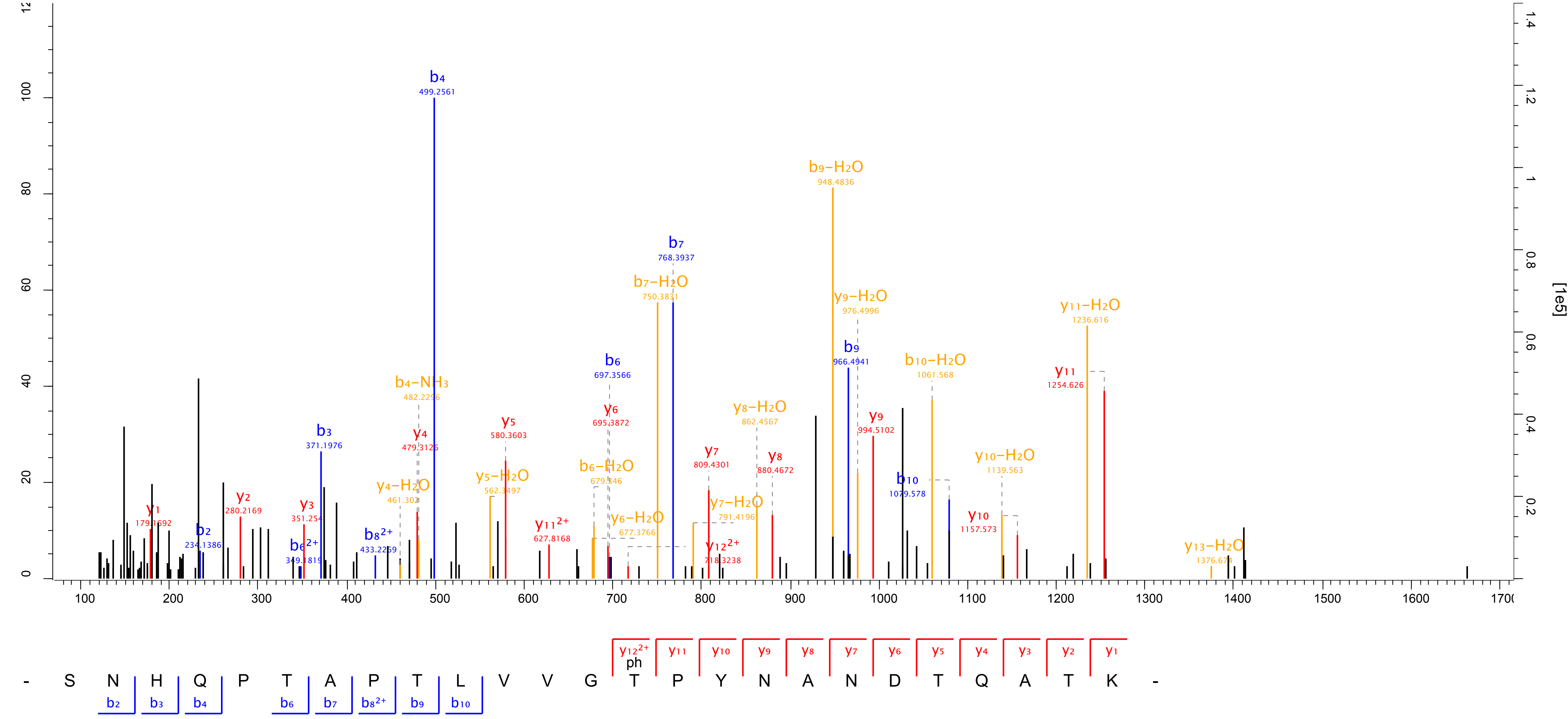

Raw file	Scan	Method	Score	m/z
20150122_PS_Syn_DimethylMix_Rep1_Phospho01	15408	FTMS; HCD	72.43	819.4

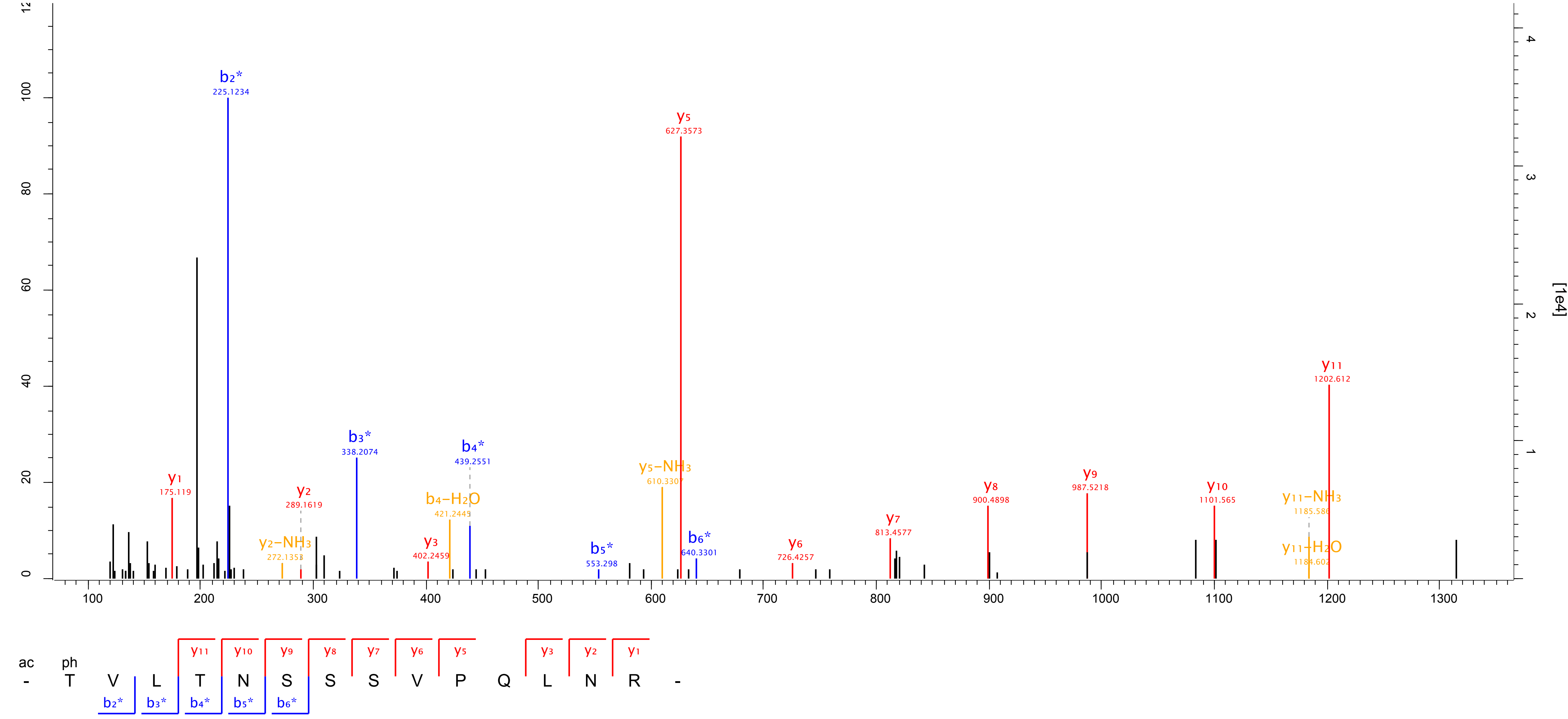

Raw file	Scan	Method	Score	m/z
20141031_PS_Syn6803wt_dimethyl_Mix_Phospho1	15647	FTMS; HCD	59.91	819.39

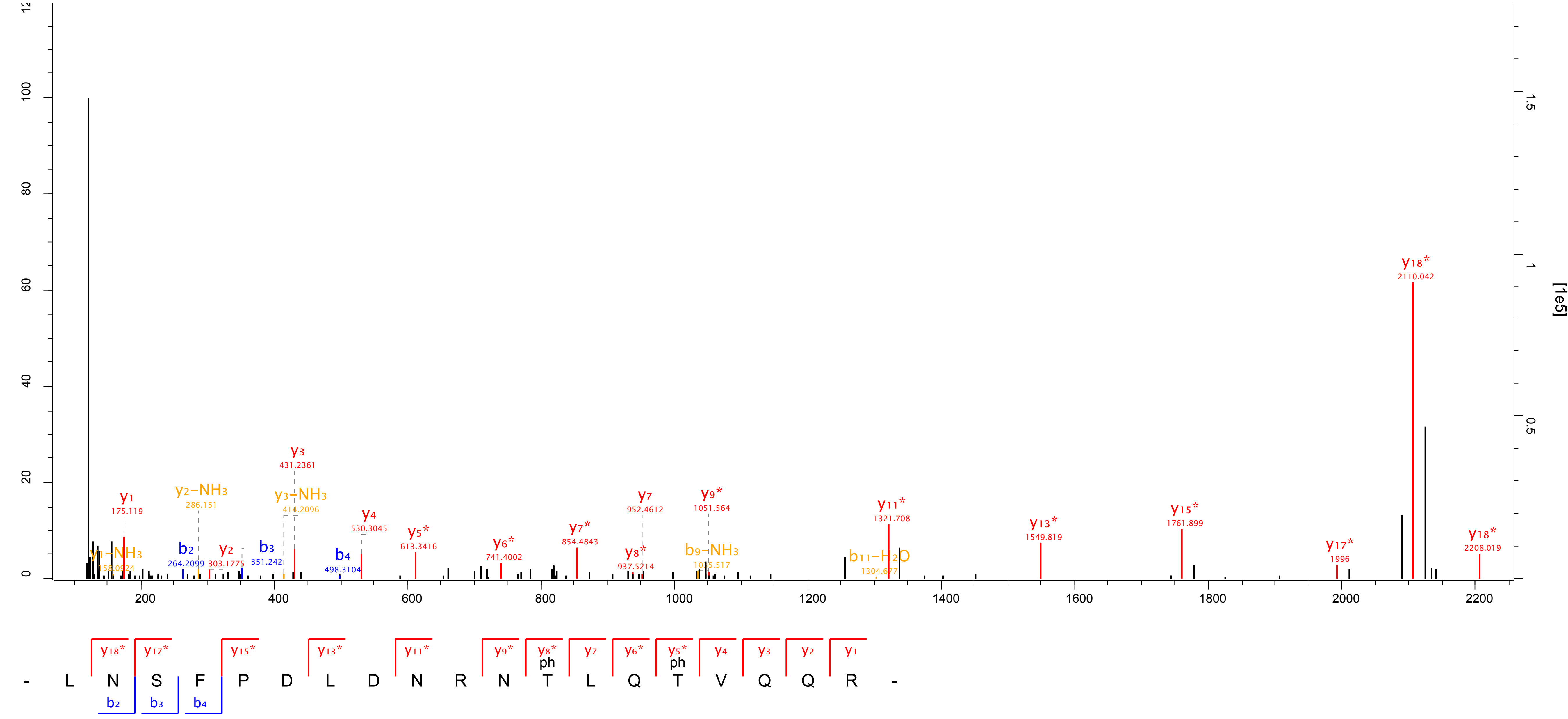

Raw file	Scan	Method	Score	m/z
20141031_PS_Syn6803wt_dimethyl_Mix_Phospho4	15778	FTMS; HCD	143.29	1194.62

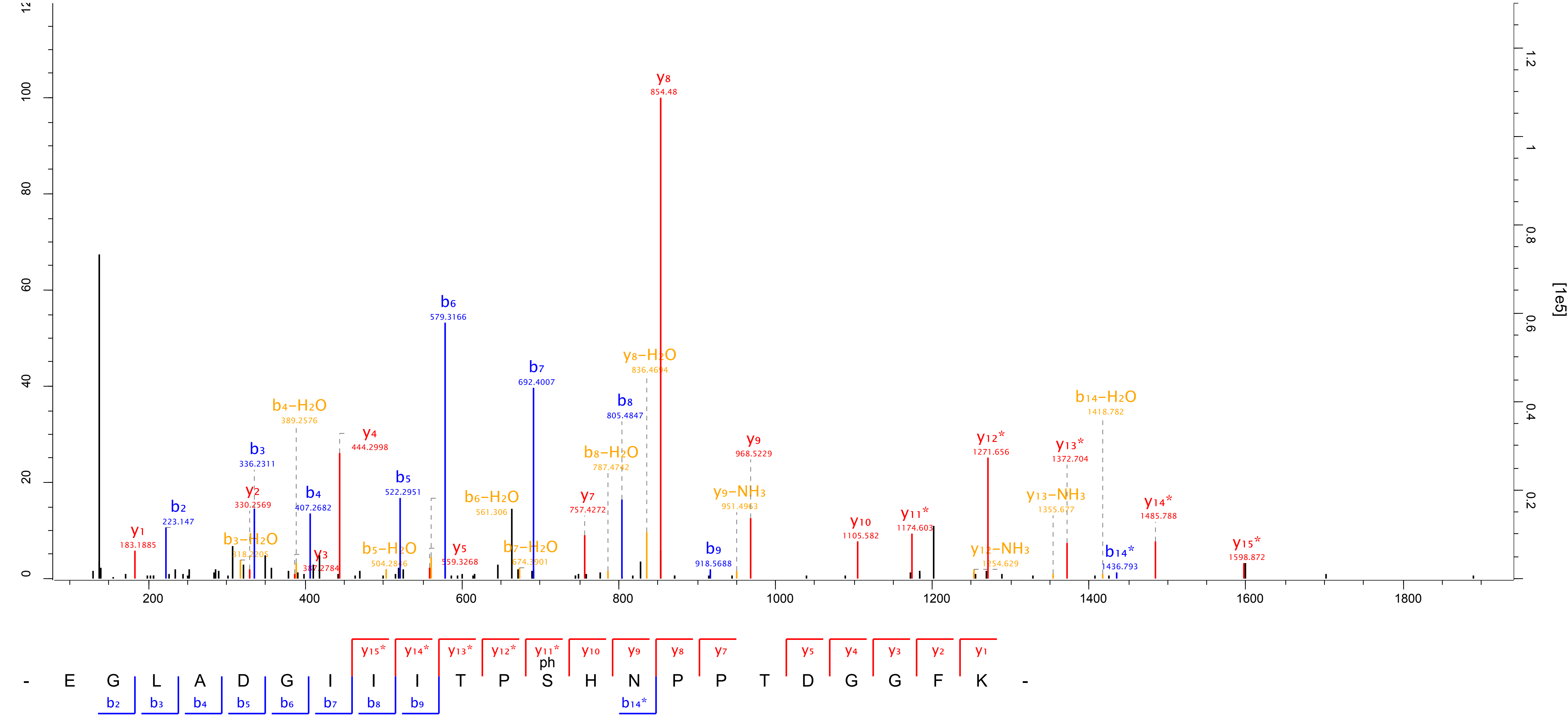

Raw file	Scan	Method	Score	m/z
20141031_PS_Syn6803wt_dimethyl_Mix_Phospho5	15781	FTMS; HCD	109.65	714.03

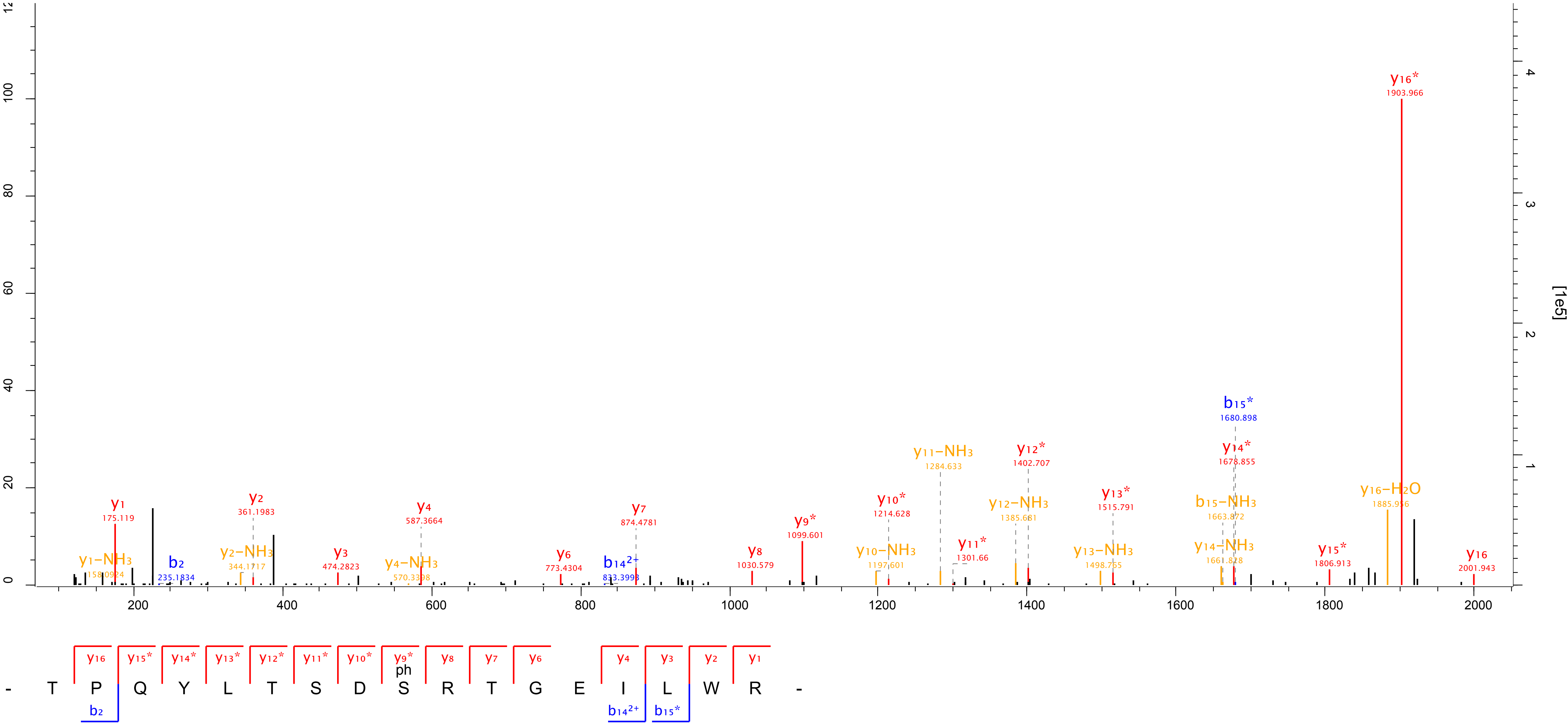

Raw file	Scan	Method	Score	m/z
20141031_PS_Syn6803wt_dimethyl_Mix_Phospho1	16061	FTMS; HCD	47.22	736.03

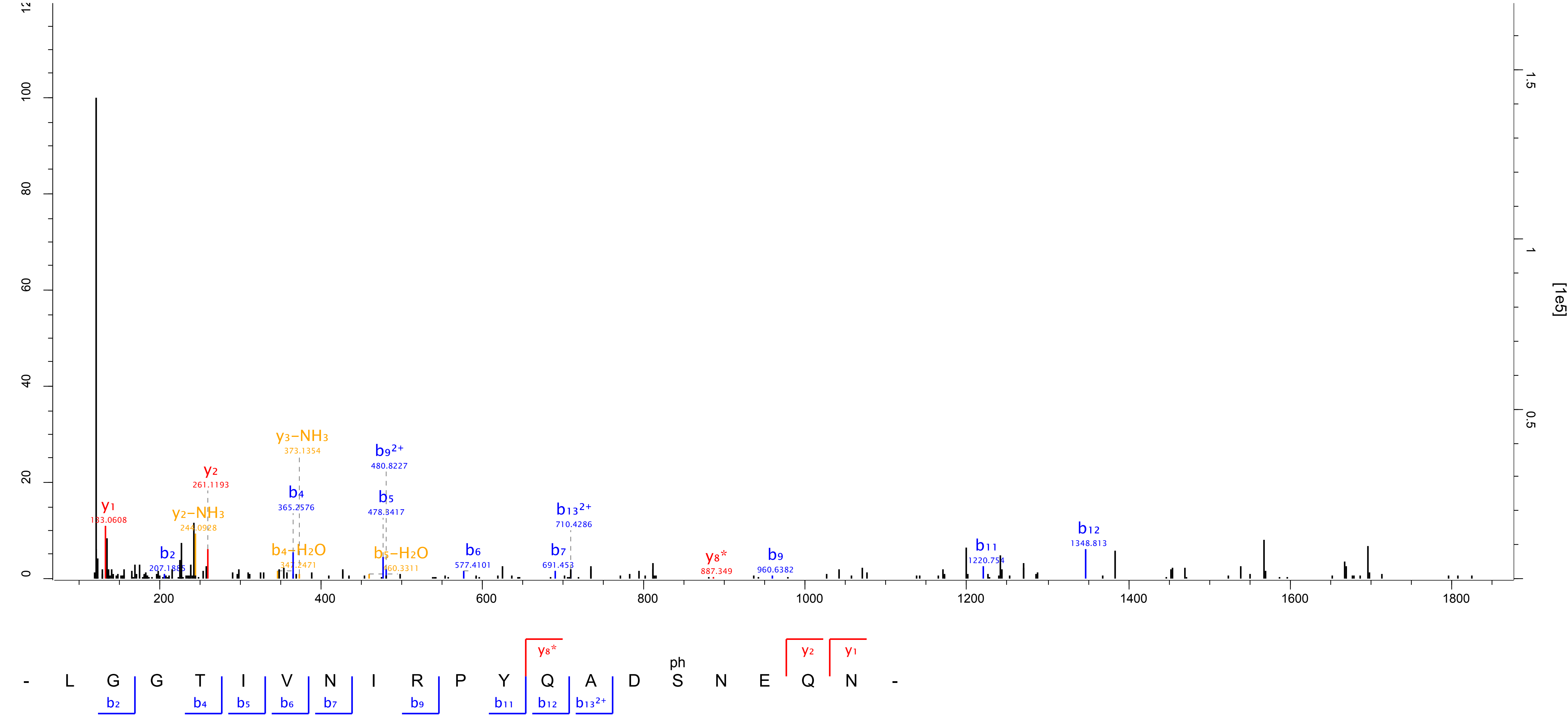

Raw file	Scan	Method	Score	m/z
20150122_PS_Syn_DimethylMix_Rep2_Phospho05	16118	FTMS; HCD	70.76	794.41

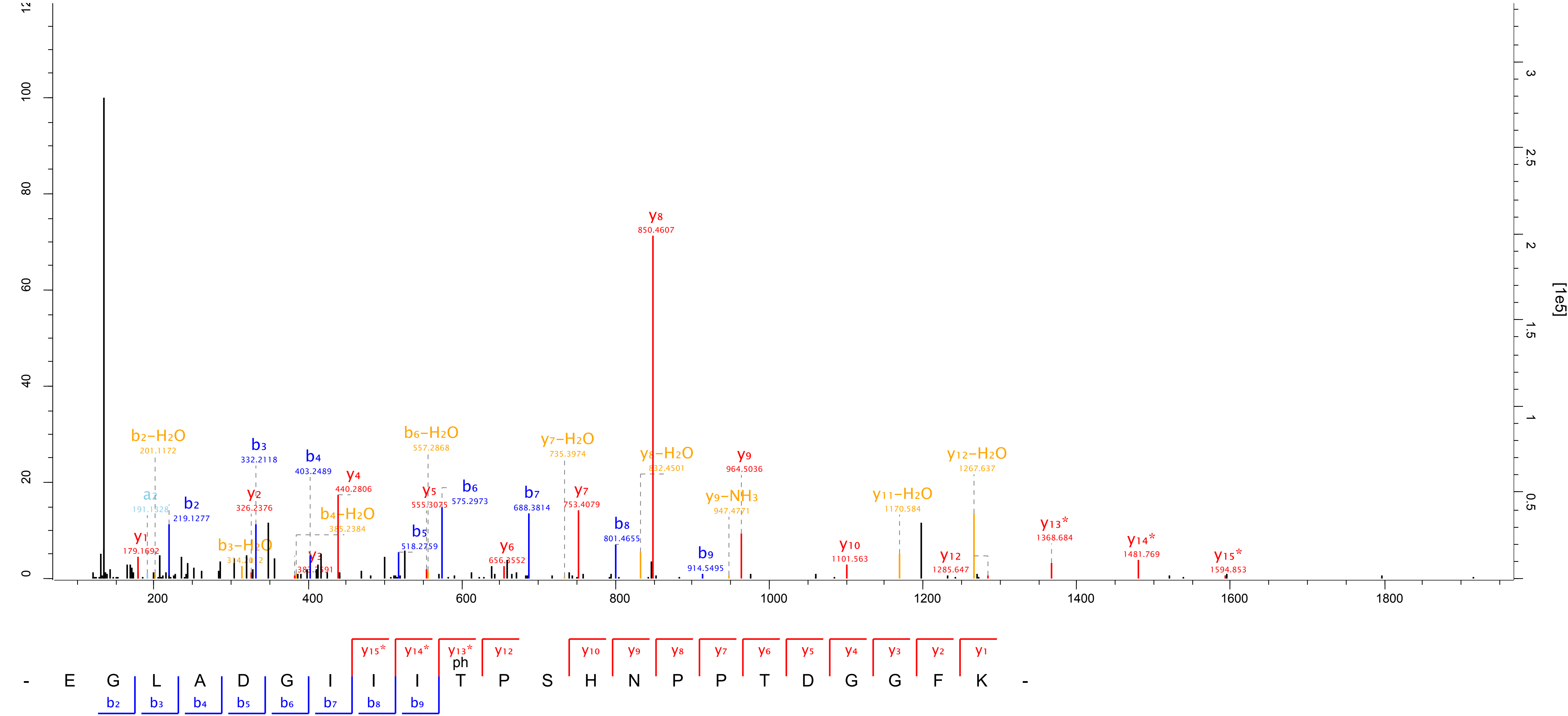

Raw file	Scan	Method	Score	m/z
201417_PS_Syn6803wt_dimethylMix_TiO2_1	16359	FTMS; HCD	78.08	948.1

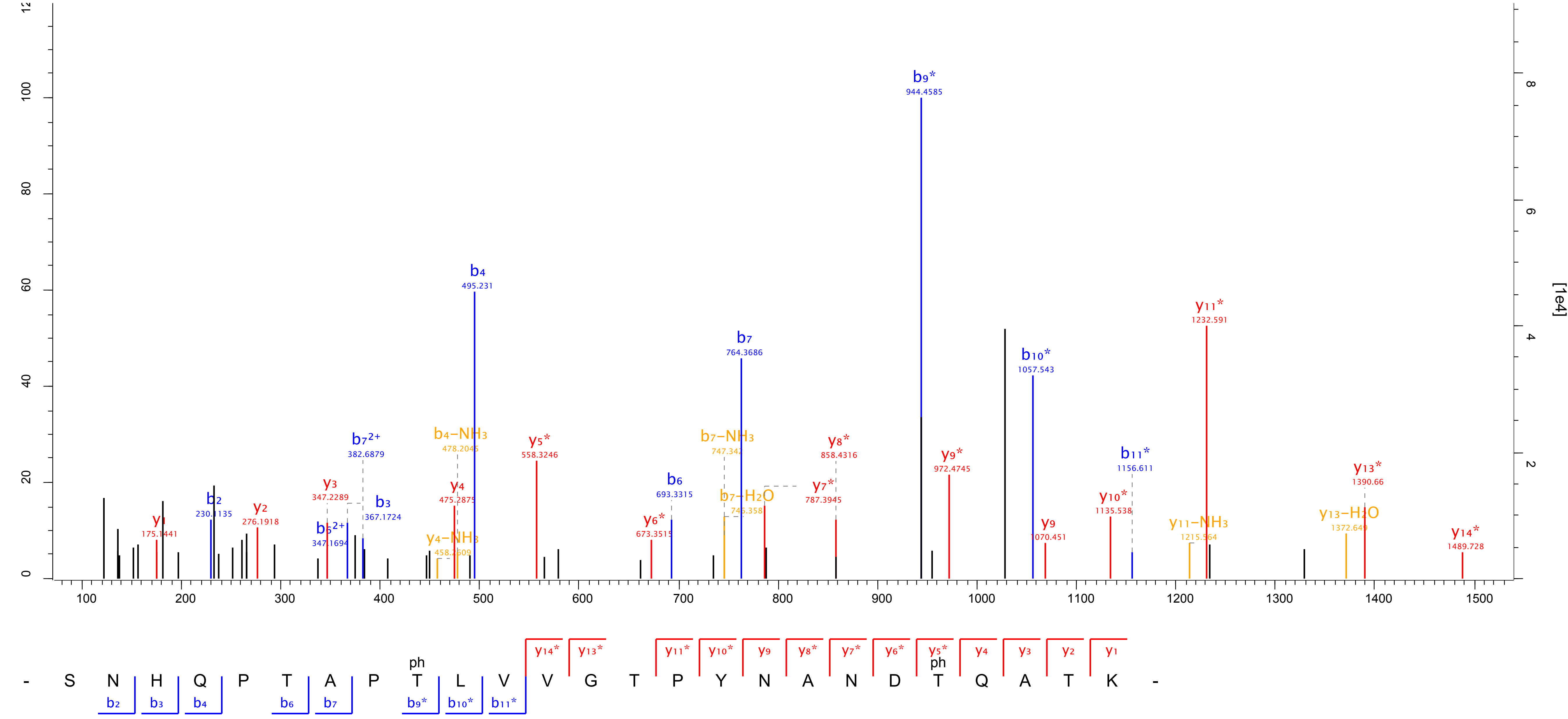

Raw file	Scan	Method	Score	m/z
20150122_PS_Syn_DimethylMix_Rep1_Phospho04	16538	FTMS; HCD	92.01	1071.54

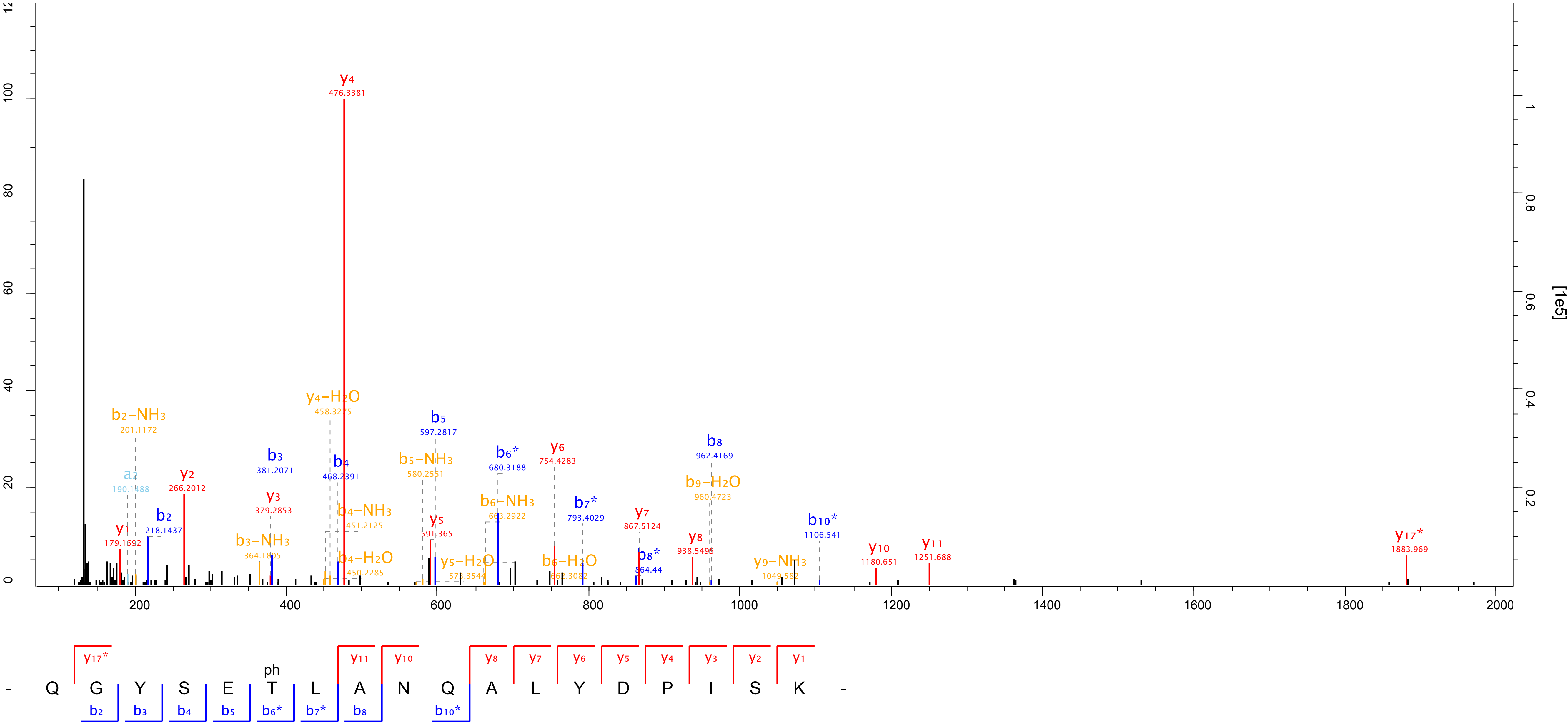

Raw file	Scan	Method	Score	m/z
20141031_PS_Syn6803wt_dimethyl_Mix_Phospho1	16570	FTMS; HCD	95.57	533.97

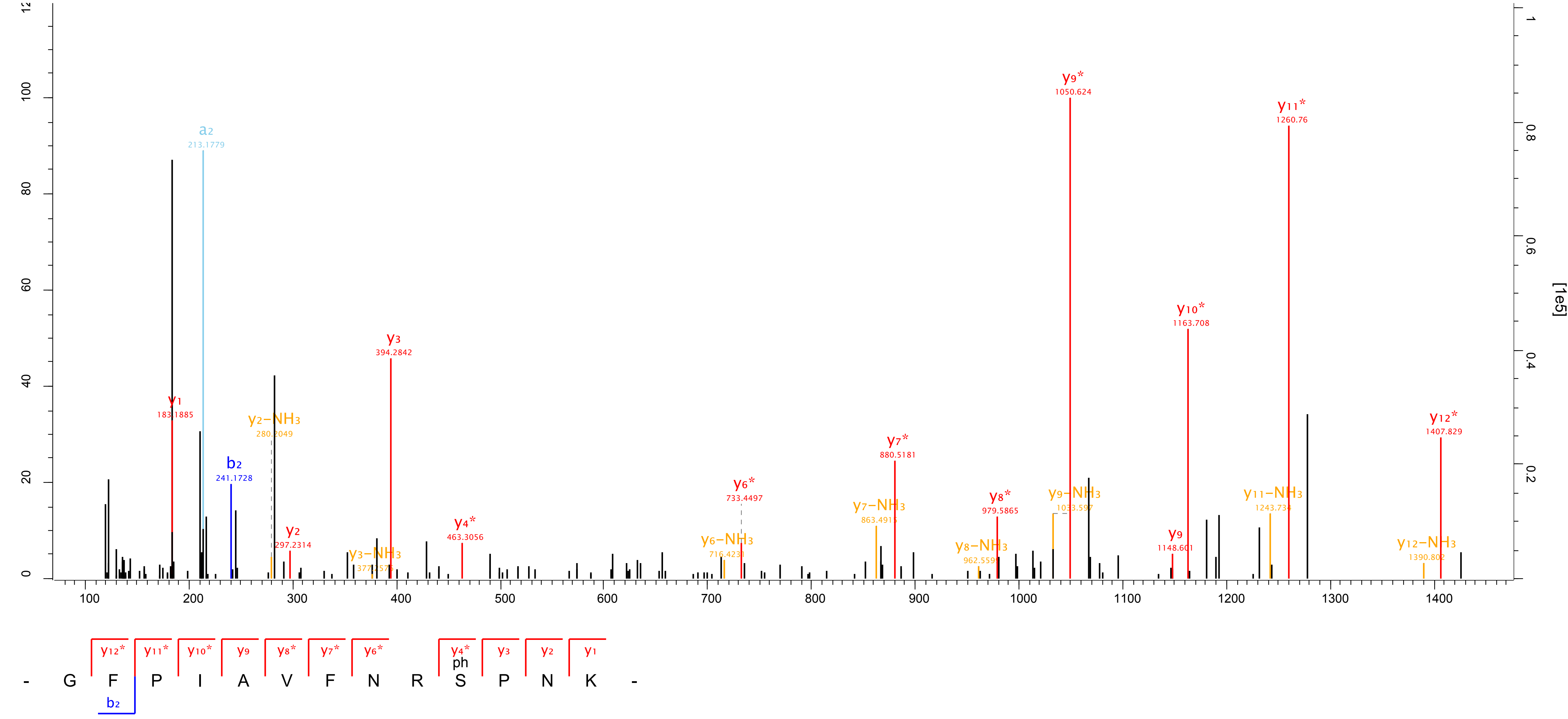

Raw file	Scan	Method	Score	m/z
20141031_PS_Syn6803wt_dimethyl_Mix_Phospho4	16582	FTMS; HCD	102.89	636.34

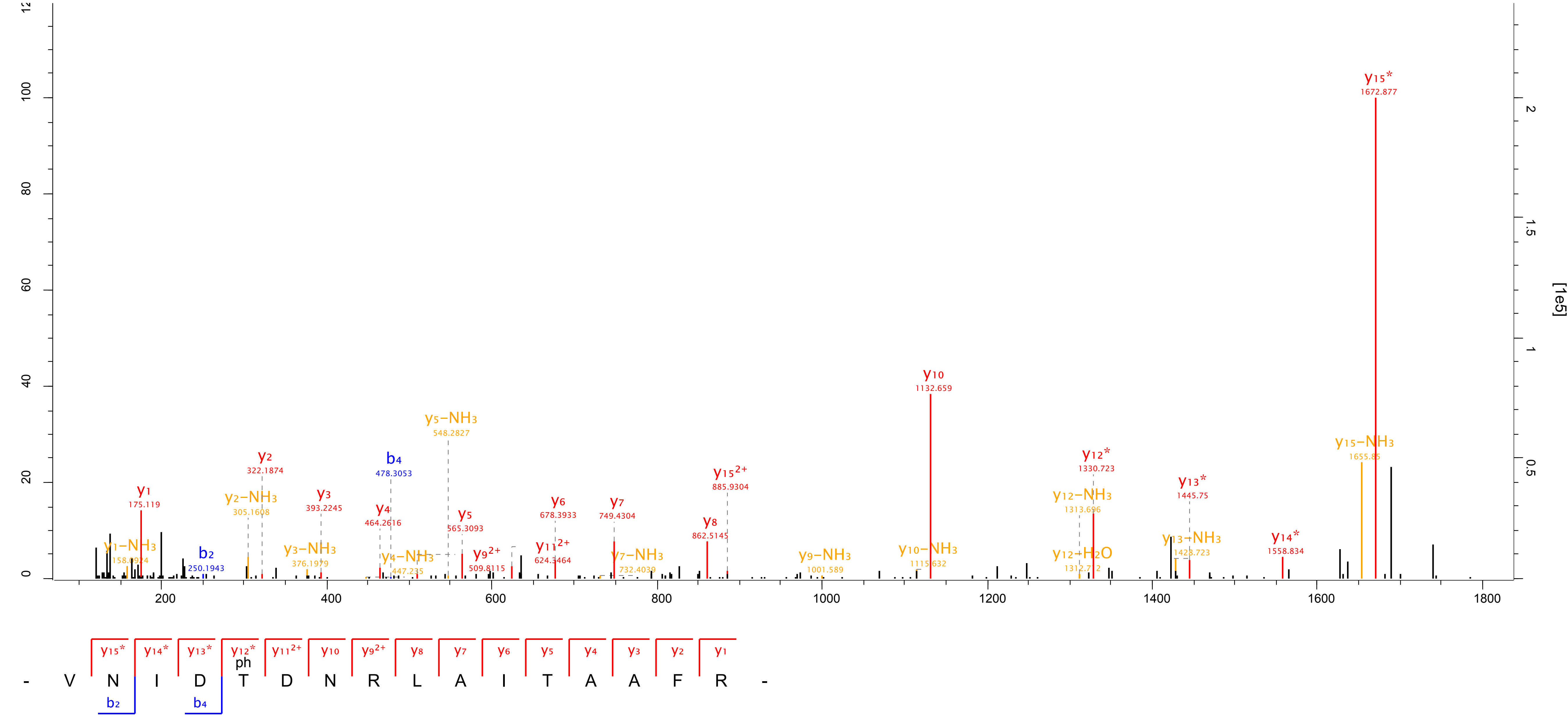

Raw file	Scan	Method	Score	m/z
20141031_PS_Syn6803wt_dimethyl_Mix_Phospho4	16781	FTMS; HCD	90.4	832.8

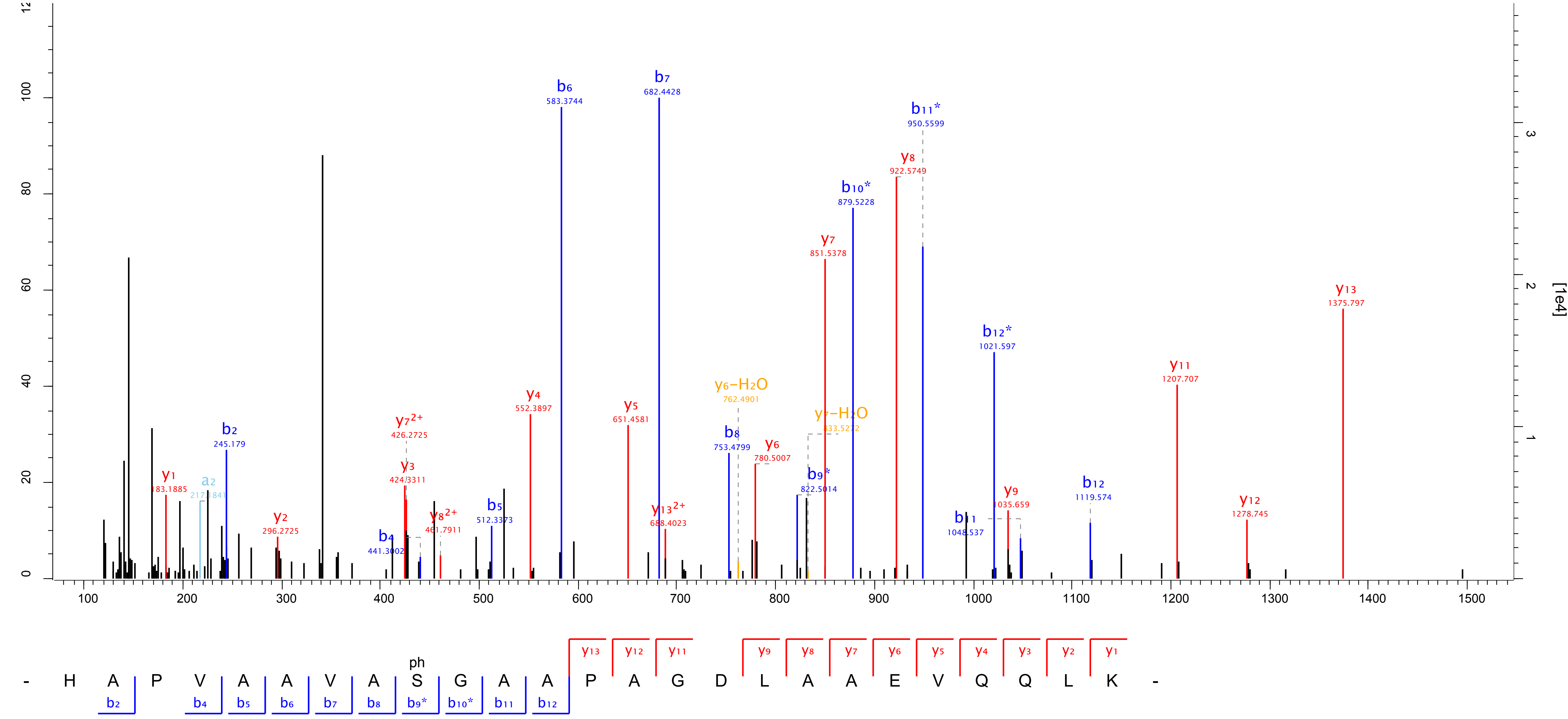

Raw file	Scan	Method	Score	m/z
20141031_PS_Syn6803wt_dimethyl_Mix_Phospho2	17054	FTMS; HCD	53.55	1025.14

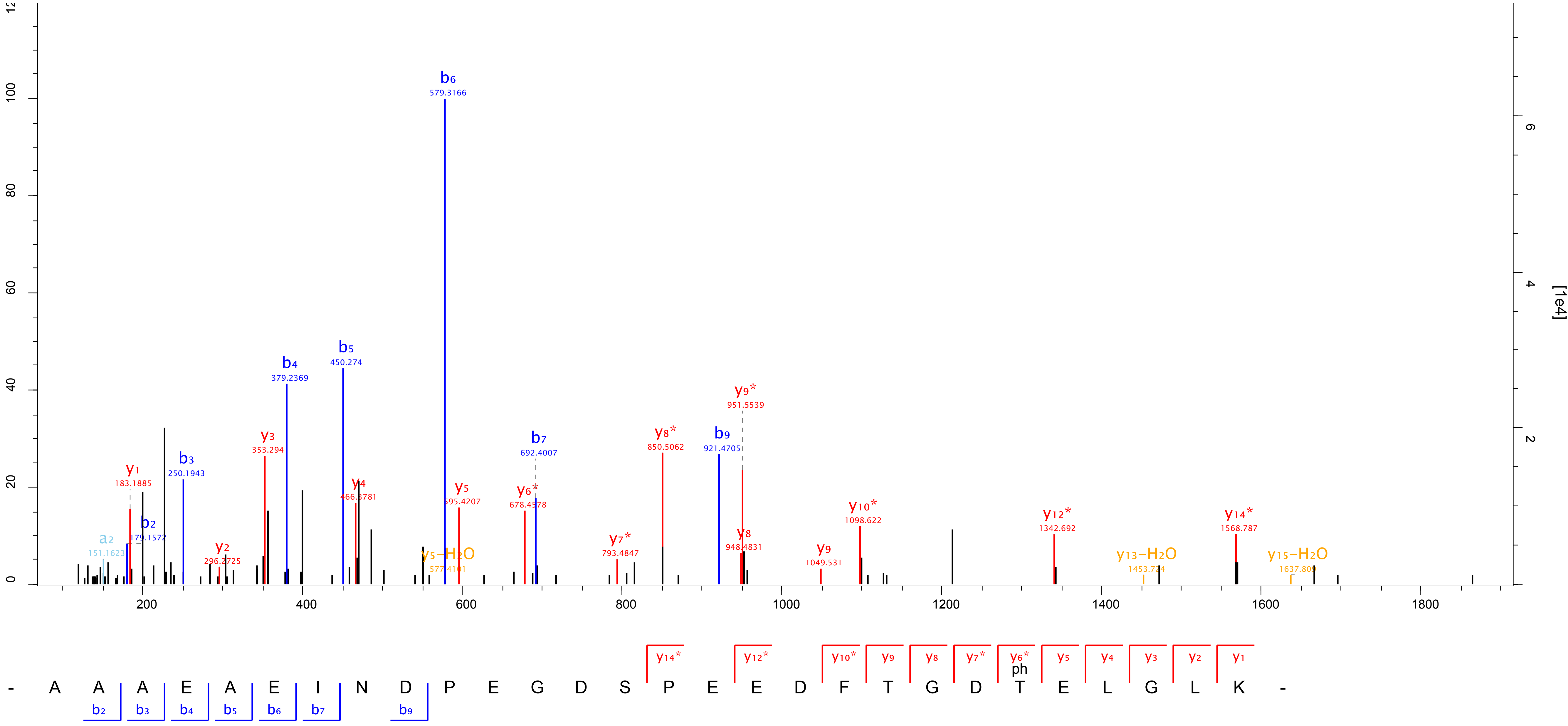

Raw file	Scan	Method	Score	m/z
20141031_PS_Syn6803wt_dimethyl_Mix_Phospho6	17111	FTMS; HCD	163.8	792.44

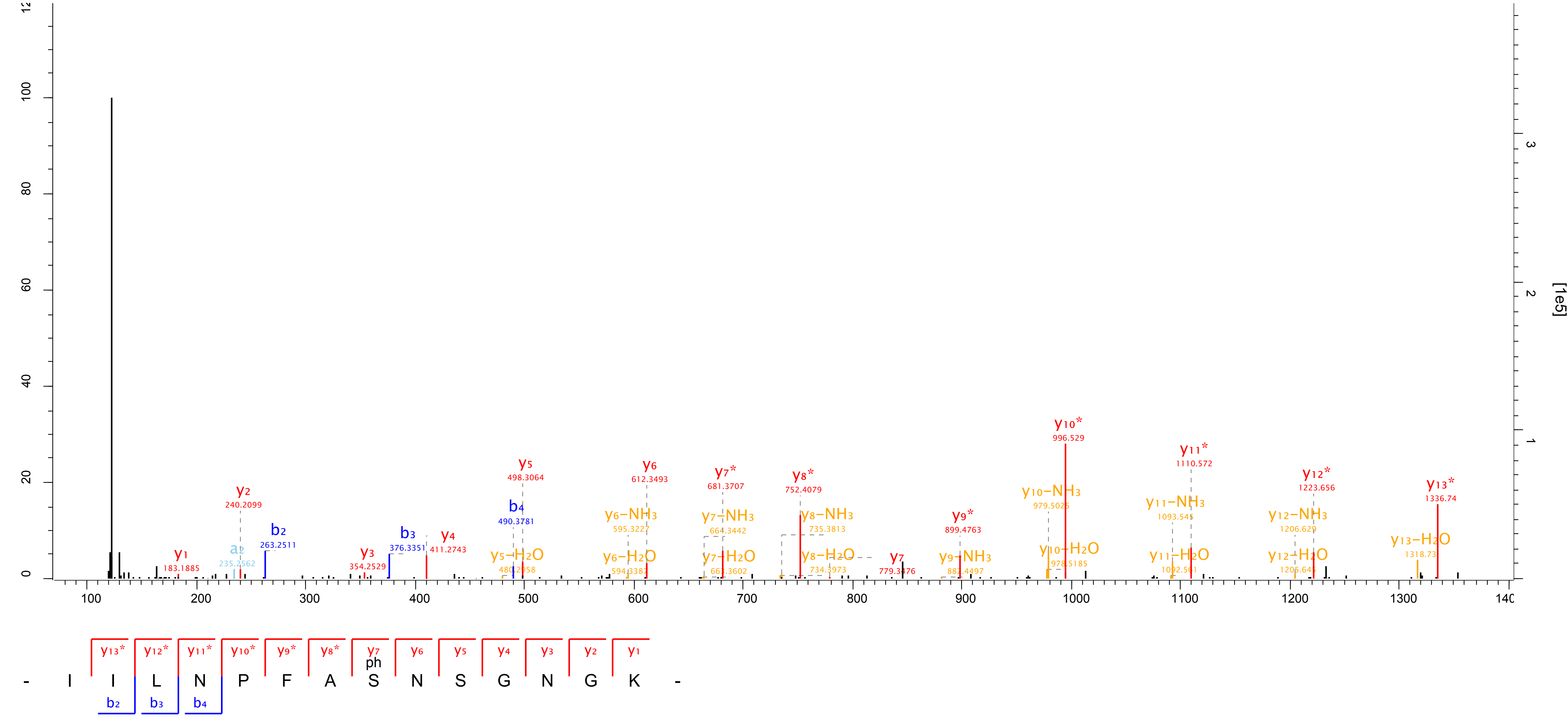

Raw file	Scan	Method	Score	m/z
20141031_PS_Syn6803wt_dimethyl_Mix_Phospho1	17202	FTMS; HCD	86.54	638.34

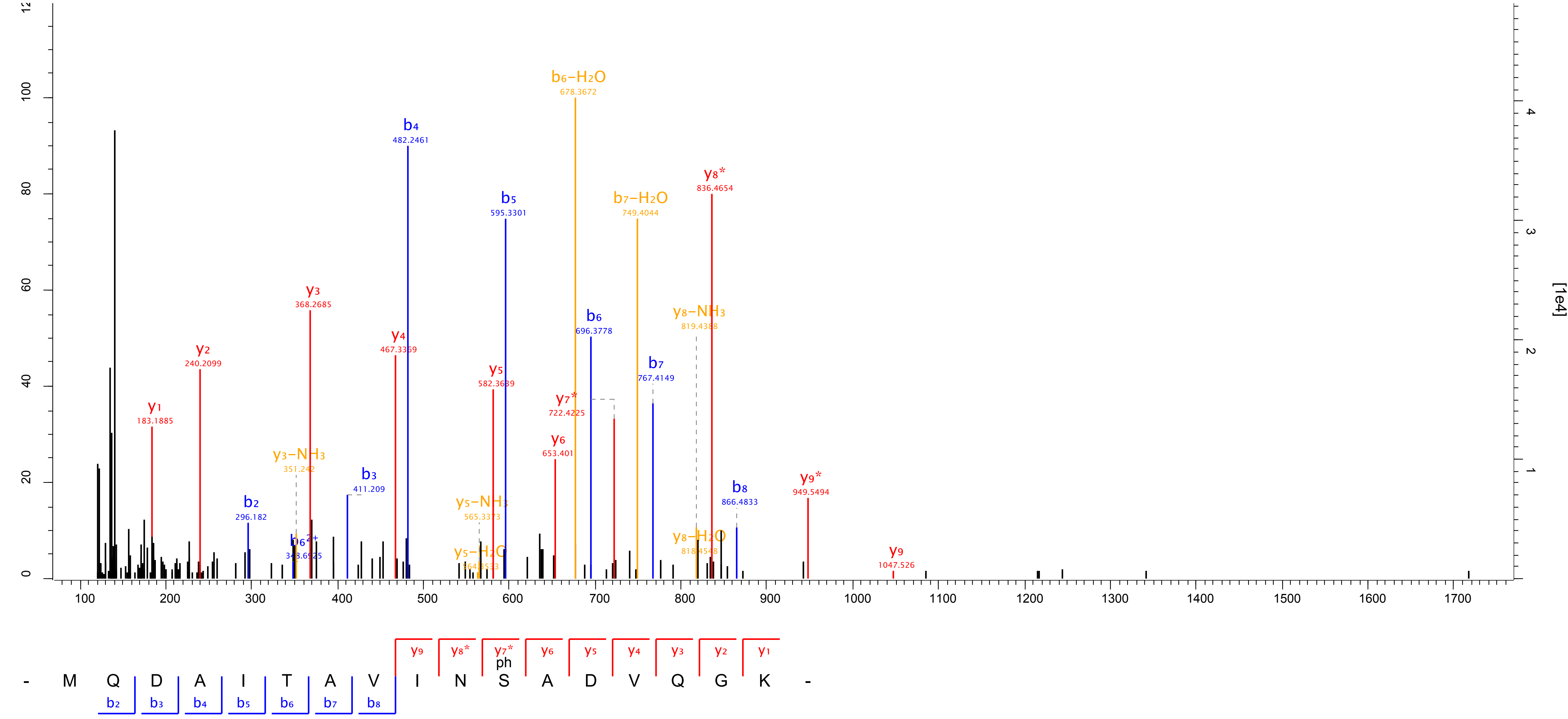

Raw file	Scan	Method	Score	m/z
20150122_PS_Syn_DimethylMix_Rep1_Phospho02	17398	FTMS; HCD	96.63	731.71

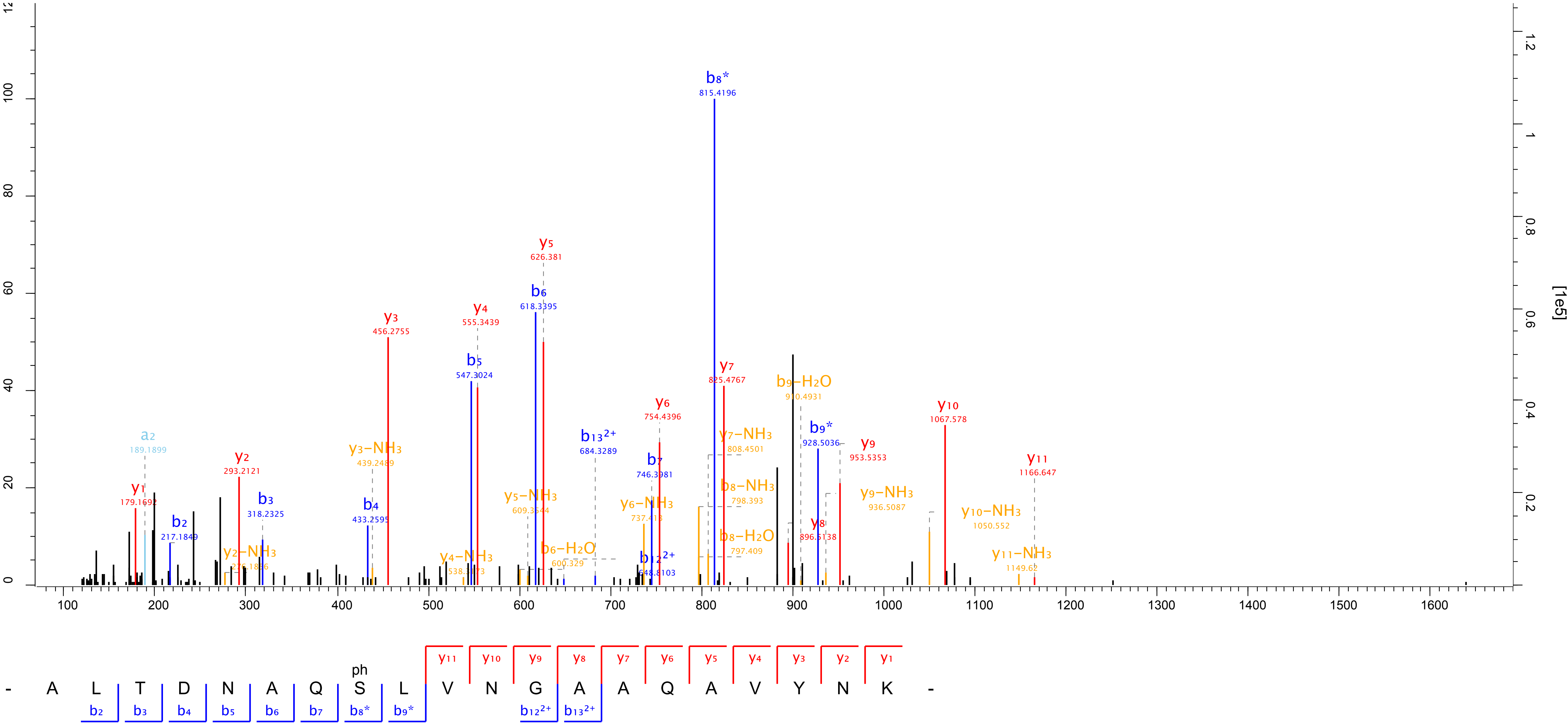

Raw file	Scan	Method	Score	m/z
20141031_PS_Syn6803wt_dimethyl_Mix_Phospho4	17442	FTMS; HCD	52.7	948.23

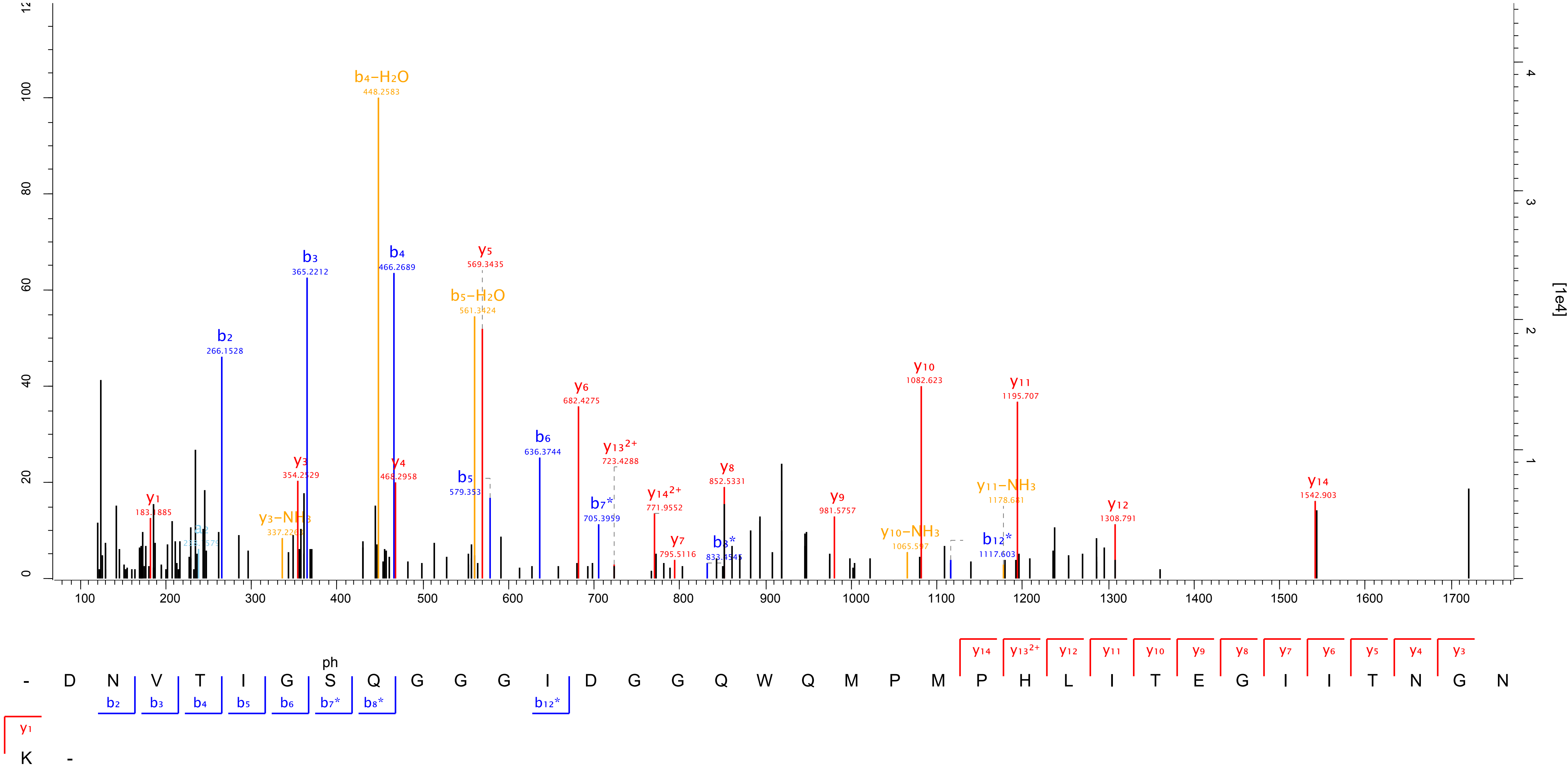

Raw file	Scan	Method	Score	m/z
201417_PS_Syn6803wt_dimethylMix_TiO2_2	17490	FTMS; HCD	125.04	791.4

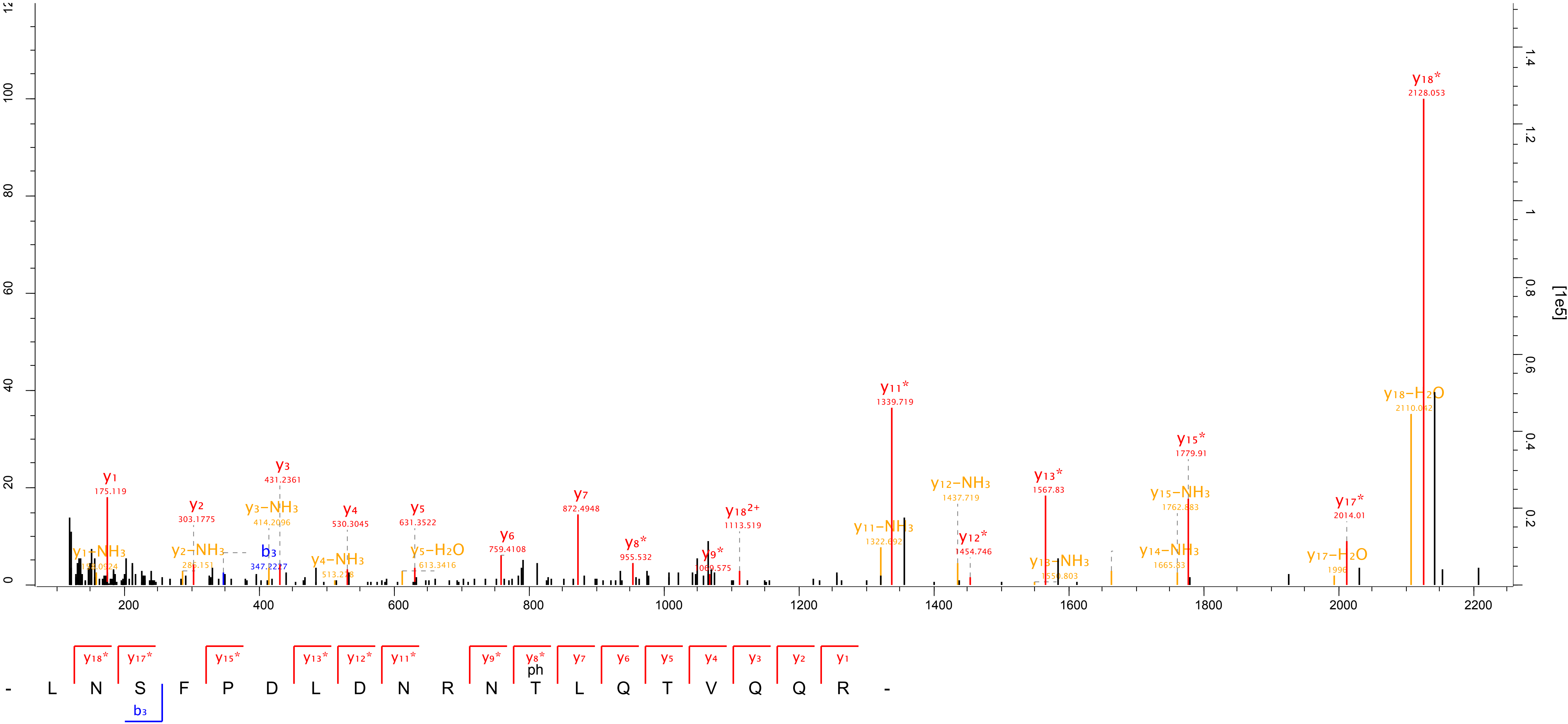

Raw file	Scan	Method	Score	m/z
20141031_PS_Syn6803wt_dimethyl_Mix_Phospho3	17807	FTMS; HCD	103.91	616.86

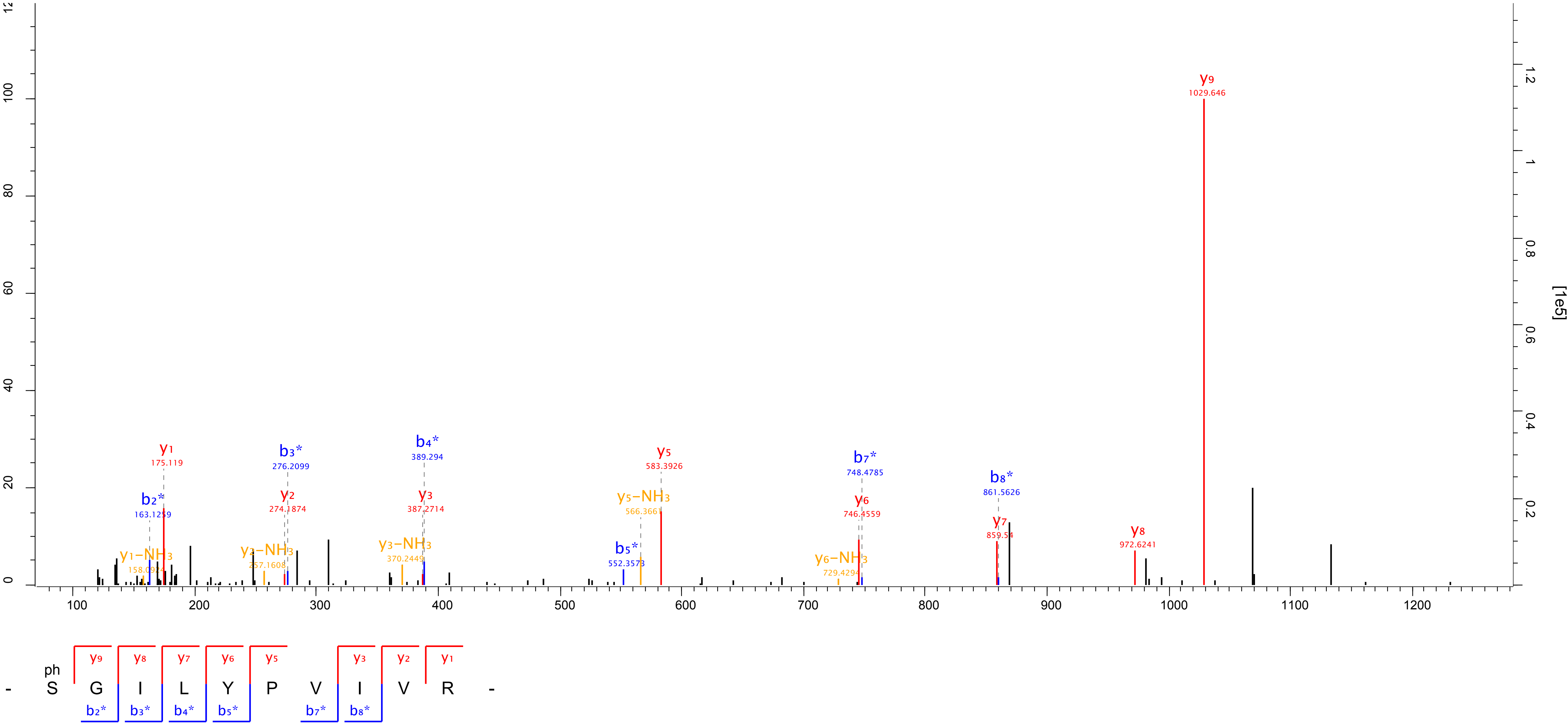

Raw file	Scan	Method	Score	m/z
20141031_PS_Syn6803wt_dimethyl_Mix_Phospho4	18101	FTMS; HCD	44.79	612.99

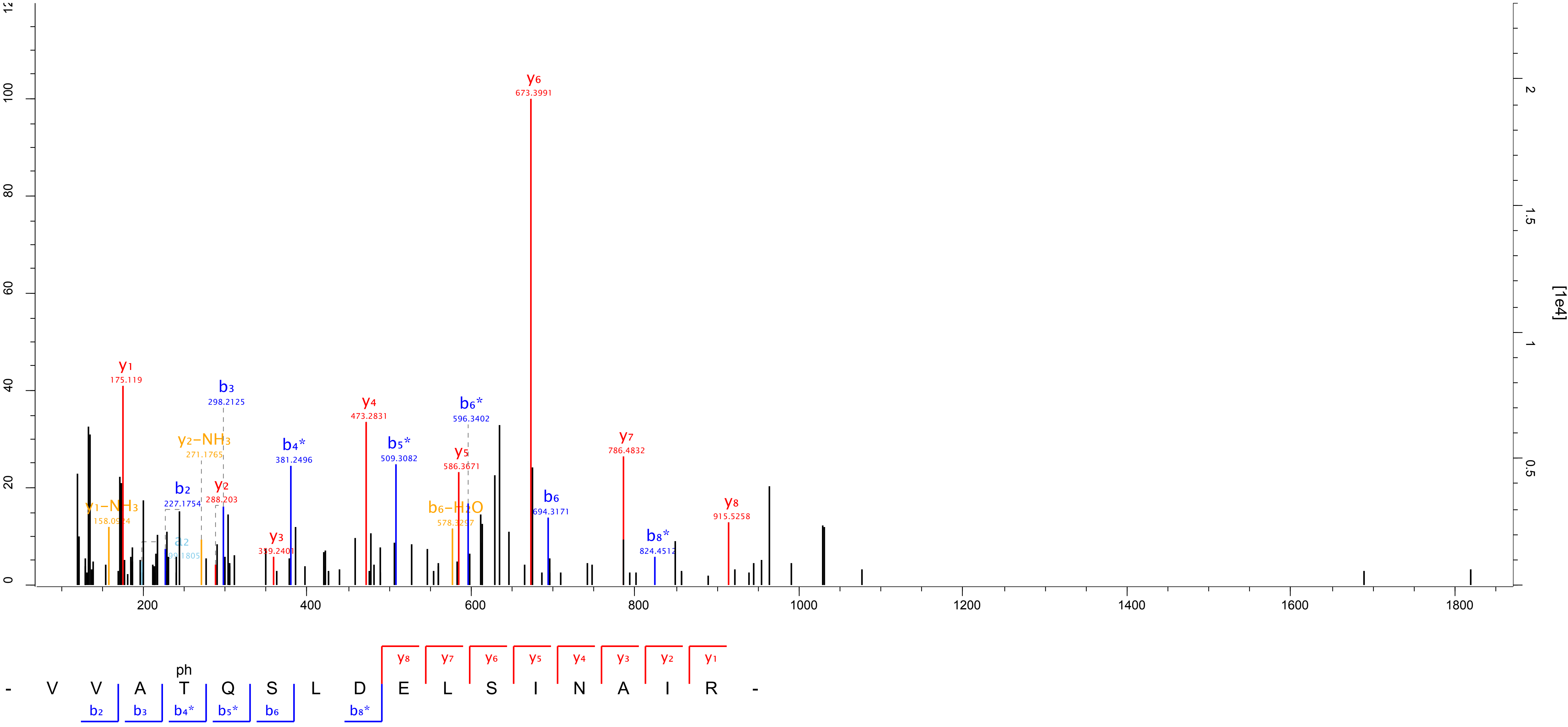

Raw file	Scan	Method	Score	m/z
20141031_PS_Syn6803wt_dimethyl_Mix_Phospho4	18005	FTMS; HCD	118.51	818.09

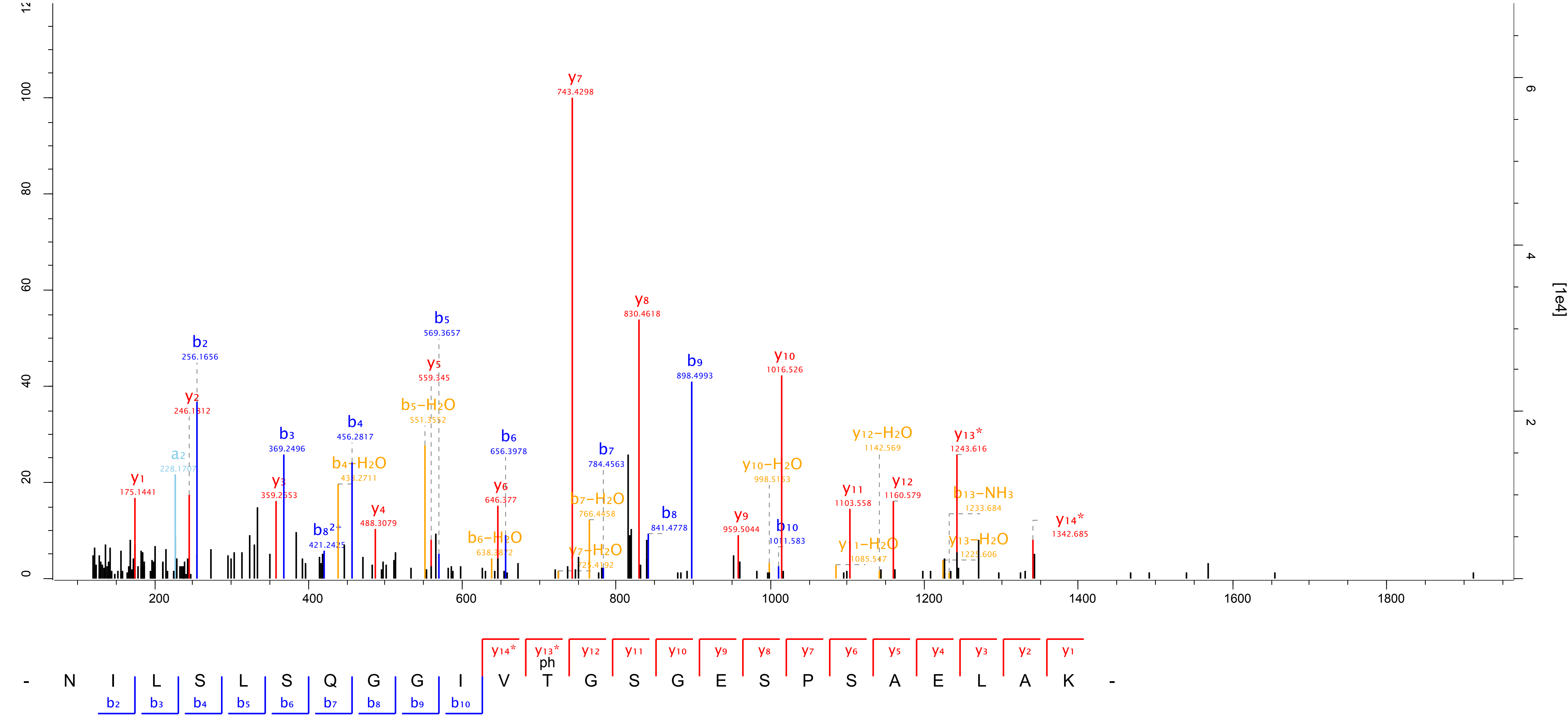

Raw file	Scan	Method	Score	m/z
20141031_PS_Syn6803wt_dimethyl_Mix_Phospho4	18762	FTMS; HCD	105.32	754.72

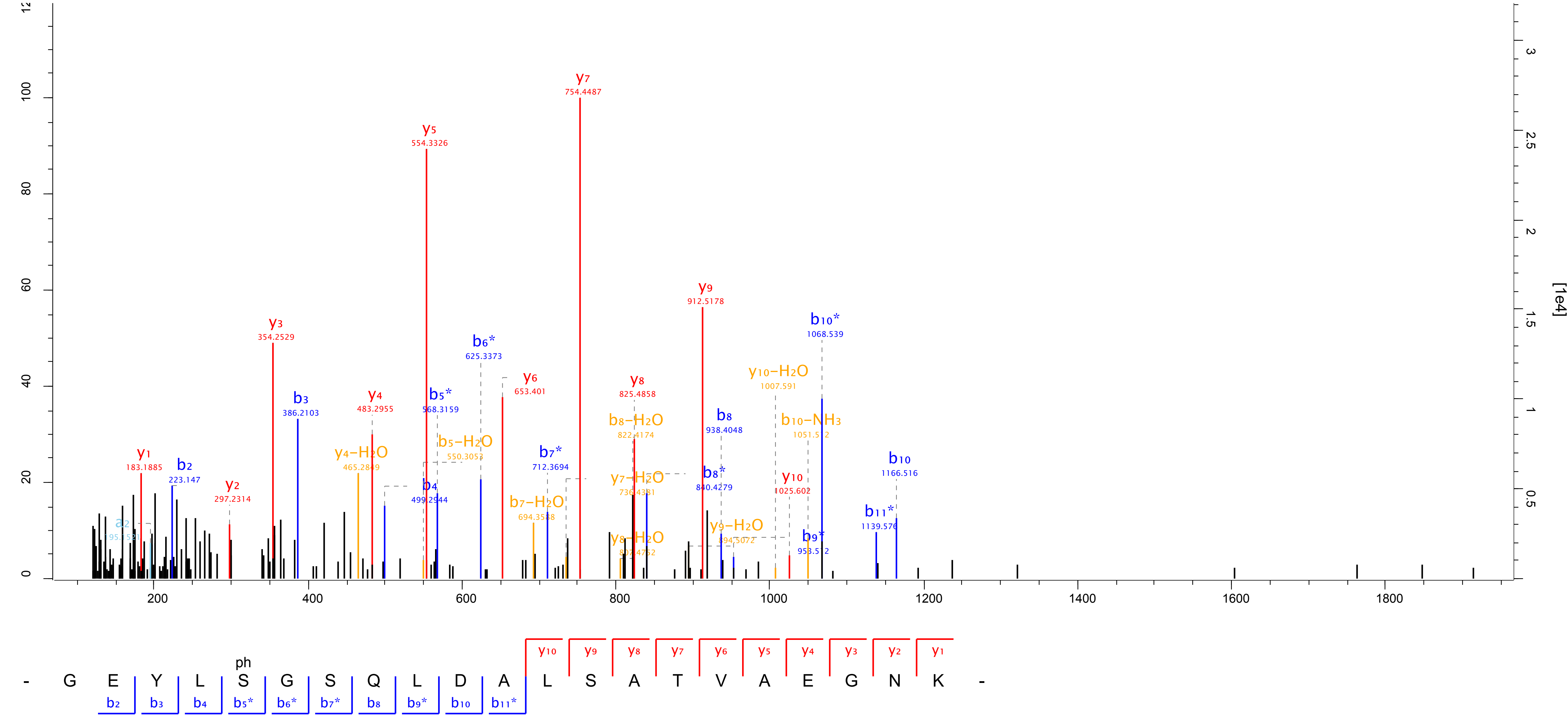

Raw file	Scan	Method	Score	m/z
20141031_PS_Syn6803wt_dimethyl_Mix_Phospho7	19755	FTMS; HCD	54.77	532.65

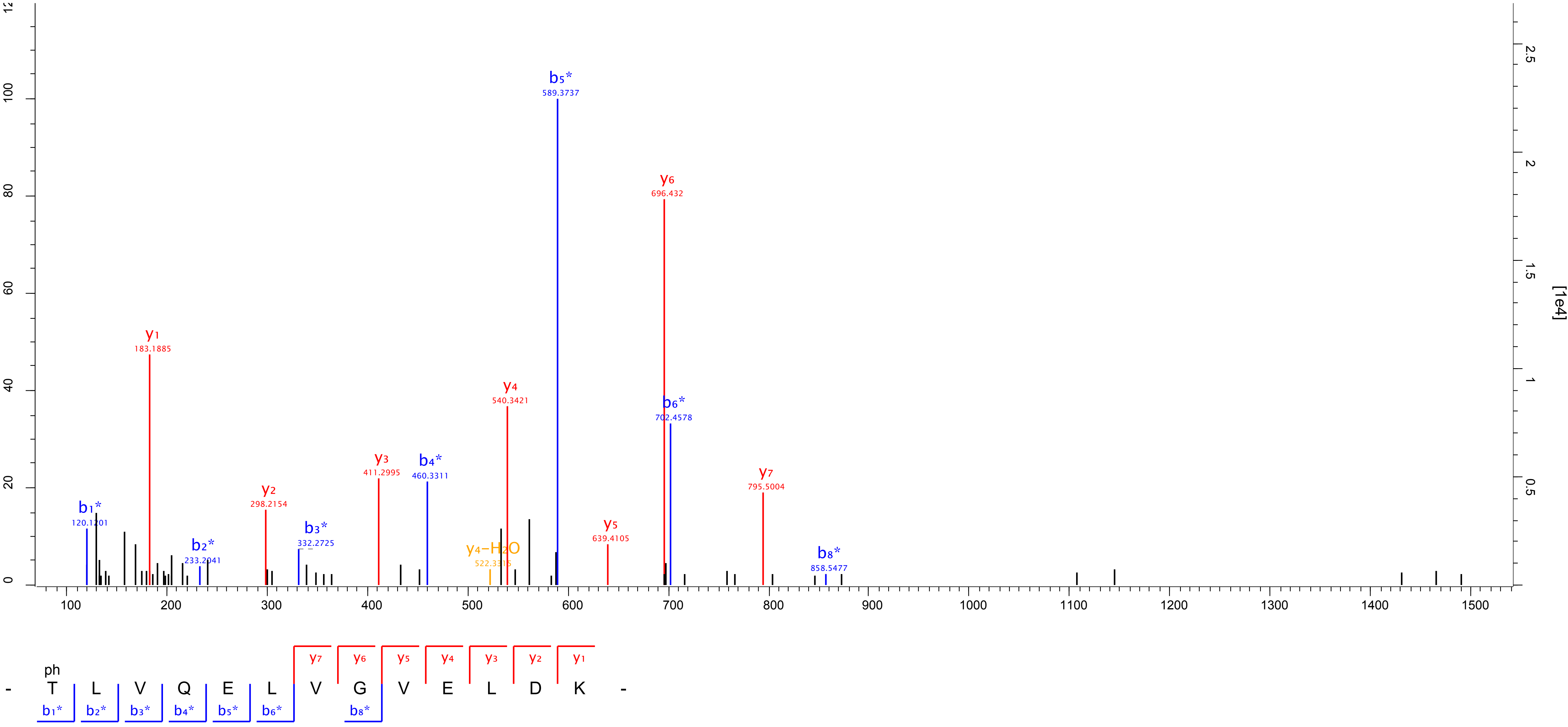

Raw file	Scan	Method	Score	m/z
20141031_PS_Syn6803wt_dimethyl_Mix_Phospho3	19781	FTMS; HCD	101.6	700.36

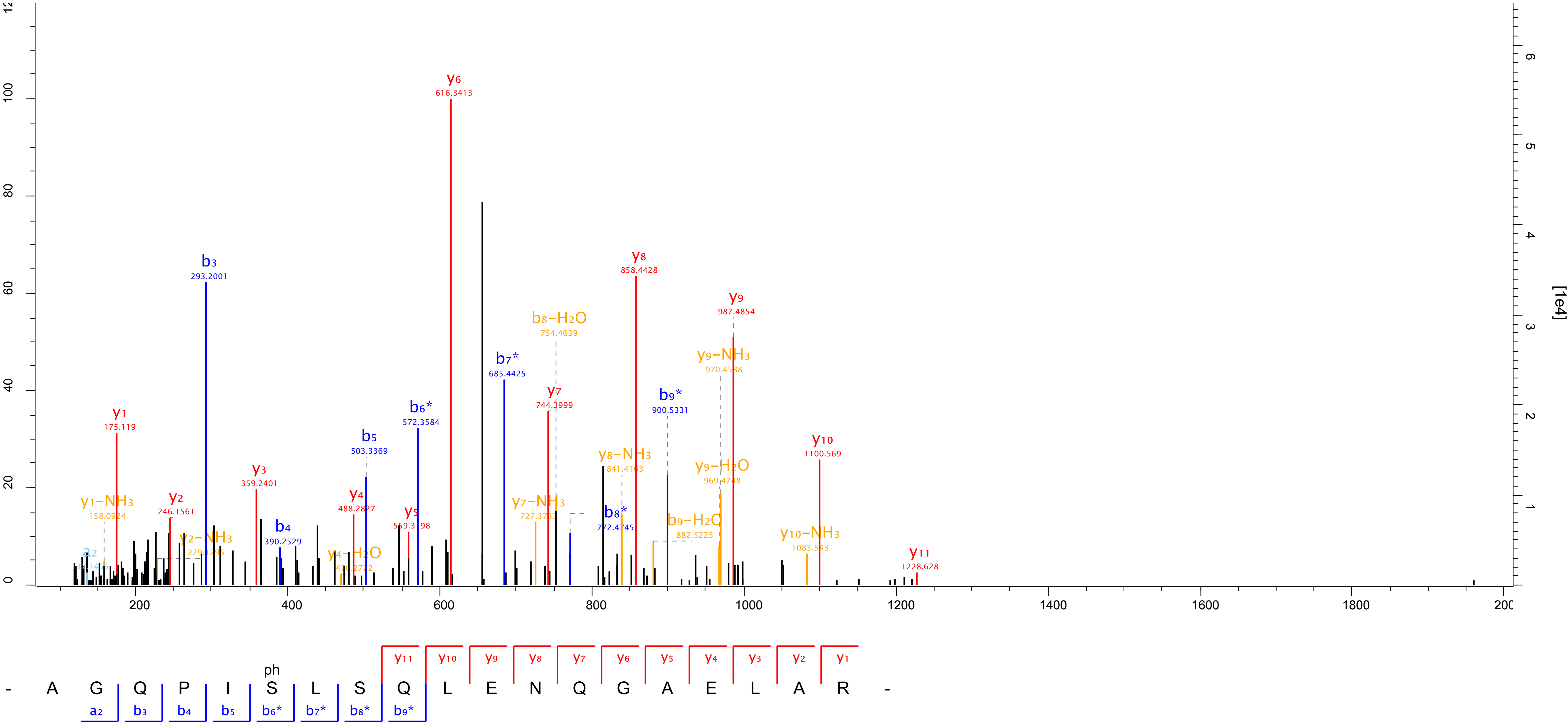

Raw file	Scan	Method	Score	m/z
20141031_PS_Syn6803wt_dimethyl_Mix_Phospho4	19819	FTMS; HCD	100.04	601.33

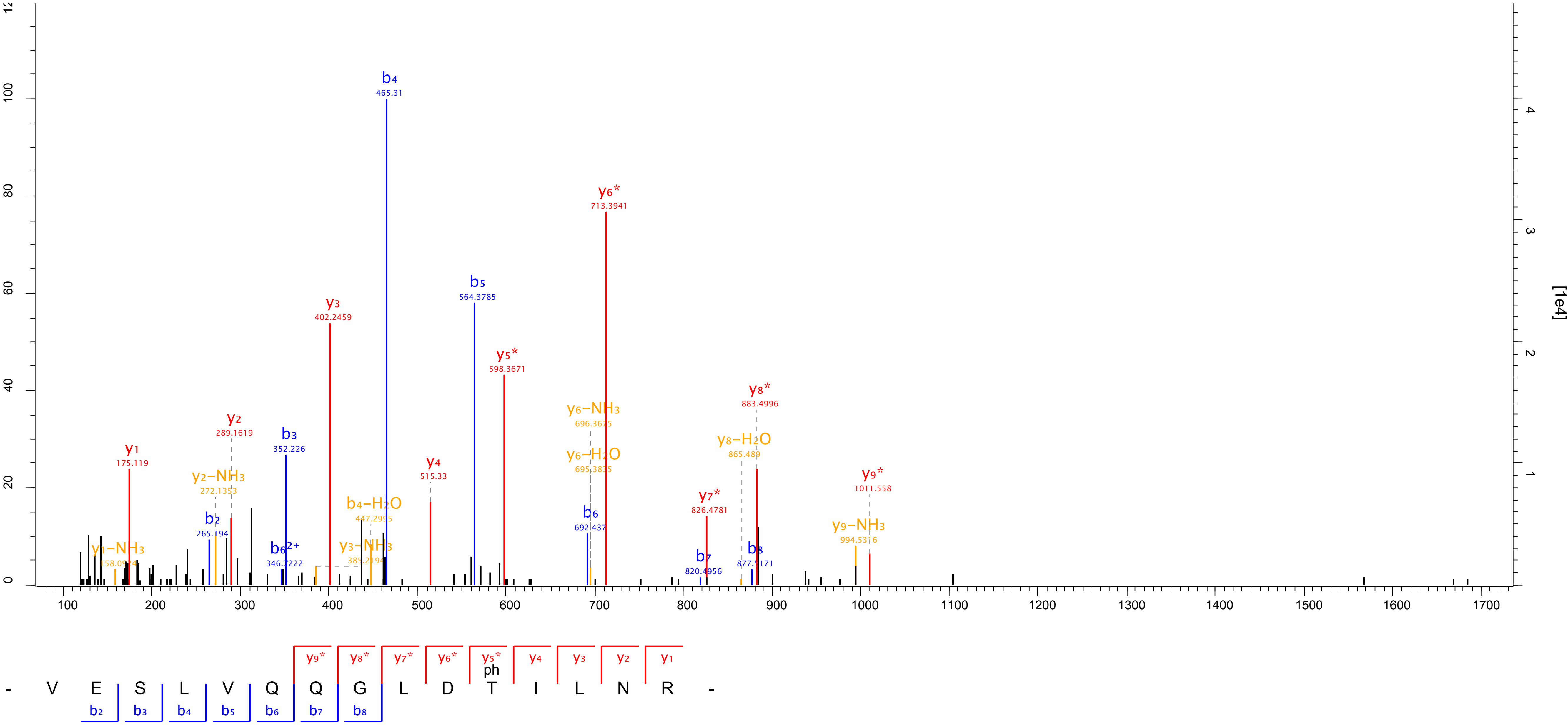

Raw file	Scan	Method	Score	m/z
20141031_PS_Syn6803wt_dimethyl_Mix_Phospho1	19992	FTMS; HCD	117.8	849.9

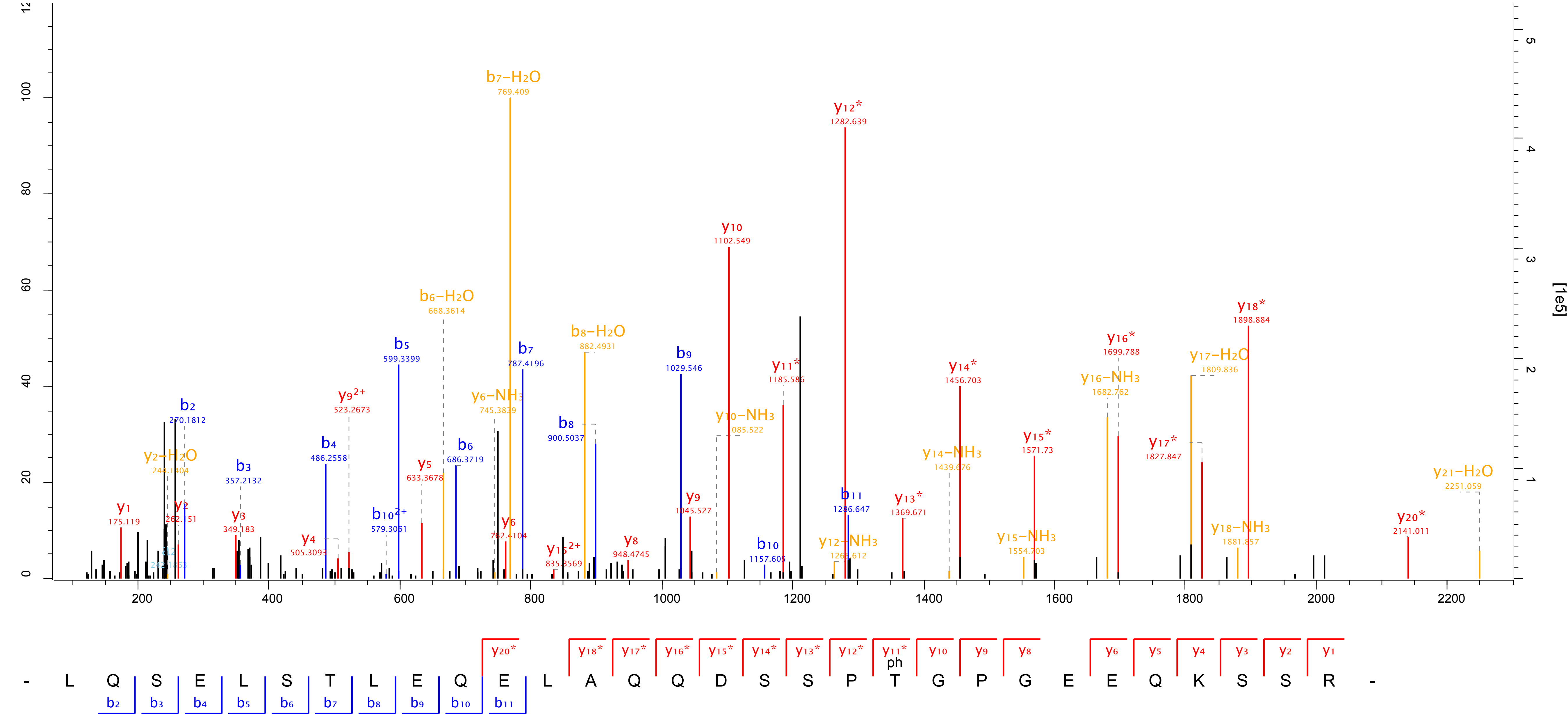

Raw file	Scan	Method	Score	m/z
20150122_PS_Syn_DimethylMix_Rep1_Phospho01	20191	FTMS; HCD	189.09	1128.06

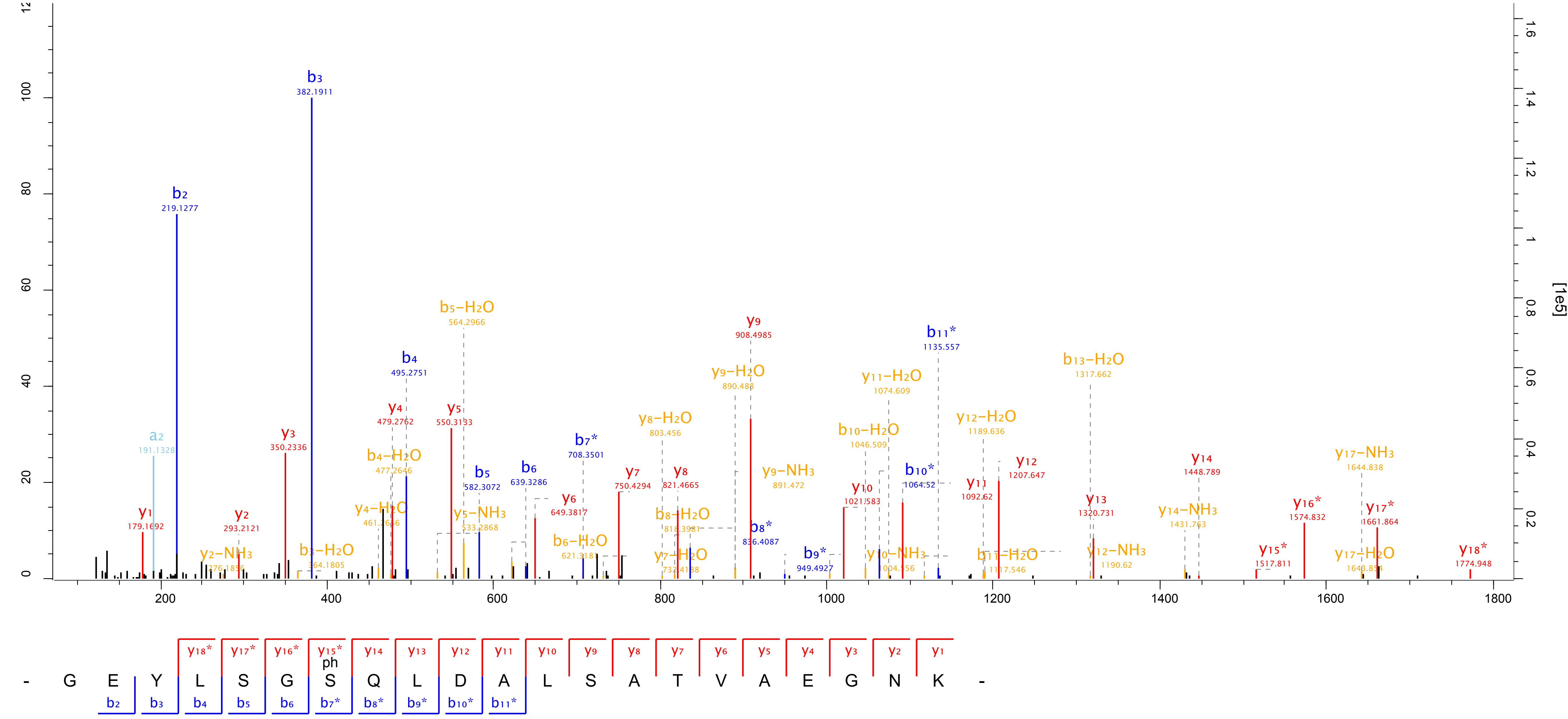

Raw file	Scan	Method	Score	m/z
201417_PS_Syn6803wt_dimethylMix_TiO2_3	20673	FTMS; HCD	99.95	1151.6

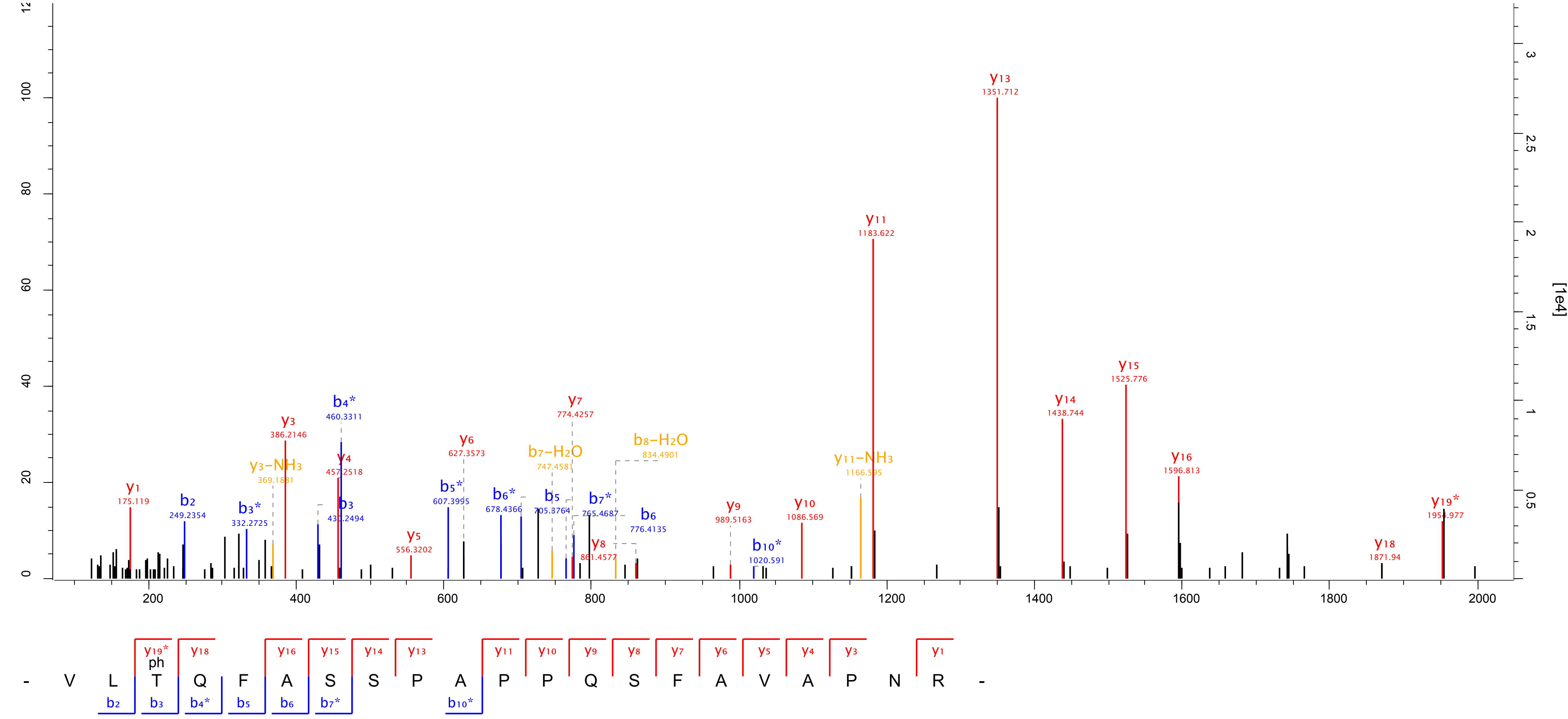

Raw file	Scan	Method	Score	m/z
201417_PS_Syn6803wt_dimethylMix_TiO2_4	21362	FTMS; HCD	74.78	994

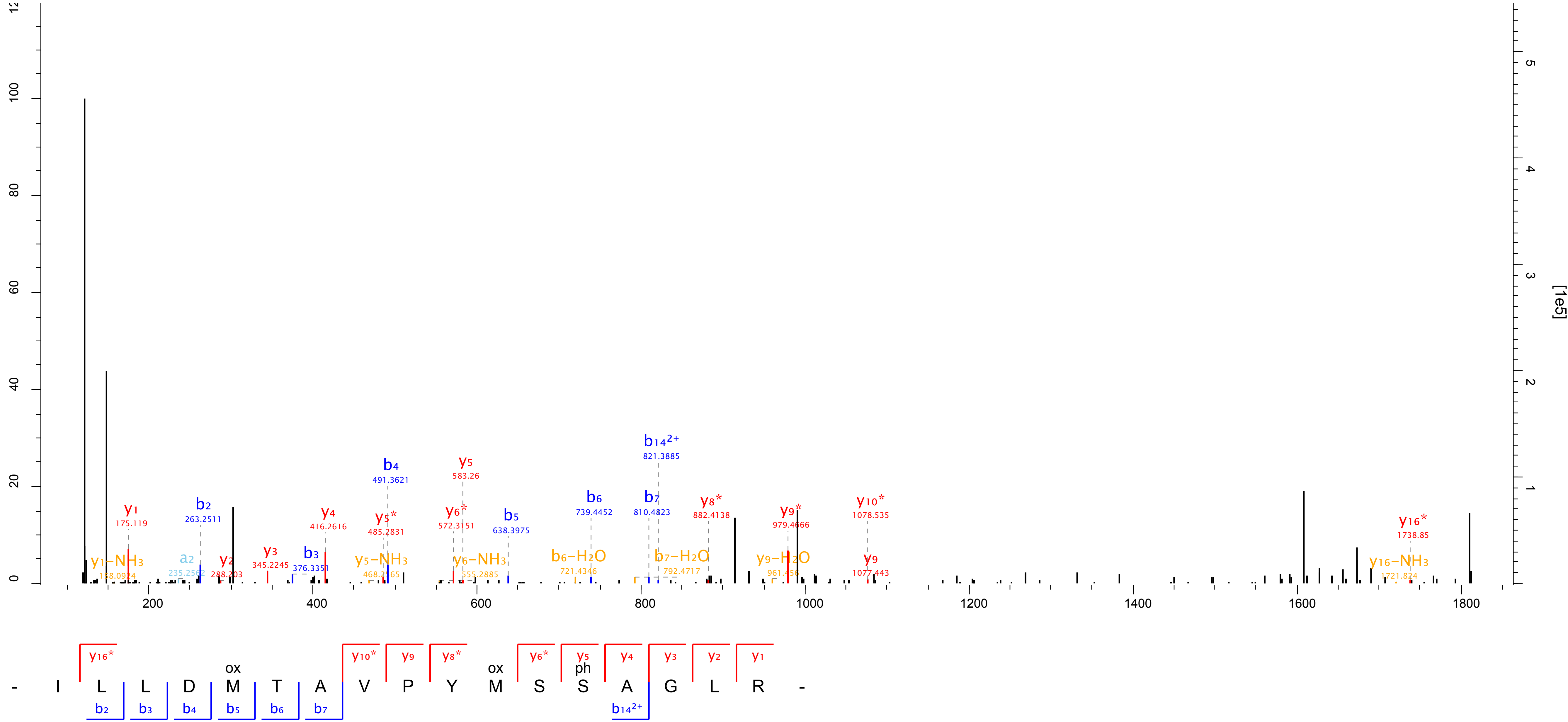

Raw file	Scan	Method	Score	m/z
20141031_PS_Syn6803wt_dimethyl_Mix_Phospho1	21375	FTMS; HCD	50.88	1109.2

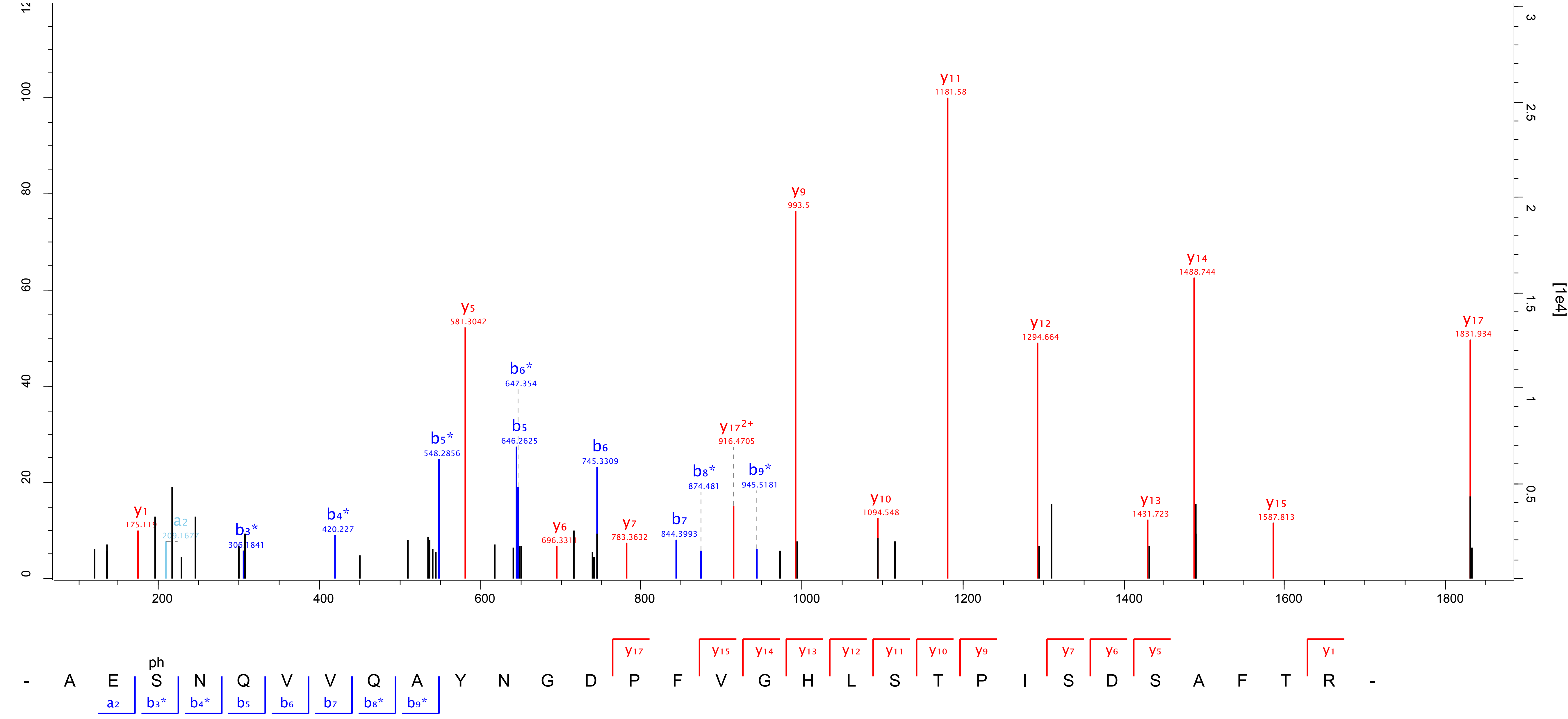

Raw file	Scan	Method	Score	m/z
20141031_PS_Syn6803wt_dimethyl_Mix_Phospho7	22020	FTMS; HCD	208.62	886.46

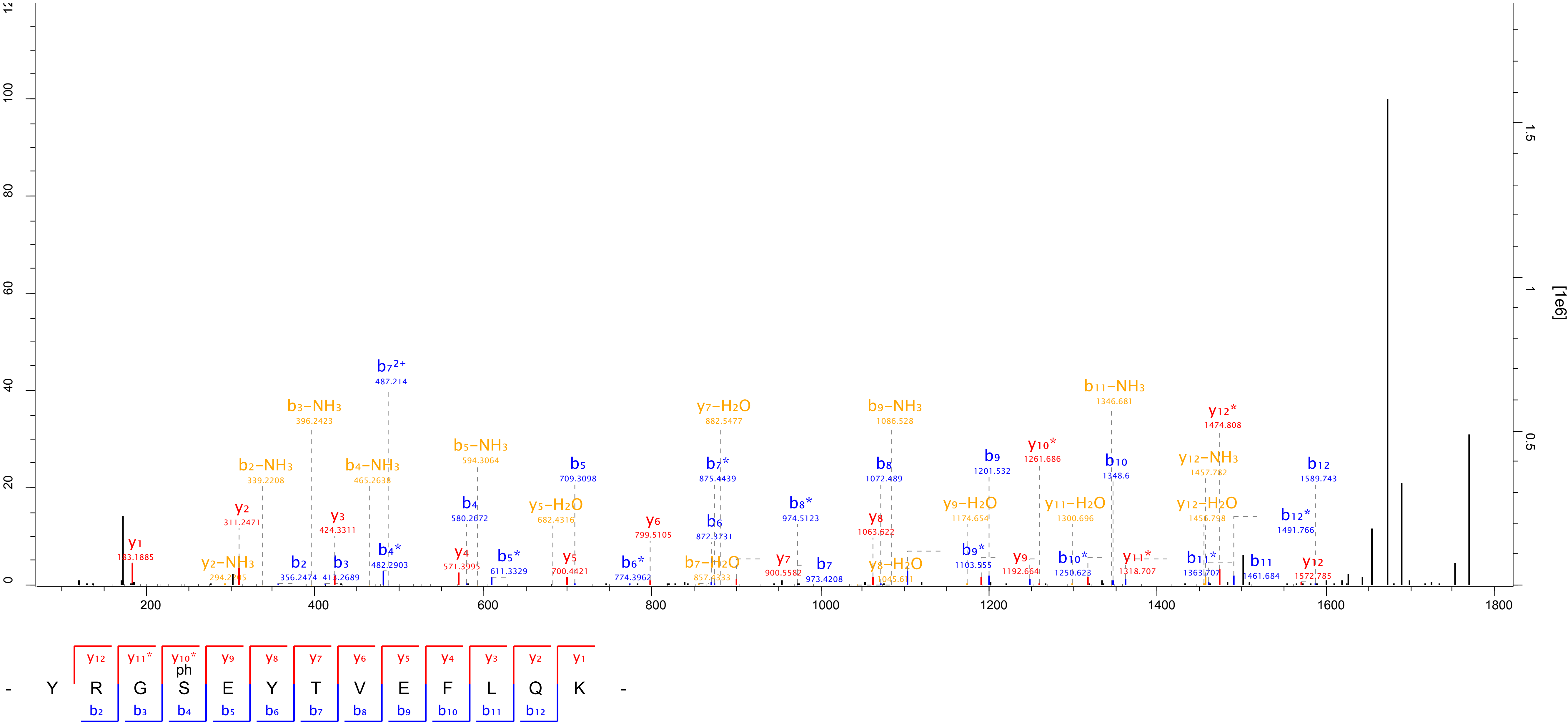

Raw file	Scan	Method	Score	m/z
20141031_PS_Syn6803wt_dimethyl_Mix_Phospho4	23107	FTMS; HCD	242.11	1079.57

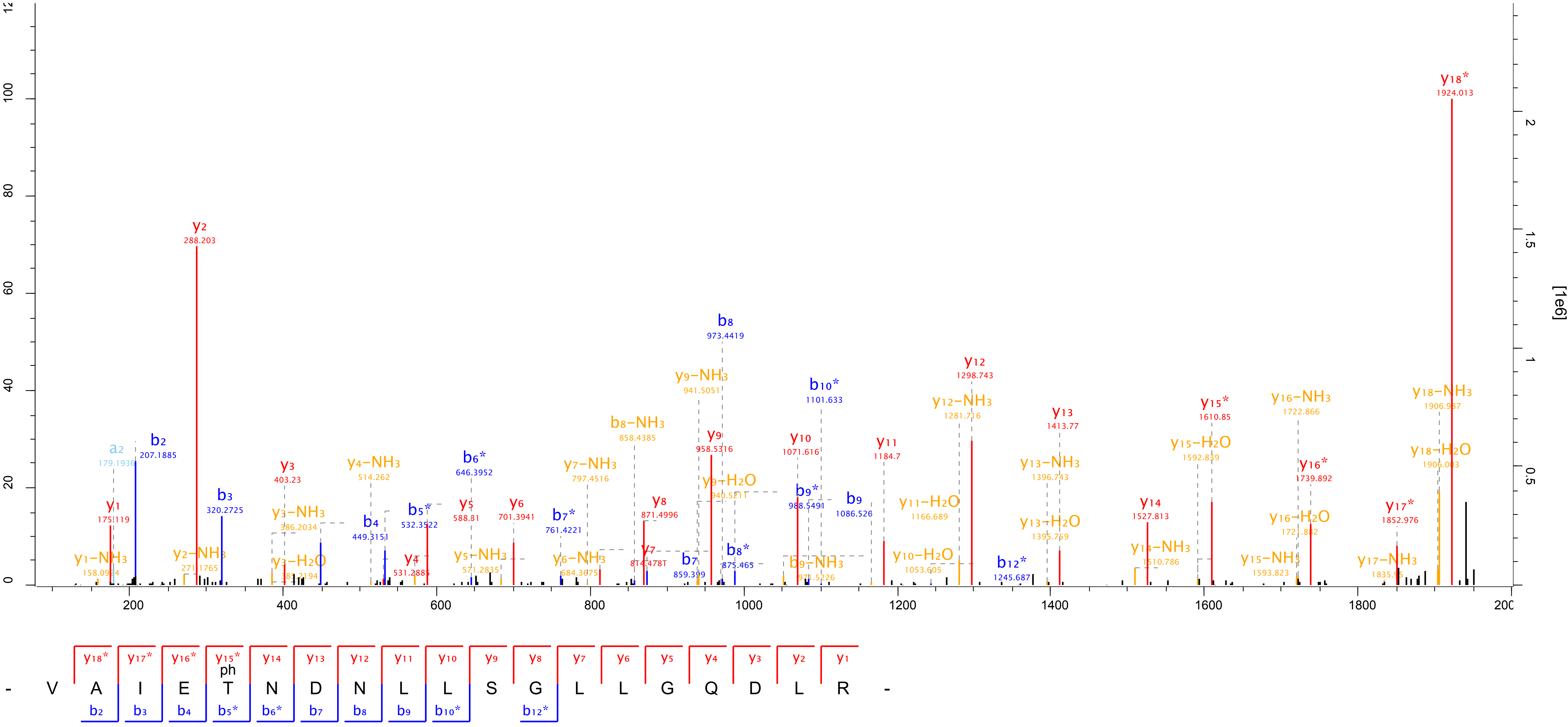

Raw file	Scan	Method	Score	m/z
201417_PS_Syn6803wt_dimethylMix_TiO2_7	3234	FTMS; HCD	45.28	605.79

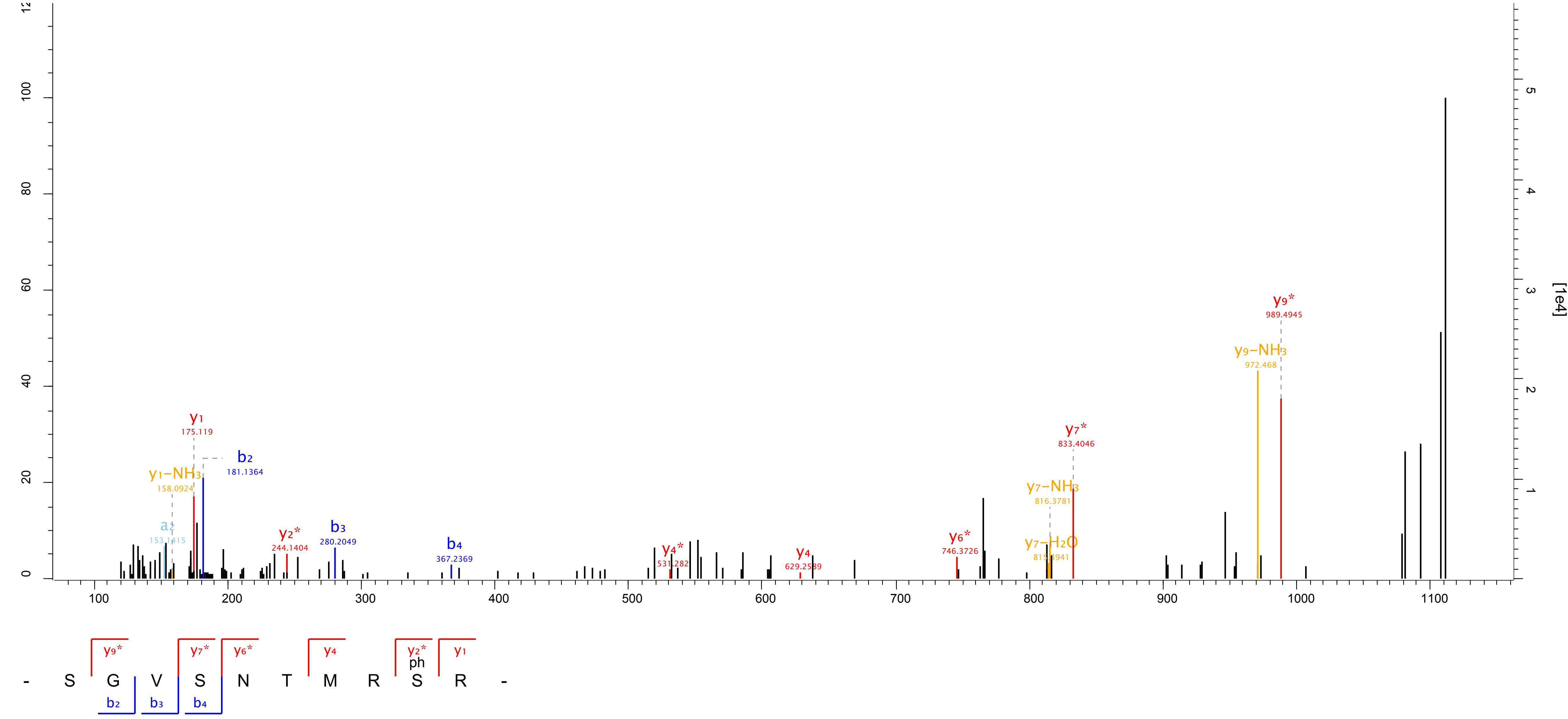

Raw file	Scan	Method	Score	m/z
201417_PS_Syn6803wt_dimethylMix_TiO2_1	3348	FTMS; HCD	43.22	835.92

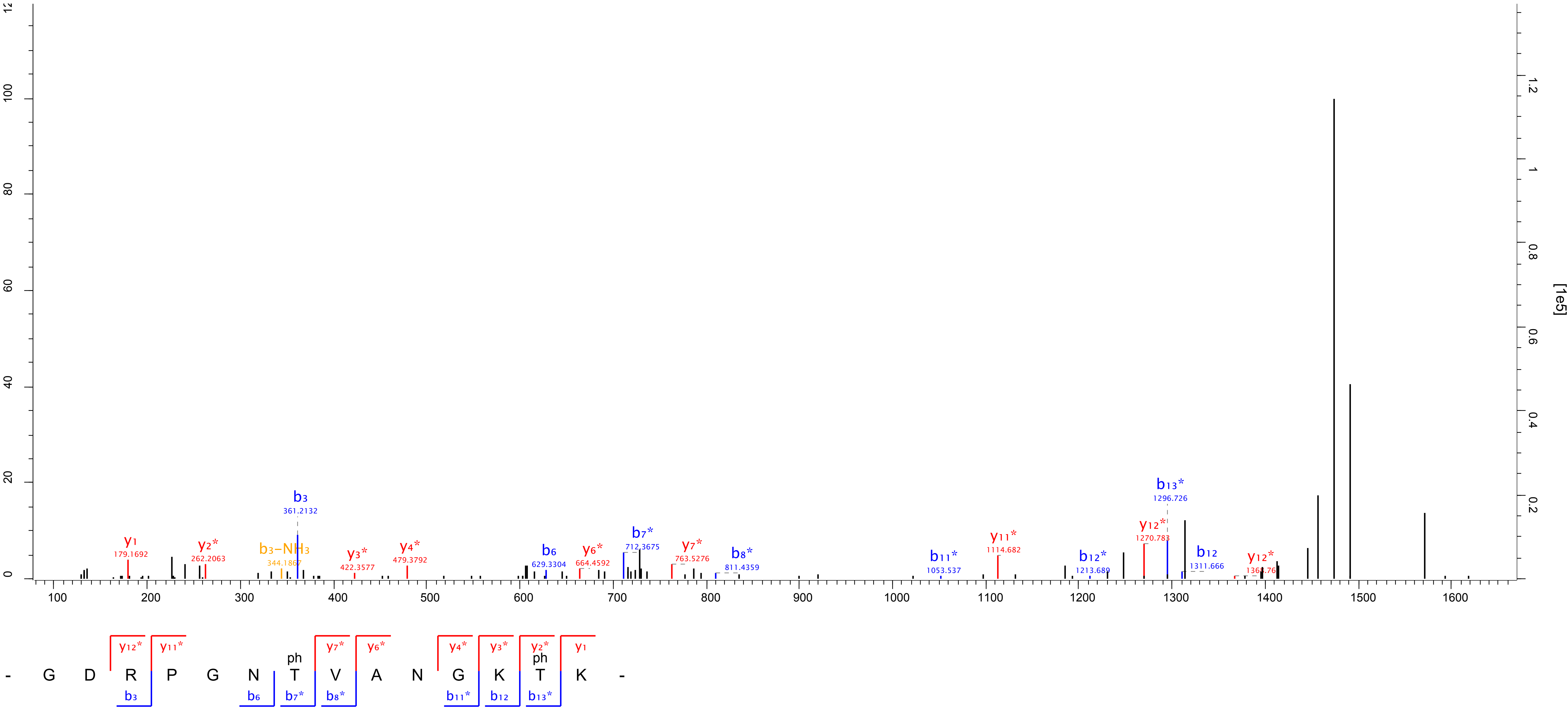

Raw file	Scan	Method	Score	m/z
20150122_PS_Syn_DimethylMix_Rep1_Phospho04	3452	FTMS; HCD	120.87	572.27

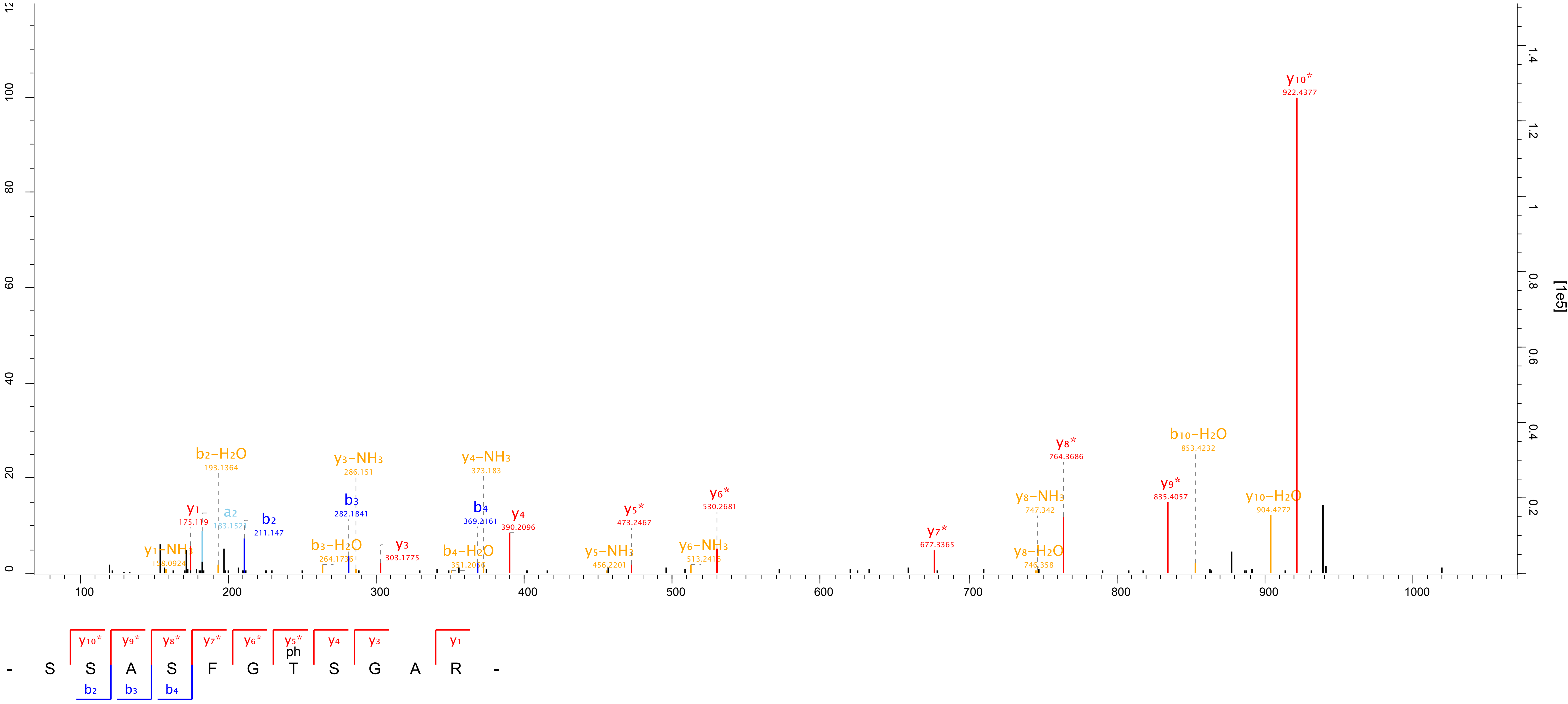

Raw file	Scan	Method	Score	m/z
201417_PS_Syn6803wt_dimethylMix_TiO2_2	3514	FTMS; HCD	80.37	626.31

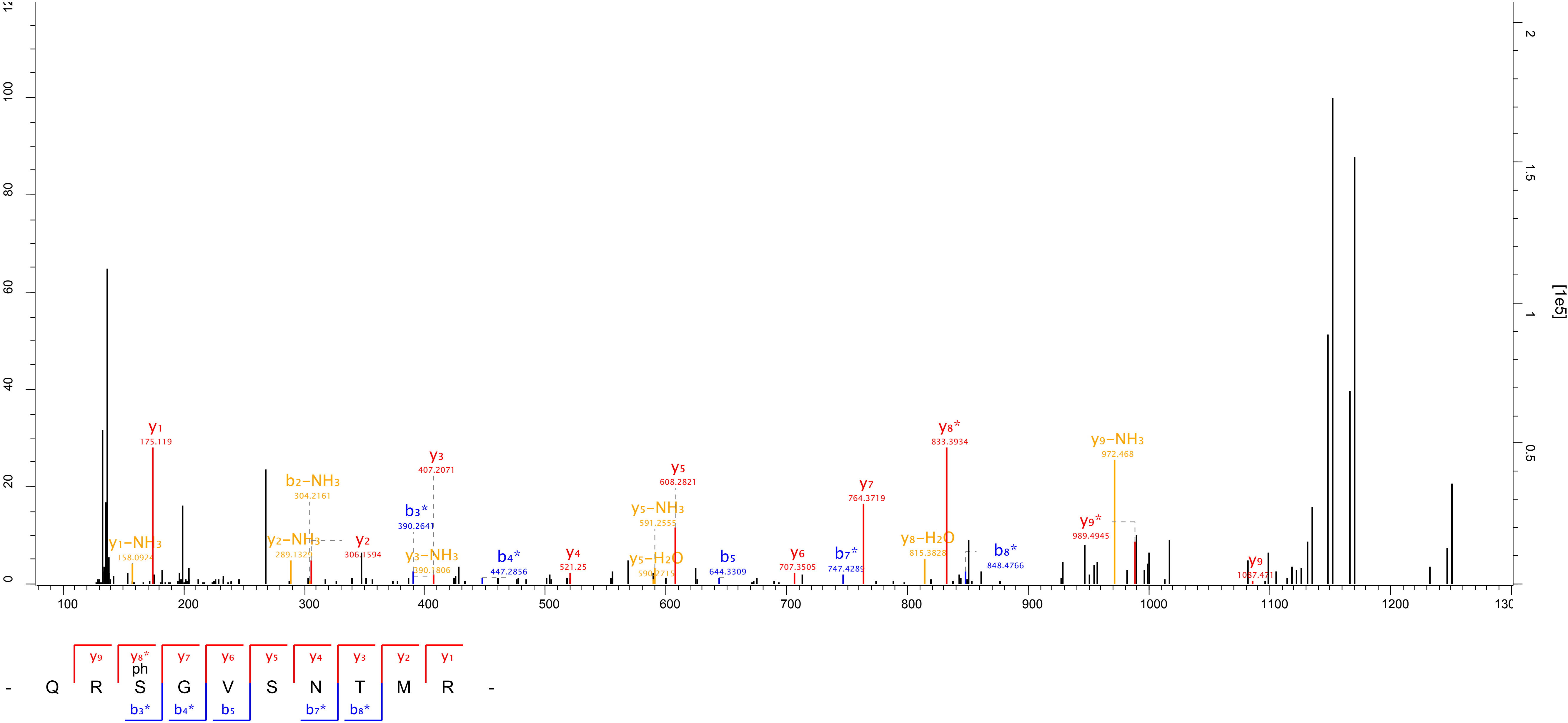

Raw file	Scan	Method	Score	m/z
201417_PS_Syn6803wt_dimethylMix_TiO2_1	3794	FTMS; HCD	202.63	742.84

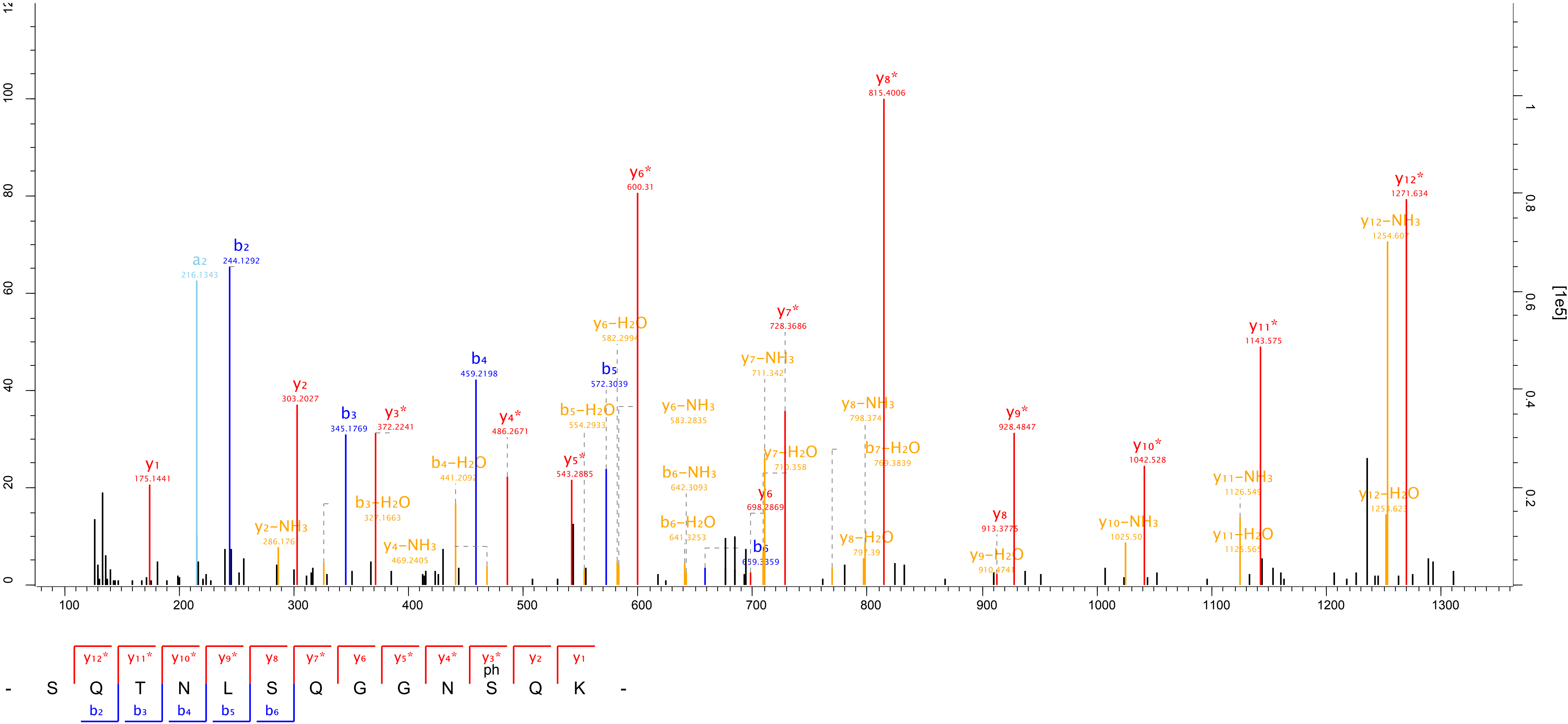

Raw file	Scan	Method	Score	m/z
20141031_PS_Syn6803wt_dimethyl_Mix_Phospho3	3918	FTMS; HCD	72.57	658.65

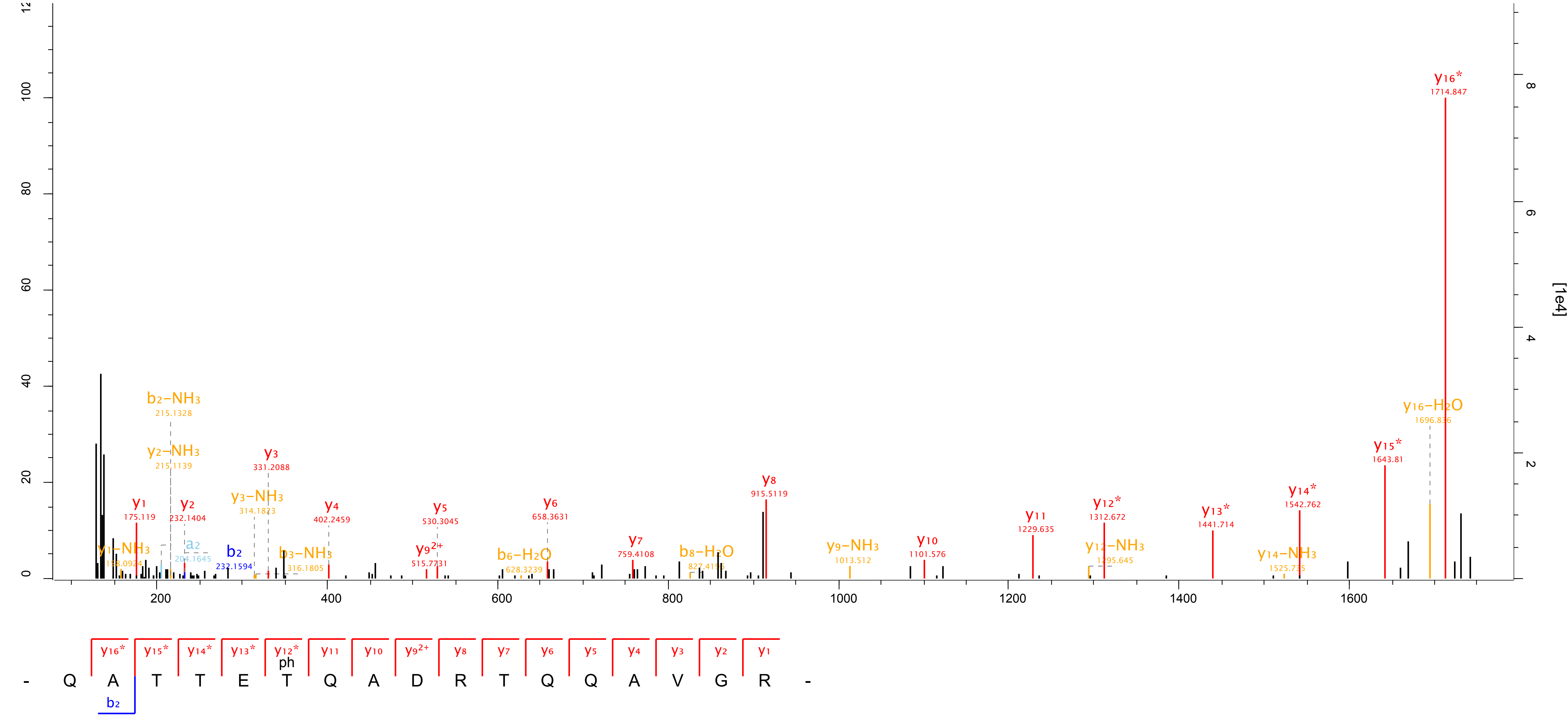

Raw file	Scan	Method	Score	m/z
20141031_PS_Syn6803wt_dimethyl_Mix_Phospho2	4801	FTMS; HCD	42.58	539.3

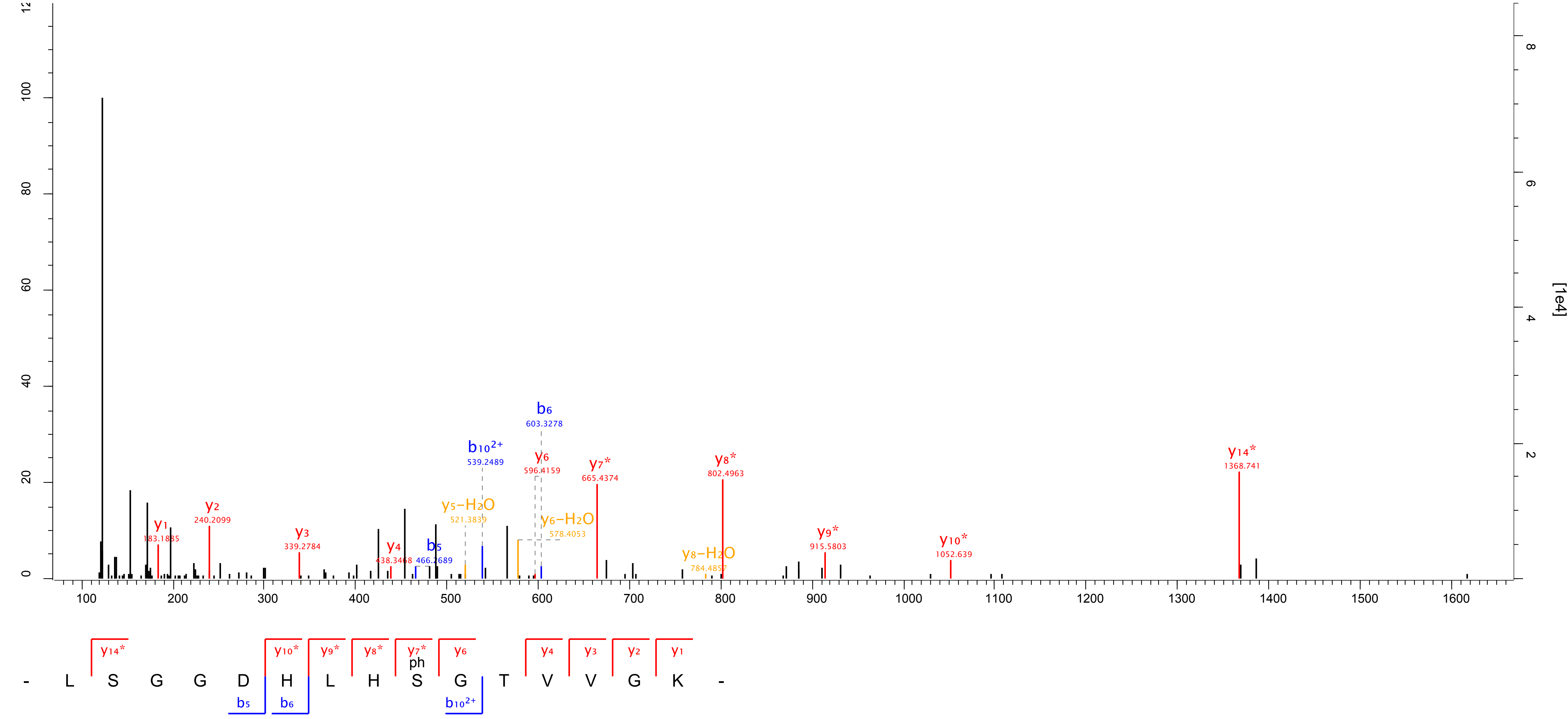

Raw file	Scan	Method	Score	m/z
201417_PS_Syn6803wt_dimethylMix_TiO2_6	4883	FTMS; HCD	117.7	728.38

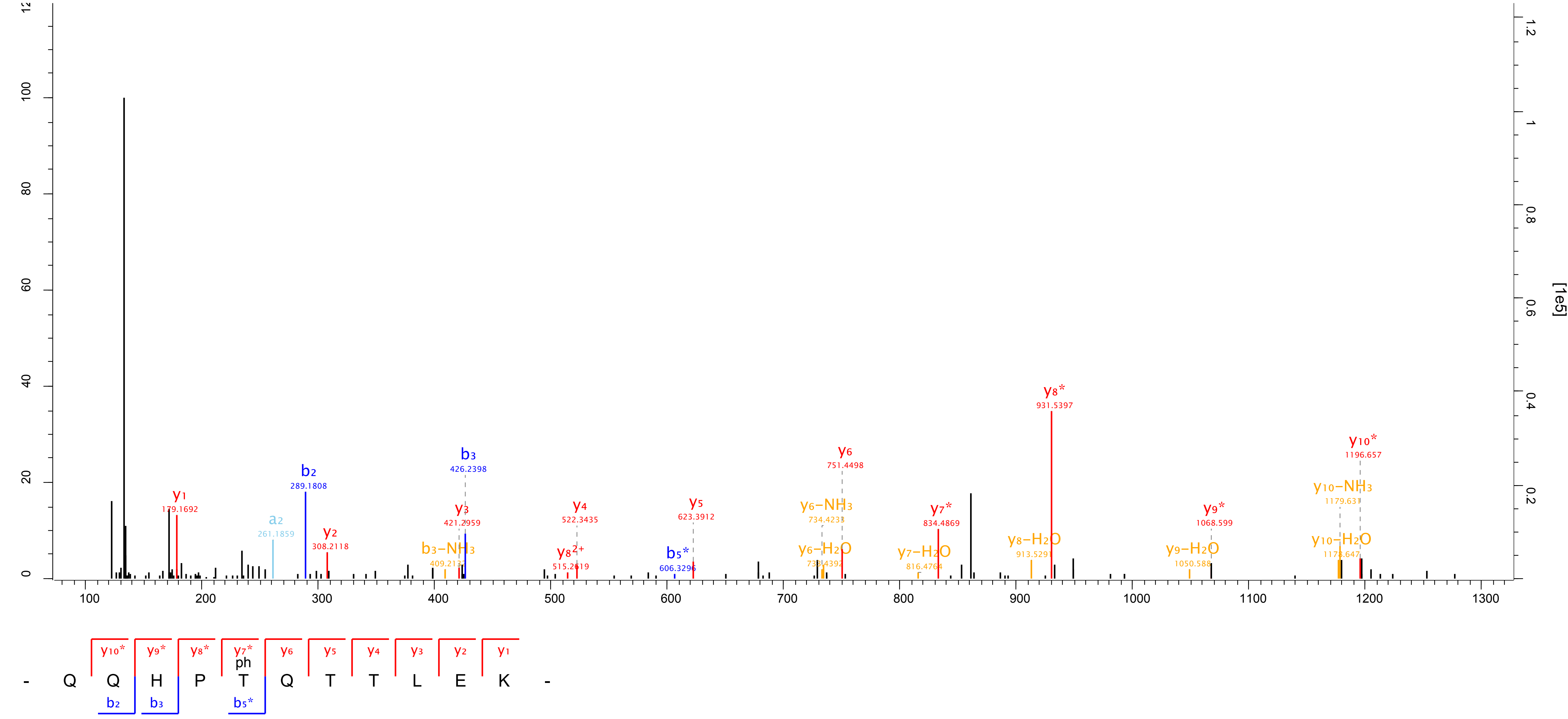

Raw file	Scan	Method	Score	m/z
20141031_PS_Syn6803wt_dimethyl_Mix_Phospho3	5028	FTMS; HCD	49.36	559.29

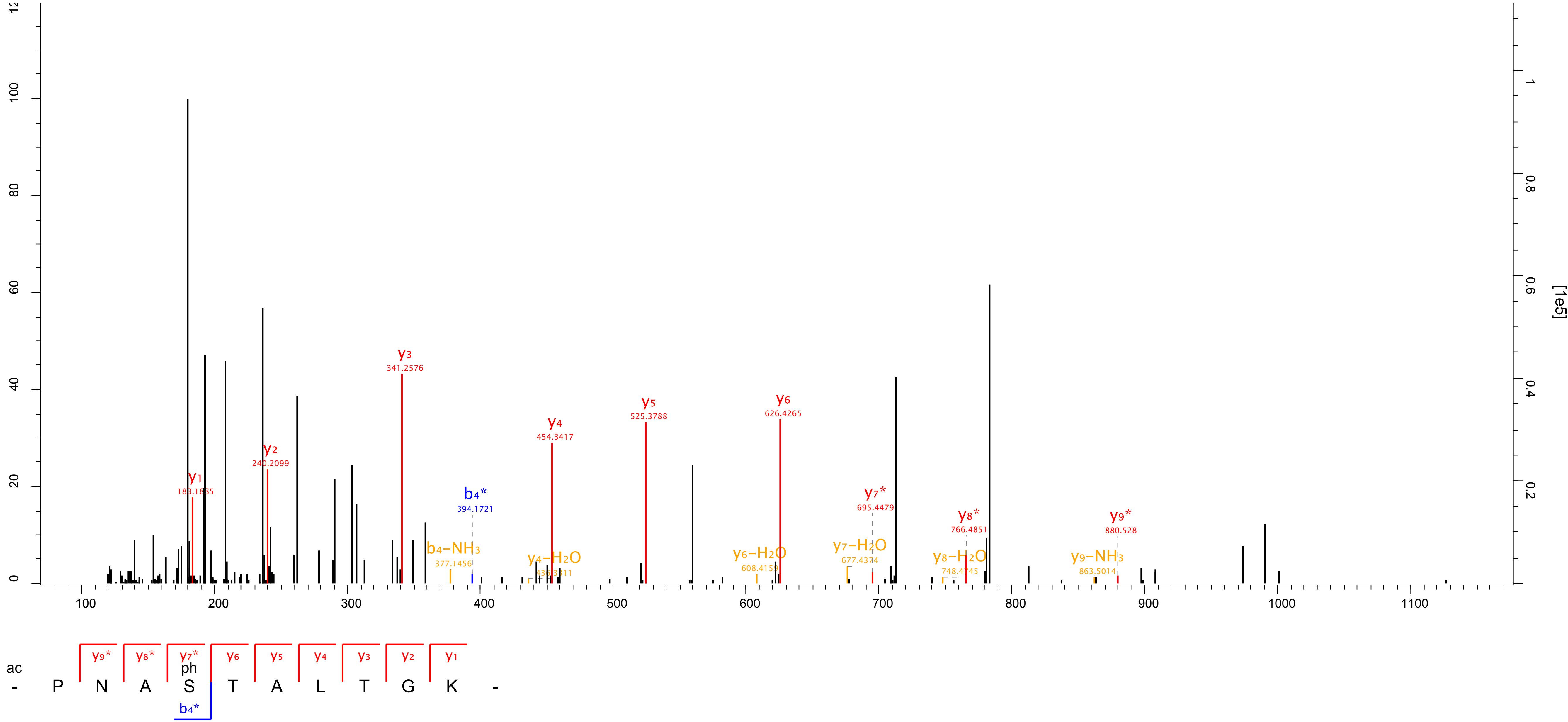

Raw file	Scan	Method	Score	m/z
201417_PS_Syn6803wt_dimethylMix_TiO2_3	5257	FTMS; HCD	113.66	430.73

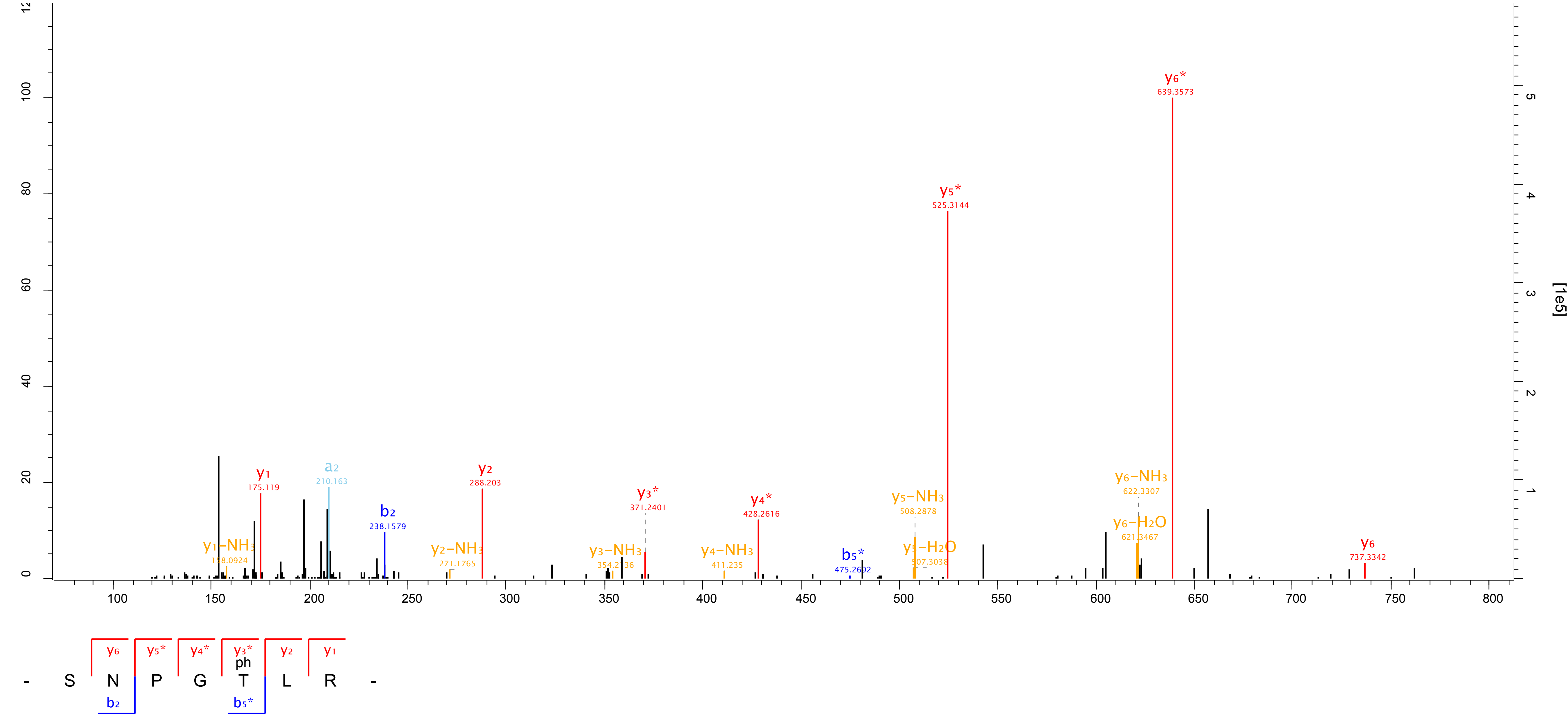

Raw file	Scan	Method	Score	m/z
20141031_PS_Syn6803wt_dimethyl_Mix_Phospho1	5306	FTMS; HCD	50.04	537.37

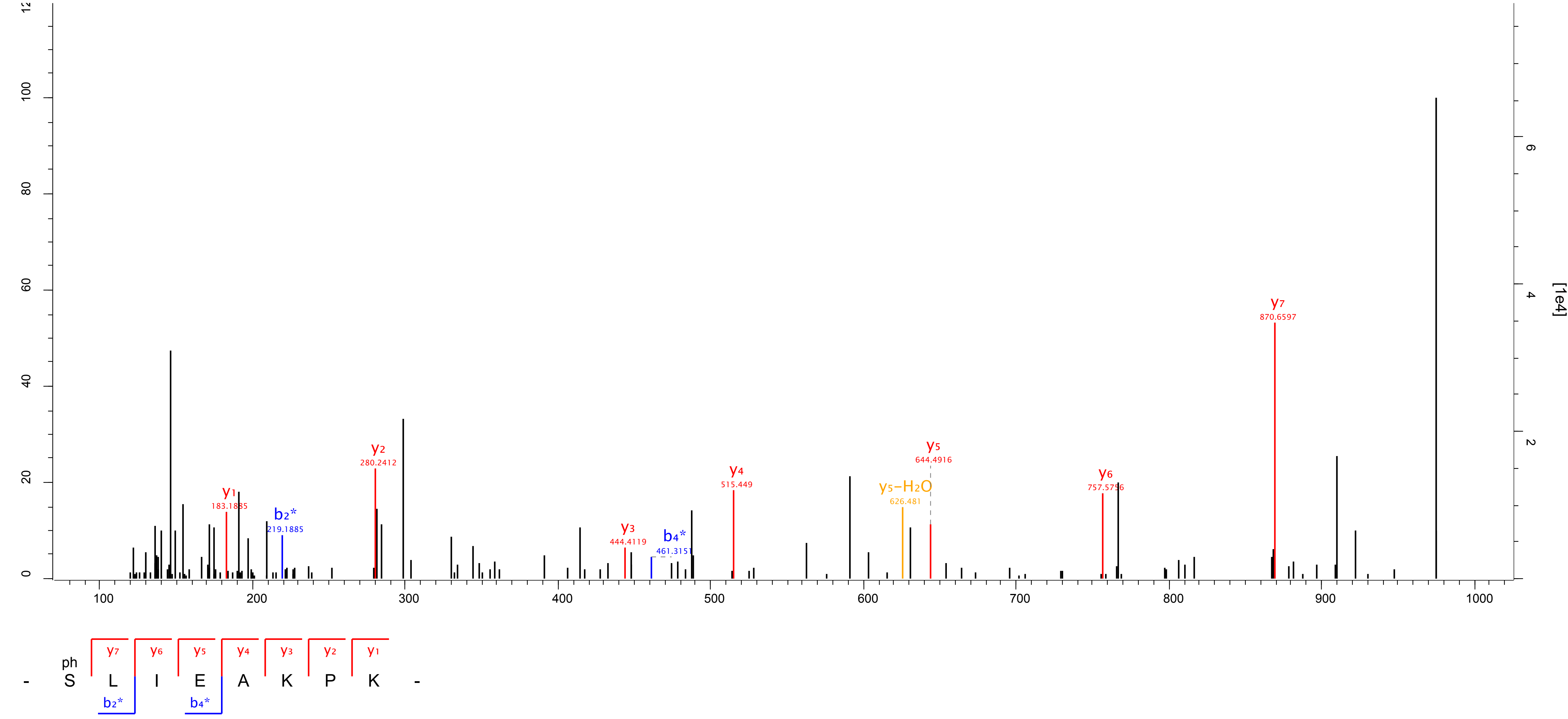

Raw file	Scan	Method	Score	m/z
201417_PS_Syn6803wt_dimethylMix_TiO2_1	5338	FTMS; HCD	78.69	516.94

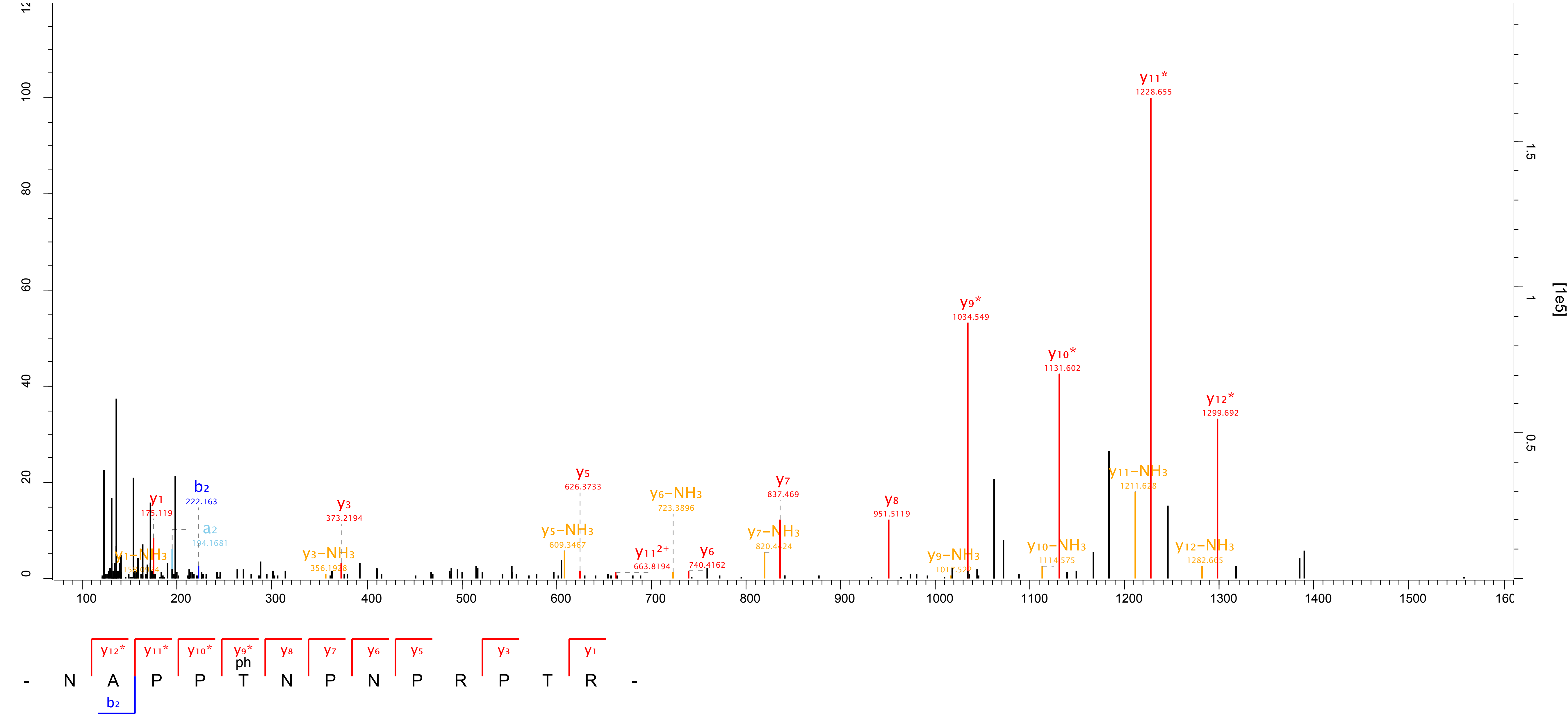

Raw file	Scan	Method	Score	m/z
201417_PS_Syn6803wt_dimethylMix_TiO2_1	5432	FTMS; HCD	62.94	552.31

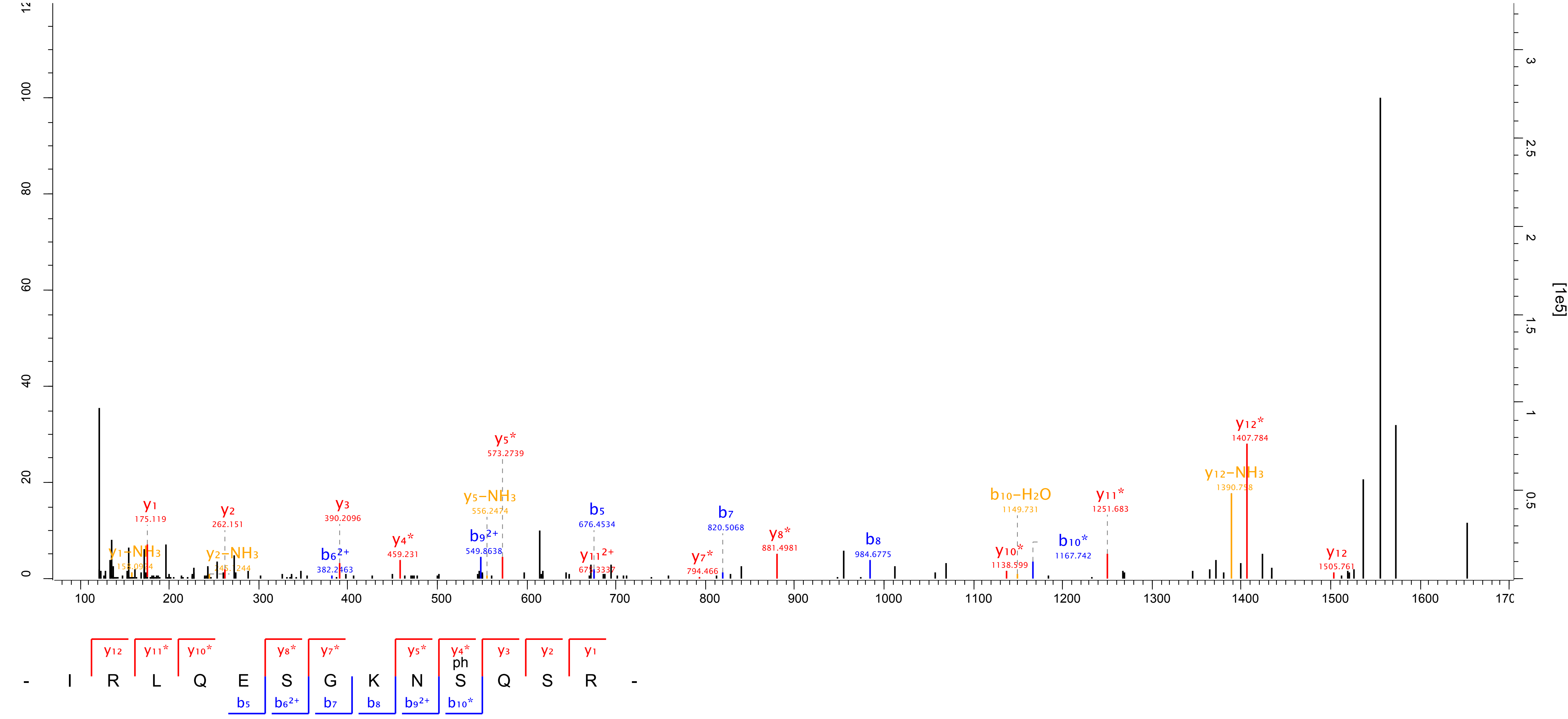

Raw file	Scan	Method	Score	m/z
20141031_PS_Syn6803wt_dimethyl_Mix_Phospho1	5841	FTMS; HCD	51.03	484.93

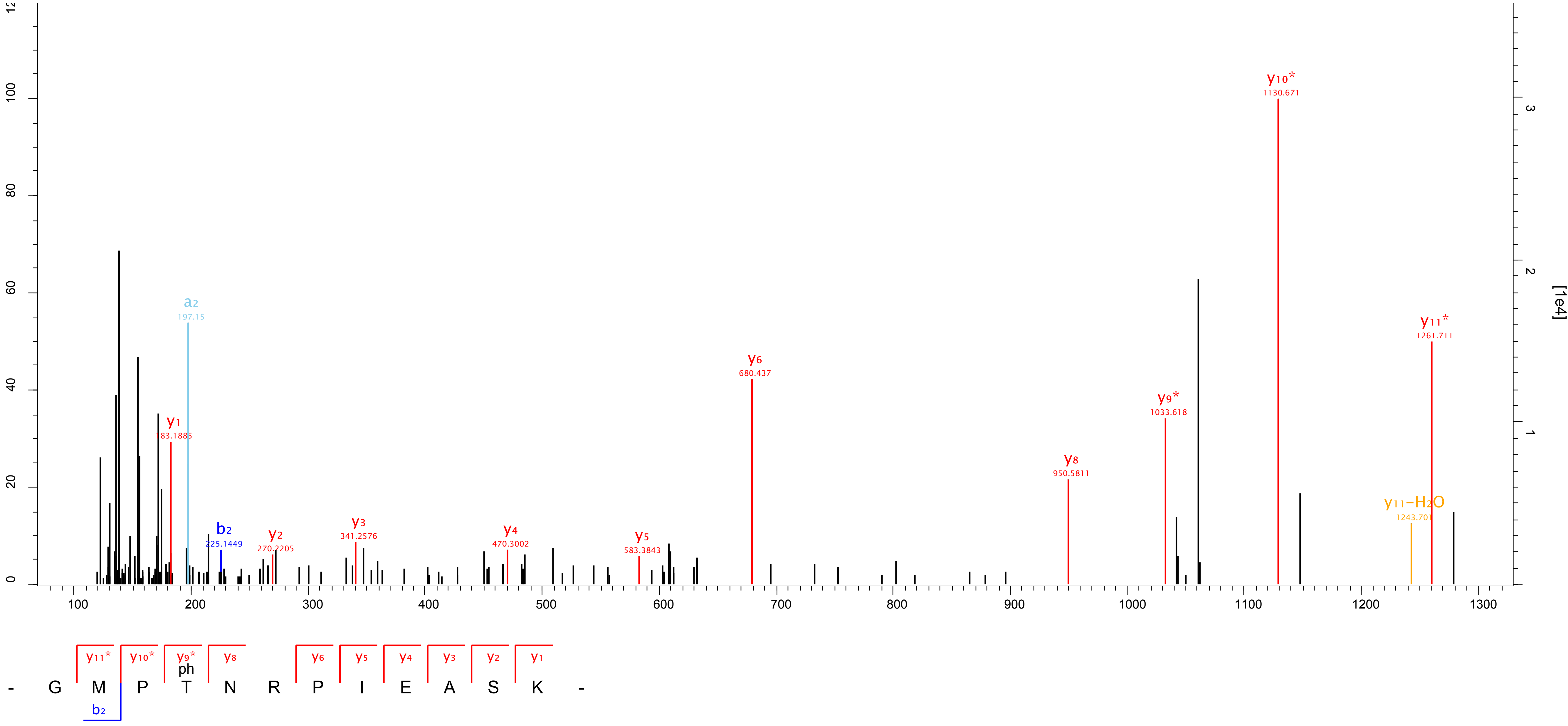

Raw file	Scan	Method	Score	m/z
20141031_PS_Syn6803wt_dimethyl_Mix_Phospho3	5881	FTMS; HCD	78.69	565.63

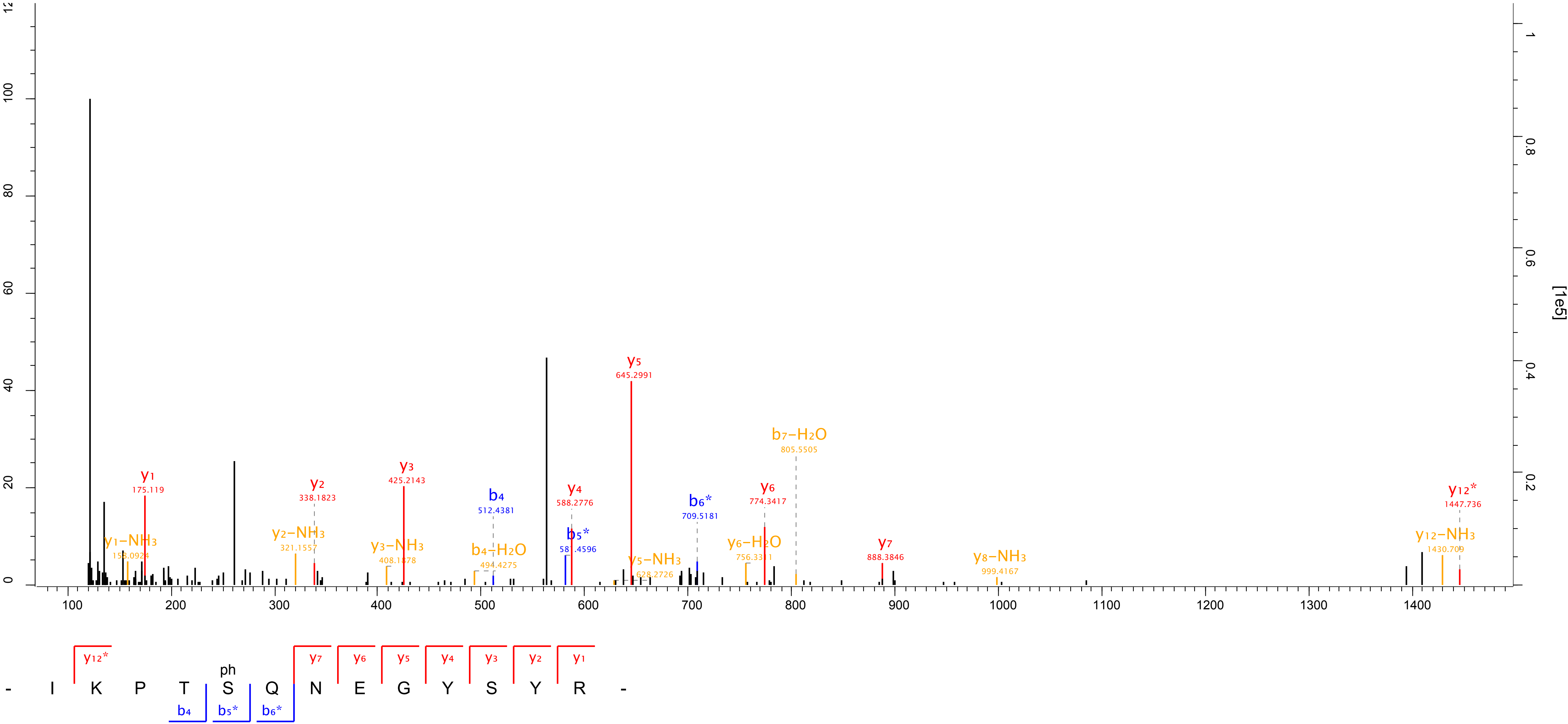

Raw file	Scan	Method	Score	m/z
20150122_PS_Syn_DimethylMix_Rep2_Phospho08	5889	FTMS; HCD	97.81	481.25

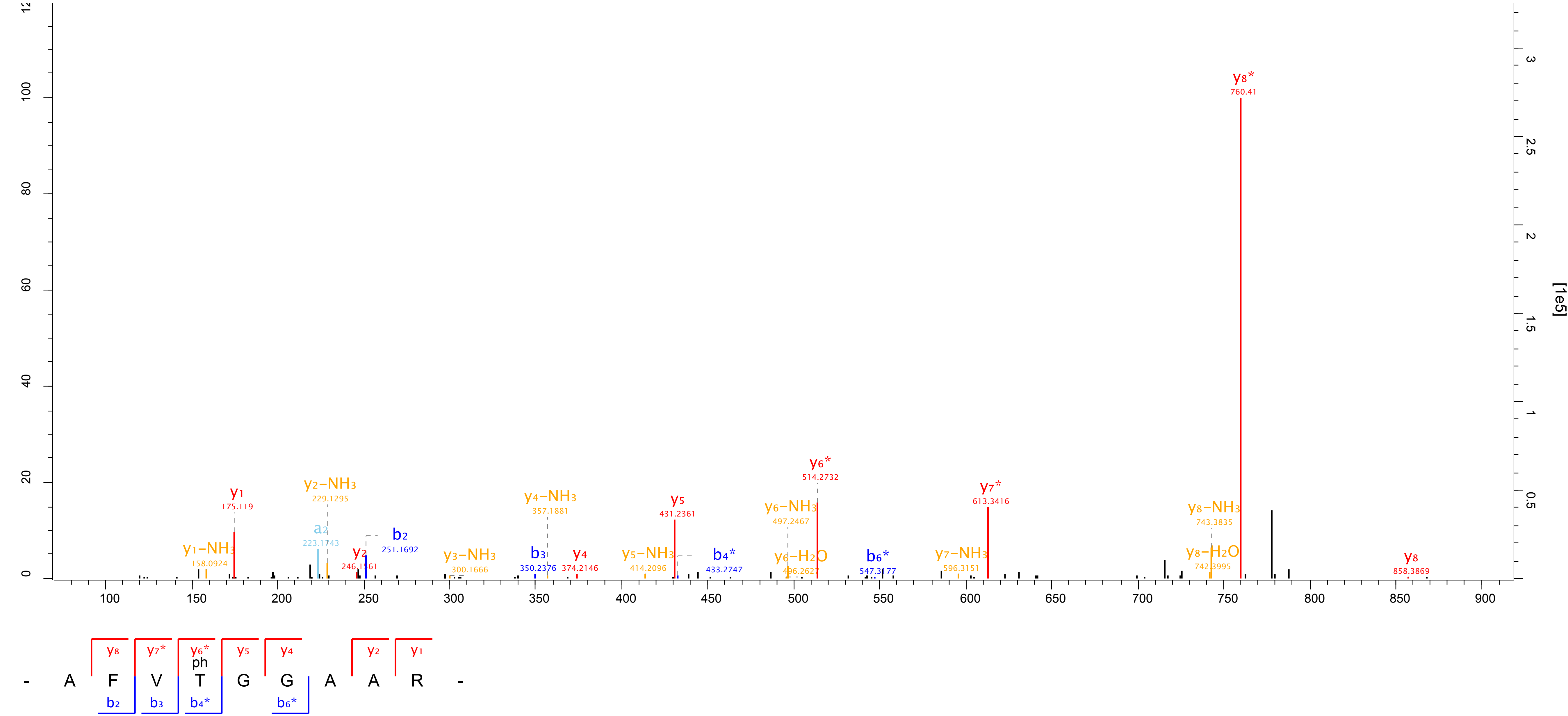

Raw file	Scan	Method	Score	m/z
201417_PS_Syn6803wt_dimethylMix_TiO2_2	6486	FTMS; HCD	84.48	570.25

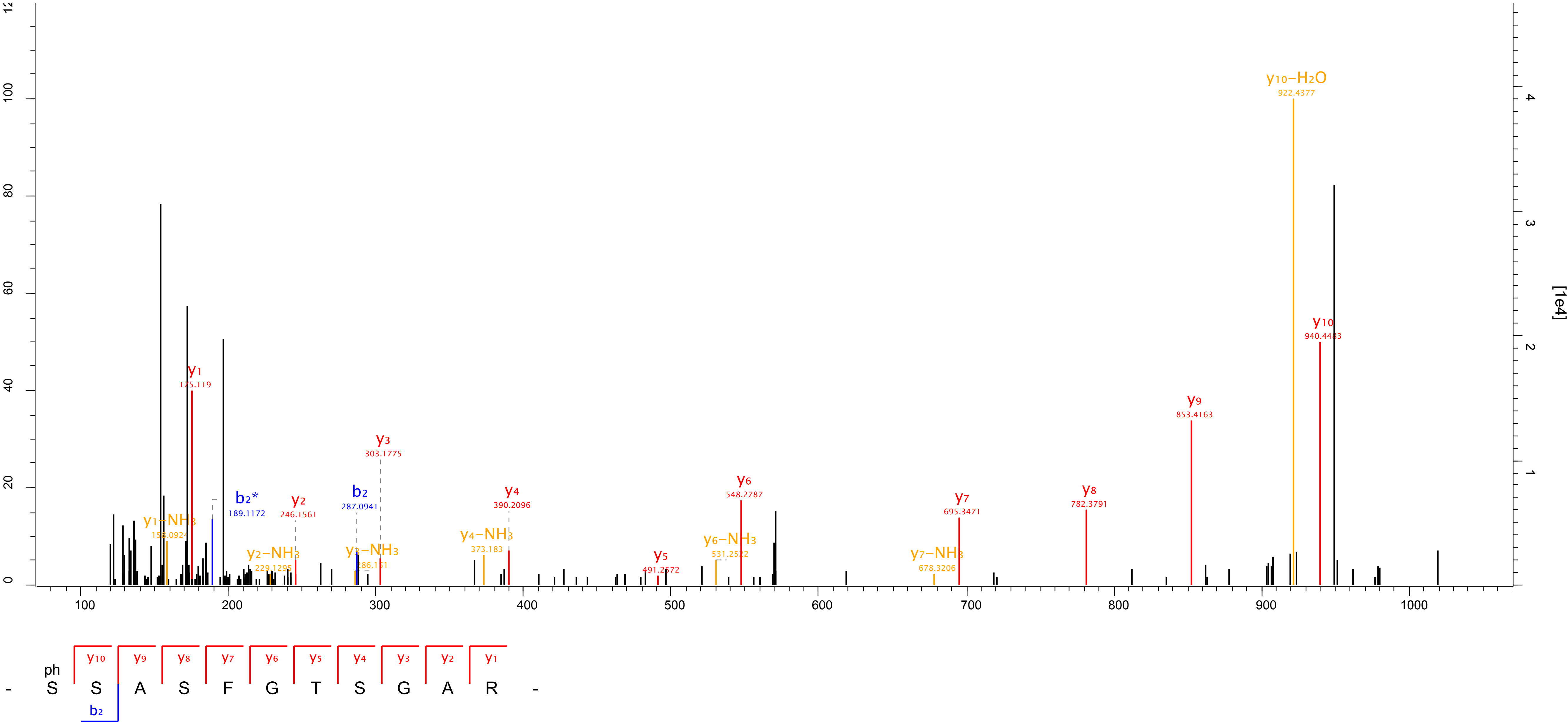

Raw file	Scan	Method	Score	m/z
201417_PS_Syn6803wt_dimethylMix_TiO2_1	6497	FTMS; HCD	127.18	735.37

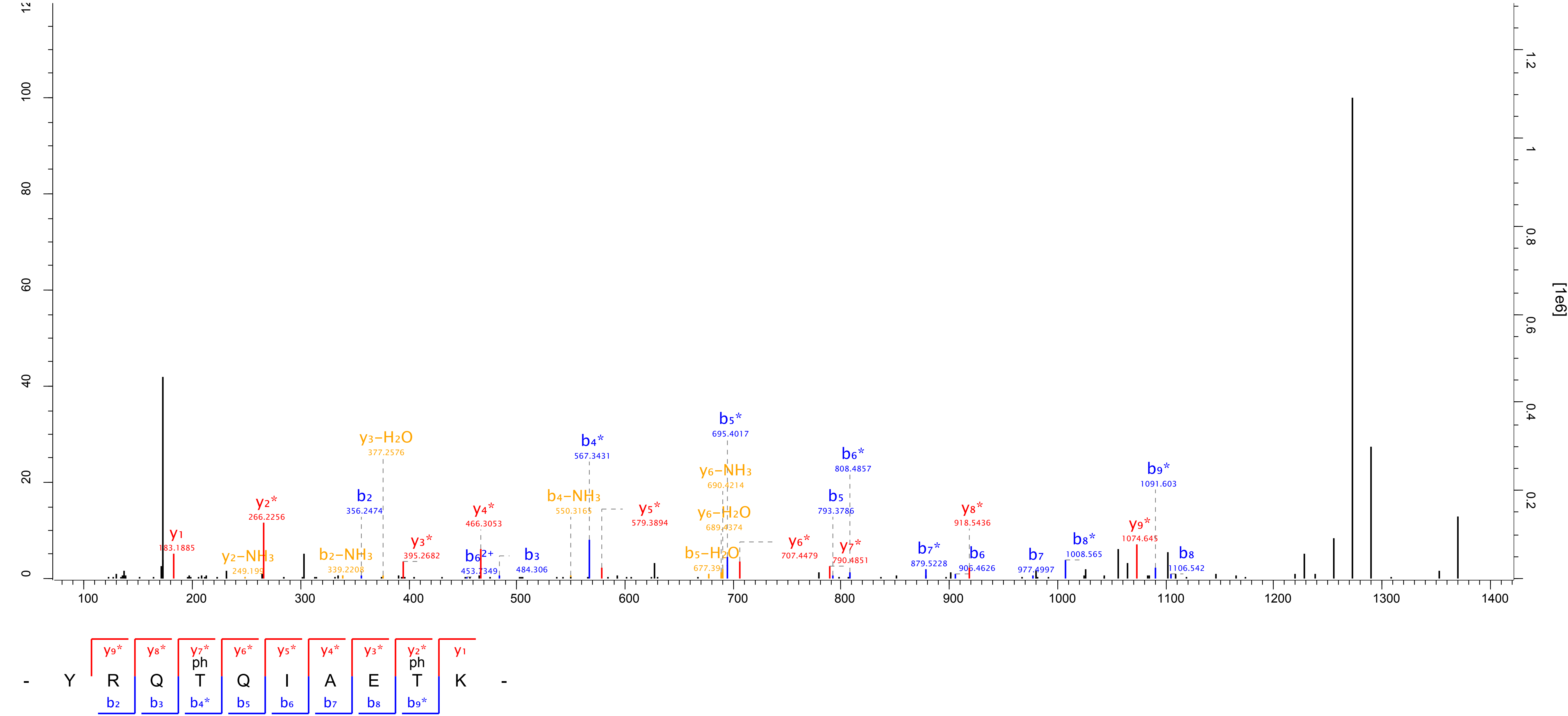

Raw file	Scan	Method	Score	m/z
20141031_PS_Syn6803wt_dimethyl_Mix_Phospho4	6516	FTMS; HCD	88.08	571.91

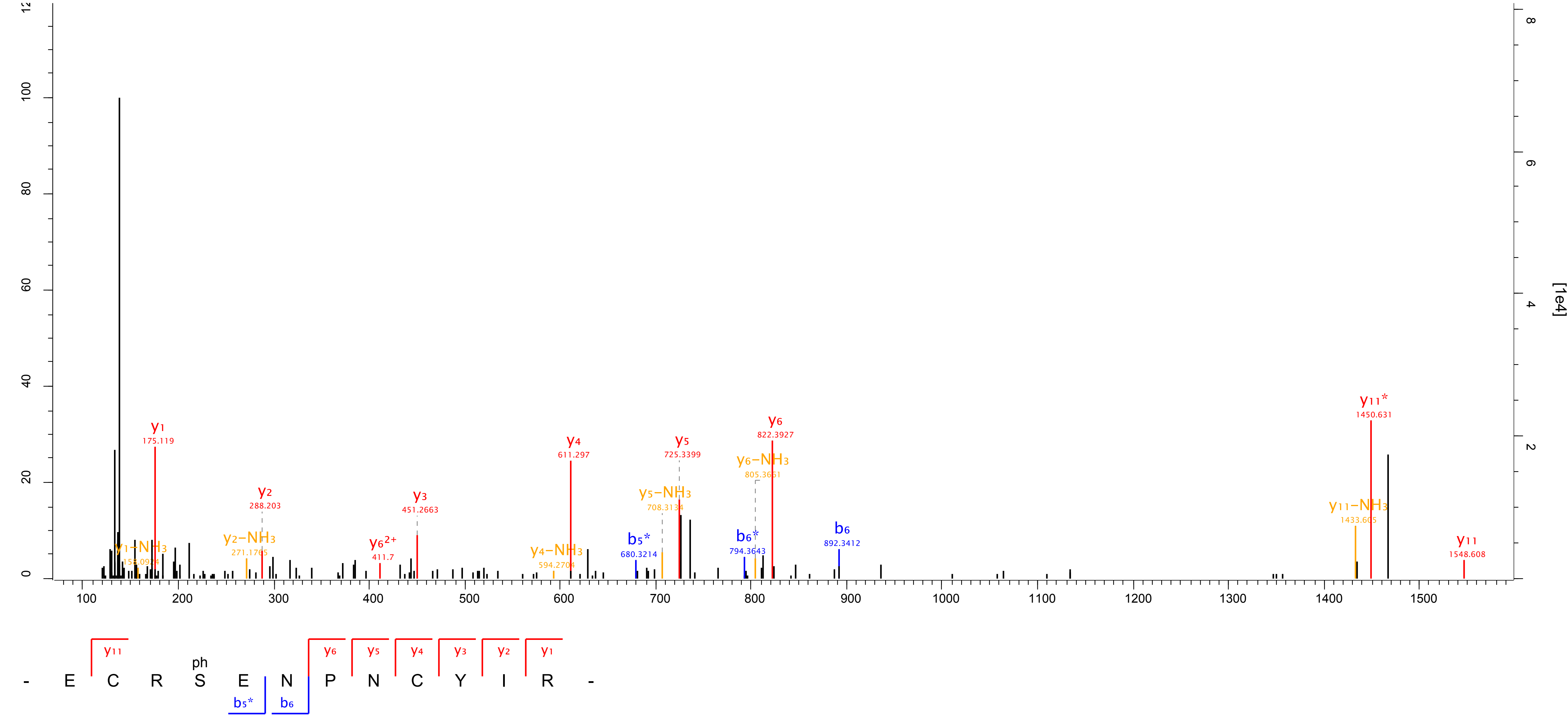

Raw file	Scan	Method	Score	m/z
20150122_PS_Syn_DimethylMix_Rep1_Phospho02	6680	FTMS; HCD	183.33	431.22

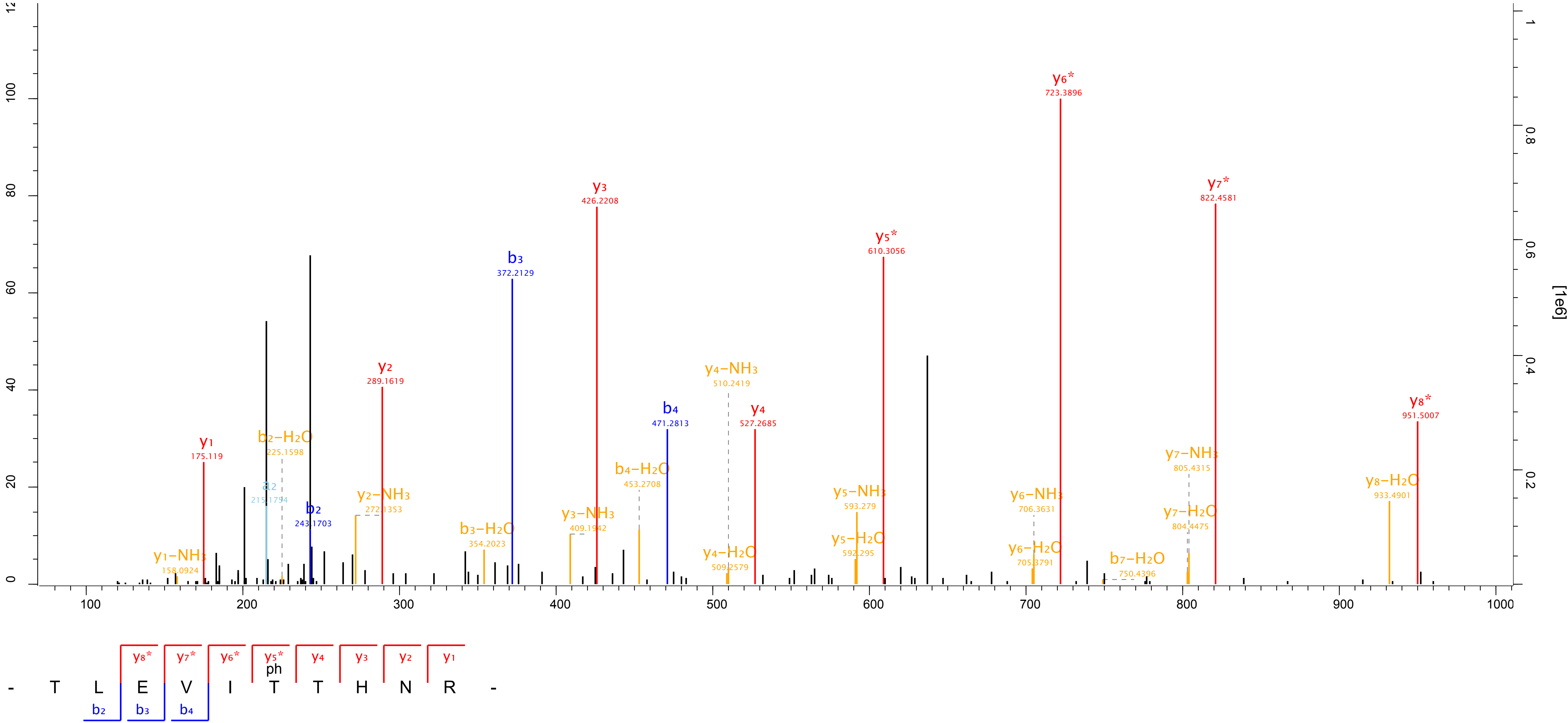

Raw file	Scan	Method	Score	m/z
20150122_PS_Syn_DimethylMix_Rep1_Phospho01	6755	FTMS; HCD	115.8	737.88

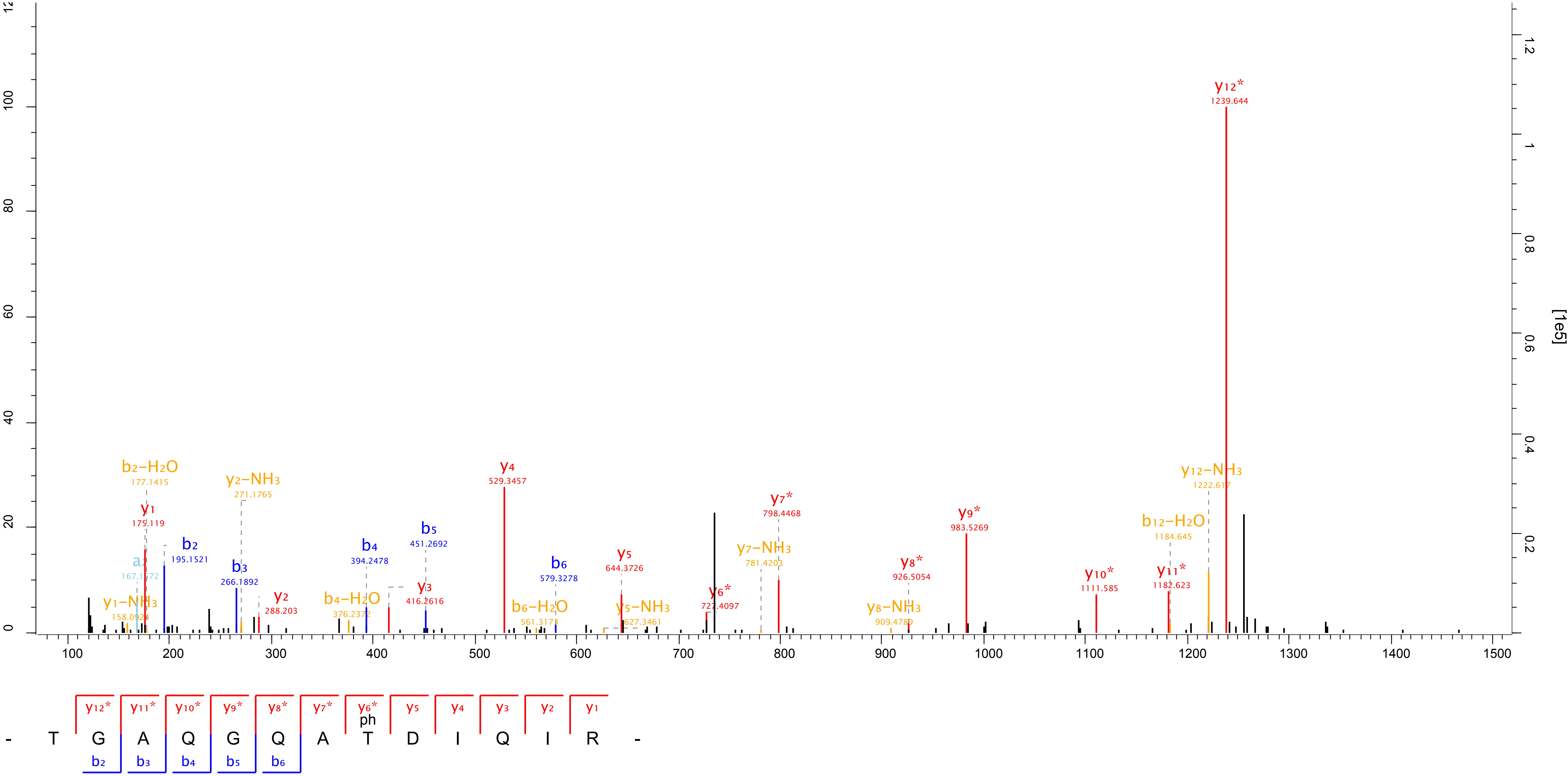

Raw file	Scan	Method	Score	m/z
20141031_PS_Syn6803wt_dimethyl_Mix_Phospho5	6805	FTMS; HCD	64.45	483

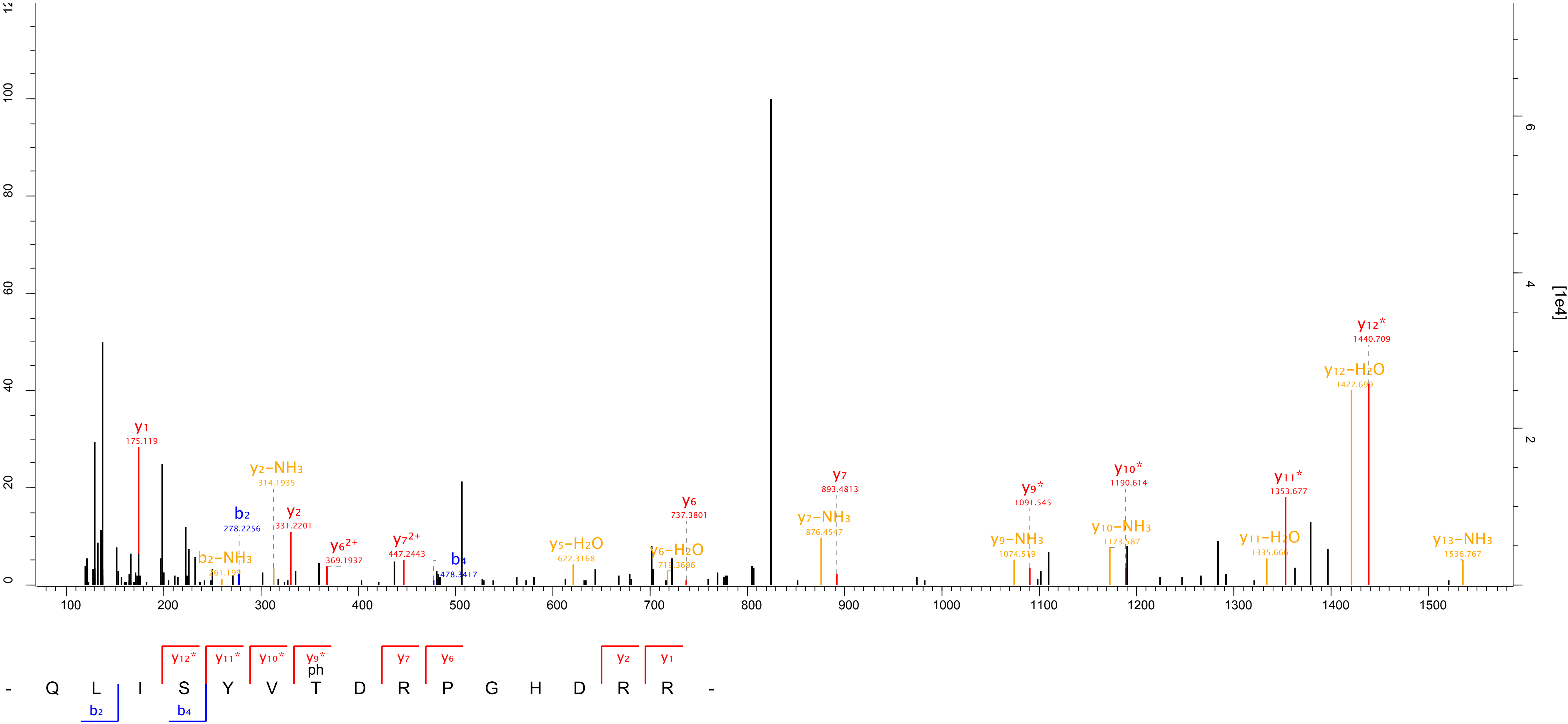

Raw file	Scan	Method	Score	m/z
20141031_PS_Syn6803wt_dimethyl_Mix_Phospho4	6836	FTMS; HCD	100.93	489.28

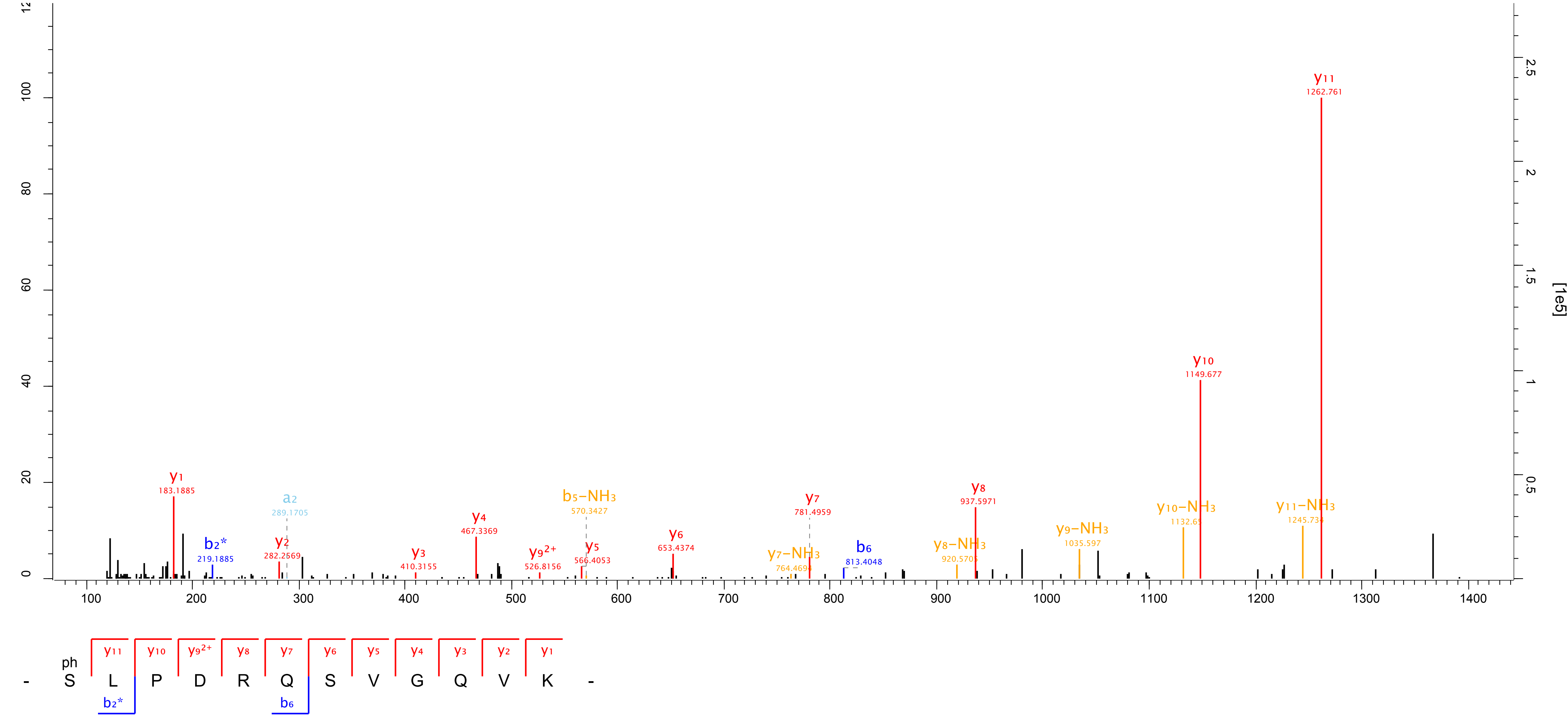

Raw file	Scan	Method	Score	m/z
20141031_PS_Syn6803wt_dimethyl_Mix_Phospho1	6984	FTMS; HCD	116.6	570.83

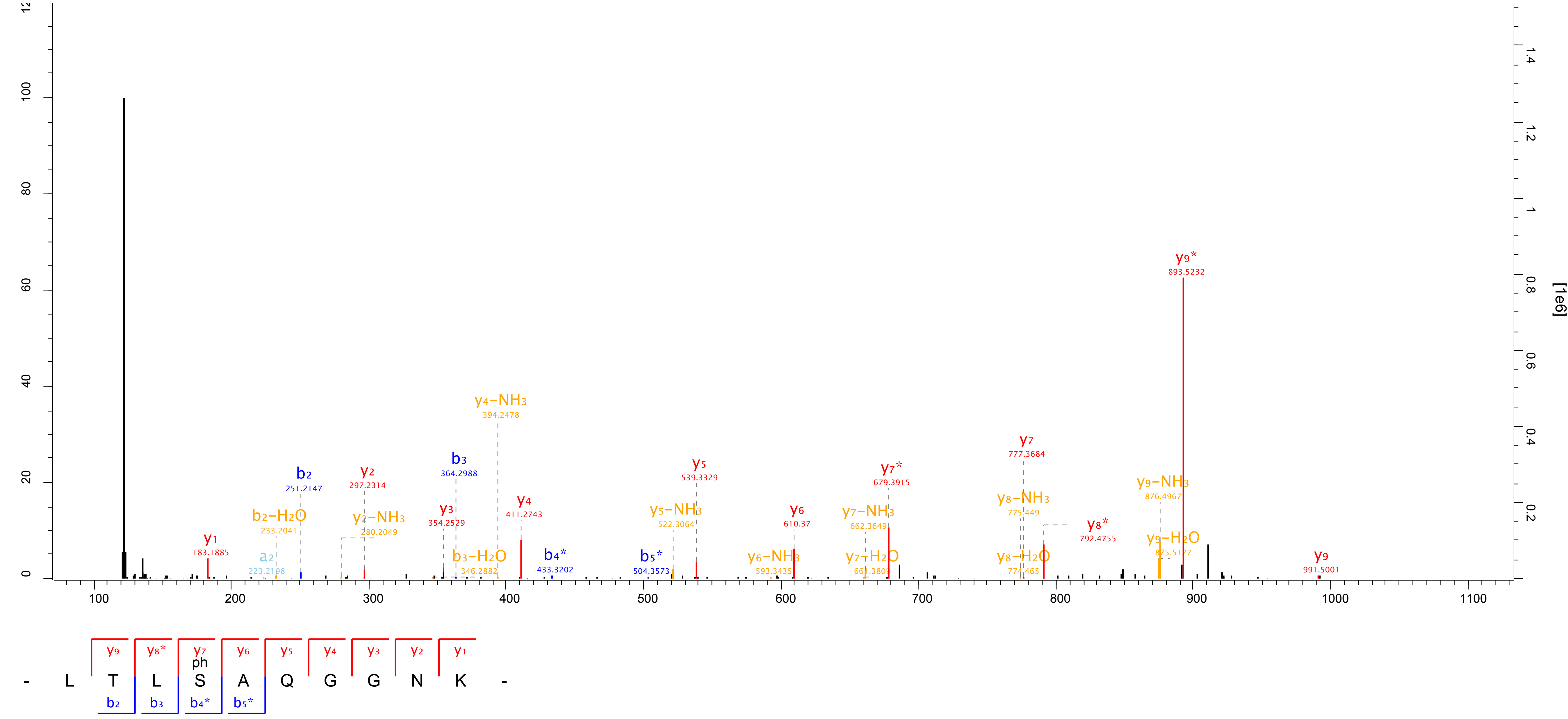

Raw file	Scan	Method	Score	m/z
20141031_PS_Syn6803wt_dimethyl_Mix_Phospho7	7202	FTMS; HCD	70.78	762.82

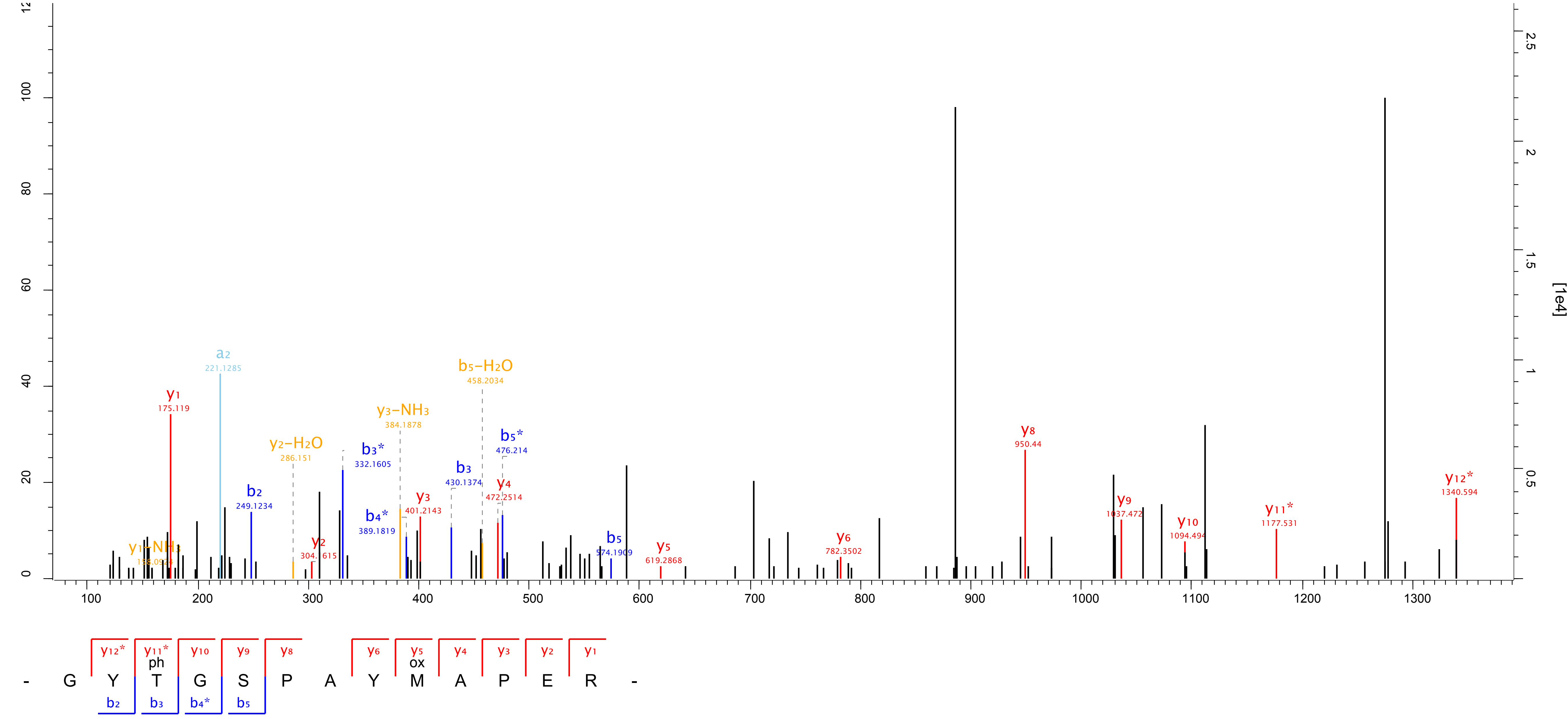

Raw file	Scan	Method	Score	m/z
20150122_PS_Syn_DimethylMix_Rep1_Phospho02	7242	FTMS; HCD	181.98	810.89

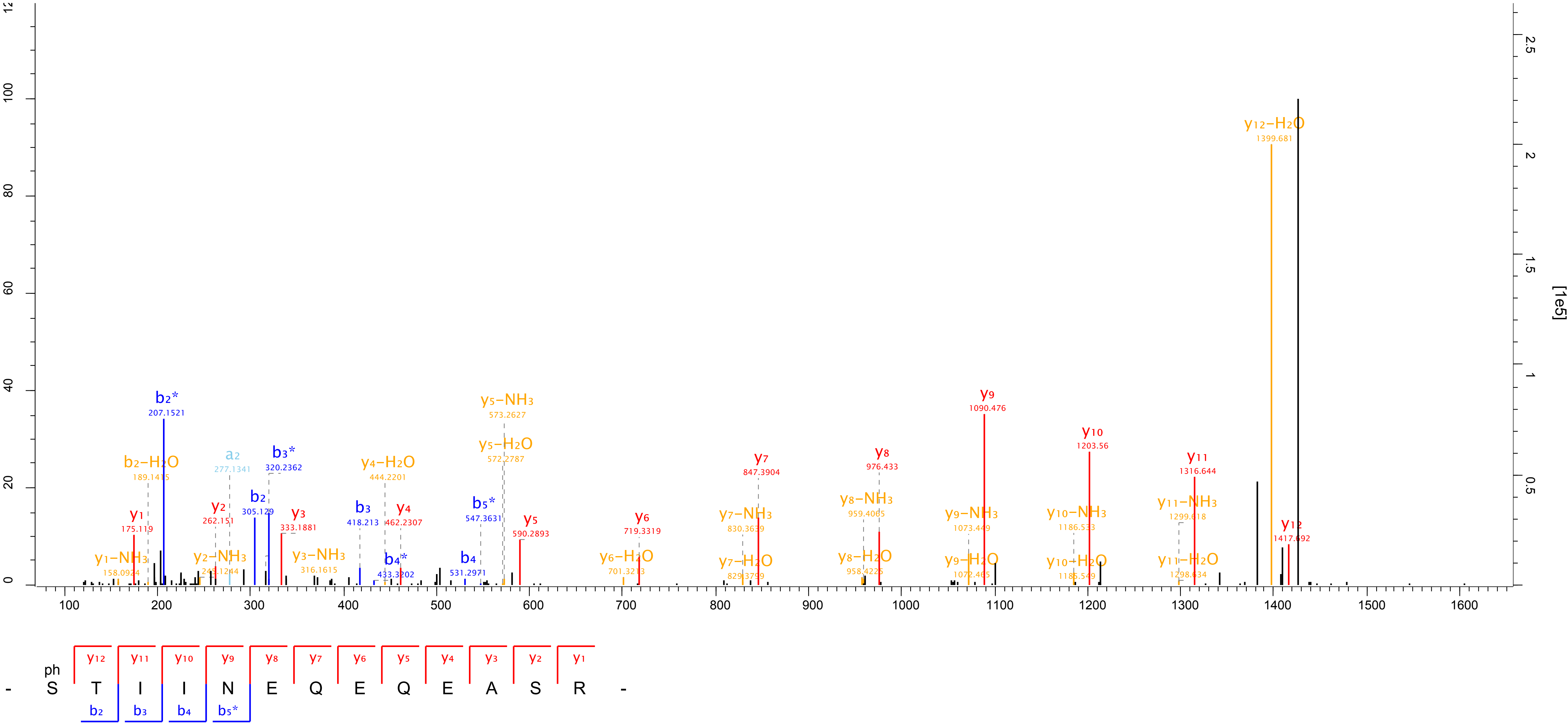

Raw file	Scan	Method	Score	m/z
201417_PS_Syn6803wt_dimethylMix_TiO2_1	7309	FTMS; HCD	95.2	760.35

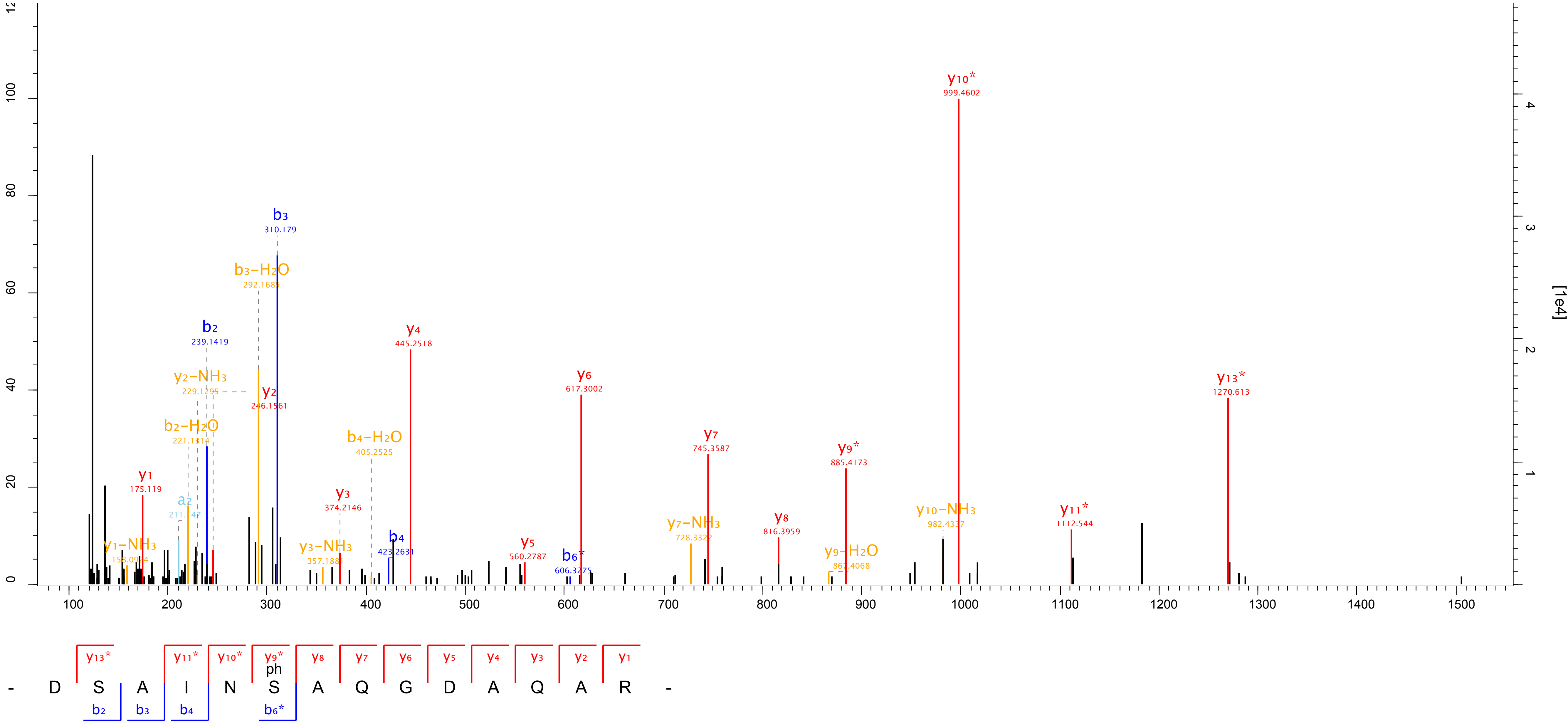

Raw file	Scan	Method	Score	m/z
20141031_PS_Syn6803wt_dimethyl_Mix_Phospho1	7444	FTMS; HCD	68.45	761.68

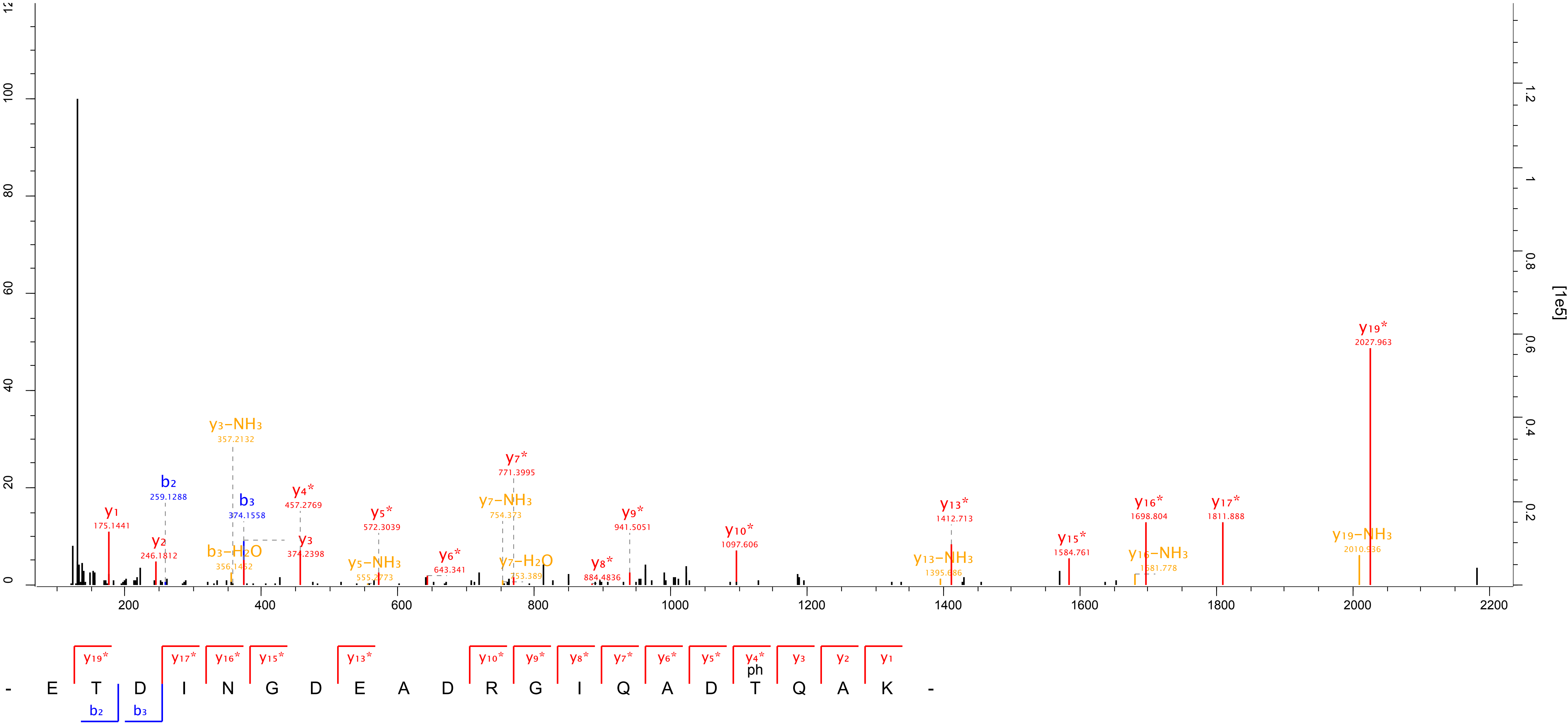

Raw file	Scan	Method	Score	m/z
20141031_PS_Syn6803wt_dimethyl_Mix_Phospho2	7539	FTMS; HCD	158.17	551.98

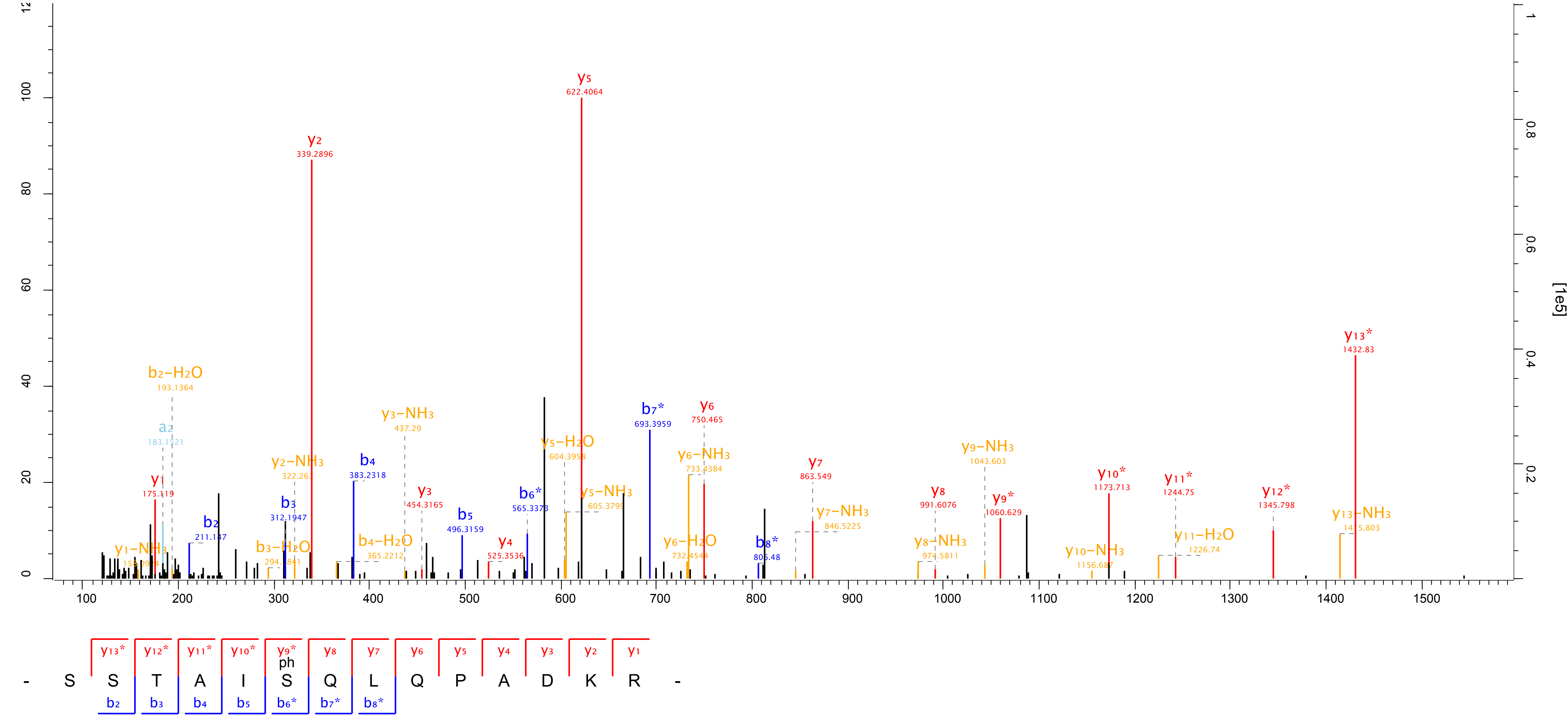

Raw file	Scan	Method	Score	m/z
20141031_PS_Syn6803wt_dimethyl_Mix_Phospho8	7903	FTMS; HCD	99.28	591.36

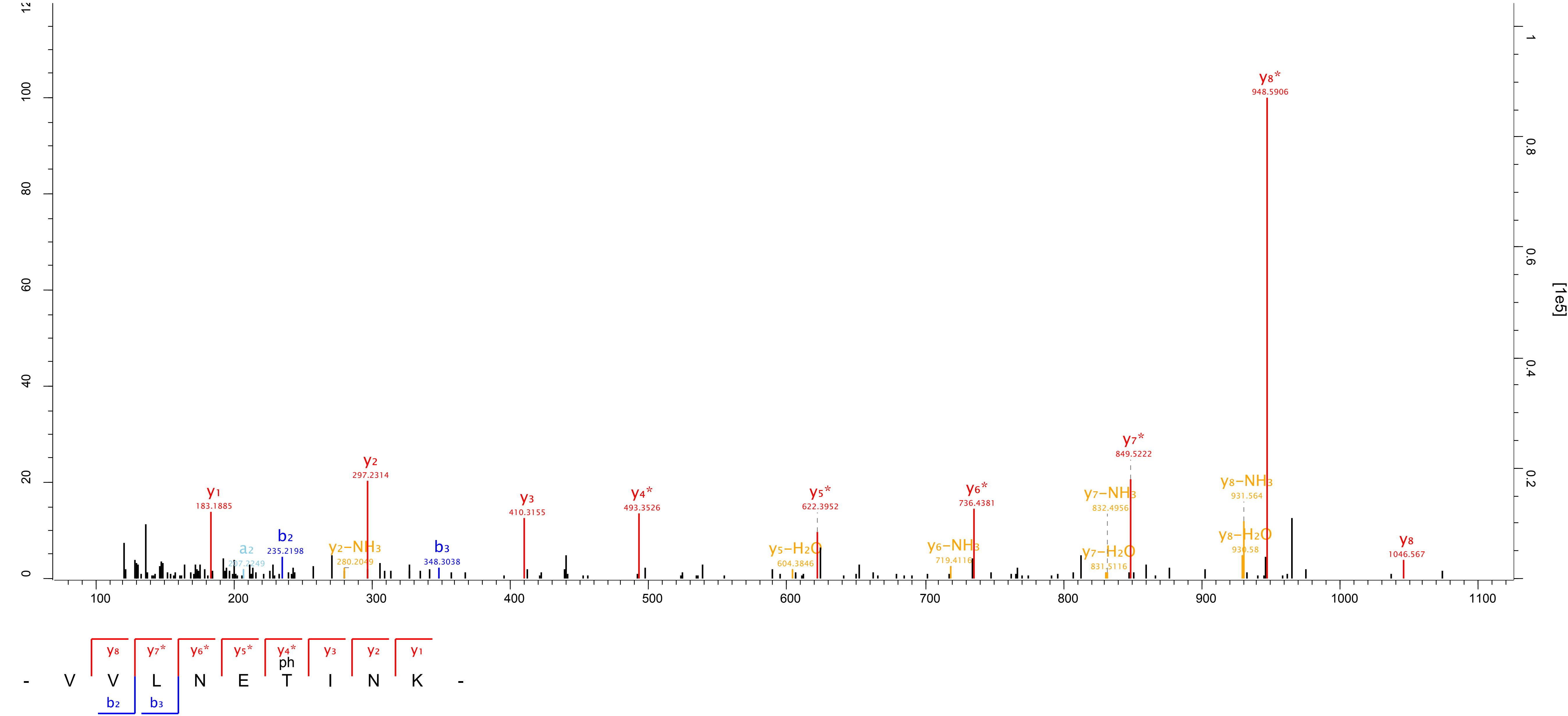

Raw file	Scan	Method	Score	m/z
20141031_PS_Syn6803wt_dimethyl_Mix_Phospho7	8043	FTMS; HCD	54.54	547.78

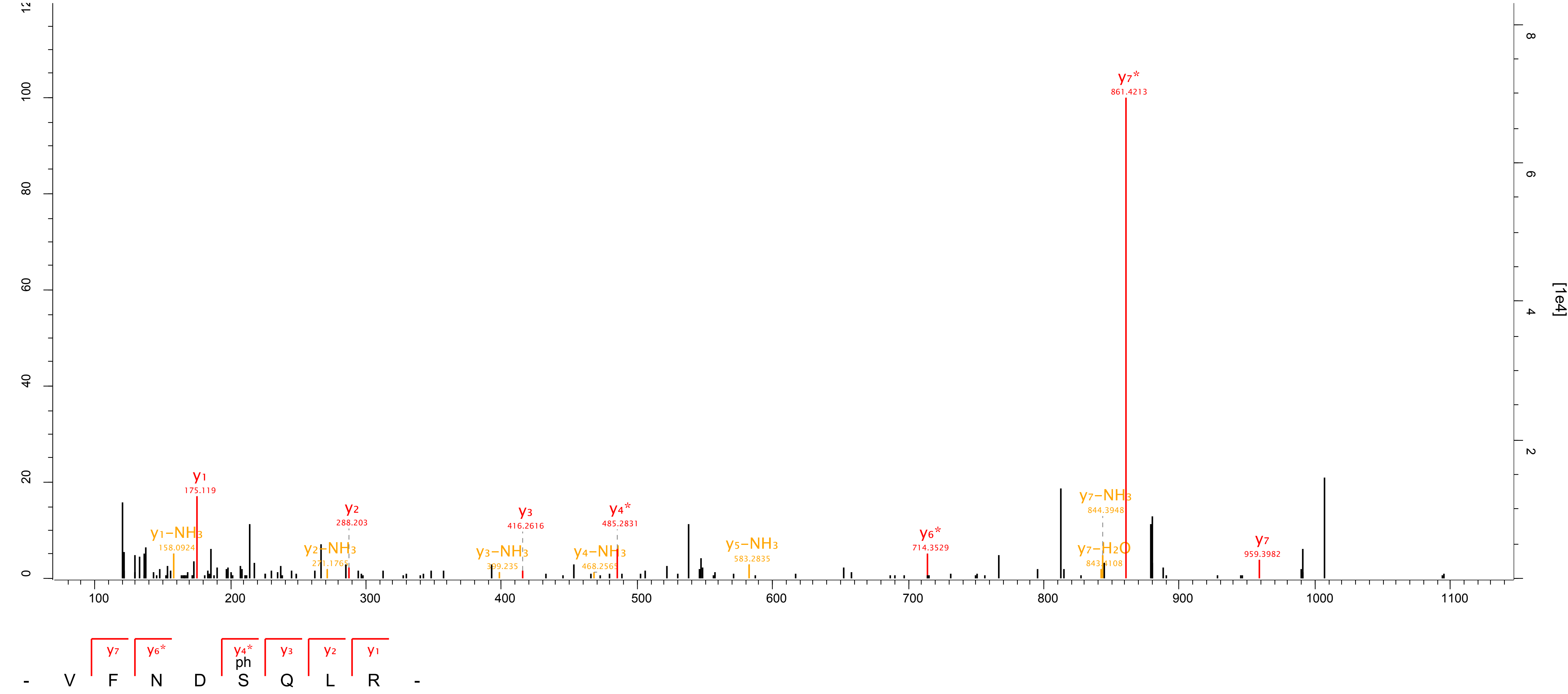

Raw file	Scan	Method	Score	m/z
20141031_PS_Syn6803wt_dimethyl_Mix_Phospho4	8188	FTMS; HCD	69.7	597.3

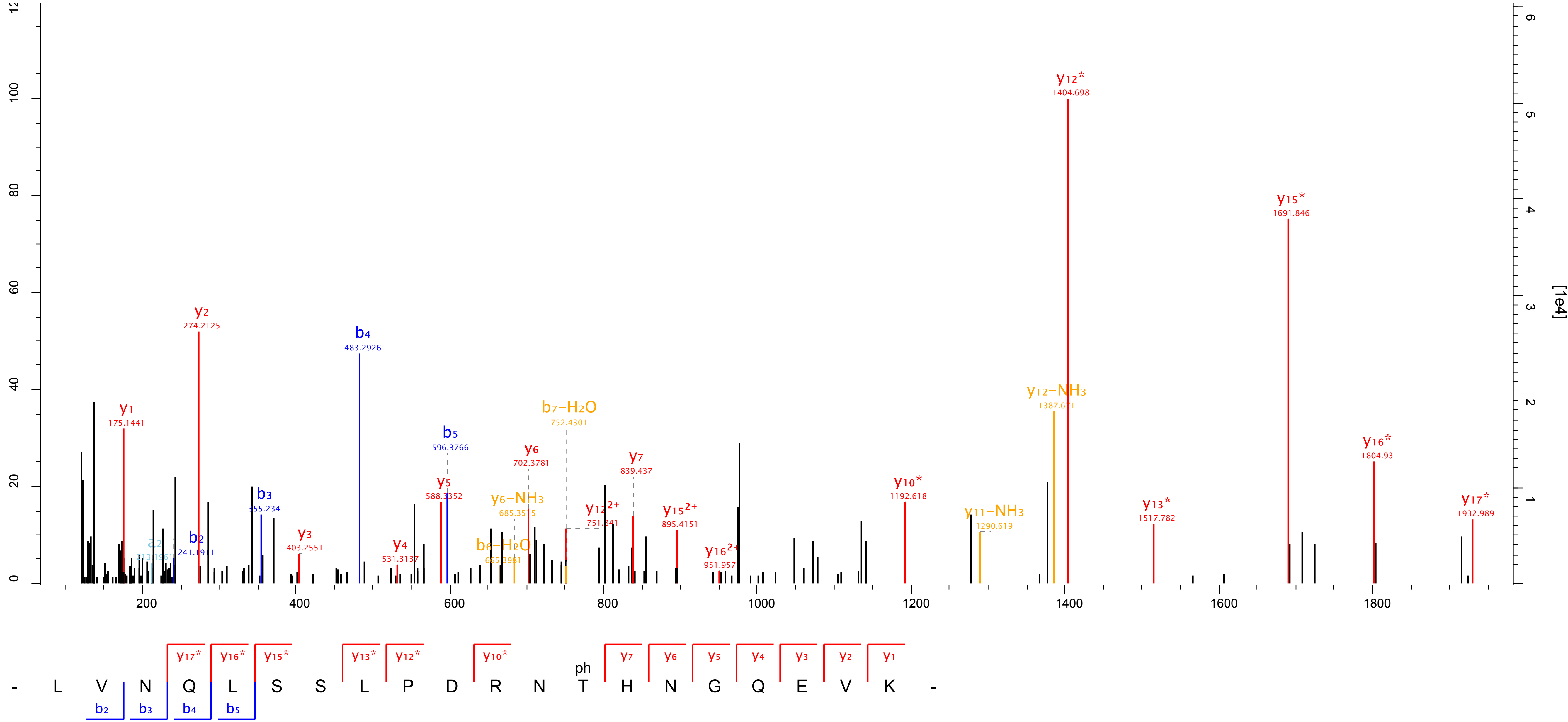

Raw file	Scan	Method	Score	m/z
20150122_PS_Syn_DimethylMix_Rep2_Phospho08	8222	FTMS; HCD	104.94	688.36

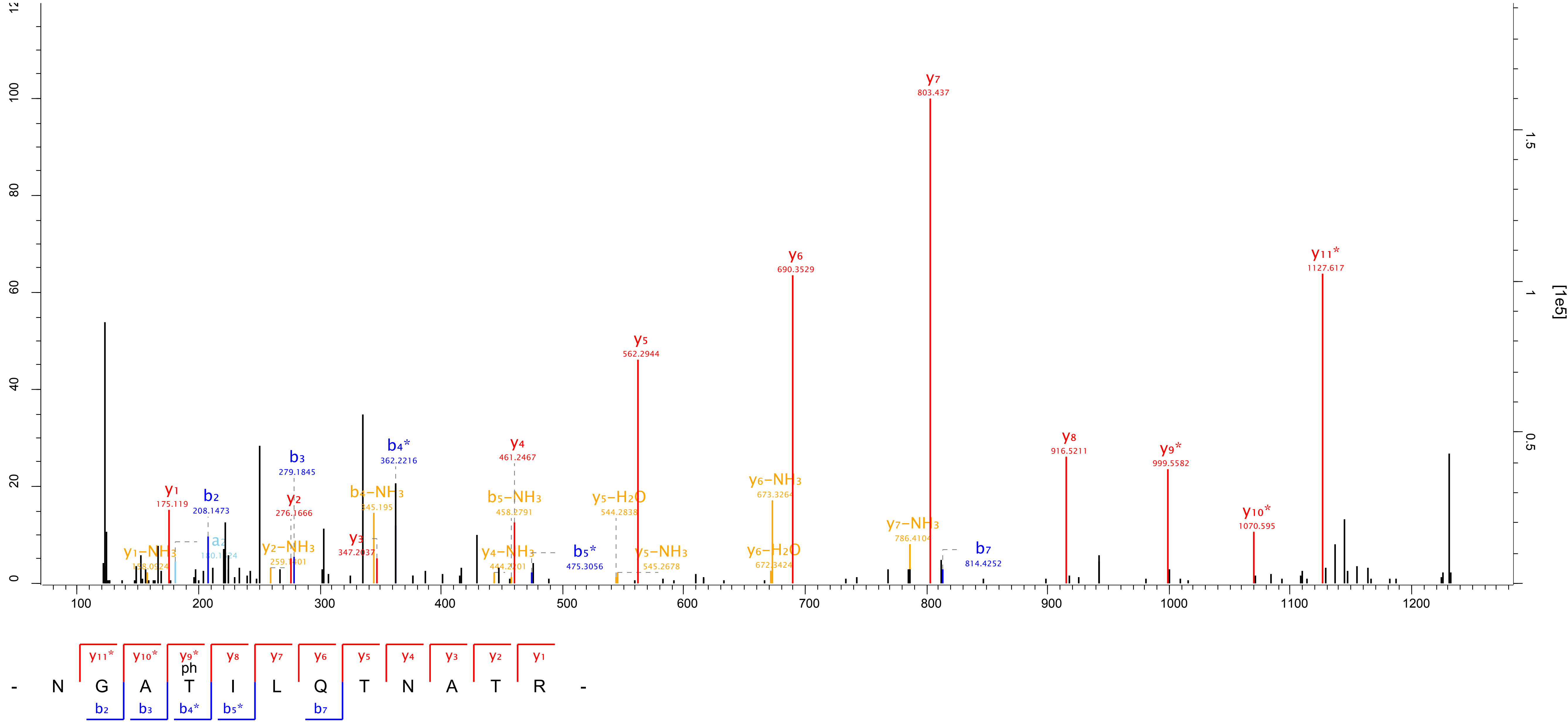

Raw file	Scan	Method	Score	m/z
201417_PS_Syn6803wt_dimethylMix_TiO2_1	8244	FTMS; HCD	84.97	755.71

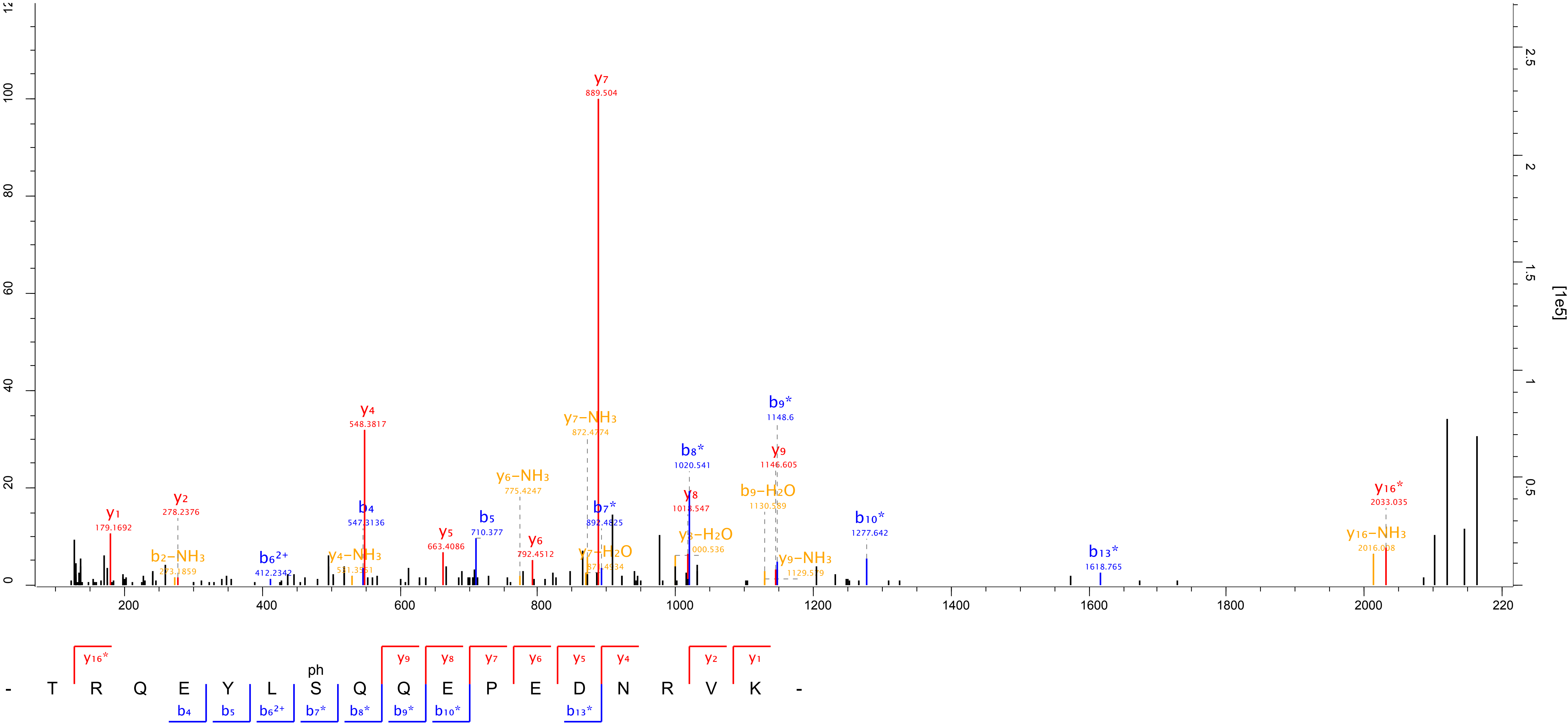

Raw file	Scan	Method	Score	m/z
201417_PS_Syn6803wt_dimethylMix_TiO2_1	8393	FTMS; HCD	88.56	754.32

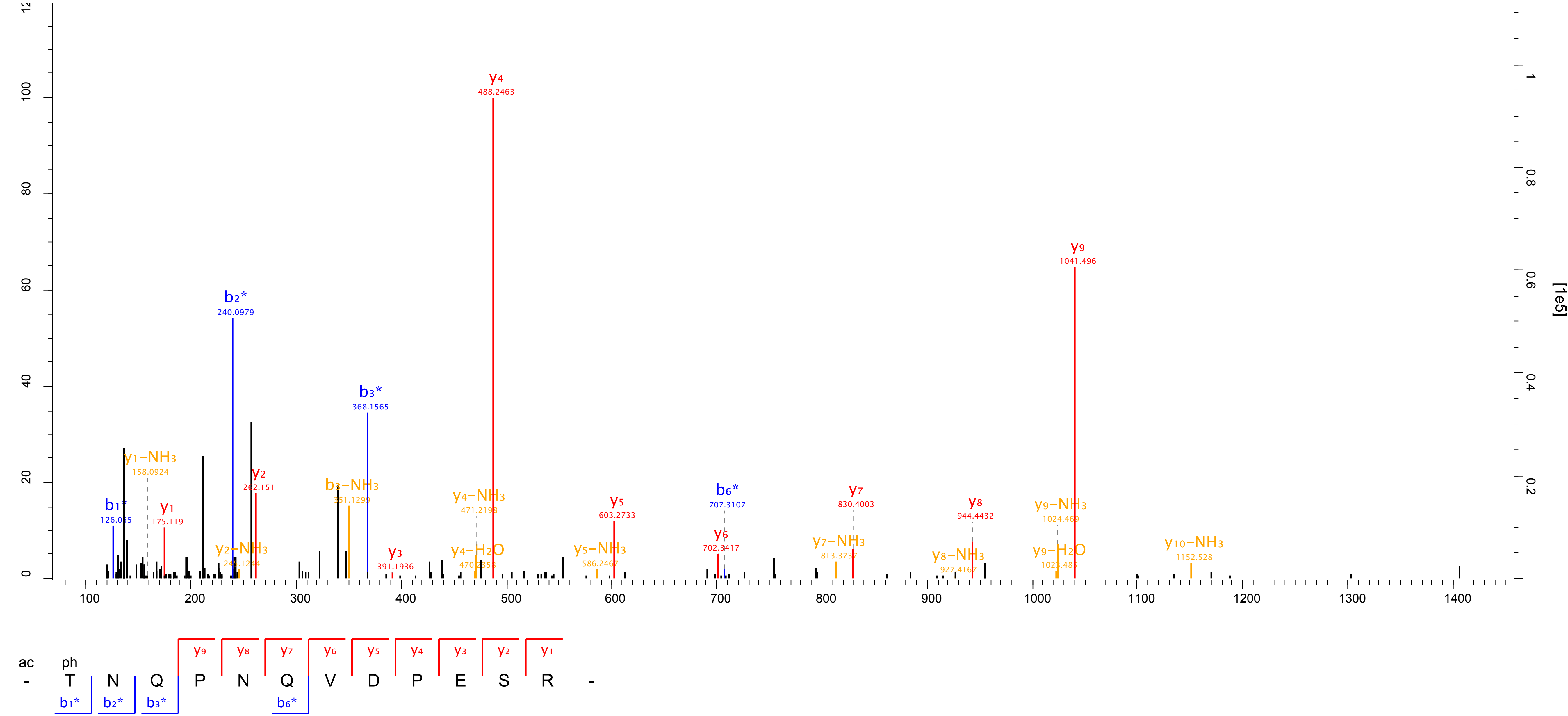

Raw file	Scan	Method	Score	m/z
20150122_PS_Syn_DimethylMix_Rep1_Phospho08	8430	FTMS; HCD	79.65	765.36

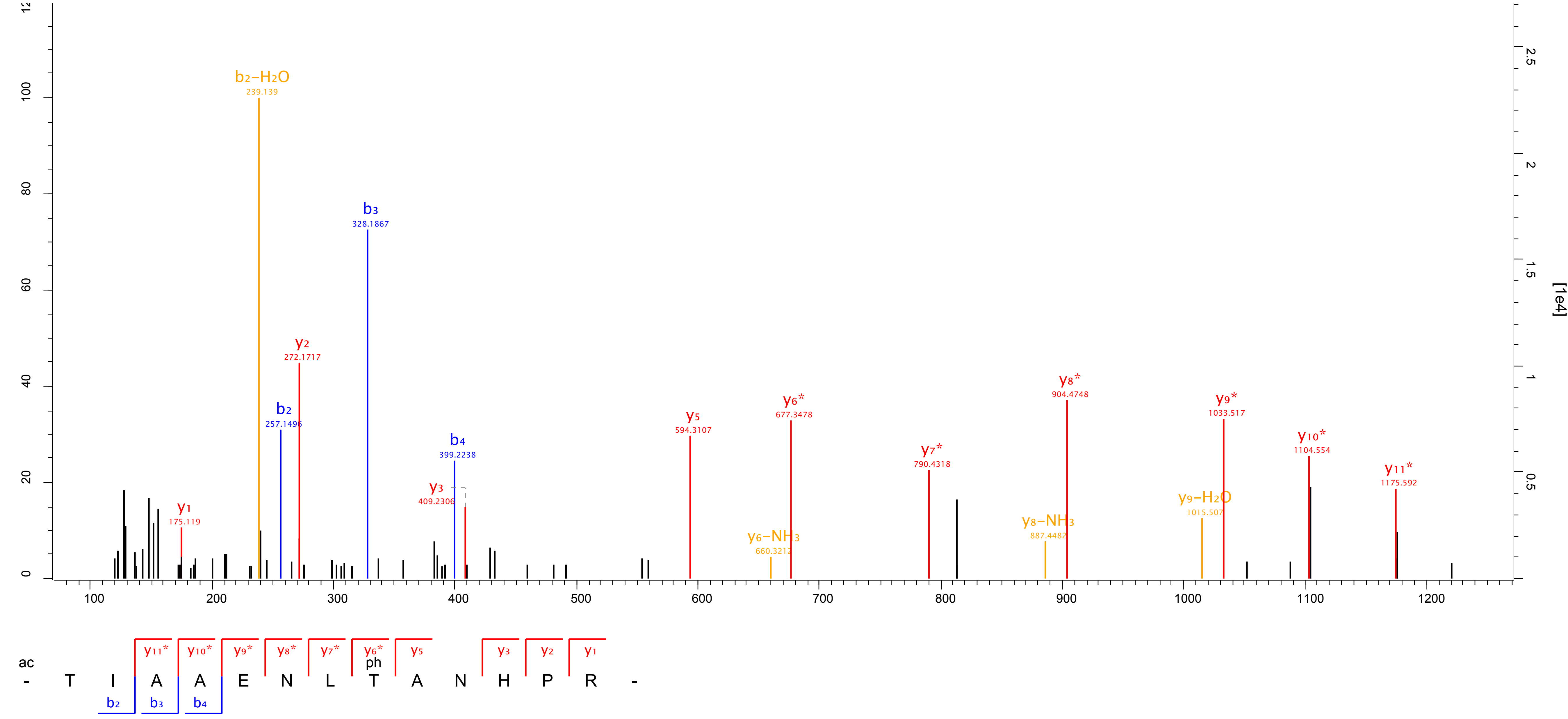

Raw file	Scan	Method	Score	m/z
20150122_PS_Syn_DimethylMix_Rep1_Phospho01	8671	FTMS; HCD	78.67	933.41

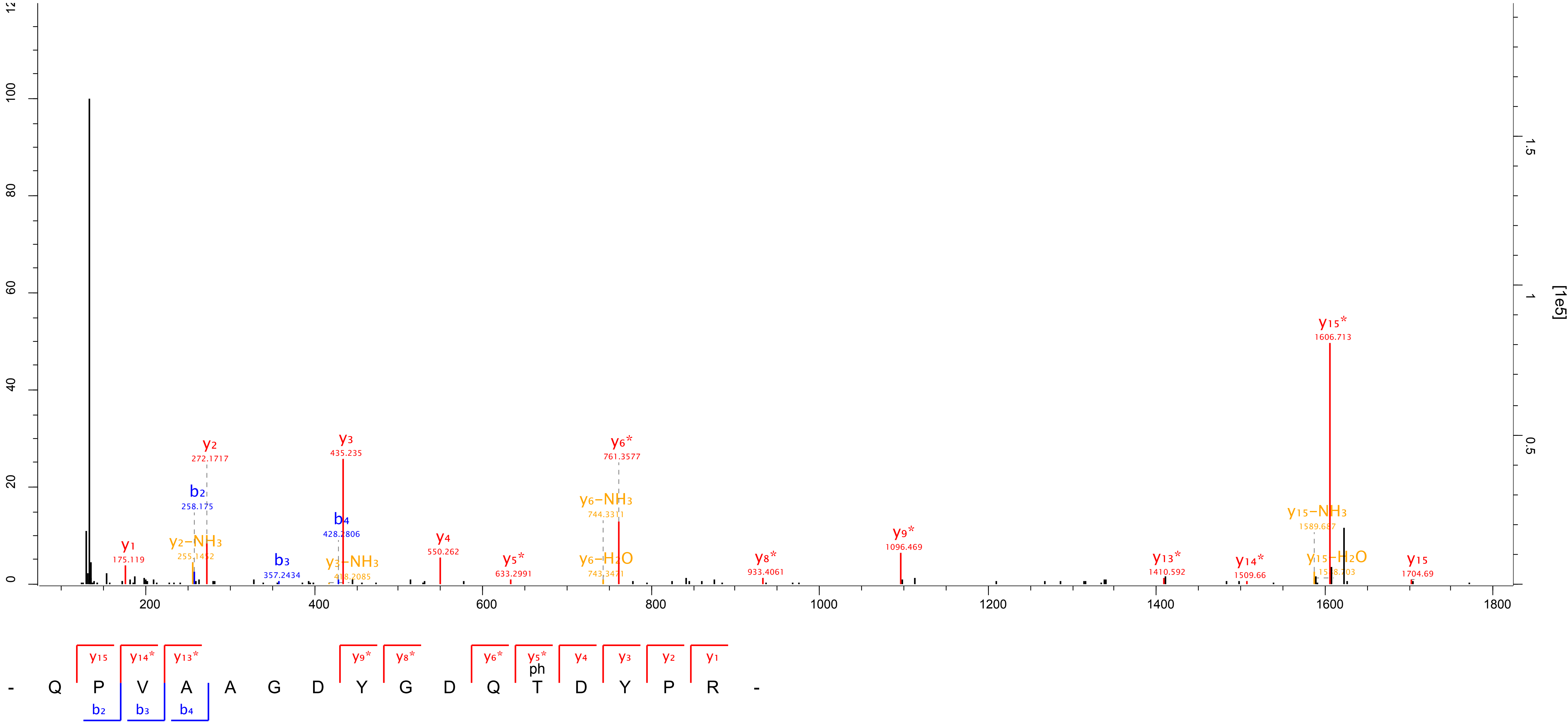

Raw file	Scan	Method	Score	m/z
20141031_PS_Syn6803wt_dimethyl_Mix_Phospho5	8773	FTMS; HCD	102.52	483.26

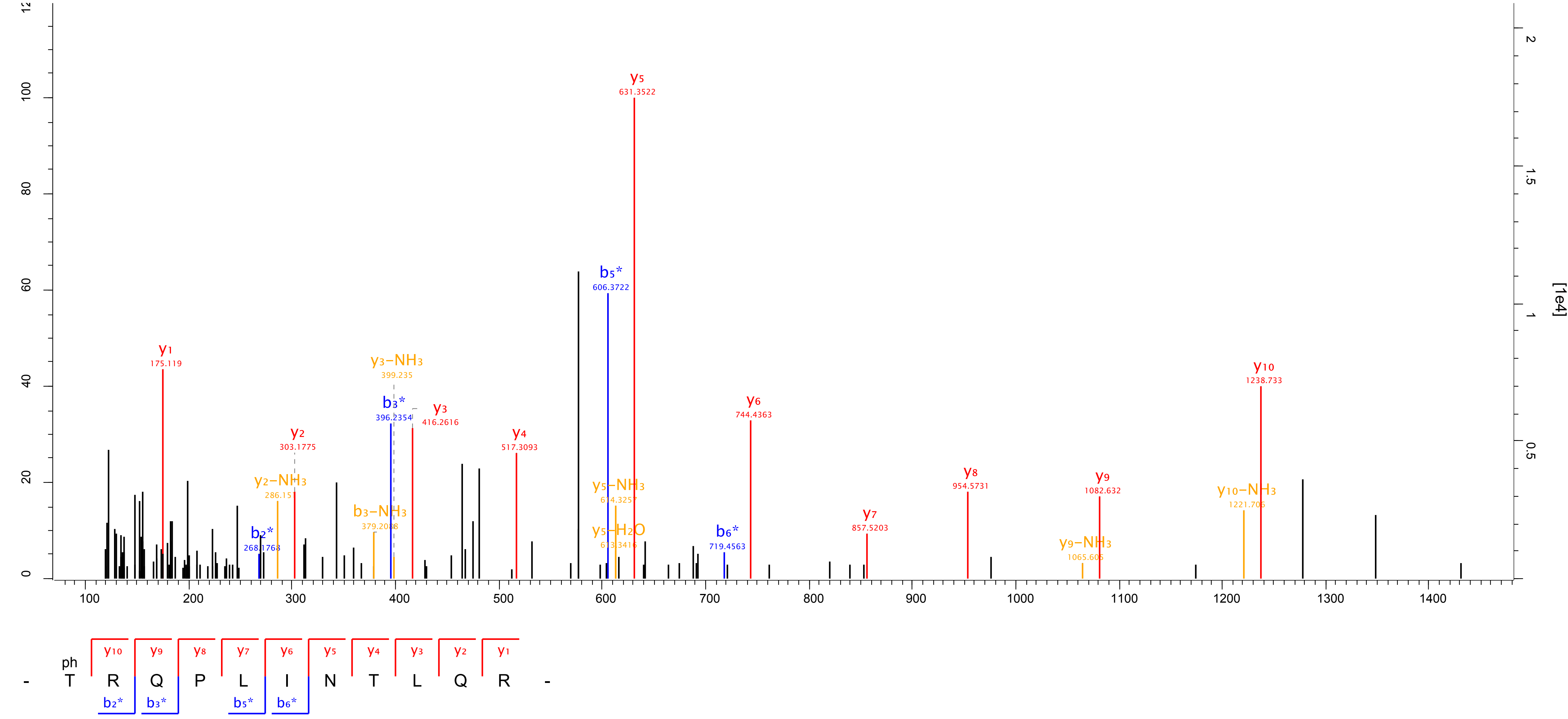

Raw file	Scan	Method	Score	m/z
20141031_PS_Syn6803wt_dimethyl_Mix_Phospho2	8890	FTMS; HCD	86	547.59

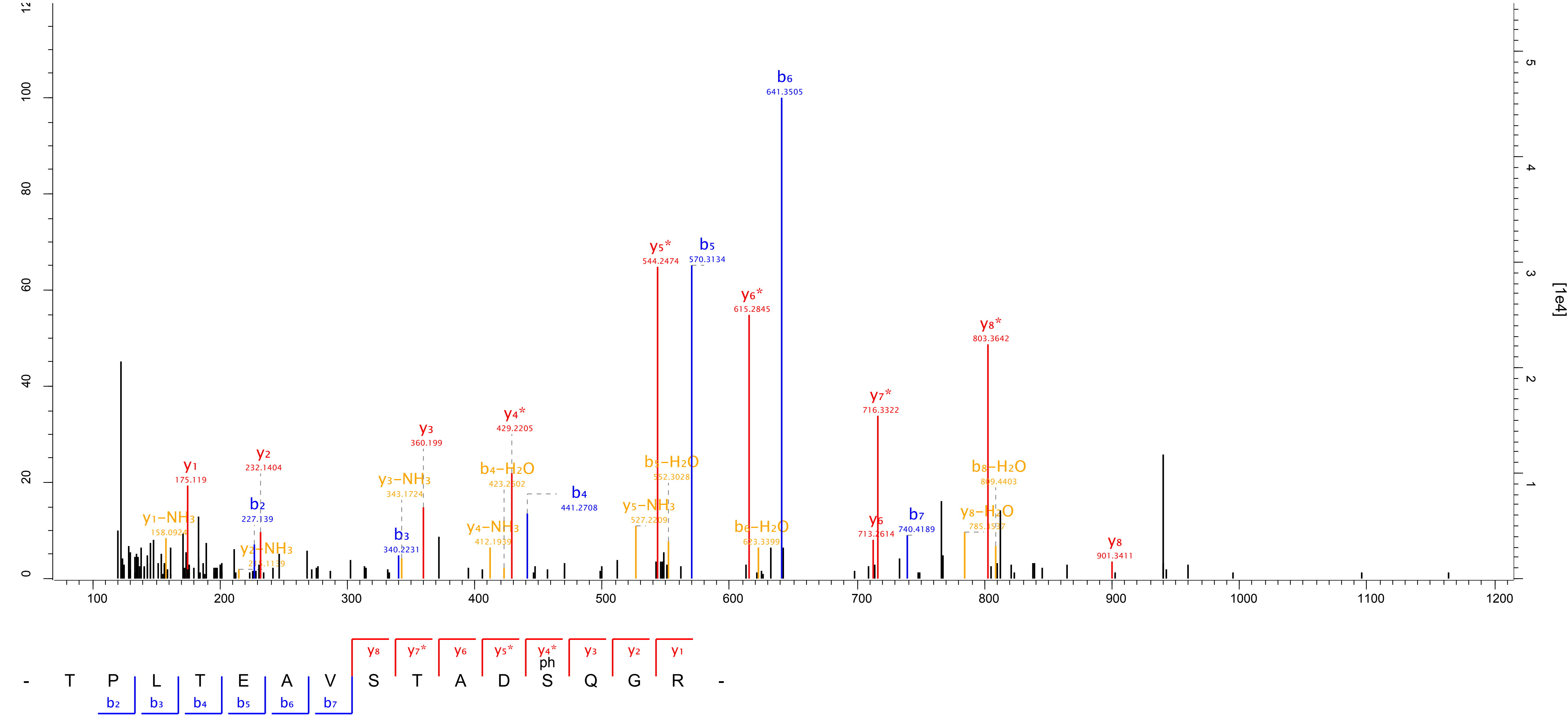

Raw file	Scan	Method	Score	m/z
20141031_PS_Syn6803wt_dimethyl_Mix_Phospho1	8928	FTMS; HCD	87.88	702.99

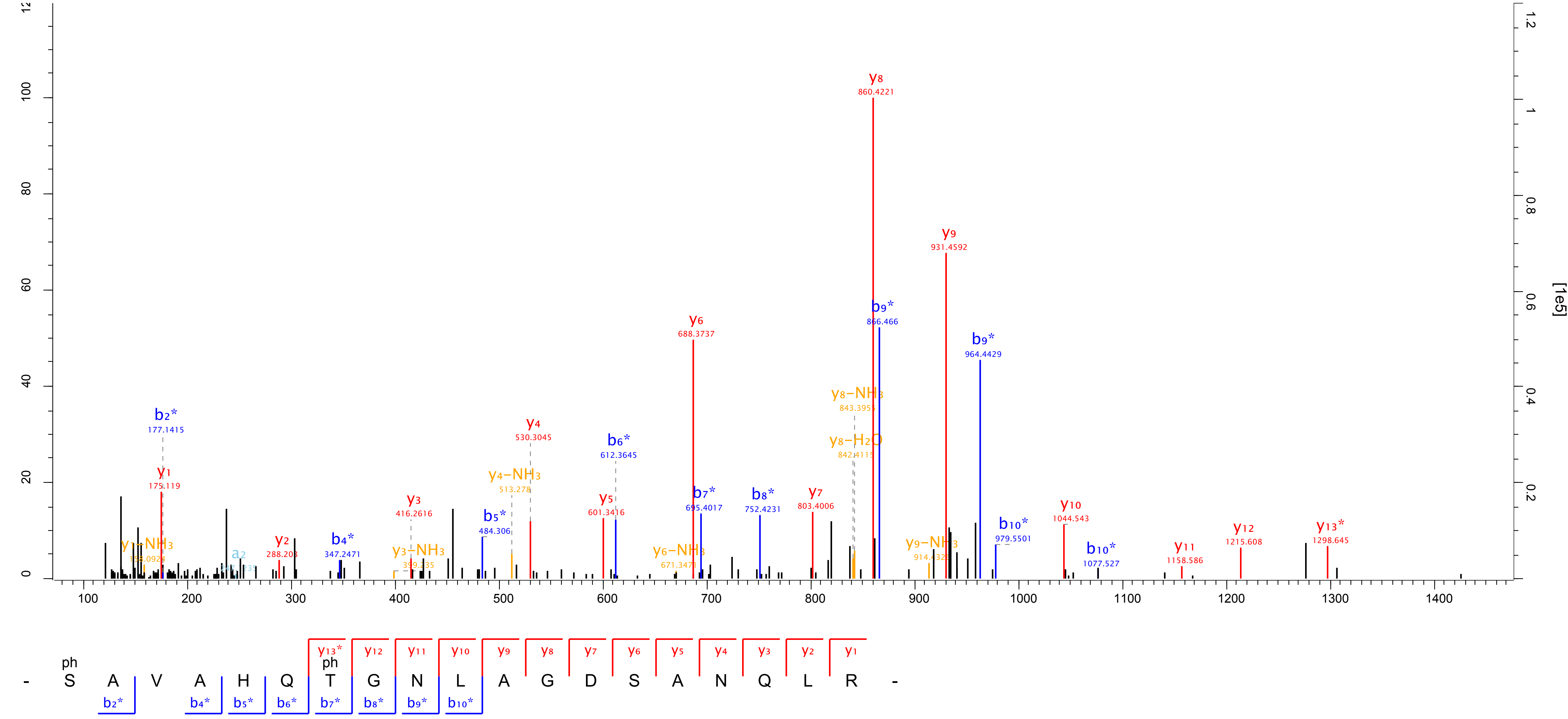

Raw file	Scan	Method	Score	m/z
20141031_PS_Syn6803wt_dimethyl_Mix_Phospho4	9043	FTMS; HCD	78.17	547.59

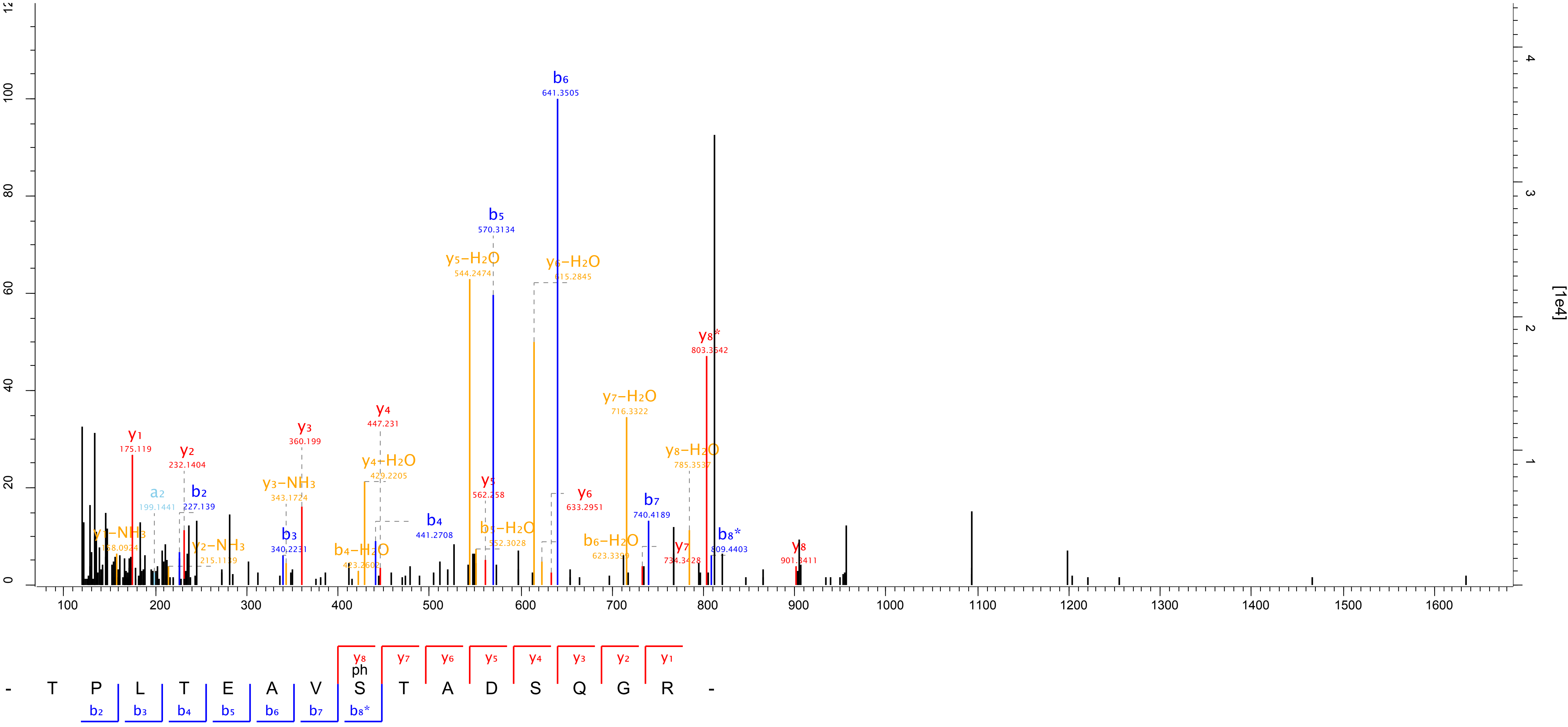

Raw file	Scan	Method	Score	m/z
20141031_PS_Syn6803wt_dimethyl_Mix_OG5	9065	FTMS; HCD	63.09	724.91

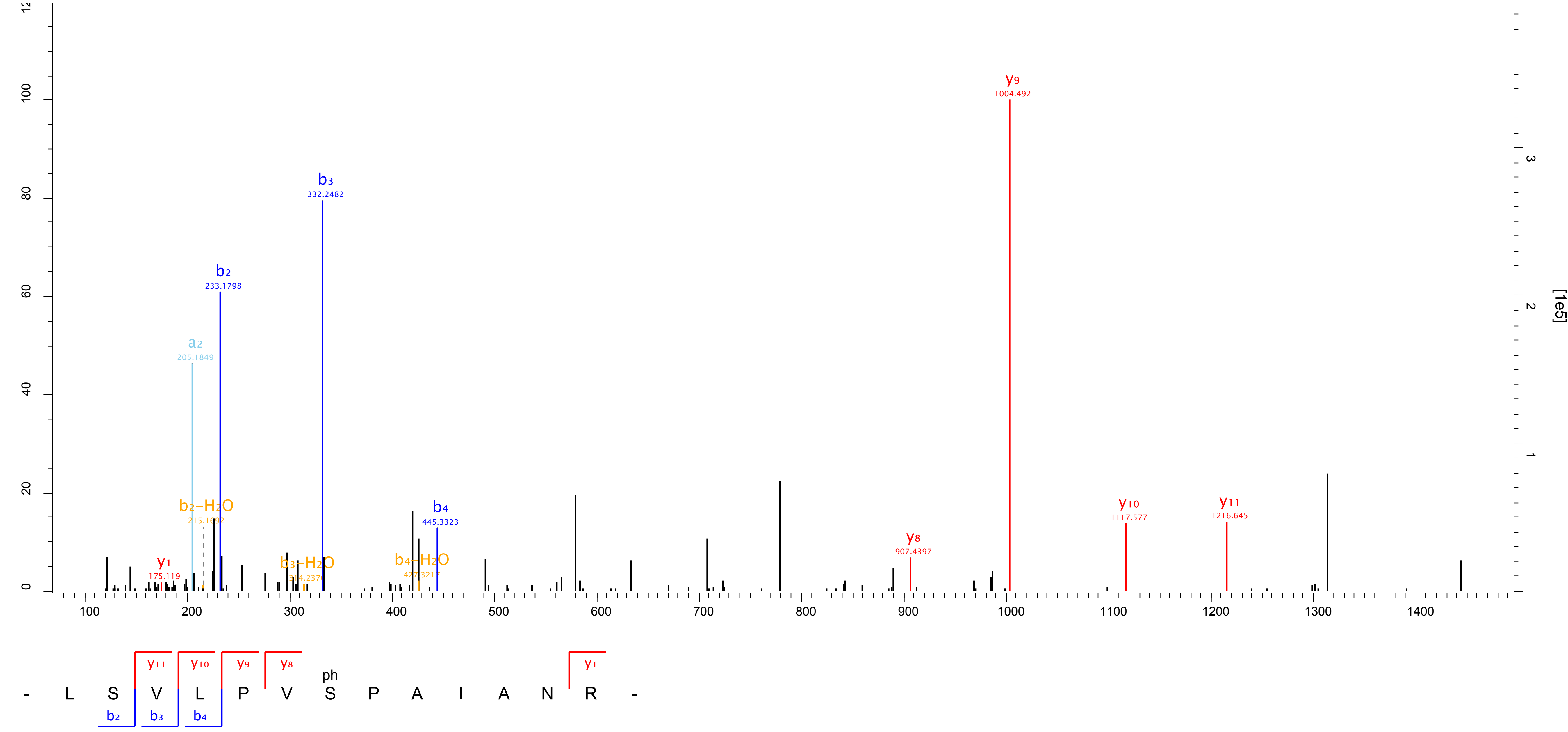

Raw file	Scan	Method	Score	m/z
20141031_PS_Syn6803wt_dimethyl_Mix_Phospho4	9229	FTMS; HCD	128.94	571.63

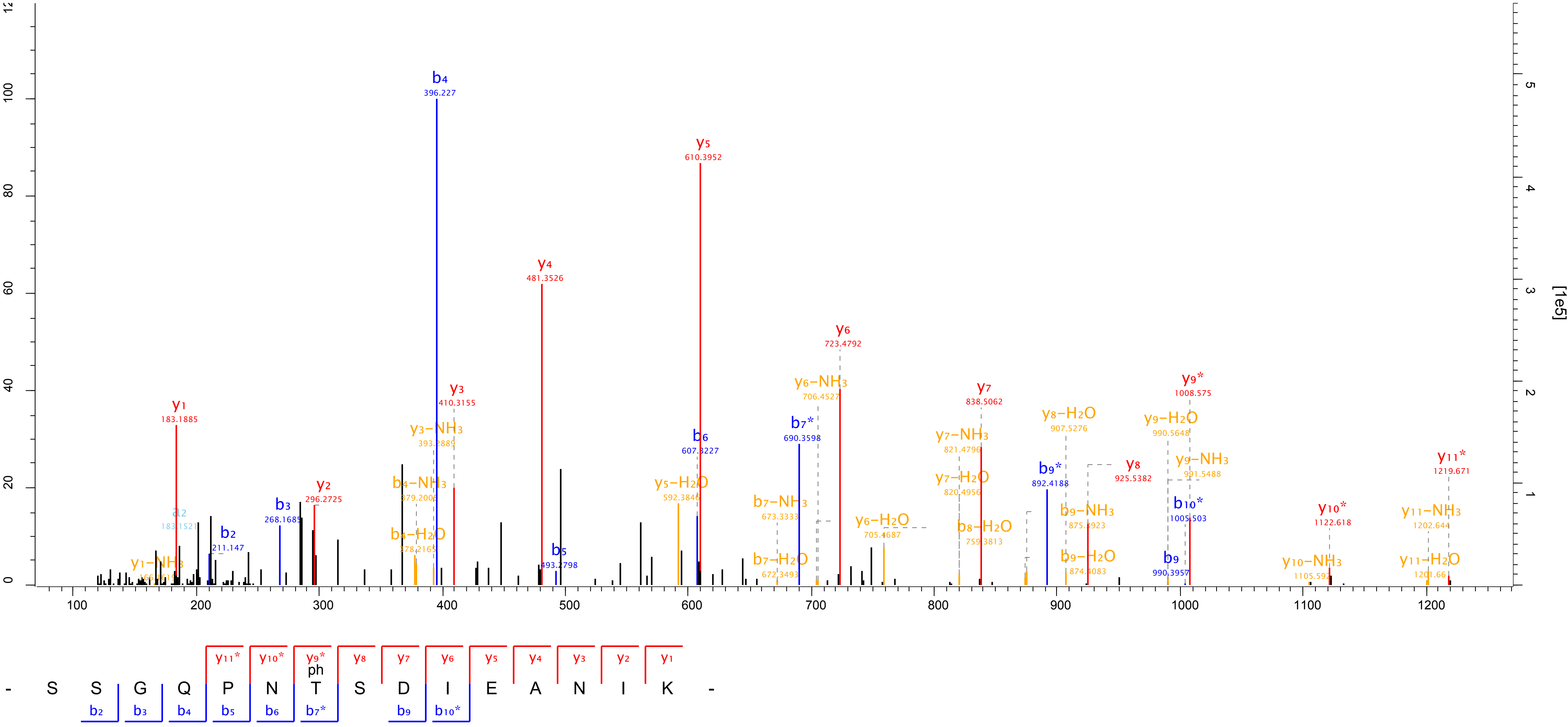

Raw file	Scan	Method	Score	m/z
20141031_PS_Syn6803wt_dimethyl_Mix_Phospho1	9403	FTMS; HCD	124.38	623.83

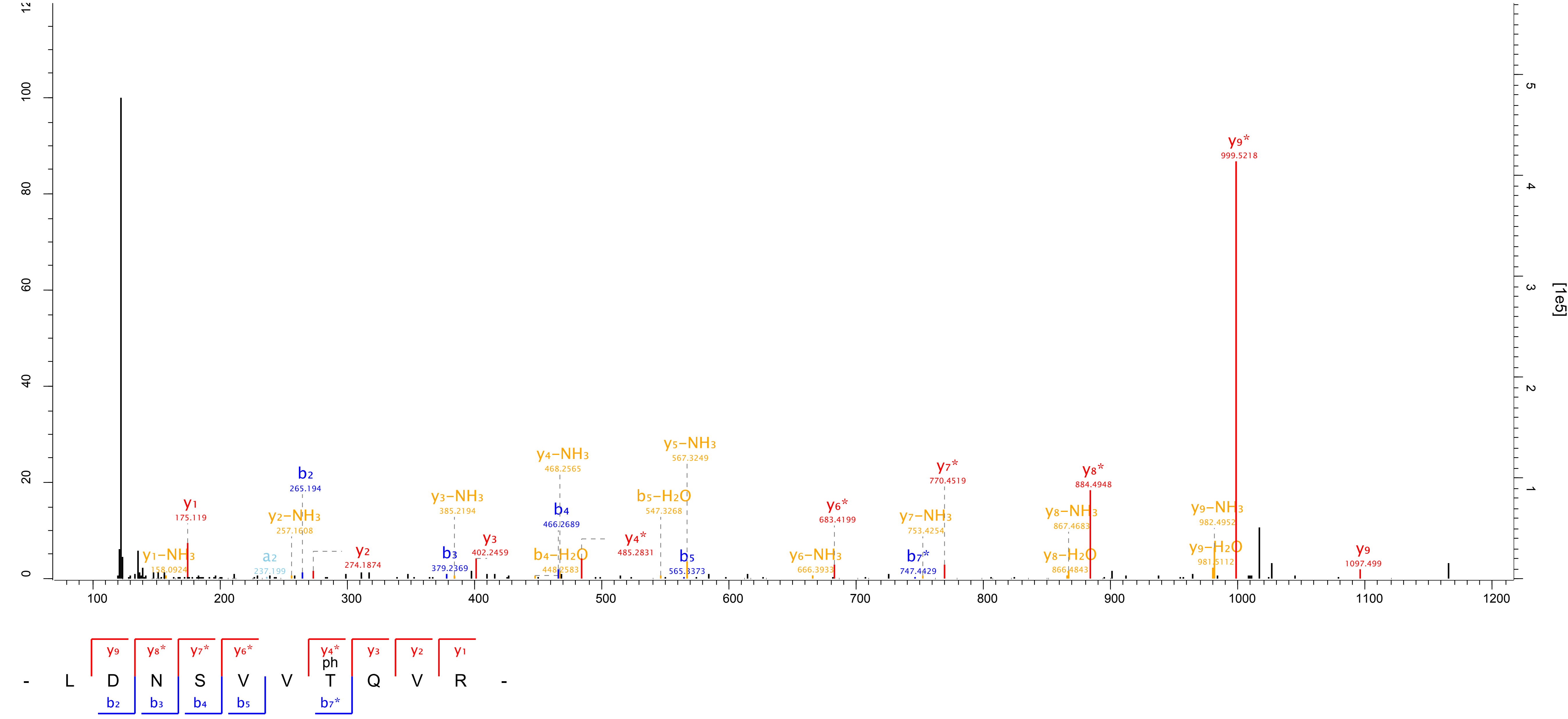

Raw file	Scan	Method	Score	m/z
20141031_PS_Syn6803wt_dimethyl_Mix_Phospho2	9431	FTMS; HCD	93.39	728.88

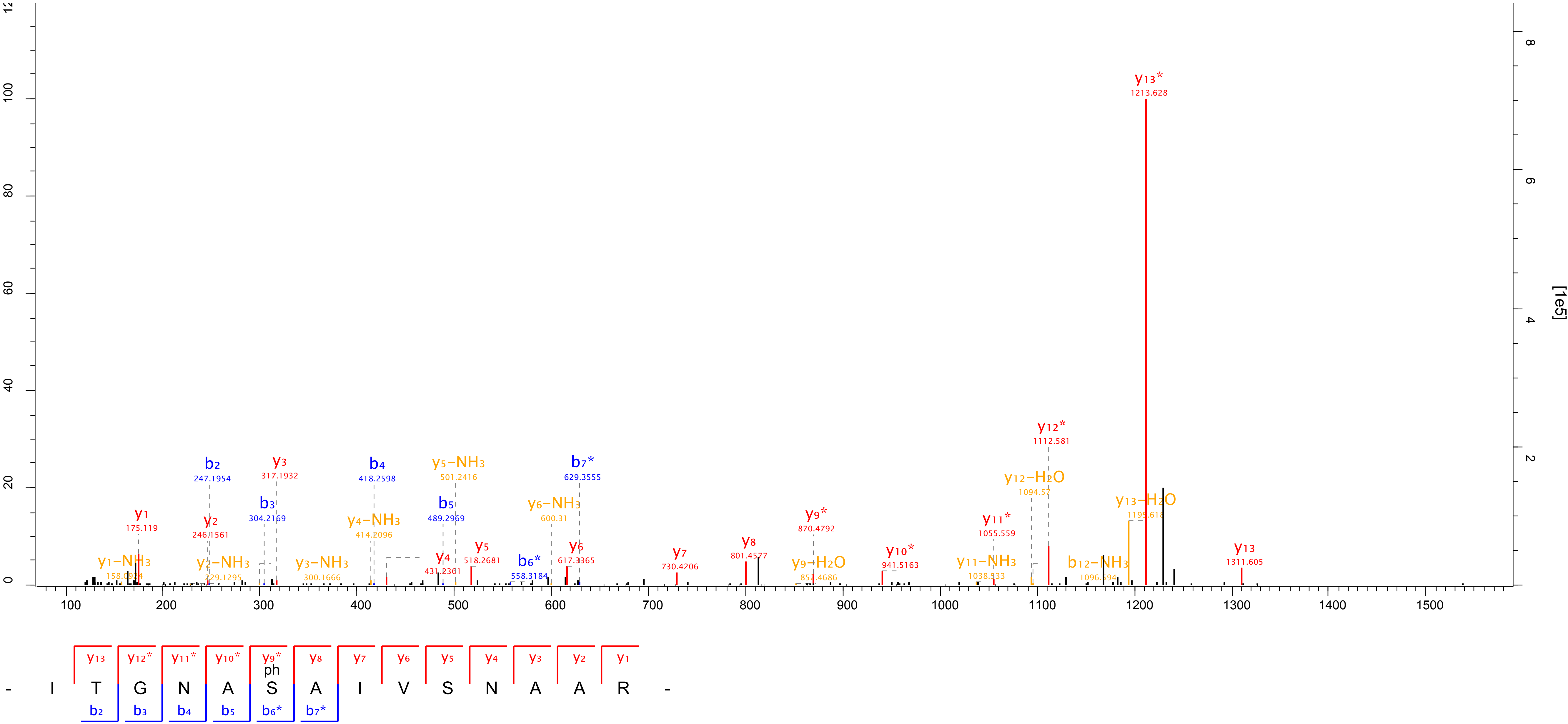

Raw file	Scan	Method	Score	m/z
20141031_PS_Syn6803wt_dimethyl_Mix_Phospho1	9539	FTMS; HCD	128.72	608.95

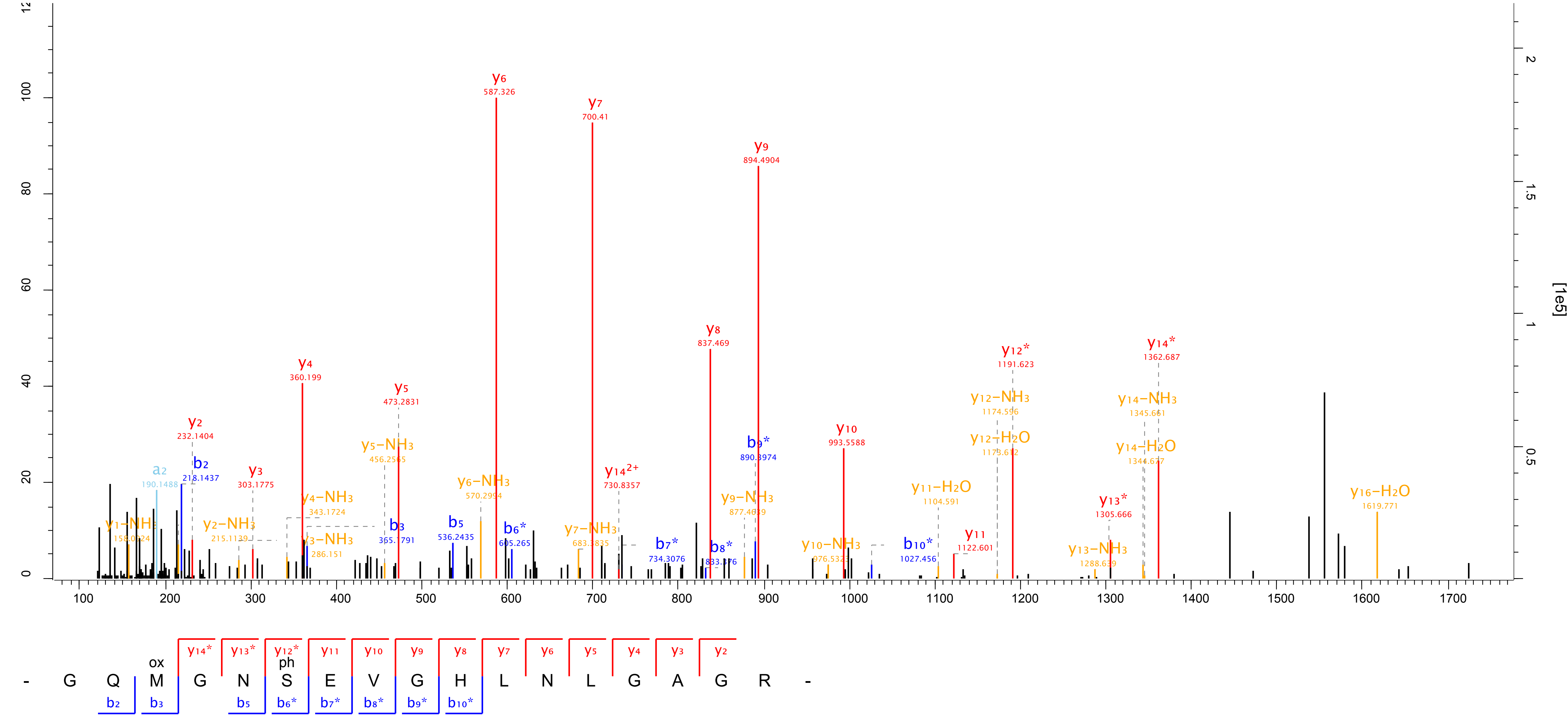

Raw file	Scan	Method	Score	m/z
20141031_PS_Syn6803wt_dimethyl_Mix_Phospho3	9690	FTMS; HCD	137.12	763.7

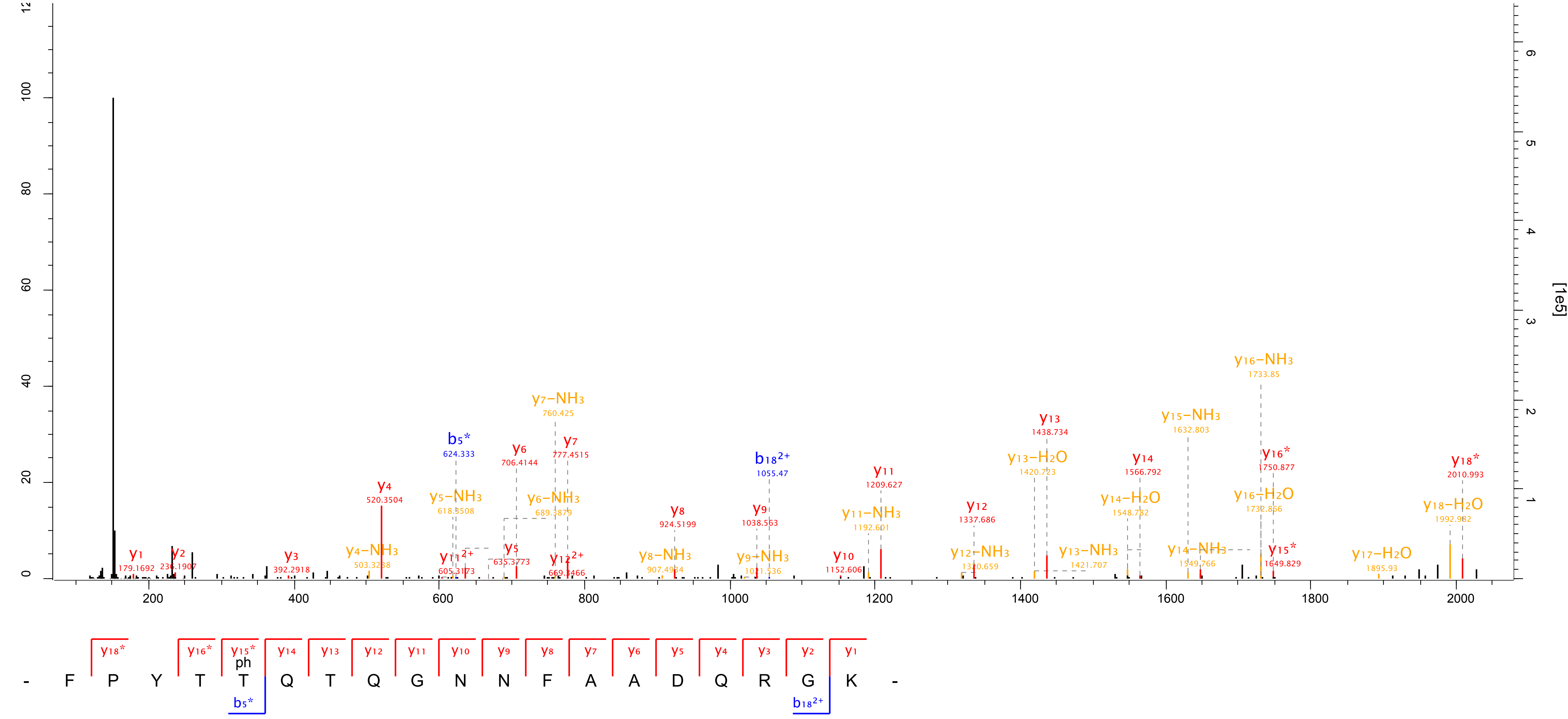

Quantitative Dataset

Phosphopeptide MS/MS Spectra (HCD)

Unlocalized phosphorylation events

Raw file	Scan	Method	Score	m/z
20141031_PS_Syn6803wt_dimethyl_Mix_Phospho1	10249	FTMS; HCD	62.03	742.36

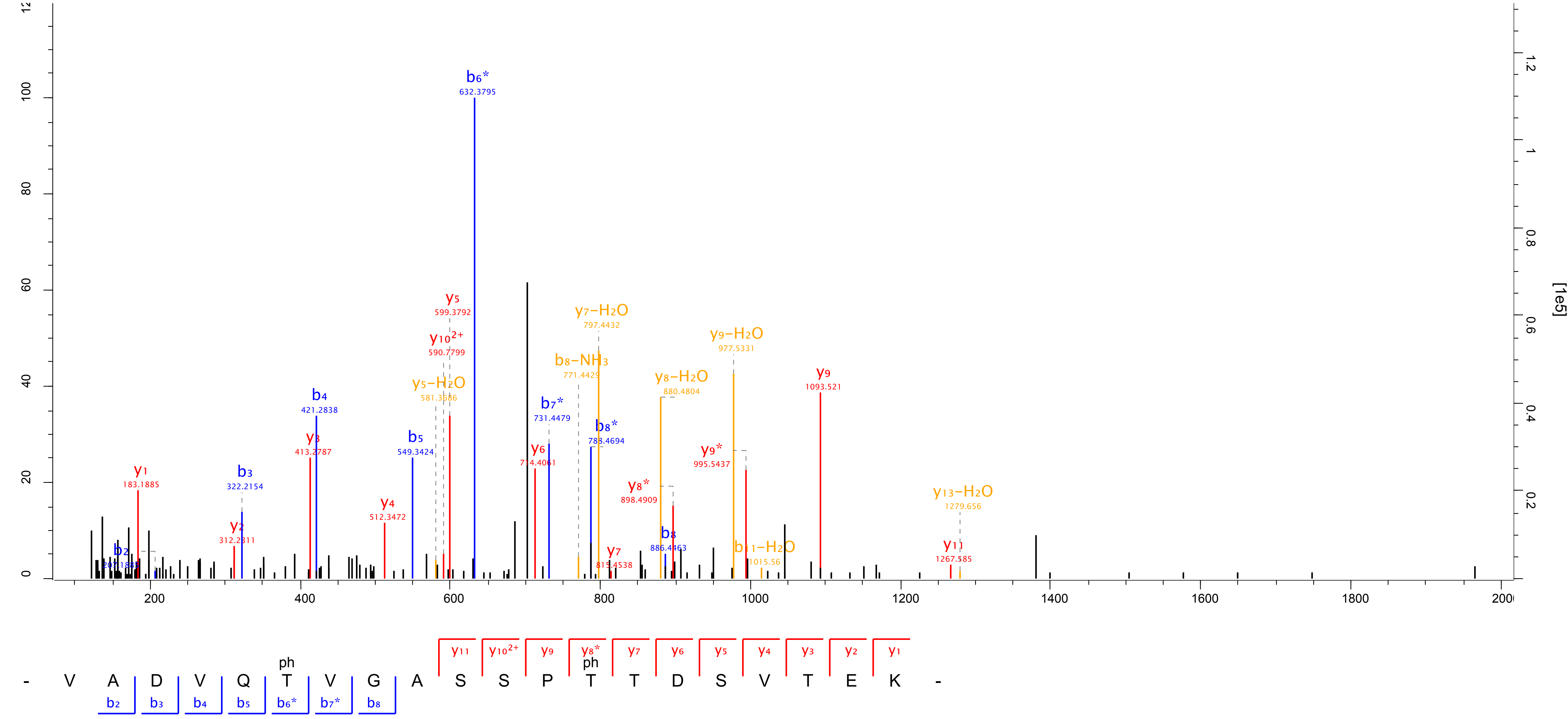

Raw file	Scan	Method	Score	m/z
201417_PS_Syn6803wt_dimethylMix_TiO2_4	10669	FTMS; HCD	48.35	506.61

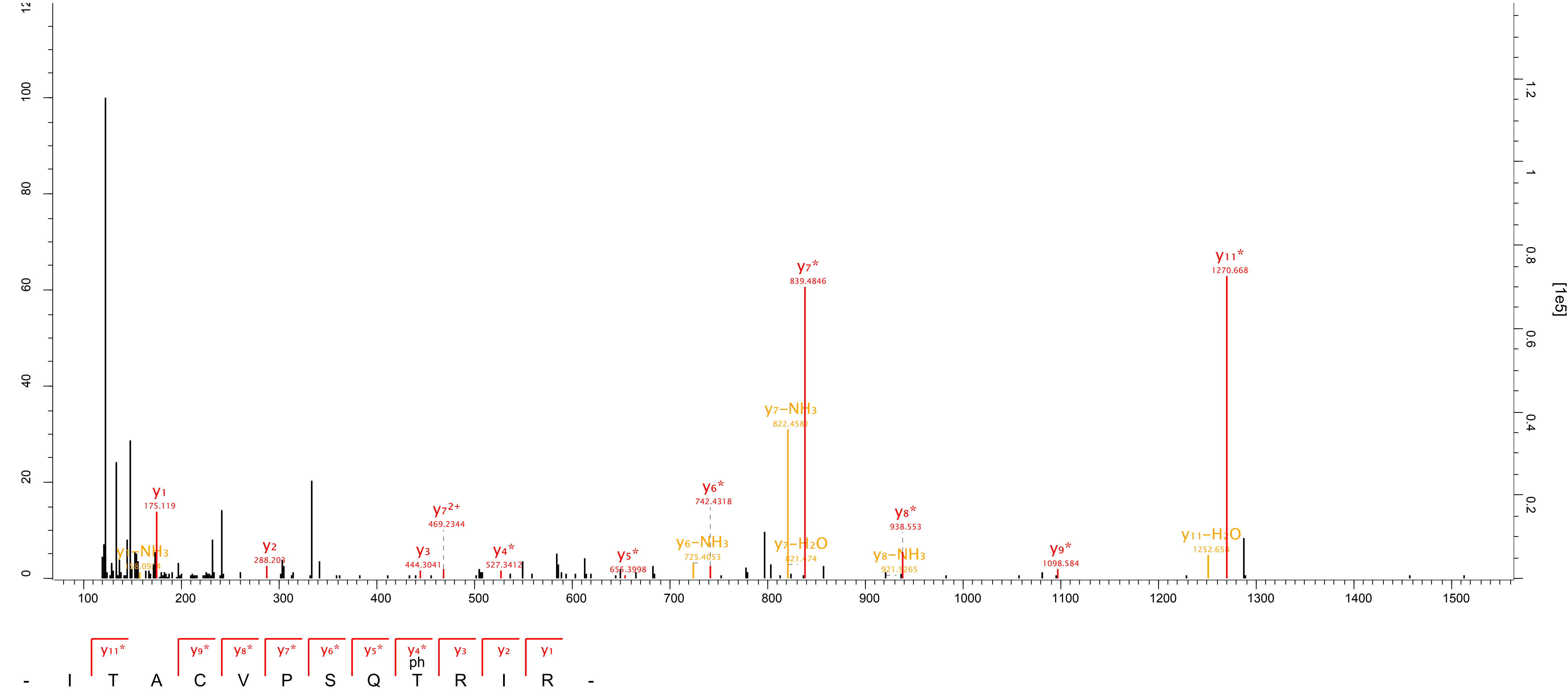

Raw file	Scan	Method	Score	m/z
201417_PS_Syn6803wt_dimethylMix_TiO2_1	10754	FTMS; HCD	51.07	736.35

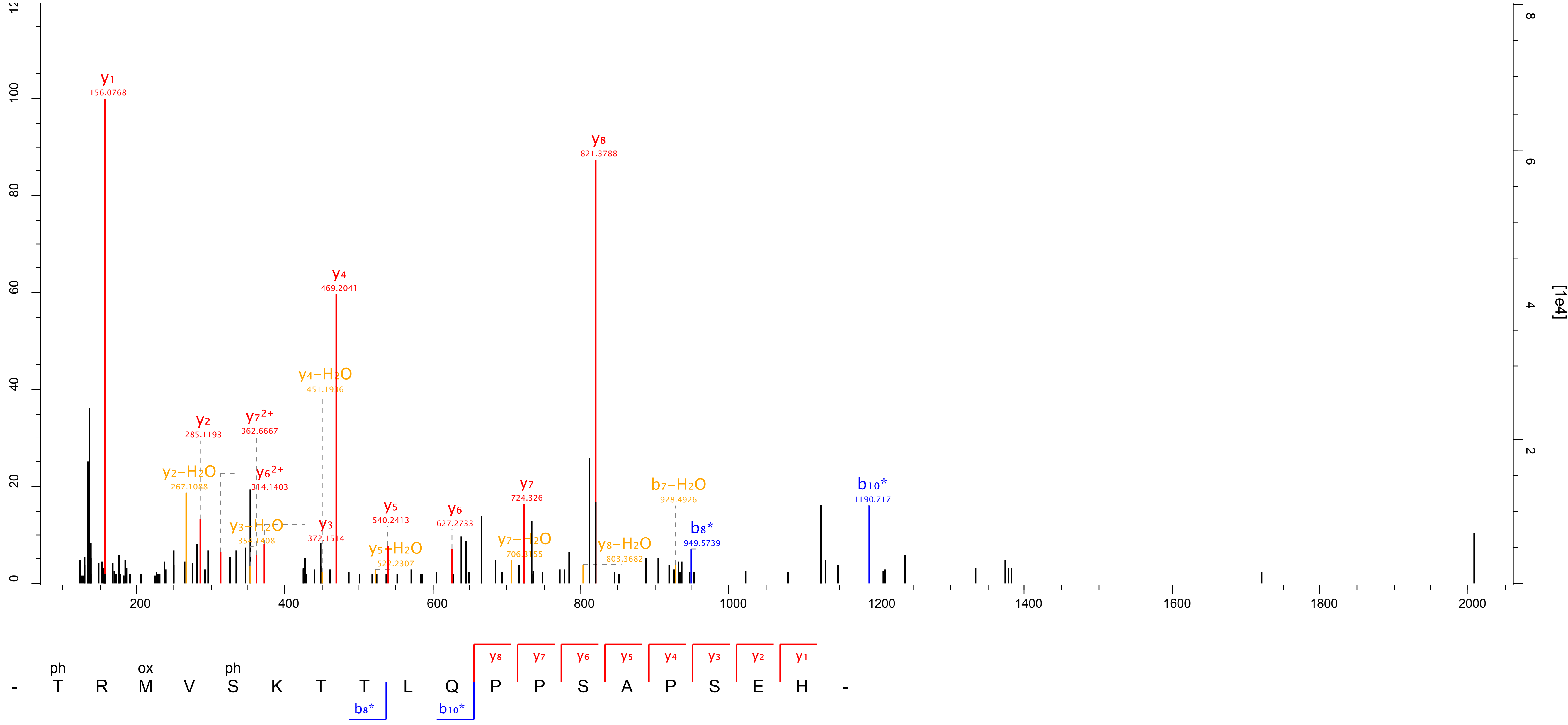

Raw file	Scan	Method	Score	m/z
20141031_PS_Syn6803wt_dimethyl_Mix_Phospho4	11139	FTMS; HCD	85.84	580.29

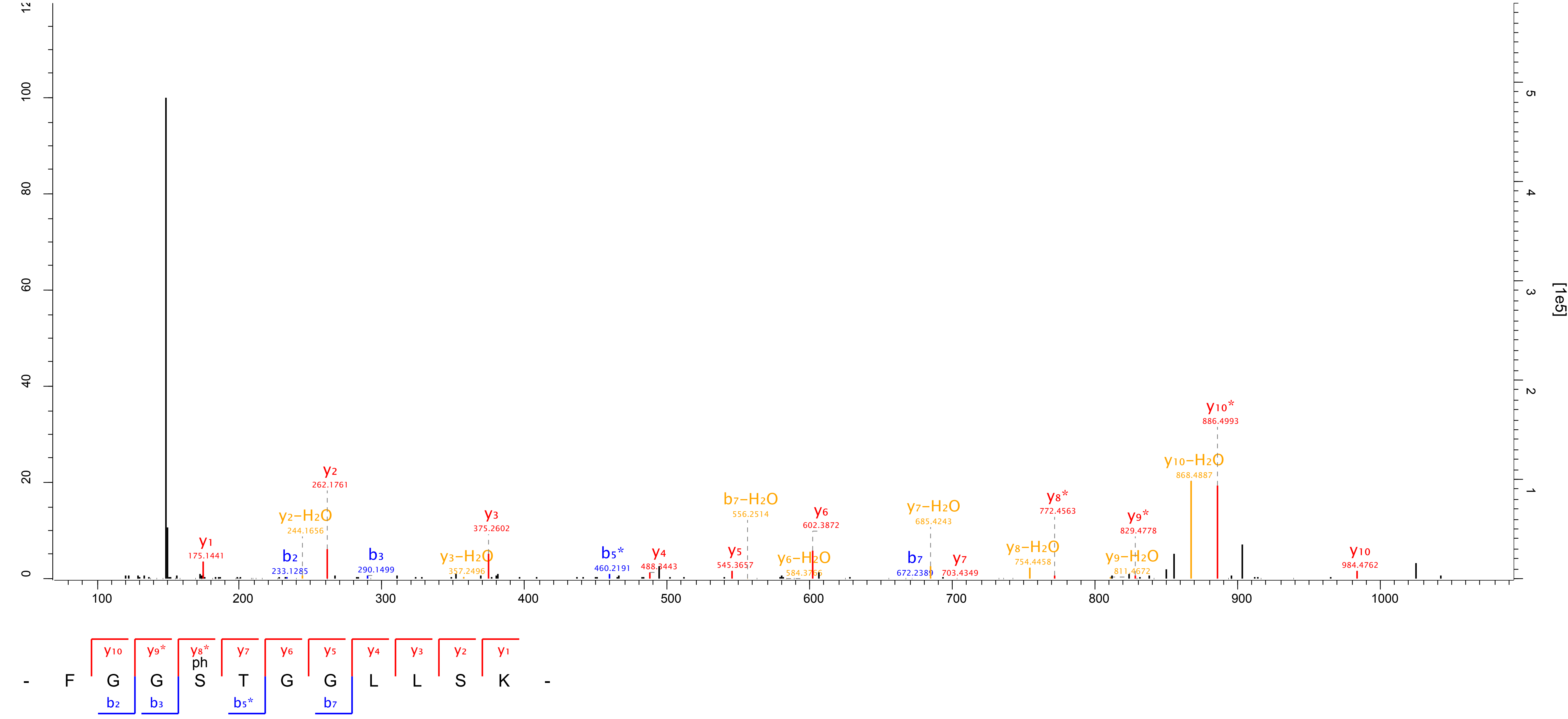

Raw file	Scan	Method	Score	m/z
20150122_PS_Syn_DimethylMix_Rep1_Phospho02	11480	FTMS; HCD	85.56	860.41

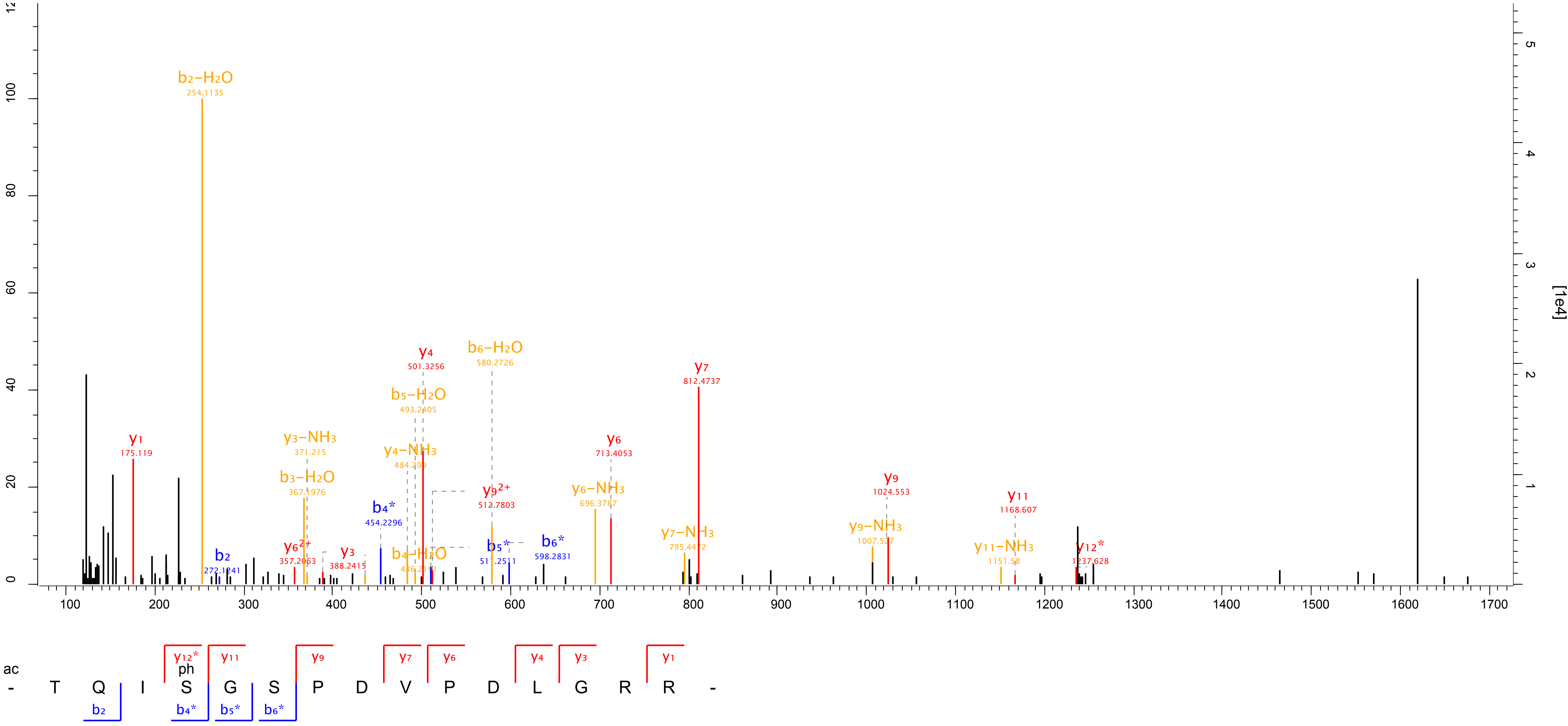

Raw file	Scan	Method	Score	m/z
20141031_PS_Syn6803wt_dimethyl_Mix_Phospho3	11646	FTMS; HCD	56.43	691.09

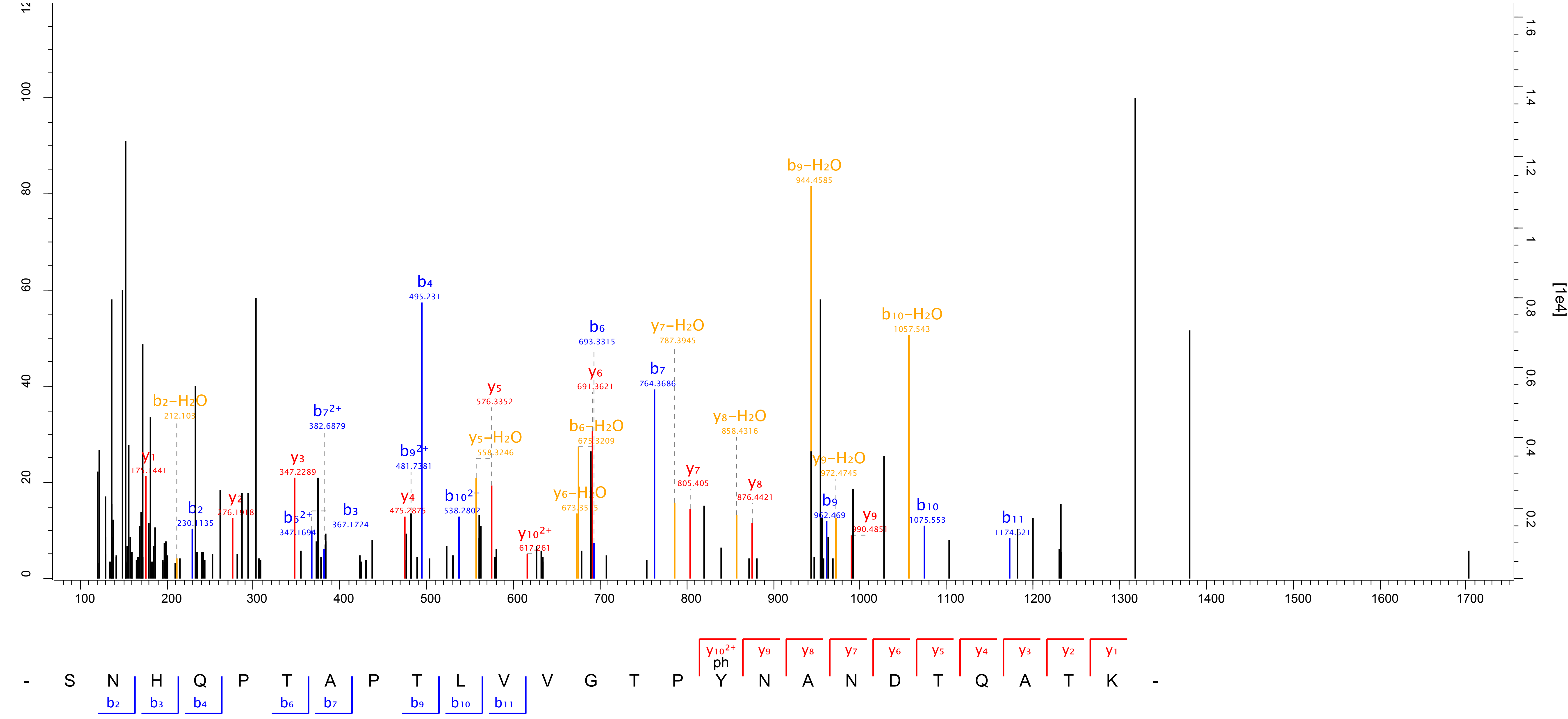

Raw file	Scan	Method	Score	m/z
20150122_PS_Syn_DimethylMix_Rep2_Phospho01	11764	FTMS; HCD	51.03	610.32

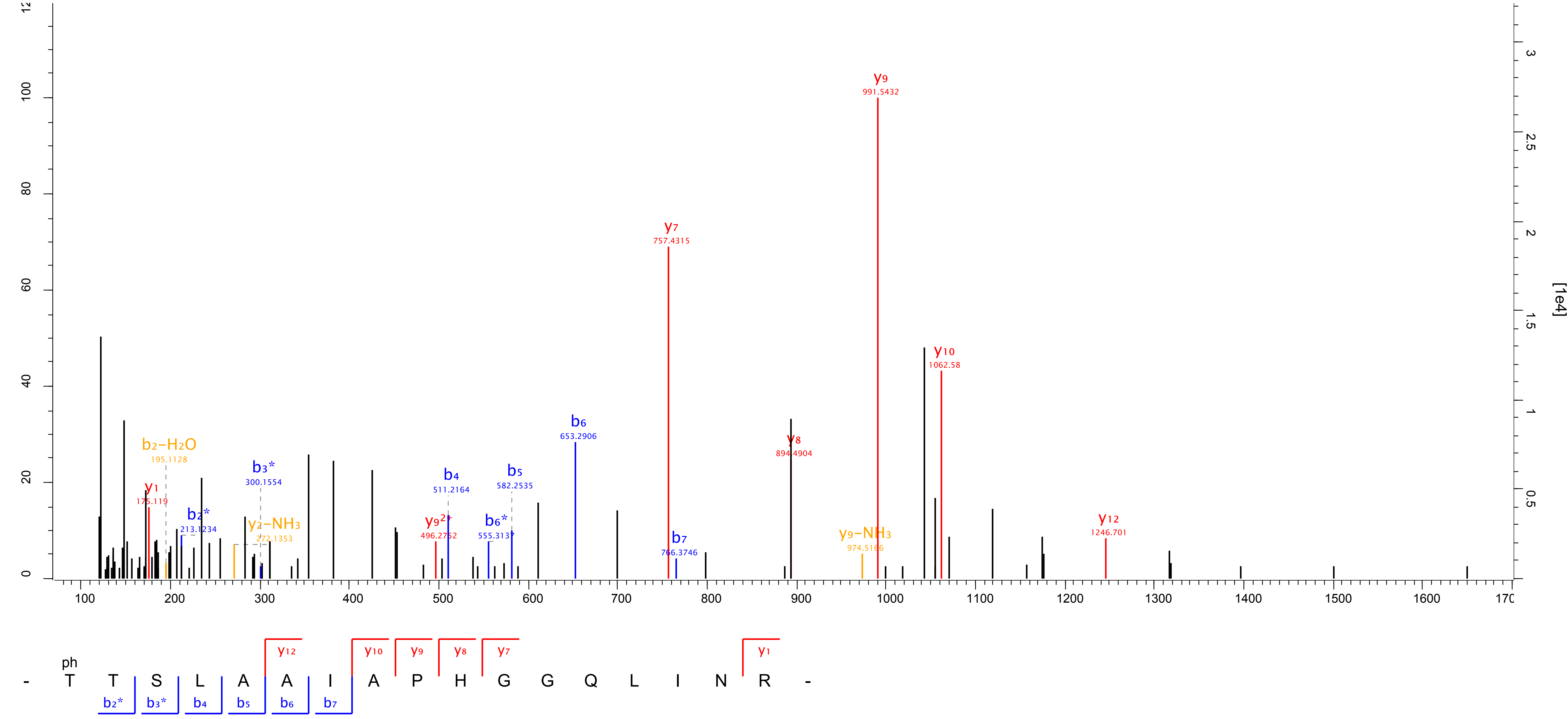

Raw file	Scan	Method	Score	m/z
20141031_PS_Syn6803wt_dimethyl_Mix_Phospho8	11904	FTMS; HCD	46.56	720.38

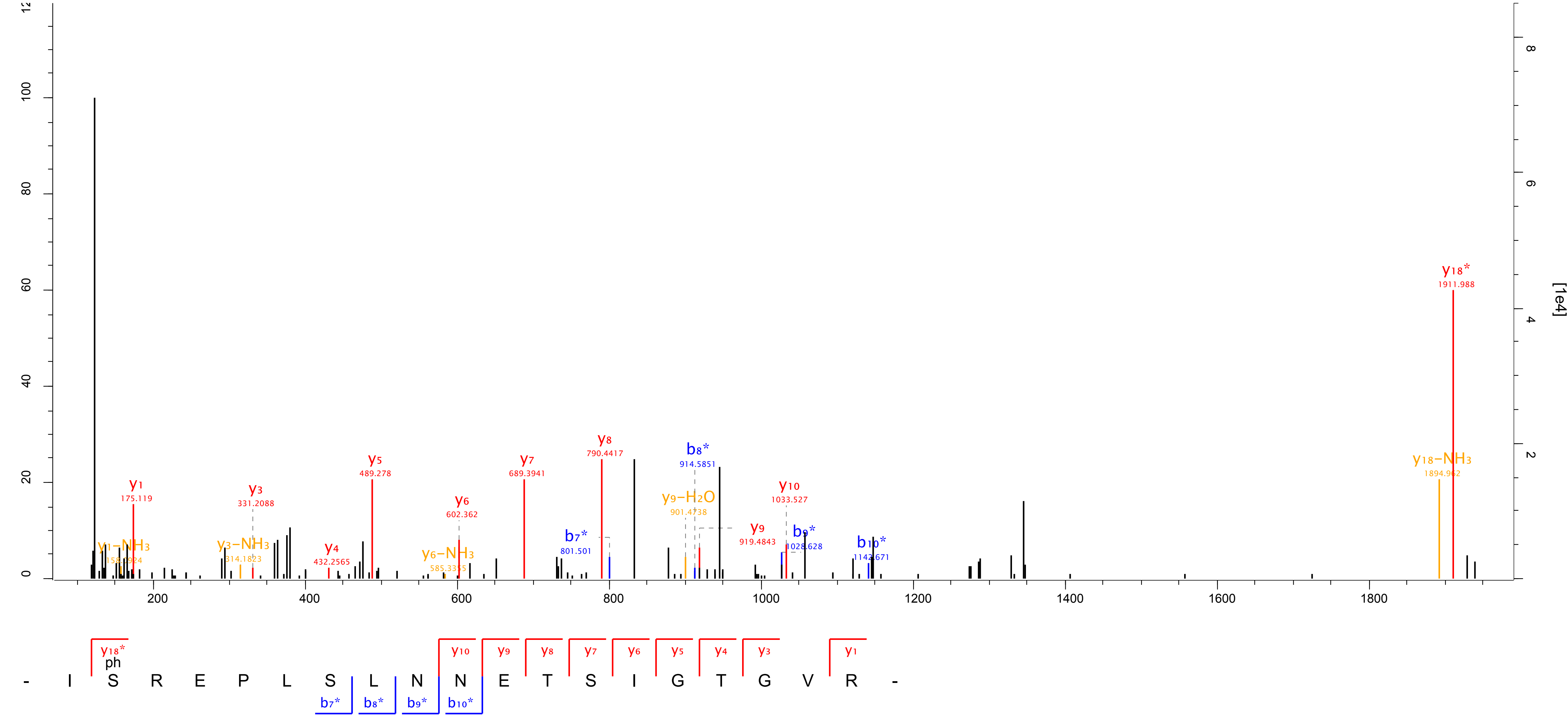

Raw file	Scan	Method	Score	m/z
20141031_PS_Syn6803wt_dimethyl_Mix_Phospho1	12299	FTMS; HCD	72.79	720.37

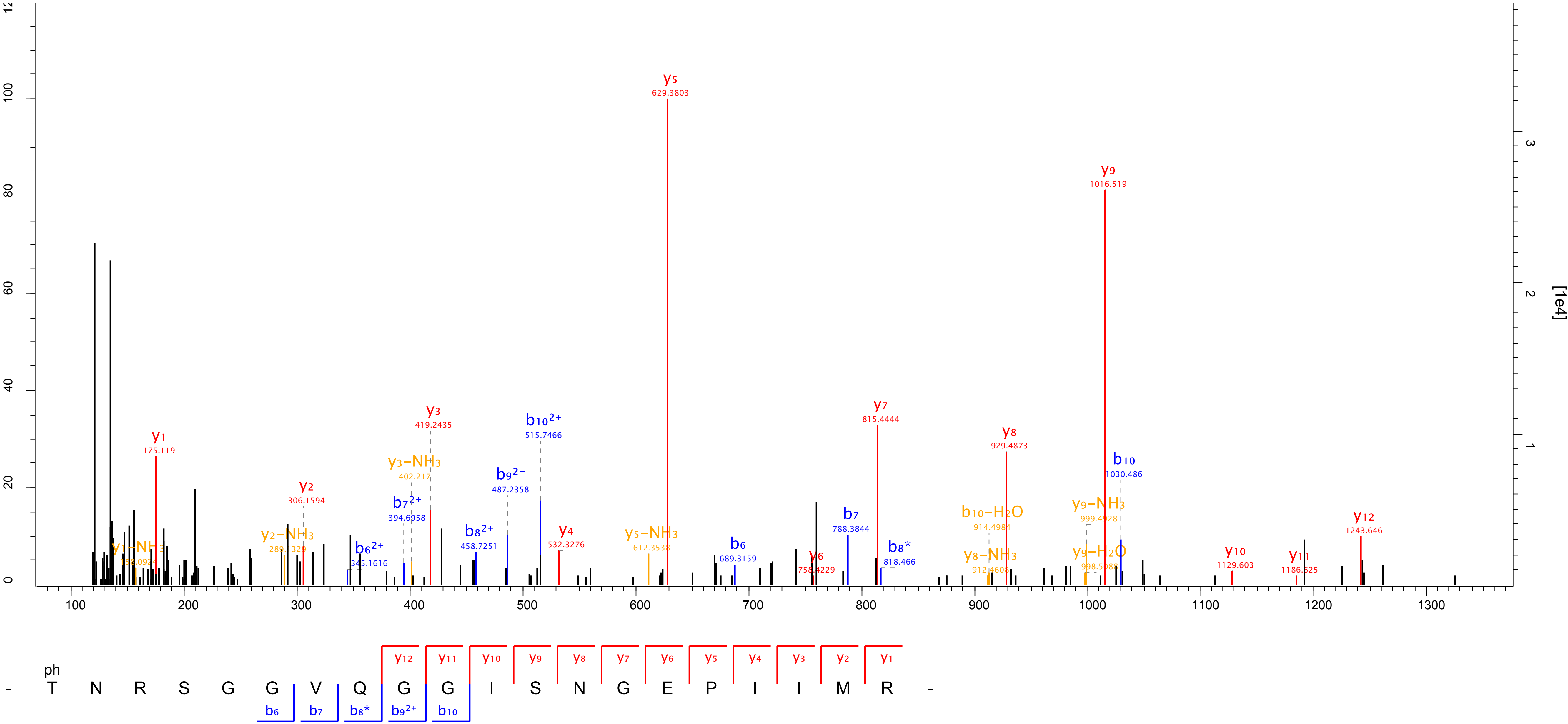

Raw file	Scan	Method	Score	m/z
20141031_PS_Syn6803wt_dimethyl_Mix_Phospho2	12418	FTMS; HCD	48.05	782.71

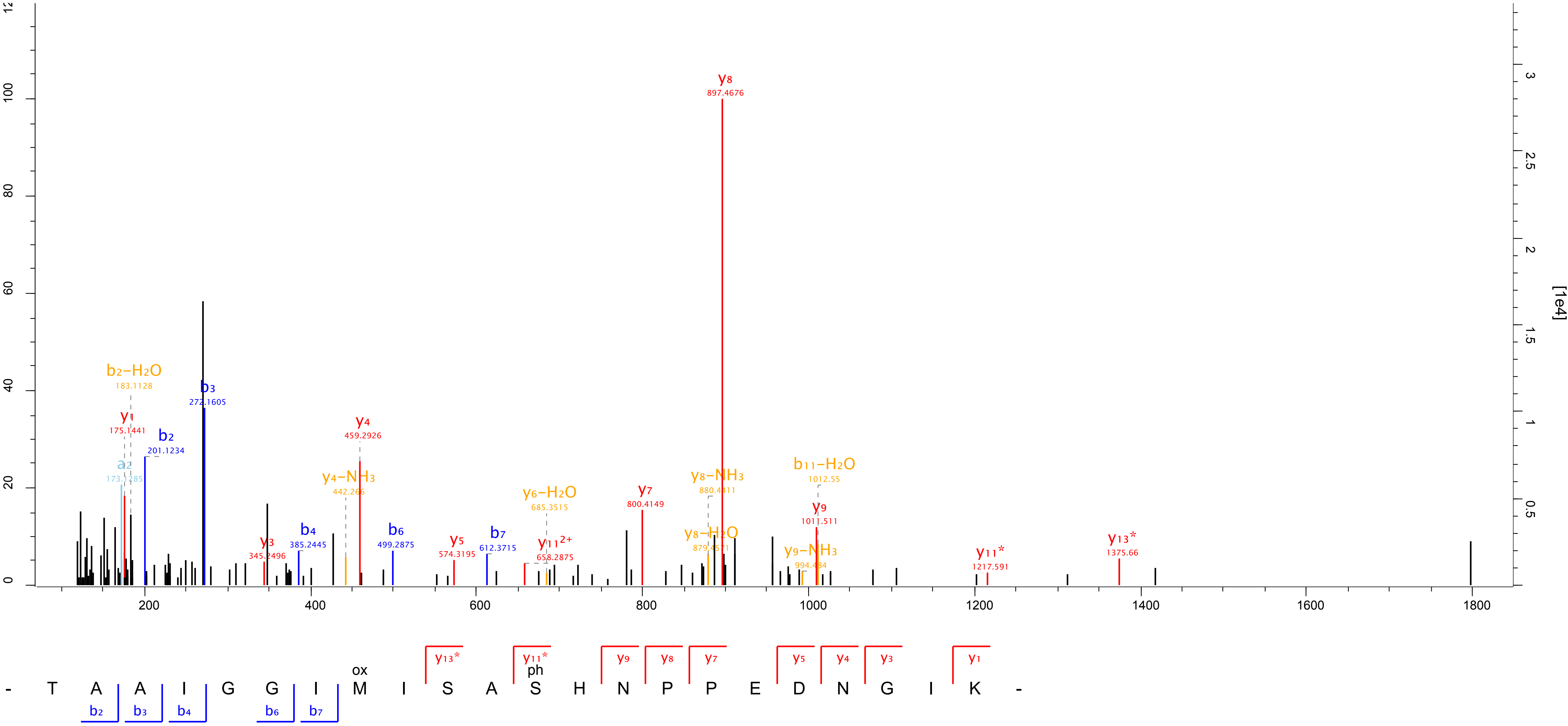

Raw file	Scan	Method	Score	m/z
20141031_PS_Syn6803wt_dimethyl_Mix_Phospho4	12434	FTMS; HCD	80.68	609.99

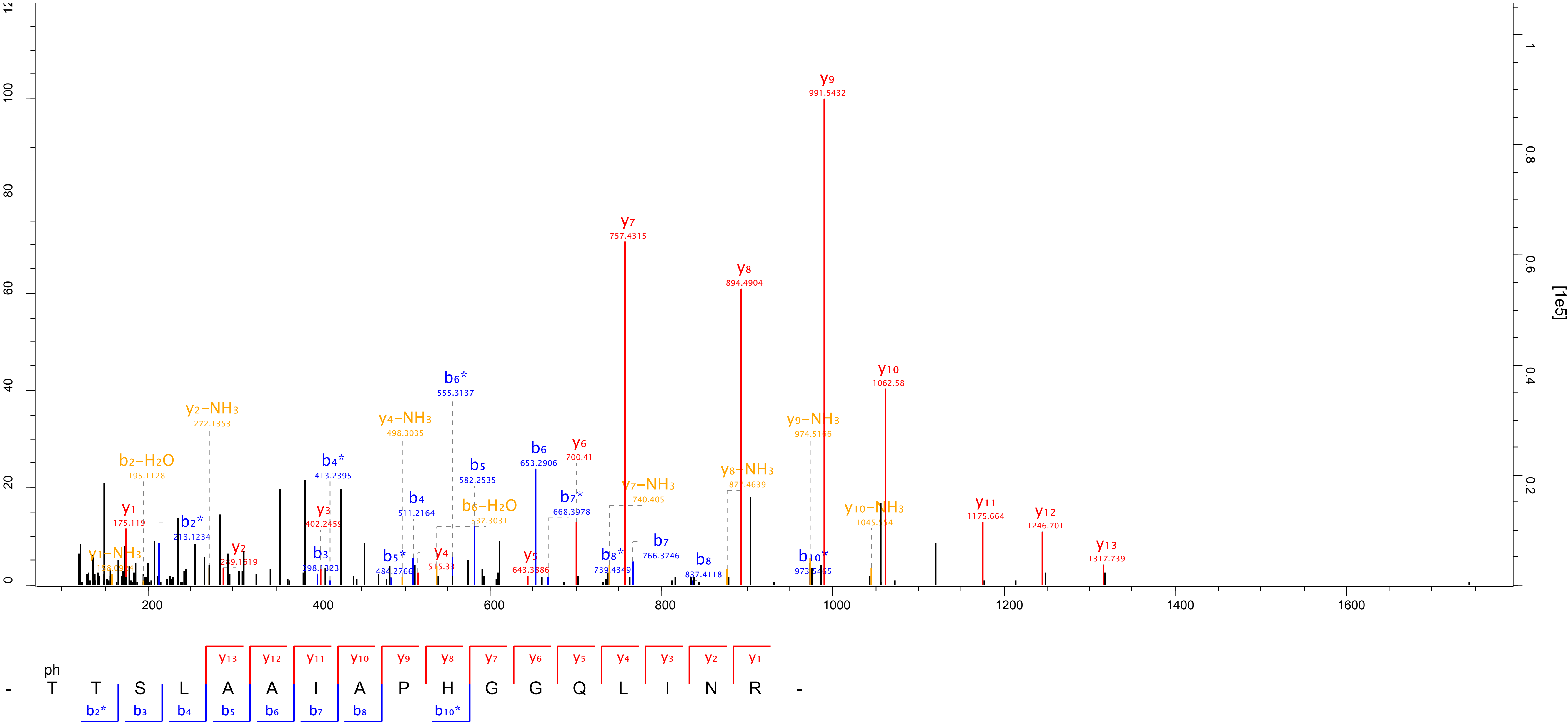

Raw file	Scan	Method	Score	m/z
20141031_PS_Syn6803wt_dimethyl_Mix_Phospho5	12788	FTMS; HCD	74.03	788.08

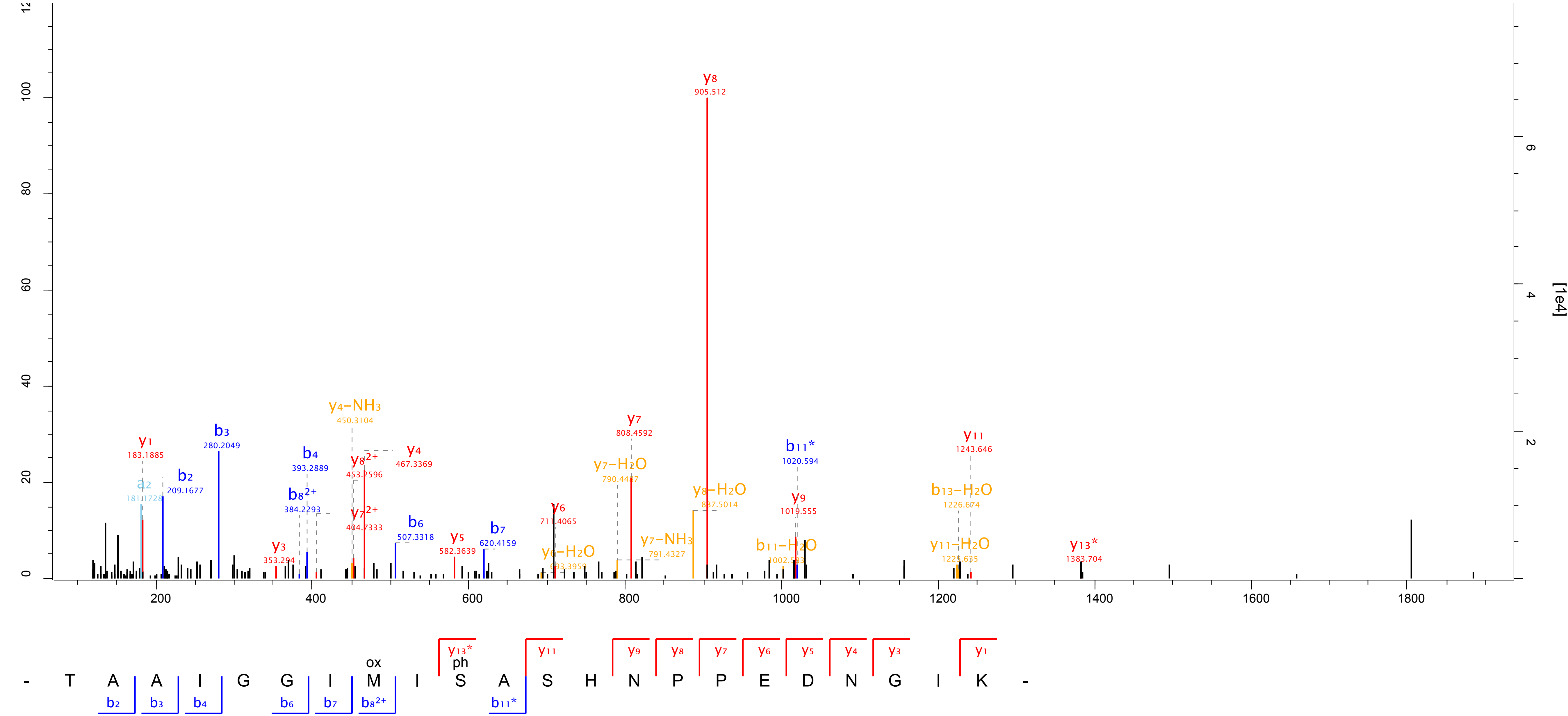

Raw file	Scan	Method	Score	m/z
20141031_PS_Syn6803wt_dimethyl_Mix_Phospho8	13015	FTMS; HCD	84.75	687.86

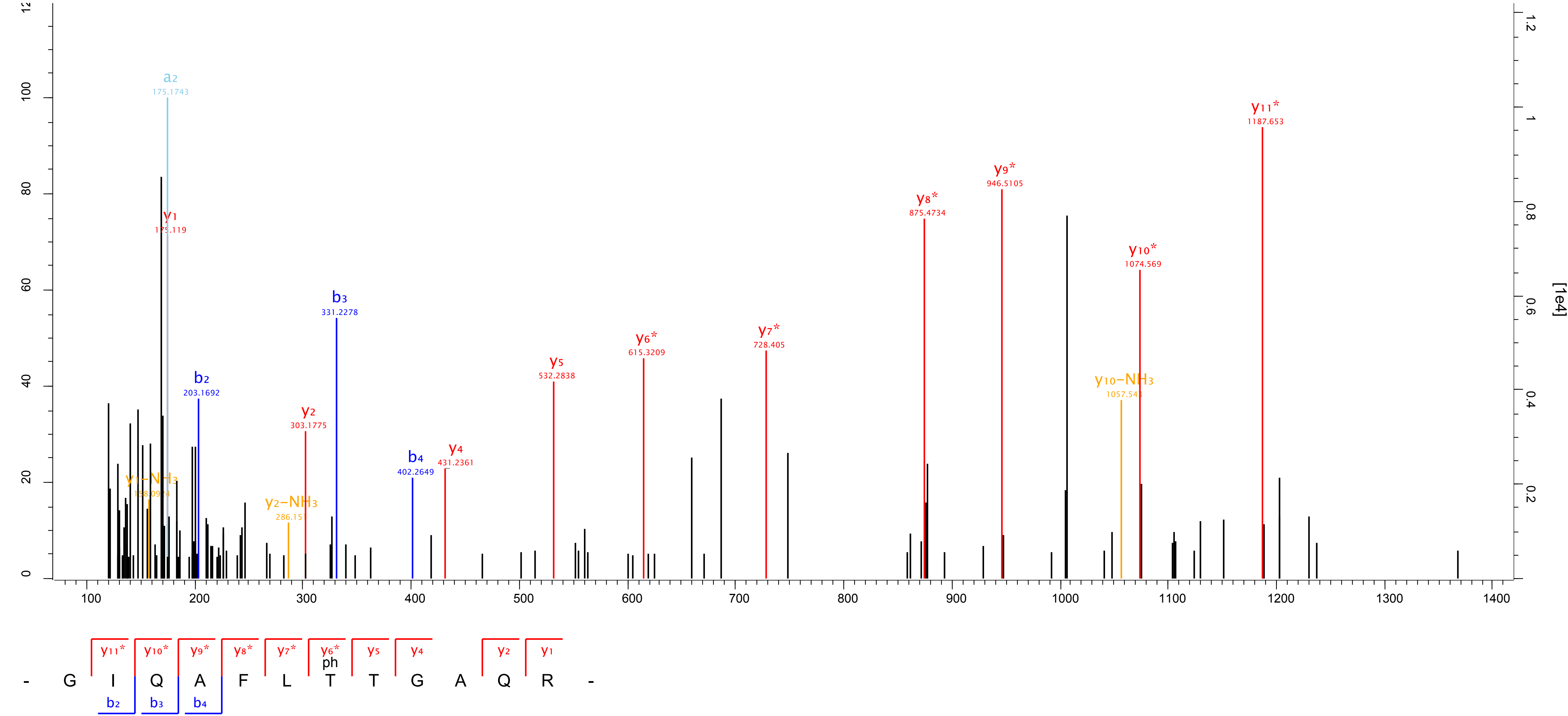

Raw file	Scan	Method	Score	m/z
20150122_PS_Syn_DimethylMix_Rep1_Phospho03	13655	FTMS; HCD	42.56	821.37

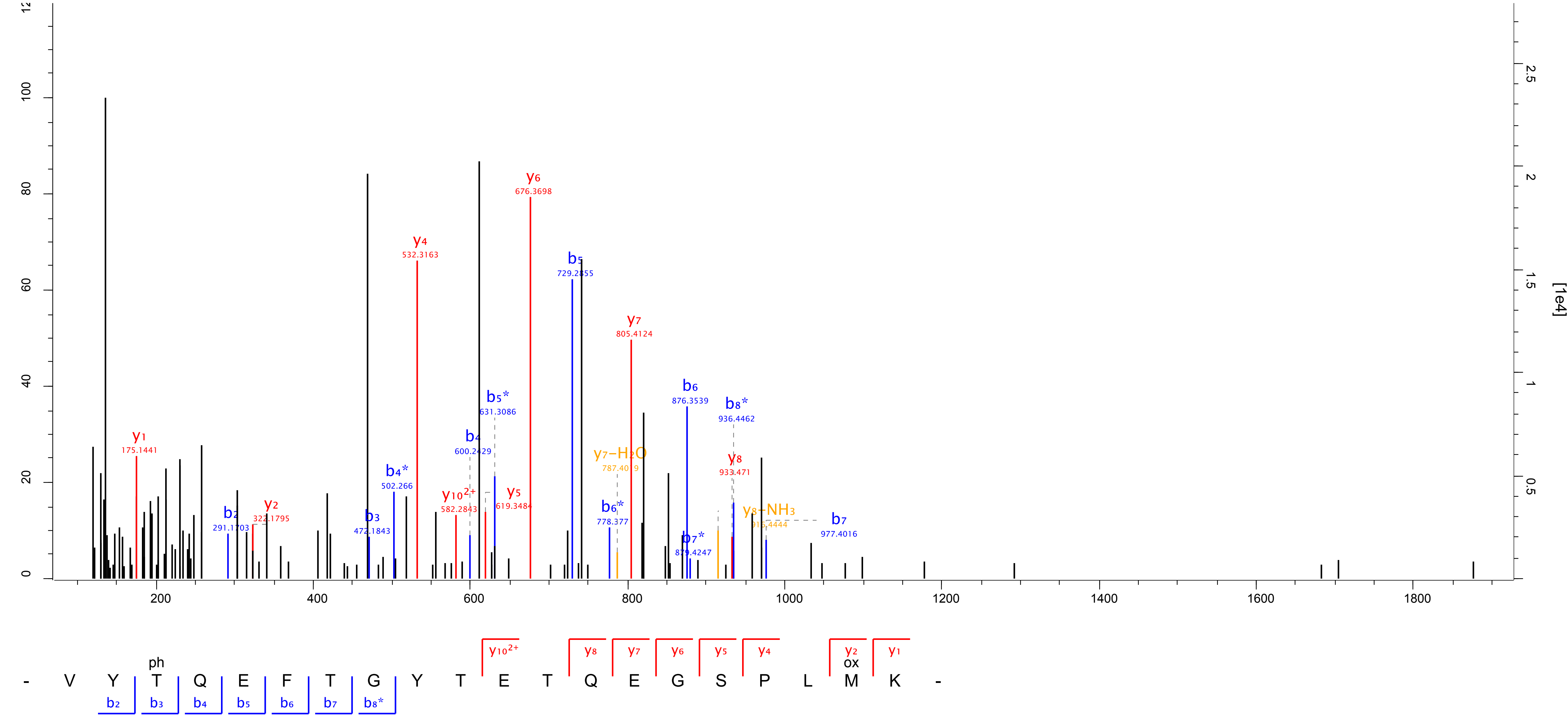

Raw file	Scan	Method	Score	m/z
20141031_PS_Syn6803wt_dimethyl_Mix_Phospho7	13726	FTMS; HCD	59.5	835.37

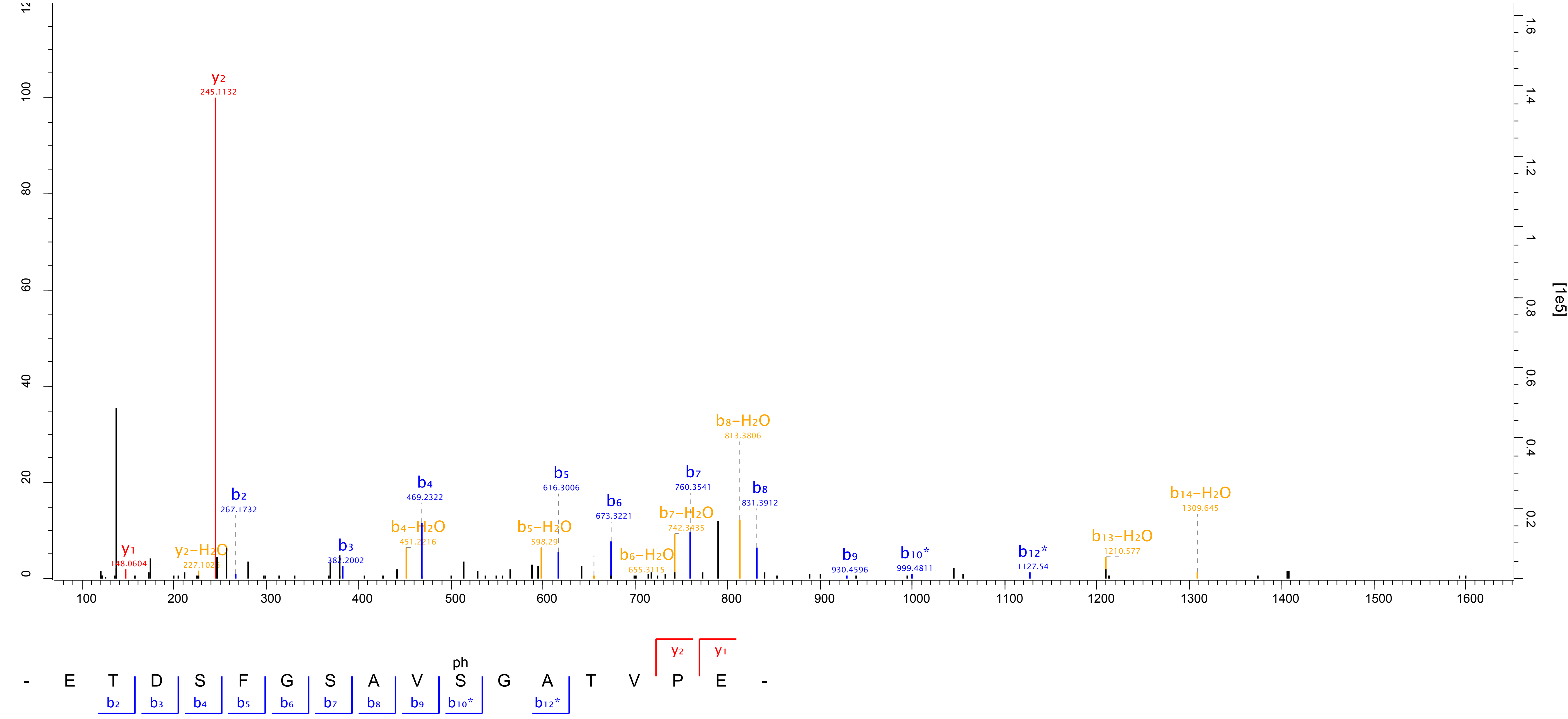

Raw file	Scan	Method	Score	m/z
20141031_PS_Syn6803wt_dimethyl_Mix_Phospho4	13979	FTMS; HCD	44.63	761.7

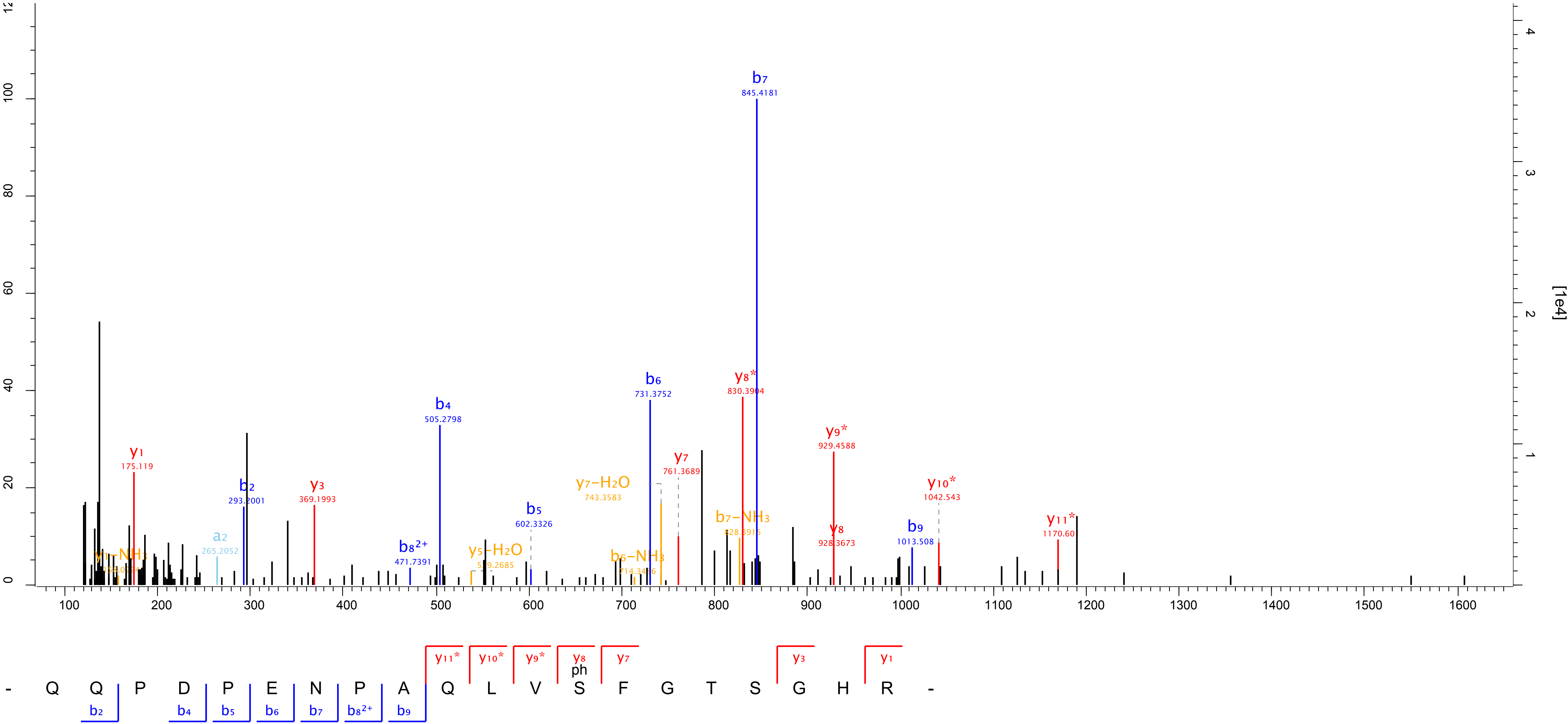

Raw file	Scan	Method	Score	m/z
20141031_PS_Syn6803wt_dimethyl_Mix_Phospho2	15843	FTMS; HCD	62.86	763.4

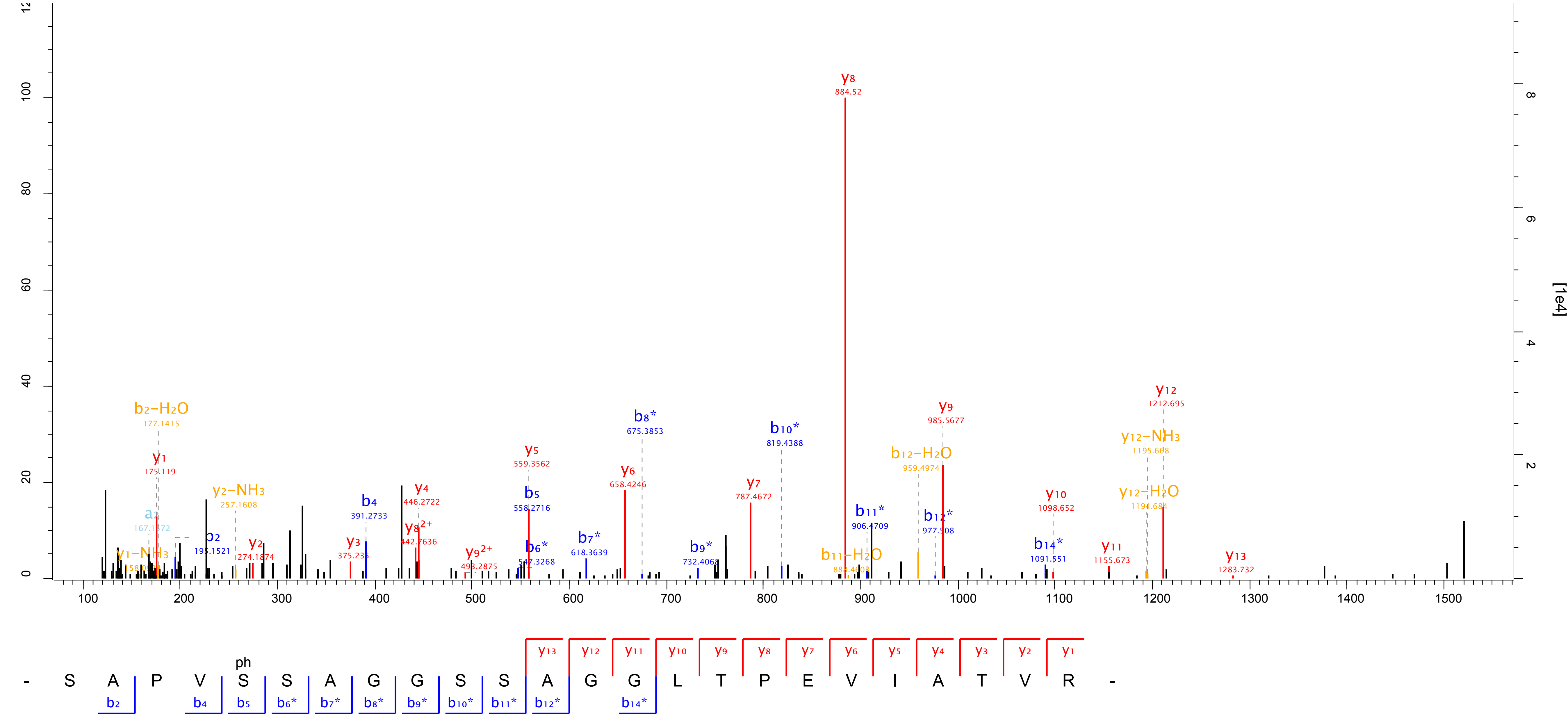

Raw file	Scan	Method	Score	m/z
20150122_PS_Syn_DimethylMix_Rep2_Phospho02	14884	FTMS; HCD	92.39	714.03

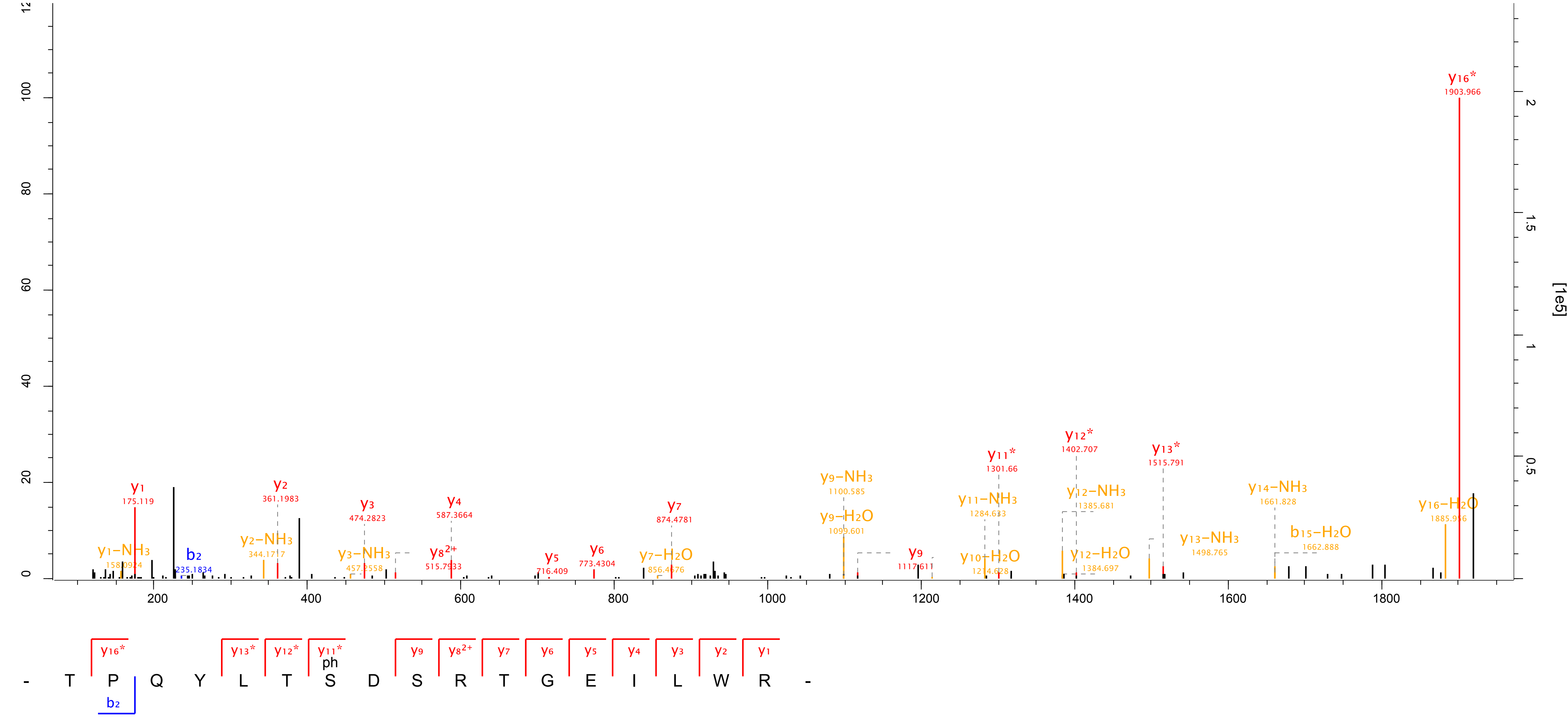

Raw file	Scan	Method	Score	m/z
20141031_PS_Syn6803wt_dimethyl_Mix_Phospho3	15002	FTMS; HCD	62.72	727.06

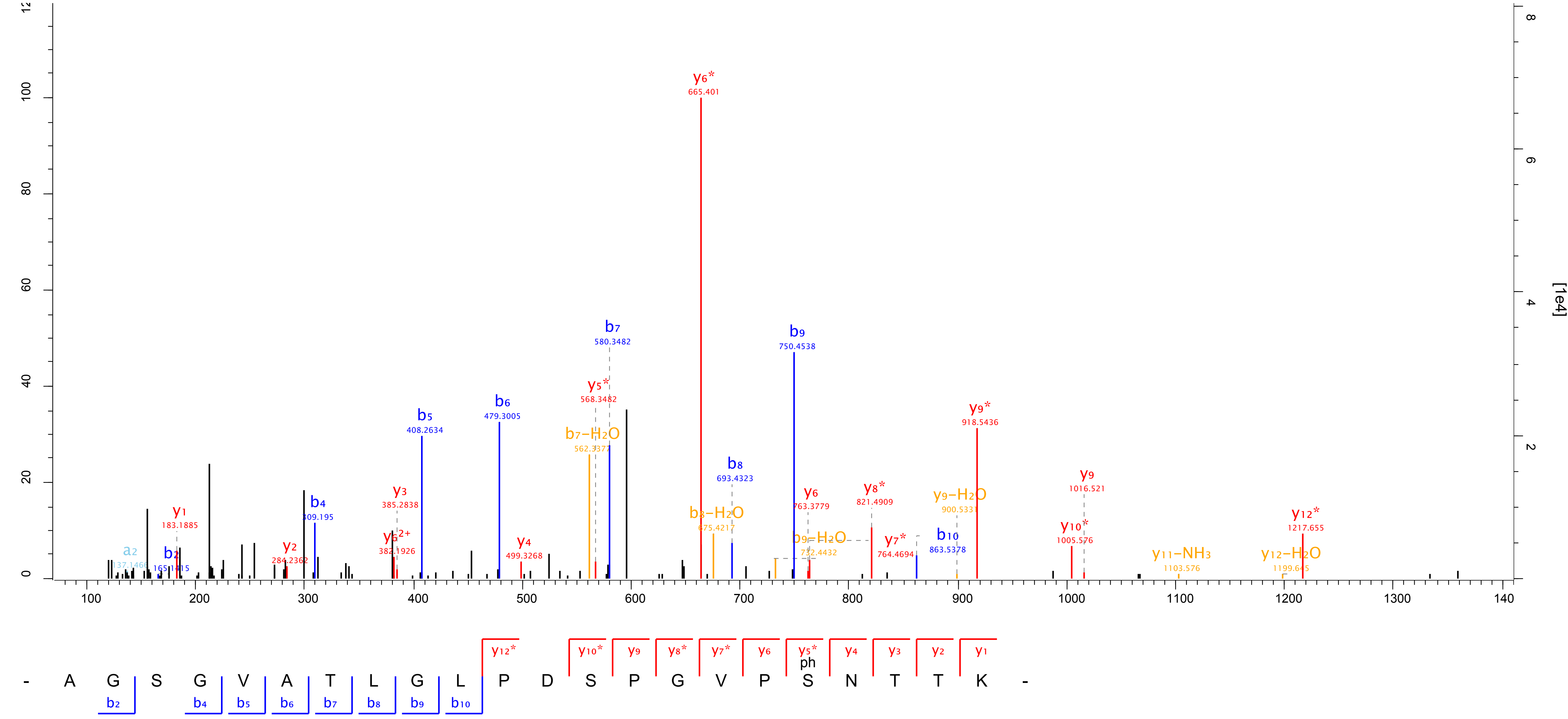

Raw file	Scan	Method	Score	m/z
20141031_PS_Syn6803wt_dimethyl_Mix_Phospho2	15694	FTMS; HCD	54.83	619.36

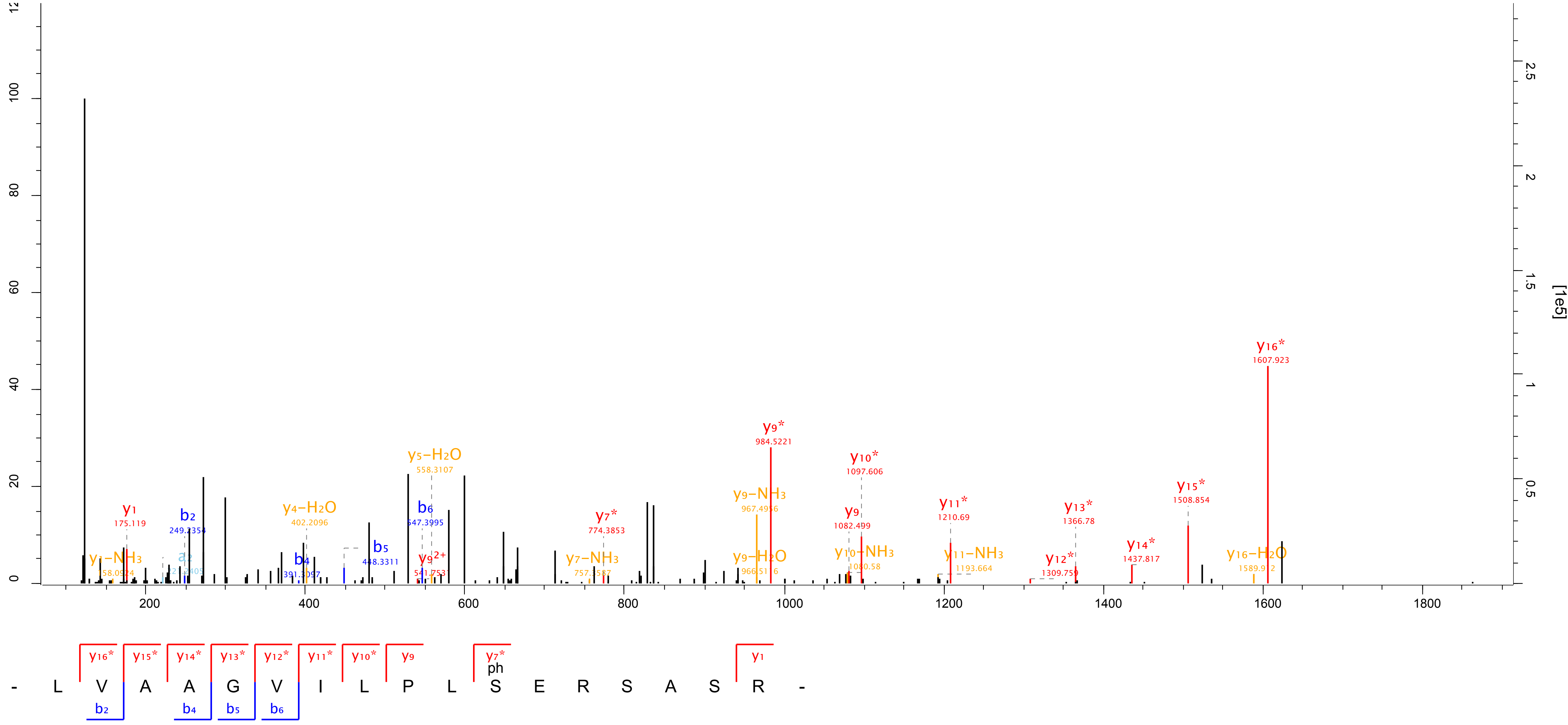

Raw file	Scan	Method	Score	m/z
20150122_PS_Syn_DimethylMix_Rep2_Phospho01	16094	FTMS; HCD	50.96	730.05

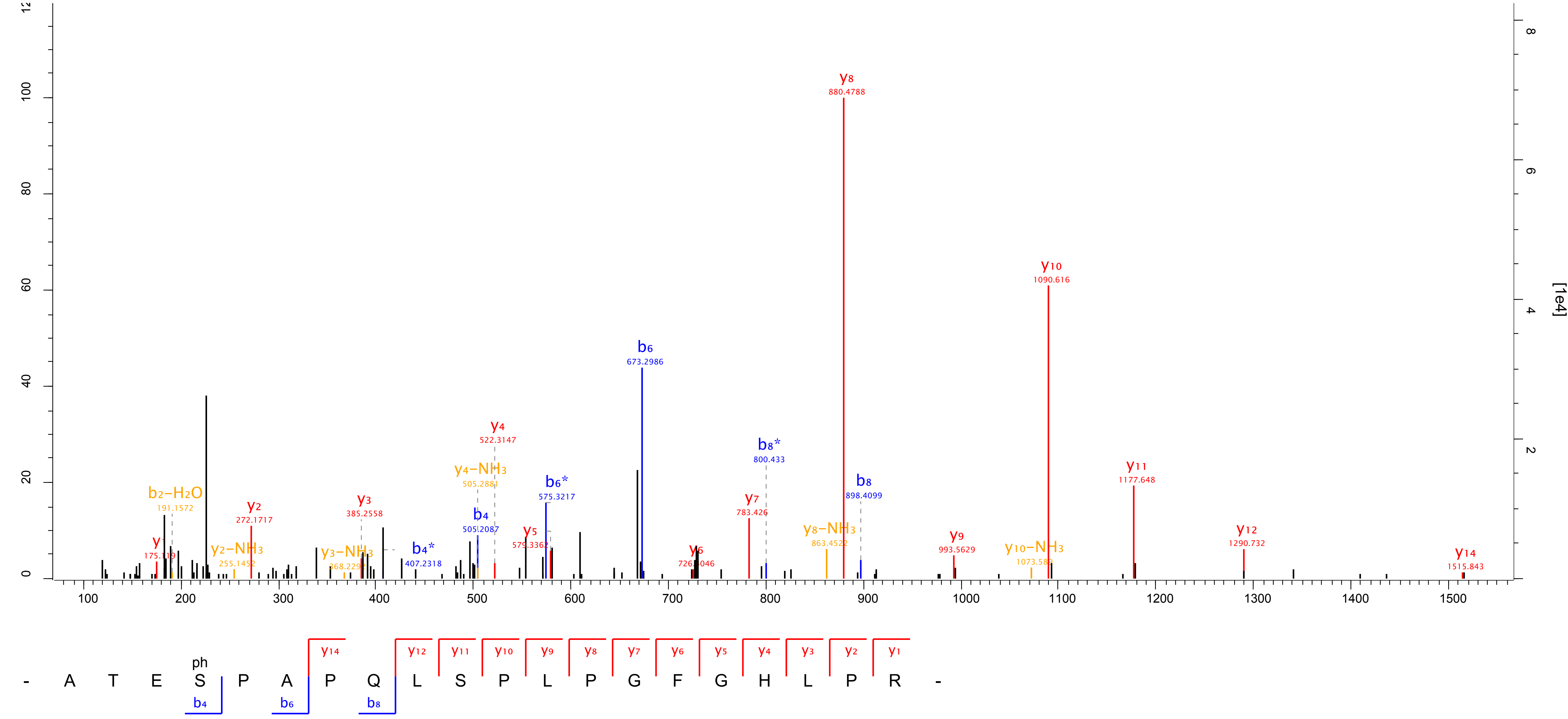

Raw file	Scan	Method	Score	m/z
20150122_PS_Syn_DimethylMix_Rep1_Phospho01	16138	FTMS; HCD	61.72	962.17

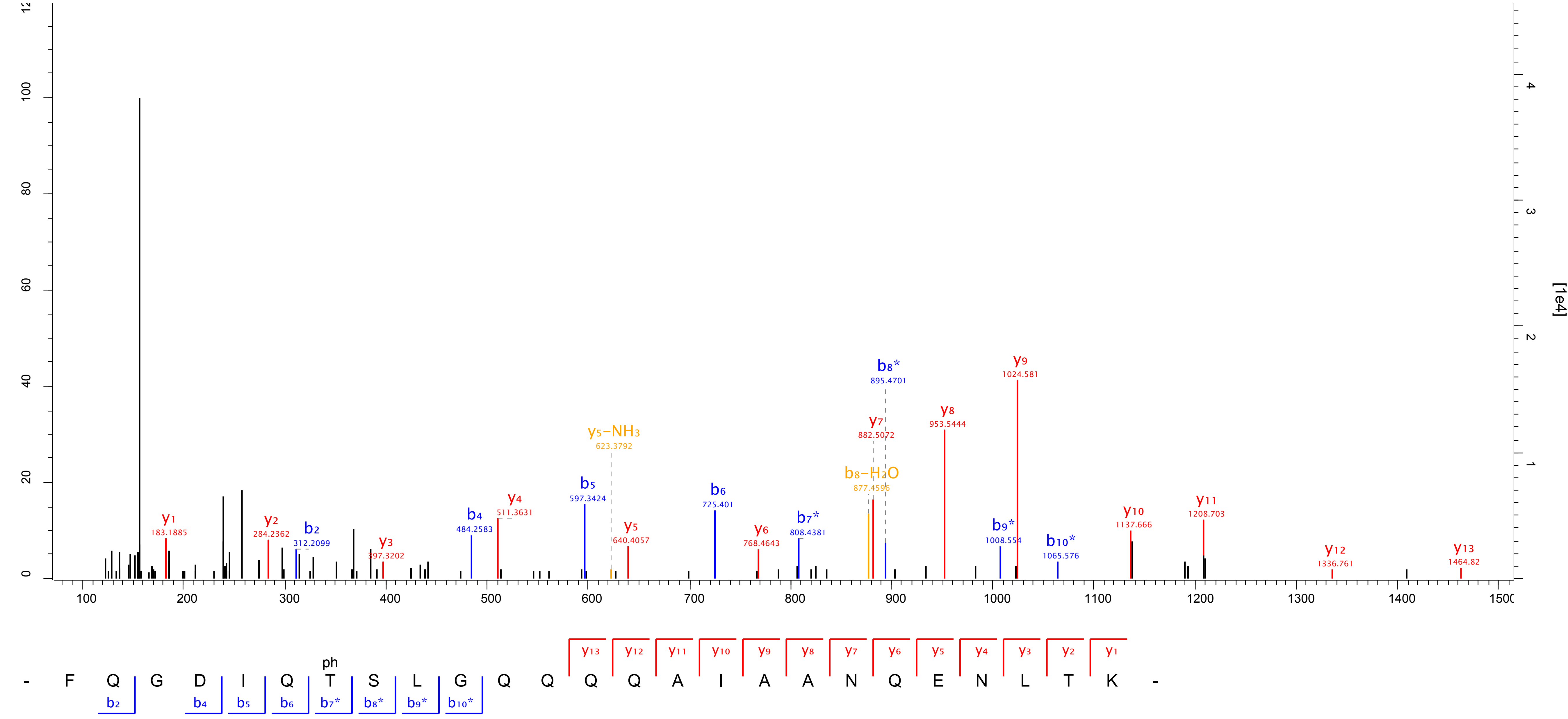

Raw file	Scan	Method	Score	m/z
20141031_PS_Syn6803wt_dimethyl_Mix_Phospho3	16143	FTMS; HCD	67.91	881.13

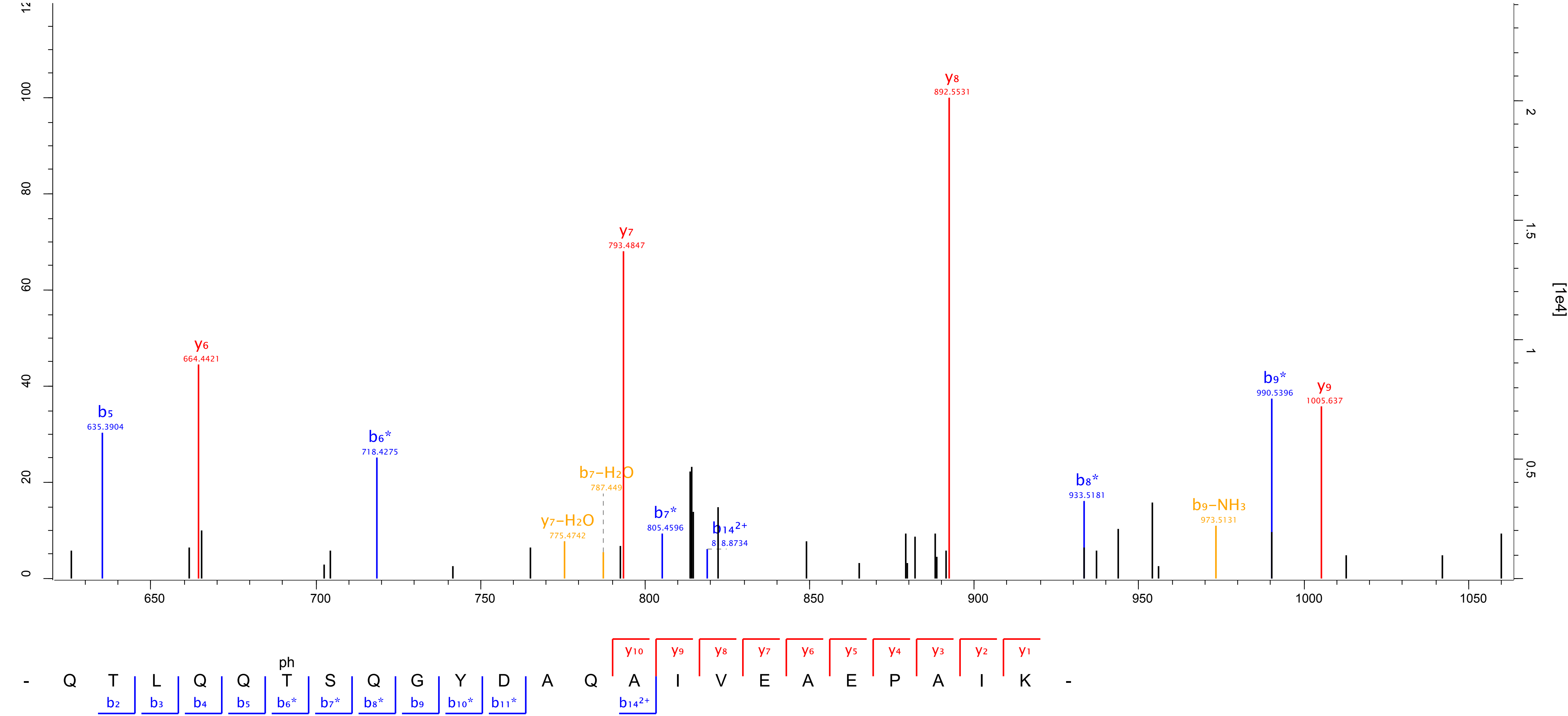

Raw file	Scan	Method	Score	m/z
201417_PS_Syn6803wt_dimethylMix_TiO2_1	16330	FTMS; HCD	57.14	1076.57

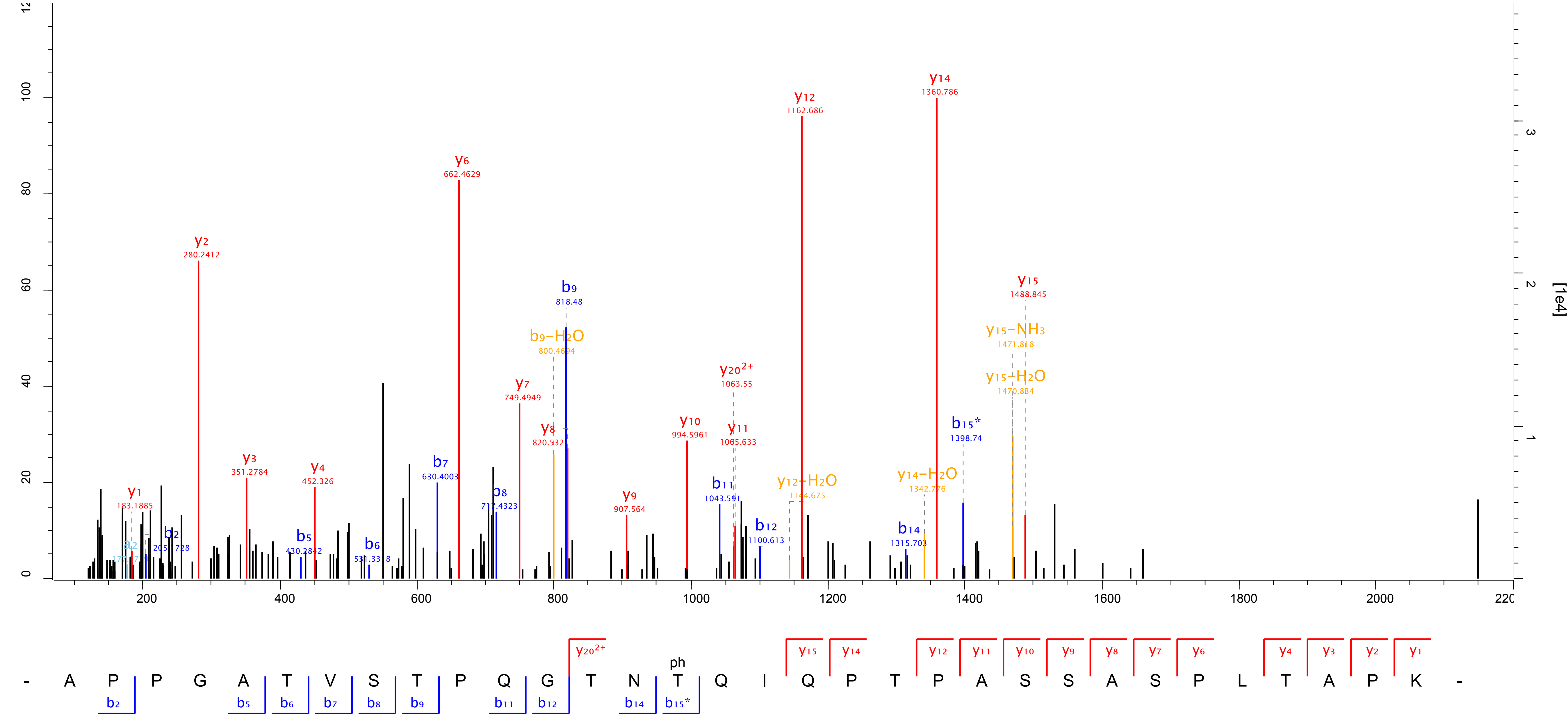

Raw file	Scan	Method	Score	m/z
201417_PS_Syn6803wt_dimethylMix_TiO2_2	16340	FTMS; HCD	115.8	1034.45

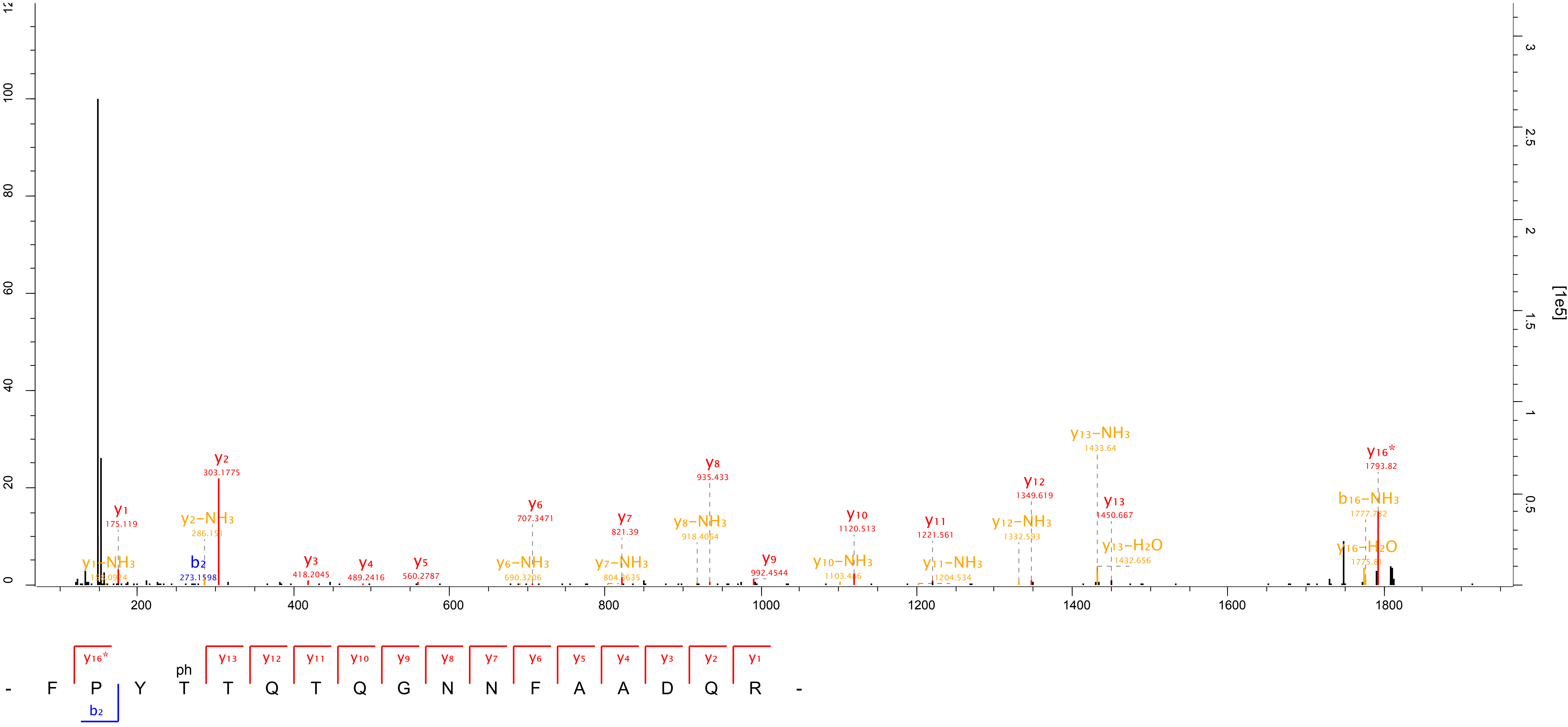

Raw file	Scan	Method	Score	m/z
201417_PS_Syn6803wt_dimethylMix_TiO2_6	16824	FTMS; HCD	88.94	992.96

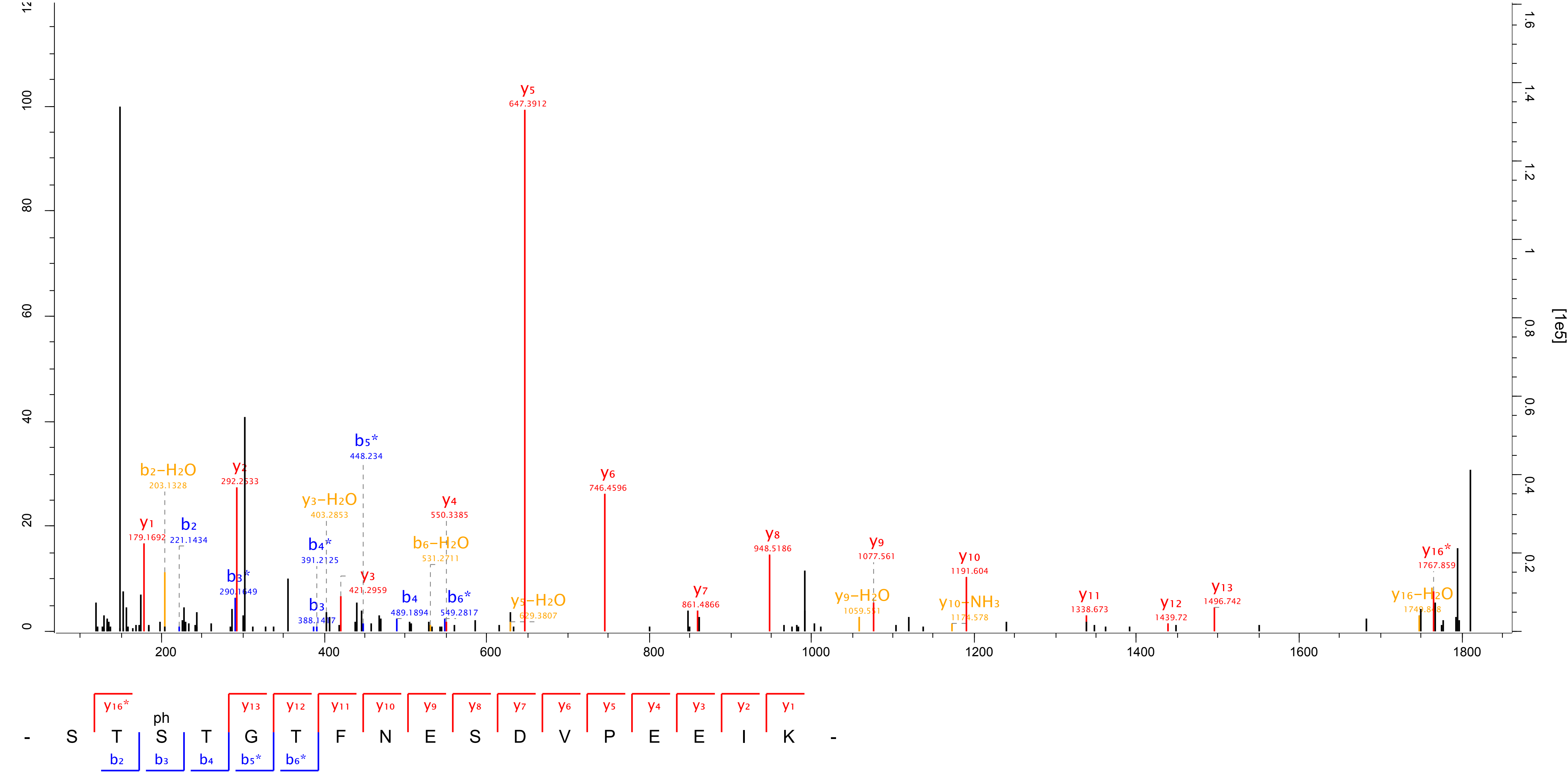

Raw file	Scan	Method	Score	m/z
20141031_PS_Syn6803wt_dimethyl_Mix_Phospho4	16760	FTMS; HCD	84.88	809.4

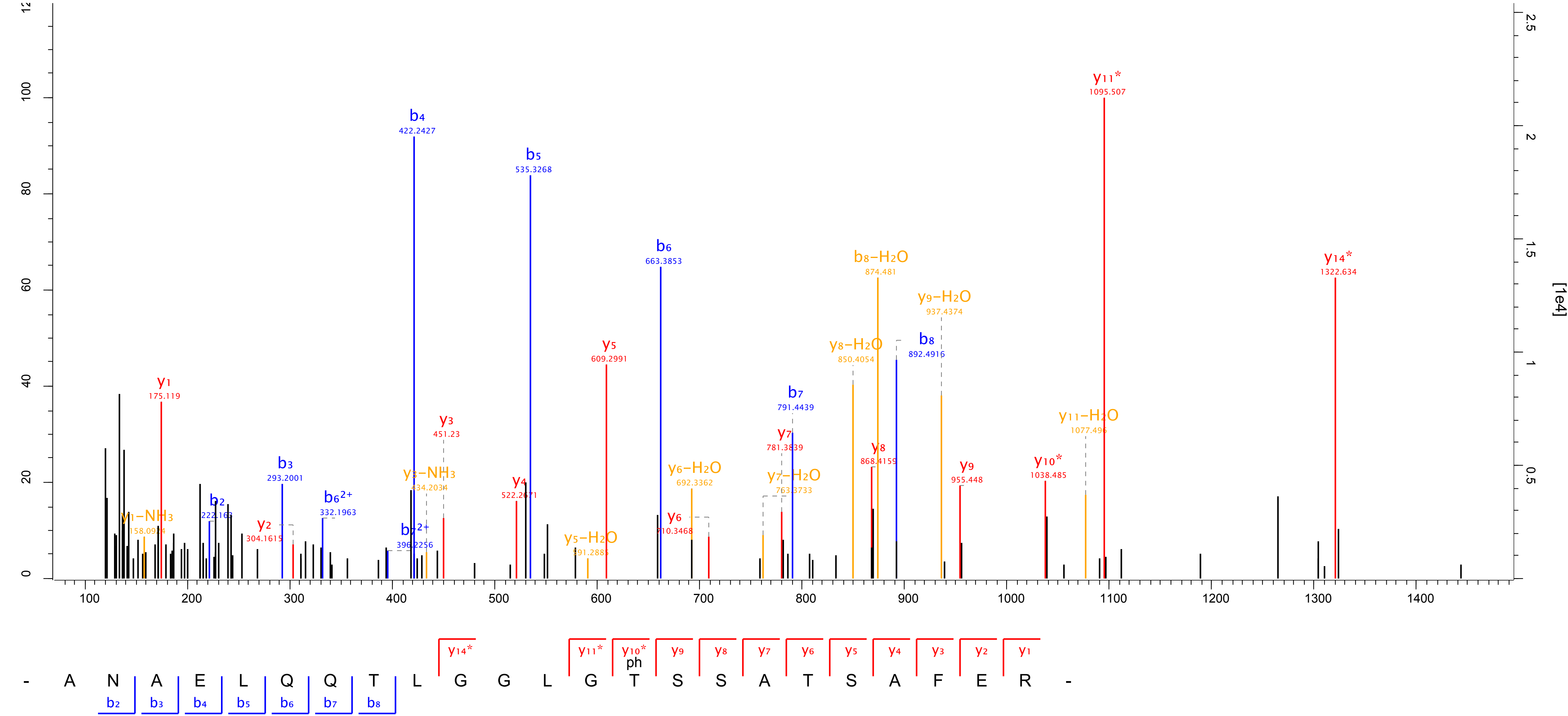

Raw file	Scan	Method	Score	m/z
20141031_PS_Syn6803wt_dimethyl_Mix_Phospho4	16744	FTMS; HCD	45.04	840.78

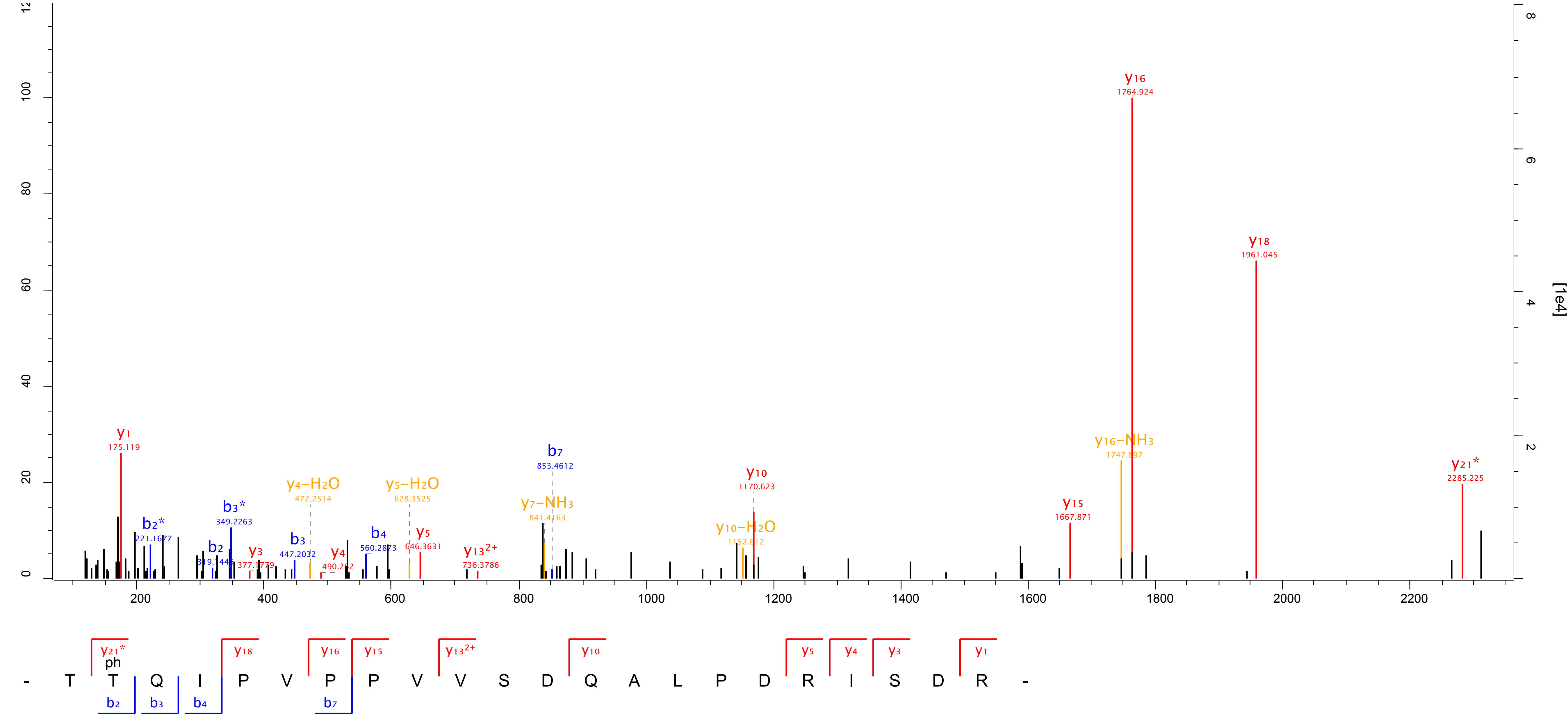

Raw file	Scan	Method	Score	m/z
20141031_PS_Syn6803wt_dimethyl_Mix_Phospho1	17994	FTMS; HCD	40.09	1045.73

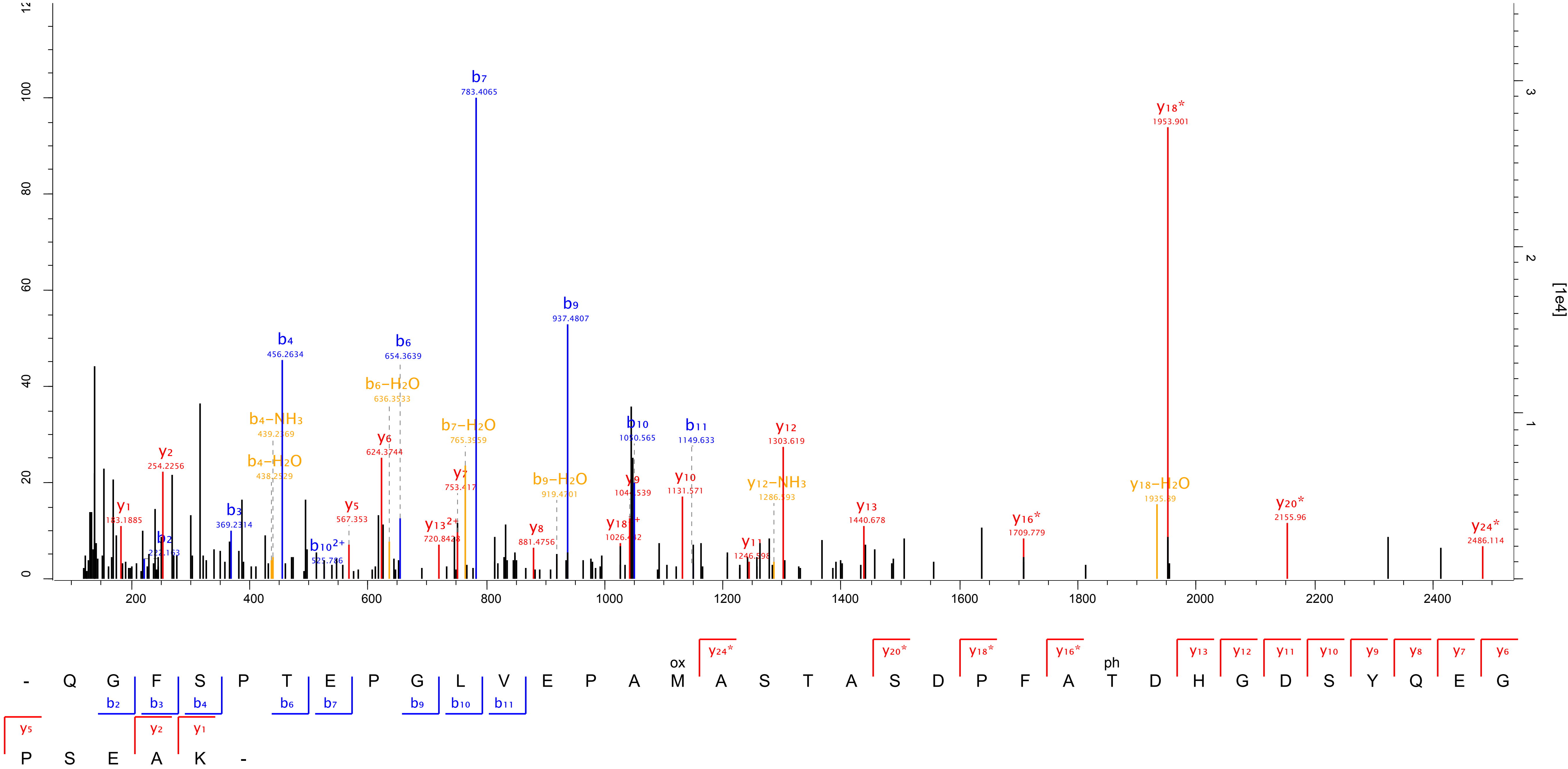

Raw file	Scan	Method	Score	m/z
20150122_PS_Syn_DimethylMix_Rep1_Phospho02	18051	FTMS; HCD	89.6	654.66

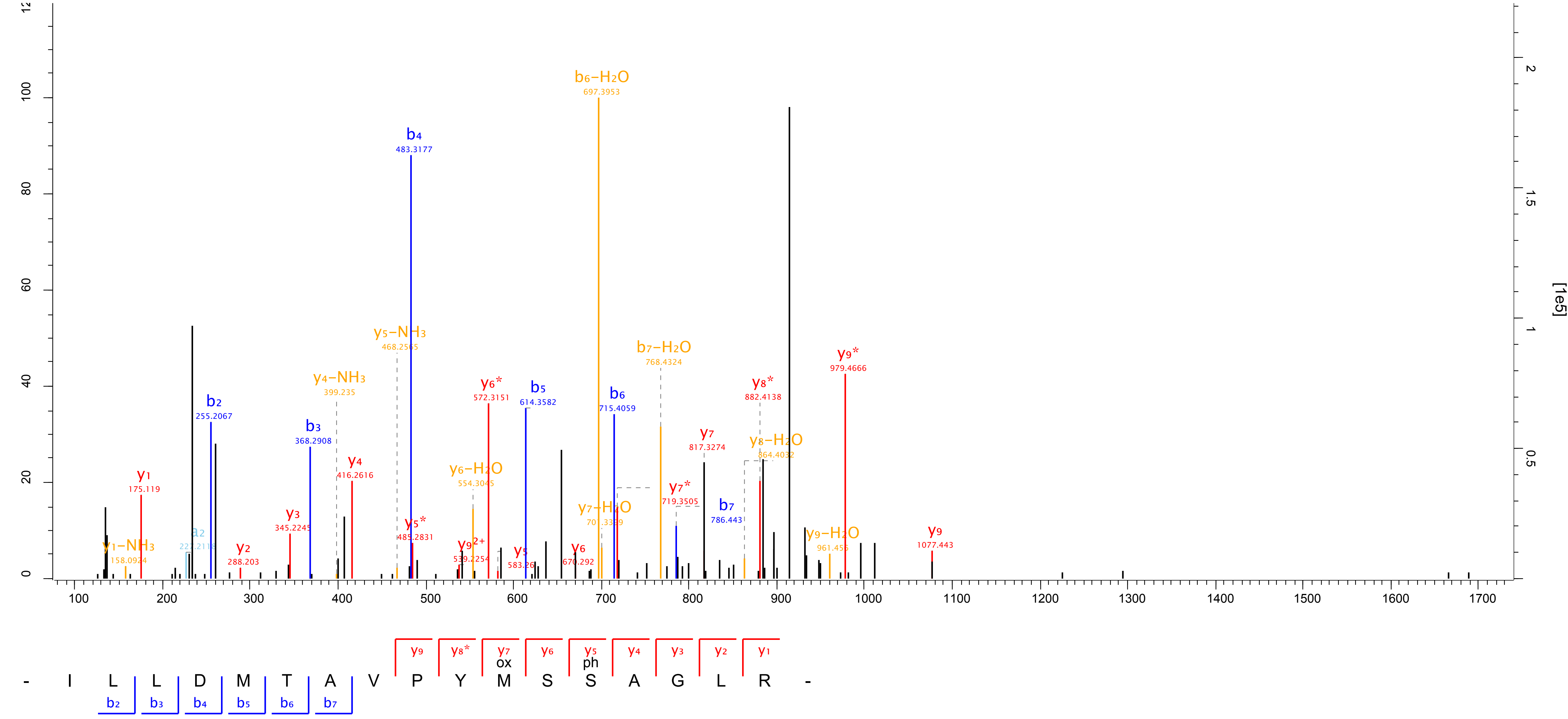

Raw file	Scan	Method	Score	m/z
20141031_PS_Syn6803wt_dimethyl_Mix_Phospho4	18508	FTMS; HCD	43.24	803.08

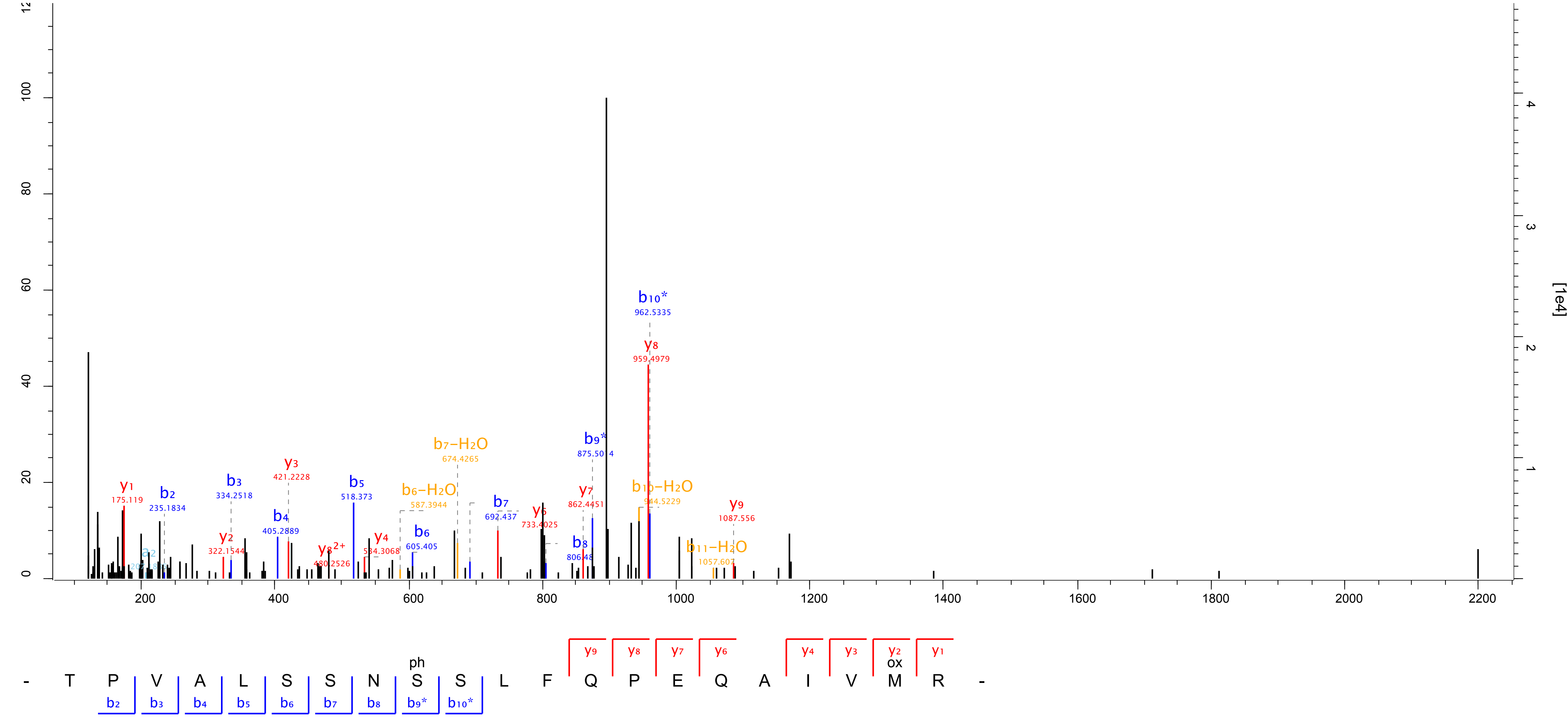

Raw file	Scan	Method	Score	m/z
20141031_PS_Syn6803wt_dimethyl_Mix_Phospho7	18756	FTMS; HCD	70.34	823.45

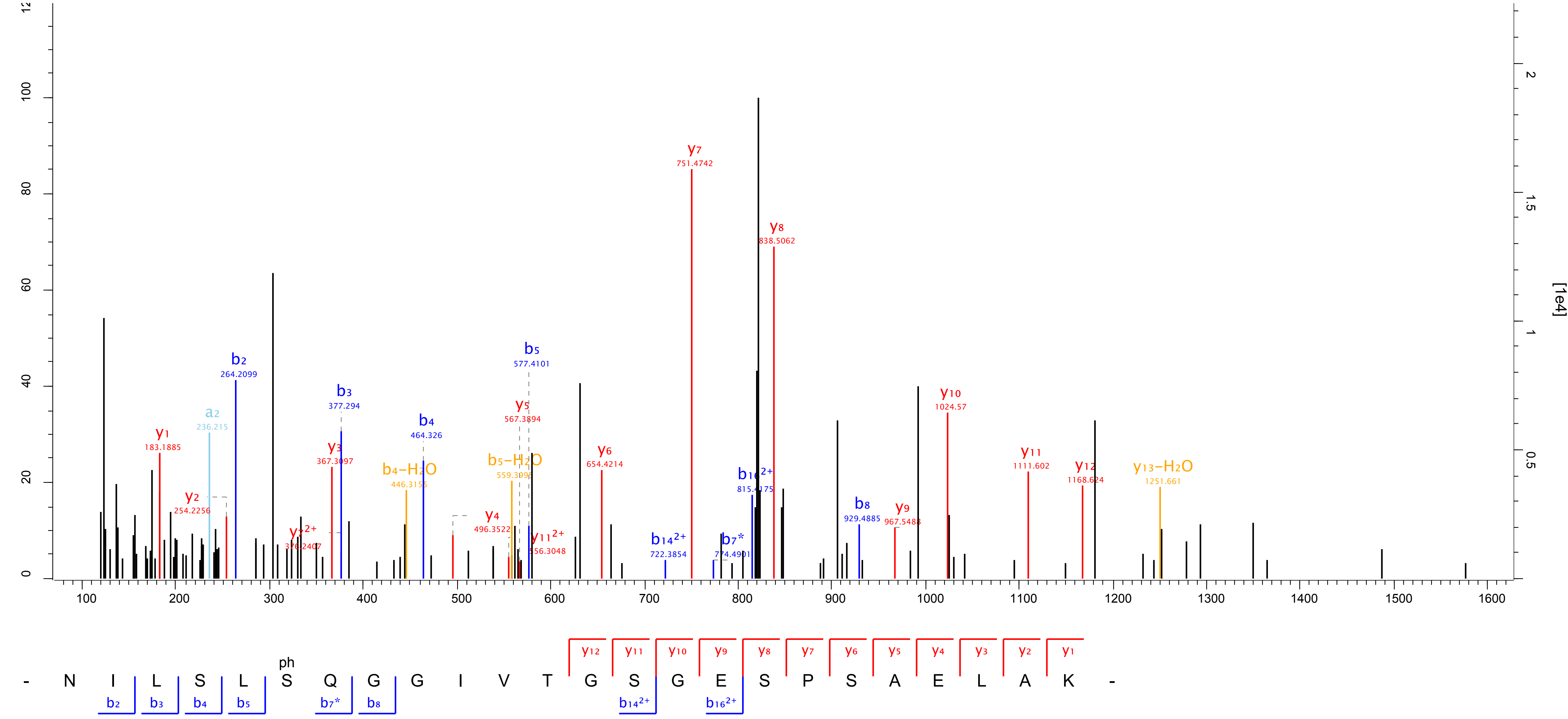

Raw file	Scan	Method	Score	m/z
20141031_PS_Syn6803wt_dimethyl_Mix_Phospho2	19717	FTMS; HCD	42.06	895.45

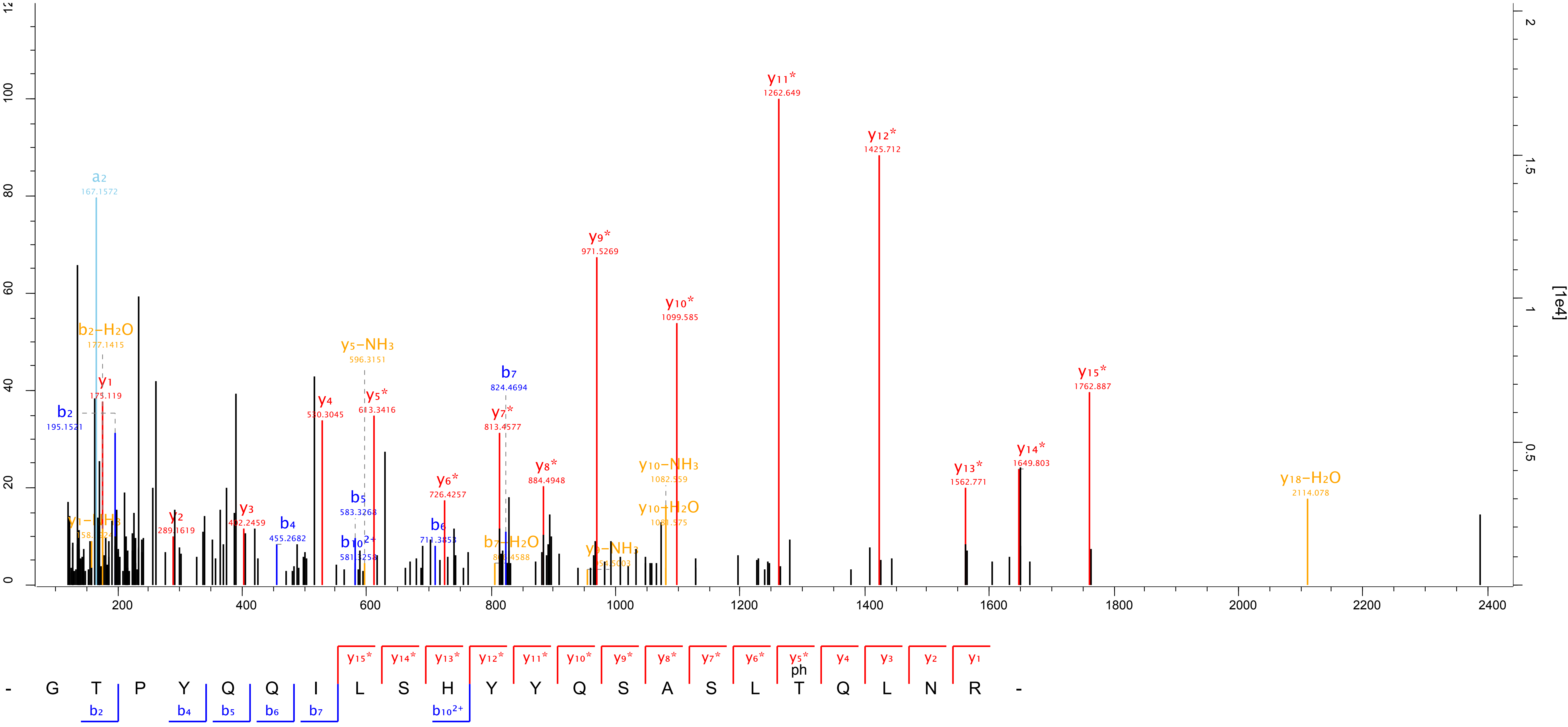

Raw file	Scan	Method	Score	m/z
20150122_PS_Syn_DimethylMix_Rep1_Phospho01	19844	FTMS; HCD	45.36	832.39

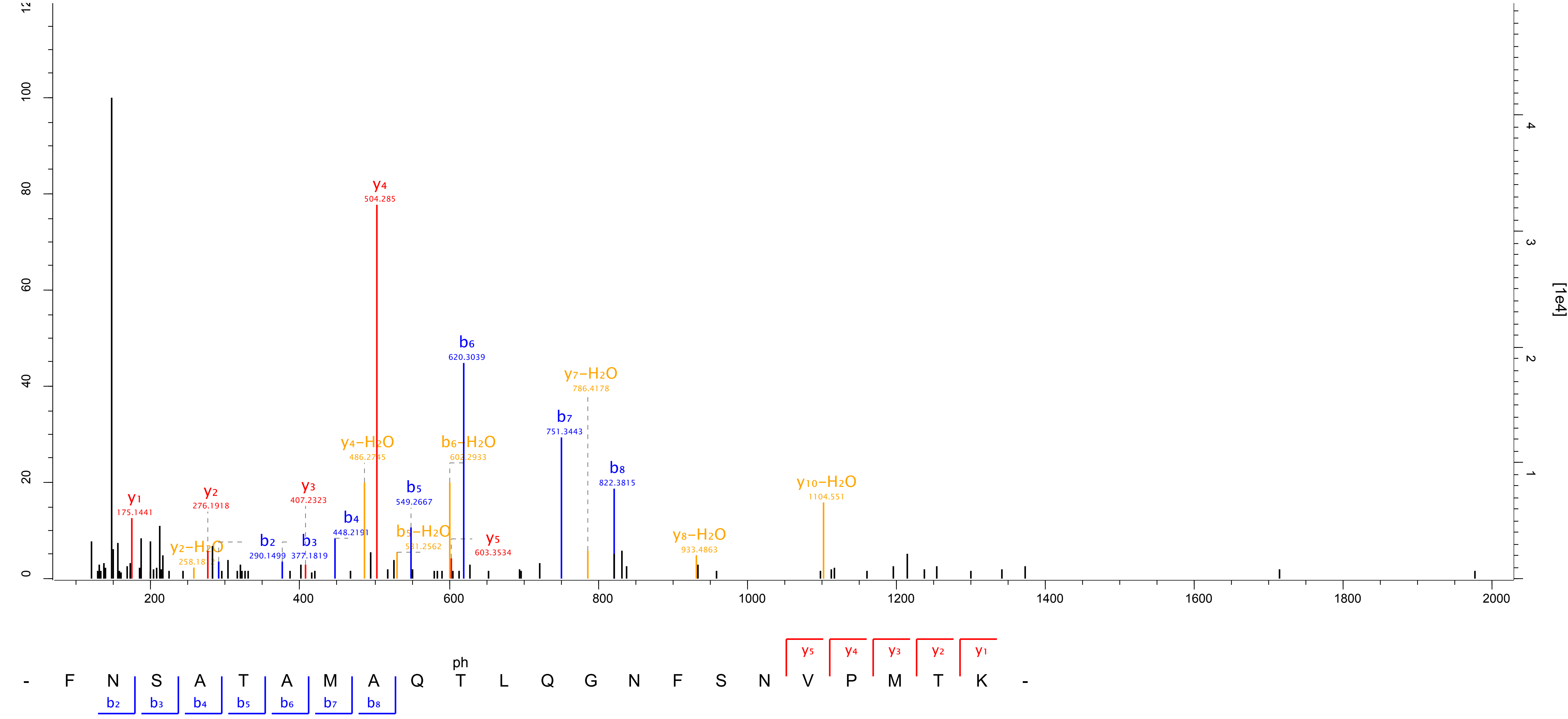

Raw file	Scan	Method	Score	m/z
20141031_PS_Syn6803wt_dimethyl_Mix_Phospho1	19868	FTMS; HCD	61.08	850.15

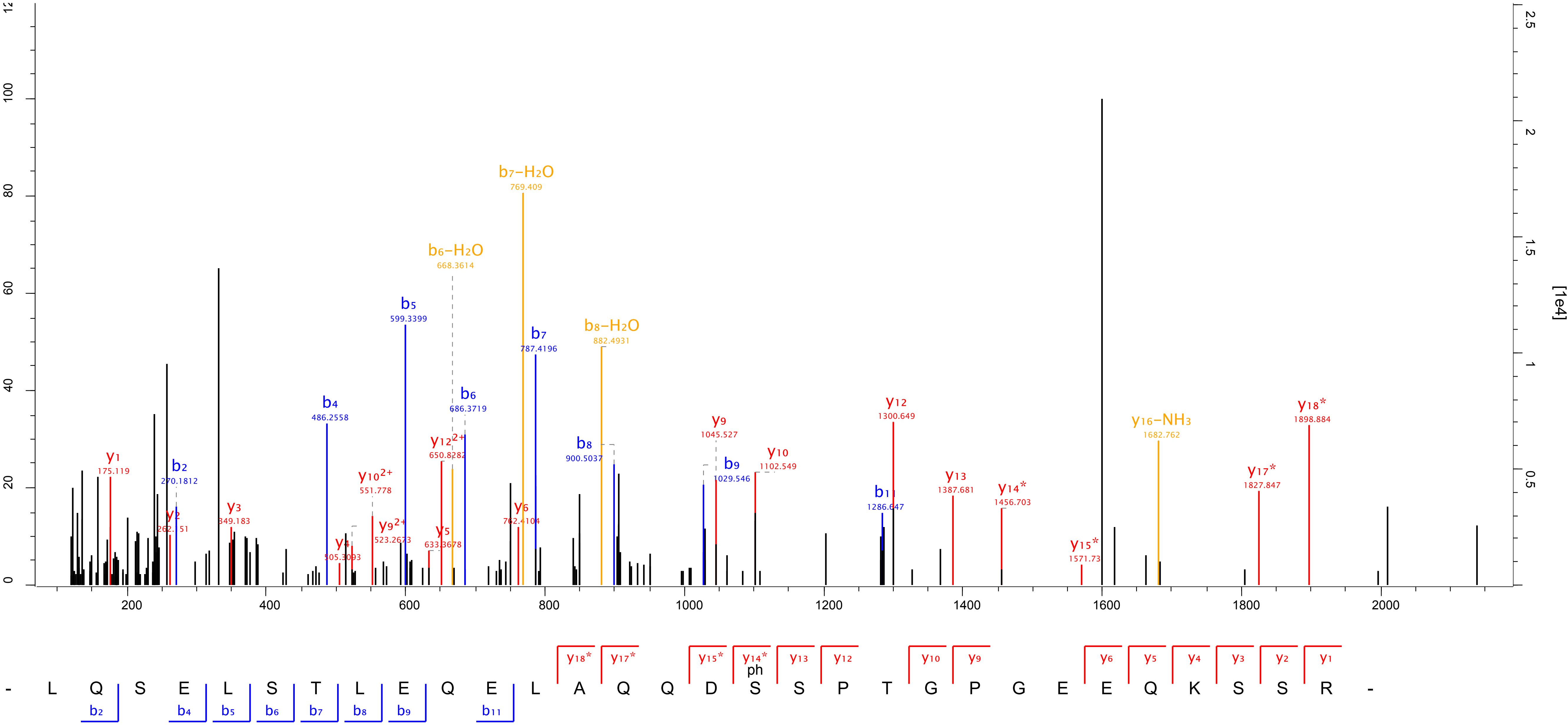

Raw file	Scan	Method	Score	m/z
20141031_PS_Syn6803wt_dimethyl_Mix_Phospho3	21033	FTMS; HCD	72.39	855.7

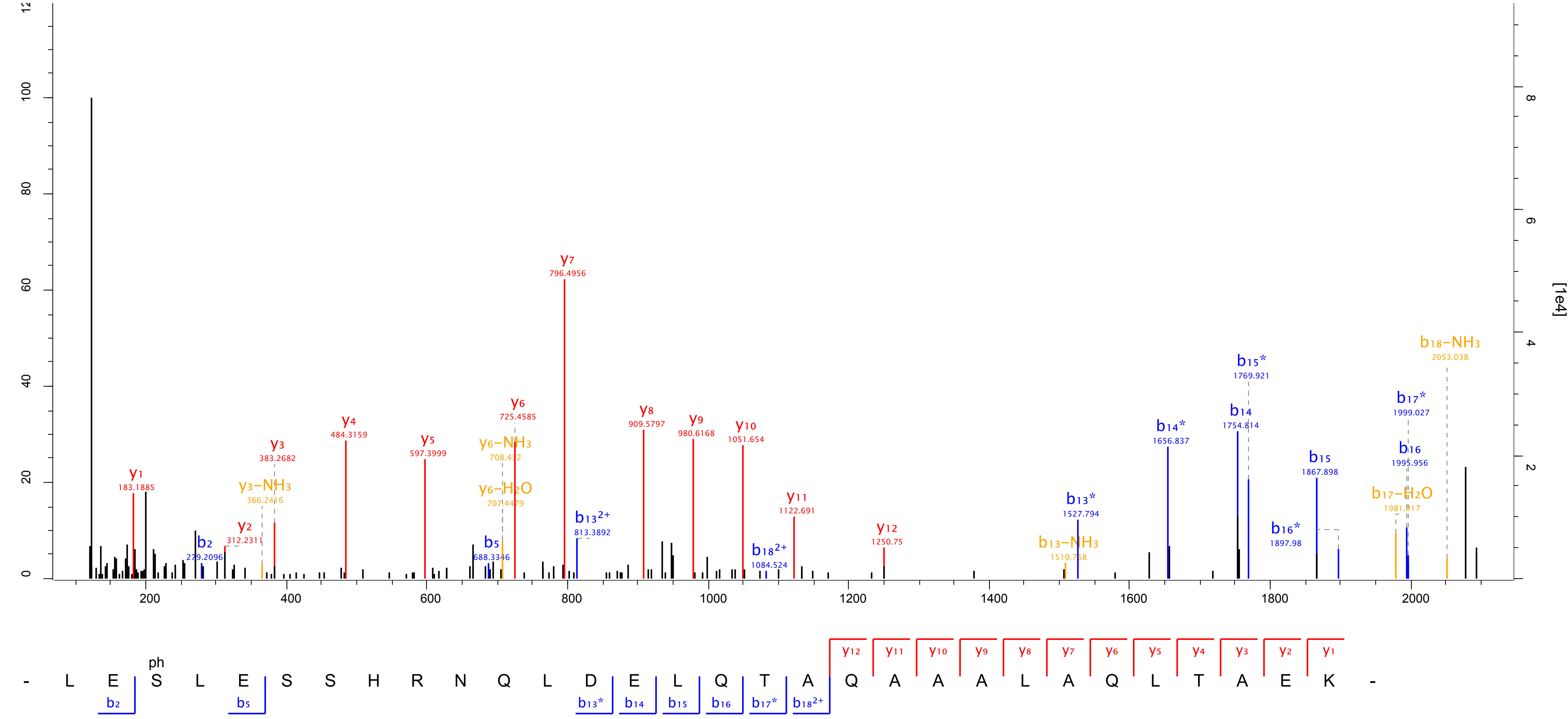

Raw file	Scan	Method	Score	m/z
20141031_PS_Syn6803wt_dimethyl_Mix_Phospho4	21370	FTMS; HCD	79.49	697

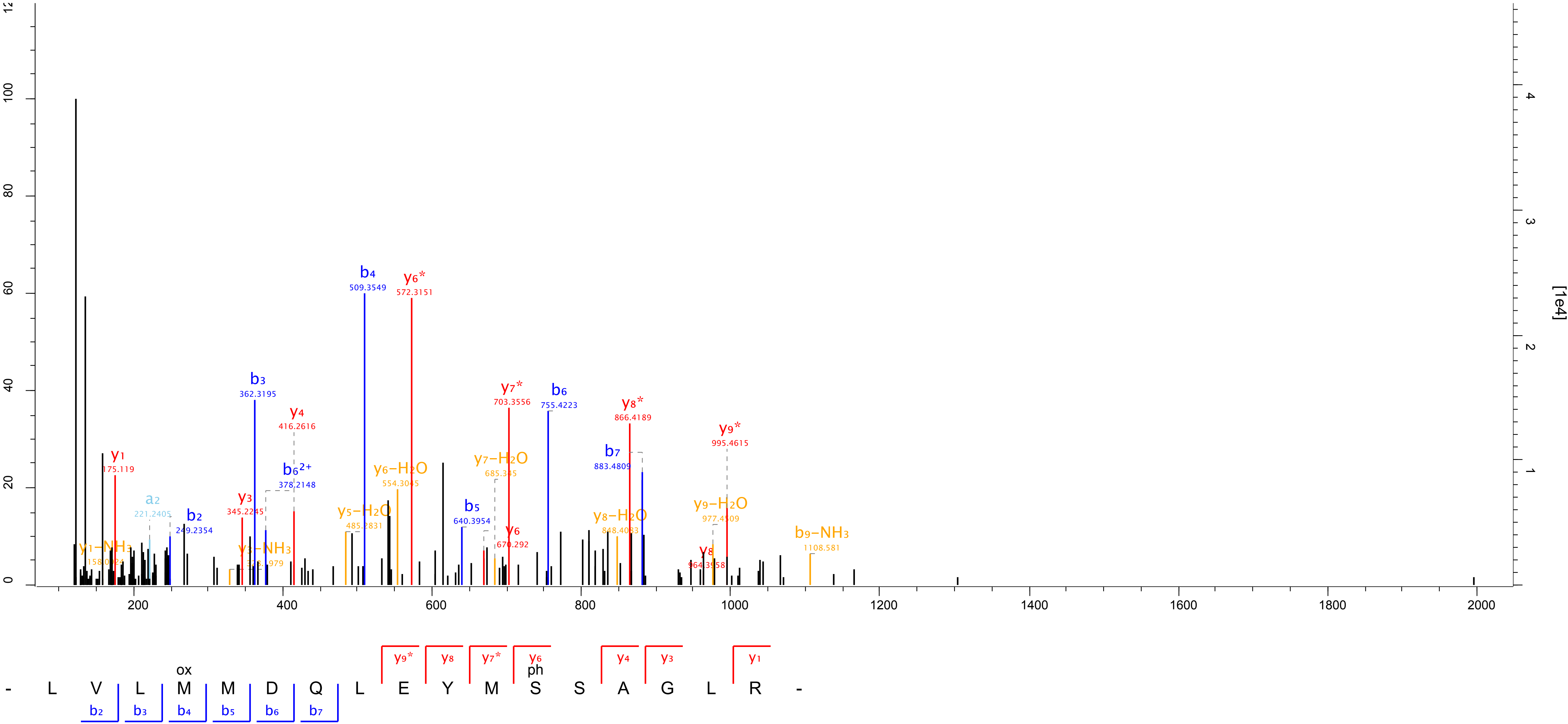

Raw file	Scan	Method	Score	m/z
20141031_PS_Syn6803wt_dimethyl_Mix_Phospho8	22080	FTMS; HCD	86.08	791.44

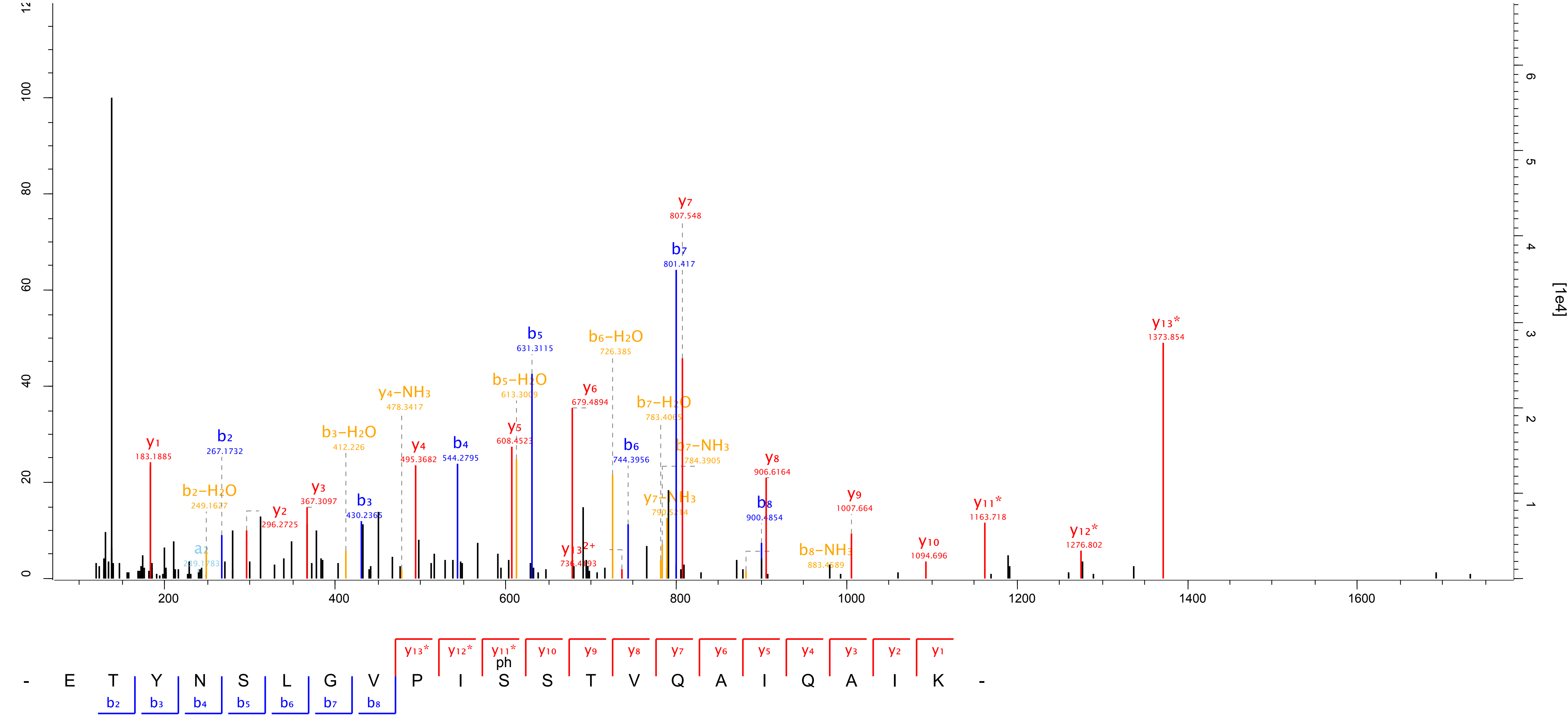

Raw file	Scan	Method	Score	m/z
20150122_PS_Syn_DimethylMix_Rep1_Phospho01	22633	FTMS; HCD	58	1034.26

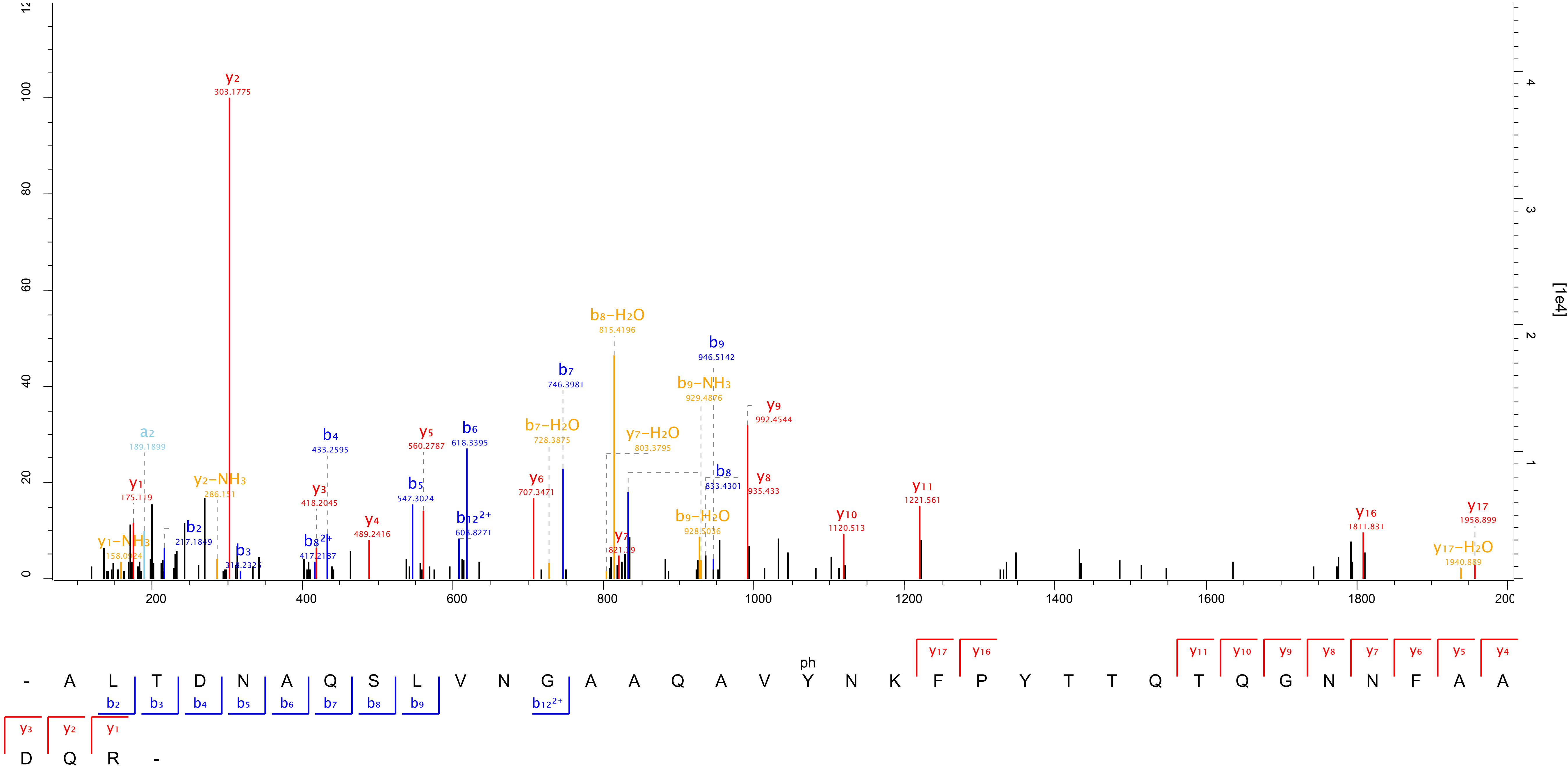

Raw file	Scan	Method	Score	m/z
20150122_PS_Syn_DimethylMix_Rep1_Phospho02	22852	FTMS; HCD	46.82	781.03

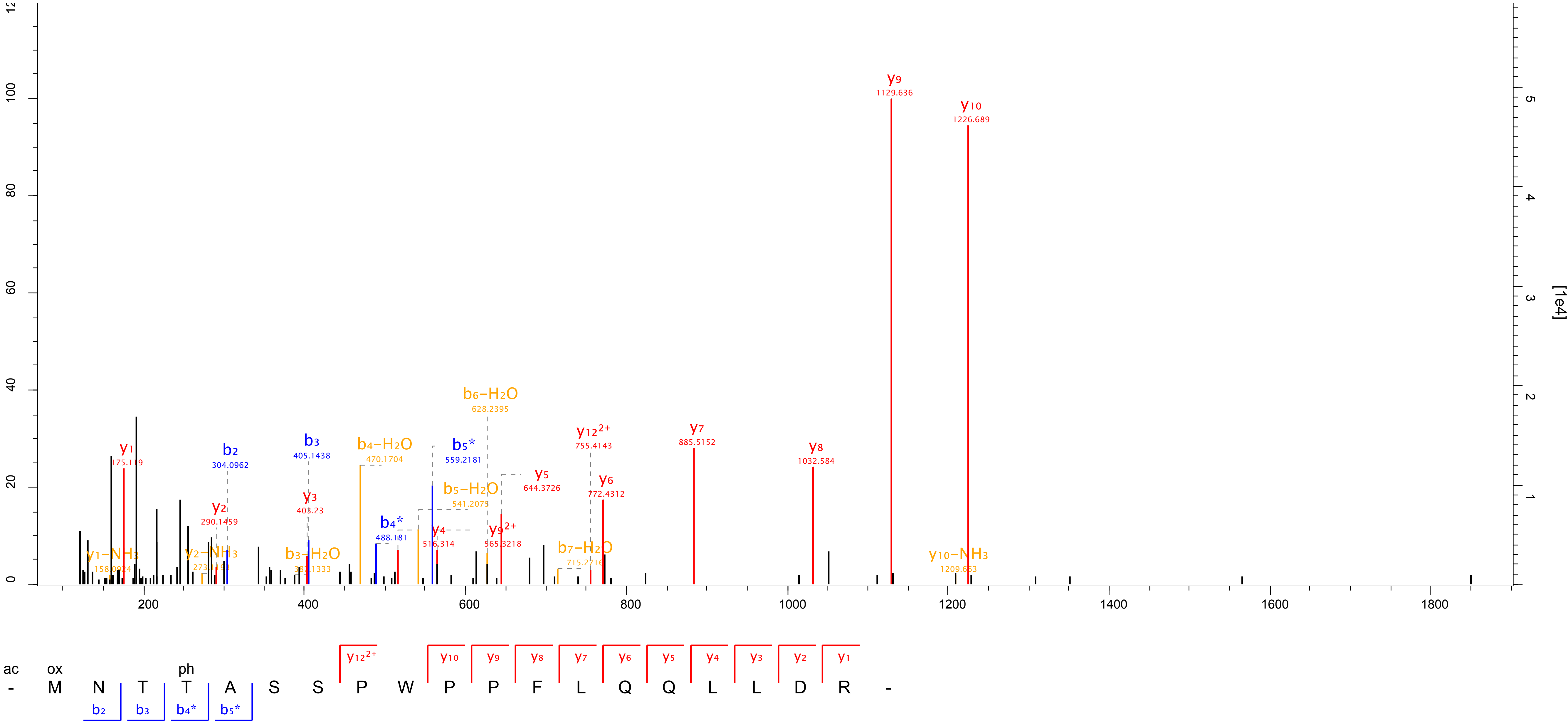

Raw file	Scan	Method	Score	m/z
20141031_PS_Syn6803wt_dimethyl_Mix_Phospho5	23448	FTMS; HCD	62.78	777.62

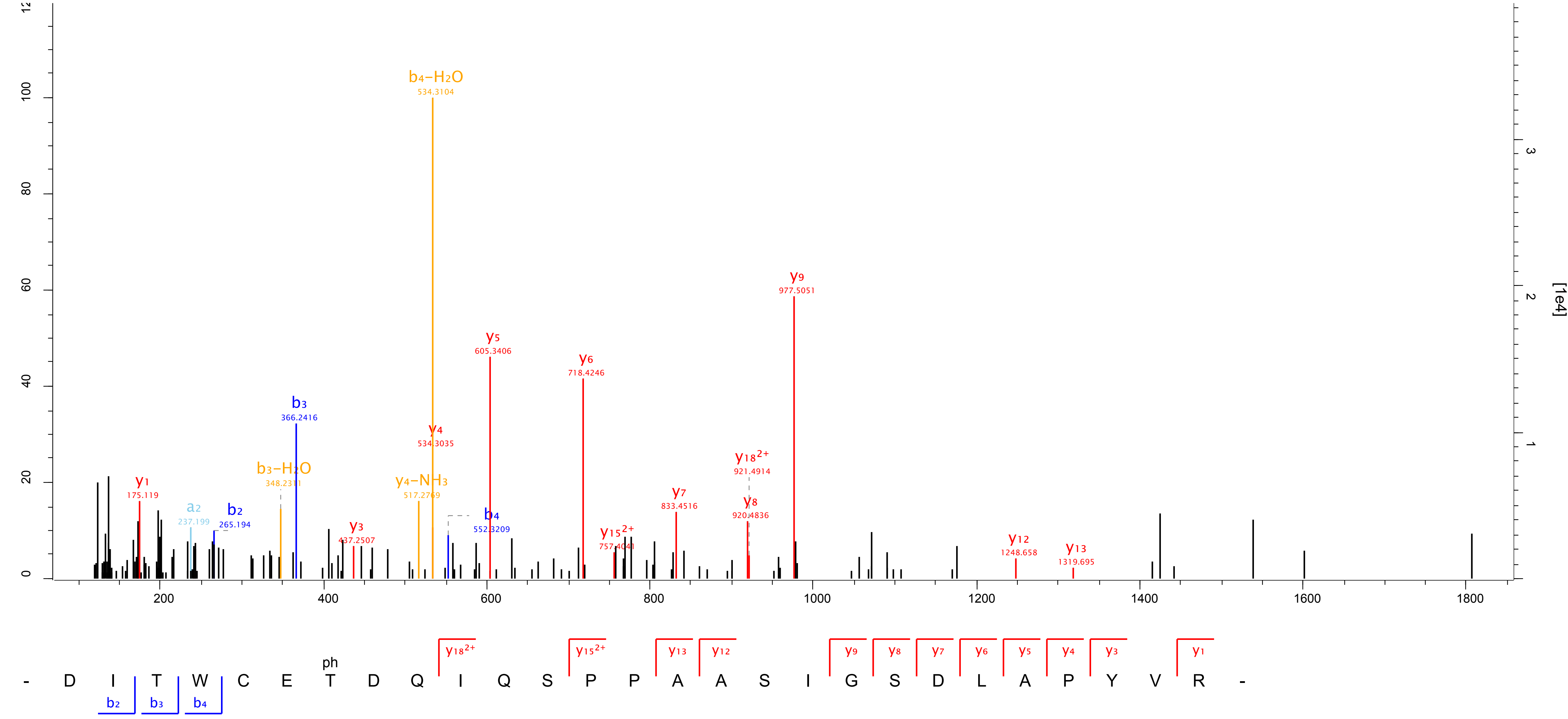

Raw file	Scan	Method	Score	m/z
20141031_PS_Syn6803wt_dimethyl_Mix_Phospho1	24841	FTMS; HCD	42.47	934.15

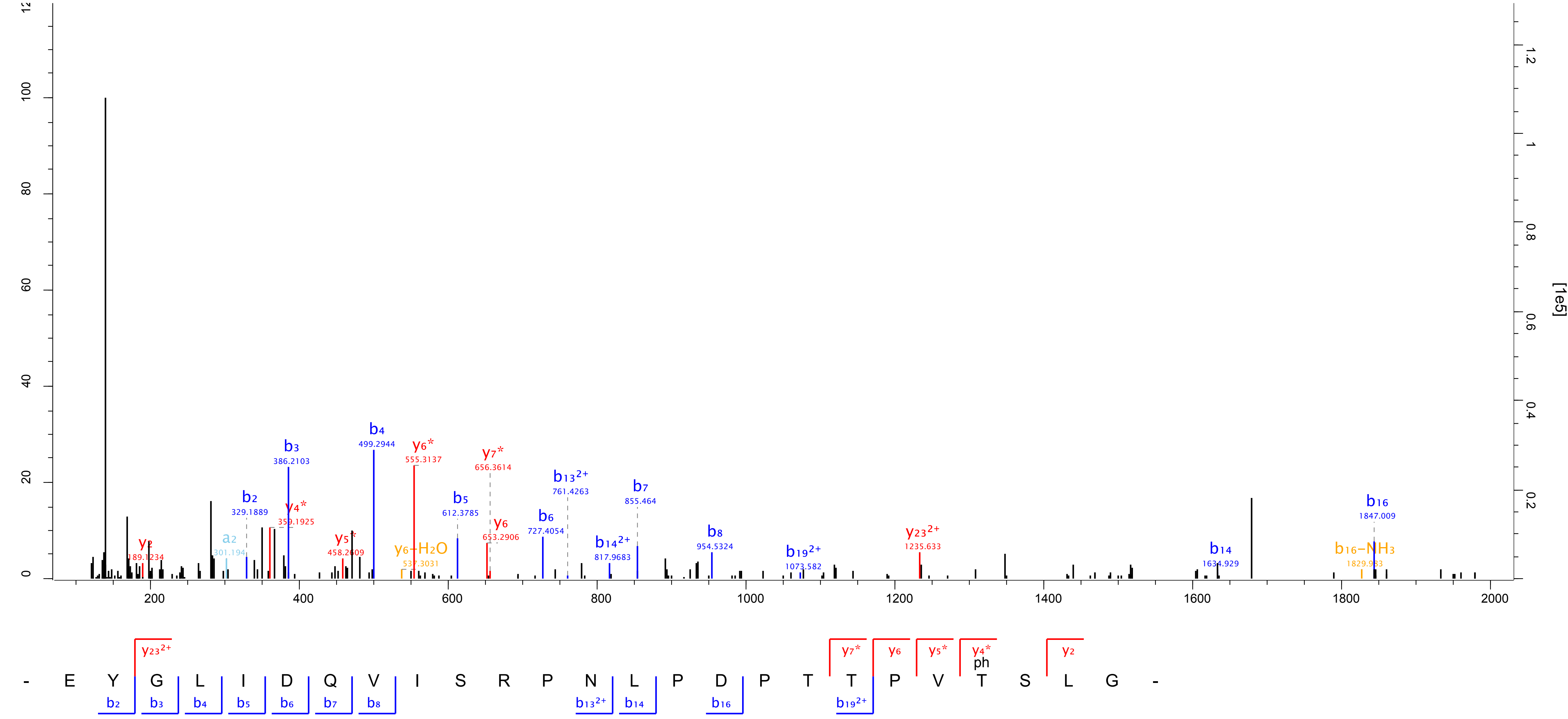

Raw file	Scan	Method	Score	m/z
201417_PS_Syn6803wt_dimethylMix_TiO2_4	28692	FTMS; HCD	44.79	973.98

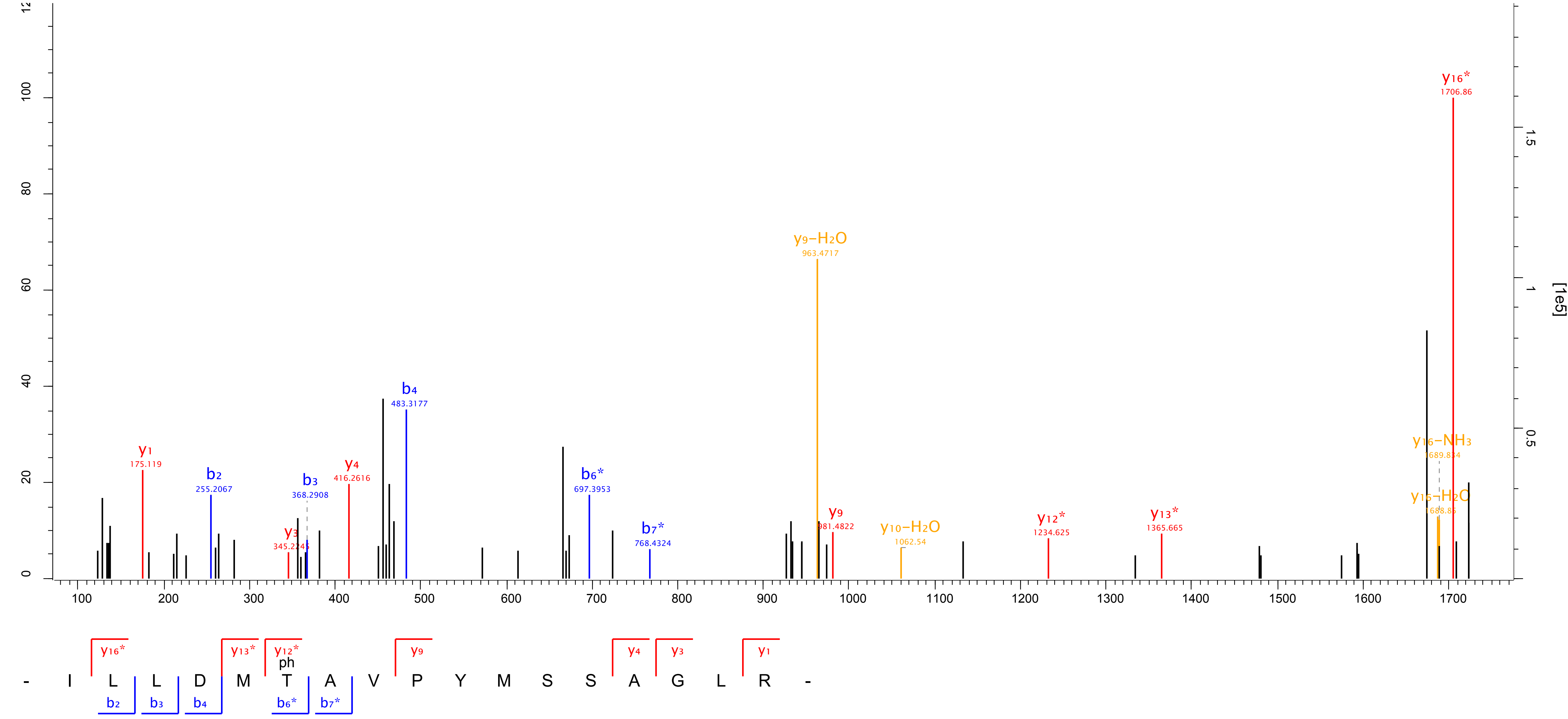

Raw file	Scan	Method	Score	m/z
20150122_PS_Syn_DimethylMix_Rep1_Phospho01	3550	FTMS; HCD	43.84	699.97

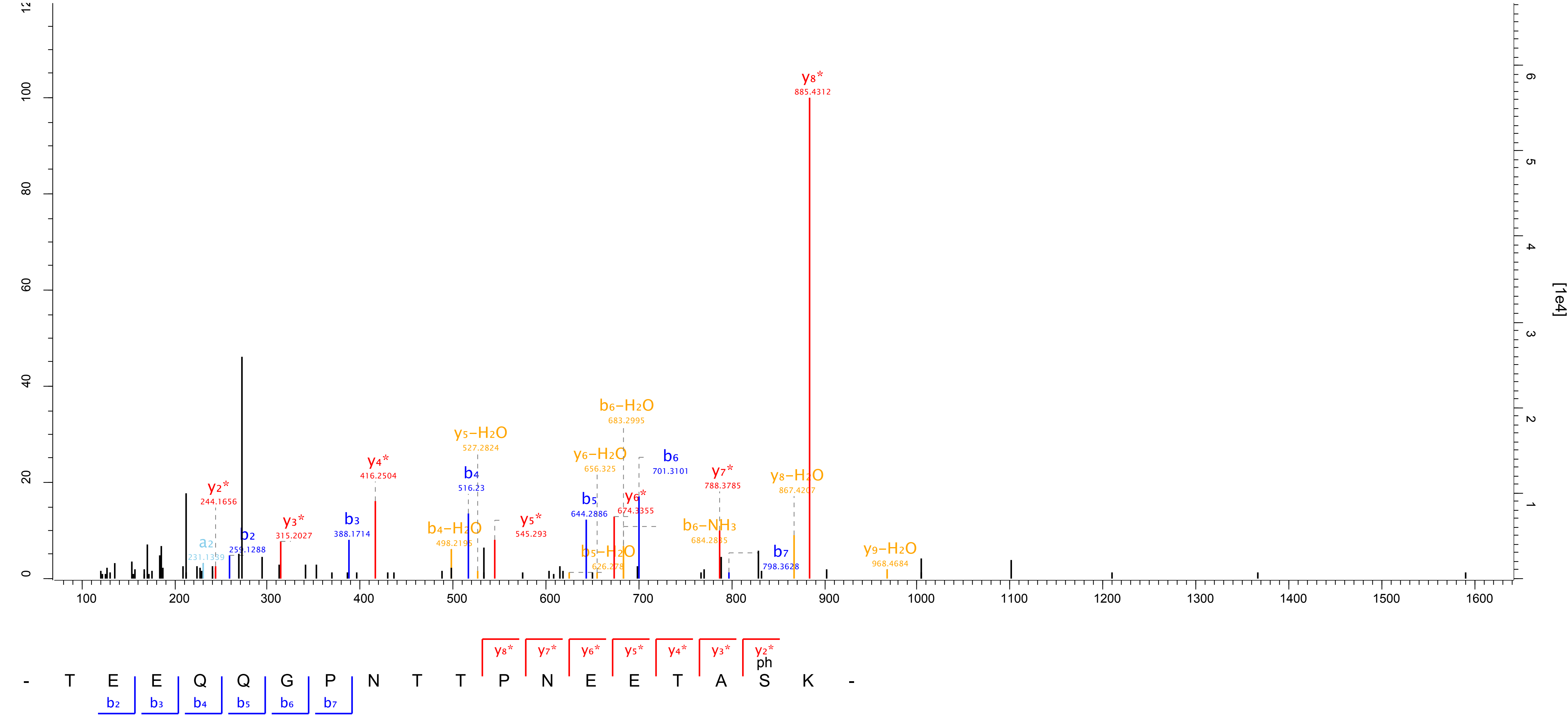

Raw file	Scan	Method	Score	m/z
20141031_PS_Syn6803wt_dimethyl_Mix_Phospho1	6000	FTMS; HCD	46.07	741.68

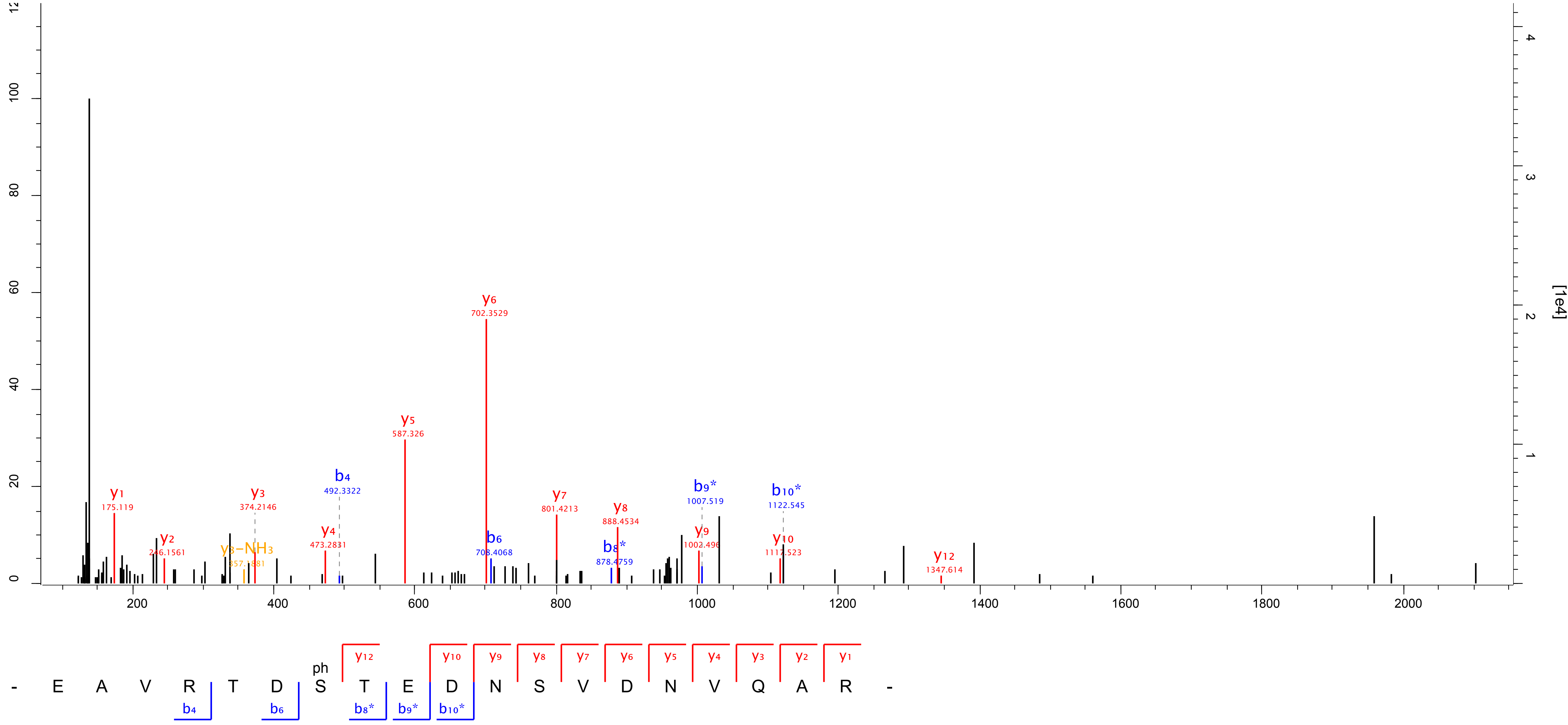

Raw file	Scan	Method	Score	m/z
20141031_PS_Syn6803wt_dimethyl_Mix_Phospho2	6178	FTMS; HCD	82.41	649.98

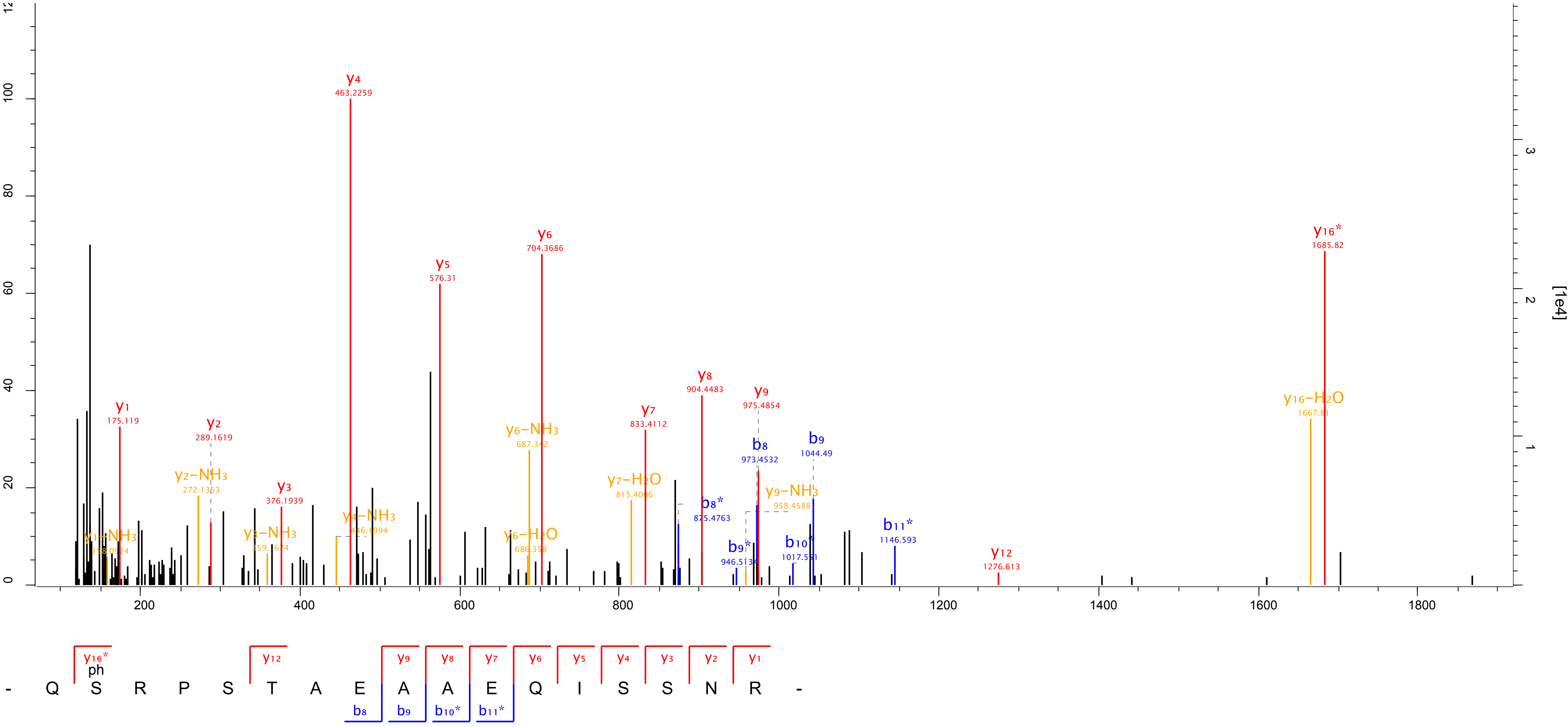

Raw file	Scan	Method	Score	m/z
201417_PS_Syn6803wt_dimethylMix_TiO2_1	6229	FTMS; HCD	106.14	696.86

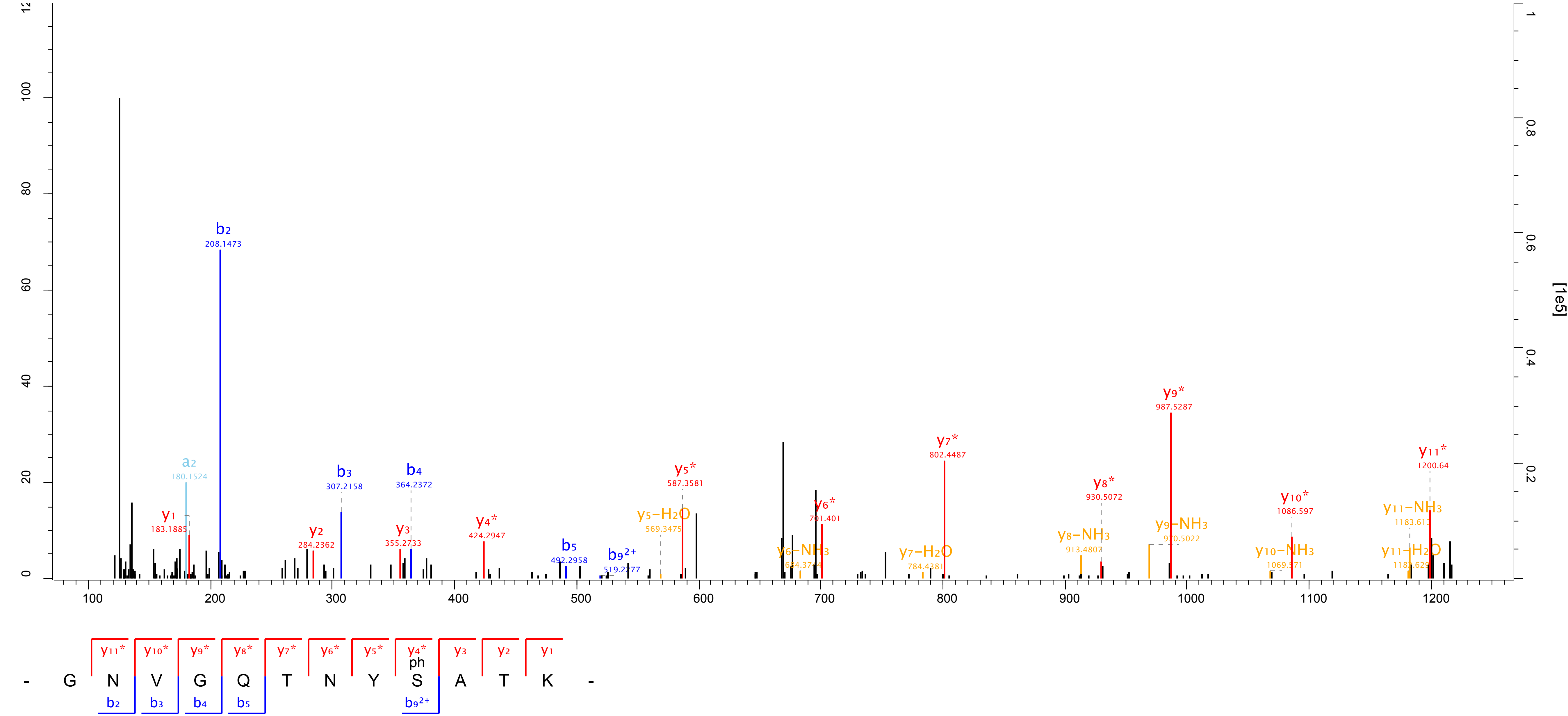

Raw file	Scan	Method	Score	m/z
20141031_PS_Syn6803wt_dimethyl_Mix_Phospho6	6233	FTMS; HCD	66.27	569.27

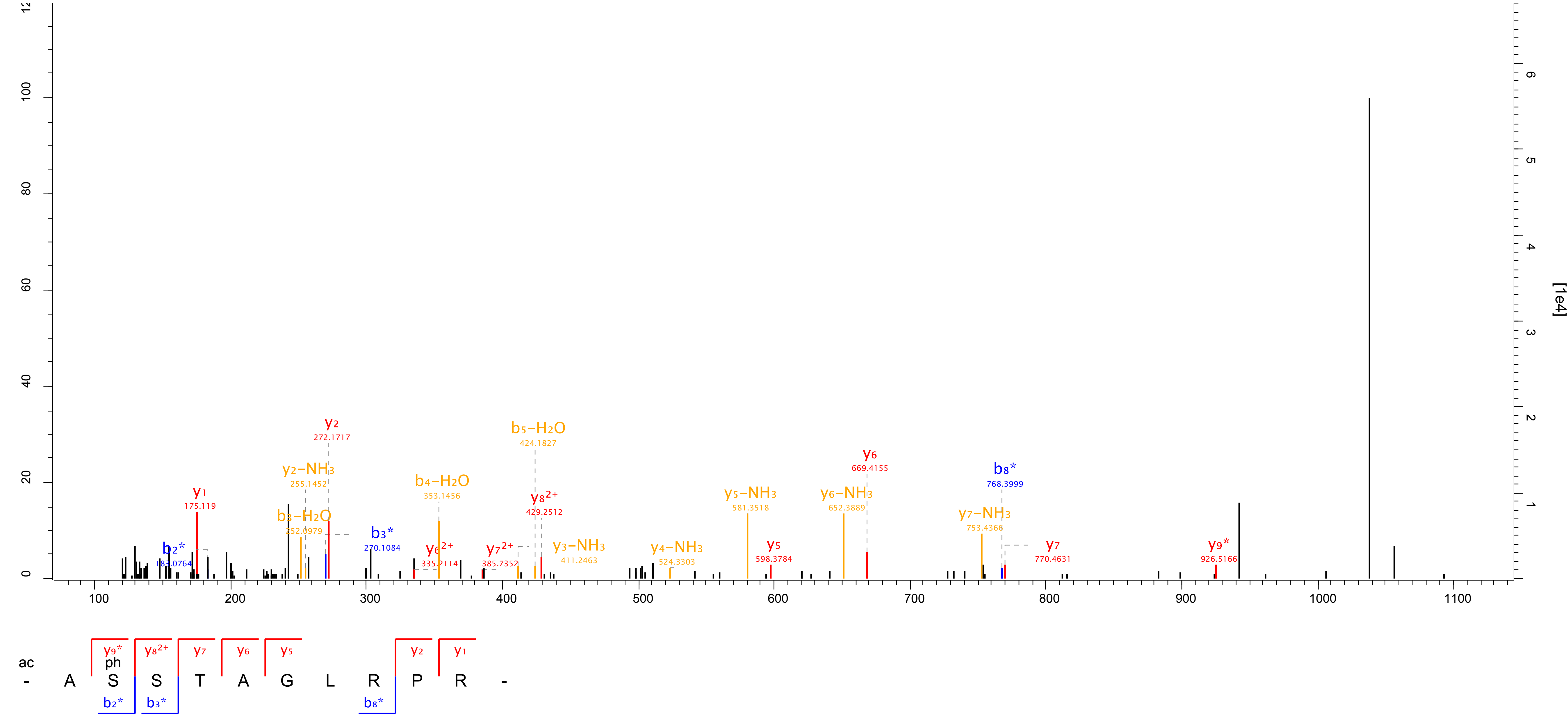

Raw file	Scan	Method	Score	m/z
20150122_PS_Syn_DimethylMix_Rep1_Phospho04	6340	FTMS; HCD	58.17	569.28

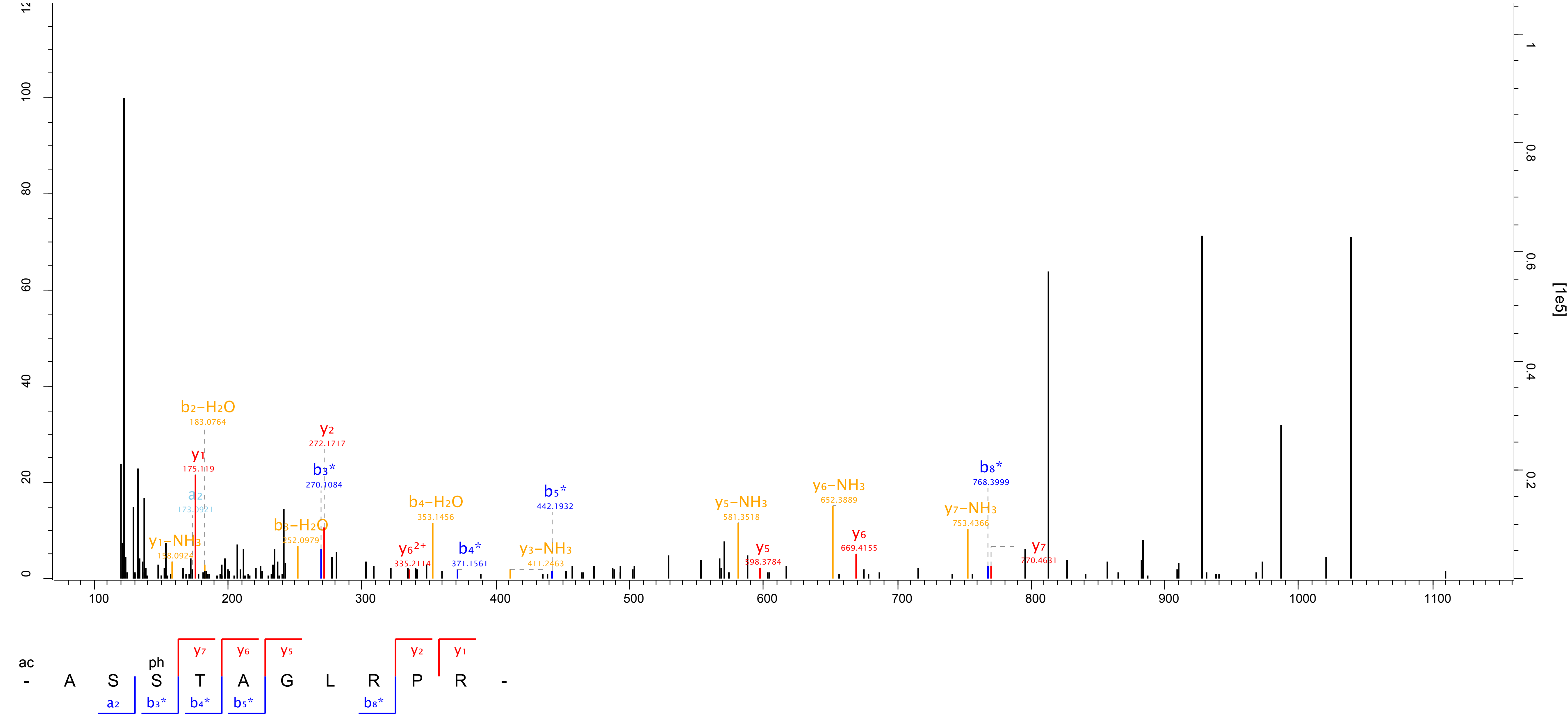

Raw file	Scan	Method	Score	m/z
201417_PS_Syn6803wt_dimethylMix_TiO2_1	6729	FTMS; HCD	69.58	907.43

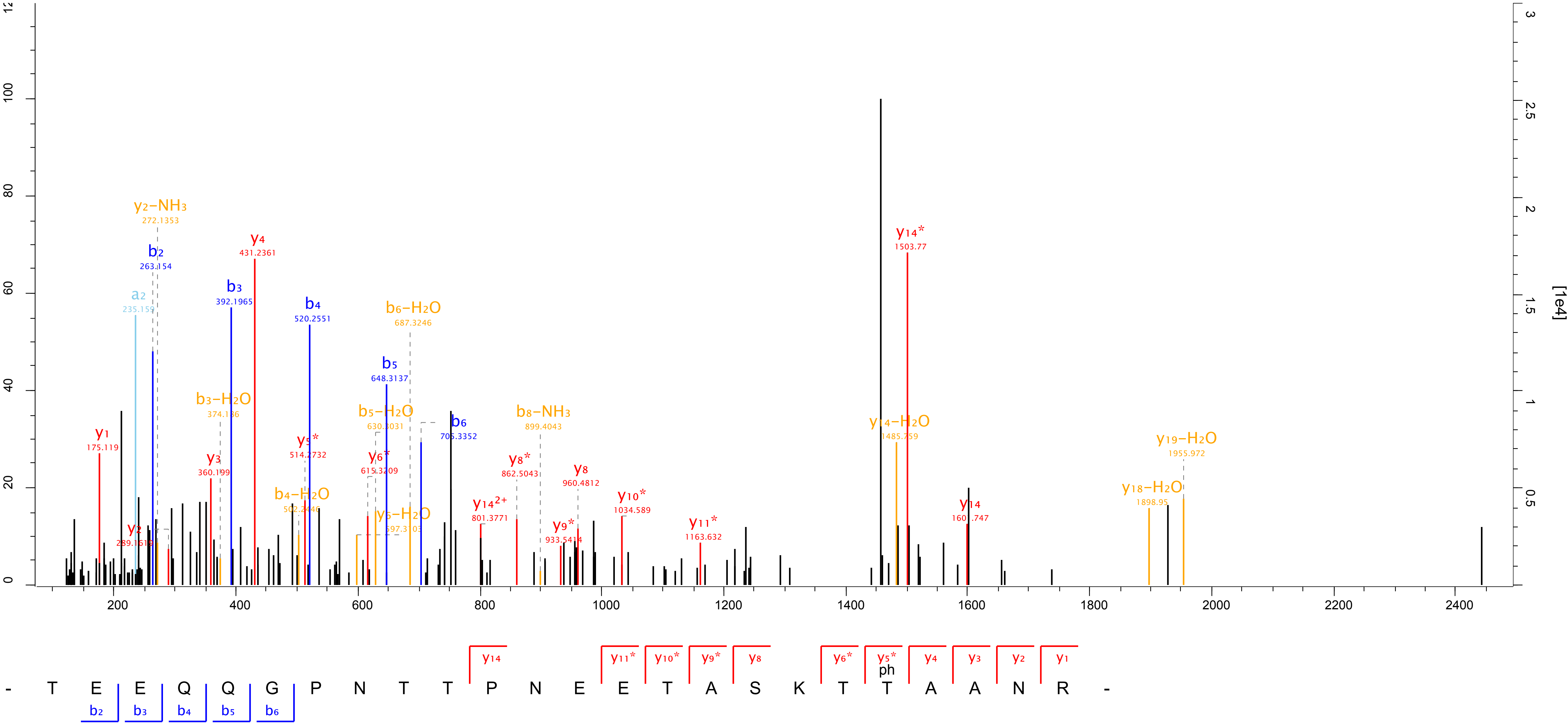

Raw file	Scan	Method	Score	m/z
20141031_PS_Syn6803wt_dimethyl_Mix_Phospho3	7109	FTMS; HCD	87.43	810.89

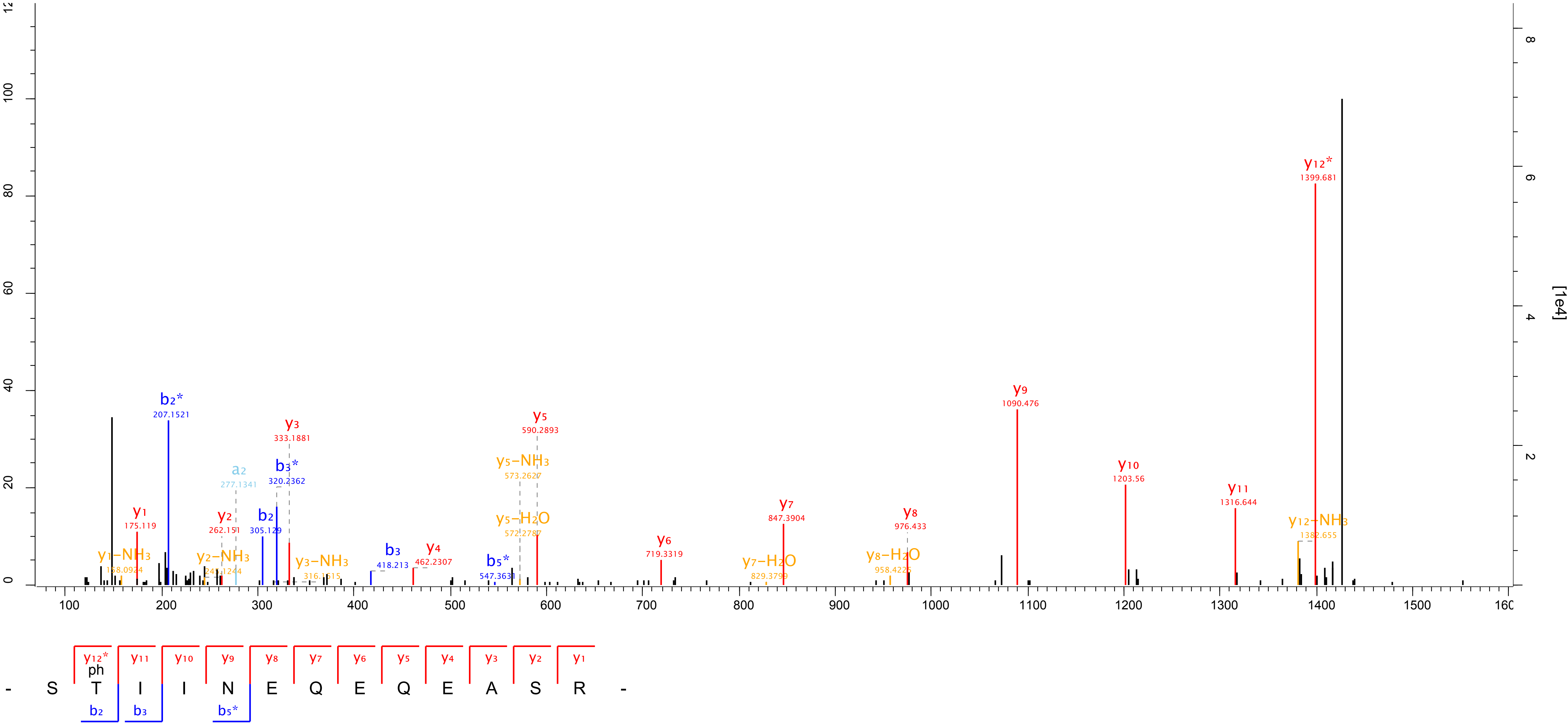

Raw file	Scan	Method	Score	m/z
201417_PS_Syn6803wt_dimethylMix_TiO2_2	7345	FTMS; HCD	53.17	709.39

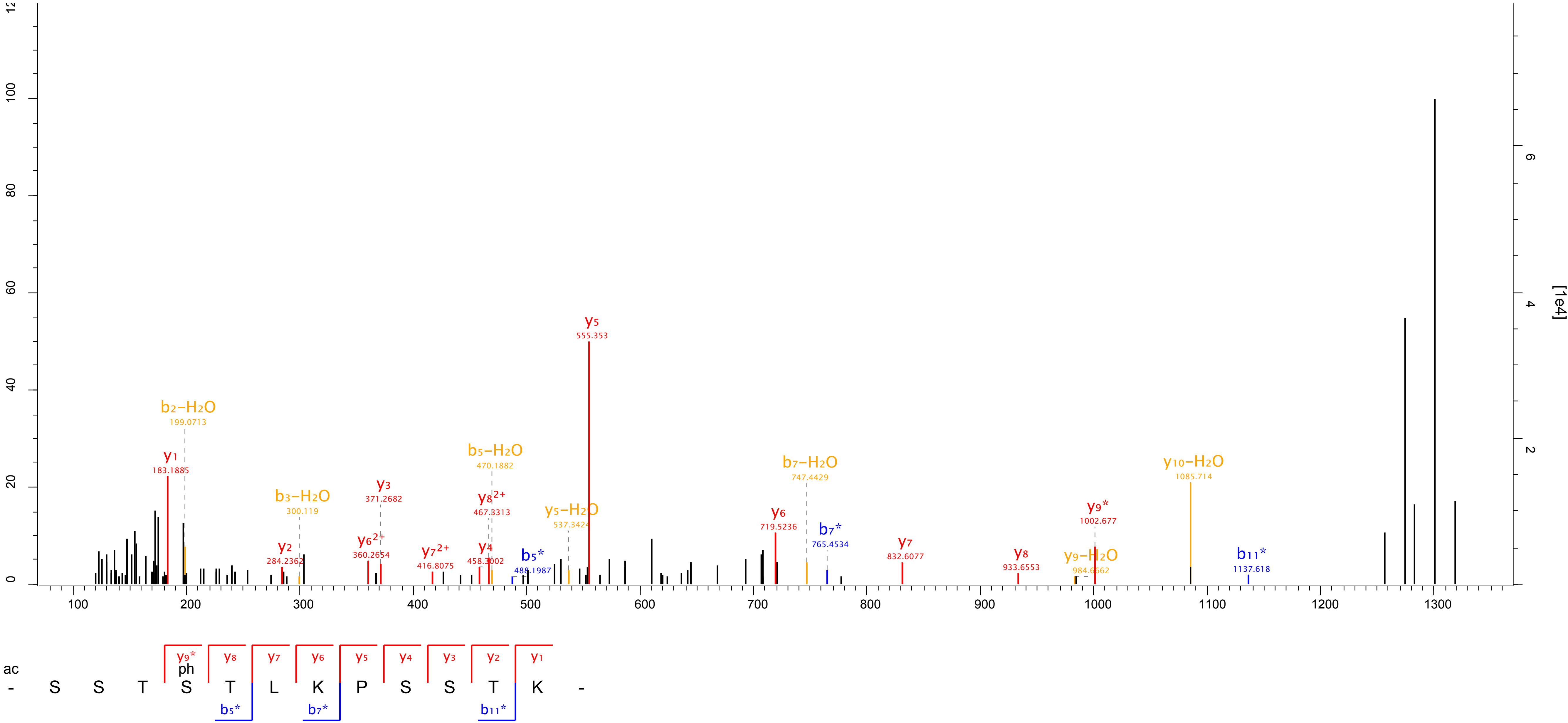

Raw file	Scan	Method	Score	m/z
201417_PS_Syn6803wt_dimethylMix_TiO2_1	7914	FTMS; HCD	88.28	840.88

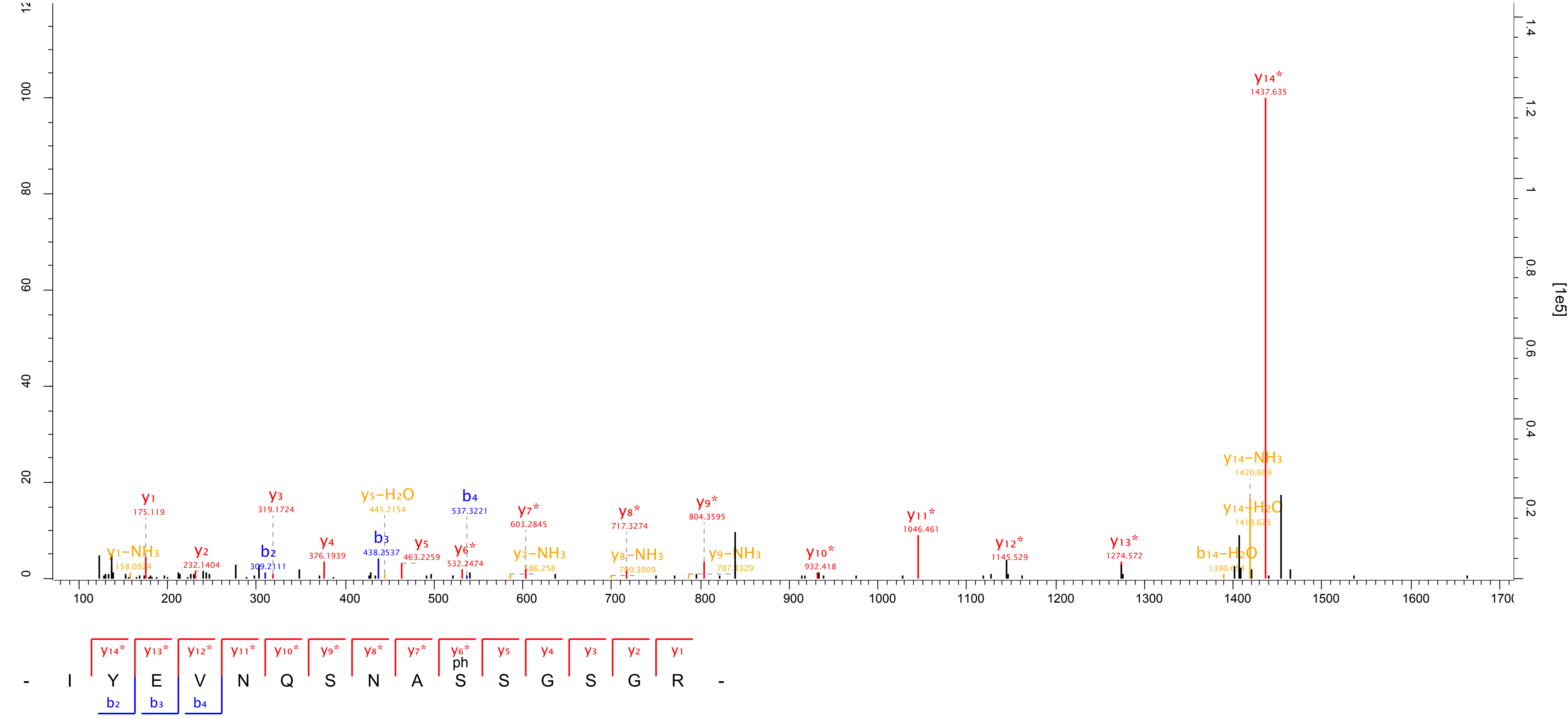

Raw file	Scan	Method	Score	m/z
20141031_PS_Syn6803wt_dimethyl_Mix_Phospho1	8195	FTMS; HCD	48.6	554.78

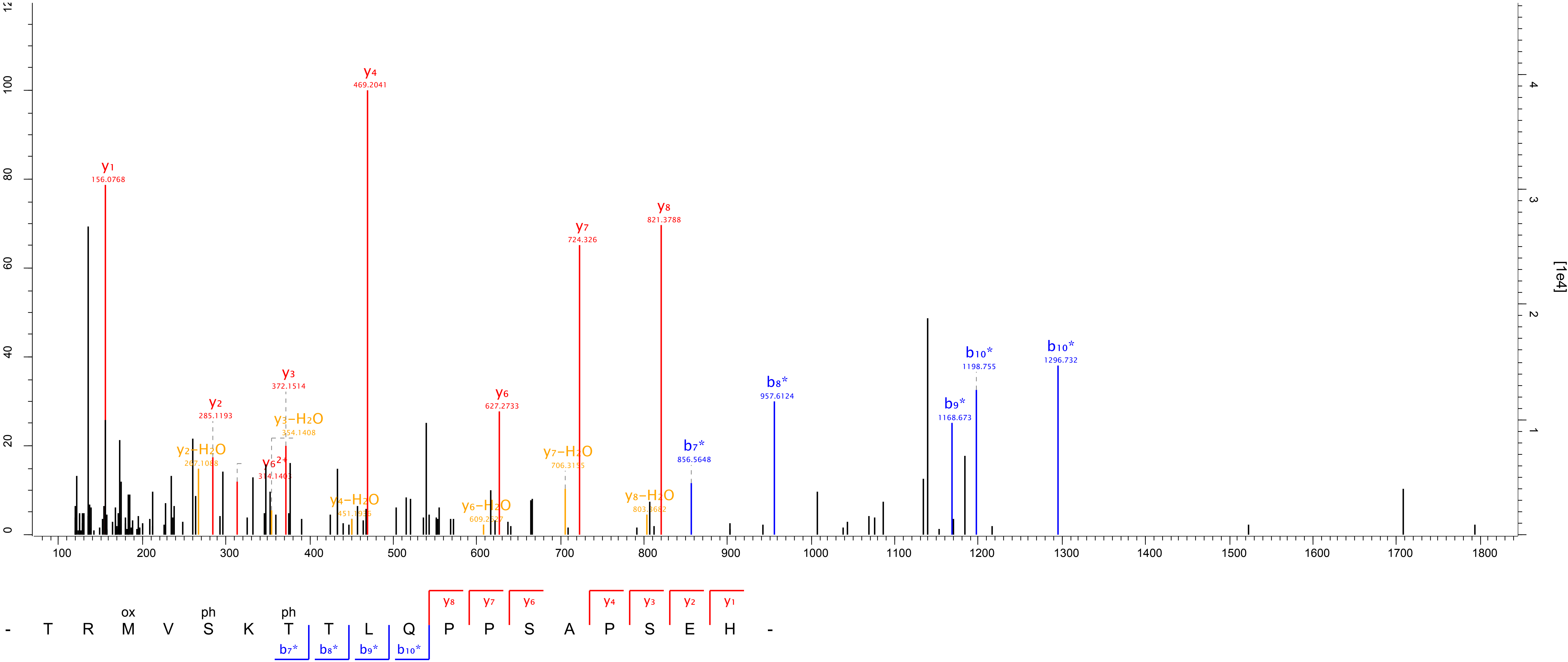

Raw file	Scan	Method	Score	m/z
20150122_PS_Syn_DimethylMix_Rep1_Phospho01	8307	FTMS; HCD	61.28	690.66

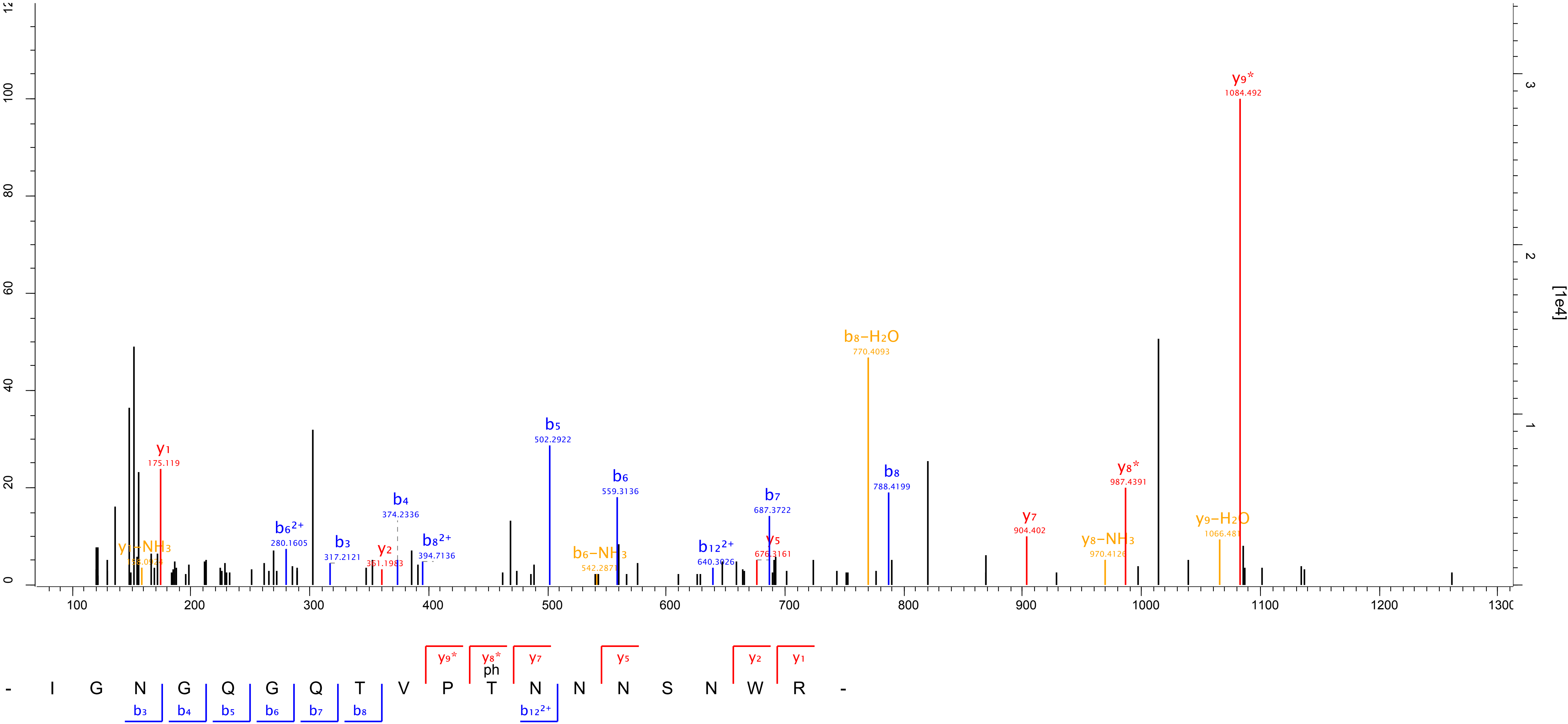

Raw file	Scan	Method	Score	m/z
20150122_PS_Syn_DimethylMix_Rep2_Phospho01	8347	FTMS; HCD	51.47	700.35

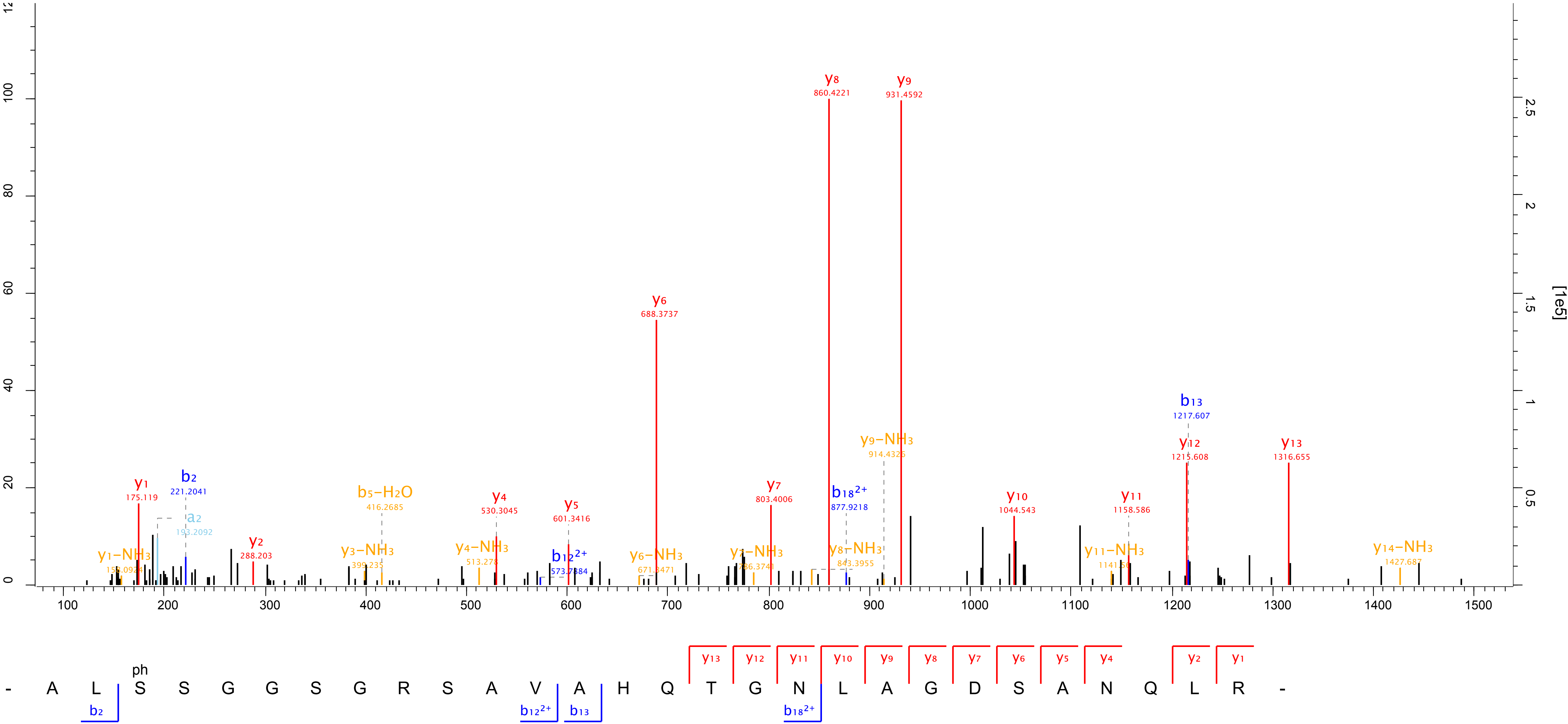

Raw file	Scan	Method	Score	m/z
20150122_PS_Syn_DimethylMix_Rep1_Phospho02	8417	FTMS; HCD	49.56	840.41

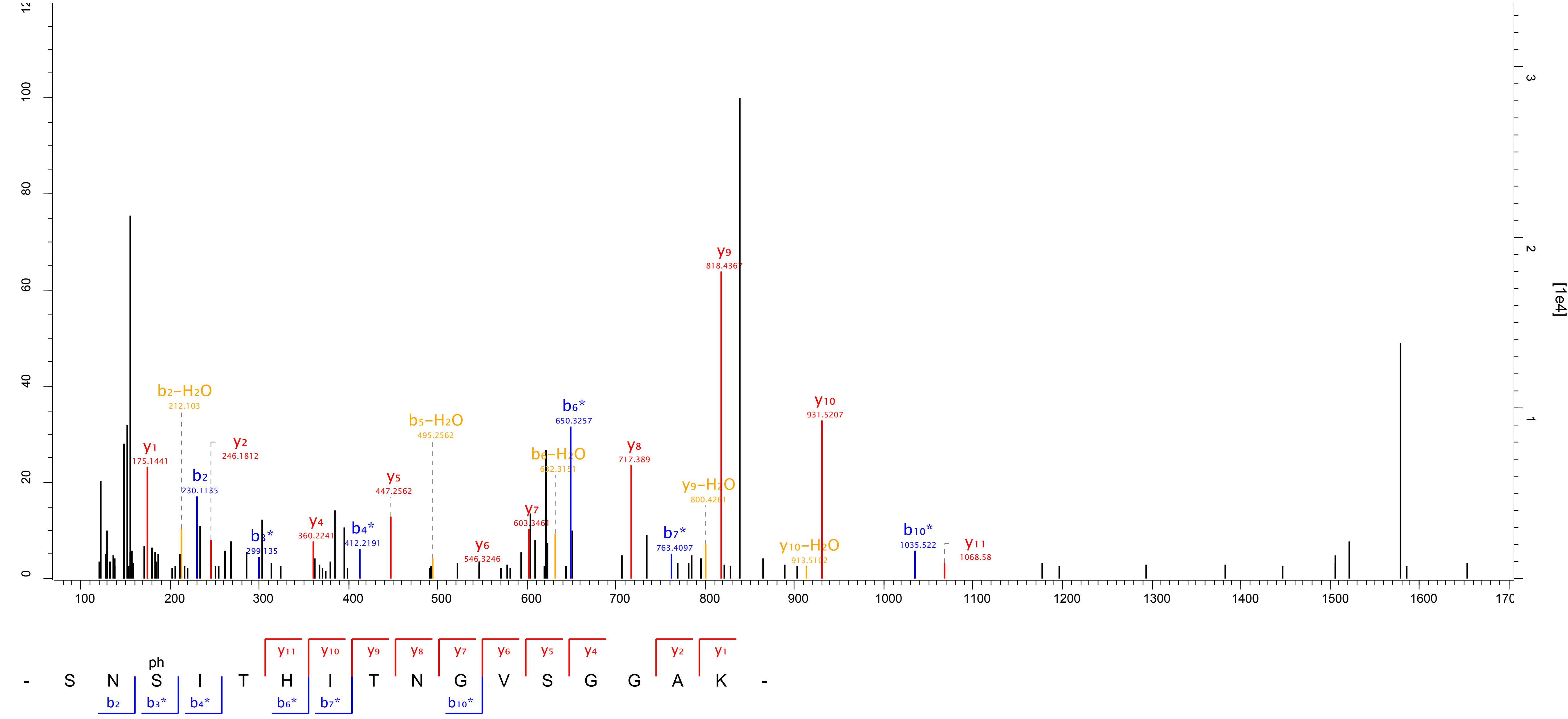

Raw file	Scan	Method	Score	m/z
20141031_PS_Syn6803wt_dimethyl_Mix_Phospho1	8448	FTMS; HCD	82.24	1006.1

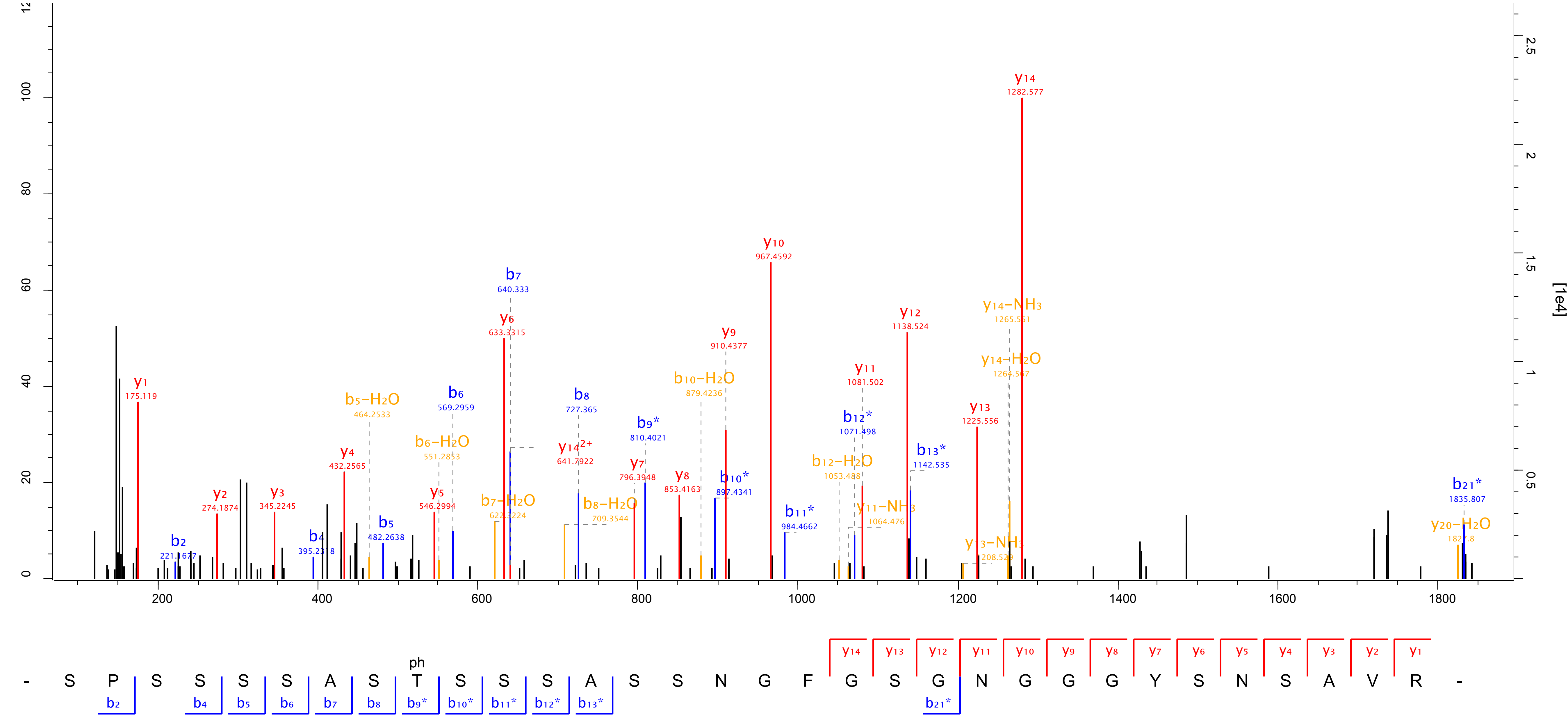

Raw file	Scan	Method	Score	m/z
20150122_PS_Syn_DimethylMix_Rep1_Phospho02	8589	FTMS; HCD	130.2	564.92

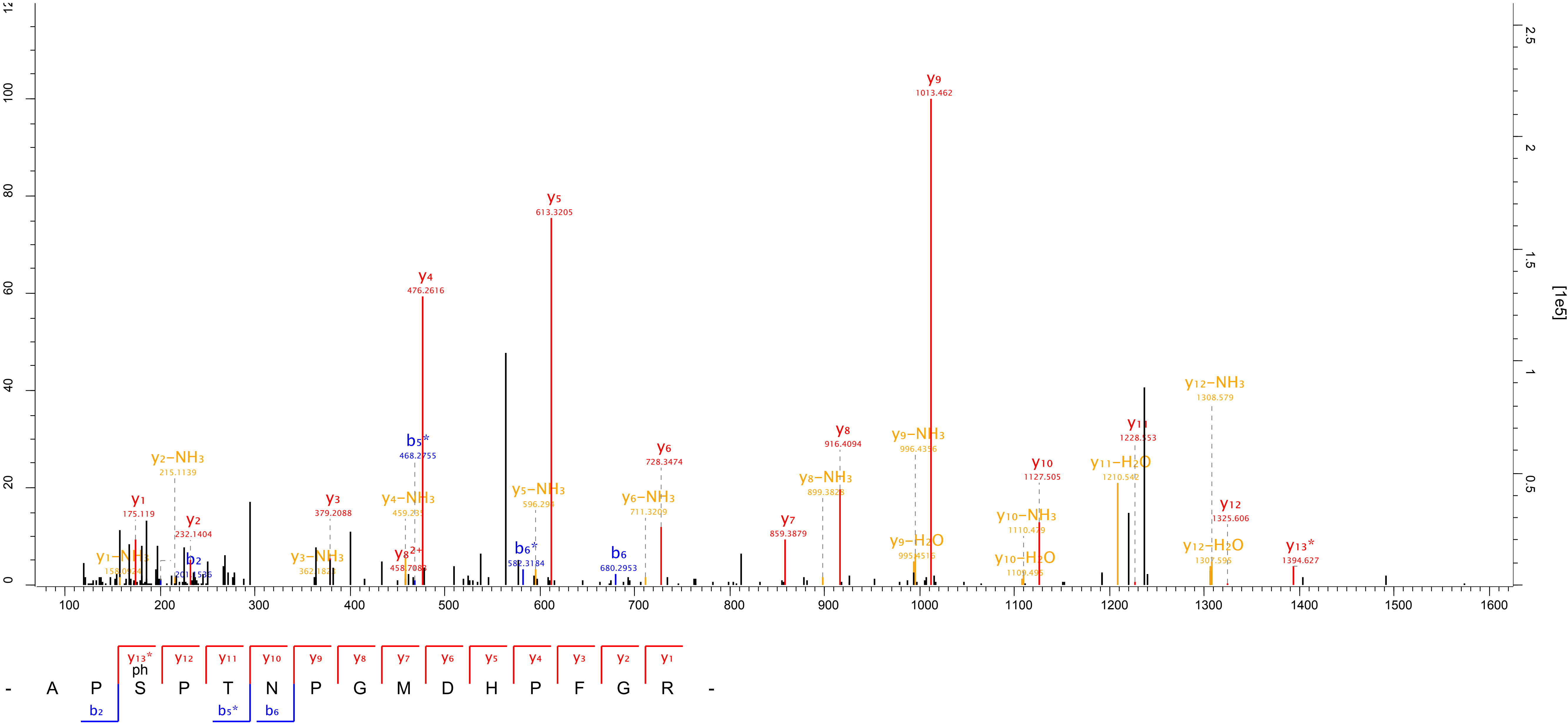

Raw file	Scan	Method	Score	m/z
20141031_PS_Syn6803wt_dimethyl_Mix_Phospho2	8736	FTMS; HCD	88.87	695.03

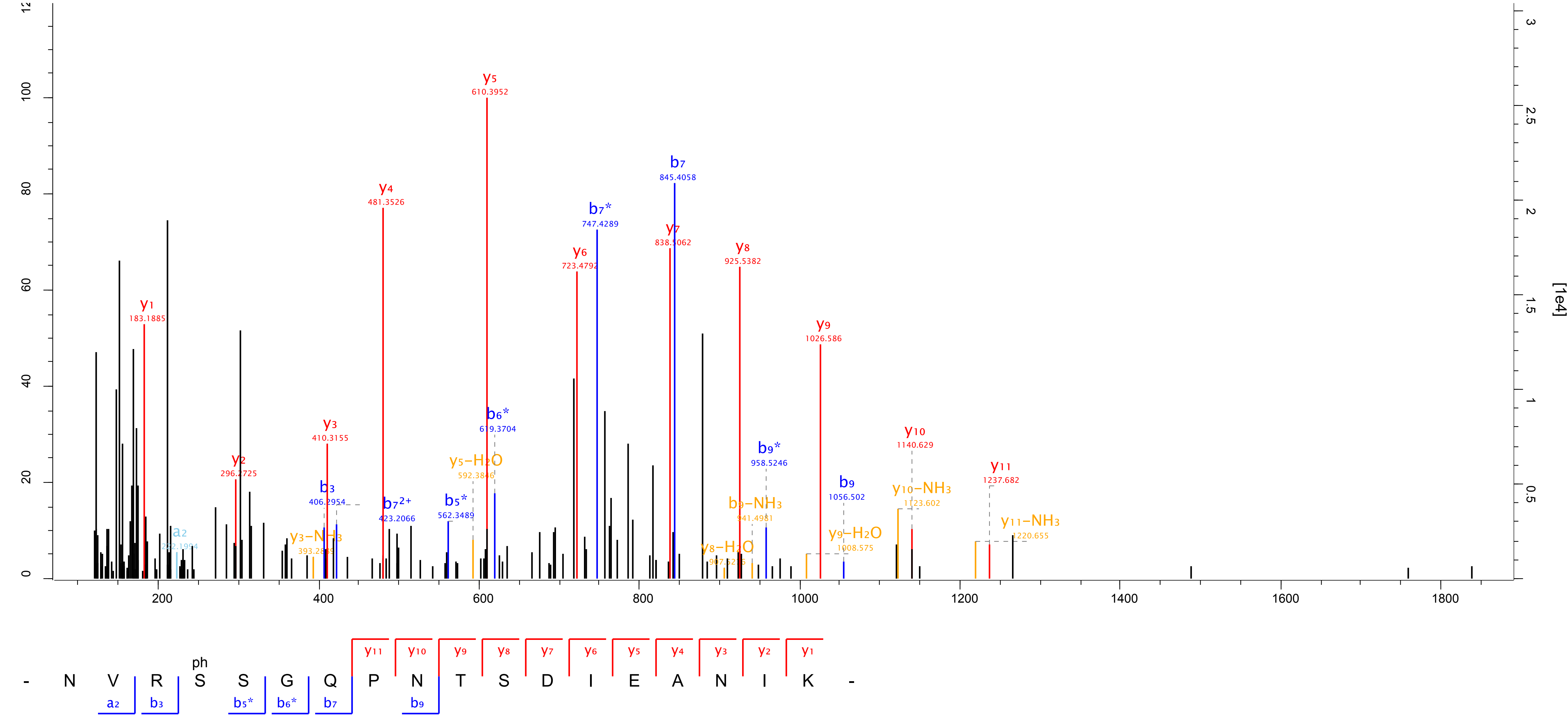

Raw file	Scan	Method	Score	m/z
20141031_PS_Syn6803wt_dimethyl_Mix_Phospho6	8821	FTMS; HCD	44.68	715.7

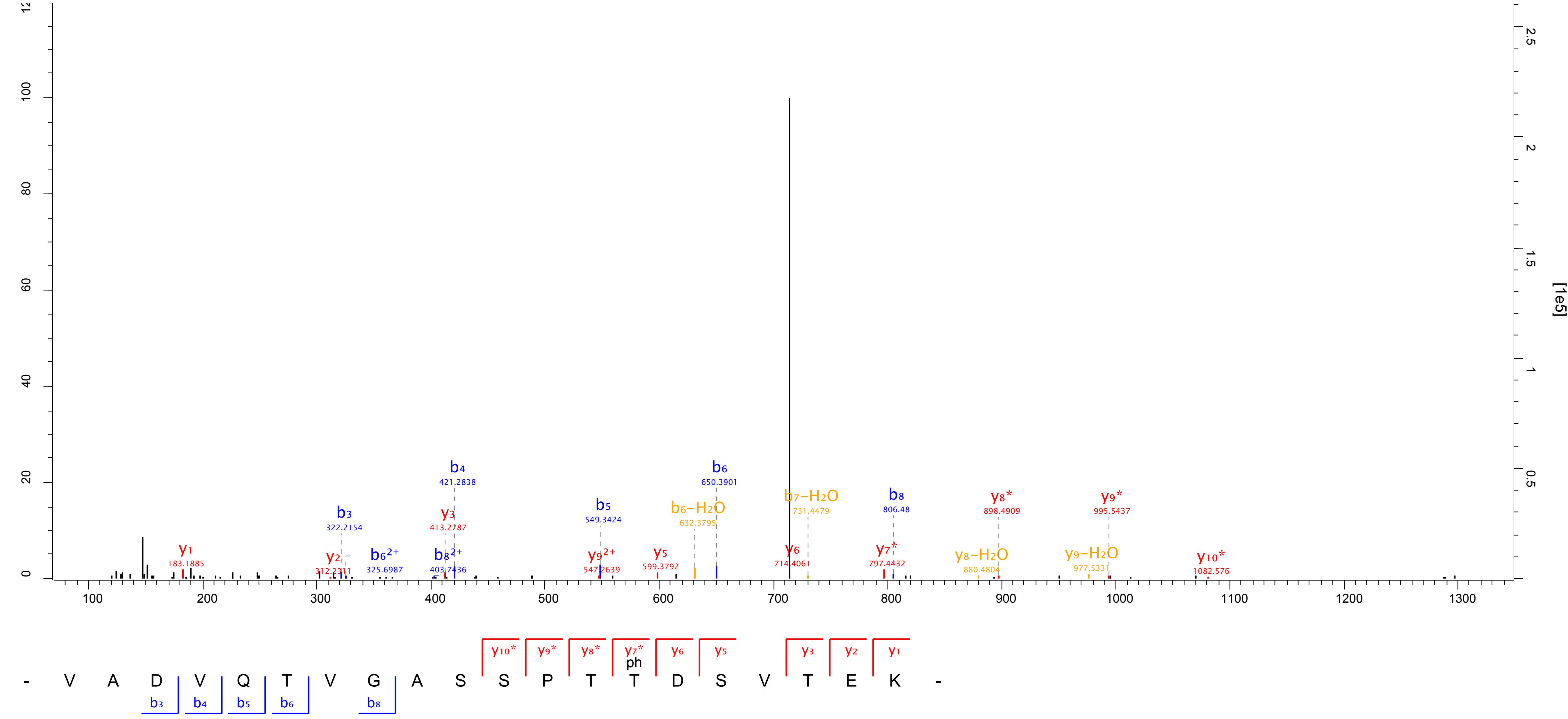

Raw file	Scan	Method	Score	m/z
20150122_PS_Syn_DimethylMix_Rep1_Phospho04	8961	FTMS; HCD	102.69	566.26

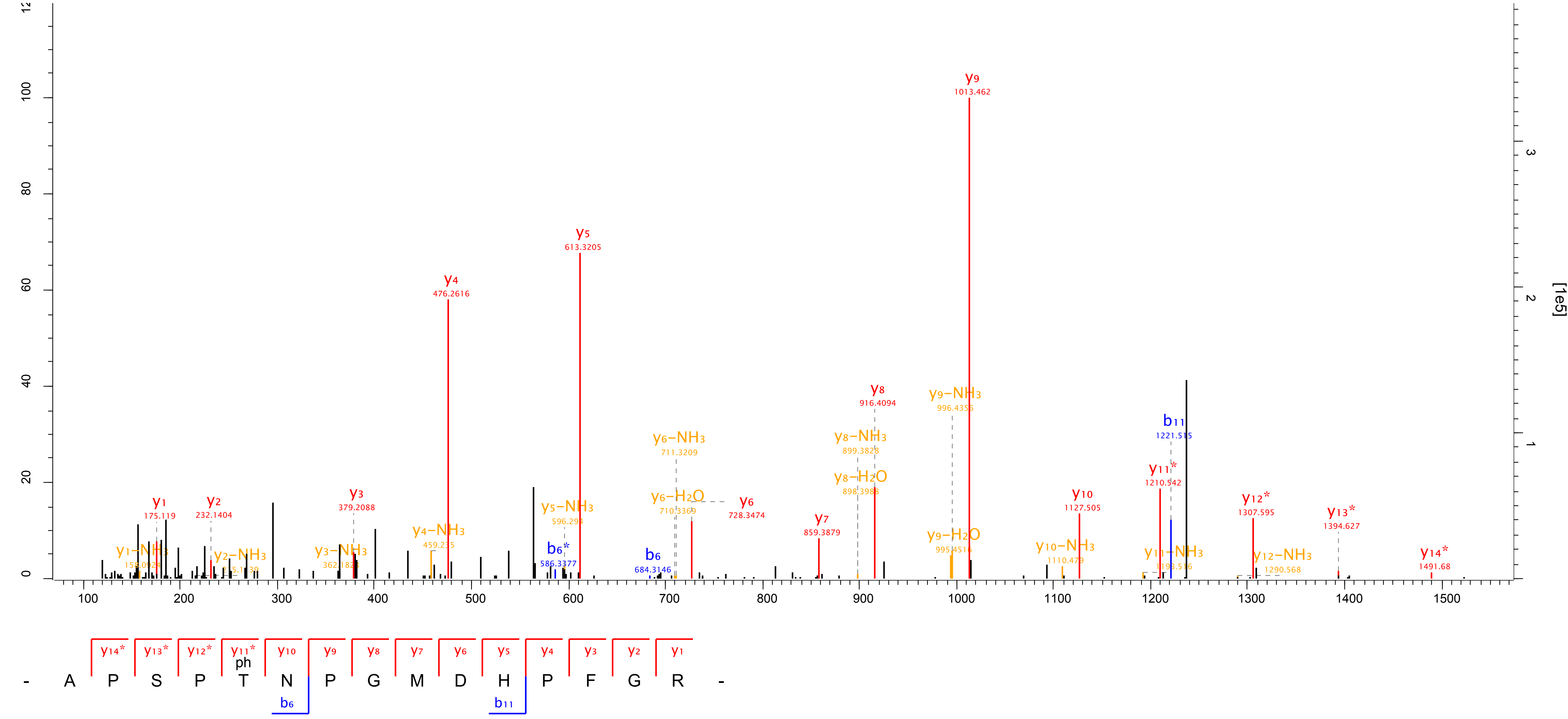

Raw file	Scan	Method	Score	m/z
20141031_PS_Syn6803wt_dimethyl_Mix_Phospho2	9025	FTMS; HCD	70.78	648.96

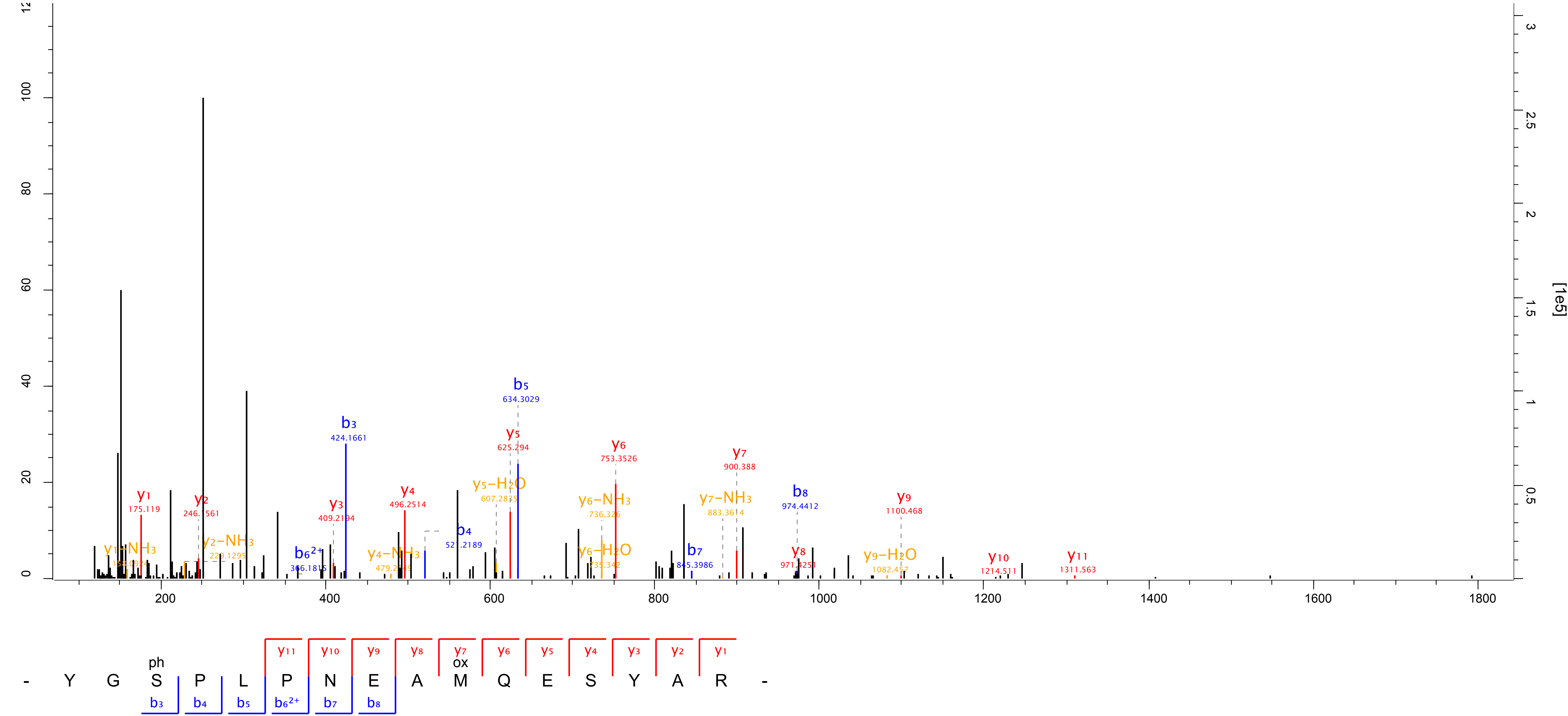

Raw file	Scan	Method	Score	m/z
20141031_PS_Syn6803wt_dimethyl_Mix_Phospho3	9244	FTMS; HCD	60.95	547.59

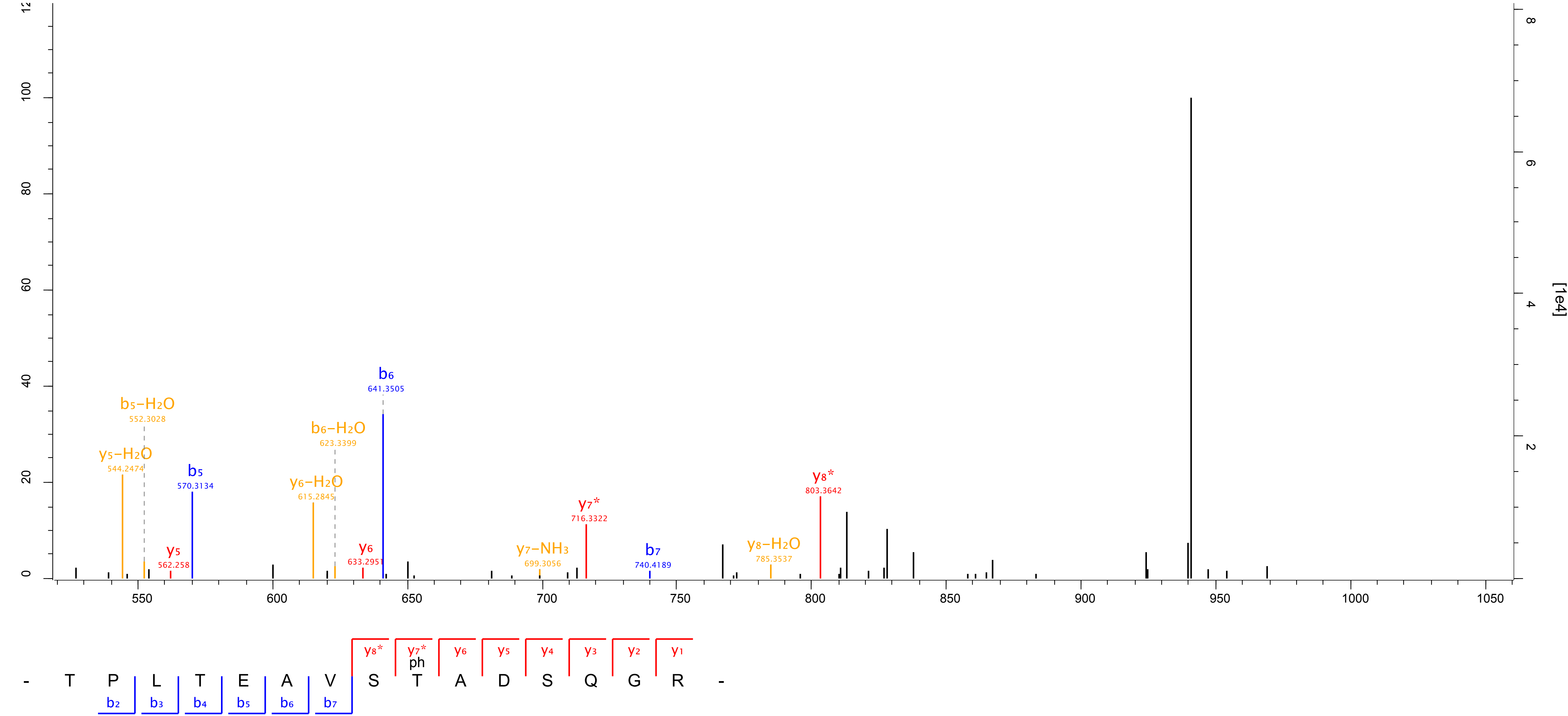

Raw file	Scan	Method	Score	m/z
20141031_PS_Syn6803wt_dimethyl_Mix_Phospho3	9561	FTMS; HCD	125.5	752.38

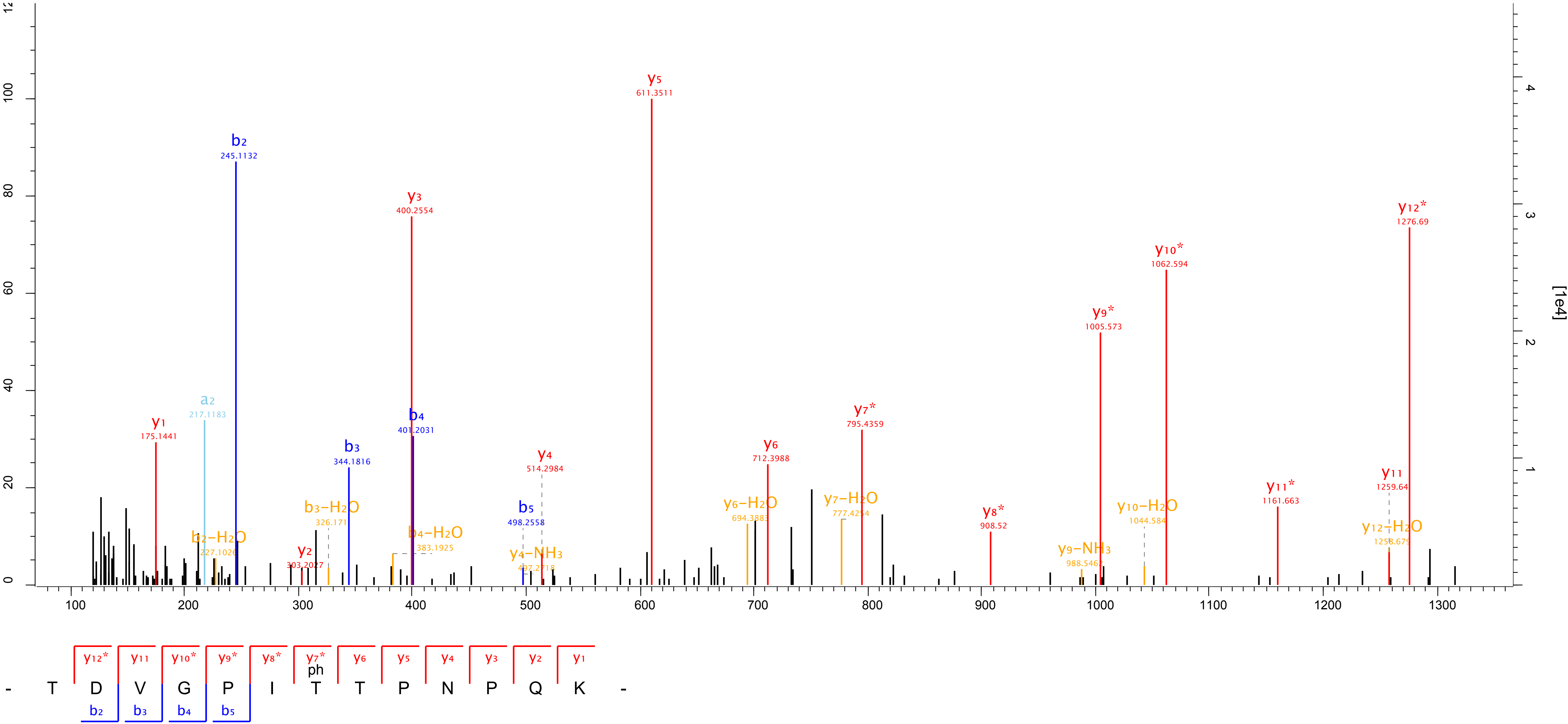

Raw file	Scan	Method	Score	m/z
20141031_PS_Syn6803wt_dimethyl_Mix_Phospho1	9777	FTMS; HCD	45.04	849.75

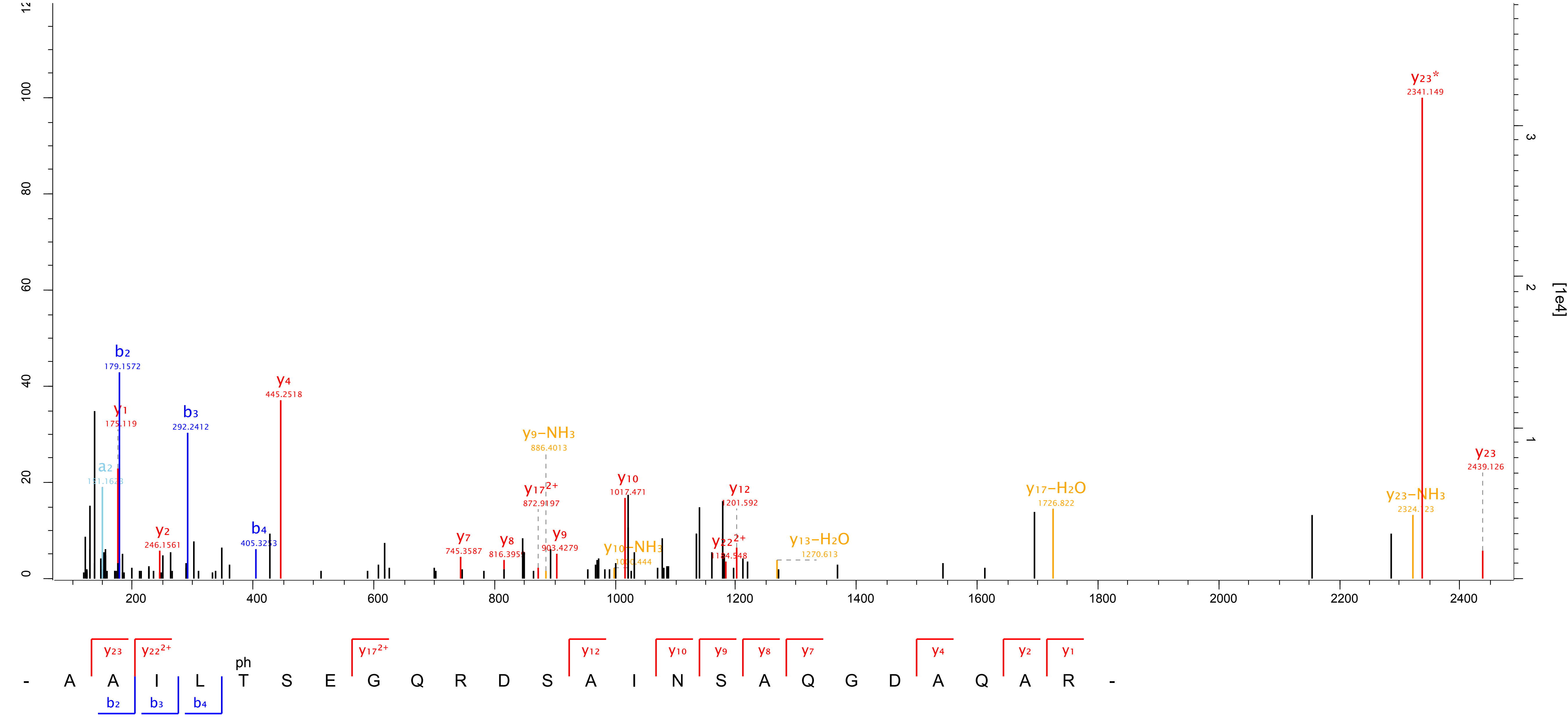

Supplement: Supplementary file 1 [file MS Spectra.PDF › data sheet 1 (1)/MS Spectra.PDF]
